# Bioinspired Synthesis of Twin *abeo*‐Steroids Bufogargarizins A and B via a Divergent Intramolecular Aldol Addition Reaction

**DOI:** 10.1002/anie.202519121

**Published:** 2025-11-19

**Authors:** Zoey J. Surma, Volodymyr Hiiuk, Eugene Zviagin, Yaxin Ouyang, Alex Chacko, Fengrui Qu, Paul M. Zimmerman, Pavel Nagorny

**Affiliations:** ^1^ Department of Chemistry University of Michigan 930 N. University Ave Ann Arbor Michigan 48109 USA

**Keywords:** Epoxide rearrangement, Intramolecular aldol, Natural product, Singlet oxygen, Steroid, Synthesis

## Abstract

This manuscript describes a concise bioinspired synthesis of twin *abeo*‐steroids bufogargarizins A and B with an unusual [7.5.6.5] and [5.7.6.5] skeletons and a highly oxidized D‐ring with an α‐pyrone substituent from (+)‐methyl estrone. The described synthetic approach features ozonolytic cleavage of the Δ^5,10^‐alkene of an easily available estrone derivative, followed by a bioinspired regio‐ and stereoselective intramolecular aldol addition reaction that produced the desired bufogargarizins A and B precursors with the [7.5.6.5] and [5.7.6.5] skeletons in 27% and 63% yields, correspondingly. This work provides direct evidence that proves the biosynthetic hypothesis for the first time, as prior synthetic studies suggest that only bufogargarizin B could be formed via an intramolecular aldol reaction. The subsequent installation of the β17‐pyrone moiety along with the β14, β15‐epoxide and β16‐acetoxy group was found to be challenging due to the instability of the α‐pyrone moiety to a range of basic, reductive, and oxidative conditions. To address this challenge, we have developed a singlet oxygen‐based oxidation method that results in a streamlined installation of the D‐ring oxidation and stereochemistry from a Δ^14,16^‐diene precursor. This enabled the completion of the syntheses of bufogargarizins A and B in 19 steps (LLS) and 0.36 and 1.5% overall yields, respectively.

## Graphical abstract

**Twin natural products**. This manuscript describes a concise biomimetic synthesis of twin *abeo*‐steroids bufogargarizins A and B with an unusual [7.5.6.5] and [5.7.6.5] skeletons in 19 steps (LLS). The described synthetic approach features the application of a bioinspired regiodivergent intramolecular aldol addition reaction and a selective singlet oxygen‐based oxidation followed by an endoperoxide transposition to streamline the installation of the D‐ring oxidation, which has enabled the concise synthesis of bufogargarizins A and B.

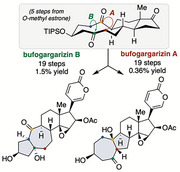

## Introduction

Steroids represent an important and diverse family of natural products that play an imperative role in regulation and signaling in eukaryotic organisms. In humans, steroids primarily serve as chemical messengers (hormones) that regulate many important functions, including metabolic, immune, and reproductive functions. The importance of steroidal natural products cannot be overstated, and steroidal motifs are present in a variety of FDA‐approved drugs.^[^ ^1^ ^]^

**Figure 1:**
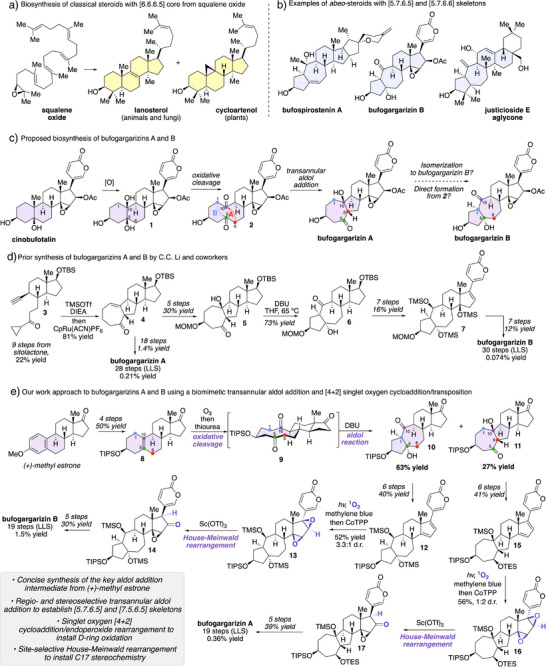
Bufogargarizins A and B biosynthesis, prior synthetic approach, and summary of this work.

In eukaryotic organisms, steroids are produced by the cyclization of a triterpene squalene that leads to either lanosterol or cycloartenol intermediates with the [6.6.6.5] tetracyclic core that is common in the steroid family (Figure 1a). For almost a century, organic chemists have tried to emulate nature and generate these polycyclic steroid skeletons. These studies have led to a plethora of creative approaches to complex steroidal natural products and fueled the development of steroid‐based therapeutic agents, many of which have become an integral part of everyday life.^[^ ^2^, ^3^, ^4^, ^5^, ^6^, ^7^, ^8^ ^]^ However, the structures of steroidal natural products are not limited to the [6.6.6.5] skeleton as biosynthesized steroids may subsequently undergo various cleavage and rearrangement events. These rearrangement events culminate in the production of a variety of secondary steroid derivatives that possess diverse and challenging to synthesize structural features. Among various steroidal derivatives, *abeo*‐steroids with unusual ring systems such as bufospirostenin A or bufogargarizins A and B (Figure 1b) have recently attracted significant attention.^[^ ^9^, ^10^, ^11^, ^12^, ^13^ ^]^ Thus, recent studies by Li,^[^ ^9^ ^]^ Gui^[^ ^10^, ^12^ ^]^ and Huang and Yang^[^ ^11^ ^]^ resulted in successful synthetic approaches to bufospirostenin A. In addition, recent work by Li and coworkers described the synthesis of [7.5.6.5] core of bufogargarizin C.^[^ ^14^ ^]^ Following this work, Li and coworkers developed a route to both bufogargarizins A and B in 2023 (Figure 1c and Scheme S2).^[^ ^15^ ^]^ In these studies, Li's group developed a 9‐step synthesis to intermediate **3** from sitolactone. Compound **3** was subjected to an intramolecular Ru(II)‐catalyzed [5 + 2] cycloaddition reaction that resulted in intermediate **4**, which contains the [7.5.6.5] skeleton. This intermediate was then converted to bufogargarizin A in a total of 28 steps (LLS) and 0.21% overall yield. Additionally, compound **5** was reassembled under basic conditions to intermediate **6** which possesses the desired [5.7.6.5] skeleton. Compound **6** was elaborated to bufogargarizin B in a total of 30 steps (LLS) and 0.074% overall yield.

Our approach based on the production of intermediate **9** is in line with the biosynthetic proposal by Ye and coworkers^[^ ^16^, ^17^, ^18^ ^]^ who isolated and characterized bufogargarizins A and B from the *Bufo bufo gargarizans* toad skin glands. It was hypothesized that both bufogargarizins A and B could be derived from the same intermediate **2** by a transannular aldol addition reaction. Intermediate **2** was proposed to result from an oxidative cleavage of the 5,10‐diol moiety of **1** that was hypothesized to arise from cinobufotalin, a bufodienolide that was isolated alongside A and B. However, this biosynthetic hypothesis was not completely consistent with the observations by the Li group (Figure 1d) that may suggest an alternative mechanism involving the isomerization of bufogargarizin A to form bufogargarizin B. The described work provides direct evidence of the biosynthetic hypothesis, as we demonstrate that both bufogargarizin A and B precursors **11** and **10** can be formed simultaneously via an intramolecular aldol reaction.

Our group has a long‐standing interest in synthesis and medicinal chemistry exploration of cardiotonic steroids,^[^ ^19^, ^20^, ^21^, ^22^, ^23^, ^24^, ^25^, ^26^ ^]^ including α‐pyrone‐containing‐bufadienolides such as cinobufotalin (Figure 1c).^[^ ^27^ ^]^ Based on the biosynthetic proposal of Ye and computed energies of possible aldol products (vide infra), we proposed that both bufogargarizins A and B could be derived from precursor **8**, which is available in 6 steps from (+)‐methyl estrone (Figure 1e).^[^ ^28^ ^]^ This manuscript describes our efforts that culminated in the biomimetic synthesis of both bufogargarizins A and B from (+)‐methyl estrone in 19 steps (LLS) for both natural products with total yields of 0.36% and 1.5%, respectively. These studies feature the development of a highly regio‐ and stereoselective cyclization of **9** to provide intermediate **11** and **10** with the [7.5.6.5] and [5.7.6.5] skeletons of bufogargarizins A and B, which is in line with the proposed biosynthetic hypothesis. The subsequent elaboration of **11** and **10** into bufogargarizins A and B was found to be challenging due to the sensitivity of the α‐pyrone moiety to various basic, acidic, reductive, and oxidative conditions that were required to install the 14β,15β‐epoxide, 16β‐acetoxy group, and 17β‐stereocenter present in both these natural products.

To address these challenges, we have implemented our recently developed method that is based on the chemoselective singlet oxygen‐based oxidation of the Δ^14,16^‐diene moiety of **15** and **12** in the presence of the α‐pyrone ring.^[^ ^27^ ^]^ The diastereoselectivity of this oxidation is strongly dependent on the configuration of the AB‐ring system of the diene precursor, with the bufogargarizin B skeleton favoring the desired β‐selectivity, and the bufogargarizin A skeleton favoring the undesired α‐*bis*‐epoxide. Following these observations, we were able to develop a one‐pot [4 + 2] cycloaddition/endoperoxide rearrangement reaction to generate intermediates **13** and **16**. These β‐*bis*‐epoxides were subjected to a House‐Meinwald rearrangement to form keto‐epoxide intermediates **14** and **17**, which, after a series of redox manipulations and a selective deprotection sequence, led to the synthesis of twin bufodienolide natural products bufogargarizins A and B.

## Results and Discussion

Based on the biosynthetic hypothesis developed by Ye and coworkers,^[^ ^16^, ^17^, ^18^ ^]^ we propose the biomimetic transannular aldol addition approach toward the synthesis of bufogargarizins A and B (*cf*. Figure 1).^[^ ^29^, ^30^, ^31^, ^32^, ^33^, ^34^, ^35^, ^36^, ^37^, ^38^, ^39^, ^40^, ^41^, ^42^ ^]^ We anticipated that these twin *abeo*‐steroids could be accessed from intermediates **10** and **11**, which could be derived from the protected *nor*‐steroid **8** that could be obtained by the reduction of (+)‐methyl estrone.

**Scheme 1:**
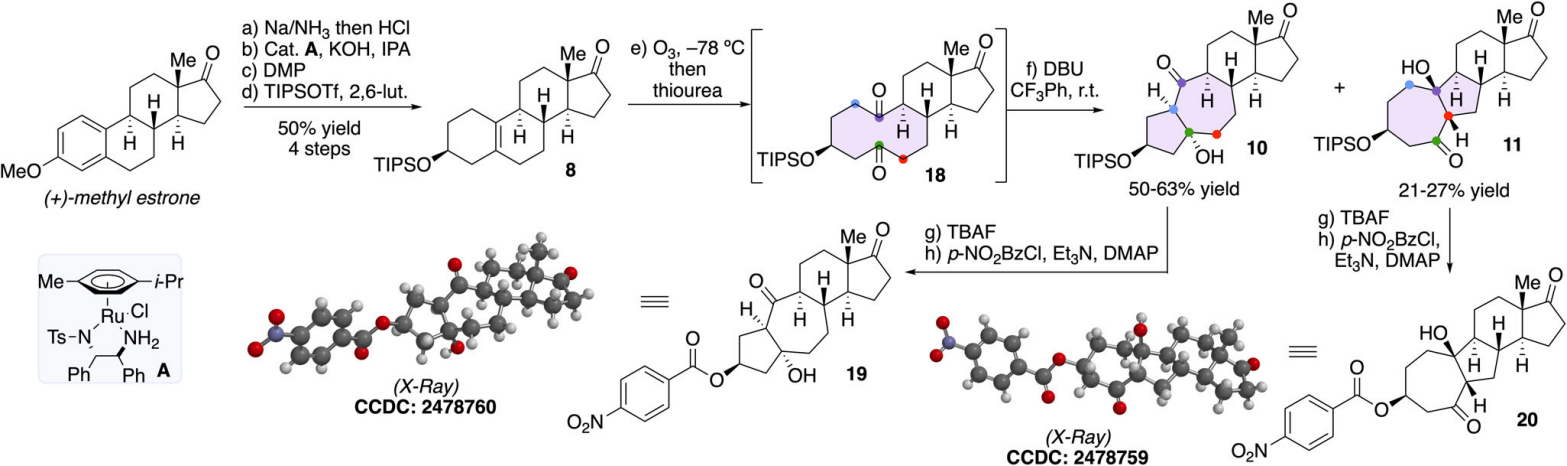
Synthesis of bufogargarizins A and B precursors **10** and **11** from (+)‐methyl estrone.

Our synthetic studies commenced with the Birch reduction of the commercially available (+)‐methyl estrone (Scheme 1). This reduction proceeded quantitatively using a previously published protocol (Na in NH_3_ and IPA in THF), and the resultant methyl enol ether was cleaved in situ using hydrochloric acid in methanol to provide the corresponding dearomatized product in 92% yield. The C3 ketone moiety of this product was subjected to diastereoselective Noyori transfer hydrogenation conditions with catalyst **A** (25 mol%), which resulted in the desired β‐C3‐alcohol configuration in 90% yield and 10:1 d.r.^[^ ^43^ ^]^ This product was subsequently subjected to a C17‐selective oxidation with Dess‐Martin Periodinane (DMP) (76% yield) and TIPS protection of the β‐C3‐alcohol (88% yield), resulting in the desired intermediate **8** in 50% yield (4 steps) from (+)‐methyl estrone. Alternatively, **8** could be generated from the Birch reduction product by a 4‐step sequence involving hydrolysis with oxalic acid and a DMP oxidation (78% yield over 3 steps), followed by a selective β‐C3‐reduction with *L*‐selectride, and protection of the resultant β‐C3‐alcohol with TIPSOTf in 78% yield (3:1 d.r. β/α) over 2 steps (*cf*. Scheme S6). With the robust access to **8**, our following studies focused on developing a biomimetic one‐pot oxidative cleavage/base‐promoted cyclization that would enable converting **8** into the transannular aldol products **10** and **11**. The oxidation step was accomplished through the ozonolysis of the Δ^5,10^‐alkene in the presence of sodium bicarbonate, followed by a reductive workup with thiourea (*cf*. Tables S1 and S3).^[^ ^44^ ^]^ This gave rise to the intermediate diketone **18**, which was subsequently subjected to various cyclization conditions with the objective to generate both bufogargarizin B core **10** and bufogargarizin A core **11** (*cf*. Table 1 and Table S1).

**Table 1:** Optimization of the one‐pot oxidative cleavage/aldol sequence leading to bufogargarizin B precursor **10** and bufogargarizin A precursor **11**.

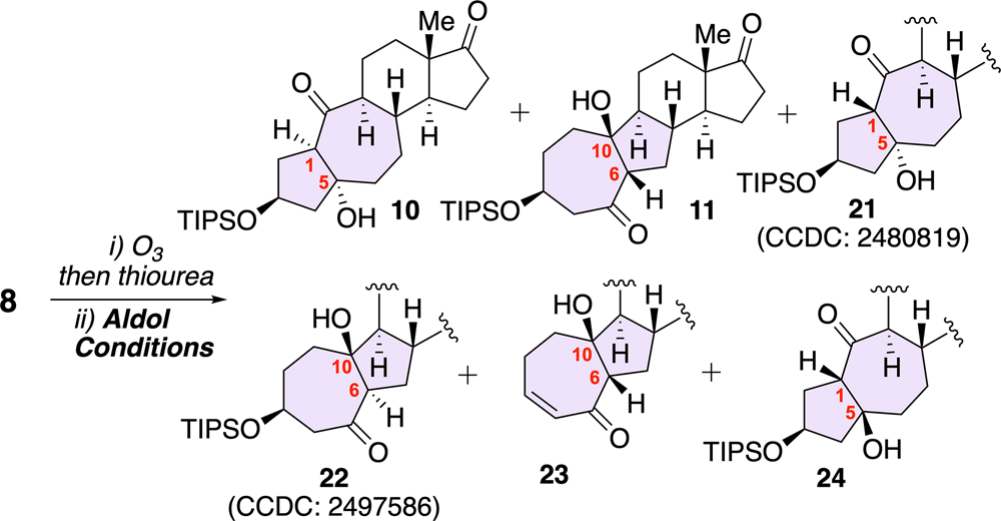		
Entry	Aldol conditions	Observed products (% yield)^b^ ^)^
1^c)^	Basic Al_2_O_3_ (54 equiv) DCM, r.t.	–
2^c)^	SiO_2_ (90 equiv) DCM, r.t.	–
3^c)^	*p*‐TsOH (0.24 equiv) DCM, r.t.	–
4^c)^	KO*t*‐Bu (2.0 equiv) THF, r.t.	–
5^d)^	DBU (15 equiv) THF, reflux, 11 h	**10** (38%), **11** (7%) **21** (13%), **22** (3%), **23**, **24**
6	DBU (10 equiv) THF, reflux, 1 h	**10** (31%), **11** (15%) **21** (18%), **22** (6%), **23**, **24**
7	DBU (1.0 equiv) THF, r.t., 3 h	**10** (41%), **11** (20%) **21** (10%), **22** (3%)
8	DBU (0.5 equiv) DCM, r.t., 1.5 h	**10** (45%), **11** (27%) **21** (6%), **22** (1%)
9	DBN (0.5 equiv) DCM, 0 °C to r.t., 5 h	**10** (44%), **11** (22%) **21** (6%), **22** (3%)
10	TBD (0.5 equiv) DCM, 0 °C to r.t., 5 h	**10** (31%), **11** (8%) **21** (8%), **22** (5%)
11	DBU (0.5 equiv) PhCF_3_, r.t., 1.5 h	**10** (63%), **11** (23%) **21** (5%), **22** (1%)

^a)^ All optimization experiments were performed on a 0.16–0.18 mmol scale of compound **8**. Refer to Table S1 for the full list of evaluated conditions.

^b)^ Isolation yields after column chromatography on silica gel. The formation of trace amounts of **24** for entries 5 and 6 was observed by ^1^H NMR analysis of the crude reaction mixture.

^c)^ Aldol products were not observed under these conditions.

^d)^ The structures of **21** and **22** were assigned by the X‐ray crystallographic analysis (CCDC: 2480819 and 2497586). The structure of **24** was assigned by X‐ray crystallographic analysis of its desilylated derivative (CCDC: 2480818), and the configuration of **23** was assigned by comparison with the published NMR data by Li and coworkers.^(^ ^Ref. 14^ ^)^

While diketone **18** was stable to isolation and exhaustive purification, the typical experiments were performed with the crude ozonolysis mixtures containing unpurified **18**. Our initial attempts to promote transannular aldolization of **18** with known reagents such as basic alumina (entry 1),^[^ ^39^, ^40^ ^]^ silica gel (entry 2), or *p*‐toluenesulfonic acid (entry 3) did not provide identifiable aldol products. Treating **18** with potassium *tert*‐butoxide (2 equiv) in THF at r.t. also did not lead to observable quantities of the aldol addition products (entry 4). Our subsequent efforts turned to evaluating mild amine bases such as DBU (entries 5–8, 11). When **18** was subjected to the conditions that were previously utilized by Li and coworkers for the isomerization of bufogargarizin A intermediate **5** into bufogargarizin B intermediate **6** (Figure 1),^[^ ^15^ ^]^ a mixture of products containing bufogargarizin B skeleton **10** as the major product was observed (entry 5). While the reported isomerization of **5** to **6** proceeded selectively in 73% yield, compound **10** was isolated in only 38% yield along with the bufogargarizin A skeleton **11** (7%), the bufogargarizin B intermediates **21** (13%) and **24**, corresponding to the C1‐epimer and C1, C5‐double epimer of **10**, respectively, the C6‐epimer of bufogargarizin A precursor **22** (3%), and the elimination product **23**. The structures of compounds **21** (CCDC 2480819), **22** (CCDC 2497586), and **24** (via the X‐ray analysis of its desilylated derivative, CCDC 2480818) were confirmed by X‐ray crystallographic analysis, and the configuration of **23** was assigned by comparison with the published NMR data (*cf*. Figures S1 and S2).^[^ ^15^ ^]^ In our initial attempts to optimize this reaction, we shortened the reaction time to 1 h and reduced the amount of base to 10 equiv (entry 6). Remarkably, these conditions led to a different distribution of **10** and **11** (31% and 15%, respectively), suggesting that **11** isomerizes to **10** upon prolonged exposure to DBU in refluxing THF. Further lowering the amount of DBU to 1.0 equiv and running the reaction at room temperature in THF resulted in increased amounts of **10** (41%) and **11** (20%), while the undesired diastereomers **21** and **22** were formed in lower amounts (10% and 3%, correspondingly) and no side product **23** was observed. Interestingly, lowering the DBU loading from 10 to 1 equiv in THF at r.t. (Table S1) had only a minor effect on the aldol product distribution, indicating that the reaction is primarily influenced by factors such as temperature and substrate concentration rather than the amount of base. Further optimization involved evaluation of various solvents and helped to identify DCM (entry 8) as the best solvent for the formation of bufogargarizin A aldol product **11** (27% yield) and trifluorotoluene (entry 11) as the best solvent to form bufogargarizin B aldol product **10** (63% yield). Our further attempts to improve the formation of **10** or **11** by using DBN, TBD, or TMG instead of DBU (entries 9, 10, and Table S1) did not lead to improvements in selectivity or yield.

The optimized ozonolysis/aldolization conditions that emerged from the studies above were applied to the preparation of key intermediates **10** and **11** (Scheme 1). These conditions typically led to the formation of bufogargarizin B precursor **10** (50%–63% yield) along with 21%–27% of bufogargarizin A precursor **11** (*cf*. Table S3 for the description of reproducibility and scale of these experiments). To confirm the regio‐ and stereoselectivity of the aldol step, both **10** and **11** were converted to the corresponding *p*‐nitrobenzoate derivatives **19** and **20**, the structure of which was further validated by single‐crystal X‐ray crystallographic analysis.

**Scheme 2:**
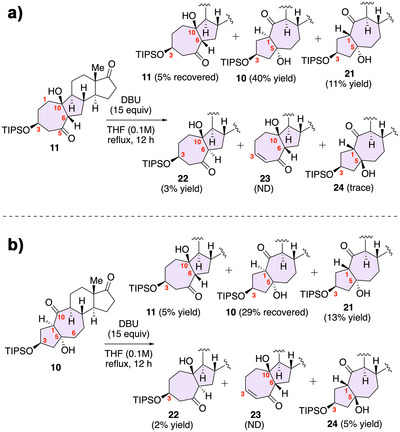
Probing the reversibility of the transannular aldol addition reaction leading to **10** and **11**.

Our studies presented in Table 1 indicated that aldol reaction of **18** is temperature dependent, and a different product distribution is observed in refluxing THF. These results imply that the conditions that involve a large excess of DBU and higher reaction temperature (i.e., entry 5, Table 1) are taking place under thermodynamic control and result in an equilibrated mixture of isomers. At the same time, the reaction proceeding at room temperature with catalytic DBU (i.e., entry 11, Table 1) is facilitated under kinetic conditions. To further probe this hypothesis, we subjected pure aldol adducts **10** and **11** to 15 equiv of DBU in refluxing THF for 12 h (*cf*. Scheme 2). Indeed, the isomerization of **11** (Scheme 2a) under more concentrated conditions (0.10 M in THF) resulted in a mixture comprised of aldol products—**10** (40% yield), **11** (5% recovered yield), **21** (11% yield), and **22** (3% yield)—along with trace amounts of **24**, elimination, and decomposition products. This product distribution is similar to that observed for the cyclization of diketone **18** under the identical conditions (i.e., entry 5, Table 1), supporting the kinetic origin of **11**. Subjecting bufogargarizin B precursor **10** to the same conditions (Scheme 2b) resulted in a similar distribution of products; except for an increased formation of the aldol product **24** (5% yield). Remarkably, performing the reaction of **10** under more dilute conditions (0.02 M in THF) significantly suppressed decomposition, resulting in predominant recovery of the starting material (70%) with only trace elimination products, while an equilibrated mixture of **11** (4% yield), **21** (16%), and **22** (2%) was still observed (*cf*. Table S2).

**Figure 2:**
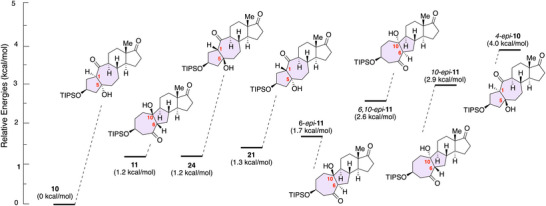
Computed Gibbs energies of aldol products relative to the most stable isomer **10**. Geometries were obtained from B3LYP/6–311 + G** with energies from wB97X‐D/cc‐pVTZ/SMD(THF). See Supporting Information for full computational details.

These observations are in great agreement with the computed Gibbs energies of **10**, **11**, and their six diastereomers (Figure 2). Thus, the product containing the bufogargarizin B skeleton **10** was found to be the most thermodynamically stable. Aldol product **11** containing bufogargarizin A skeleton was found to be less stable than **10** by ∼1.2 kcal mol^−1^, which corresponds to the equilibrium ratio of **10**:**11 **= 7:1. The C1‐epimer of **10**, compound **21**, was found to have similar to **11** energy, which corresponds to the observation that it was also observed during the equilibration of **10** (Scheme 2b). Diastereomeric to **10** compound **24**, was found to be ∼1.2 kcal mol^−1^ less stable than **10**, which explains why it was also observed during the experiments performed at higher temperatures and excess of DBU.

The successful access to both bufogargarizin B intermediate **10** and bufogargarizin A intermediate **11** enabled our subsequent studies focused on the introduction of the β17‐pyrone moiety, which represents a significant challenge.^[^ ^5^, ^15^, ^27^, ^45^, ^46^, ^47^ ^]^ Our prior studies focused on the synthesis of steroids of the bufadienolide family, suggesting that β17‐pyrone may undergo extensive degradation after its exposure to various hydroboration conditions. Similarly, selective attempts to hydrogenate the Δ^15,16^‐alkene resulted in competitive reduction of the α‐pyrone ring, and multiple hydrogenation and C–O hydrogenolysis products were isolated.

**Scheme 3:**
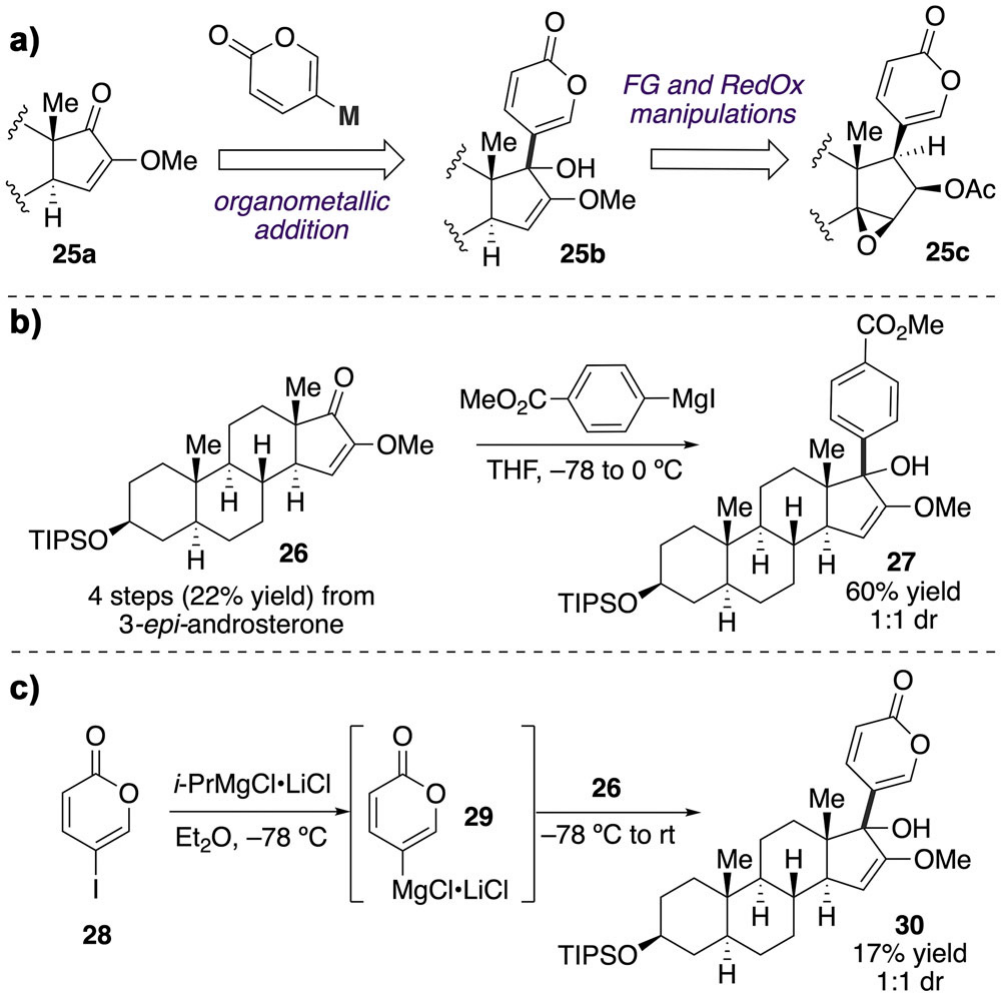
Initial studies on the installation of β17‐pyrone.

Based on these observations, a new strategy, summarized in Scheme 3a, was pursued. We envisioned introducing the α‐pyrone moiety through an organometallic addition of a metal‐functionalized pyrone^[^ ^48^, ^49^, ^50^ ^]^ to 2‐methoxy‐2‐cyclopentenone **25a**,^[^ ^51^ ^]^ which could be derived from **10** or **11** by oxidation of the D‐ring. It was anticipated that coupling product **25b** would be converted to a fully functionalized D‐ring present in **25c** through the subsequent redox manipulations.

To probe this strategy, the synthesis of model substrate **26** was accomplished in 4 steps (22% yield) from 3‐*epi*‐androsterone (Scheme 3b). The access to **26** allowed us to investigate the formation and reactions of organometallic reagents containing the C5‐functionalized α‐pyrone moiety (Table S5). Considering that the reaction of **34** with *p*‐carbomethoxyphenylmagnesium iodide proceeded to provide tertiary alcohol **27** in 60% yield (Scheme 3b),^[^ ^52^ ^]^ our next studies focused on generating the organolithium, organozinc, and organomagnesium species from various α‐pyrone derivatives (*cf*. Table S5 for additional details).

While most of these efforts were not successful, we discovered that subjecting iodide **28** to magnesium/halogen exchange with Turbo‐Grignard reagent^[^ ^53^ ^]^ at −78 °C resulted in an unstable organomagnesium reagent **29** along with oligomerized **28**. Attempts to trap this reagent with **26** resulted in **30** formed as a ∼1:1 mixture of diastereomers in 17% yield.

Since further attempts to improve the yield for the formation of **30** failed, a new strategy for the introduction of the β17‐pyrone moiety with the required oxidation at the C14, C15, and C16‐positions was pursued (*cf*. Figure 1e). This strategy is based on our recent synthesis of cinobufagin,^[^ ^27^ ^]^ a steroid isolated from the traditional Chinese medicine, *ChanSu*. Thus, we proposed that the installation of the α‐pyrone at the C17 position, resulting in compounds **12** and **15**, will be accomplished through a Stille or Suzuki cross‐coupling reaction with vinyl triflates derived from **10** and **11**. The cross‐coupling product **12** will be subjected to a [4 + 2] cycloaddition with singlet oxygen to provide the corresponding *endo*‐peroxides, which could be rearranged to the corresponding *bis*‐epoxides **13** and **16**.^[^ ^54^, ^55^, ^56^, ^57^, ^58^, ^59^ ^]^ Upon activation with a Lewis acid, **13** or **16** would rearrange to keto‐epoxides **14** or **17**, which could be elaborated to both bufogargarizins A and B via redox and protecting group manipulations.

**Scheme 4:**
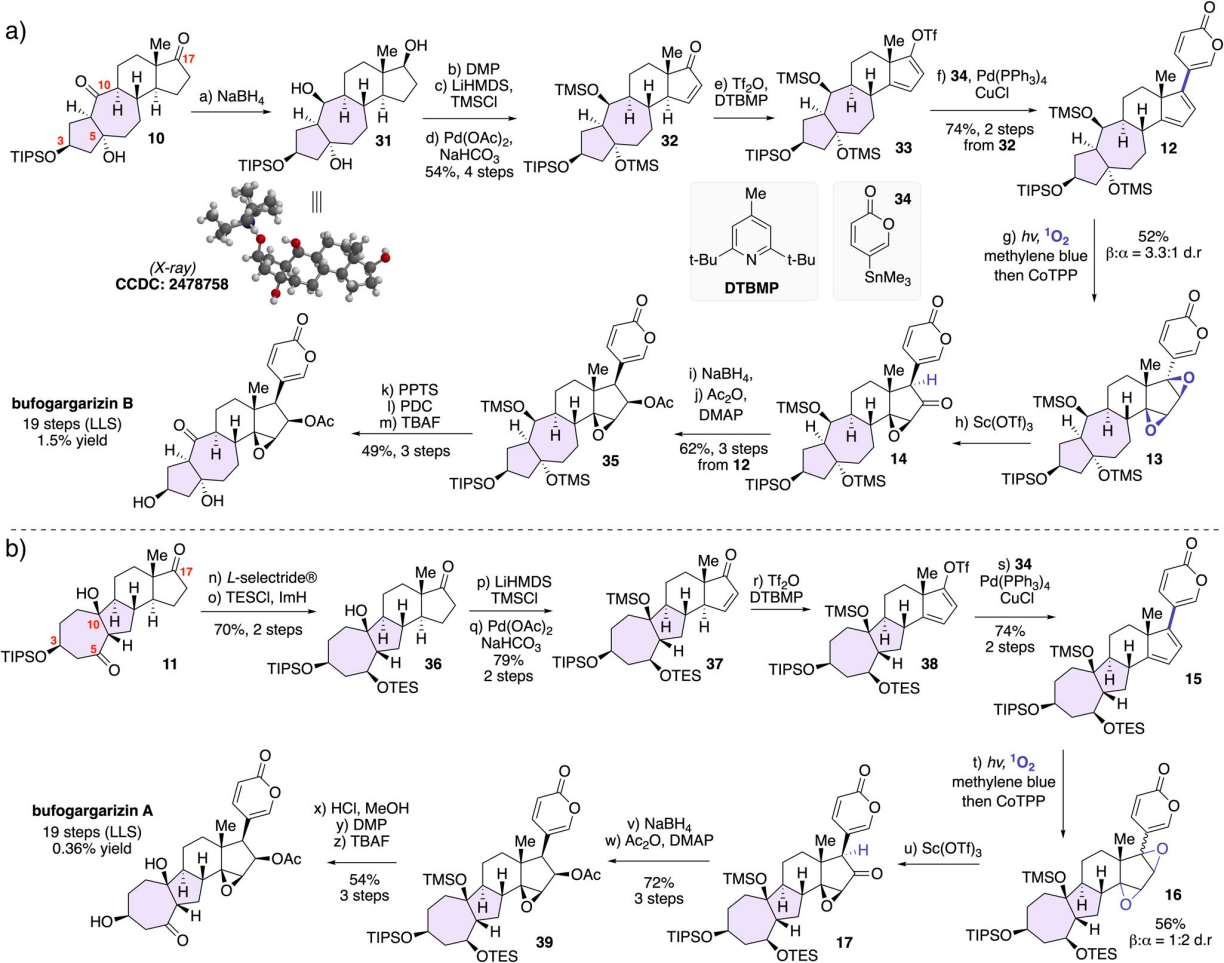
Completion of the syntheses of bufogargarizins A and B from aldol products **10** and **11**. Reagents and conditions: a) **Bufogargarizin B sequence**: (a) NaBH_4_ (20 equiv), MeOH, 1 h, r.t.; (b) Dess–Martin Periodinane (1.1 equiv), DCM, 3 h, r.t., 79% yield, 2 steps; (c) LiHMDS (10 equiv, TMSCl (10 equiv), THF, –78 °C, 3 h; (d) Pd(OAc)_2_ (1.2 equiv), NaHCO_3_ (2.4 equiv), 5:1 MeCN/DCM, r.t., 16 h, 68% yield; (e) Tf_2_O (2 equiv), DTBMP (10 equiv), DCM, 15 min, –78 °C to r.t.; (f) **34** (5 equiv), Pd(PPh_3_)_4_ (10 mol%), LiCl (10 equiv), CuCl (5 equiv), DMSO, 60 °C, 3 h, 74% yield, 2 steps; (g) O_2_ (1 atm), methylene blue (MB), DCM, –78 °C, *hv* (flood lamp), 45 min then CoTPP (0.1 equiv), –78 °C, 15 min, 40% yield of **13** and 12% yield of (α14,α15,α16,α17)‐diastereomer of **13**; (h) Sc(OTf)_3_ (1 mol%), DCM, r.t., 10 min; (i) NaBH_4_ (1.1 equiv), THF/MeOH (2:1),15 min, r.t.; (j) Ac_2_O (5 equiv), DMAP (0.3 equiv), Py, r.t., 62% yield, 3 steps; (k) PPTS (6 equiv), MeOH, r.t., 30 h, 63% yield (77% BRSM); (l) PDC (12 equiv), DCM, r.t., 21 h; (m) TBAF (2 equiv), THF, r.t., 22 h, 77% yield, 2 steps. b) **Bufogargarizin A sequence**: (n) *L*‐selectride (1.05 equiv), THF, –90 °C then NaOH, H_2_O_2_, MeOH, r.t., 77% yield; (o) TESCl (2.4 equiv), imidazole (3.6 equiv), DMAP (0.24 equiv), DMF, 0 °C to r.t., 91% yield; (p) LiHMDS (5 equiv), TMSCl (10 equiv), Et_3_N (10 equiv), THF, –78 °C, 1 h then r.t., 1 h; (q) Pd(OAc)_2_ (1.2 equiv), NaHCO_3_ (2.0 equiv), 5:1 MeCN, r.t., 12 h, 79% yield, 2 steps; (r) Tf_2_O (2 equiv), DTBMP (10 equiv), DCM, 15 min, –78 °C to r.t.; (s) **34** (5 equiv), Pd(PPh_3_)_4_ (10 mol%), LiCl (10 equiv), CuCl (5 equiv), DMSO, 60 °C, 74% yield, 2 steps; (t) O_2_ (1 atm), methylene blue (MB), NaHCO_3_ (2.0 equiv), DCM, –78 °C, *hv* (flood lamp), then CoTPP (0.1 equiv), –78 °C, 56% yield, 2:1 mixture of undesired (α14,α15,α16,α17)‐diastereomer of **16** and desired (β14, β15, β16, β17)‐diastereomer of **16**; (u) Sc(OTf)_3_ (1 mol%), DCM, r.t., 10 min; (v) NaBH_4_ (1.1 equiv), THF/MeOH (1:1),10 min, r.t.; (w) Ac_2_O (3.5 equiv), DMAP (0.2 equiv), Py, r.t., 72% yield, 3 steps; (x) HCl, MeOH, r.t.,15 min, 86% (90% BRSM); (y) Dess‐Martin Periodinane (1.3 equiv), NaHCO_3_ (3.0 equiv), DCM, r.t.; (z) TBAF (1.0 equiv), THF, r.t., 63% yield, 2 steps.

Our subsequent studies focused on advancing the [5.7.6.5] aldol product **10** to Δ^14,16^‐diene **12** and probing its singlet oxygen oxidation/epoxide rearrangement (*cf*. Scheme 4a). Considering that the C5 tertiary alcohol is prone to elimination to generate a Δ^5^‐alkene, our studies commenced with protection of the C10‐ketone by reduction with sodium borohydride to provide **31** as a single diastereomer, the structure of which was proved by X‐ray crystallographic analysis. This was followed by a selective oxidation of the C17‐alcohol using Dess–Martin Periodinane to form the corresponding C17‐ketone in 79% yield (2 steps). This product was converted into a silyl enol ether (LiHMDS, TMSCl), which was subjected to the Saegusa‐Ito oxidation to provide **32** in 68% yield over two steps. It was observed that the classical Saegusa‐Ito oxidation conditions lead to the partial deprotection of TMS protecting groups, and the inclusion of NaHCO_3_ was required to suppress this side‐reaction (*cf*. Table S4).

Finally, **32** was enolized with triflic anhydride in the presence of DTBMP^[^ ^54^ ^]^ to generate vinyl triflate intermediate **33** that was subjected to Stille cross‐coupling with a known stannane **34** to produce **12** in 74% yield over two steps. It was noted that diene **12** was unstable upon prolonged storage, and upon generation, it was immediately subjected to the next step. The subsequent [4 + 2] singlet oxygen cycloaddition using previously optimized conditions^[^ ^27^ ^]^ resulted in a 3.3:1 mixture of unstable diastereomeric endoperoxides that were prone to decomposition and rearrangement at the temperatures above –78 °C (see SI).^[^ ^60^, ^61^, ^62^, ^63^ ^]^ These intermediates were directly subjected to an in situ rearrangement with CoTPP at –78 °C,^[^ ^27^, ^63^, ^64^ ^]^ which produced the desired β‐addition product **13** in 40% yield, and its separable diastereomeric α‐*bis*‐epoxide resulting from an α‐addition in 12% yield. The access to the desired β‐*bis*‐epoxide, allowed us to explore the selective House–Meinwald rearrangement next.^[^ ^24^, ^45^, ^65^ ^]^ While the undesired (α14,α15,α16,α17)‐diastereomer of **13** underwent a facile and selective activation under a variety of acidic conditions, using these conditions for β‐*bis*‐epoxide resulted in an extensive decomposition and formation of various pyrone degradation side‐products (*cf*. Table S6). The evaluation of various Lewis acid promoters helped to identify scandium(III)‐trifluoromethanesulfonate as the exclusive catalyst to effectively promote the rearrangement to **14** in quantitative yield. To avoid epimerization, the obtained crude keto‐epoxide product **14** was immediately subjected to reduction with sodium borohydride, which led to the exclusive formation of the β16‐alcohol intermediate. This crude alcohol was subjected to acetylation with acetic anhydride and DMAP to provide the desired intermediate **35** in 62% yield over 3 steps. Intermediate **35** was extensively characterized using 2D NMR spectroscopy, and it was found to possess the desired fully functionalized D‐ring configuration present in natural bufogargarizins A and B.^[^ ^15^, ^16^, ^17^, ^18^ ^]^

It was anticipated that compound **35** would be readily converted to bufogargarizin B via a three‐step sequence that would involve 1) selective deprotection of the C10 secondary and tertiary C5 trimethylsilyl ethers; 2) oxidation of the C10 alcohol to the corresponding ketone; 3) deprotection of the C3‐triisopropylsilyl ether. Our model studies with compound **10** (*cf*. Table S9) suggested that acids and HF•Py may cause significant elimination of the C5 tertiary hydroxyl group under the deprotection conditions, and basic conditions relying on TBAF were found to be optimal. Unfortunately, subjecting **35** to the reaction with TBAF resulted in an unselective deprotection of the C3, C5, and C10 silyl ethers, producing a triol product. While subjecting this triol to double oxidation with PDC followed by 3β‐selective reduction with *L‐*selectride did produce bufogargarizin B, significant amounts of inseparable by HPLC impurities were also observed alongside (see Supporting Information).

In attempts to improve this route, a more vigorous optimization of the selective trimethylsilyl ether cleavage in the presence of the TIPS group was carried out (*cf*. Table S7). It was discovered that PPTS in methanol promoted a selective TMS‐group deprotection in **35** to provide the corresponding diol in 63% yield (77% BRSM). The resultant product was subjected to PDC oxidation that furnished TIPS‐protected bufogargarizin B, which was subjected to TBAF deprotection to produce bufogargarizin B in 77% yield over 2 steps. The spectroscopic characteristics of synthetic bufogargarizin B matched the data previously reported by the Ye and Li groups (*cf*. Tables S11 and S12).^[^ ^15^, ^16^, ^17^, ^18^ ^]^

This approach was subsequently utilized for converting aldol product **11** into bufogargarizin A (Scheme 4b). To avoid potential side reactions, the C5‐ketone moiety of **11** was reduced with *L*‐selectride and protected as a TES‐ether to provide **36** in 70% yield over 2 steps. Enolization followed by a silyl enol ether formation and subsequent Saegusa‐Ito oxidation of **36** using previously developed conditions led to enone **37** in 79% yield. This compound was converted to vinyl triflate **38**, which was subjected to previously developed Stille coupling to form **15** in 74% yield (2 steps). Subjecting the diene moiety of **15** to singlet oxygen oxidation/endoperoxide rearrangement led to a 2:1 mixture of diastereomeric products **16** in 56% yield. Disappointingly, the analysis of this mixture revealed that the desired β‐diastereomer of **16** was formed as the minor product.

**Figure 3:**
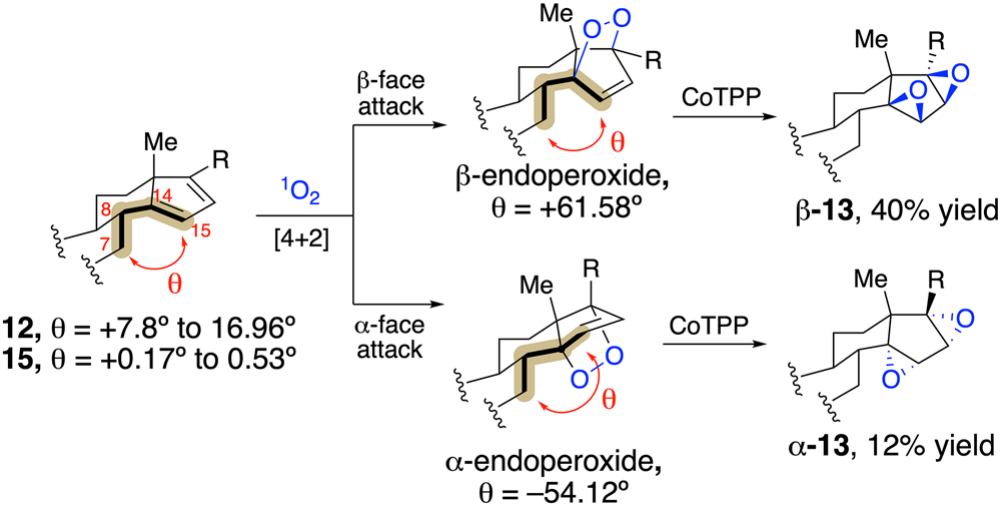
Computational studies of the selectivity for the singlet oxygen oxidation of **12** leading to **13** (DFT, B3LYP, 6–311 + G**).

The significant difference in the observed diastereoselectivity for the oxidation of **12** and **15** could arise from the differences in the tortional strain imposed by different AB‐ring systems, and such effects were previously observed by our group^[^ ^27^ ^]^ as well as others.^[^ ^58^, ^59^ ^]^ To probe the origins of these effects, the DFT‐based geometry optimization (B3LYP, 6–311 + G**, gas phase) of **12**, **15** and α‐ and β‐endoperoxide precursors to **13** and **16** was carried out (see Supporting Information). Consistent with the experimentally observed results, β‐endoperoxide precursor to bufogargarizin B (Figure 3) was found to be slightly more stable than the corresponding α‐endoperoxide by 0.27 kcal mol^−1^. At the same time, this trend was reversed for bufogargarizin A, and α‐endoperoxide precursor to **16** was calculated to be more stable by 0.81 kcal mol^−1^. While the observed α‐selectivity for the oxidation of bufogargarizin A intermediate **15** is in line with the prior literature precedents,^[^ ^56^, ^57^, ^58^, ^59^ ^]^ the preference for the β‐endoperoxide formed from bufogargarizin B skeleton is unexpected. The analysis of the low‐energy conformers of **12** and **15** revealed that the dihedral angle θ for the C7‐C8‐C14‐C15 portion is substantially larger for bufogargarizin B intermediate **12** than for **15** (+7.8° to + 16.96° for **12** versus +0.17° to + 0.53° for **15**). The formation of β‐endoperoxide requires structural reorganization to achieve θ = +61.58° while the formation of the α‐diastereomer would require a more significant reorganization to achieve θ = –54.12°. Considering that these torsional effects are most likely translated to the corresponding transition state energies leading to diastereomeric endoperoxides, we believe that the value of θ is an important parameter to consider when predicting the diastereoselectivity of this reaction.

To complete the bufogargarizin A synthesis, β‐*bis*‐epoxide **16** was subjected to Sc(OTf)_3_‐catalyzed House–Meinwald rearrangement/sodium borohydride reduction/acetylation sequence that led to **39** in 72% yield of 3 steps. The TMS‐ and TES silyl ether moieties of **39** were removed under acidic conditions (HCl in methanol), and the resultant diol was oxidized with Dess–Martin Periodinane to re‐install the C5‐ketone moiety. Finally, the removal of the TIPS‐ether with TBAF proceeded cleanly and provided bufogargarizin A in 54% yield from **39**. The spectroscopic properties of the synthetic bufogargarizin A were identical to the corresponding data published for the natural and synthetic samples (*cf*. Tables S13 and S14).

## Conclusion

In conclusion, we have developed a concise synthesis of the natural twin *abeo*‐steroids bufogargarizins A and B containing unusual [7.5.6.5] and [5.7.6.5] skeletons and a highly oxidized D‐ring with a challenging to install β17‐pyrone moiety. The described synthetic approach features an expedient synthesis of the key intermediate **10** and **11** from (+)‐methyl estrone via an ozonolytic cleavage of the Δ^5,10^‐alkene followed by a biomimetic regio‐ and stereoselective transannular aldol addition reaction. This transformation could be carried out either under kinetic or thermodynamic control to provide **10** and **11** as the major aldol products, along with various amounts of other diastereomers. The optimized one‐pot sequence ran under kinetic conditions consistently produced the desired bufogargarizins A and B precursors **10** and **11** in 50%–63% and 21%–27% yields, respectively. These studies provide direct evidence for the proposed biosynthetic hypothesis for the first time that both bufogargarizins A and B scaffolds can be synthesized via an intramolecular transannular aldol reaction. The subsequent elaboration of **10** and **11** to bufogargarizins A and B was accomplished in 13 steps. Both routes feature a singlet oxygen‐based oxidation method that results in a streamlined installation of the D‐ring oxidation and stereochemistry from a Δ^14,16^‐diene. Remarkably, the diastereoselectivity of the singlet oxygen oxidation step was found to be dependent on the AB‐ring configuration and proceeded with 3.3:1 d.r. favoring the desired β‐face selectivity for bufogargarizin B system, and with 1:2 d.r. for the bufogargarizin A system, favoring the undesired α‐diastereomer. This enabled the completion of bufogargarizins A and B syntheses in 19 steps (LLS), and 0.36% and 1.5% overall yield. We believe that the current strategy represents an improvement over the prior work in terms of the overall number of steps and efficiency of the synthesis of bufogargarizin B.^[^ ^66^ ^]^

## Supporting Information

The experimental procedures, characterization data, ^1^H and ^13^C NMR spectra of reaction products and intermediates are available free of charge.

## Conflict of Interests

The authors declare no conflict of interest.

## Supporting information



Supporting Information

Supporting Information

## Data Availability

The data that support the findings of this study are available in the Supporting Information of this article.
