# Supplementary material for: Bioinspired Synthesis of Twin abeo‐Steroids Bufogargarizins A and B via a Divergent Intramolecular Aldol Addition Reaction
Source: Angew Chem Int Ed Engl. 2025 Nov 19;65(2):e19121. doi: 10.1002/anie.202519121 (PMC12681978; doi:10.1002/anie.202519121)
Supplement: Supplementary file 1 — Supporting Information [file ANIE-65-e19121-s002.pdf]

Supporting Information

Bioinspired Synthesis of Twin *abeo*-Steroids Bufogargarizins A and B via a Divergent Intramolecular Aldol Addition Reaction

Zoey J. Surma,[a,#] Volodymyr Hiiuk,[a,#] Eugene Zviagin,[a,#] Yaxin Ouyang, Alex Chacko,[a] Fengrui Qu,[a] Paul M. Zimmerman,[a] and Pavel Nagorny[a,*]

[a]*Department of Chemistry, University of Michigan, 930 N. University Ave., Ann Arbor, MI 48109*

[#]*Equally contributing co-authors*
**Corresponding author. Email: nagorny@umich.edu*

Table of Contents

1. **Synthetic Route** **S3**
   - i. Steroid nomenclature used in the manuscript and SI S3
   - ii. Summary of the prior synthetic work S4
   - iii. Synthetic approaches to intermediates **8**, **10**, and **11** S7
   - iv. Summary of the synthesis of bufogargarizins A and B S8
2. **Optimization Studies** **S10**
   - i. Transannular ozonolysis/aldol cascade optimization S10
   - ii. Saegusa-Ito oxidation optimization S16
   - iii. Pyrone installation studies S17
   - iv. Singlet oxygen model system studies S18
   - v. House-Meinwald rearrangement optimization S19
   - vi. Late-stage sequence optimization S20
   - vii. Triethyl silyl protection optimization for bufogargarizin A synthesis S22
3. **Experimental Procedures** **S23**
   - i. General information S23
   - ii. Synthesis of aldol products **10** and **11** S24
   - iii. Alternative access to intermediate **8** S32
   - iv. Synthesis of bufogargarizin B S34
   - v. Synthesis of bufogargarizin A S49
4. **X-Ray Crystallography** **S61**
   - i. Synthesis of compounds **19**, **20** and **64** S61
   - ii. X-ray crystallographic data of **19** S64
   - iii. X-ray crystallographic data of **20** S71
   - iv. X-ray crystallographic data of **21** S78
   - v. X-ray crystallographic data of **22** S85
   - vi. X-ray crystallographic data of **31** S93
   - vii. X-ray crystallographic data of **64** S101
5. **Flood Lamp Emission Spectra** **S107**
6. **Computational Studies** **S108**
7. **References** **S144**
8. **^{1}H, ^{13}C, and 2D NMR Spectra of Synthesized Compounds** **S146**

1. Synthetic Route

i. Steroid nomenclature used in the manuscript and SI

Scheme S1. Tetracyclic ring system numbering and α/β designation.

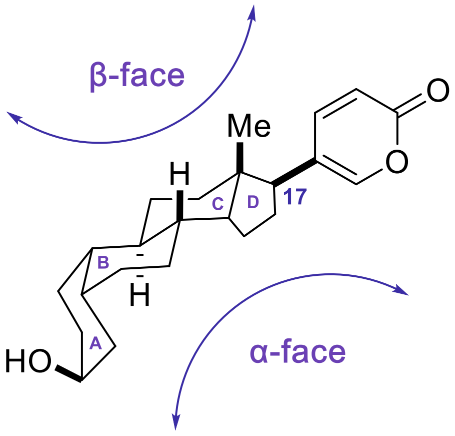

generic bufadienolide

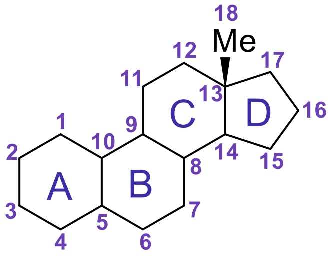

[6.6.6.5]

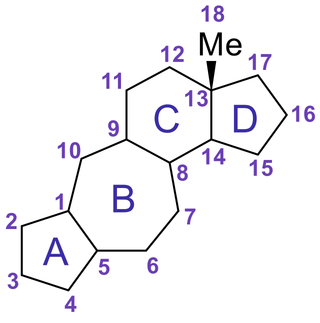

[5.7.6.5]

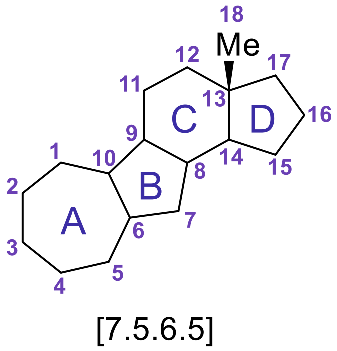

[7.5.6.5]

The top face of the steroid is referred as the β-face and the bottom face is referred as the α-face. Typical [6.6.6.5] steroid skeletons follow the conventional numbering system, whereas *abeo*-steroids with rearranged ring systems follow a different numbering scheme based on the IUPAC nomenclature shown above.

ii. Summary of the prior synthetic work

Scheme S2. First total synthesis of bufogargarizins A and B (Li et al., 2023).[1]

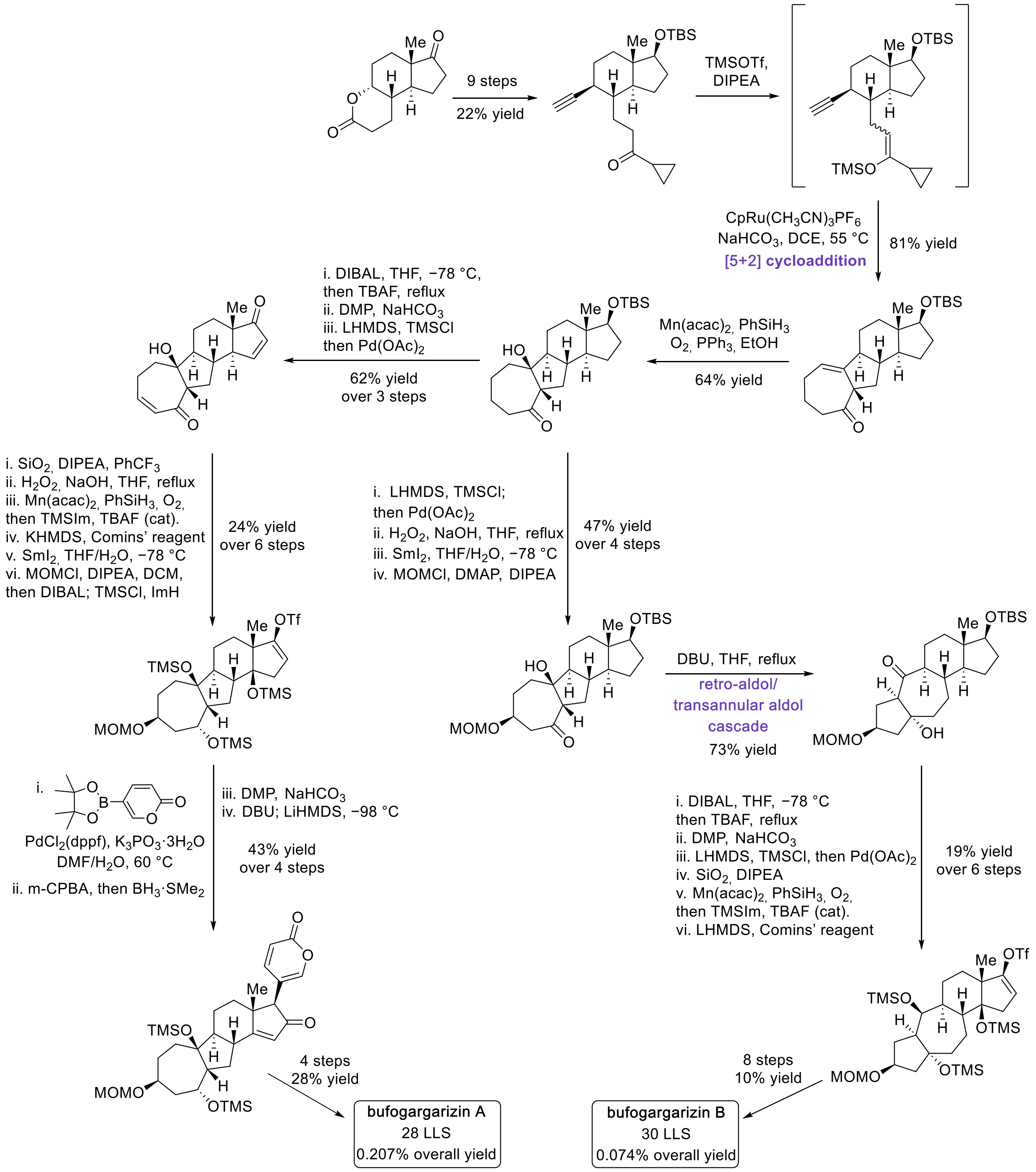

In 2023, the Li research group reported the first asymmetric total syntheses of bufogargarizins A and B in 28 and 30 linear steps, respectively.[1] A ruthenium-catalyzed [5+2] cycloaddition was employed to construct the [7.5.6.5] tetracyclic scaffold, which was elaborated into bufogargarizin A. Remarkably, this [7.5.6.5] framework could be transformed into the [5.7.6.5] system through a retro-aldol/aldol cascade, thereby enabling access to bufogargarizin B. These achievements represent the first syntheses of these structurally complex natural products, distinguished by their rearranged A/B ring systems, highly oxygenated D ring, α-pyrone motif sensitive to diverse conditions, and ten stereocenters.

Scheme S3. Formal synthesis of bufogargarizin B (Xu et al., 2025).[2]

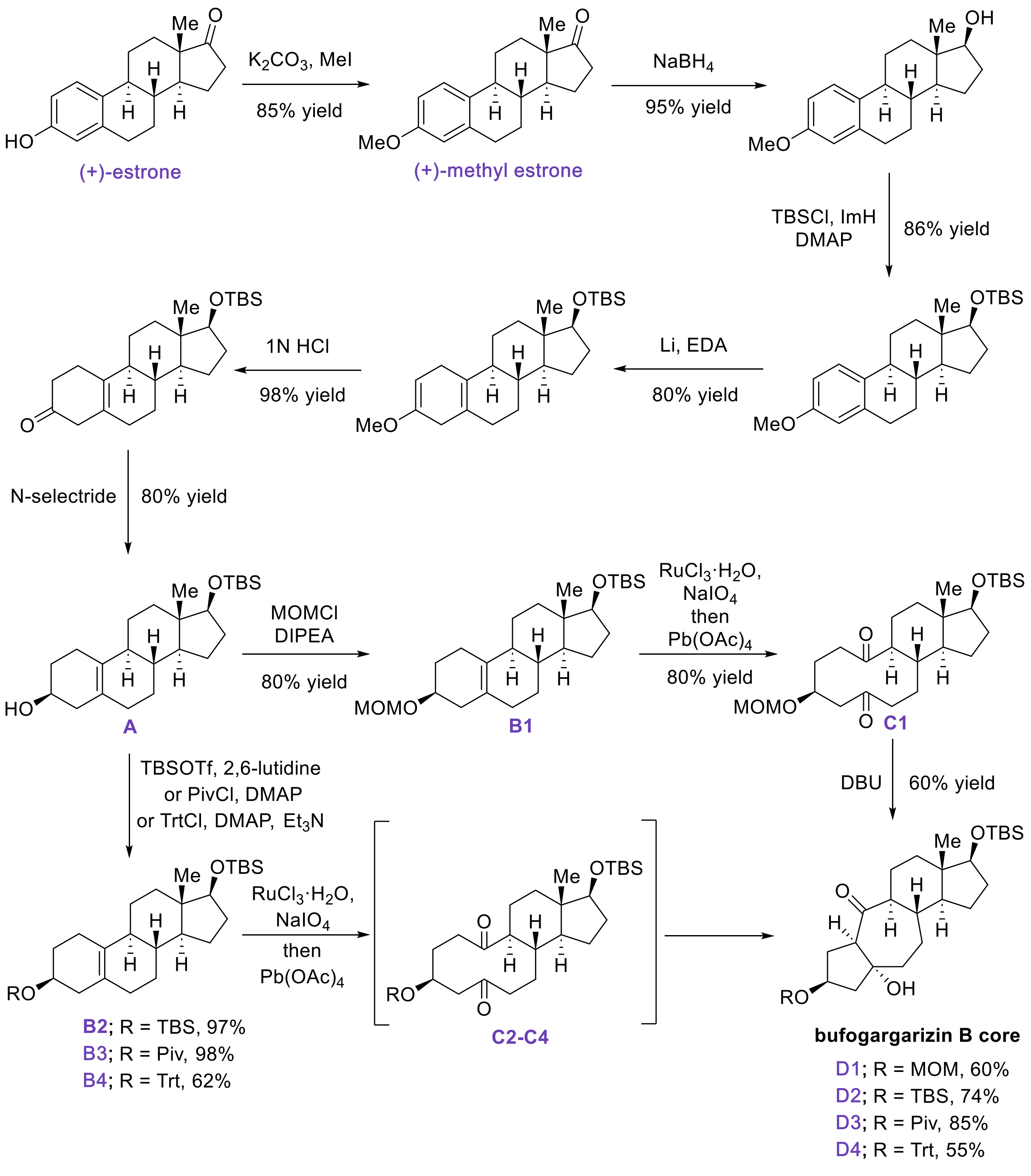

A contemporaneous report by the Xu group (published in June of 2025) employs a strategy similar to ours for the construction of the bufogargarizin B core.[2] Xu and coworkers synthesized compound **A** in six steps with an overall yield of 44% and used it to access steroids **B1-B4** in 80%, 97%, 98%, and 62% yields, respectively. These compounds were then treated sequentially with $RuCl_3·H_2O$ and $NaIO_4$, followed by $Pb(OAc)_4$ to afford the diketone intermediates **C1-C4**. Here, they demonstrated that the protecting group affects the yield of the transannular aldol reaction, with Piv being the most optimal C_3 hydroxyl protecting group. Subjecting **C1** to DBU in refluxing THF furnished the bufogargarizin B core **D1**, which was previously synthesized by the Li group (*cf.* Scheme S2). Alternatively, **B2-B4** were directly subjected to dihydroxylation/glycol oxidation with lead(IV) tetraacetate to produce **D2-D4**.

Scheme S4. Detailed retrosynthetic analysis highlighting the key steps.

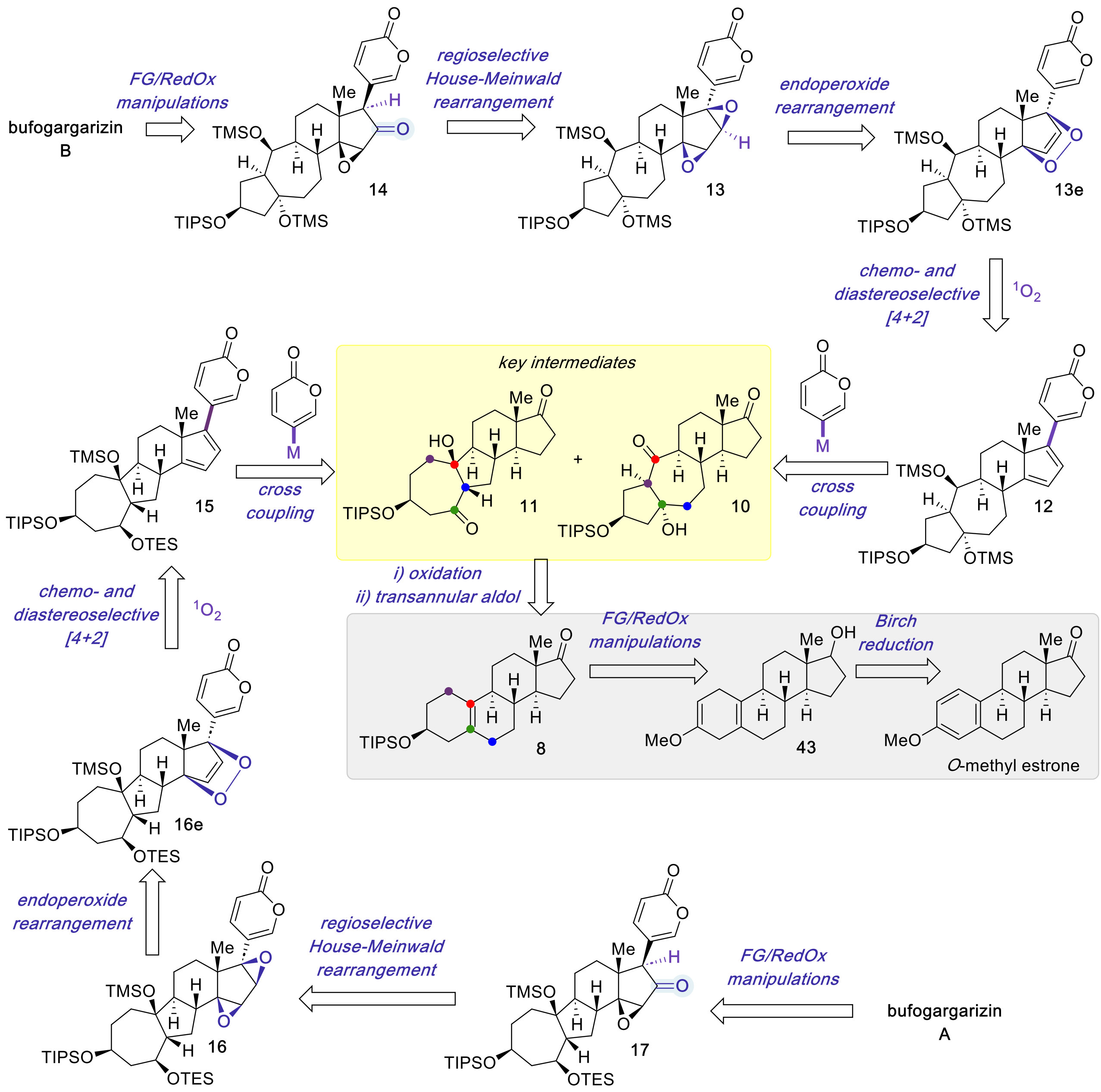

iii. Synthetic approaches to intermediates 8, 10, and 11

Scheme S5. Synthesis of **8**, **10**, and **11**.

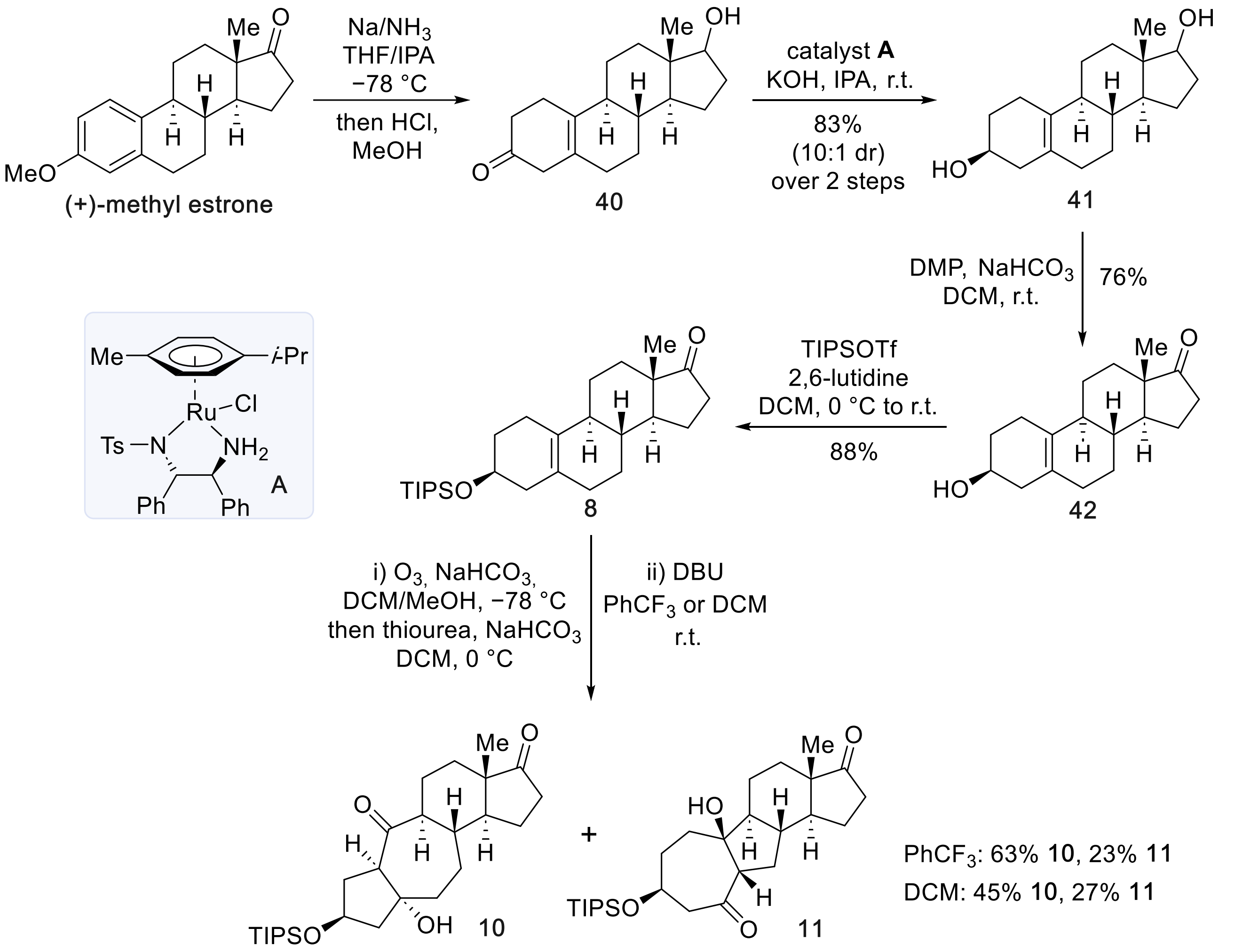

Scheme S6. Alternative synthesis of intermediate **8**.

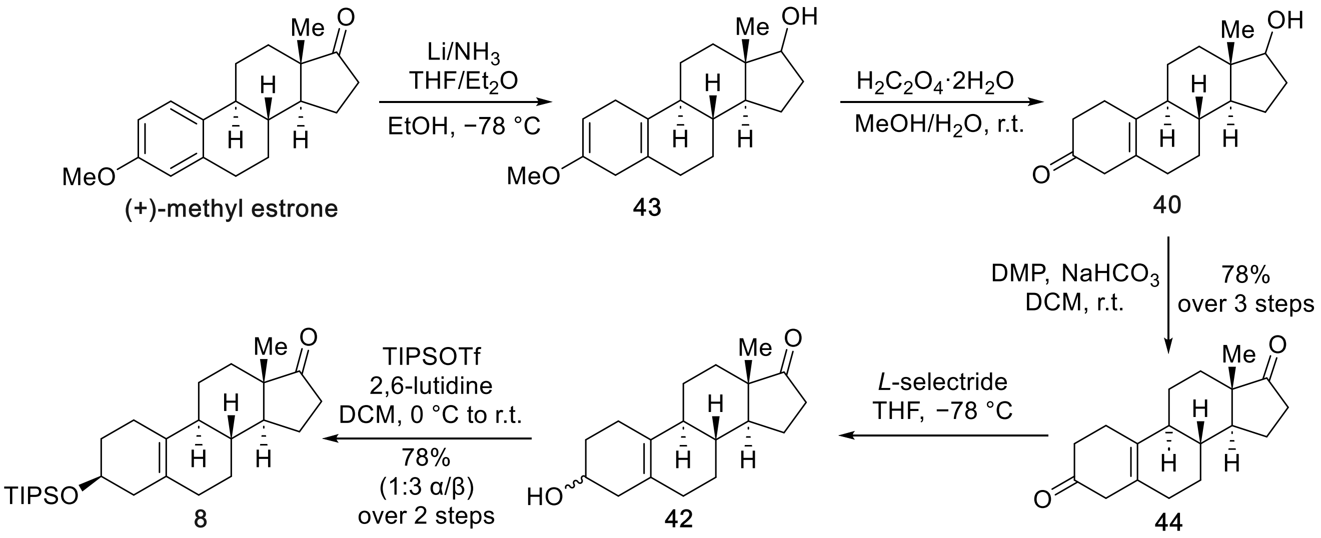

iv. Summary of the synthesis of bufogargarizins A and B

Scheme S7. Synthesis of bufogargarizin B from **10**.

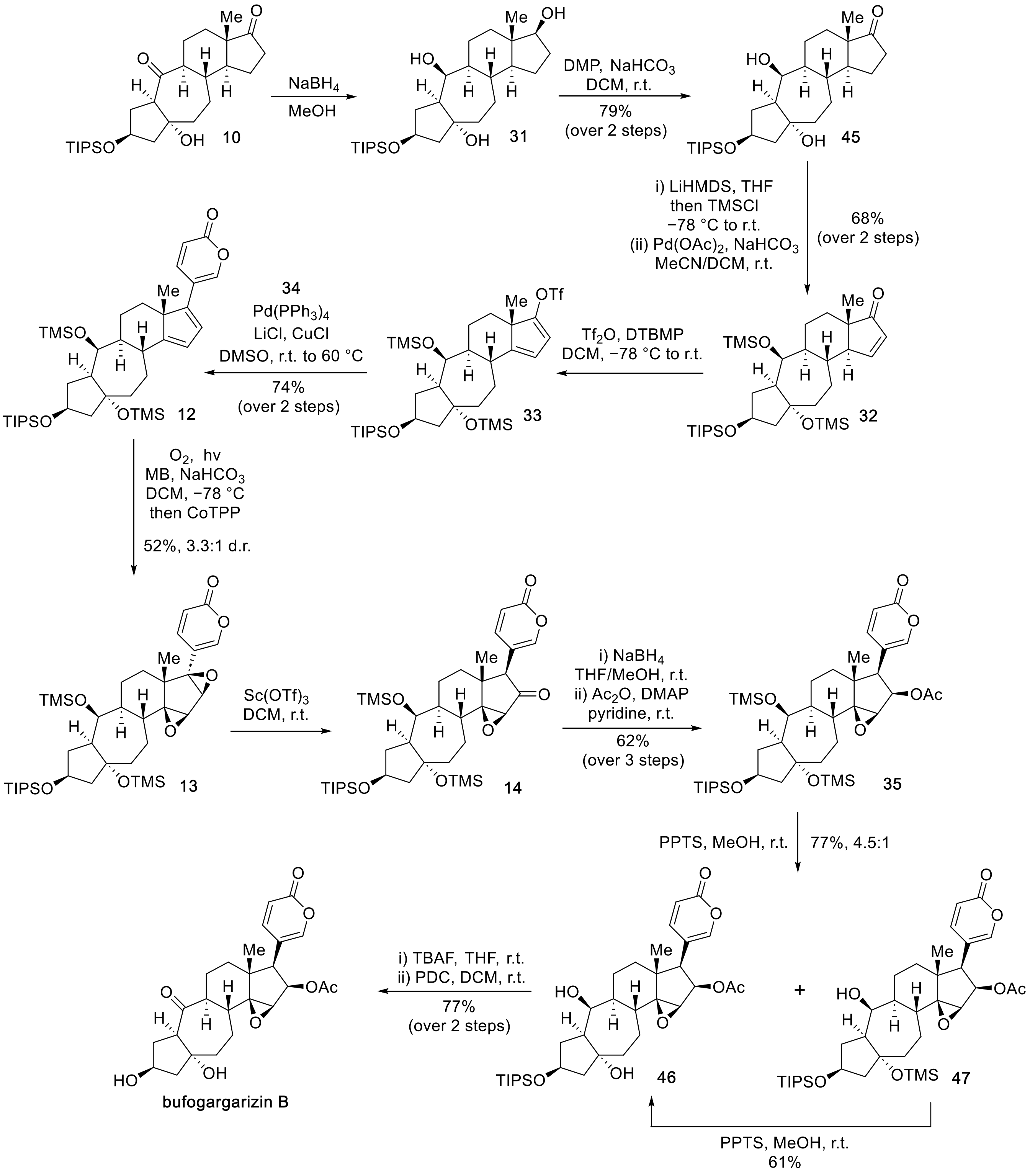

Scheme S8. Synthesis of bufogargarizin A from **11**.

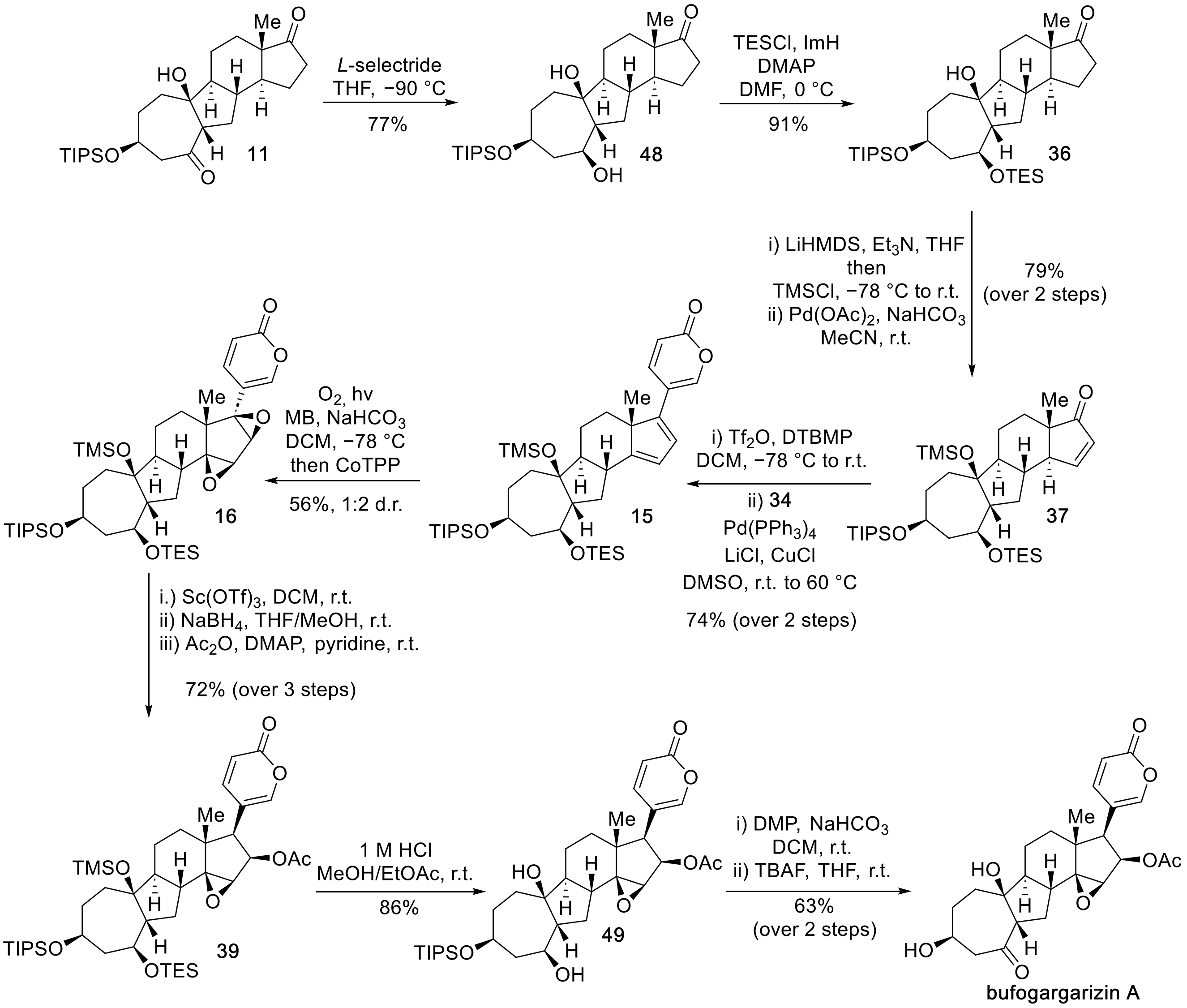

2. Optimization Studies

i. Transannular ozonolysis/aldol cascade optimization

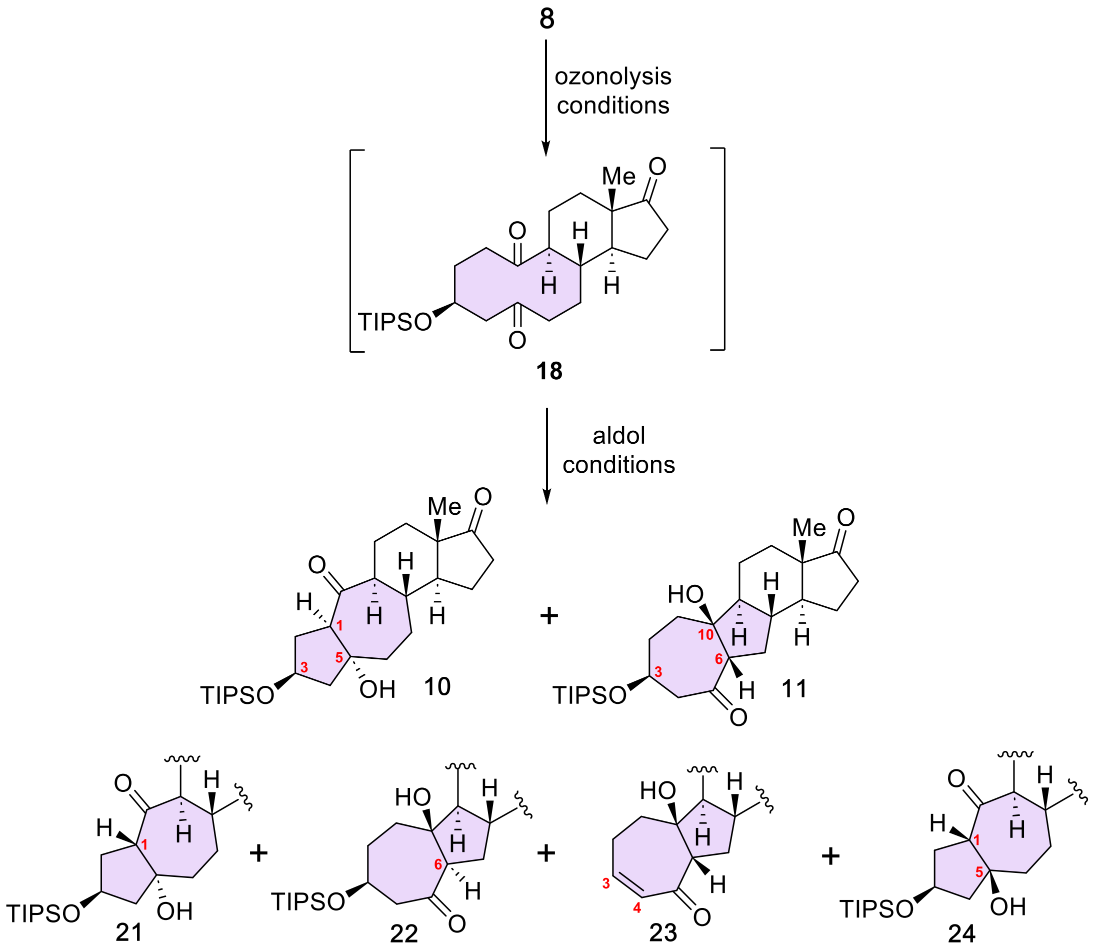

Note: The formation of compound **18** as an intermediate in the transannular ozonolysis/aldol cascade was confirmed by NMR and HRMS (see page S28).

Table S1. Transannular ozonolysis/aldol cascade: optimization studies.

Entry	Ozonolysis conditions		Aldol conditions		Observed products (% yield)
	DCM:MeOH (x:y)	thiourea (equiv)	Base/acid	Solvent t˚C, rxn time	
**1**	5:1 to 6:1	1.2	basic Al_2O_3 (54 equiv)	DCM (0.02 M), r.t., 26 h	no aldol reaction
**2**	5:1 to 6:1	1.2	SiO_2 (90 equiv)	DCM (0.02 M), r.t., 48 h	no aldol reaction
**3**	5:1 to 6:1	1.2	*p*-TsOH·H_2O (0.24 equiv)	DCM (0.02 M), 0 °C to r.t., 2 h	no aldol reaction
**4**	3:1 to 4:1	1.2	*trans*-4F-Pro (0.2 equiv)	DMSO (0.5 M), r.t., 17 h	no aldol reaction
**5**	3:1 to 4:1	2.0	KO*t*-Bu (1.0 equiv.)	THF (0.02 M), 0 °C, 2 h	-
**6**	3:1 to 4:1	2.0	KO*t*-Bu (2.0 equiv.)	THF(0.05 M), r.t., 4 h	-

Entry	Ozonolysis conditions		Aldol conditions		Observed products (% yield)
	DCM:MeOH (x:y)	thiourea (equiv)	Base/acid	Solvent t°C, rxn time	
**7**	3:1 to 4:1	2.0	DBU (15 equiv)	THF (0.02 M), reflux, 11 h	**10** (38%), **11** (7%) **21** (13%), **22** (3%), **23**, **24**
**8**	5:1 to 6:1	1.2	DBU (10 equiv)	THF (0.02 M), reflux, 1 h	**10** (31%), **11** (15%) **21** (18%), **22** (6%) **23**, **24**
**9**	5:1 to 6:1	1.2	DBU (10 equiv)	THF (0.02 M), r.t., 2 h	**10** (37%), **11** (17%) **21** (11%), **22** (3%)
**10**	3:1 to 4:1	1.2	DBU (1.0 equiv)	THF (0.02 M), r.t., 3 h	**10** (41%), **11** (20%) **21** (10%), **22** (3%)
**11**	3:1 to 4:1	1.2	DBU (2.0 equiv)	DCM (0.03 M), −30 °C to −5 °C, 3 h	**10** (41%), **11** (20%) **21** (2%), **22** (1%)
**12**	3:1 to 4:1	1.2	DBU (0.5 equiv)	DCM (0.03 M), 0 °C, 2 h	**10** (48%), **11** (26%) **21** (3%), **22** (1%)
**13**	2:1 to 3:1	1.2	DBU (0.5 equiv)	DCM (0.02 M), 0 °C to r.t., 2h	**10** (46%), **11** (24%) **21** (2%), **22** (1%)
**14**	2:1 to 3:1	1.2	DBN (0.5 equiv)	DCM (0.02 M), 0 °C to r.t., 5 h	**10** (44%), **11** (22%) **21** (6%), **22** (3%)
**15**	2:1 to 3:1	1.2	TBD (0.5 equiv)	DCM (0.02 M), 0 °C to r.t., 5 h	**10** (31%), **11** (8%) **21** (8%), **22** (5%)
**16**	2:1 to 3:1	1.2	TMG (7.0 equiv)	DCM (0.02 M), 0 °C to r.t., 20 h	**10** (40%), **11** (23%) **21** (6%), **22** (3%)
**17**	3:1 to 4:1	2.0	DBU (0.5 equiv)	DCM (0.02 M), r.t., 1.5 h	**10** (45%), **11** (27%) **21** (6%), **22** (1%)
**18**	3:1 to 4:1	2.0	DBU (0.5 equiv)	1.4-dioxane (0.02 M), r.t., 3.5 h	**10** (51%), **11** (16%) **21** (8%), **22** (2%)
**19**	3:1 to 4:1	2.0	DBU (0.5 equiv)	DMF (0.02 M), r.t., 1.5 h	**10** (38%), **11** (25%) **21** (7%), **22** (5%)
**20**	3:1 to 4:1	2.0	DBU (0.5 equiv)	$PhCF_3$ (0.02 M), r.t., 1.5 h	**10** (63%), **11** (23%) **21** (5%), **22** (1%)
**21**	3:1 to 4:1	2.0	DBU (0.5 equiv)	$PhCF_3$ (0.02 M), reflux, 1.5 h	**10** (35%), **11** (16%) **21** (13%), **22** (9%), **23**, **24**

Note: Compounds **23** and **24** were not isolated by column chromatography to determine their yields in the experiments described in entries 7, 8, and 21. However, characteristic peaks corresponding to these compounds were observed in the ^{1}H NMR spectra of the respective crude mixtures.

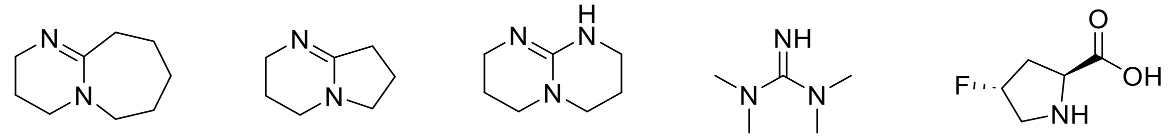

DBU (entries 7-13; 17-21)

DBN (entry 14)

TBD (entry 15)

TMG (entry 16)

***trans*-4F-Pro** *trans*-4-fluoro proline (entry 4)

Note: All experiments presented in Table S1 carried out on a scale of approximately 70–80 mg of compound **8** (0.16–0.18 mmol) as the starting material. Solvent, base/acid, concentration, reaction time, and temperature were varied as indicated in Table S1. The detailed procedure for the transannular ozonolysis/aldol cascade is described on page 27 of the SI.

To identify optimal conditions for the transannular ozonolysis/aldol cascade leading to the desired products **10** and **11**, we systematically investigated a range of reaction parameters, including the loading and chemical nature of the aldol promoters, solvent, and reaction temperature.

Initial attempts focused on post-ozonolysis treatment with weakly basic or acidic additives, including basic alumina (Al_2O_3), silica gel (SiO_2), *p*-toluenesulfonic acid monohydrate (*p*-TsOH·H_2O), and *trans*-4-fluoroproline (*trans*-4F-Pro) (entries 1–4). In all cases, no aldol products were observed, indicating that these additives were either insufficiently basic to promote enolate formation or incompatible with the reaction intermediates.

We next turned to stronger bases, beginning with KO*t*-Bu in THF (entries 5–6). These conditions also failed to provide aldol products, even at increased equivalents or prolonged reaction times, suggesting possible decomposition or base-induced side reactions incompatible with the ozonolysis-derived intermediates.

Encouraging results were obtained using DBU, a non-nucleophilic, sterically accessible amidine base (entries 7–13). Heating a THF solution of the ozonolysis product with DBU (15 equiv) at reflux (entry 7) led to the formation of desired product **10** in 40% yield, albeit with multiple byproducts including **21–24**. Lowering the DBU loading to 10 equiv (entries 8–9) and conducting the reaction at room temperature or under shorter reflux time improved the ratio of desired to side products but did not significantly enhance overall yield.

A notable improvement was observed upon reducing the DBU loading to 1.0 or 0.5 equiv (entries 10–13) and performing the aldol condensation at 0°C to room temperature in DCM. Under these conditions, product **10** was obtained in up to 48% yield, accompanied by **11** (26%), and minimal quantities of side products. These results underscore the importance of both base stoichiometry and reaction temperature in suppressing undesired pathways while favoring productive enolate formation and aldol addition.

To probe the influence of base structure, alternative amidine and guanidine bases were evaluated (entries 14–16). DBN (entry 14) offered comparable results to DBU, affording **10** and **11** in 44% and 22% yield, respectively. In contrast, TBD and TMG led to diminished selectivity and increased byproduct formation. These findings suggest that

while strong, non-nucleophilic bases are necessary, subtle differences in structure and basicity can significantly influence both conversion and chemoselectivity.

The solvent proved to be another key variable. When reactions were conducted with 0.5 equiv DBU in a range of solvents (entries 17–21), significant differences in product distribution emerged. Although DCM, 1,4-dioxane, and DMF supported moderate formation of **10** and **11**, the best performance was achieved in trifluorotoluene ($PhCF_3$) at room temperature (entry 20), providing **10** in 63% yield and **11** in 23% yield, with minimal side-product formation. Performing the reaction in $PhCF_3$ at reflux (entry 21) decreased selectivity, presumably due to thermal degradation or competitive side reactions. These results suggest that $PhCF_3$ offers an ideal balance of solvent polarity, stability, and inertness for promoting the aldol cascade.

a. Probing the reversibility of the transannular aldol addition reaction using 10 or 11

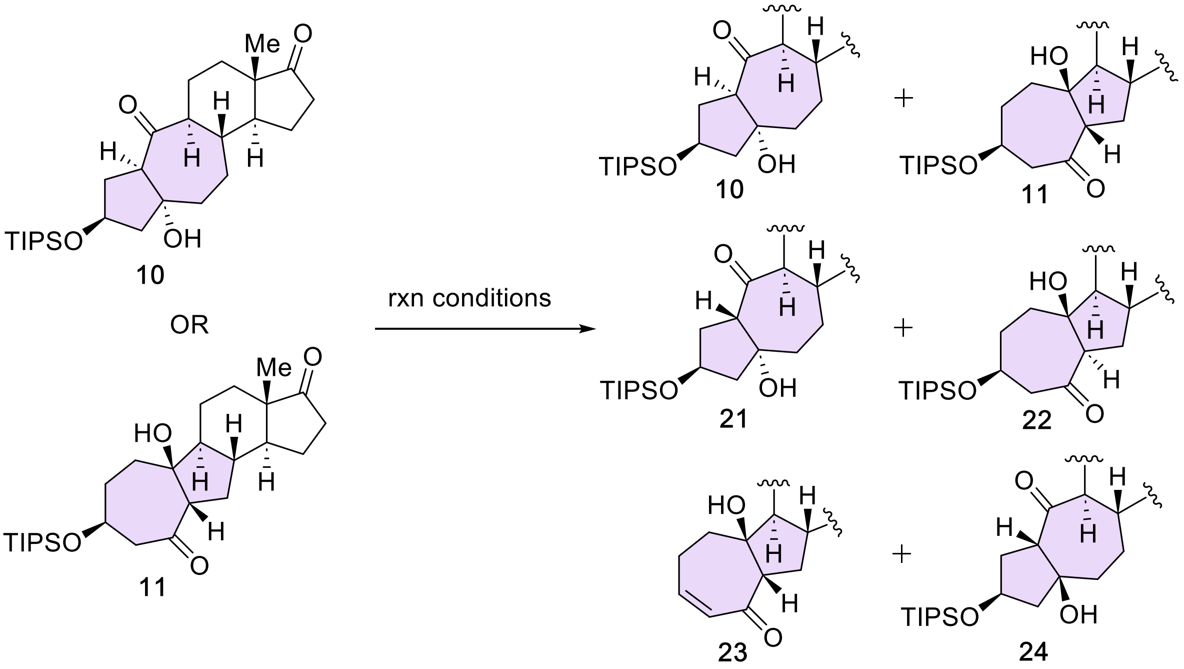

Table S2. Probing the reversibility of the transannular aldol addition reaction.

Entry	SM	Scale	rxn conditions	Observed products (% yield)
**1**	**10**	100.8 mg	DBU (15 equiv)<br>THF (0.10 M for SM)<br>reflux, 12 h	**10** (23%), **11** (3%)<br>**21** (9%), **22** (1%), **24** (6%)<br>**elimPdts**, including **23** + **decPdts**
**2**	**10**	29.8 mg	DBU (15 equiv)<br>THF (0.10 M for SM)<br>reflux, 12 h	**10** (29%), **11** (5%)<br>**21** (13%), **22** (2%), **24** (5%)<br>**elimPdts**, including **23** + **decPdts**
**3**	**10**	99.3 mg	DBU (15 equiv)<br>THF (0.02 M for SM)<br>reflux, 12 h	**10** (70%), **11** (4%)<br>**21** (16%), **22** (2%), **24** (trace)<br>**elimPdts** (trace), including **23**
**4**	**10**	29.6 mg	DBU (0.2 equiv)<br>THF (0.10 M for SM)<br>reflux, 12 h	**10** (23%), **11** (5%)<br>**21** (10%), **22** (3%), **24** (22%)<br>**elimPdts**, including **23** + **decPdts**
**5**	**11**	19.4 mg	DBU (15 equiv)<br>THF (0.10 M for SM)<br>reflux, 12 h	**10** (40%), **11** (5%)<br>**21** (11%), **22** (3%), **24** (trace)<br>**elimPdts**, including **23** + **decPdts**

SM = starting material**;** **elimPdts** = elimination products; **decPdts** = decomposition products

To evaluate the reversibility of the aldol reaction, isolated compounds **10** and **11** were subjected to DBU-mediated conditions in refluxing THF for 12 h. Under concentrated conditions (0.10 M for SM, 15 equiv DBU), both **10** (entries 1 and 2) and **11** (entry 4) were converted into a complex mixture of isomeric aldol products (**10**, **11**, **21**, **22**, and **24**), along with elimination and decomposition products. NMR analysis identified compound **23** as the major elimination product. Notably, compound **11**

underwent significant isomerization to **10** (40% recovery), supporting its kinetic origin. Increasing the reaction scale from 29.8 mg to 100.8 mg of **10** (entries 1 and 2) did not significantly affect the outcome, affording a similar aldol product distribution under the same conditions. Running the reaction of **10** under more dilute conditions (0.02 M) markedly suppressed decomposition, resulting in predominant recovery of the starting material (70%) and only trace amounts of elimination products, indicating that substrate concentration has a noticeable effect on the reaction outcome. When catalytic DBU (0.2 equiv) was employed at 0.10 M, a product distribution similar to that in entry 1 was observed, except for an increased formation of **24** (22%), suggesting that the amount of DBU has only a minor influence on the product distribution.

Overall, these results demonstrate that the aldol step is reversible and that the product distribution can be tuned between kinetic and thermodynamic regimes by varying the temperature and substrate concentration.

b. Transannular ozonolysis/aldol cascade: reproducibility and scalability

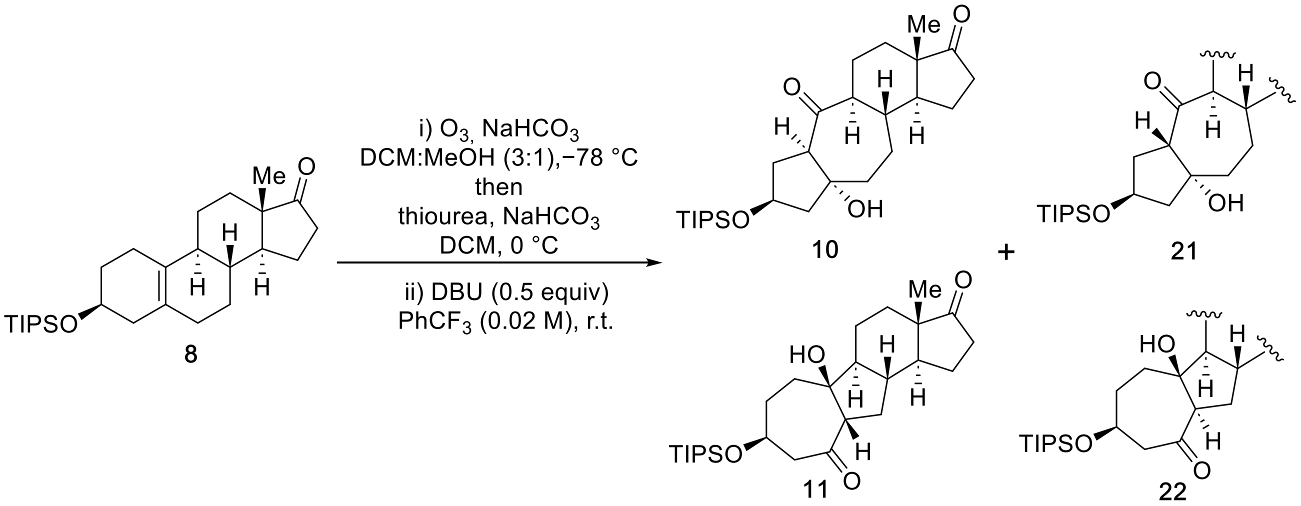

Table S3. Transannular ozonolysis/aldol cascade: reproducibility and scalability.

Entry	Scale	Observed products (% yield)
**1**	58.5 mg	**10** (54%), **11** (21%), **21** (4%), **22** (1%)
**2**	67.9 mg	**10** (63%), **11** (23%), **21** (5%), **22** (1%)
**3**	173.9 mg	**10** (52%), **11** (20%), **21** (2%), **22** (1%)
**4**	189.0 mg	**10** (50%), **11** (20%), **21** (6%), **22** (1%)
**5**	522.4 mg	**10** (52%), **11** (22%), **21** (5%), **22** (1%)

The transannular ozonolysis/aldol cascade was evaluated for reproducibility and scalability using starting material **8** across a range of reaction scales (58–522 mg). As shown in Table S3, consistent yields of the desired products **10** (50–63%) and **11** (20–23%) were obtained across all scales under the same conditions, with only minor formation of side products **21** and **22** (≤6% and ~1%, respectively). These results demonstrate the robustness and scalability of the optimized conditions without compromising product distribution or selectivity.

ii. Saegusa-Ito oxidation optimization

In the earlier stages of the sequence, we observed significant amounts of TMS-deprotection during the Saegusa-Ito oxidation. This was thought to be arising from the production of acetic acid from the precipitation of Pd^0 from $Pd(OAc)_2$. We began by screening conditions with various bases to mitigate this undesirable byproduct by using **(+)-estrone** as the model system.

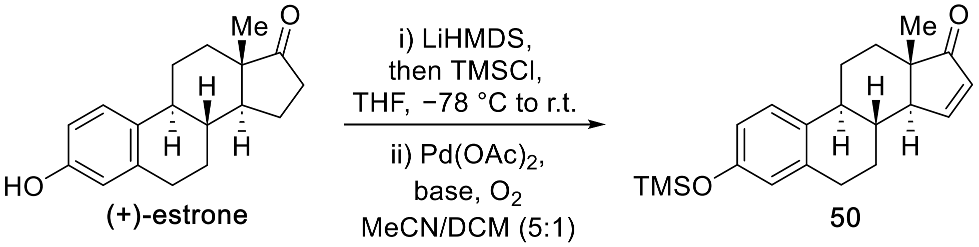

Table S4. Saegusa-Ito oxidation optimization.

Entry	Scale	Base	Equiv of $Pd(OAc)_2$	NMR conversion of 50
**1**	20 mg	Pyridine (1.2 equiv)	1.2	~20%
**2**	20 mg	Et_3N (1.2 equiv)	1.2	trace
**3**	20 mg	DIPA (1.2 equiv)	1.2	trace
**4**	20 mg	K_3PO_4 (1.2 equiv)	1.2	trace
**5**	20 mg	K_3PO_4 (2.4 equiv)	1.2	trace
**6**	20 mg	$NaHCO_3$ (1.2 equiv)	1.2	79%
**7**	20 mg	$NaHCO_3$ (1.2 equiv)	0.5	Incomplete
**8**	20 mg	$NaHCO_3$ (2.4 equiv)	1.2	91%
**9**	20 mg	$NaHCO_3$ (10 equiv)	1.2	32%

Each of these experiments were conducted in a flame dried 2-dram vial equipped with a stir bar and under an inert atmosphere of N_2 (g). Estrone was added to each of these vials and dissolved in THF (0.07 M) and cooled to -78°C, then LiHMDS (4 equiv, 0.295 mmol) was added as a solution in 1 mL of THF. The solution was stirred for 40 minutes at this temperature and then TMSCl (4 equiv, 0.295 mmol) was added. The reaction was stirred for 1 h and then warmed to r.t. and stirred for an additional hour. After this time, the reactions were quenched with 2 mL of sat. $NaHCO_3$ (aq) and extracted 3 × 5 mL of EtOAc, washed with 5 mL of brine, dried with Na_2SO_4, and concentrated. The products were then analyzed by ^1H NMR spectroscopy to confirm the silylation went to full conversion. The crude material was then dissolved in dry MeCN/DCM (5:1, 0.03 M) and placed under an atmosphere of O_2. Then, the base was added (see Table S4, entries 1-9) and $Pd(OAc)_2$ was added as a solid. The reactions were stirred overnight for 14 h, after this time the reactions were filtered through celite and washed with EtOAc, concentrated and analyzed via ^1H and ^{13}C NMR spectroscopy.

Since a silylated hydroxy group of a phenol is known to be incredibly unstable, we knew this was a great model system to investigate the effects of base and its ability to inhibit the undesired TMS deprotection. In entry 8, we found that 2.4 equiv of solid $NaHCO_3$ gave the highest conversion to our desired product, **50**. However, increasing the amount to 10 equiv (entry 9) led to only a 32% yield of the desired product, with the majority of the material remaining as the unoxidized silyl enol ether. This may be due to the fact that excess base scavenges the residual acid in the reaction mixture, thereby promoting desilyation through the formation of TMS-OAc adducts, or alternatively, excess base could deactivate $Pd(OAc)_2$.

iii. Pyrone installation studies

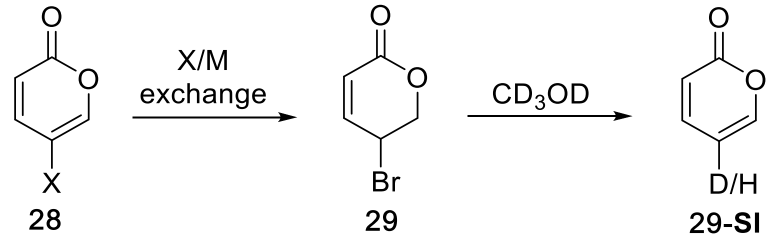

Table S5. Optimizing the formation of a 5-metallo pyrone.

Entry	X	Conditions	M	Result
**1**	Br	Mg, I_2, THF, −15 °C	MgBr	NR
**2**	Br	*n*-BuLi, $MgBr_2$, Et_2O, −95 °C	MgBr	Decomposition
**3**	Br	*i*-PrMgCl, LiCl, $ZnCl_2$, THF, 0 °C	MgCl·LiCl/ZnCl	Partial decomposition
**4**	Br	*i*-PrMgCl, LiCl, THF, −50 °C	MgCl·LiCl	Decomposition
**5**	Br	*i*-PrMgCl, LiCl, $ZnCl_2$, THF, −50 °C	MgCl·LiCl/ZnCl	Partial decomposition
**6**	Br	Activated Zn, THF, r.t.	ZnBr	NR
**7**	Br	Rieke Zn, THF, r.t.	ZnBr	NR
**8**	Br	Rieke Zn, THF, reflux	ZnBr	NR
**9**	Br	$ZnEt_2$, -30°C to r.t.	ZnEt	NR
**10**	Br	Nanosized Zn, LiCl, $MgBr_2$, THF or PhMe, 70 °C	ZnBr	**29-SI** (H only)
**11**	$SnBu_3$	*n*-BuLi, $MgBr_2$, Et_2O, −95 °C	MgBr	NR
**12**	I	Mg, I_2, THF	MgI	NR
**13**	I	*i*-PrMgCl, LiCl, THF, −50 °C	MgCl·LiCl	Decomposition
**14**	I	*i*-PrMgCl, LiCl, $ZnCl_2$, THF, −50 °C	MgCl·LiCl	Decomposition
**15**	I	Turbo-Grignard, THF, −50 °C	MgCl·LiCl	**29-SI** (H only) + **28** + decomp.
**16**	I	Turbo-Grignard, Et_2O, −50 °C	MgCl·LiCl	**29-SI** (D) + **28** + decomp.

Entry	X	Conditions	M	Result
**17**	I	Turbo-Grignard, DME, −50 °C	MgCl·LiCl	**29-SI** (D) + decomp**.**
**18**	I	Turbo-Grignard, DME, −50 °C	MgCl·LiCl	**29-SI** (D) + **28** + decomp.

Compound **28** was subjected to various conditions to generate **29**, and the reaction was quenched with CD_3OD, prompting the formation of deuterated pyrone **29-SI**. The efficiency of the halogen/metal exchange was accessed using ^{1}H NMR analysis of the crude mixture containing **29-SI**. The formation of protonated (rather than deuterated) **29-SI** is explained by the H-radical abstraction from the solvent by the pyrone-containing radical species. Justification of switching from THF to DME is explained by the fact that DME is a poorer H· donor, as explained in a study by Oshima and coworkers.[3]

iv. Singlet oxygen model system studies

Note: Endoperoxide decomposes at temperatures above −78 °C.

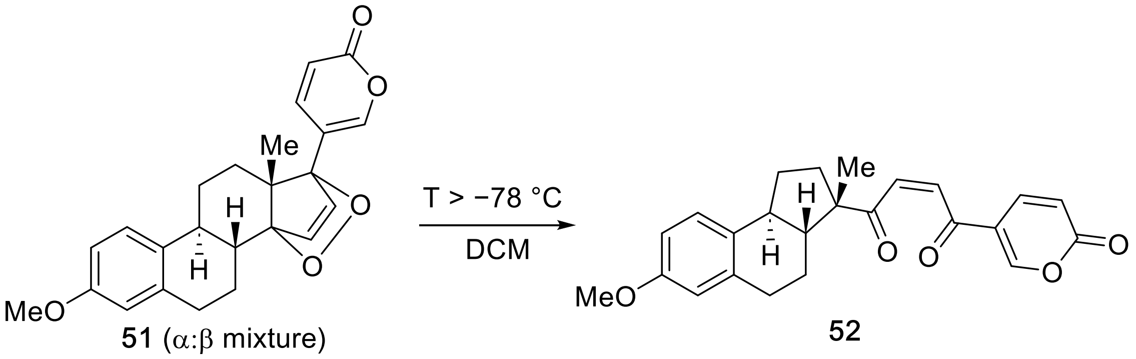

51 (α:β mixture) **52**

Endoperoxide **51** was synthesized according to our previously reported procedure[4] and warmed to room temperature. The reaction mixture was concentrated and purified by chromatography on Et_3N-treated silica (3:7, EtOAc:hexanes) to afford **52**, the major decomposition product, which was characterized as described below.

^{1}H NMR for 52 (500 MHz, C_6D_6) δ 7.26 (dd, *J* = 9.8, 2.7 Hz, 1H), 7.22 (dd, *J* = 2.7, 1.2 Hz, 1H), 6.94 (dd, *J* = 8.3, 1.1 Hz, 1H), 6.73 (dd, *J* = 8.4, 2.7 Hz, 1H), 6.69 (d, *J* = 2.7 Hz, 1H), 6.08 (d, *J* = 11.8 Hz, 1H), 5.69 – 5.57 (m, 2H), 3.41 (s, 3H), 2.75 (dt, *J* = 15.8, 8.0 Hz, 2H), 2.64 (td, *J* = 10.9, 5.6 Hz, 1H), 2.54 (ddd, *J* = 12.7, 4.6, 2.2 Hz, 1H), 2.23 – 2.15 (m, 1H), 1.72 (ddt, *J* = 11.4, 6.9, 2.1 Hz, 1H), 1.44 – 1.33 (m, 4H), 1.30 (d, *J* = 2.5 Hz, 1H), 1.12 (s, 3H).
^{13}C NMR for 52 (126 MHz, $CDCl_3$) δ 205.1, 187.7, 158.7, 158.6, 158.1, 139.5, 137.0, 136.6, 133.4, 133.3, 127.7, 118.6, 115.8, 114.0, 111.9, 55.7, 55.1, 54.8, 44.7, 36.1, 31.2, 24.5, 23.8, 20.6.
HRMS (ESI): calculated for $C_{24}H_{25}O_5Na^+$ $[M+Na]^+$ 415.1516, found 415.1520.

v. House-Meinwald rearrangement optimization

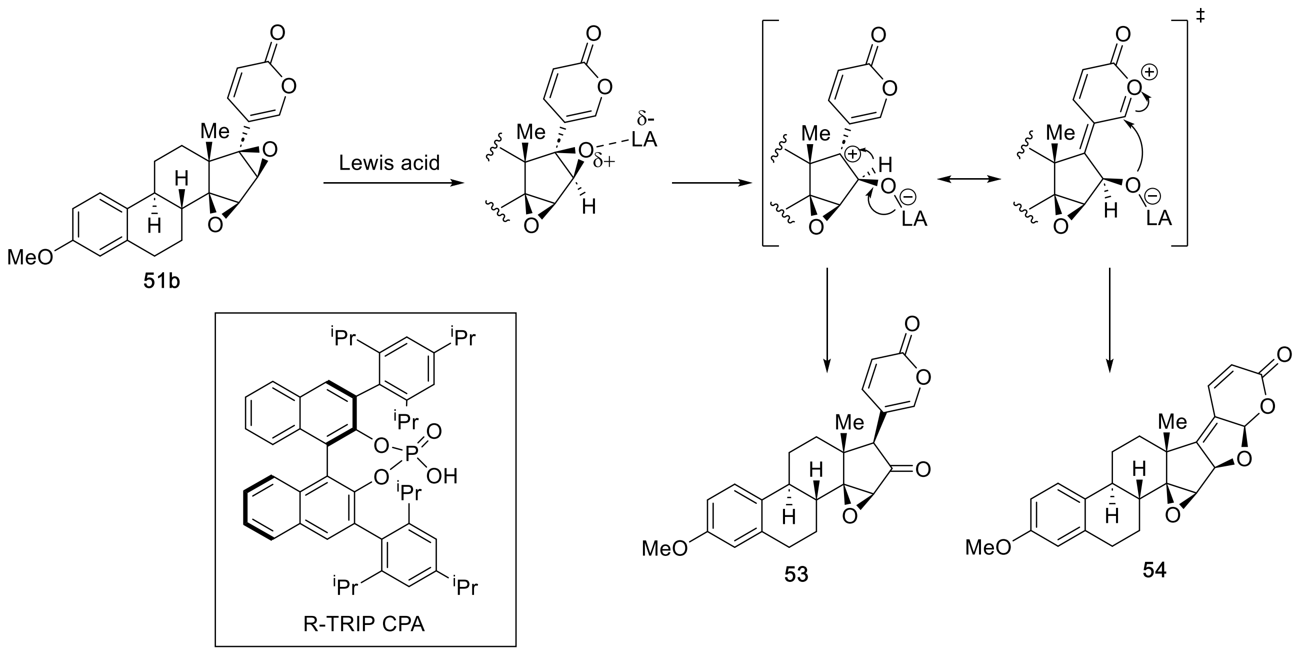

Table S6. Model studies for the House-Meinwald rearrangement optimization.

Entry	Scale	Conditions	Result
**1**	8.2 mg	TMSOTf (2.4 equiv), 2,6-lutidine (5 equiv), DCM, −78 °C to r.t.	NR
**2**	14 mg	SiO_2, DCM, r.t.	Trace **53**, decomposition
**3**	15 mg	$BF_3{\cdot}Et_2O$ (0.1 equiv), DCM, −40 °C	**54**, trace **53**
**4**	9.4 mg	TMSOTf (0.1 equiv), DCM, −78 °C	32% yield **53**
**5**	5.1 mg	TMSOTf (1 equiv), DCM, −78 °C	Decomposition
**6**	1.2 mg	$B(C_6F_5)_3$ (0.1 equiv), DCM, −78 °C	**54**, decomposition
**7**	3.5 mg	R-TRIP CPA (0.2 equiv), DCM, −78 °C	Some **54**, mostly unreacted
**8**	17 mg	$Sc(OTf)_3$ (0.1 equiv), DCM, r.t.	**53** quantitative yield

vi. Late-stage sequence optimization

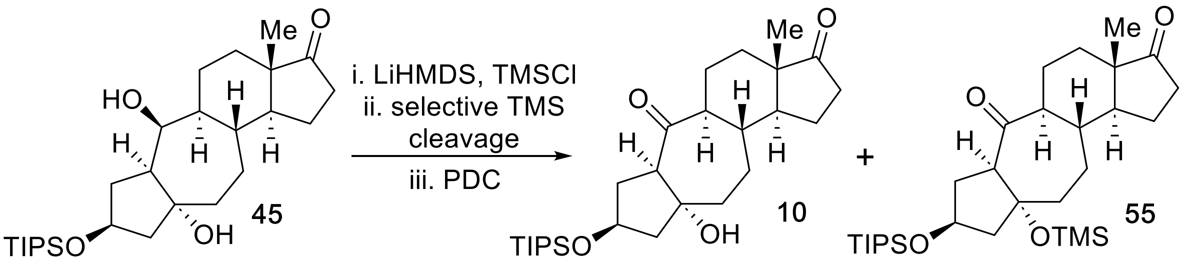

Table S7. Selective TMS removal & oxidation.

Entry	Scale	Conditions (ii)	Result
**1**	7.7 mg	K_2CO_3, MeOH	38% yield of **10** (88% brsm)
**2**	20 mg	$NaHCO_3$, MeOH	Complex mixture
**3**	20 mg	PPTS, MeOH	79% yield (4:1, **10**, **55**)

These studies first commenced with screening conditions for the selective trimethylsilyl cleavage in the presence of triisopropylsilyl protecting group at the C3 position. **45** was first silylated under the standard conditions using LiHMDS (10 equiv) followed by treatment with TMSCl (10 equiv) in THF (0.03 M). The silylated product was sufficiently pure and fully converted by ^1H and ^{13}C NMR, and it was advanced into the next step without purification. The crude material was then subjected to various conditions to selectively cleave TMS groups at the C10 and C5 positions.

Each of the selective C10 and C5 deprotection reactions (Table S7, entries 1-3) were run in a flame dried 2-dram vial equipped with a stir bar and under an inert atmosphere of N_2 (g). The silylated crude material was added and subsequently dissolved in methanol (0.02 M), then 4 equiv of either K_2CO_3 (entry 1), $NaHCO_3$ (entry 2), and PPTS (entry 3) were added to the reaction which was stirred at r.t. until completion on TLC. The reaction was concentrated, quenched with 2 mL of sat. $NaHCO_3$ (aq), extracted 3 × 5 mL of EtOAc, washed with 5 mL of sat. NaCl (aq) and dried with Na_2SO_4 and concentrated. The crude products were then dissolved in DCM (0.01 M) and 4 equiv of PDC was added as a solid. The reaction was stirred at room temperature until completion by TLC and then filtered through celite and concentrated. The following material was subjected to column chromatography (30% EtOAc in Hex) and analyzed by ^1H and ^{13}C NMR spectroscopy.

To our delight, K_2CO_3 in methanol led to 38% yield of **10**; however, with 50% recovery of starting material (Table S7). Unfortunately, when we subjected pyrone-containing compound **56** (*cf.* Table S8 below), to these conditions we found that decomposition was rapid. Treating our model system with $NaHCO_3$ in methanol led to a complex mixture of products. With these results in hand, we switched to slightly more acidic conditions to perform the selective trimethylsilyl group deprotection (Table S7, entry 3). Treatment with PPTS led to full consumption of the starting material in 3 h. The crude mixture was then subjected to PDC oxidation and gave the desired final product in 79% (4:1 ratio **10** and **55**) yield over 2 steps. Additionally, 10 mg of **56** was subjected

to PPTS in methanol (Table S8, entry 2) and to our delight we collected 10 mg of this starting material back, which indicates these conditions can tolerate the unstable α-pyrone ring. With these successful results, we next turned to optimization of the C3 deprotection.

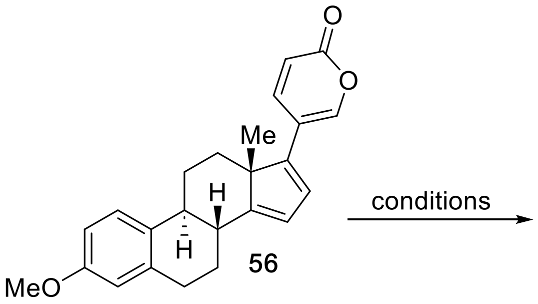

Table S8. α-pyrone stability to selective TMS deprotection.

Entry	Scale	Conditions	Result
**1**	10 mg	K_2CO_3 (4 equiv, 0.111 mmol), MeOH (0.02 M, 1.4 mL), r.t.	Full decomposition after 2 h
**2**	10 mg	PPTS (4 equiv, 0.111 mmol), MeOH (0.02 M, 1.4 mL), 4 h at r.t.	10 mg of starting material collected back

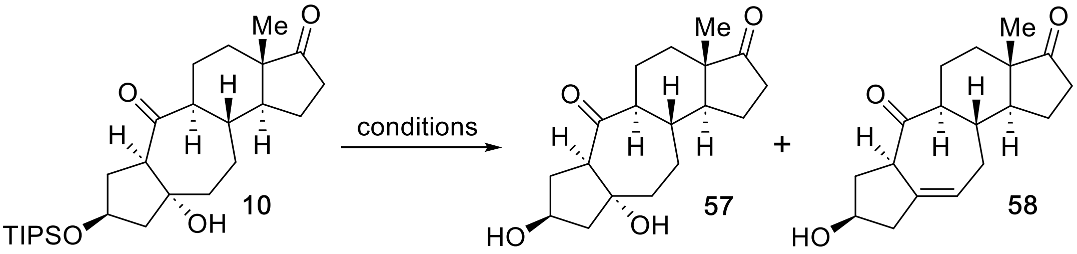

Table S9. TIPS deprotection optimization.

Entry	Scale	Conditions	Result
**1**	10 mg	TBAF (4 equiv), AcOH (4 equiv), THF (0.03 M), r.t., 22 h	**58**
**2**	10 mg	TASF (2 equiv), MeCN (0.04 M), r.t., 22 h	NR
**3**	10 mg	0.01 M HCl (2 equiv) in ethanol (0.03 M), r.t., 24 h	NR, some decomposition.
**4**	10 mg	PTSA (4 equiv), MeOH (0.03 M), r.t., 20 h	NR, some decomposition.
**5**	10 mg	CsF (5 equiv), ethanol (0.03 M), 5 days, r.t.	NR
**6**	10 mg	$NaClO_4$ (6 equiv), THF/H_2O (3:1, 0.006 M), THF, r.t., 20 h	NR
**7**	10 mg	$Et_3N{\cdot}3HF$ (3 equiv), THF (0.03 M), r.t., 18 h (sealed plastic tube)	NR
**8**	20 mg	TBAF (6 equiv), THF (0.03 M), r.t., 22 h	Full conversion. 3:1 **57:58.**

Various conditions for the triisopropylsilyl ether cleavage were investigated, as summarized in Table S9. Each of these reactions were run in a flame dried 2-dram vial equipped with a stir bar and under an inert atmosphere of N_2 (g). **10** was added and dissolved in the corresponding solvent and the de-silyating agent was added to the mixture at room temperature and was monitored by TLC. After this time, the reactions were quenched with 2 mL of $NaHCO_3$ (sat), extracted 3 × 5 mL of EtOAc, washed with 5 mL of sat. NaCl (aq) and dried with Na_2SO_4 and concentrated. The crude material was analyzed by ^{1}H and ^{13}C NMR spectroscopy.

These studies helped to identify TBAF (6 equiv) in THF (entry 8, Table S9) as the optimal conditions that result in minimal elimination byproduct. Although some elimination was present, decreasing the amount of TBAF to 1.5 equiv and running the reaction longer would prove to be the optimal strategy, as excess TBAF did not lead to full elimination in this experiment.

vii. Triethyl silyl protection optimization for bufogargarizin A synthesis

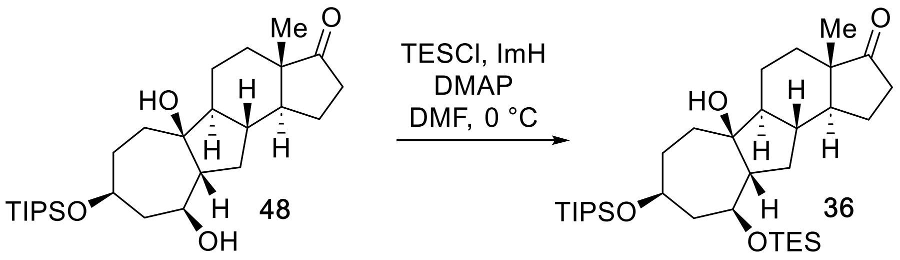

Table S10. Triethyl silyl protection optimization.

Entry	SM (equiv)	TESCl (equiv)	ImH (equiv)	DMAP (equiv)	rxn time	Yield of 36
**1**	1.0	1.1	1.65	-	50 h	5% (34% brsm)
**2**	1.0	2.0	3.00	0.20	3 h	79% (88% brsm)
**3**	1.0	2.4	3.60	0.24	1 h	91%

3. Experimental Procedures

i. General information

All reagents and solvents were purchased from commercial sources and were used as received without further purification unless otherwise specified. Anhydrous THF, DMF, and DCM were purified by Innovative Technology's Pure-Solve System using basic alumina. All reactions were carried out under a positive pressure of nitrogen in flame- or oven-dried glassware with magnetic stirring unless otherwise noted. Reactions were cooled using external cooling baths (dry ice/acetone, −78 °C) and heating was achieved using a silicone oil bath controlled by an electronic contact thermometer. Deionized water was used in the preparation of all aqueous solutions and for all aqueous extractions. Solvents used for extraction and chromatography were ACS or HPLC grade. Photochemical reactions were irradiated with either a Sealy Power Products MD520C.v2 400 W floodlamp with a maximum emission wavelength of 759 nm or a H160 Tuna Flora Kessil lamp on the red setting with a maximum emission wavelength of 655 nm. Full emission spectra for both light sources are included in a later section. Purification of reaction mixtures was performed on SiliCycle SiliaFlash F60 (230-400 mesh) or by using a CombiFlash Rf+ Lumen Automated Flash Chromatography System with UV detector using either SiliCycle SiLala's F60 (230-400 mesh) or Teledyne RediSep Rf Gold Silica (20-40 µm spherical). Yields indicate the isolated yield of the title compound with ≥95% purity as determined by ^{1}H NMR analysis from a single experiment. Diastereomeric ratios were determined by ^{1}H NMR analysis. ^{1}H NMR spectra were recorded on a Bruker Avance Neo 500 (500 MHz), Varian vnmrs 700 (700 MHz), Varian vnmrs 600 (600 MHz), Varian vnmrs 500 (500 MHZ), Varian MR400 (400 MHz) or a Varian Inova 400 (500 MHz) spectrometer and chemical shifts (δ) are reported in parts per million (ppm) with solvent resonance as the internal standard ($CDCl_3$ at δ 7.26, C_6D_6 at δ 7.16, $(CD_3)_2CO$ at δ 2.05, and CD_3OD at δ 3.30). Tabulated ^{1}H NMR data are reported as s = singlet, d = doublet, t = triplet, q = quartet, p = pentet, m = multiplet, br = broad, and coupling constants are reported in Hertz. Proton-decoupled ^{13}C NMR spectra were recorded on the instruments listed previously and chemical shifts (δ) are reported in parts per million (ppm) with solvent resonance used as the internal standard ($CDCl_3$ at δ 77.16 ppm, $(CD_3)_2CO$ at δ 206.26 and 29.84, CD_3OD at δ 49.00, and C_6D_6 at δ 128.06 ppm). All two-dimensional NMR spectra were recorded on a Bruker Avance Neo 500 (500 MHz) or Varian vnmrs 700 (700 MHz) spectrometers. High resolution mass spectra (HRMS) were performed and recorded on an Agilent 6230 TOF mass spectrometer in the University of Michigan mass spectrometry laboratory. Infrared (IR) spectra were recorded as liquids on a Thermo-Nicolet IS-50 FTIR spectrometer and absorbance peaks are reported in wavenumbers (cm^{-1}). Optical rotations were measured at room temperature in DCM or MeOH on a Jasco P-2000 polarimeter.

ii. Synthesis of aldol products 10 and 11

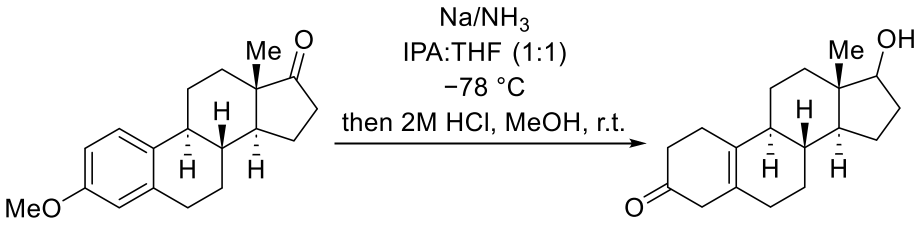

(+)-methyl estrone **40**

A 500-mL three-neck round-bottom flask equipped with a magnetic stir bar, a dry ice/acetone-cooled condenser, and a nitrogen inlet was charged with anhydrous ammonia (~200 mL), which was condensed into the flask at −78 °C under a stream of nitrogen. The condenser was continuously replenished with dry ice to maintain temperature throughout the ammonia collection.

To the cold solution of liquid ammonia, a precooled solution of (+)-methyl estrone (4.00 g, 14.1 mmol, 1.0 equiv) in a 1:1 mixture of isopropanol/tetrahydrofuran (120 mL) was added. The resulting mixture was stirred at −78 °C for 15 min, after which sodium metal (3.23 g, 140.7 mmol, 10.0 equiv) was added in small portions over 15 min. The reaction mixture was stirred at −78 °C for an additional 2.5–3 h. Solid ammonium chloride (10.0 g) was then added carefully to quench the reaction. After stirring for 15 min, the cooling bath was removed, and the ammonia was allowed to evaporate under a nitrogen stream. The resulting residue was dissolved in methanol (120 mL), and 2 M aqueous HCl (120 mL) was added. The mixture was stirred for 10 min and then quenched with saturated aqueous $NaHCO_3$ (400 mL). The aqueous layer was extracted with DCM (3 × 200 mL), and the combined organic extracts were washed with H_2O (200 mL) and brine (200 mL), dried over Na_2SO_4, filtered, and concentrated under reduced pressure. The desired product **40** was obtained as a white solid (3.54 g, 92%) and was used in the next step without further purification.

^{1}H NMR (600 MHz, C_6D_6) δ 3.44 (td, *J* = 8.2,4.1 Hz, 1H), 2.59 – 2.43 (m, 2H), 2.30 – 2.19 (m, 1H), 2.16 – 1.98 (m, 2H), 1.89 (qt, *J* = 11.1, 4.2 Hz, 2H), 1.81 – 1.63 (m, 2H), 1.56 (ddt, *J* = 13.7, 4.9, 2.4 Hz, 2H), 1.49 (ddt, *J* = 12.6, 6.5, 1.8 Hz, 1H), 1.43 – 1.33 (m, 3H), 1.19 – 1.07 (m, 2H), 0.99 – 0.90 (m, 3H), 0.82 (ddd, *J* = 23.8, 12.0, 6.1 Hz, 2H), 0.72 (s, 3H).
^{13}C NMR (151 MHz, C_6D_6) δ 208.2, 130.9, 126.7, 81.7, 49.9, 46.4, 44.7, 43.8, 39.3, 39.1, 37.4, 31.1, 30.8, 27.7, 26.8, 25.3, 23.3, 11.5.
HRMS (ESI): calculated for $C_{18}H_{27}O_2^+$ [M+H]$^+$ 275.2006, found 275.2004.

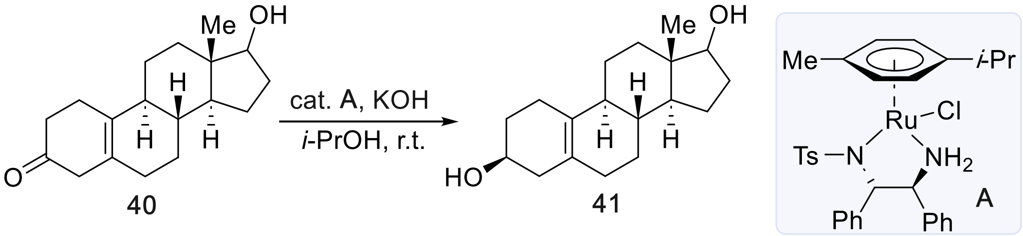

A solution of the **40** (1.00 g, 3.64 mmol, 1.0 equiv) and catalyst **A** (0.58 g, 0.91 mmol, 0.25 equiv) in *i*-PrOH (110 mL) was prepared, and a solution of KOH (0.05 g, 0.91 mmol, 0.25 equiv) in *i*-PrOH (4 mL) was added in one portion under stirring at room temperature. The reaction mixture was stirred overnight at room temperature. It was then diluted with EtOAc (200 mL) and washed with 1 M aqueous HCl (1 × 100 mL). The aqueous phase was extracted with EtOAc (2 × 50 mL), and the combined organic layers were washed sequentially with 1 M aqueous HCl (3 × 60 mL), saturated $NaHCO_3$ (3 × 60 mL), and brine (1 × 100 mL). The organic layer was dried over Na_2SO_4, filtered, and concentrated with Celite, then loaded directly onto a silica gel column. Purification by flash column chromatography (1:4 to 1:1, EtOAc:hexanes) afforded **41** (901.5 mg, 90%) as a white solid with a diastereomeric ratio of 10:1 (β:α), as determined by ^1H and ^{13}C NMR spectroscopy, in good agreement with previously reported data.[5]

^{1}H NMR (600 MHz, $CDCl_3$) δ 4.06 (ttd, *J* = 6.4, 4.2, 2.3 Hz, 1H), 3.68 (td, *J* = 8.6, 5.9 Hz, 1H), 2.29 – 2.21 (m, 1H), 2.18 (d, J = 3.9 Hz, 1H), 2.12 – 2.04 (m, 1H), 2.04 – 1.98 (m, 1H), 1.98 – 1.93 (m, 1H), 1.92 – 1.80 (m, 4H), 1.77 (dtdd, *J* = 13.7, 6.0, 4.3, 1.5 Hz, 1H), 1.70 – 1.65 (m, 2H), 1.65 – 1.58 (m, 1H), 1.58 – 1.55 (m, 1H), 1.49 – 1.40 (m, 2H), 1.37 – 1.27 (m, 3H), 1.21 (qd, *J* = 12.9, 3.4 Hz, 1H), 1.16 – 1.06 (m, 3H), 0.76 (s, 3H).
^{13}C NMR (151 MHz, $CDCl_3$) δ 129.8, 124.6, 82.1, 66.1, 50.1, 46.6, 43.7, 39.3, 39.2, 37.2, 31.3, 30.8, 30.1, 26.9, 25.3, 23.1, 22.6, 11.4.
HRMS (ESI): calculated for $C_{18}H_{29}O_2^+$ $[M+H]^+$ 277.2162, found 277.2163.

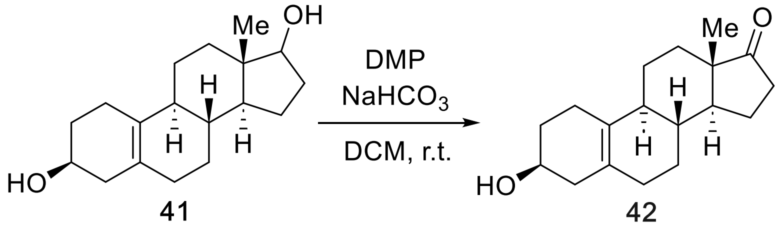

To a stirred solution of **41** (320 mg, 1.16 mmol, 1.0 equiv) and $NaHCO_3$ (292 mg, 3.47 mmol, 3.0 equiv) in dry DCM (200 mL) under a nitrogen atmosphere was added a solution of Dess–Martin periodinane (DMP, 565 mg, 1.33 mmol, 1.15 equiv) in dry DCM (25 mL) via syringe pump at a rate of 12.5 mL/h. Upon complete consumption of the starting material (monitored by TLC), saturated aqueous $NaHCO_3$ (70 mL) and saturated aqueous $Na_2S_2O_3$ (70 mL) were added, and the biphasic mixture was stirred for 2 h. The organic layer was separated, and the aqueous phase was extracted with

DCM (2 × 50 mL). The combined organic layers were washed with brine (100 mL), dried over Na_2SO_4, filtered, and concentrated under reduced pressure. The crude residue was purified by flash column chromatography (1:4 to 2:3, EtOAc:hexanes) to afford the **42** (240 mg, 76%) and an overoxidized diketone byproduct (51.7 mg).

^{1}H NMR (600 MHz, $CDCl_3$) δ 4.07 (s, 1H), 2.46 (dd, *J* = 19.2, 1.2 Hz, 1H), 2.30 – 2.22 (m, 1H), 2.19 (dd, *J* = 17.8, 3.6 Hz, 1H), 2.09 (dt, *J* = 19.2, 9.0 Hz, 1H), 2.00 – 1.94 (m, 3H), 1.92 – 1.75 (m, 5H), 1.70 – 1.60 (m, 2H), 1.59 – 1.53 (m, 1H), 1.52 – 1.44 (m, 2H), 1.41 (ddd, *J* = 12.6, 11.0, 5.7 Hz, 1H), 1.32 (td, *J* = 13.0, 3.8 Hz, 1H), 1.28 – 1.17 (m, 3H), 0.89 (s, 3H).
^{13}C NMR (151 MHz, $CDCl_3$) δ 221.3, 129.4, 124.8, 65.9, 50.4, 48.4, 46.5, 39.2, 38.7, 36.0, 32.0, 31.1, 30.0, 26.2, 24.9, 22.5, 21.6, 14.2.
HRMS (ESI): calculated for $C_{18}H_{27}O_2^+$ $[M+H]^+$ 275.2006, found 275.2009.

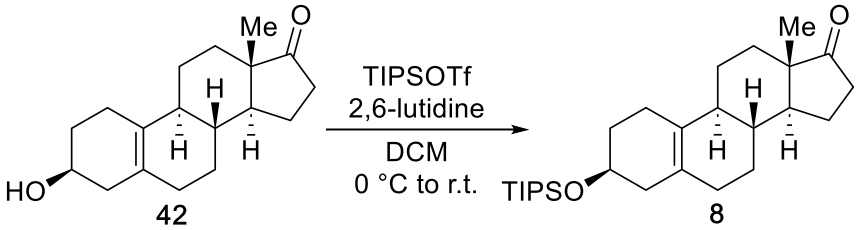

To a stirred solution of **42** (206.3 mg, 0.75 mmol, 1.0 equiv) in anhydrous DCM (15 mL) at 0 °C was added 2,6-lutidine (0.13 mL, 120.8 mg, 1.13 mmol, 1.5 equiv). Triisopropylsilyl trifluoromethanesulfonate (TIPSOTf, 0.26 mL, 299.5 mg, 0.98 mmol, 1.3 equiv) was then added dropwise. The reaction mixture was stirred at 0 °C for 10–15 min, then allowed to warm to room temperature and stirred for an additional 1 h. The reaction was quenched with saturated aqueous $NaHCO_3$ (15 mL), and the layers were separated. The aqueous phase was extracted with DCM (2 × 10 mL), and the combined organic extracts were washed with H_2O (10 mL) and brine (10 mL), dried over anhydrous Na_2SO_4, filtered, and concentrated under reduced pressure. The crude residue was purified by flash column chromatography (100% hexanes, then 98:2 hexanes/MTBE) to afford the TIPS-protected compound **8** as a white oily solid (283.8 mg, 88%).

^{1}H NMR (500 MHz, C_6D_6) δ 4.09 (dq, *J* = 7.0, 2.3 Hz, 1H), 2.37 – 2.28 (m, 1H), 2.17 – 2.07 (m, 3H), 2.06 – 1.98 (m, 1H), 1.93 – 1.68 (m, 7H), 1.67 – 1.56 (m, 2H), 1.48 (ddt, *J* = 14.4, 11.4, 4.5 Hz, 2H), 1.34 – 1.23 (m, 3H), 1.11 – 1.03 (m, 18H), 1.01 – 0.91 (m, 3H), 0.63 (s, 3H).
^{13}C NMR (126 MHz, C_6D_6) δ 217.5, 129.4, 124.5, 66.7, 49.9, 47.6, 46.2, 40.3, 38.5, 35.3, 32.2, 31.3, 31.2, 26.1, 24.9, 23.4, 21.1, 13.6.
HRMS (ESI): calculated for $C_{27}H_{47}O_2Si^+$ $[M+H]^+$ 431.3340, found 431.3337.
$[\alpha]_D^{27}$ = +87.35 (*c* = 1.432 in DCM).
R_f = 0.89 (ethyl acetate in hexanes, 2:8).
IR (film, cm^{-1}): 2962, 2941, 2922, 2890, 2864, 2834, 2183, 1742, 1665, 1619, 1498, 1465, 1407, 1385, 1370, 1291, 1253, 1204, 1159, 1101, 1084, 1068, 1044, 1013, 994, 961, 920, 882, 839, 802, 753, 677, 657, 643, 582, 559, 514, 498, 460, 443.

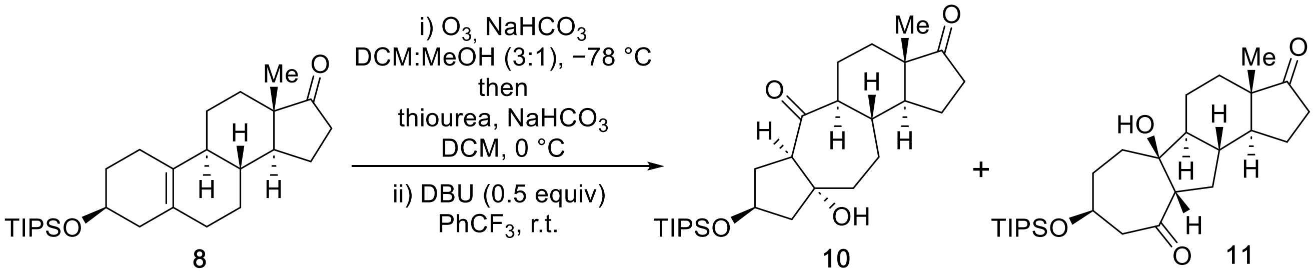

(i) To a solution of compound **8** (67.9 mg, 0.158 mmol, 1.0 equiv) and $NaHCO_3$ (tip of a spatula) in DCM/MeOH (7.9 mL, 3:1) at −78 °C, ozone was bubbled through the mixture until a persistent light-blue color appeared (~ 1 min on this scale). The mixture was then purged with nitrogen until the color disappeared. The resulting solution was transferred via cannula into a separate flask containing thiourea (24.0 mg, 0.315 mmol, 2.0 equiv) and $NaHCO_3$ (13.2 mg, 0.158 mmol, 1.0 equiv) in DCM (2.0 mL) at 0 °C. Upon completion of the addition, the reaction mixture was stirred under nitrogen until judged complete by TLC. The reaction was concentrated under reduced pressure, dissolved in H_2O (10 mL), and extracted with DCM (3 × 5 mL). The combined organic layers were washed with brine (1 × 5 mL), dried over Na_2SO_4, filtered, and concentrated in vacuo.

(ii) The crude residue was dissolved in trifluorotoluene ($PhCF_3$, 7.9 mL), placed under a nitrogen atmosphere, and treated with DBU (11.8 µL, 12 mg, 0.079 mmol, 0.5 equiv) at room temperature. The reaction was stirred until completion by TLC, then quenched with saturated aqueous NH_4Cl (10 mL). The mixture was extracted with DCM (3 × 5 mL), washed with brine (1 × 10 mL), dried over Na_2SO_4, filtered, and concentrated under reduced pressure. Purification by flash column chromatography (1:9 to 3:17, EtOAc:hexanes) afforded compounds **10** (46.2 mg, 63%) and **11** (16.8 mg, 23%) as white solids.

Note: Under the conditions described in the procedure above, side products such as compounds **21** and **22** were also formed, whereas compounds **23** and **24** were detected in addition to **21** and **22** in experiments carried out at reflux in THF or $PhCF_3$ (see Table S1).

^{1}H NMR for 10 (700 MHz, C_6D_6) δ 4.56 (td, J = 9.8, 4.9 Hz, 1H), 2.85 (t, J = 9.1 Hz, 1H), 2.50 – 2.43 (m, 1H), 2.17 (dd, J = 14.5, 10.5 Hz, 1H), 2.14 – 2.07 (m, 2H), 2.04 (dt, J = 13.7, 7.0 Hz, 1H), 1.96 (dd, J = 13.0, 6.1 Hz, 1H), 1.86 – 1.81 (m, 1H), 1.76 (dt, J = 18.8, 9.3 Hz, 1H), 1.66 (dt, J = 15.5, 7.9 Hz, 2H), 1.48 (tq, J = 12.2, 6.9, 5.7 Hz, 1H),1.42 (t, J = 12.6 Hz, 1H), 1.39 – 1.34 (m, 1H), 1.34 – 1.29 (m, 1H), 1.26 (t, J = 13.7 Hz, 1H), 1.17 (d, J = 16.6 Hz, 1H), 1.14 (d, J = 6.9 Hz, 23H), 1.09 (d, J = 7.7 Hz, 2H), 1.06 – 0.95 (m, 1H), 0.83 (td, J = 11.9, 5.9 Hz, 1H), 0.68 (s, 3H).

^{13}C NMR for 10 (126 MHz, C_6D_6) δ 218.3, 210.4, 77.6, 71.6, 62.2, 57.2, 53.7, 50.5, 47.6, 40.2, 39.6, 36.2, 35.4, 30.9, 26.6, 23.7, 22.2, 18.4, 13.6, 12.6.

HRMS (ESI) **for 10**: calculated for $C_{27}H_{47}O_4Si^+$ $[M+H]^+$ 463.3238, found 463.3228.

$[\alpha]_D^{27}$ **for 10** = +20.3 (c = 0.22 in DCM).

R$_f$ for 10 = 0.20 (ethyl acetate in hexanes, 3:7).

IR (film, cm^{-1}) **for 10**: 2960, 2923, 2865, 2852, 2151, 2038, 2007, 1971, 1945, 1744, 1728, 1706, 1496, 1460, 1407, 1376, 1308, 1277, 1256, 1238, 1209, 1179, 1122, 1100, 1073, 1047, 1013, 994, 968, 920, 883, 854, 821, 779, 749, 702, 686, 658, 573, 538, 515, 418.

^{1}H NMR for 11 (700 MHz, C_6D_6) δ 3.90 (td, *J* = 8.0, 3.8 Hz, 1H), 3.10 (t, *J* = 8.5 Hz, 1H), 2.77 –2.70 (m, 2H), 2.14 – 2.04 (m, 2H), 1.93 – 1.89 (m, 1H), 1.87 – 1.77 (m, 2H), 1.74 – 1.64 (m, 2H), 1.58 (ddd, *J* = 12.3, 8.0, 5.8 Hz, 1H), 1.54 (ddd, *J* = 15.3, 6.6, 2.4 Hz, 1H), 1.48 (ddd, *J* = 12.2, 8.6, 5.9 Hz, 1H), 1.28 (qd, *J* = 12.9, 4.0 Hz, 1H), 1.21 – 1.11 (m, 3H), 1.11 – 0.99 (m, 21H), 0.93 (dddd, *J* = 17.9, 14.7, 11.6, 4.1 Hz, 3H), 0.66 (s, 3H), 0.46 (td, *J* = 12.0, 4.0 Hz, 1H).
^{13}C NMR for 11 (176 MHz, C_6D_6) δ 217.5, 206.7, 80.2, 70.5, 64.1, 57.4, 53.6, 50.9, 48.8, 39.1, 35.7, 33.3, 32.6, 31.6, 28.3, 22.4, 19.3, 18.3, 14.0, 12.6.
HRMS (ESI) **for 11**: calculated for $C_{27}H_{47}O_4Si^+$ $[M+H]^+$ 463.3238, found 463.3234.
$[\alpha]_D^{24}$ **for 11** = +29.7 (*c* = 0.53 in DCM).
R_f for 11 = 0.29 (ethyl acetate in hexanes, 3:7).
IR (film, cm^{-1}) **for 11**: 2963, 2942, 2925, 2865, 1740, 1703, 1689, 1601, 1467, 1459, 1371, 1345, 1242, 1087, 1066, 1013, 995, 882, 819, 765, 683, 657.

Note: To confirm the regio- and stereoselectivity of the aldol step, compounds **10** and **11** were converted into the corresponding *p*-nitrobenzoate derivatives **19** and **20**, whose structures were established by single-crystal X-ray diffraction analysis (see X-ray crystallography section).

^{1}H NMR for 18 (500 MHz, C_6D_6) δ 4.59 (tt, *J* = 10.5, 3.3 Hz, 1H), 2.89 (dd, *J* = 15.9, 10.8 Hz, 1H), 2.47 (dd, *J* = 15.9, 3.0 Hz, 2H), 2.37 – 2.28 (m, 1H), 2.23 (dd, *J* = 17.1, 11.9 Hz, 1H), 2.06 (dd, *J* = 19.1, 8.9 Hz, 1H), 2.00 – 1.92 (m, 3H), 1.89 – 1.76 (m, 2H), 1.76 – 1.60 (m, 3H), 1.44 (ddd, *J* = 12.3, 8.8, 5.9 Hz, 1H), 1.37 – 1.24 (m, 2H), 1.18 – 1.08 (m, 21H), 1.07 – 0.95 (m, 3H), 0.83 (tt, *J* = 11.9, 6.7 Hz, 1H), 0.58 (s, 3H).
^{13}C NMR for 18 (126 MHz, C_6D_6) δ 217.41, 213.42, 210.05, 70.32, 56.99, 52.12, 48.86, 47.30, 42.46, 38.35, 36.97, 35.28, 33.03, 31.79, 27.24, 26.35, 22.35, 18.38, 13.34, 12.71.
HRMS (ESI) **for 18**: calculated for $C_{27}H_{47}O_4Si^+$ $[M+H]^+$ 463.3238, found 463.3329.

Side products formed in the aldol reaction:

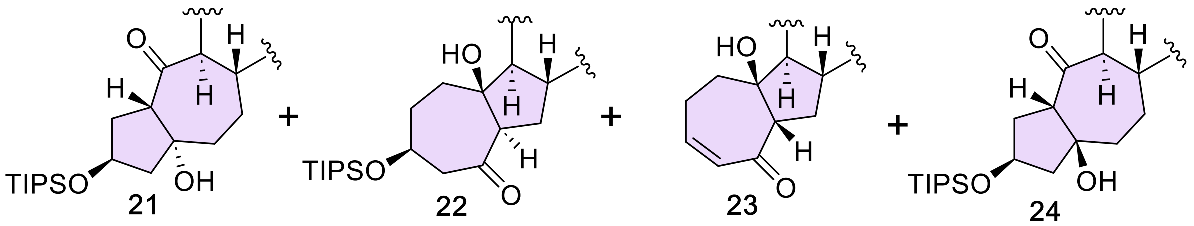

Note: The structures of **21** and **22** were determined by single-crystal X-ray analysis (see X-ray crystallography section). The configuration of compound **23** was assigned by comparison with NMR data reported by Prof. C.-C. Li and coworkers (see pages 30-31).[1] The structure of compound **24** was confirmed by single-crystal X-ray analysis of its deprotected derivative **64** (see X-ray crystallography section).

^{1}H NMR for 21 (700 MHz, C_6D_6) δ 4.48 – 4.42 (m, 1H), 3.39 (dd, *J* = 10.5, 7.9 Hz, 1H), 2.94 (ddd, *J* = 14.0, 10.4, 6.9 Hz, 1H), 2.10 – 2.03 (m, 2H), 2.00 – 1.97 (m, 1H), 1.96 – 1.92 (m, 1H), 1.86 – 1.73 (m, 5H), 1.70 (dt, *J* = 18.6, 9.2 Hz, 1H), 1.48 – 1.40 (m, 4H), 1.20 – 1.13 (m, 3H), 1.12 – 1.08 (m, 18H), 1.07 – 1.01 (m, 3H), 1.00 – 0.95 (m, 1H), 0.81 – 0.75 (m, 1H), 0.50 (s, 3H).
^{13}C NMR for 21 (176 MHz, C_6D_6) δ 217.3, 210.4, 81.4, 71.7, 59.3, 56.7, 55.3, 49.2, 47.7, 42.1, 38.3, 36.4, 35.3, 31.9, 26.8, 26.7, 22.1, 18.3, 18.3, 13.7, 12.4.
HRMS (ESI) **for 21**: calculated for $C_{27}H_{47}O_4Si^+$ $[M+H]^+$ 463.3238, found 463.3232.
R*f* = 0.18 (ethyl acetate in hexanes, 3:7).

^{1}H NMR for 22 (600 MHz, C_6D_6) δ 4.14 (tdd, *J* = 9.4, 3.4, 1.7 Hz, 1H), 2.88 (dd, *J* = 17.6, 9.1 Hz, 1H), 2.78 (ddd, *J* = 12.4, 8.0, 3.9 Hz, 1H), 2.70 (ddd, *J* = 17.6, 3.4, 1.8 Hz, 1H), 2.63 (dd, *J* = 10.8, 3.8 Hz, 1H), 2.11 (ddd, *J* = 18.9, 9.0, 1.2 Hz, 1H), 2.02 (dtd, *J* = 14.5, 10.1, 4.8 Hz, 1H), 1.93 – 1.70 (m, 6H), 1.53 – 1.45 (m, 1H), 1.26 – 1.14 (m, 5H), 1.08 (d, *J* = 6.8 Hz, 18H), 1.06 – 0.99 (m, 3H), 0.99 – 0.93 (m, 1H), 0.88 (dt, *J* = 12.9, 10.7 Hz, 1H), 0.58 (s, 3H), 0.47 (m, 1H).
^{13}C NMR for 22 (151 MHz, C_6D_6) δ 217.4, 204.6, 79.7, 69.5, 60.4, 58.8, 54.1, 51.7, 48.9, 39.2, 35.6, 35.5, 34.7, 31.6, 25.9, 22.4, 19.0, 18.3, 14.1, 12.6.
HRMS (ESI) **for 22**: calculated for $C_{27}H_{47}O_4Si^+$ $[M+H]^+$ 463.3238, found 463.3241.
R*f* = 0.32 (ethyl acetate in hexanes, 3:7).

^{1}H NMR for 23 (500 MHz, $CDCl_3$) δ 6.52 (ddd, *J* = 12.0, 6.7, 2.3 Hz, 1H), 6.07 (dd, *J* = 12.0, 3.4 Hz, 1H), 3.18 – 3.12 (m, 1H), 2.87 – 2.74 (m, 1H), 2.52 – 2.42 (m, 1H), 2.42 – 2.30 (m, 1H), 2.17 – 2.08 (m, 1H), 2.04 – 1.98 (m, 4H), 1.90 – 1.83 (m, 2H), 1.72 – 1.64 (m, 1H), 1.59 – 1.46 (m, 3H), 1.43 (dd, *J* = 12.7, 4.1 Hz, 1H), 1.27 (m, 1H), 0.91 (s, 3H), 0.90 – 0.81 (m, 1H).
^{13}C NMR for 23 (126 MHz, $CDCl_3$) δ 220.3, 201.9, 143.8, 131.8, 81.9, 65.2, 56.4, 51.3, 49.1, 38.8, 36.9, 36.0, 31.0, 26.8, 26.3, 22.6, 19.4, 14.2.
HRMS (ESI) **for 23**: calculated for $C_{18}H_{25}O_3^+$ $[M+H]^+$ 289.1798, found 289.1796.
R*f* = 0.35 (ethyl acetate in hexanes, 6:4).

^{1}H NMR for 24 (700 MHz, C_6D_6) δ 4.66 (s, 1H), 4.27 (t, *J* = 3.8 Hz, 1H), 3.72 (t, *J* = 8.1 Hz, 1H), 2.80 (ddd, *J* = 14.1, 7.8, 4.4 Hz, 1H), 2.42 (qd, *J* = 11.2, 5.7 Hz, 1H), 2.17 (tt, *J* = 12.6, 6.1 Hz, 1H), 2.02 (dd, *J* = 19.0, 9.0 Hz, 1H), 1.97 (dd, *J* = 14.5, 6.3 Hz, 1H), 1.93 – 1.88 (m, 2H), 1.82 – 1.74 (m, 3H), 1.70 (dt, *J* = 18.6, 9.1 Hz, 1H), 1.46 (ddd, *J* = 14.1, 8.5, 6.0 Hz, 1H), 1.33 – 1.20 (m, 4H), 1.19 – 1.13 (m, 1H), 1.05 – 0.97 (m, 18H), 0.97 – 0.91 (m, 3H), 0.91 – 0.84 (m, 1H), 0.79 – 0.73 (m, 1H), 0.56 (s, 3H).
^{13}C NMR for 24 (176 MHz, C_6D_6) δ 217.3, 210.5, 83.6, 75.6, 59.5, 56.3, 50.4, 49.2, 47.6, 35.8, 35.4, 33.8, 33.5, 31.9, 26.7, 26.2, 21.7, 18.1, 13.8, 12.1.
HRMS (ESI) **for 24**: calculated for $C_{27}H_{47}O_4Si^+$ $[M+H]^+$ 463.3238, found 463.3230.
R*f* = 0.46 (ethyl acetate in hexanes, 3:7).

Figure S1. Comparison of ^{1}H NMR spectra of compounds **23**, **S31**, and **S24**.

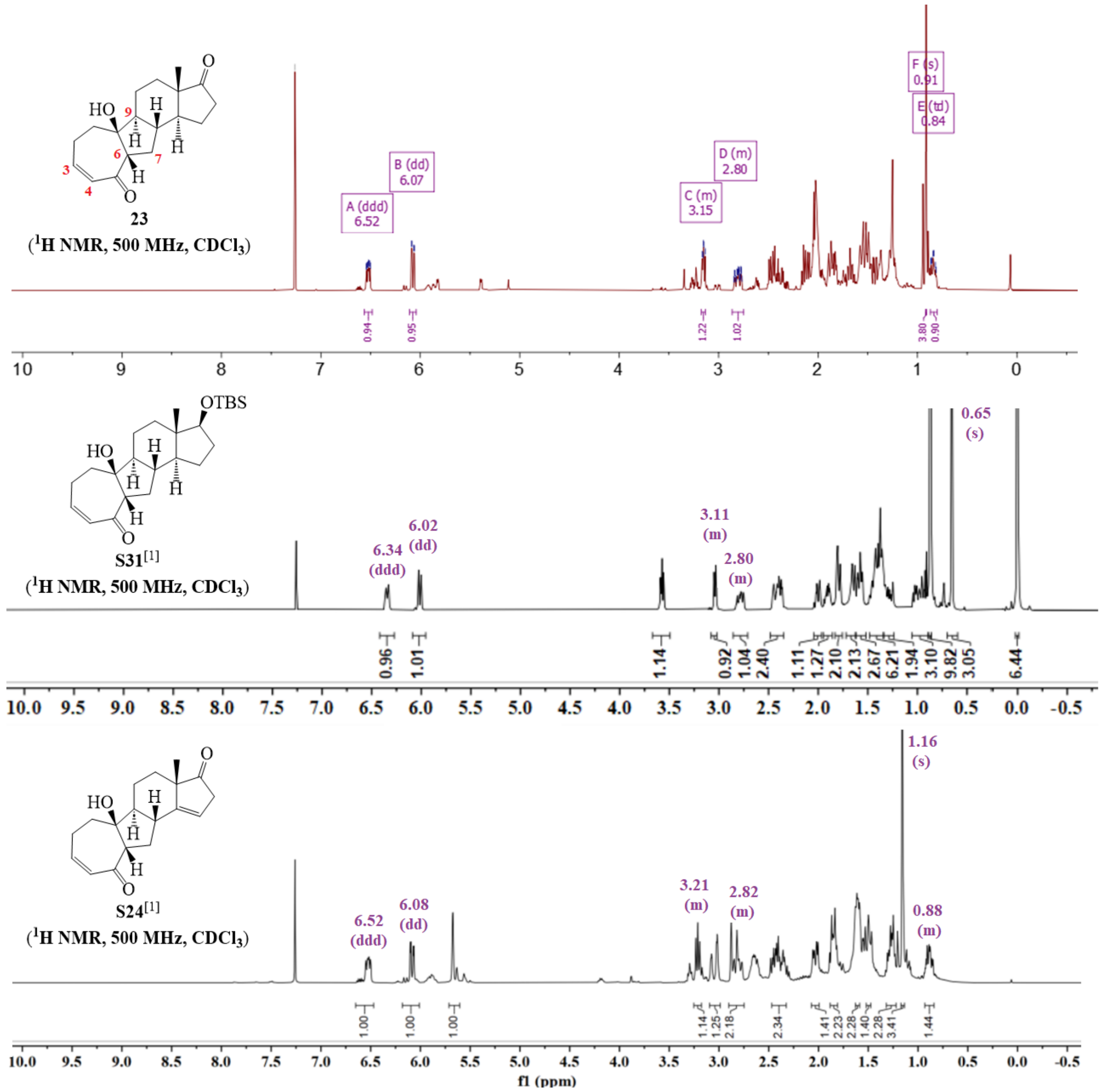

Comparative analysis of the selected proton and carbon NMR signals for compounds **23**, **S31**, and **S24** clearly indicates that compound **23** is a direct structural analog of **S31** and **S24**, both possessing the bufogargarizin A skeleton.

The ¹H NMR spectra of all three compounds (Figure S1) show closely matching resonances at 6.3–6.5 ppm and 6.0–6.1 ppm, corresponding to the C3 and C4 olefinic protons, respectively. In addition, the chemical shifts of one of the C7 methylene protons (~2.8 ppm), the C9 proton (~0.9 ppm) and the C6 proton (3.1–3.2 ppm) are consistent across all three compounds. At the same time, the multiplicities of these selected ¹H NMR peaks remain unchanged.

The ¹³C NMR data also show excellent agreement for the C3, C4, C5, C6, and C10 carbons across all three compounds (Figure S2).

Based on these observations, the configuration of compound **23** was assigned as depicted in the manuscript and Supporting Information.

Note: The NMR spectra of compounds **S24** and **S31** were previously reported by the Li research group.[1] In compound **S31**, the C9 proton resonance presumably overlaps with other signals near 1 ppm.

Figure S2. Comparison of ¹³C NMR spectra of compounds **23**, **S31**, and **S24**.

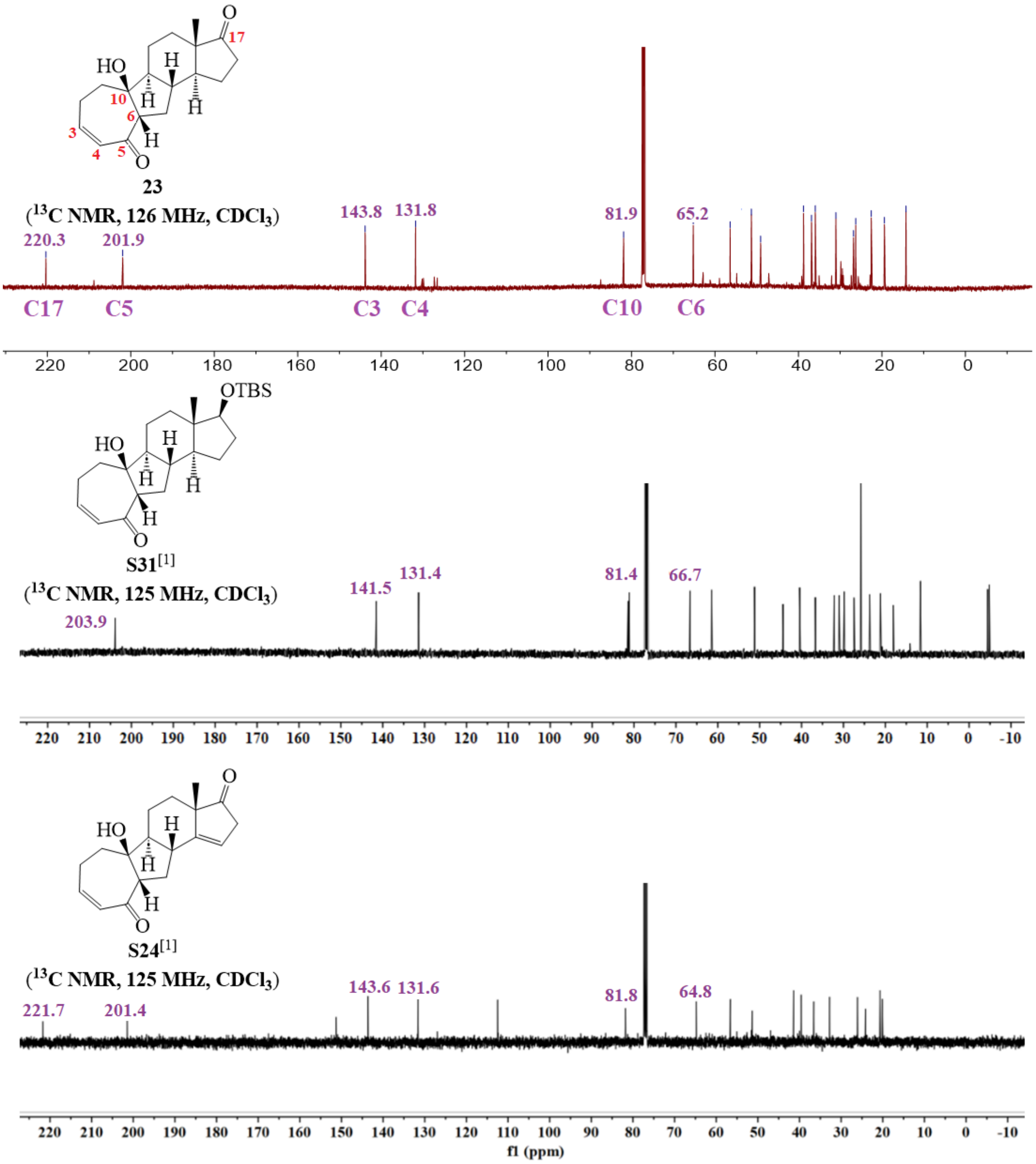

iii. Alternative access to intermediate 8

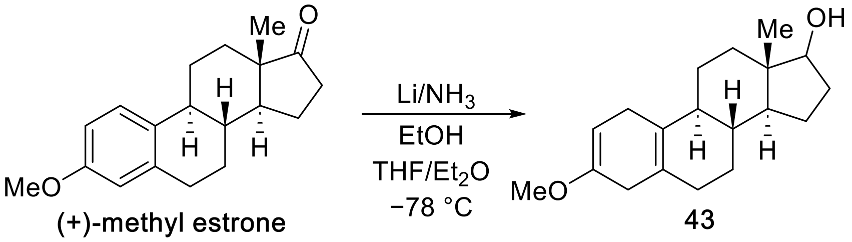

(+)-methyl estrone 43

Gaseous ammonia was condensed into a three-neck round-bottom flask equipped with a stir bar, a dry ice/acetone condenser, and a nitrogen inlet, and maintained at −78 °C. Approximately 100 mL of liquid ammonia was collected, and lithium wire (1.00 g, 141 mmol, 20 equiv; cut into small flat pieces) was added slowly at −78 °C. The lithium was allowed to dissolve in the ammonia for 1.5 h. A suspension of (+)-methyl estrone (2.00 g, 7.00 mmol, 1 equiv) in THF (30 mL) and Et_2O (30 mL) was added to the Li/NH_3 mixture and stirred at −78 °C for 1 h. Ethanol (0.82 mL, 14.1 mmol, 2 equiv) was then added in 0.2 equiv portions (0.16 mL, 1.41 mmol) every 20 min, followed by the slow addition of additional EtOH until the blue color disappeared. The reaction mixture was then stirred overnight at room temperature under a nitrogen stream to allow ammonia and ethanol to evaporate. The resulting residue was dissolved in Et_2O (300 mL) and H_2O (300 mL), and the aqueous layer was extracted with Et_2O (2 × 300 mL). The combined organic layers were washed sequentially with saturated NH_4Cl(aq) (300 mL), H_2O, and brine, dried over Na_2SO_4, and concentrated *in vacuo* to afford compound **43** as a white solid, which was used in the next step without further purification.

^{1}H NMR (600 MHz, C_6D_6) δ 4.58 (t, *J* = 3.3 Hz, 1H), 3.42 (td, *J* = 8.5, 5.6 Hz, 1H), 3.34 (s, 3H), 2.96 – 2.78 (m, 2H), 2.78 – 2.58 (m, 2H), 1.98 – 1.83 (m, 2H), 1.81 – 1.71 (m, 3H), 1.60 – 1.47 (m, 2H), 1.46 – 1.37 (m, 1H), 1.36 – 1.31 (m, 1H), 1.28 (ddd, *J* = 12.2, 9.6, 2.5 Hz, 1H), 1.21 – 1.12 (m, 2H), 1.06 (dq, *J* = 12.3, 6.3 Hz, 1H), 0.96 (td, *J* = 13.6, 13.0, 4.3 Hz, 1H), 0.84 (td, *J* = 11.8, 7.2 Hz, 1H), 0.76 (d, *J* = 5.7 Hz, 1H), 0.72 (s, 3H).
^{13}C NMR (126 MHz, C_6D_6) δ 153.3, 128.4, 125.1, 90.7, 81.8, 53.6, 50.2, 45.9, 43.6, 39.3, 37.5, 34.9, 31.1, 30.8, 28.9, 27.2, 25.8, 23.3, 11.4.
HRMS (ESI) calculated for $C_{19}H_{29}O_2^+$ $[M+H]^+$ 289.2162, found 289.2159.

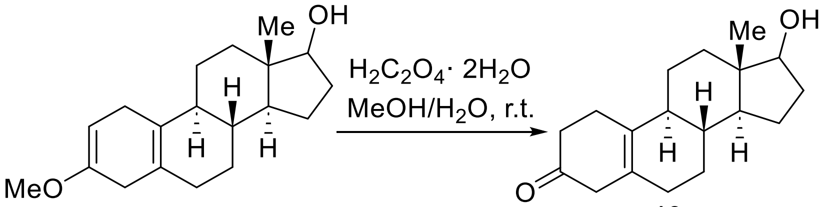

43 40

2 g (1 equiv, 6.93 mmol) of **43** was dissolved in 100 mL of methanol, and then 1.31 g (1.5 equiv, 10.4 mmol) of oxalic acid dihydrate solution in 20 mL water was added

at room temperature. The reaction completion was indicated by TLC after 2 hours. The reaction mixture was dissolved in 100 mL Et_2O, quenched with 100 mL $NaHCO_3$ (aq), and then the aqueous layer was extracted with 2 × 100 mL Et_2O. The organic layers were combined, concentrated, washed with brine, dried with Na_2SO_4, and concentrated *in vacuo*. **40** was obtained as a white solid and used in the next step without purification.

Note: Characterization data for compound **40** are provided above.

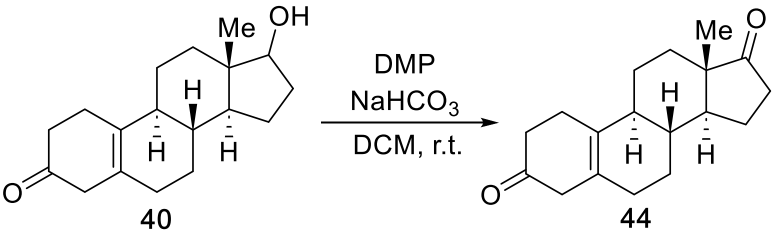

40 44

1.4 g (1 equiv, 5.1 mmol) of **40** was dissolved in dry DCM and 1.3 g of $NaHCO_3$ (3 equiv, 15.3 mmol) were suspended in the solution. A solution of 2.4 g of DMP (1.1 equiv, 5.6 mmol) in DCM was slowly added to the stirred reaction mixture, and then the stirring continued for another 1.5 h until the starting material disappeared indicated by TLC (usually 2 hours). The reaction was quenched by addition of 50 mL of saturated $NaHCO_3$ (aq) and 50 mL of saturated $Na_2S_2O_3$ (aq) solutions. The biphasic mixture was stirred for 1.5 hours. The aqueous layer was extracted with DCM (2 × 100 mL), and the combined organic extracts were washed with brine, dried over Na_2SO_4, and concentrated *in vacuo*. The crude was subjected to column chromatography (1:4, EtOAc:hexanes) to produce the product **44** as white solid in 78% yield over 3 steps.

^{1}H NMR (500 MHz, C_6D_6) δ 2.57 – 2.44 (m, 2H), 2.28 – 2.21 (m, 1H), 2.19 – 2.08 (m, 2H), 2.04 – 1.96 (m, 1H), 1.88 – 1.77 (m, 3H), 1.74 – 1.65 (m, 1H), 1.59 (dd, *J* = 17.0, 5.6 Hz, 1H), 1.53 (dd, *J* = 13.4, 3.4 Hz, 1H), 1.49 – 1.46 (m, 2H), 1.31 (d, *J* = 12.6 Hz, 1H), 1.22 (td, *J* = 13.2, 4.1 Hz, 1H), 1.16 – 1.06 (m, 2H), 0.98 (td, *J* = 11.7, 5.6 Hz, 1H), 0.94 (dt, *J* = 12.5, 6.1 Hz, 1H), 0.85 (qd, *J* = 13.2, 3.9 Hz, 1H), 0.65 (s, 3H).
^{13}C NMR (126 MHz, C_6D_6) δ 217.6, 207.9, 130.6, 126.7, 50.0, 48.0, 46.2, 44.7, 39.0, 38.6, 35.6, 32.4, 30.6, 27.5, 25.9, 24.8, 21.4, 14.0.
HRMS (ESI): calculated for $C_{18}H_{25}O_2^+$ $[M+H]^+$ 273.1849, found 273.1837.
$[\alpha]_D^{27}$ = +231.67 (*c* = 0.29 in DCM).
R*f* = 0.53 (ethyl acetate in hexanes, 2:8).
IR (film, cm^{-1}): 2962, 2925, 2912, 2853, 2830, 2155, 1974, 1738, 1718, 1472, 1453, 1406, 1374, 1339, 1297, 1257, 1212, 1183, 1149, 1119, 1081, 1045, 1002, 961, 918, 871, 836, 580, 543, 503, 479.

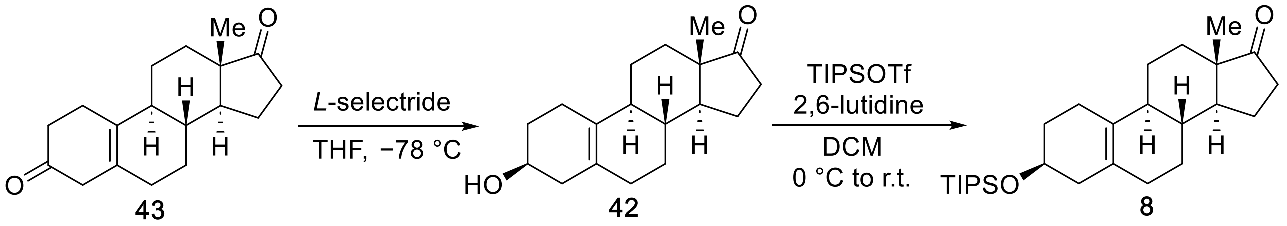

870 mg (1 equiv, 3.19 mmol) of **43** was dissolved in 50 mL of THF and the solution was cooled down to −78 °C. 3.5 mL of 1.0M solution of *L*-selectride in THF (1.1 equiv, 3.5 mmol) was slowly added dropwise, and the reaction was stirred at −78 °C until completion on TLC (usually 1.5 h; **42**, R_f = 0.27 ethyl acetate in hexanes, 2:8). 3.5 mL of 1M NaOH solution and 3.5 mL of 30% H_2O_2 solution were added, the reaction flask was brought to room temperature and stirred for 2 hours. The organic layer was separated, and the aqueous layer was extracted by 2 × 50 mL EtOAc. The organic layers were combined, washed with saturated NH_4Cl (aq), NaCl (aq), dried by Na_2SO_4, concentrated *in vacuo* to give a crude product **42** that was carried to the next step without purification.

Crude **42** was dissolved in 50 mL of DCM, and 554 μL (1.5 equiv, 4.79 mmol, 513 mg) of distilled 2,6-lutidine was added. The mixture was cooled down to 0 °C and then 1.11 mL (1.3 equiv, 4.15 mmol, 1272 mg) of TIPSOTf was added. The mixture was allowed to warm up to room temperature and stirred until completion was indicated by TLC (usually 2 hours). The reaction was then quenched with 50 mL of saturated $NaHCO_3$ (aq) and extracted with 3 × 50 mL DCM. The combined organic layers were washed with NaCl (aq), dried with Na_2SO_4, and concentrated *in vacuo*. Column chromatography (100% hexanes, then 98:2 hexanes/MTBE) gave compound **8** in 78% yield with 1:3 α/β (1.07 g) over 2 steps.

Note: Characterization data for compound **8** are provided above.

iv. Synthesis of bufogargarizin B

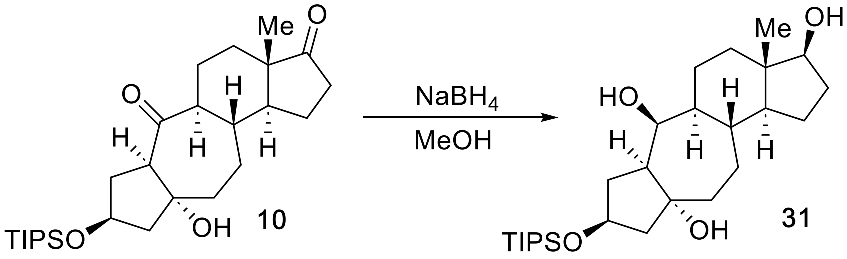

100 mg (1 equiv, 0.216 mmol) of **10** was dissolved in 100 mL of MeOH into a round bottom flask equipped with a stir bar and placed under an atmosphere of nitrogen. 164 mg (20 equiv, 4.32 mmol) of $NaBH_4$ was added in portions and stirred for 1 h. The reaction was monitored by TLC and after completion (usually 1 hour) was concentrated in vacuo, quenched with 30 mL of saturated NH_4Cl (aq) and extracted with 3 × 30 mL EtOAc. The combined organic layers were washed with NaCl (aq), dried with Na_2SO_4, and concentrated *in vacuo*. Crude **31** possessed sufficient purity based on NMR and used in the next step without additional purification.

^{1}H NMR (500 MHz, $CDCl_3$) δ 4.51 (tt, *J* = 6.2, 2.8 Hz, 1H), 3.72 (s, 1H), 3.66 – 3.56 (m, 2H), 2.50 (ddd, *J* = 14.6, 9.1, 6.0 Hz, 1H), 2.24 – 2.11 (m, 2H), 2.06 (dd, *J* = 13.6, 9.0 Hz, 2H), 1.97 – 1.80 (m, 3H), 1.77 (dt, *J* = 12.6, 3.3 Hz, 1H), 1.72 – 1.54 (m, 5H), 1.44 (tdt, *J* = 12.4, 8.4, 3.5 Hz, 2H), 1.32 (tt, *J* = 12.2, 6.0 Hz, 1H), 1.25 (s, 1H), 1.13 – 1.09 (m, 4H), 1.06 (d, *J* = 6.2 Hz, 21H), 0.80 (s, 3H).
^{13}C NMR (126 MHz, $CDCl_3$) δ 84.4, 82.2, 78.8, 74.0, 56.4, 55.3, 53.1, 50.7, 43.3, 40.8, 38.9, 37.0, 30.7, 29.9, 29.4, 27.5, 24.1, 18.1, 12.1, 11.4.
HRMS (ESI): calculated for $C_{27}H_{51}O_4Si^+$ [M+H]$^+$ 467.3551, found 467.3538.
R$_f$ = 0.41 (ethyl acetate in hexanes, 1:1).

Note: The structure of compound **31** was confirmed by single-crystal X-ray diffraction analysis (see X-ray crystallography section). Suitable crystals were grown from a methanol/DCM mixture (1:3) at 25 °C via slow evaporation.

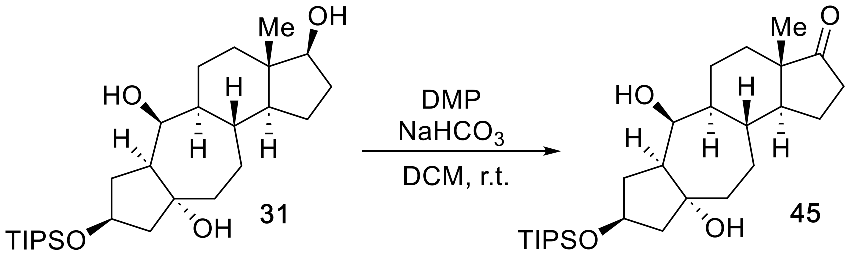

31 (1 equiv, 0.216 mmol) was dissolved in dry DCM (100 mL) and 54 mg of $NaHCO_3$ (3 equiv, 0.648 mmol) under an atmosphere of nitrogen. A diluted solution of 96 mg (1.1 equiv, 0.227 mmol) DMP in 5 mL of DCM (20 mg/mL) was added via syringe pump at 5 mL/hour rate. The reaction was quenched after completion indicated by TLC with 50 mL of 0.5M $Na_2S_2O_3$ and 50 mL of saturated $NaHCO_3$ (aq) and stirred at room temperature for 2 hours. The mixture was extracted with DCM (3 × 100 mL), washed with 50 mL of NaCl (aq), dried with Na_2SO_4, and concentrated *in vacuo*. Column chromatography (1:4 to 1:1, EtOAc:hexanes) gave 79% yield (79 mg) of **45** over 2 steps as a white solid.

^{1}H NMR (500 MHz, C_6D_6) δ 4.43 (tt, *J* = 6.4, 3.8 Hz, 1H), 3.51 (s, 1H), 3.41 (s, 1H), 2.45 (ddt, *J* = 14.3, 8.9, 4.7 Hz, 1H), 2.30 (dd, *J* = 14.3, 11.6 Hz, 1H), 2.19 – 2.05 (m, 2H), 1.97 (dd, *J* = 9.3, 3.7 Hz, 1H), 1.92 (d, *J* = 5.3 Hz, 1H), 1.90 – 1.70 (m, 5H), 1.67 (dt, *J* = 14.0, 3.6 Hz, 1H), 1.58 (ddd, *J* = 14.0, 8.6, 5.7 Hz, 1H), 1.50 (ddd, *J* = 14.5, 8.1, 2.5 Hz, 1H), 1.37 – 1.27 (m, 3H), 1.15 (q, *J* = 6.8, 5.0 Hz, 3H), 1.12 – 0.98 (m, 21H), 0.89 (dq, *J* = 10.6, 6.3, 4.5 Hz, 1H), 0.73 (s, 3H).
^{13}C NMR (176 MHz, C_6D_6) δ 218.9, 83.2, 78.4, 74.1, 56.2, 55.5, 53.0, 51.0, 48.0, 43.1, 40.7, 38.1, 35.9, 32.3, 29.2, 26.9, 22.4, 18.2, 14.0, 12.3.
HRMS (ESI): calculated for $C_{27}H_{49}O_4Si^+$ [M+H]$^+$ 465.3395, found 465.3377.
$[\alpha]_D^{27}$= +30.47 (*c* = 0.09 in DCM).
R$_f$ = 0.59 (ethyl acetate in hexanes, 1:1).
IR (film, cm^{-1}): 2959, 2923, 2865, 2852, 2270, 2244, 2145, 2198, 2168, 2155, 2118, 2038, 2005, 1742, 1603, 1496, 1457, 1376, 1254, 1154, 1104, 1095, 1061,1010, 993, 882, 828, 751, 700, 683, 591, 475, 437, 421.

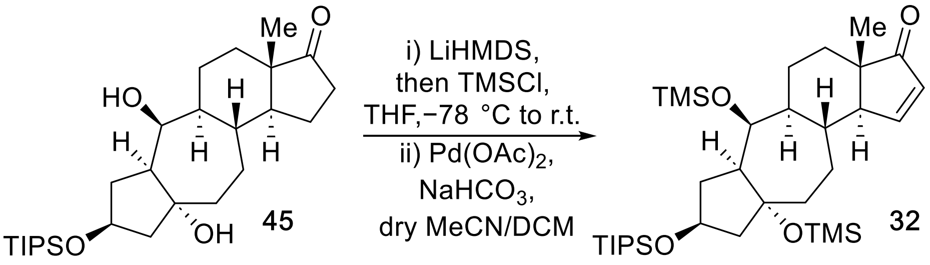

82.8 mg (1 equiv, 0.178 mmol) of compound **45** was dissolved in 4 mL THF and cooled down to −78 °C. 298 mg (10 equiv, 1.78 mmol) of LiHMDS dissolved in 2 mL of dry THF was added at −78 °C. The reaction was allowed to stir for 40 min under an atmosphere of nitrogen, then 0.232 mL (10 equiv, 1.78 mmol, 194 mg) of freshly distilled TMSCl was added. The reaction was stirred for another hour and then warmed to room temperature for another 2 hours. The reaction was quenched with saturated $NaHCO_3$ (aq) and extracted with 3 × 40 mL of EtOAc. The organic layers were combined, dried with Na_2SO_4, and concentrated in vacuo. The crude material was dissolved in 6 mL of 5:1 dry MeCN and DCM respectively. 48 mg (1.2 equiv, 0.22 mmol) of $Pd(OAc)_2$ and 36 mg (2.4 equiv, 0.42 mmol) of solid $NaHCO_3$ were added to the solution under nitrogen. The reaction was stirred for 18 h and then filtered through celite and washed with EtOAc. The crude material was concentrated *in vacuo* and chromatographed on a Et_3N-treated column (100% hexanes, then 10:90 EtOAc/hexanes) to produce **32** as a white solid in 68% (73 mg) yield.

^{1}H NMR (700 MHz, acetone-*d*$_6$) δ 7.78 (d, *J* = 6.1 Hz, 1H), 5.97 (dd, *J* = 6.2, 3.1 Hz, 1H), 4.63 – 4.37 (m, 1H), 3.62 (s, 1H), 2.40 – 2.30 (m, 2H), 2.14 – 2.08 (m, 2H), 2.08 – 1.99 (m, 3H), 1.91 (td, *J* = 12.2, 11.7, 5.9 Hz, 2H), 1.82 (td, *J* = 14.0, 13.6, 5.5 Hz, 1H), 1.74 (dd, *J* = 11.5, 4.7 Hz, 1H), 1.61 – 1.53 (m, 3H), 1.49 – 1.41 (m, 1H), 1.29 (td, *J* = 11.6, 10.2, 4.6 Hz, 1H), 1.10 (d, *J* = 3.0 Hz, 21H), 1.05 (s, 3H), 0.23 – 0.22 (m, 8H), 0.14 (d, *J* = 1.6 Hz, 8H).
^{13}C NMR (176 MHz, acetone-*d*$_6$) δ 212.1, 159.6, 132.1, 85.8, 80.8, 72.9, 57.3, 57.1, 54.4, 52.8, 51.6, 41.1, 38.6, 35.7, 30.1, 29.4, 27.4, 21.0, 18.5, 13.0, 2.8, 1.5.
HRMS (ESI): calculated for $C_{33}H_{63}O_4Si_3^+$ $[M+H]^+$ 607.4029, found 607.4028.
$[\alpha]_D^{27}$= -33.70 (*c* = 0.696 in DCM).
R*f* = 0.81 (ethyl acetate in hexanes, 2:8).
IR (film, cm^{-1}): 3827, 3760, 3602, 3510, 2960, 2923, 2894, 2865, 2852, 2514, 2483, 2359, 2317, 2258, 2227, 2209, 2181, 2141, 2113, 2090, 2049, 2030, 2001, 1982, 1920, 1901, 1745, 1718, 1464, 1440, 1377, 1310, 1251, 1181, 1165, 1145, 1116, 1098, 1061, 1019, 953, 919, 883, 866, 838, 816, 753, 682, 655, 581, 558, 465, 441.

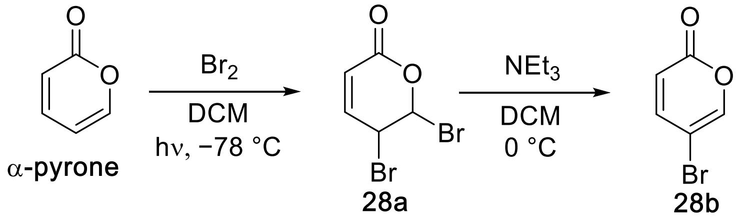

1 g of **α-pyrone** (1 equiv, 10.4 mmol) was dissolved in 15 mL of DCM in a 25×150 mm test tube equipped with a stir bar, capped with a rubber septum and the solution was cooled down to −78 °C under N_2 atmosphere. A 400 W flood light lamp was placed 5 cm away from the solution and turned on. A solution of 0.65 mL (1.2 equiv, 12.5 mmol, 2.00 g) Br_2 in 5 mL DCM was added dropwise over 1 hour. After 7 hours the reaction was completed, warmed to room temperature, concentrated *in vacuo* to give **28a**.

Crude **28a** was then dissolved in DCM, the solution was cooled down to 0 °C, and 7.25 mL (5 equiv, 52 mmol, 5.26 g) of triethylamine was added dropwise. After 1.5 h stirring the reaction was completed indicated by TLC, and the reaction was concentrated in vacuo. The residue was dissolved in Et_2O, filtered, and concentrated *in vacuo*. Column chromatography (100% hexanes, then 30:70 EtOAc/hexanes) gave 85% yield (1.55 g) of **28b**. Spectral data corresponds to previously reported results.[6]

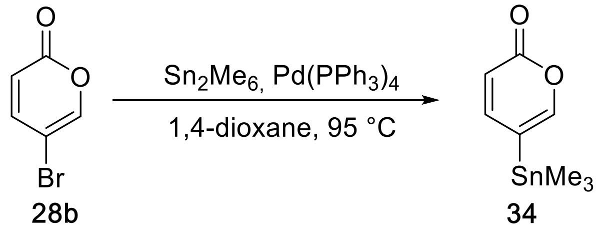

A round bottom flask, containing a stir bar and 720 mg (1 equiv, 4.1 mmol) of **28b**, was placed in a glovebox. 473 mg (0.1 equiv, 0.41 mmol) of $Pd(PPh_3)_4$ was weighed into the flask, which was then capped with a rubber septum, removed from the glove box and placed under a N_2 atmosphere. A solution of 1.7 mL (2 equiv, 8.2 mmol, 2687 mg) of Sn_2Me_6 in 50 mL of 1,4-dioxane was added, after which the reaction mixture was heated to 95 °C for 16 hours. The solution was concentrated *in vacuo* and chromatographed (100% hexanes, then 30:70 EtOAc/hexanes), allowing 77% yield (821 mg) of **34**. Spectral data corresponds to previously reported results.[6]

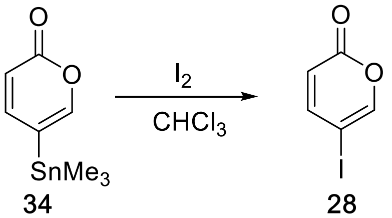

250 mg (1 equiv, 0.965 mmol) of **34** was dissolved in 10 mL of chloroform. The flask was wrapped with aluminum foil, and a solution of 250 mg (1.02 equiv, 0.984 mmol) of I_2 in 10 mL of chloroform was added. The reaction was monitored by ^1H NMR. After 20 h the starting material disappeared, and the reaction was quenched with 10 mL of 0.5M $Na_2S_2O_3$ solution. The organic layer was separated, dried with Na_2SO_4, and

concentrated *in vacuo*. Column chromatography (1:9; EtOAc:hexanes) gave 70% yield (150 mg) of **28**.

^{1}H NMR (500 MHz, C_6D_6) δ 6.50 (dd, *J* = 2.6, 1.1 Hz, 1H), 6.13 (dd, *J* = 9.8, 2.6 Hz, 1H), 5.40 (dd, *J* = 9.8, 1.1 Hz, 1H).

^{13}C NMR (126 MHz, C_6D_6) δ 158.2, 154.1, 148.3, 117.8, 67.0.

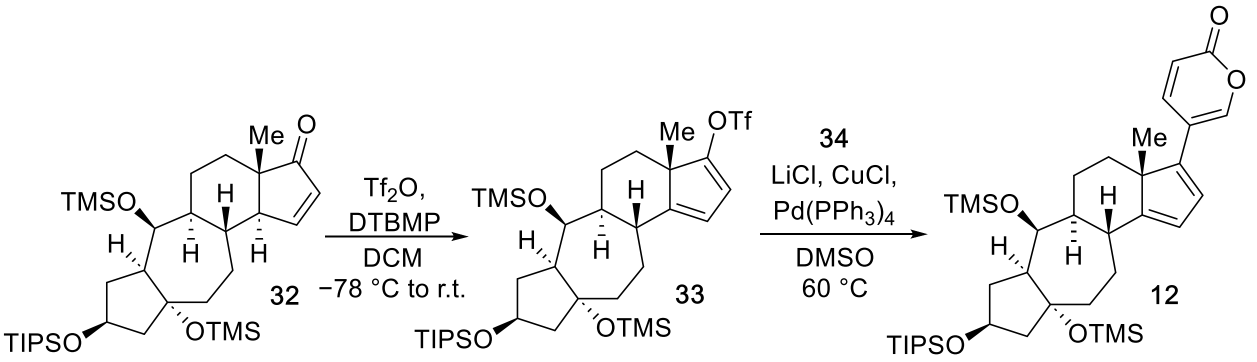

83 mg (1 equiv, 0.136 mmol) of **32** was dissolved in 1 mL DCM and 280 mg (10 equiv, 1.36 mmol) of di-*tert*-butylmethylpyridine (DTBMP) was added to the solution. The mixture was cooled down to −78 °C, and then 46 µL (2 equiv, 0.273 mmol. 76.6 mg) of Tf_2O was added. The stirring continued for another 5 minutes and then the solution was brought to room temperature. After the starting material was consumed via TLC monitoring (usually 10 mins; **33**, R_f = 0.87, ethyl acetate in hexanes, 3:7) the reaction was quenched with 1 mL saturated $NaHCO_3$ (aq), extracted with 3 × 1 mL DCM, organic layers were combined, dried with Na_2SO_4, and concentrated *in vacuo*. The crude **33** is used for the next step unpurified.

To a flame-dried flask with a stir bar 176 mg (5 equiv, 0.68 mmol) of **34** was added. The flask was brought to the glove box. 15.7 mg (0.1 equiv, 0.0136 mmol) of $Pd(PPh_3)_4$, 67 mg (5 equiv, 0.68 mmol) of CuCl and 57 mg (10 equiv, 1.36 mmol) of LiCl were added. The flask was capped with a septum and brought out of the glovebox. Under nitrogen atmosphere the solution of crude **33** in 7 mL of dry DMSO was added, then nitrogen was bubbled through the suspension for 1 hour at room temperature. The reaction mixture was then stirred under N_2 atmosphere at 60 °C and monitored by TLC. After disappearance of starting material (usually 3 hours), the reaction was quenched with 30 mL $NaHCO_3$ (aq), extracted 6 × 30 mL EtOAc, and the combined organic layers were washed sequentially with saturated NH_4Cl (aq), $NaHCO_3$ (aq), NaCl (aq), dried with Na_2SO_4, and concentrated *in vacuo*. Purification by column chromatography (Et_3N-neutralized silica gel; 100% hexanes, then 20:80 EtOAc/hexanes) afforded **12** as a white solid (68.9 mg, 74% yield).

^{1}H NMR (700 MHz, C_6D_6) δ 7.07 (d, *J* = 2.6 Hz, 1H), 6.82 (dd, *J* = 9.7, 2.7 Hz, 1H), 6.13 (d, *J* = 2.3 Hz, 1H), 6.03 – 5.99 (m, 2H), 4.72 (p, *J* = 8.1 Hz, 1H), 3.46 (s, 1H), 2.51 (t, *J* = 11.0 Hz, 1H), 2.45 (q, *J* = 9.8 Hz, 1H), 2.24 (td, *J* = 13.5, 11.3, 8.3 Hz, 2H), 2.13 (t, *J* = 9.0 Hz, 1H), 2.05 (dd, *J* = 14.5, 7.6 Hz, 1H), 1.95 (dt, *J* = 18.5, 10.6 Hz, 2H), 1.78 (t, *J* = 11.4 Hz, 1H), 1.68 (dt, *J* = 12.8, 8.9 Hz, 1H), 1.58 (td, *J* = 13.3, 3.6 Hz, 1H), 1.47 – 1.40 (m, 1H), 1.21 (t, *J* = 5.9 Hz, 21H), 1.05 – 0.97 (m, 2H), 0.88 (s, 3H), 0.79 (td, *J* = 13.2, 3.7 Hz, 1H), 0.30 (s, 9H), 0.28 (s, 9H).

^{13}C NMR (176 MHz, C_6D_6) δ 165.3, 160.1, 147.4, 145.7, 142.6, 126.3, 119.5, 117.0, 114.8, 85.5, 80.0, 72.6, 56.3, 55.9, 52.8, 52.3, 40.9, 38.2, 38.1, 36.7, 29.7, 26.7, 19.9, 18.4, 18.4, 12.7, 2.8, 1.3.
HRMS (ESI): calculated for $C_{38}H_{65}O_5Si_3^+$ $[M+H]^+$ 685.4134, found 685.4131.
$[\alpha]_D^{27}$ = +136.52 (*c* = 0.44 in DCM).
R*f* = 0.77 (ethyl acetate in hexanes, 3:7).
IR (film, cm^{-1}): 2960, 2943, 2894, 2865, 2183, 2143, 1753, 1739, 1713, 1606, 1546, 1525, 1464, 1436, 1369, 1315, 1250, 1225, 1174, 1154, 1124, 1103, 1064, 1026, 996, 965, 946, 882, 839, 753, 683, 858, 644, 500, 442, 419.

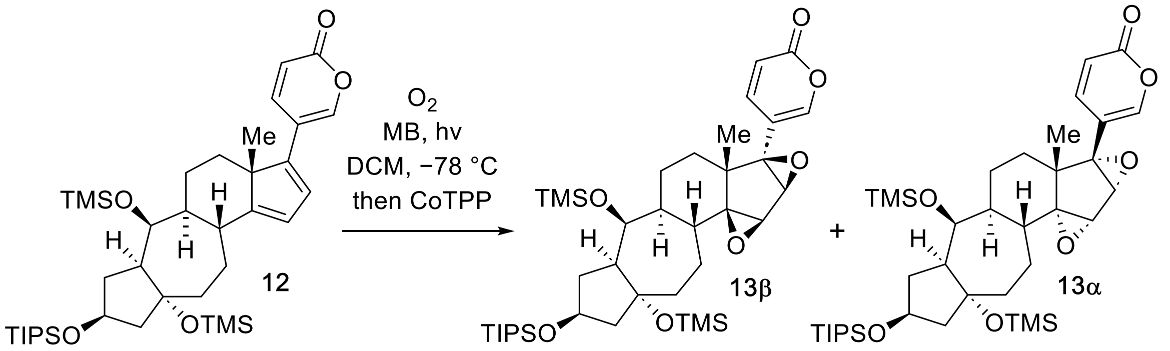

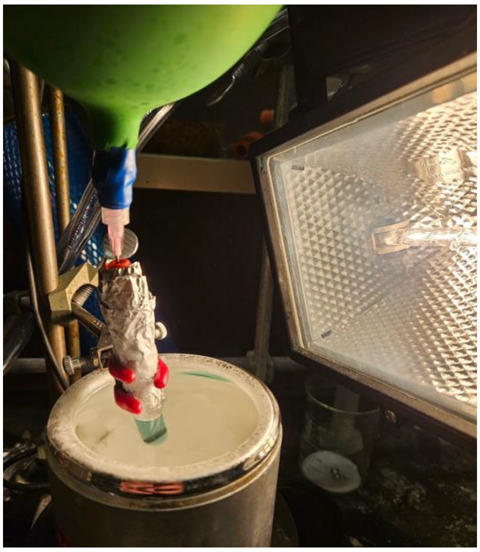

A 13×100 mm test-tube was equipped with a stir bar, then 7.8 mg (2 equiv, 0.0934 mmol) of dry $NaHCO_3$ and 1%w of methylene blue were added. 32 mg (1 equiv, 0.0467 mmol) of **12** was added to the reaction vessel as a solution of 4 mL of dry DCM. The test tube was capped with a septum. Parafilm, and then electric tape were used to seal the septum to the test tube. An oxygen balloon was installed and the space above the solution level was wrapped with aluminum foil. The reaction vessel was then cooled to −78 °C with the solution level completely submerged into the bath. A 400 W flood light lamp was installed 10 cm away from the solution. Upon completion of the reaction, as indicated by TLC (typically 45 min), the oxygen balloon was removed, and the solution was placed under vacuum on a Schlenk line at −78 °C to remove residual oxygen. The vacuum was then released, and a N_2 balloon was installed. A solution of 3.1 mg (0.1 equiv, 0.00467 mmol) of CoTPP in 0.3 mL of DCM was slowly added to the reaction mixture. The CoTPP mediated rearrangement was monitored by TLC and subsequently concentrated *in vacuo* and chromatographed (Et_3N-neutralized silica gel; 100% hexanes, then 30:70 EtOAc/hexanes) to produce the desired compound **13β** in 40% yield and **13α** in 12% yield in 52% yield with 3.3:1, which can be separated by column. Below are the spectral data for the desired and major isomer **13β** and **13α**.

Note: As the reaction is sensitive to overoxidation, it is recommended to turn off the light when taking and developing TLC plates.

^{1}H NMR for 13β (600 MHz, C_6D_6) δ 6.78 (dd, *J* = 2.6, 1.1 Hz, 1H), 6.62 (dd, *J* = 9.7, 2.6 Hz, 1H), 5.88 (dd, *J* = 9.6, 1.1 Hz, 1H), 4.80 – 4.58 (m, 1H), 3.46 (s, 1H), 3.03 (s, 1H), 2.84 (s, 1H), 2.47 (dt, *J* = 12.5, 8.3 Hz, 1H), 2.21 – 2.12 (m, 2H), 2.05 – 1.85 (m, 3H), 1.80 – 1.63 (m, 3H), 1.42 – 1.28 (m, 2H), 1.19 (p, *J* = 4.2, 3.5 Hz, 21H), 1.14 – 1.08 (m, 2H), 1.07 (s, 3H), 0.98 (dt, *J* = 13.0, 3.5 Hz, 1H), 0.78 – 0.65 (m, 1H), 0.23 (s, 18H).
^{13}C NMR for 13β (126 MHz, C_6D_6) δ 159.9, 151.2, 143.0, 116.1, 114.3, 85.7, 80.7, 78.3, 74.6, 72.5, 56.7, 56.4, 54.2, 52.0, 51.2, 40.8, 39.8, 37.6, 37.0, 35.1, 28.0, 21.7, 18.4, 18.4, 15.1, 12.6, 2.7, 1.2.
HRMS (ESI) **for 13β**: calculated for $C_{38}H_{65}O_7Si_3^+$ $[M+H]^+$ 717.4033, found 717.4024.
$[\alpha]_D^{27}$ **for 13β** = +0.358 (*c* = 0.135 in DCM).
R*f* for 13β = 0.51 (ethyl acetate in hexanes, 3:7).
IR (film, cm^{-1}) **for 13β**: 3947, 3933, 3901, 3862, 3837, 3810, 3782, 3750, 3679, 3661, 3589, 3492, 3462, 3417, 3332, 3313, 3263, 3244, 3207, 3131, 2959, 2821, 2866, 2852, 2782, 2614, 2587, 2524, 2493, 2404, 2359, 2242, 2204, 2166, 2152, 2132, 2109, 2087, 2073, 2059, 2037, 2019, 1990, 1962, 1936, 1911, 1881, 1847, 1816, 1749, 1730, 1724, 1638, 1612, 1538, 1501, 1465, 1441, 1375, 1319, 1250, 1232, 1175, 1158, 1127, 1105, 1066, 1050, 1027, 998, 966, 942, 917, 883, 838, 752, 684, 639, 604, 588, 566, 537, 508, 473, 448, 432, 416.

^{1}H NMR for 13α (700 MHz, C_6D_6) δ 6.74 (s, 1H), 6.60 (dt, *J* = 9.6, 2.2 Hz, 1H), 5.92 (d, *J* = 9.6 Hz, 1H), 4.72 – 4.62 (m, 1H), 3.39 (s, 1H), 3.19 (s, 1H), 2.89 (s, 1H), 2.38 (dt, *J* = 12.3, 8.4 Hz, 1H), 2.27 (t, *J* = 11.1 Hz, 1H), 2.15 (ddt, *J* = 59.1, 19.2, 9.2 Hz, 4H), 2.01 – 1.88 (m, 2H), 1.77 – 1.58 (m, 5H), 1.51 (tt, *J* = 17.4, 7.5 Hz, 1H), 1.21 – 1.12 (m, 30H), 1.08 – 1.01 (m, 2H), 0.51 (s, 3H), 0.27 (d, *J* = 1.8 Hz, 8H), 0.25 (d, *J* = 1.8 Hz, 8H).
^{13}C NMR for 13α (176 MHz, C_6D_6) δ159.9, 151.0, 143.0, 116.3, 115.4, 85.3, 80.7, 80.2, 72.5, 71.7, 56.0, 55.5, 55.2, 52.5, 49.3, 40.8, 40.4, 37.4, 36.3, 28.4, 28.2, 23.1, 20.1, 18.4, 18.4, 12.6, 2.8, 1.3.
HRMS (ESI) **for 13α**: calculated for $C_{38}H_{65}O_7Si_3^+$ $[M+H]^+$ 717.4033, found 717.4024.

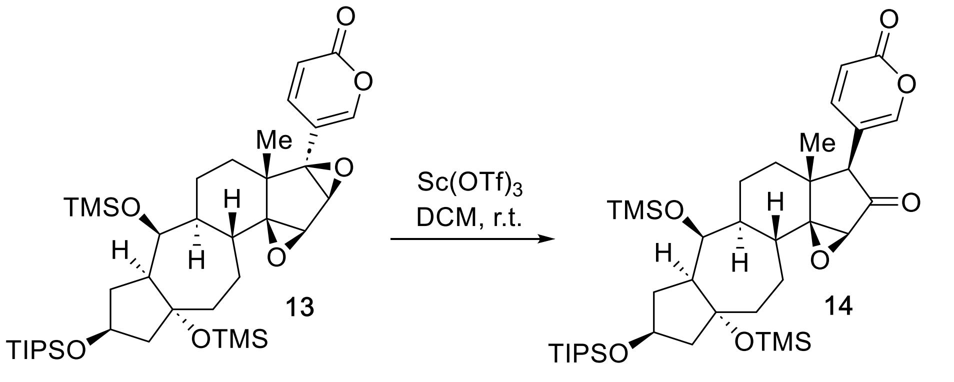

24.9 mg (1equiv, 34.7 μmol) of **13** and 1.7 mg (0.1 equiv, 3.47 μmol) of $Sc(OTf)_3$ was added to a flame dried 2-dram vial equipped with stir bar and 4 Å molecular sieves beads, then dissolved in 2 mL of DCM and placed under an N_2 atmosphere. After 10 min or after completion on TLC, the reaction was quenched with 2 mL of saturated aqueous $NaHCO_3$, extracted with 3 × 2mL of DCM, washed with brine, dried over

Na_2SO_4, and concentrated *in vacuo*. To maximize the yield the crude residue was used in the next step without purification. However, one of the batches was subjected to flash column chromatography and **14** was separated and characterized as shown below.

^{1}H NMR (500 MHz, C_6D_6) δ 7.03 – 6.96 (m, 1H), 6.59 (d, *J* = 2.7 Hz, 1H), 5.86 (d, *J* = 9.9 Hz, 1H), 4.75 – 4.64 (m, 1H), 3.36 (s, 1H), 3.23 (s, 1H), 2.53 – 2.47 (m, 1H), 2.15 – 2.09 (m, 3H), 1.90 (dd, *J* = 15.8, 8.9 Hz, 2H), 1.70 – 1.60 (m, 3H), 1.39 (d, *J* = 1.9 Hz, 2H), 1.22 – 1.10 (m, 21H), 1.09 – 0.99 (m, 1H), 0.96 – 0.92 (m, 3H), 0.87 – 0.81 (m, 1H), 0.57 (d, *J* = 5.8 Hz, 3H), 0.22 (s, 8H), 0.20 (s, 8H).
^{13}C NMR (126 MHz, C_6D_6) δ 206.4, 160.0, 151.5, 144.5, 116.1, 115.9, 85.5, 80.5, 74.8, 72.5, 58.6, 58.4, 55.9, 53.3, 51.8, 50.8, 42.0, 40.9, 40.0, 37.3, 35.6, 30.2, 28.4, 21.8, 18.4, 18.4, 16.7, 12.6, 2.7, 1.2.
HRMS (ESI): calculated for $C_{38}H_{65}O_7Si_3^+$ $[M+H]^+$ 717.4033, found 717.3997.

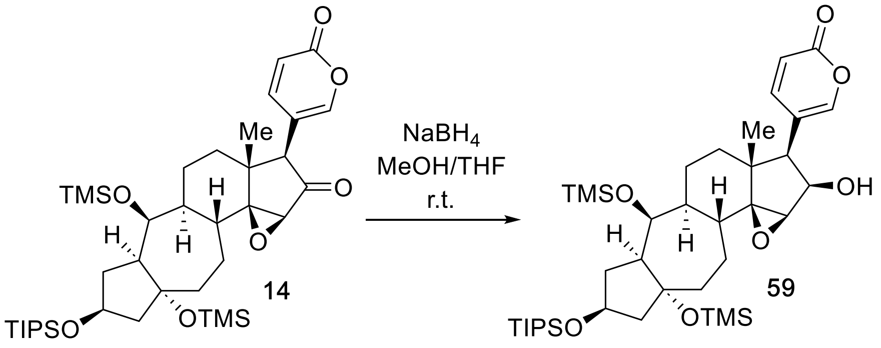

Crude **14** from above was dissolved in a mixture of 2 mL of THF and 1 mL of MeOH. A suspension of 1.44 mg (1.1 equiv, 38.2 μmol) of $NaBH_4$ in 1 mL of THF was added via syringe. The reaction was monitored by TLC (usually 15 mins) and quenched with 3 mL of saturated NH_4Cl (aq) and extracted with 3 × 2 mL EtOAc. The combined organic layers were washed with NaCl (aq), dried with Na_2SO_4, and concentrated *in vacuo*. To maximize the yields the crude residue was used in the next step without purification. However, one of the batches was subjected to flash column chromatography and **59** was separated and characterized as shown below.

^{1}H NMR (500 MHz, C_6D_6) δ 7.87 (d, *J* = 8.9 Hz, 1H), 6.65 (s, 1H), 6.00 (d, *J* = 9.8 Hz, 1H), 4.66 (t, *J* = 9.1 Hz, 1H), 4.15 (d, *J* = 9.1 Hz, 1H), 3.37 (s, 1H), 3.26 (s, 1H), 2.45 (q, *J* = 10.0 Hz, 1H), 2.16 – 1.77 (m, 7H), 1.73 – 1.58 (m, 3H), 1.49 (d, *J* = 12.7 Hz, 1H), 1.22 – 1.03 (m, 21H), 1.03 – 0.84 (m, 5H), 0.58 (s, 3H), 0.19 (s, 8H), 0.17 (s, 8H).
^{13}C NMR (126 MHz, C_6D_6) δ 161.1, 150.8, 148.1, 116.5, 113.7, 85.4, 80.3, 72.2, 71.0, 61.4, 55.7, 52.1, 51.5, 51.2, 44.5, 40.5, 39.4, 37.1, 35.3, 29.8, 28.5, 21.8, 18.0, 18.0, 17.0, 12.2, 2.3, 0.8.
HRMS (ESI): calculated for $C_{38}H_{67}O_7Si_3^+$ $[M+H]^+$ 719.4189, found 719.4186.

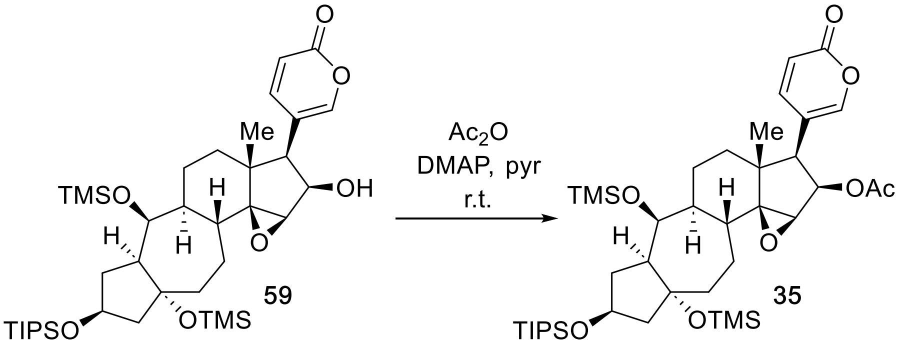

Crude **59** from above was dissolved in 0.7 mL (0.04 M for SM) of pyridine and 1.27 mg of DMAP (0.2 equiv, 10.4 μmol) was added to the mixture under an atmosphere of nitrogen. Then, 16 μL (18 equiv, 173.5 μmol, 17.7 mg) of acetic anhydride was slowly added. After completion by TLC (usually 15 mins) the mixture was quenched with 1 mL of saturated NH_4Cl (aq) and extracted with 3 × 1 mL of EtOAc. The combined organic layers were washed with NaCl (aq), dried with Na_2SO_4, and concentrated *in vacuo*. Column chromatography (100% hexanes, then 20:80 EtOAc/hexanes) produced **35** in 62% (16.5 mg) in 3 steps as a white solid.

^{1}H NMR (700 MHz, C_6D_6) δ 7.83 (s, 1H), 6.81 – 6.57 (m, 1H), 6.03 (d, *J* = 9.8 Hz, 1H), 5.16 (d, *J* = 9.2 Hz, 1H), 4.70 (q, *J* = 8.2 Hz, 1H), 3.40 (s, 1H), 3.28 (s, 1H), 2.50 (q, *J* = 9.7 Hz, 1H), 2.40 (d, *J* = 9.3 Hz, 1H), 2.16 (dt, *J* = 16.8, 7.5 Hz, 2H), 2.07 (dd, *J* = 14.1, 9.5 Hz, 1H), 1.99 (dd, *J* = 14.5, 10.6 Hz, 1H), 1.92 (dd, *J* = 14.5, 8.6 Hz, 1H), 1.85 – 1.77 (m, 1H), 1.75 – 1.61 (m, 2H), 1.47 (s, 3H), 1.32 (s, 3H), 1.24 – 1.11 (m, 21H), 1.10 – 1.04 (m, 1H), 1.00 – 0.97 (m, 1H), 0.92 (t, *J* = 6.8 Hz, 1H), 0.58 (s, 3H), 0.23 (s, 9H), 0.21 (s, 9H).
^{13}C NMR (126 MHz, C_6D_6) δ 169.7, 160.8, 152.1, 147.9, 115.8, 114.3, 85.9, 80.8, 75.1, 72.8, 71.4, 59.5, 56.3, 52.0, 51.6, 50.7, 45.0, 41.1, 39.9, 37.6, 35.8, 30.4, 28.9, 22.3, 20.1, 18.6, 18.6, 17.4, 12.8, 2.9, 1.4.
HRMS (ESI): calculated for $C_{40}H_{69}O_8Si_3^+$ $[M+H]^+$ 761.4295, found 761.4288.
$[\alpha]_D^{27}$ = -1.94 (*c* = 0.47 in DCM).
R*f* = 0.36 (ethyl acetate in hexanes, 2:8).
IR (film, cm^{-1}): 2959, 2924, 2892, 2865, 2851, 2400, 2225, 2205, 2151, 2090, 2051, 2026, 2010, 1971, 1751, 1727, 1638, 1609, 1539, 1466, 1375, 1316, 1251, 1231, 1175, 1148, 1128, 1104, 1067, 1050, 1026, 997, 966, 944, 921, 883, 839, 785, 753, 684, 660, 514, 462, 436, 416.

a. First-generation approach to the completion of bufogargarizin B

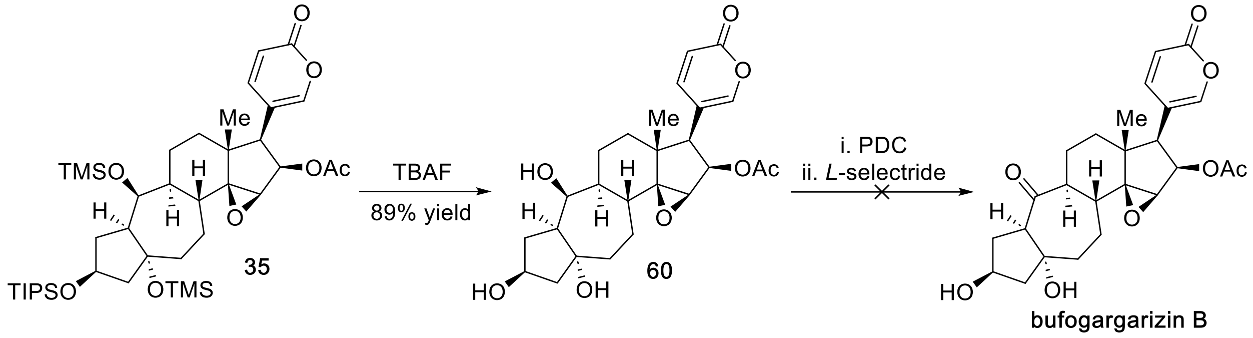

6.5 mg (1 equiv, 8.5 μmol) of **35** was dissolved in 0.5 mL of THF. Then, 60 μL (7 equiv, 60 μmol, 1.0M solution in THF) of TBAF was added, and the reaction was stirred for 12 hours. After the disappearance of starting material, as indicated by TLC, the reaction mixture was quenched by saturated $NaHCO_3$ (aq) and extracted with 3 × 1 mL EtOAc. The combined organic layers were washed with NaCl (aq), dried with Na_2SO_4, and concentrated *in vacuo*. Column chromatography (1:9, MeOH:DCM) allowed 3.5 mg (89%) of **60**.

^{1}H NMR (600 MHz, CD_3OD) δ 8.05 (s, 1H), 7.37 (s, 1H), 6.24 (d, *J* = 9.8 Hz, 1H), 5.47 (dd, *J* = 9.2, 1.3 Hz, 1H), 4.38 – 4.31 (m, 1H), 3.62 (s, 1H), 3.53 (s, 1H), 3.15 – 3.03 (m, 3H), 2.92 (d, *J* = 9.2 Hz, 1H), 2.34 (dd, *J* = 12.7, 7.4 Hz, 1H), 2.17 (td, *J* = 11.4, 2.3 Hz, 1H), 2.02 (t, *J* = 7.9 Hz, 1H), 1.97 – 1.92 (m, 2H), 1.86 (s, 3H), 1.83 – 1.77 (m, 2H), 1.66 – 1.60 (m, 3H), 1.44 – 1.40 (m, 3H), 1.22 – 1.16 (m, 1H), 0.90 (t, *J* = 6.9 Hz, 1H), 0.85 (s, 3H).
^{13}C NMR (176 MHz, CD_3OD) δ 171.6, 164.1, 155.9, 150.9, 114.1, 83.2, 78.5, 76.7, 72.8, 72.3, 60.7, 55.2, 54.1, 53.2, 52.1, 51.4, 46.2, 41.3, 40.7, 39.2, 36.7, 27.1, 22.5, 20.4, 17.6.
HRMS (ESI): calculated for $C_{25}H_{29}O_8^+$ $[M+H]^+$ 461.2170, found 461.2173.

Unfortunately, the subsequent efforts to advance **60** to **bufogargarizin B** via PDC oxidation followed by selective C3-ketone reduction led to the inseparable mixtures of **bufogargarizin B** and unidentified degradation products. Therefore, a different approach described below was pursued.

b. Successful end-game route

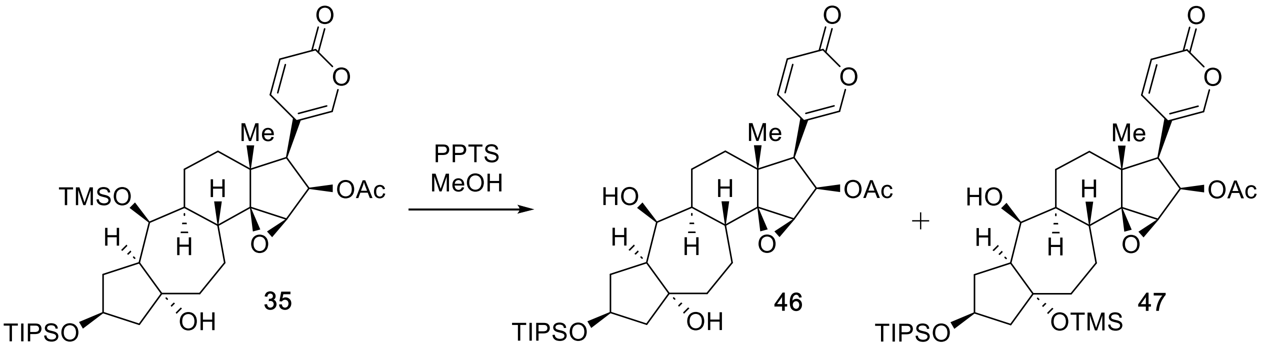

50.1 mg (1 equiv, 0.066 mmol) of **35** was dissolved in 2.2 mL (0.03 M for SM) of dry methanol and placed under an atmosphere of nitrogen. 66 mg of PPTS (4 equiv, 0.26 mmol) was added and the reaction was allowed to stir for 24 hours. After this time, approximately 20% of the starting material remained, and 66 mg of PPTS (4 equiv, 0.26 mmol) was added and the reaction was allowed to stir for another 6 hours. The reaction was then quenched with 2 mL of saturated $NaHCO_3$ (aq). The mixture was then extracted with 3 × 2 mL portions of ethyl acetate, washed with brine, dried with Na_2SO_4, and concentrated *in vacuo*. The resulting crude mixture was subjected to column chromatography to produce 25.7 mg **46** in 63% yield and 6.4 mg of **47** in 14% yield. 6.4 mg of compound **47** was resubjected to the PPTS deprotection conditions to provide an additional 3.5 mg of **46** in 61% yield.

^{1}H NMR for 46 (700 MHz, C_6D_6) δ 7.81 (s, 1H), 6.64 (s, 1H), 6.00 (d, *J* = 9.8 Hz, 1H), 5.22 (d, *J* = 9.2 Hz, 1H), 4.36 (s, 1H), 4.08 (s, 1H), 3.51 (s, 1H), 3.18 (s, 2H), 2.51 (dt, *J* = 14.3, 7.4 Hz, 1H), 2.36 (d, *J* = 9.2 Hz, 1H), 2.31 (t, *J* = 11.3 Hz, 1H), 2.20 – 2.12 (m, 1H), 2.03 (d, *J* = 9.0 Hz, 1H), 1.98 (dd, *J* = 14.6, 6.7 Hz, 1H), 1.91 (q, *J* = 11.8, 11.2 Hz, 1H), 1.83 (dd, *J* = 24.5, 13.3 Hz, 2H), 1.70 – 1.60 (m, 2H), 1.46 (s, 3H), 1.36 (s, 2H), 1.25 (d, *J* = 13.6 Hz, 1H), 1.17 (dd, *J* = 24.6, 10.0 Hz, 2H), 1.06 (dd, *J* = 7.1, 3.3 Hz, 21H), 0.60 (s, 3H).

^{13}C NMR for 46 (176 MHz, C_6D_6) δ 169.6, 160.7, 152.0, 147.8, 116.0, 114.1, 83.9, 78.5, 75.1, 74.6, 71.5, 59.2, 55.8, 51.7, 50.6, 45.0, 43.6, 40.2, 40.2, 36.5, 30.2, 28.7, 22.1, 19.9, 18.1, 18.1, 17.2, 12.2.

HRMS (ESI) **for 46**: calculated for $C_{34}H_{53}O_8Si_3^+$ [M+H]$^+$ 617.3504, found 617.3505.

$[\alpha]_D^{27}$ **for 46** = +12.04 (*c* = 0.16 in DCM).

R*f* for 46 = 0.36 (ethyl acetate in hexanes, 2:8).

IR (film, cm^{-1}) **for 46:** 3931, 3899, 3861, 3784, 3752, 3730, 3691, 3671, 3638, 3620, 3605, 3573, 3542, 3511, 3489, 3310, 3387, 3361, 3334, 3315, 3274, 3251, 3198, 3165, 3140, 3102, 3068, 3032, 2960, 2923, 2866, 2851, 2540, 2427, 2355, 2270, 2238, 2222, 2203, 2184, 2165, 2149, 2120, 2070, 2049, 2036, 2020, 2003, 1984, 1968, 1947, 1918, 1872, 1815, 1750, 1727, 1636, 1617, 1560, 1537, 1463, 1376, 1251, 1234, 1174, 1154, 1125, 1103, 1067, 1050, 1037, 996, 966, 946, 882, 838, 793, 753, 684, 658, 643, 626, 594, 579, 562, 537, 493, 478, 462, 447, 435, 416.

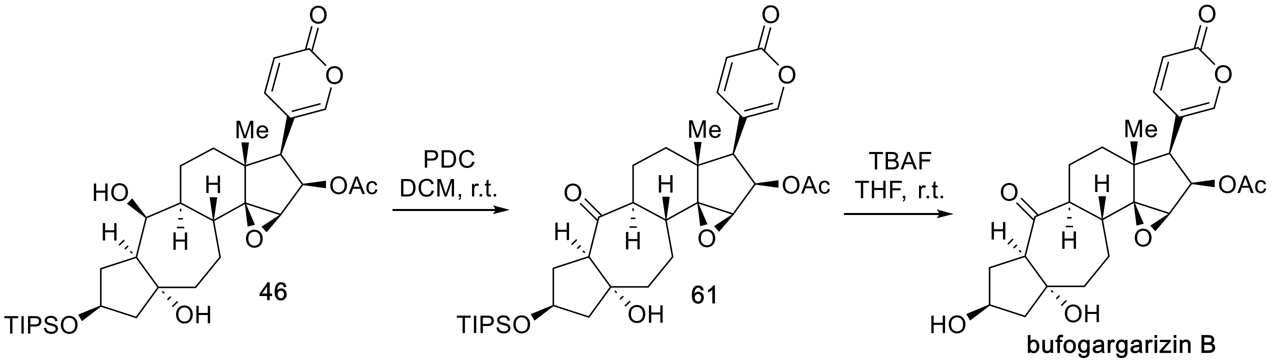

6.2 mg of **46** was dissolved in 0.6 mL (0.02 M for SM) of dry dichloromethane and placed under an atmosphere of nitrogen. 45 mg of PDC (12 equiv, 0.121 mmol) was added and the reaction was allowed to stir for 21 hours. The reaction was filtered through celite, washed with DCM, concentrated *in vacuo*, and then filtered through a pad of silica and concentrated. The crude **61** was dissolved in 0.26 mL of dry THF (0.03 M) and placed under a nitrogen atmosphere. 8 μL of TBAF (1M in THF, 1 equiv, 0.0078 mmol) was added, and the reaction mixture was stirred at room temperature for 6 hours. After this time, the reaction was still incomplete, and another 8 μL (1 equiv, 0.0078 mmol) of TBAF in 1M THF was added and allowed to stir for another 16 hours. The reaction quenched with saturated $NaHCO_3$, extracted with EtOAc (6 × 5mL), and concentrated *in vacuo*. The resulting crude mixture was subjected to column chromatography (100% DCM, then 10:90 MeOH/DCM) to produce **bufogargarizin B** in 77% yield (3.2 mg). This procedure was verified 2 more times, producing in total 14.8 mg of **bufogargarizin B** which was then characterized.

^{1}H NMR (700 MHz, CD_3OD) δ 7.99 (d, *J* = 9.9 Hz, 1H), 7.37 (s, 1H), 6.24 (d, *J* = 9.8 Hz, 1H), 5.50 (dd, *J* = 9.2, 1.6 Hz, 1H), 4.38 (ddd, *J* = 15.8, 9.4, 6.5 Hz, 1H), 3.76 (s, 1H), 3.12 (t, *J* = 8.3 Hz, 1H), 2.94 (d, *J* = 9.3 Hz, 1H), 2.74 (td, *J* = 11.9, 3.5 Hz, 1H), 2.08 – 1.99 (m, 2H), 1.96 – 1.93 (m, 3H), 1.86 (s, 3H), 1.80 (dd, *J* = 13.7, 3.1 Hz, 1H), 1.76 (td, *J* = 11.7, 2.9 Hz, 1H), 1.68 (ddd, *J* = 11.4, 8.5, 2.9 Hz, 1H), 1.62 – 1.54 (m, 2H), 1.50 (dd, *J* = 14.2, 3.5 Hz, 1H), 1.41 (dd, *J* = 12.7, 10.1 Hz, 1H), 1.19 (dd, *J* = 15.1, 11.4 Hz, 1H), 0.86 (s, 3H).

^{13}C NMR (176 MHz, CD_3OD) δ 212.7, 171.5, 164.0, 153.7, 150.7, 118.2, 114.2, 79.8, 76.4, 72.2, 71.7, 62.7, 61.0, 58.0, 51.8, 51.2, 46.5, 39.9, 39.7, 39.4, 35.6, 24.3, 22.4, 20.3, 17.5.

HRMS (ESI): calculated for $C_{25}H_{31}O_8^+$ $[M+H]^+$ 459.2013, found 459.2019.

$[\alpha]_D^{27}$ = +2.07 (*c* = 0.47 in MeOH).

$[\alpha]_D^{27}$ for isolated bufogargarizin B = +4.2 (*c* = 0.1 in MeOH), reported by Ye et al.[7]

R*f* = 0.6 (methanol in DCM, 1:9).

IR (film, cm^{-1}): 3403, 2961, 2920, 2851, 2203, 2192, 2098, 1871, 1795, 1737, 1722, 1706, 1602, 1557, 1532, 1467, 1451, 1376, 1317, 1260, 1234, 1174, 1157, 1102, 1067, 1024, 1015, 909, 891, 840, 796, 753, 723, 700, 682, 641, 580, 557, 520, 495, 475, 454, 442, 428, 418.

c. Comparison of the ^{1}H NMR data of our synthetic bufogargarizin B with the authentic sample and the previously reported synthetic material

The ^{1}H NMR spectrum of synthetic **bufogargarizin B** (recorded in CD_3OD) closely matches that of the isolated natural product[7] and the synthetic sample reported by the Li research group[1]. Chemical shifts, multiplicities, and coupling constants for all key protons are consistent within ±0.02 ppm, confirming that our synthetic material is structurally identical to both the natural isolated and the previously reported synthetic compound.

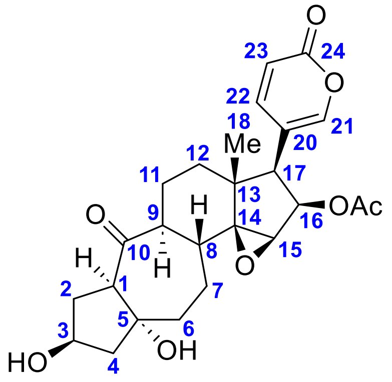

bufogargarizin B

Table S11. Comparison of ^{1}H NMR data.

Position	Synthetic[a] (Syn.) 700 MHz	Isolated[b] (Iso.) 400 MHz	Li's work[a] (Li) 500 MHz	Error (Iso.-Syn.)	Error (Li-Syn.)
**1α**	3.12 (t, 8.3)	3.10 (dd, 8.2, 8.2)	3.11 (dd, 8.3, 8.3)	-0.02	-0.01
**2α**	2.03[c]	2.04[c]	2.03	0.01	0
**2β**	2.03[c]	2.04[c]	2.03	0.01	0
**3α**	4.38 (ddd, 15.8, 9.4, 6.5)	4.38, m	4.38, m	0	0
**4α**	1.94[c]	1.94[c]	1.94	0	0
**4β**	1.41 (dd, 12.7, 10.1)	1.41 (dd, 12, 10)	1.41 (dd, 12.7, 10.2)	0	0
**6α**	1.95[c]	1.95[c]	1.95	0	0
**6β**	1.19 (dd, 15.1, 11.4)	1.20 (dd, 14.1, 11.2)	1.19 (dd, 15, 11.2)	0.01	0
**7α**	1.58[c]	1.58[c]	1.58[c]	0	0
**7β**	1.68 (ddd, 11.4, 8.5, 2.9)	1.67[c]	1.67[c]	-0.01	-0.01
**8β**	1.76 (td, 11.7, 2.9)	1.74[c]	1.74[c]	-0.02	-0.02
**9α**	2.74 (td, 11.9, 3.5)	2.73 (ddd, 11.9, 11.3, 3.7)	2.74 (ddd, 11.9, 11.9, 3.6)	-0.01	0
**11α**	1.96[c]	1.97[c]	1.97[c]	0.01	0.01
**11β**	1.50 (dd, 14.2, 3.5)	1.48[c]	1.48[c]	-0.02	-0.02

Position	Synthetic[a] (Syn.) 700 MHz	Isolated[b] (Iso.) 400 MHz	Li's work[a] (Li) 500 MHz	Error (Iso.-Syn.)	Error (Li-Syn.)
**12α**	1.59[c]	1.59[c]	1.59[c]	0	0
**12β**	1.80 (dd, 13.7, 3.1)	1.79[c]	1.79[c]	-0.01	-0.01
**15**	3.76 (s)	3.76, (br s)	3.76 (br s)	0	0
**16**	5.50 (dd, 9.2, 1.6)	5.49 (d, 8.6)	5.50 (d, 9.3, 1.1)	-0.01	0
**17**	2.94 (d, 9.3)	2.94 (d, 8.6)	2.95	0	0.01
**18**	0.86 (s)	0.86 (s)	0.86 (s)	0	0
**22**	7.37 (br s)	7.38 (d, 1.0)	7.38 (s)	0.01	0.01
**23**	7.99 (d, 9.9)	8.00 (dd, 9.0, 1.0)	8.00 (d, 8.3)	0.01	0.01
**24**	6.24 (d, 9.8)	6.24 (d, 8.0)	6.24 (d, 9.7)	0	0
**CH_3 (Ac)**	1.86 (s)	1.86 (s)	1.86 (s)	0	0

[a]Chemical shifts referenced to CD_3OD at 3.30 ppm.
[b]Chemical shift of CD_3OD not reported. [c]Overlapping signals.

d. Comparison of the ^{13}C NMR data of our synthetic bufogargarizin B with the authentic sample and the previously reported synthetic material

The ^{13}C NMR spectrum of synthetic **bufogargarizin B** (recorded in CD_3OD) closely matches that of the isolated natural product[7] and the synthetic sample reported by the Li research group[1]. Chemical shifts for all key carbon resonances are consistent within ±0.1 ppm, confirming that our synthetic material is structurally identical to both the natural isolate and the previously reported synthetic compound.

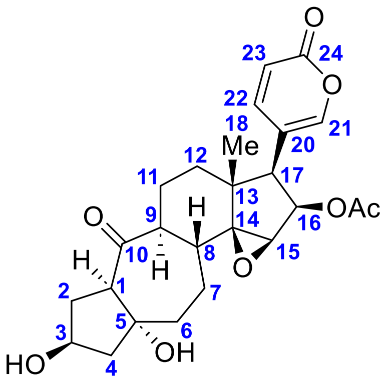

bufogargarizin B

Table S12. Comparison of ^{13}C NMR data.

Position	Synthetic[a] (Syn.) 176 MHz	Isolated[b] (Iso.) 100 MHz	Li's work[a] (Li) 125 MHz	Error (Iso.-Syn.)	Error (Li-Syn.)
**1**	62.7	62.8	62.7	0.1	0
**2**	35.6	35.6	35.7	0	0.1

Position	Synthetic[a] (Syn.) 176 MHz	Isolated[b] (Iso.) 100 MHz	Li's work[a] (Li) 125 MHz	Error (Iso.-Syn.)	Error (Li-Syn.)
**3**	71.7	71.7	71.8	0	0.1
**4**	51.8	51.9	51.9	0.1	0.1
**5**	79.8	79.9	79.9	0.1	0.1
**6**	39.7	39.7	39.7	0	0
**7**	22.4	22.4	22.5	0	0.1
**8**	39.9	39.9	40.0	0	0.1
**9**	58.1	58.1	58.1	0	0
**10**	212.7	212.7	212.7	0	0
**11**	24.3	24.3	24.3	0	0
**12**	39.4	39.4	39.5	0	0.1
**13**	46.5	46.5	46.5	0	0
**14**	72.2	72.3	72.3	0.1	0.1
**15**	61.0	61.0	61.1	0	0.1
**16**	76.4	76.4	76.4	0	0
**17**	51.2	51.2	51.3	0	0.1
**18**	17.5	17.5	17.5	0	0
**20**	118.2	118.1	118.2	-0.1	0
**21**	153.7	153.7	153.7	0	0
**22**	150.7	150.7	150.8	0	0.1
**23**	114.2	114.1	114.2	-0.1	0
**24**	164.0	164.0	164.0	0	0
**CH_3 (Ac)**	20.3	20.3	20.4	0	0.1
**C=O (Ac)**	171.6	171.6	171.6	0	0

[a]Chemical shifts referenced to CD_3OD at 49 ppm. [b]Chemical shift of CD_3OD not reported.

v. Synthesis of bufogargarizin A

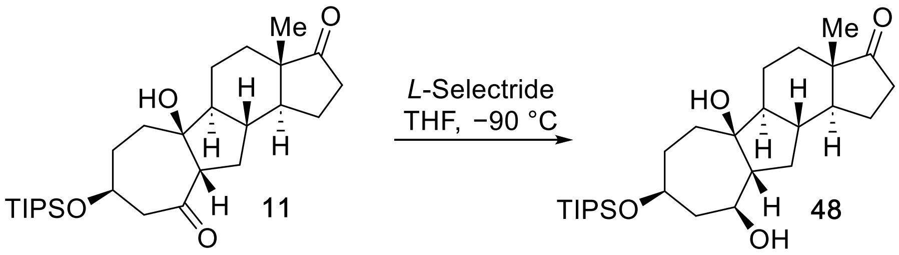

To a solution of compound **11** (70.1 mg, 0.151 mmol, 1.00 equiv) in anhydrous THF (7.6 mL) at −90 °C was added dropwise a precooled solution of *L*-selectride (1.0M in THF, 0.16 mL, 0.168 mmol, 1.05 equiv) under an argon atmosphere. The reaction mixture was stirred at -−90 °C until complete consumption of the starting material was confirmed by TLC. The reaction was quenched by slow addition of cold methanol (1.0 mL) and allowed to warm to room temperature. A solution of 30% H_2O_2 (0.16 mL) and 1.0 M NaOH (0.16 mL) was then added, and the reaction mixture was stirred at room temperature for 1.5 h. The mixture was then diluted with EtOAc (10 mL) and washed with saturated aqueous NH_4Cl (3 × 5 mL), followed by washes with a 1:1 mixture of H_2O/brine (2 × 5 mL). The organic layer was dried over anhydrous Na_2SO_4, filtered, and concentrated under reduced pressure. The crude residue was purified by flash column chromatography on silica gel (1:4, EtOAc:hexanes) to afford **48** as a white solid (54.1 mg, 77%).

^{1}H NMR (500 MHz, $CDCl_3$) δ 4.10 (td, *J* = 8.7, 4.3 Hz, 1H + bs, 1H), 2.45 (dd, *J* = 19.2, 8.7 Hz, 1H), 2.28 – 2.13 (m, 2H), 2.08 (dd, *J* = 19.1, 9.2 Hz, 1H), 2.02 – 1.74 (m, 7H), 1.71 – 1.60 1.67 (m, 3H), 1.56 – 1.48 (m, 1H), 1.46 – 1.36 (m, 2H), 1.28 – 1.13 (m, 3H), 1.11 – 1.01 (m, 21H), 0.94 – 0.85 (m, 1H), 0.90 (s, 3H).
^{13}C NMR (126 MHz, $CDCl_3$) δ 220.8, 81.2, 69.3, 57.4, 56.4, 51.5, 49.1, 44.8, 38.5, 36.0, 33.0, 32.2, 31.7, 31.2, 29.8, 22.6, 19.2, 18.3, 18.3, 14.2, 12.5.
HRMS (ESI): calculated for $C_{27}H_{49}O_4Si^+$ [M+H]$^+$ 465.3395, found 465.3394.
$[\alpha]_D^{26}$ = +28.1 (*c* = 0.515 in DCM).
R*f* = 0.38 (ethyl acetate in hexanes, 4:6).
IR (film): 3434, 2946, 2928, 2865, 1726, 1467, 1386, 1374, 1277, 1244, 1081, 1061, 1034, 1008, 916, 883, 821, 716, 677, 661.

Note: Due to the conformational flexibility of the seven-membered ring, the ^{13}C NMR signals corresponding to that ring are relatively weak and broadened.

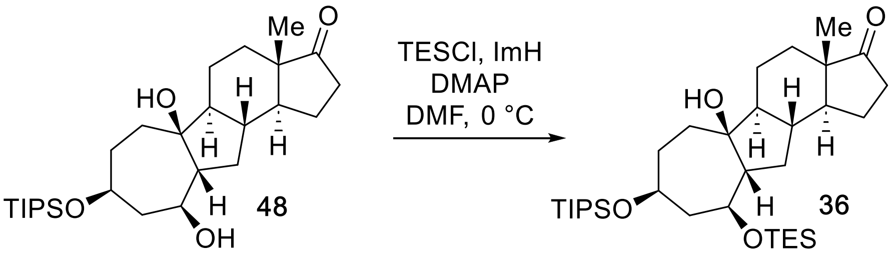

To a stirred, ice-cooled solution of **48** (108.7 mg, 0.234 mmol, 1.00 equiv) and 4-dimethylaminopyridine (DMAP, 6.9 mg, 0.056 mmol, 0.24 equiv) in anhydrous DMF (10 mL) under an argon atmosphere was added a solution of imidazole (57.3 mg, 0.842 mmol, 3.60 equiv) in DMF (1.7 mL). After stirring for 10 min at 0 °C, triethylsilyl chloride (TESCl, 94.2 μL, 84.6 mg, 0.561 mmol, 2.40 equiv) was added dropwise. The reaction was monitored by TLC and allowed to warm to room temperature upon completion. The reaction was quenched with water (10 mL), and the mixture was extracted with Et_2O (4 × 10 mL). The combined organic extracts were washed with water (3 × 10 mL) and brine (1 × 15 mL), then dried over anhydrous Na_2SO_4, filtered, and concentrated under reduced pressure. The crude product was purified by flash column chromatography (1:9, EtOAc:hexanes) to afford the TES-protected ketone **36** as a colorless foam (123.4 mg, 91%).

^{1}H NMR at 55 °C (600 MHz, C_6D_6) δ 4.19 (tt, *J* = 9.8, 3.9 Hz, 1H), 3.91 (bs, 1H), 2.30 (dt, *J* = 12.3, 5.2 Hz, 1H), 2.16 (dd, *J* = 19.0, 8.8 Hz, 1H), 2.02 (t, *J* = 9.2 Hz, 1H), 1.98 – 1.89 (m, 2H), 1.88 –1.73 (m, 4H), 1.73 – 1.58 (m, 4H), 1.34 – 1.22 (m, 6H), 1.22 – 1.08 (m, 22H), 1.00 (t, *J* = 8.0 Hz, 9H), 0.81 (td, *J* = 12.9, 8.8 Hz, 1H), 0.73 (s, 3H), 0.63 (q, *J* = 7.9 Hz, 6H).
^{13}C NMR at 55 °C (151 MHz, C_6D_6) δ 217.2, 80.9, 71.1, 70.8, 58.3, 57.4, 52.1, 49.0, 46.5, 38.9, 35.7, 33.6, 32.5, 32.3, 32.1, 22.8, 19.5, 18.6, 18.5, 14.2, 13.1, 7.3, 5.7.
HRMS (ESI): calculated for $C_{33}H_{62}O_4Si_2Na^+$ $[M+Na]^+$ 601.4079, found 601.4075.
$[\alpha]_D^{25}$ = +17.3 (*c* = 0.503 in DCM).
R*f* = 0.50 (ethyl acetate in hexanes, 3:7).
IR (film, cm^{-1}): 3470, 2942, 2866, 1741, 1463, 1380, 1240, 1083, 1070, 1036, 1004, 940, 927, 883, 818, 740, 726, 680.

Note: Due to the conformational flexibility of the seven-membered ring in **36**, the ^{13}C NMR signals corresponding to that ring are relatively weak and broadened. Spectra were recorded at 55 °C to mitigate the effects of dynamic conformational exchange.

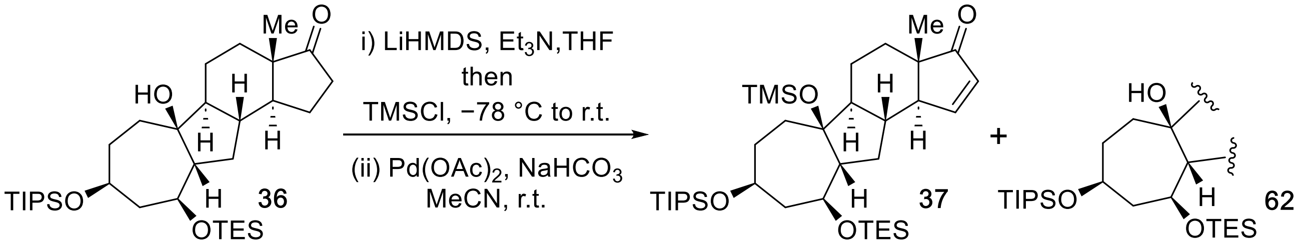

To a stirred solution of compound **36** (123.4 mg, 0.213 mmol, 1.0 equiv) in anhydrous THF (8.52 mL) at -78°C was added triethylamine (Et_3N, 0.30 mL, 2.131 mmol, 10 equiv) under an argon atmosphere, followed by addition of a precooled solution of lithium bis(trimethylsilyl)amide (LiHMDS, 178.3 mg, 1.066 mmol, 5.0 equiv) in THF (2.13 mL). The reaction mixture was stirred at −78 °C for 40 min, after which freshly distilled trimethylsilyl chloride (TMSCl, 0.27 mL, 2.131 mmol, 10 equiv) was added. The reaction was maintained at −78 °C for 1 h and then allowed to warm gradually to room temperature, followed by stirring for an additional 1 h. The reaction was quenched with saturated aqueous $NaHCO_3$ (10 mL) and extracted with EtOAc (4 × 10 mL). The combined organic layers were dried over anhydrous Na_2SO_4, filtered, and concentrated under reduced pressure. The resulting residue was dissolved in dry MeCN (10.65 mL), and $Pd(OAc)_2$ (57.4 mg, 0.256 mmol, 1.2 equiv) and $NaHCO_3$ (35.8 mg, 0.426 mmol, 2.0 equiv) were added under an argon atmosphere. The mixture was stirred at room temperature overnight. Upon completion, the reaction was filtered through a short pad of Celite and washed with EtOAc. The filtrate was concentrated under reduced pressure, and the residue was purified by flash column chromatography (Et_3N-neutralized silica gel; 1:19, EtOAc:hexanes) to afford **37** (109.7 mg, 79%) as a light-yellow oil. In addition, a minor byproduct, the C10-hydroxylated enone **62**, was isolated in 7.4 mg.

^{1}H NMR at 55 °C (700 MHz, C_6D_6) δ 6.99 (dd, *J* = 6.0, 1.8 Hz, 1H), 5.87 (dd, *J* = 6.0, 3.1 Hz, 1H), 4.40 (s, 1H), 4.08 (dt, *J* = 7.6, 4.2 Hz, 1H), 2.45 (s, 1H), 2.19 (d, *J* = 11.6 Hz, 2H), 2.06 – 1.95 (m, 4H), 1.82 – 1.69 (m, 2H), 1.69 – 1.58 (m, 2H), 1.46 (td, *J* = 12.8, 4.7 Hz, 1H), 1.38 (dd, *J* = 13.2, 5.7 Hz, 1H), 1.20 – 0.99 (m, 23H), 1.08 (s, 3H), 1.04 (t, J = 8.0 Hz, 9H), 0.82 (td, *J* = 11.6, 4.4 Hz, 1H), 0.69 (q, *J* = 7.9 Hz, 6H), 0.22 (s, 9H).
^{13}C NMR at 55 °C (176 MHz, C_6D_6) δ 210.2, 157.9, 132.3, 85.0, 69.4, 59.0, 58.5, 57.2, 52.7, 44.2, 36.1, 33.4, 30.8, 30.6, 29.4, 21.2, 19.7, 18.6, 18.5, 13.1, 7.3, 5.7, 2.9.
HRMS (ESI): calculated for $C_{36}H_{69}O_4Si_3^+$ $[M+H]^+$ 649.4498, found 649.4498.
$[\alpha]_D^{25}$ = -24.2 (*c* = 0.50 in DCM).
R*f* = 0.60 (ethyl acetate in hexanes, 2:8).
IR (film, cm^{-1}): 2940, 2866, 1716, 1459, 1249, 1063, 1004, 920, 882, 835, 815, 743, 678.

Note: Due to the conformational flexibility of the seven-membered ring in **37**, the ^{13}C NMR signals corresponding to that ring are relatively weak and broadened. Spectra were recorded at 55 °C to mitigate the effects of dynamic conformational exchange.

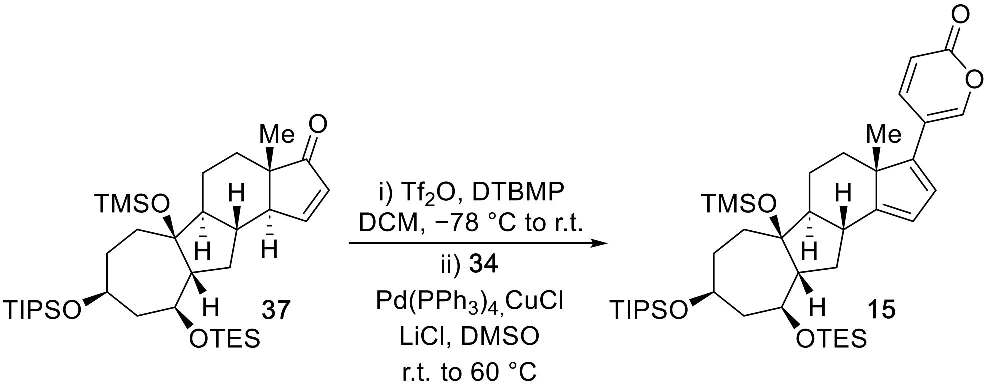

To a stirred solution of enone **37** (71.8 mg, 0.11 mmol, 1.0 equiv) and 2,6-di-tert-butyl-4-methylpyridine (DTBMP, 227.1 mg, 1.10 mmol, 10 equiv) in anhydrous DCM (5.5 mL) at −78 °C under an argon atmosphere was added trifluoromethanesulfonic anhydride (Tf_2O, 37.2 µL, 0.22 mmol, 2.0 equiv). The reaction mixture was stirred at −78 °C for 5 min, then allowed to warm to room temperature and stirred for an additional 15 min. The reaction was quenched with saturated aqueous $NaHCO_3$ (6 mL) and extracted with DCM (2 × 5 mL). The combined organic layers were dried over anhydrous Na_2SO_4, filtered, and concentrated under reduced pressure. The resulting crude triflate **38** was used directly in the subsequent coupling reaction without further purification. To a flame-dried vial containing the crude triflate **38** were added $Pd(PPh_3)_4$ (12.8 mg, 0.011 mmol, 0.1 equiv), CuCl (54.7 mg, 0.55 mmol, 5.0 equiv), and LiCl (46.9 mg, 1.10 mmol, 10 equiv) under an inert atmosphere in a glovebox. The vial was sealed with a septum, removed from the glovebox, and anhydrous DMSO (4.07 mL) was added via syringe. A solution of **34** (143.2 mg, 0.55 mmol, 5.0 equiv) in DMSO (1.43 mL, 100 mg/mL) was then added. The resulting suspension was sparged with nitrogen at room temperature for 1 h, then heated to 60 °C and stirred for 1 h under a nitrogen atmosphere. After cooling to room temperature, the mixture was quenched with saturated aqueous $NaHCO_3$ (6 mL) and extracted with EtOAc (4 × 5 mL). The combined organic layers were washed sequentially with saturated aqueous NH_4Cl, saturated aqueous $NaHCO_3$, and brine, then dried over anhydrous Na_2SO_4, filtered, and concentrated under reduced pressure. The crude product was purified by flash column chromatography (Et_3N-neutralized silica gel; 1:19, EtOAc:hexanes) to afford the diene **15** (59.5 mg, 74%) as a white solid.

^{1}H NMR at 55 °C (700 MHz, C_6D_6) δ 7.16 (s, 1H, overlapped with C_6D_6), 6.88 (ddd, *J* = 9.8, 2.8, 1.0 Hz, 1H), 6.14 (d, *J* = 2.3 Hz, 1H), 5.98 (dt, *J* = 9.8, 1.0 Hz, 1H), 5.95 (d, *J* = 2.1 Hz, 1H), 4.22 (s, 1H), 4.10 (s, 1H), 2.76 – 2.67 (m, 1H), 2.48 (bs, 1H), 2.25 – 2.08 (m, 3H), 1.82 (bs, 1H), 1.77 – 1.68 (m, 4H), 1.56 (qd, *J* = 13.0, 3.5 Hz, 1H), 1.39 – 1.34 (m, 1H), 1.20 – 1.11 (m, 22H), 1.04 (t, *J* = 8.0 Hz, 9H), 0.97 (dd, 1H), 0.95 (s, 3H), 0.91 (dd, *J* = 13.1, 4.2 Hz, 1H), 0.69 (q, *J* = 8.0 Hz, 6H), 0.30 (s, 9H).
^{13}C NMR at 55 °C (176 MHz, C_6D_6) δ 163.8, 160.0, 147.4, 146.0, 142.8, 126.9, 116.9, 116.8, 115.2, 86.1, 69.9, 61.4, 58.3, 54.1, 45.4, 39.8, 37.9, 33.7, 31.5, 30.2, 30.0, 21.0, 20.6, 18.6, 18.5, 13.1, 7.4, 5.7, 3.0.
HRMS (ESI): calculated for $C_{41}H_{71}O_5Si_3^+$ $[M+H]^+$ 727.4604, found 727.4600.

$[\alpha]_D^{24}$ = +166.9 (c = 0.50 in DCM).
R*f* = 0.52 (ethyl acetate in hexanes, 2:8).
IR (film, cm^{-1}): 2943, 2866, 1732, 1460, 1249, 1080, 1007, 882, 834, 740, 678.

Note: Due to the conformational flexibility of the seven-membered ring in **15**, the ^{13}C NMR signals corresponding to that ring are relatively weak and broadened. Spectra were recorded at 55 °C to mitigate the effects of dynamic conformational exchange.

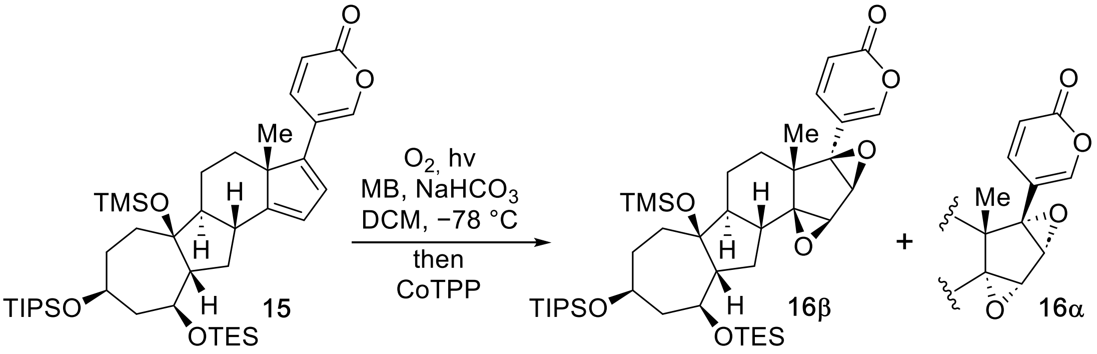

To a flame-dried 18×100 mm test tube equipped with a magnetic stir bar were added sodium bicarbonate ($NaHCO_3$, 20.1 mg, 0.240 mmol, 2.0 equiv), methylene blue (MB, tip of spatula), and capped with a septum. The septum was sealed using Parafilm® and electrical tape to ensure an airtight environment. A solution of the diene **15** (87.1 mg, 0.120 mmol, 1.0 equiv) in DCM (3.0 mL) was added via syringe under an O_2 atmosphere. The headspace above the reaction mixture was wrapped with aluminum foil to minimize light exposure to the gas phase. The reaction vessel was cooled to −78 °C using a dry ice/acetone bath, ensuring the entire solution was submerged. A 400 W floodlamp was positioned approximately 11 cm from the reaction vessel, and the mixture was irradiated under these conditions. After completion of the reaction (~30 min), the O_2 balloon was removed, and the mixture was subjected to vacuum on a Schlenk line at −78 °C to remove residual oxygen. The vacuum was released, and the test tube was backfilled with N_2.To the reaction mixture, a precooled solution of cobalt(II) tetraphenylporphyrin (CoTPP, 8.0 mg, 0.012 mmol, 0.10 equiv) in DCM (0.5 mL) was added via syringe at −78 °C under nitrogen. The reaction was stirred for 1 h at this temperature. The mixture was then concentrated under reduced pressure, and the crude residue was purified by flash column chromatography on silica gel (1:4, EtOAc:hexanes) to afford a mixture of the desired product **16β** and its diastereomer **16α**, resulting from opposite-face singlet oxygen addition. The combined yield of both diastereomers was 51.0 mg (56%), with a diastereomeric ratio (d.r.) of 1:2 (β:α), as determined by ^{1}H NMR analysis. The diastereomers were further separated by exhaustive flash column chromatography (4:1, MTBE:hexanes).

^{1}H NMR at 55 °C for 16β (700 MHz, C_6D_6) δ 6.76 (dd, J = 2.8, 1.6 Hz, 1H), 6.64 (dt, J = 9.6, 1.9 Hz, 1H), 5.84 (d, J = 9.6 Hz, 1H), 4.18 (dq, J = 8.9, 4.3 Hz, 1H), 3.37 (s, 1H), 2.93 (s, 1H), 2.33 (m, 1H), 2.18 (m, 2H), 1.96 – 1.87 (m, 2H), 1.86 – 1.78 (m, 1H), 1.78 – 1.71 (m, 2H), 1.32 – 1.25 (m, 2H), 1.20 – 1.15 (m, 24H), 1.15 – 1.11 (m, 3H),

1.10 – 1.06 (m, 1H), 1.06 – 1.04 (m, 1H), 1.02 (td, *J* =8.0, 1.4 Hz, 9H), 0.94 – 0.89 (m, 1H), 0.66 (tt, *J* = 12.4, 5.3 Hz, 6H), 0.24 (s, 9H).

^{13}C NMR at 55 °C for 16β (176 MHz, C_6D_6) δ 159.6, 151.3, 142.8, 116.1, 114.6, 85.9, 80.2, 75.0, 70.0, 57.8, 56.9, 56.3, 54.3, 45.6, 41.2, 38.2, 37.0, 33.8, 31.2, 30.2, 29.0, 20.5, 18.5, 18.5, 15.4, 13.1, 7.4, 5.7, 2.9.

HRMS (ESI) **for 16β**: calculated for $C_{41}H_{71}O_7Si_3^+$ $[M+H]^+$ 759.4502, found 759.4502.

$[\alpha]_D^{25}$ **for 16β** = +15.2 (*c* = 0.547 in DCM).

R*f* for 16β = 0.71 (hexanes in MTBE, 2:8).

IR (film, cm^{-1}) **for 16β**: 2944, 2867, 2361, 1751, 1462, 1250, 1087, 892, 837, 668.

^{1}H NMR at 55 °C for 16α (700 MHz, C_6D_6) δ 6.73 (dd, *J* = 2.7, 1.3 Hz, 1H), 6.61 (dd, *J* = 9.6, 2.6 Hz, 1H), 5.87 (dd, *J* = 9.6, 1.2 Hz, 1H), 4.04 (s, 1H), 3.18 (s, 1H), 2.84 (d, *J* = 1.3 Hz, 1H), 2.54 (td, *J* = 11.2, 6.2 Hz, 1H), 2.46 (bs, 1H), 2.32 – 2.06 (m, 2H), 1.91 (td, *J* = 12.5, 5.0 Hz, 1H), 1.79 – 1.68 (m, 3H), 1.60 – 1.44 (m, 6H), 1.37 – 1.30 (m, 3H), 1.20 – 1.14 (m, 21H), 1.14 – 1.10 (m, 3H), 1.09 (t, *J* = 8.0 Hz, 9H), 0.76 (qd, *J* = 7.9, 3.6 Hz, 6H), 0.57 (s, 3H), 0.28 (s, 9H).

^{13}C NMR at 55 °C for 16α (176 MHz, C_6D_6) δ 159.7, 151.0, 142.9, 116.3, 115.4, 85.2, 79.2, 71.9, 69.4, 65.9, 58.4, 56.2, 55.7, 43.9, 41.9, 37.0, 33.3, 30.8, 30.2, 28.0, 25.0, 20.3, 19.7, 18.6, 18.5, 13.1, 7.4, 5.7, 2.9.

HRMS (ESI) **for 16α**: calculated for $C_{41}H_{71}O_7Si_3^+$ $[M+H]^+$ 759.4502, found 759.4502.

R*f* for 16α = 0.67 (hexanes in MTBE, 2:8).

Note: Due to the conformational flexibility of the seven-membered ring in **16α** and **16β**, the ^{13}C NMR signals corresponding to that ring are relatively weak and broadened. Spectra were recorded at 55 °C to mitigate the effects of dynamic conformational exchange.

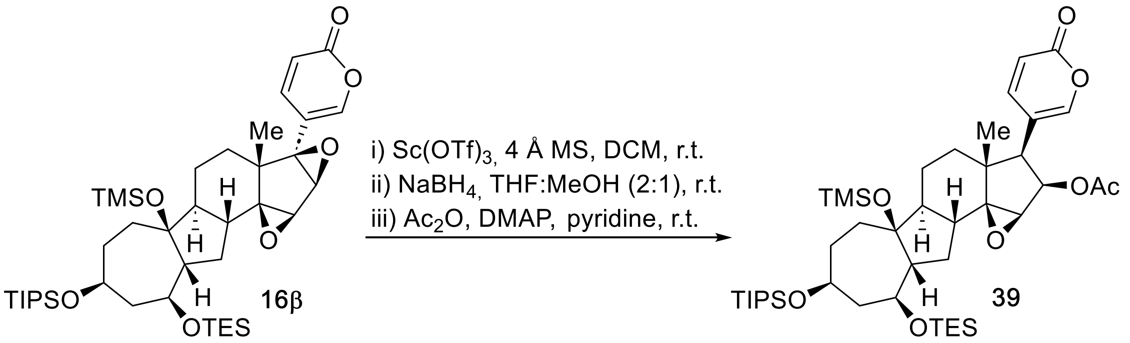

To a flame-dried 2-dram vial equipped with a magnetic stir bar and 4 Å molecular sieves was added a solution of compound **16β** (15 mg, 0.020 mmol, 1.0 equiv) in anhydrous DCM (1.0 mL) under a nitrogen atmosphere. After stirring at room temperature for 10 min, a solution of scandium trifluoromethanesulfonate ($Sc(OTf)_3$, 0.97 mg, 0.002 mmol, 0.1 equiv) in MeCN (9.7 µL, 100 mg/mL) was added. The reaction mixture was stirred for 10–15 min and then quenched with saturated aqueous $NaHCO_3$ (1.0 mL), and the mixture was extracted with DCM (3 × 0.5 mL). The combined organic layers were dried over anhydrous Na_2SO_4, filtered, and concentrated under reduced pressure. The crude product (**i**) was carried forward without purification.

The crude product (**i**) was dissolved in a mixture of THF (0.8 mL) and MeOH (0.4 mL), and $NaBH_4$ (0.82 mg, 0.022 mmol, 1.1 equiv) was then added at room temperature. After stirring for 15 min, the reaction was quenched with saturated aqueous NH_4Cl (1.5 mL) and extracted with EtOAc (4 × 1.0 mL). The combined organic layers were washed with brine, dried over anhydrous Na_2SO_4, filtered, and concentrated in vacuo. The resulting residue (**ii**) was used directly in the next step.

The crude product (**ii**) was dissolved in pyridine (0.4 mL) under nitrogen, followed by addition of DMAP (0.5 mg, 0.004 mmol, 0.2 equiv). Acetic anhydride (Ac_2O, 6.5 μL, 7.06 mg, 0.07 mmol, 3.5 equiv) was then added. The reaction was stirred at room temperature for 1 h, then quenched with saturated aqueous NH_4Cl (1.0 mL) and extracted with EtOAc (4 × 1.0 mL). The combined organic layers were washed with brine, dried over anhydrous Na_2SO_4, filtered, and concentrated under reduced pressure. The crude product was purified by flash column chromatography (1:9, EtOAc:hexanes) to afford the acetate **39** (11.5 mg, 72% over 3 steps) as a white solid.

^{1}H NMR at 55 °C (700 MHz, C_6D_6) δ 7.82 (s, 1H), 6.75 (s, 1H), 6.02 (d, *J* = 9.8 Hz, 1H), 5.36 (d, *J* = 9.3 Hz, 1H), 4.15 (dt, *J* = 9.1, 5.1 Hz, 1H), 4.11 (bs, 1H), 3.68 (s, 1H), 2.82 (q, *J* = 7.3 Hz, 1H), 2.51 (d, *J* = 9.3 Hz, 1H), 2.41 (td, *J* = 12.0, 6.1 Hz, 1H), 2.30 (s, 1H), 2.14 (s, 2H), 1.96 (dt, *J* = 13.8, 7.4 Hz, 1H), 1.86 (q, *J* = 10.6 Hz, 1H), 1.77 – 1.68 (m, 3H), 1.53 – 1.48 (m, 1H), 1.48 (s, 3H), 1.46 – 1.42 (m, 1H), 1.36 – 1.30 (m, 1H), 1.23 (td, *J* = 12.3, 3.5 Hz, 1H), 1.20 – 1.11 (m, 22H), 1.03 (t, *J* = 8.0 Hz, 9H), 0.72 (s, 3H), 0.65 (qd, *J* = 7.8, 2.6 Hz, 6H), 0.24 (s, 9H).
^{13}C NMR at 55 °C (176 MHz, C_6D_6) δ 169.6, 160.5, 152.0, 147.7, 115.8, 114.2, 86.0, 75.2, 72.9, 70.0, 59.7, 58.2, 57.9, 57.0, 50.6, 46.1, 45.4, 41.4, 36.5, 33.8, 31.2, 29.4, 21.0, 19.9, 18.5, 18.5, 17.5, 13.1, 7.3, 5.8, 2.9.
HRMS (ESI): calculated for $C_{43}H_{75}O_8Si_3^+$ $[M+H]^+$ 803.4764, found 803.4770.
$[\alpha]_D^{25}$ = -0.9 (*c* = 0.50 in DCM).
R_f = 0.48 (ethyl acetate in hexanes, 3:7).
IR (film, cm^{-1}): 2946, 2868, 1748, 1539, 1458, 1374, 1249, 1087, 889, 837, 747, 683, 605.

Note: Due to the conformational flexibility of the seven-membered ring in **39**, the ^{13}C NMR signals corresponding to that ring are relatively weak and broadened. Spectra were recorded at 55 °C to mitigate the effects of dynamic conformational exchange.

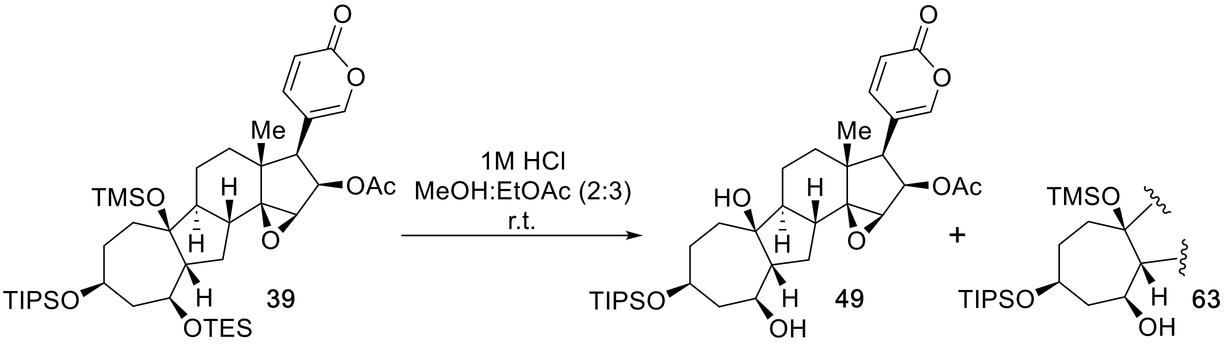

To a solution of the acetate **39** (11 mg, 0.0137 mmol, 1.0 equiv) in MeOH/EtOAc (2:3, 0.7 mL) was added 1 drop of 1M aqueous HCl. The reaction mixture was stirred at room temperature for 10–15 min and then quenched by addition of saturated aqueous $NaHCO_3$ to adjust the pH to neutral. The aqueous layer was extracted with EtOAc (3 × 1.0 mL), and the combined organic extracts were washed with brine, dried over anhydrous Na_2SO_4, filtered, and concentrated under reduced pressure. Purification of the crude material by flash column chromatography (3:7 to 1:1, EtOAc:hexanes) afforded **49** (7.3 mg, 86%) as a white solid. Additionally, a minor product **63** (0.5 mg), corresponding to the TMS-protected alcohol at the C10 position, was also detected.

^{1}H NMR (500 MHz, C_6D_6) δ 7.84 (s, 1H), 6.68 (s, 1H), 6.01 (d, *J* = 9.8 Hz, 1H), 5.11 (d, *J* = 9.3 Hz, 1H), 4.21 (tt, *J* = 9.9, 4.2 Hz, 1H), 3.49 (s, 1H), 3.41 (bs, 1H), 2.43 (d, *J* = 9.3 Hz, 1H), 2.32 (td, *J* = 12.0, 6.1 Hz, 1H), 2.21 (bs, 1H), 1.96 – 1.87 (m, 2H), 1.87 – 1.80 (m, 1H), 1.80 – 1.72 (m, 2H), 1.61 – 1.53 (m, 2H), 1.47 (s, 3H), 1.34 – 1.29 (m, 2H), 1.23 – 1.15 (m, 23H), 1.12 – 1.05 (m, 2H), 0.98 – 0.92 (m, 2H), 0.63 (s, 3H).
^{13}C NMR (176 MHz, C_6D_6) δ 169.7, 160.9, 151.9, 148.0, 115.9, 114.1, 81.2, 75.2, 72.7, 70.5, 69.8, 59.3, 57.5, 56.0, 50.3, 46.3, 45.9, 41.1, 36.5, 33.7, 32.7, 30.1, 20.5, 19.9, 18.5, 18.5, 17.5, 12.9.
HRMS (ESI): calculated for $C_{34}H_{53}O_8Si^+$ [M+H]$^+$ 617.3504, found 617.3497.
HRMS (ESI): calculated for $C_{34}H_{52}O_8SiCl$ [M+Cl]$^-$ 651.3125, found 651.3126.
$[\alpha]_D^{25}$ = -11.3 (*c* = 0.50 in DCM).
R*f* = 0.39 (ethyl acetate in hexanes, 1:1).
IR (film, cm^{-1}): 2928, 2865, 1722, 1711, 1380, 1240, 1064, 884.

Note: Due to the conformational flexibility of the seven-membered ring, the ^{13}C NMR signals corresponding to that ring are relatively weak and broadened.

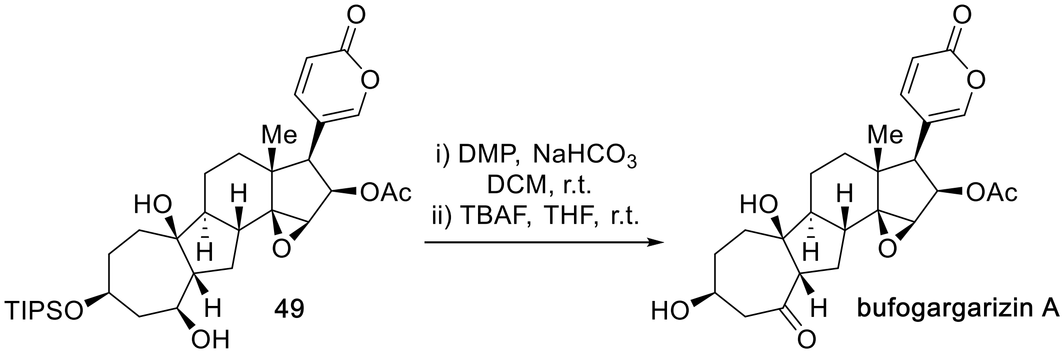

(i) To a stirred solution of **49** (6.8 mg, 0.011 mmol, 1.0 equiv) and $NaHCO_3$ (2.8 mg, 0.033 mmol, 3.0 equiv) in DCM (0.98 mL) under a nitrogen atmosphere was added a solution of Dess-Martin periodinane (DMP, 6.1 mg, 0.014 mmol, 1.3 equiv) in DCM (0.12 mL) at room temperature. The reaction was stirred for 1 h, then quenched by the addition of saturated aqueous $NaHCO_3$ (0.75 mL) and saturated aqueous $Na_2S_2O_3$ (0.75 mL). The biphasic mixture was stirred for an additional 1 h, and the layers were separated. The aqueous phase was extracted with DCM (4 × 0.5 mL), and the combined organic layers were washed with brine, dried over anhydrous Na_2SO_4, filtered, and concentrated under reduced pressure. The crude residue was used directly in the next step without purification.

(ii) The crude oxidized intermediate (**i**) was dissolved in anhydrous THF (0.55 mL) under a nitrogen atmosphere. A solution of tetrabutylammonium fluoride (TBAF, 2.88 mg, 0.011 mmol, 1.0 equiv) in THF (11 µL, 1 M) was then added, and the reaction was stirred at room temperature for 1 h. The reaction was quenched with saturated aqueous NH_4Cl (1.0 mL) and extracted with EtOAc (6 × 0.5 mL). The combined organic layers were washed with a 1:1 mixture of water/brine (4 × 0.5 mL), dried over anhydrous Na_2SO_4, filtered, and concentrated under reduced pressure. Purification by flash column chromatography (100% DCM, then 5:95 MeOH/DCM) afforded **bufogargarizin A** as a white solid (3.2 mg, 63% yield over 2 steps).

^{1}H NMR (600 MHz, CD_3OD) δ 8.01 (s, 1H), 7.36 (s, 1H), 6.24 (d, *J* = 9.8 Hz, 1H), 5.46 (dd, *J* = 9.3, 1.5 Hz, 1H), 3.80 (s, 1H), 3.65 – 3.59 (m, 1H), 3.17 (t, *J* = 8.5 Hz, 1H), 2.99 (d, *J* = 9.3 Hz, 1H), 2.85 (t, *J* = 10.4 Hz, 1H), 2.67 (ddd, *J* = 9.9, 4.6, 1.9 Hz, 1H), 2.37 – 2.30 (m, 1H), 2.13 – 2.05 (m, 1H), 2.00 – 1.95 (m, 1H), 1.87 (m, 1H), 1.85 (m, 1H), 1.85 (s, 3H), 1.62 – 1.57 (m, 2H), 1.54 – 1.48 (m, 3H), 1.13 – 1.03 (m, 2H), 0.84 (s, 3H).
^{13}C NMR (151 MHz, CD_3OD) δ 209.9, 171.6, 164.0, 153.6, 150.8, 118.3, 114.1, 81.4, 76.7, 73.5, 70.8, 64.5, 60.8, 58.0, 54.9, 51.0, 47.0, 41.7, 37.8, 34.0, 32.3, 26.8, 21.4, 20.3, 17.9.
HRMS (ESI): calculated for $C_{25}H_{31}O_8^+$ $[M+H]^+$ 459.2014, found 459.2000.
HRMS (ESI): calculated for $C_{25}H_{30}O_8Cl^-$ $[M+Cl]^-$ 493.1635, found 493.1633.
$[\alpha]_D^{25}$ = -17.0 (*c* = 0.10 in MeOH).
$[\alpha]_D^{27}$ for isolated bufogargarizin A = -24.5 (c = 0.2 in MeOH), reported by Ye et al.[7]
R*f* = 0.18 (ethyl acetate in hexanes, 9:1).
IR (film, cm^{-1}): 3358, 2923, 2852, 1719, 1635, 1558, 1457, 1376, 1241, 1048, 883, 610.

a. Comparison of the ^{1}H NMR data of our synthetic bufogargarizin A with the authentic sample and the previously reported synthetic material

The ^{1}H NMR spectrum of synthetic **bufogargarizin A** (recorded in CD_3OD) closely matches that of the isolated natural product[7] and the synthetic sample reported by the Li research group[1]. Chemical shifts, multiplicities, and coupling constants for all key protons are consistent within ±0.03 ppm, confirming that our synthetic material is structurally identical to both the natural isolated and the previously reported synthetic compound.

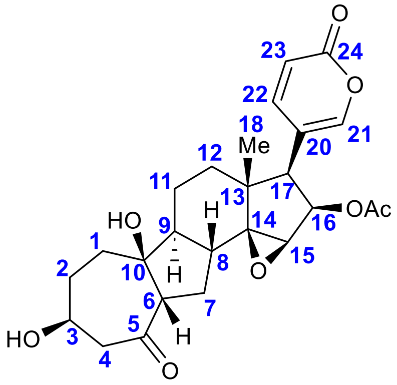

Table S13. Comparison of ^{1}H NMR data.

Position	Synthetic[a] (Syn.) 600 MHz	Isolated[b] (Iso.) 400 MHz	Li's work[a] (Li) 400 MHz	Error (Iso.-Syn.)	Error (Li-Syn.)
**1α**	1.06[c]	1.05[c]	1.05[c]	-0.01	-0.01
**1β**	1.98, m	1.95, m	1.95, m	-0.03	-0.03
**2α**	1.85[c], m	1.85, m	1.85, m	0	0
**2β**	2.09, m	2.10, m	2.10, m	0.01	0.01
**3α**	3.62, m	3.62, m	3.62, m	0	0
**4α**	2.67 (dddd, 9.9, 4.6, 1.9, 2)	2.68 (dd, 10.0, 4.6)	2.68 (dd, 9.9, 4.5)	0.01	0.01
**4β**	2.85 (t, 10.4)	2.85 (dd, 10.0, 10.0)	2.85 (dd, 10.3, 10.3)	0	0
**6β**	3.17 (t, 8.5)	3.17 (dd, 8.4, 8.4)	3.17 (dd, 8.5, 8.5)	0	0
**7α**	1.58[c]	1.58[c]	1.58[c]	0	0
**7β**	1.58[c]	1.58[c]	1.58[c]	0	0
**8β**	2.34, m	2.34, m	2.33, m	0	-0.01
**9α**	1.11[c]	1.09[c]	1.09[c]	-0.02	-0.02
**11α**	1.51[c]	1.51[c]	1.51[c]	0	0
**11β**	1.51[c]	1.51[c]	1.51[c]	0	0
**12α**	1.53[c]	1.53[c]	1.53[c]	0	0
**12β**	1.87[c]	1.87[c]	1.87[c]	0	0
**15**	3.80, s	3.80 (d, 1.3)	3.80 (d, 1.5)	0	0
**16**	5.46 (dd, 9.3, 1.5)	5.46 (dd, 8.6, 1.3)	5.46 (dd, 9.3, 1.5)	0	0

Position	Synthetic[a] (Syn.) 600 MHz	Isolated[b] (Iso.) 400 MHz	Li's work[a] (Li) 400 MHz	Error (Iso.-Syn.)	Error (Li-Syn.)
**17**	2.99 (d, 9.3)	2.98 (d, 8.6)	2.99 (d, 9.3)	-0.01	0
**18**	0.84, s	0.84, s	0.84, s	0	0
**22**	7.36, s	7.36 (d,1.0)	7.36, s	0	0
**23**	8.01, s	8.01 (dd, 8.0, 1.0)	8.01 (d, 9.5)	0	0
**24**	6.24 (d, 9.8)	6.23(d, 8.0)	6.24 (dd, 9.8)	-0.01	0
**CH_3 (Ac)**	1.85, s	1.85, s	1.85, s	0	0

[a]Chemical shifts referenced to CD_3OD at 3.30 ppm.
[b]Chemical shift of CD_3OD not reported. [c]Overlapping signals.

b. Comparison of the ^{13}C NMR data of our synthetic bufogargarizin A with the authentic sample and the previously reported synthetic material

The ^{13}C NMR spectrum of synthetic **bufogargarizin A** (recorded in CD_3OD) closely matches that of the isolated natural product[7] and the synthetic sample reported by the Li research group[1]. Chemical shifts for all key carbon resonances are consistent within ±0.1 ppm, confirming that our synthetic material is structurally identical to both the natural isolate and the previously reported synthetic compound.

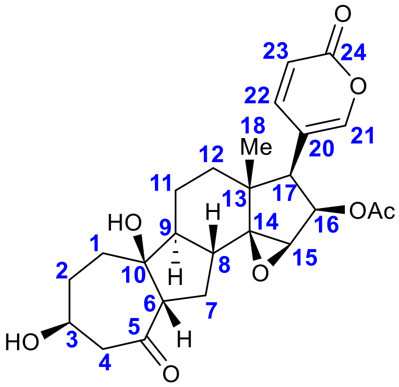

Table S14. Comparison of ^{13}C NMR data.

Position	Synthetic[a] (Syn.) 151 MHz	Isolated[b] (Iso.) 100 MHz	Li's work[a] (Li) 125 MHz	Error (Iso.-Syn.)	Error (Li-Syn.)
**1**	34.0	34.0	34.0	0	0
**2**	32.3	32.3	32.2	0	-0.1
**3**	70.8	70.8	70.8	0	0
**4**	54.9	54.9	54.8	0	-0.1
**5**	209.9	209.9	209.9	0	0
**6**	64.5	64.5	64.5	0	0
**7**	26.8	26.8	26.8	0	0
**8**	37.8	37.8	37.8	0	0

Position	Synthetic[a] (Syn.) 151 MHz	Isolated[b] (Iso.) 100 MHz	Li's work[a] (Li) 125 MHz	Error (Iso.-Syn.)	Error (Li-Syn.)
**9**	58.0	58.0	58.0	0	0
**10**	81.4	81.4	81.3	0	-0.1
**11**	21.4	21.4	21.4	0	0
**12**	41.7	41.7	41.6	0	-0.1
**13**	47.0	47.0	47.0	0	0
**14**	73.5	73.5	73.5	0	0
**15**	60.8	60.8	60.7	0	-0.1
**16**	76.7	76.7	76.6	0	-0.1
**17**	51.0	50.9	50.9	-0.1	-0.1
**18**	17.9	17.8	17.8	-0.1	-0.1
**20**	118.3	118.2	118.2	-0.1	-0.1
**21**	153.6	153.6	153.6	0	0
**22**	150.8	150.8	150.8	0	0
**23**	114.1	114.1	114.1	0	0
**24**	164.0	163.9	163.9	-0.1	-0.1
**C=O (Ac)**	171.6	171.6	171.5	0	-0.1
**CH_3 (Ac)**	20.3	20.3	20.3	0	0

[a]Chemical shifts referenced to CD_3OD at 49 ppm. [b]Chemical shift of CD_3OD not reported.

4. X-Ray Crystallography

i. Synthesis of compounds 19, 20 and 64

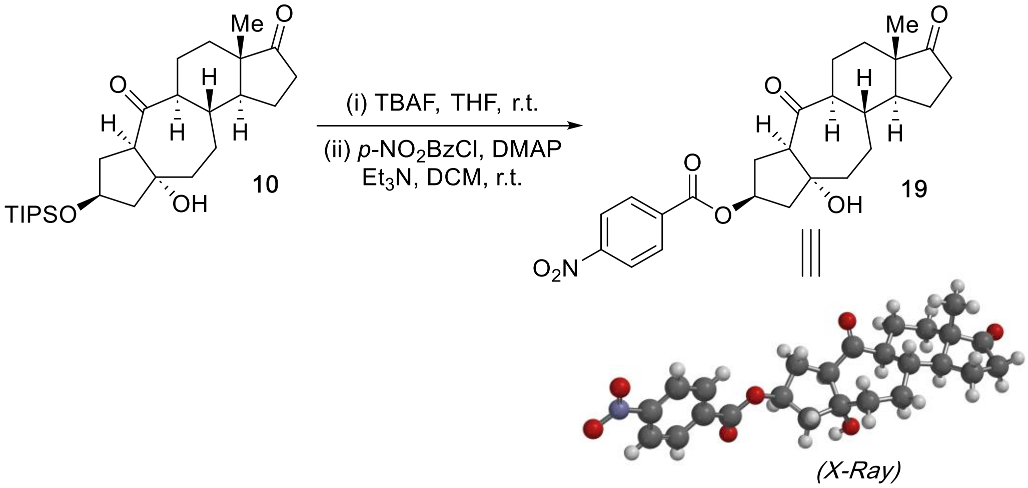

To a solution of compound **10** (72.3 mg, 0.156 mmol, 1.0 equiv) in THF (7.8 mL) was added a solution of tetrabutylammonium fluoride (TBAF, 0.312 mmol, 2.0 equiv) in THF (0.31 mL, 1 M). The reaction mixture was stirred at room temperature overnight. The reaction was quenched with saturated aqueous NH_4Cl (10 mL) and extracted with EtOAc (6 × 5 mL). The combined organic layers were washed with a 1:1 mixture of water/brine (4 × 5 mL), dried over anhydrous Na_2SO_4, filtered, and concentrated under reduced pressure. The resulting residue was used directly in the next step without further purification.

To a solution of the crude product in anhydrous DCM (7.8 mL) were added Et_3N (0.5 mL), DMAP (9.5 mg, 0.078 mmol, 0.5 equiv), and 4-nitrobenzoyl chloride (*p*-NO_2BzCl, 95.7 mg, 0.515 mmol, 3.3 equiv). The reaction mixture was stirred at room temperature for 5 h, then quenched with saturated aqueous $NaHCO_3$ (10 mL) and extracted with DCM (2 × 5 mL). The combined organic layers were washed with H_2O (1 × 5 mL) and brine (1 × 5 mL), dried over anhydrous Na_2SO_4, filtered, and concentrated under reduced pressure. The crude product was purified by flash column chromatography on silica gel (1:4 to 1:1, EtOAc:hexanes) to afford compound **19** as a white solid (28.0 mg, 39% over 2 steps).

^{1}H NMR (500 MHz, $CDCl_3$) δ 8.29 (d, *J* = 8.5 Hz, 2H), 8.20 (d, *J* = 8.7 Hz, 2H), 5.53 (tt, *J* = 8.6, 6.6 Hz, 1H), 3.20 (t, *J* = 8.5 Hz, 1H), 2.53 – 2.41 (m, 3H), 2.39 – 2.28 (m, 2H), 2.15 – 1.99 (m, 4H), 1.89 – 1.77 (m, 3H), 1.75 – 1.66 (m, 1H), 1.63 – 1.59 (m, 2H), 1.55 – 1.36 (m, 3H), 1.29 (m, 1H), 0.95 (s, 3H).
^{13}C NMR (126 MHz, $CDCl_3$) δ 220.2, 210.9, 164.6, 150.7, 135.7, 130.9, 123.7, 79.0, 75.0, 61.5, 58.0, 50.8, 49.0, 47.9, 40.7, 39.9, 35.7, 31.8, 30.4, 26.3, 23.3, 22.3, 13.9.
HRMS (ESI): calculated for $C_{25}H_{29}NO_7Cl^-$ $[M+Cl]^-$ 490.1638, found 490.1632.
R*f* = 0.16 (ethyl acetate in hexanes, 1:1).

Note: The structure of compound **19** was confirmed by single crystal X-ray diffraction analysis. Suitable crystals were grown from a methanol/DCM mixture (1:3) at 25 °C via slow evaporation.

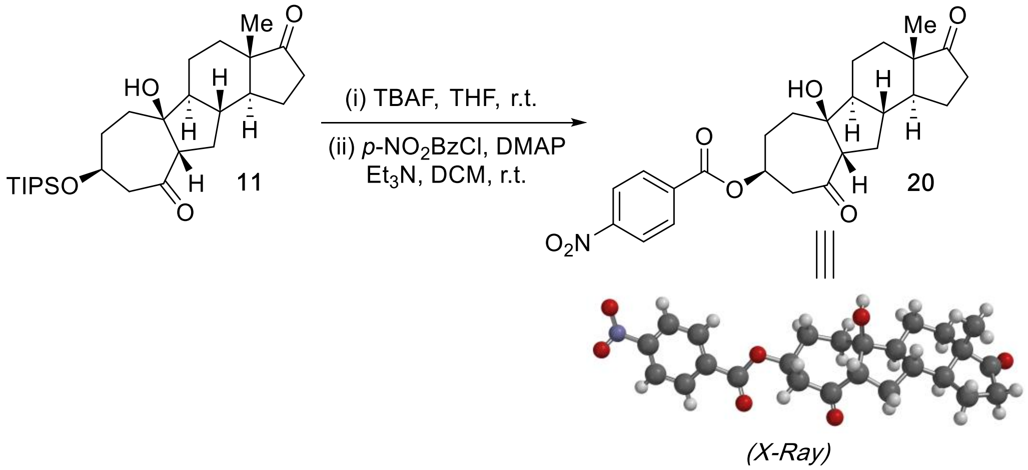

To a solution of compound **11** (42.0 mg, 0.091 mmol, 1.0 equiv) in THF (4.54 mL) was added a solution of tetrabutylammonium fluoride (TBAF, 0.181 mmol, 2.0 equiv) in THF (0.18 mL, 1 M). The reaction mixture was stirred at room temperature overnight. The reaction was quenched with saturated aqueous NH_4Cl (5 mL) and extracted with EtOAc (6 × 3 mL). The combined organic layers were washed with a 1:1 mixture of water/brine (4 × 5 mL), dried over anhydrous Na_2SO_4, filtered, and concentrated under reduced pressure. The resulting residue was used directly in the next step without further purification.

To a solution of the crude product in anhydrous DCM (4.54 mL) were added Et_3N (0.5 mL), DMAP (5.5 mg, 0.045 mmol, 0.5 equiv), and 4-nitrobenzoyl chloride (*p*-NO_2BzCl, 55.6 mg, 0.300 mmol, 3.3 equiv). The reaction mixture was stirred at room temperature for 5 h, then quenched with saturated aqueous $NaHCO_3$ (5 mL) and extracted with DCM (2 × 5 mL). The combined organic layers were washed with H_2O (1 × 5 mL) and brine (1 × 5 mL), dried over anhydrous Na_2SO_4, filtered, and concentrated under reduced pressure. The crude product was purified by flash column chromatography on silica gel (1:4 to 1:1, EtOAc:hexanes) to afford compound **20** as a white solid (18.2 mg, 44% over 2 steps).

^{1}H NMR (500 MHz, $CDCl_3$) δ 8.30 (d, *J* = 8.7 Hz, 2H), 8.20 (d, *J* = 8.5 Hz, 2H), 5.14 (tt, *J* = 10.3, 4.4 Hz, 1H), 3.25 (t, *J* = 8.4 Hz, 1H), 3.11 (t, *J* = 10.6 Hz, 1H), 2.97 (ddd, *J* = 10.5, 4.6, 1.9 Hz, 1H), 2.47 (dd, *J* = 19.3, 8.7 Hz, 1H), 2.40 – 2.31 (m, 1H), 2.16 – 2.09 (m, 2H), 2.05 – 1.96 (m, 2H), 1.94 – 1.87 (m, 2H), 1.84 (td, J = 7.3, 3.9 Hz, 1H), 1.79 – 1.73 (m, 1H), 1.71 – 1.65 (m, 1H), 1.60 – 1.53 (m, 1H), 1.47 – 1.39 (m, 2H), 1.30 – 1.23 (m, 2H), 0.92 (s, 3H), 0.81 (td, J = 12.0, 3.9 Hz, 1H).
^{13}C NMR (176 MHz, $CDCl_3$) δ 219.9, 206.2, 163.8, 150.8, 135.6, 130.9, 123.7, 80.6, 72.7, 63.9, 57.3, 51.0, 49.9, 49.0, 39.0, 35.9, 33.3, 31.0, 28.4, 28.0, 22.5, 19.2, 14.2.
HRMS (ESI): calculated for $C_{25}H_{29}NO_7Na^+$ [M+Na]$^+$ 478.1836, found 478.1838.
R_f = 0.26 (ethyl acetate in hexanes, 1:1).

Note: The structure of compound **20** was confirmed by single crystal X-ray diffraction analysis. Suitable crystals were grown from a methanol/DCM mixture (1:3) at 25 °C via slow evaporation.

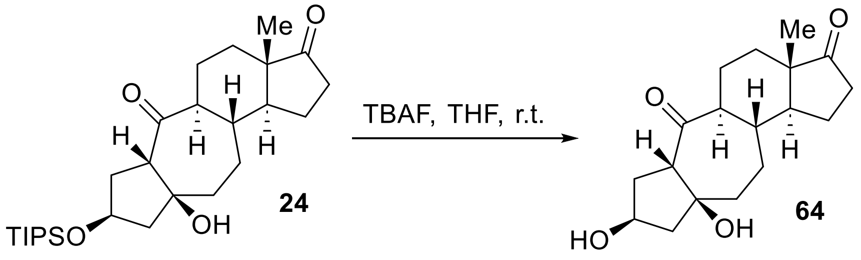

To a solution of compound **24** (6.2 mg, 0.013 mmol, 1.0 equiv) in THF (0.54 mL) was added a solution of tetrabutylammonium fluoride (TBAF, 0.013 mmol, 1.0 equiv) in THF (13.4 μL, 1 M). The reaction mixture was stirred at room temperature overnight, quenched with saturated aqueous NH_4Cl (1 mL) and extracted with EtOAc (6 × 1 mL). The combined organic layers were washed with a 1:1 mixture of water/brine (4 × 1 mL), dried over anhydrous Na_2SO_4, filtered, and concentrated under reduced pressure. The crude product was purified by flash column chromatography on silica gel (1:1, EtOAc:hexanes) to afford compound **64** as a white solid.

^{1}H NMR (700 MHz, C_6D_6) δ 4.07 (s, 1H), 3.87 (t, *J* = 4.0 Hz, 1H), 3.49 (t, *J* = 8.2 Hz, 1H), 3.31 (t, *J* = 5.8 Hz, 1H), 2.65 (ddd, *J* = 14.3, 7.8, 4.4 Hz, 1H), 2.33 (qd, *J* = 11.2, 5.6 Hz, 1H), 2.10 – 2.01 (m, 2H), 1.88 – 1.81 (m, 2H), 1.79 – 1.73 (m, 1H), 1.70 (dt, *J* = 18.6, 9.1 Hz, 2H), 1.58 (dd, *J* = 13.7, 3.2 Hz, 1H), 1.51 (ddd, *J* = 14.3, 8.6, 3.2 Hz, 1H), 1.43 (ddd, *J* = 14.1, 8.7, 6.1 Hz, 1H), 1.24 – 1.16 (m, 4H), 1.16 – 1.11 (m, 1H), 0.81 (td, *J* = 12.2, 6.5 Hz, 1H), 0.73 (td, *J* = 11.9, 5.9 Hz, 1H), 0.60 (s, 3H).
^{13}C NMR (176 MHz, C_6D_6) δ 217.4, 210.5, 83.5, 74.1, 59.0, 56.4, 50.3, 48.6, 47.6, 35.4, 35.0, 33.7, 33.4, 31.9, 26.6, 26.2, 21.7, 13.8.
HRMS (ESI): calculated for $C_{18}H_{26}O_4Cl^-$ $[M+Cl]^-$ 341.1525, found 341.1526.
R*f* = 0.15 (ethyl acetate in hexanes, 6:4).

***Note**:* The structure of compound **64** was confirmed by single-crystal X-ray diffraction analysis. Suitable crystals were grown from methanol at 25 °C via slow evaporation.

ii. X-ray crystallographic data of 19

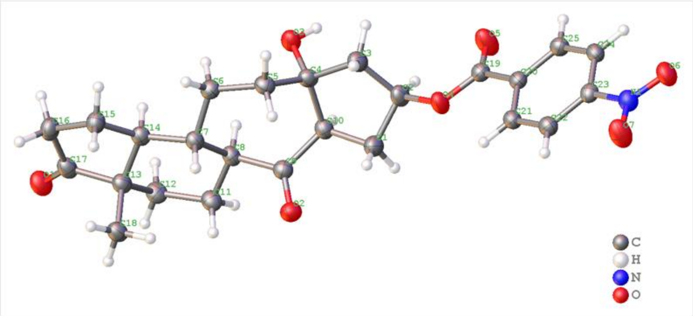

Experimental. Single colourless block-shaped crystals of **19** were used as supplied. A suitable crystal with dimensions 0.16 × 0.14 × 0.11 mm^3 was selected and mounted on a dtrek-CrysAlisPro-abstract goniometer imported rigaku-d*trek images diffractometer. The crystal was kept at a steady T = 85 K during data collection. The structure was solved with the ShelXT 2018/2 (Sheldrick, 2018)[8] solution program using dual methods and by using Olex2 1.5-alpha (Dolomanov et al., 2009)[9] as the graphical interface. The model was refined with ShelXL 2019/3 (Sheldrick, 2015)[10] using full matrix least squares minimization on F^2.

Crystal Data. $C_{25}H_{29}NO_7$, M_r = 455.49, orthorhombic, $P2_12_12_1$ (No. 19), a = 10.2506(2) Å, b = 11.2665(2) Å, c = 18.9402(3) Å, $\alpha = \beta = \gamma$ = 90°, V = 2187.37(7) Å^3, T = 85 K, Z = 4, Z' = 1, μ(Cu K$_\alpha$) = 0.835, 31764 reflections measured, 4079 unique (R_{int} = 0.0680) which were used in all calculations. The final wR_2 was 0.0926 (all data) and R_1 was 0.0368 (I≥2 s(I)).

Compound	19
CCDC	2478760
Formula	$C_{25}H_{29}NO_7$
$D_{calc.}$/ g cm^{-3}	1.383
μ/mm^{-1}	0.835
Formula Weight	455.49
Colour	colourless
Shape	block-shaped
Size/mm^3	0.16×0.14×0.11
T/K	85
Crystal System	orthorhombic
Flack Parameter	0.02(9)
Hooft Parameter	0.00(8)
Space Group	$P2_12_12_1$
a/Å	10.2506(2)
b/Å	11.2665(2)
c/Å	18.9402(3)
α/°	90
β/°	90
γ/°	90
V/Å^3	2187.37(7)
Z	4
Z'	1
Wavelength/Å	1.54184
Radiation type	Cu K$_\alpha$
Θ_{min}/°	4.567
Θ_{max}/°	69.385
Measured Refl's.	31764
Indep't Refl's	4079
Refl's I≥2 s(I)	3888
R_{int}	0.0680
Parameters	301
Restraints	0
Largest Peak	0.226
Deepest Hole	-0.266
GooF	1.095
wR_2 (all data)	0.0926
wR_2	0.0887
R_1 (all data)	0.0395
R_1	0.0368

Structure Quality Indicators for 19

**Reflections:**	d min (CuKα) 2Θ=138.8° 0.82	I/σ(I) 30.0	Rint m=7.81 6.80%	Full 135.4° 99.8
**Refinement:**	Shift 0.000	Max Peak 0.2	Min Peak -0.3	GooF 1.095
	Hooft .00(8)			

A colourless block-shaped crystal with dimensions 0.16 × 0.14 × 0.11 mm^3 was mounted. Data were collected using a dtrek-CrysAlisPro-abstract goniometer imported rigaku-d*trek images diffractometer operating at T = 85 K.

Data were measured using ω scans with Cu K$_\alpha$ radiation. The diffraction pattern was indexed and the total number of runs and images was based on the strategy calculation from the program DTREK_VERSION=d*TREK version 9.9.9.4 W9RSSI – Aug 22, 2012. The maximum resolution that was achieved was Θ = 69.385° (0.82 Å).

The unit cell was refined using CrysAlisPro 1.171.43.130a (Rigaku OD, 2024)[20] on 14671 reflections, 46% of the observed reflections.

Data reduction, scaling and absorption corrections were performed using CrysAlisPro 1.171.43.130a (Rigaku OD, 2024). The final completeness is 99.90 % out to 69.385° in Θ. A multi-scan absorption correction was performed using CrysAlisPro 1.171.43.130a (Rigaku Oxford Diffraction, 2024). Empirical absorption correction using spherical harmonics, implemented in SCALE3 ABSPACK scaling algorithm. The absorption coefficient μ of this material is 0.835 mm^{-1} at this wavelength (λ = 1.54184 Å), and the minimum and maximum transmissions are 0.859 and 1.000.

The structure was solved and the space group $P2_12_12_1$ (# 19) determined by the ShelXT 2018/2 (Sheldrick, 2018) structure solution program using dual methods and refined by full matrix least squares minimization on F^2 using version 2019/3 of ShelXL 2019/3 (Sheldrick, 2015). All non-hydrogen atoms were refined anisotropically. Hydrogen atom positions were calculated geometrically and refined using the riding model.

_exptl_absorpt_process_details: CrysAlisPro 1.171.43.130a (Rigaku Oxford Diffraction, 2024) using spherical harmonics, implemented in SCALE3 ABSPACK scaling algorithm.

There is a single formula unit in the asymmetric unit, which is represented by the reported sum formula. In other words: Z is 4 and Z' is 1. The moiety formula is $C_{25}H_{29}NO_7$.

The Flack parameter was refined to 0.02(9). Determination of absolute structure using Bayesian statistics on Bijvoet differences using the Olex2 results in 0.00(8). The chiral atoms in this structure are: C2(S), C4(R), C7(S), C8(S), C10(R), C13(S), C14(S).

Note: The Flack parameter is used to determine chirality of the crystal studied, the value should be near 0, a value of 1 means that the stereochemistry is wrong and the model should be inverted. A value of 0.5 means that the crystal consists of a racemic mixture of the two enantiomers.

Table S15. Fractional Atomic Coordinates (×10^4^) and Equivalent Isotropic Displacement Parameters (Å^2×10^3) for **19**. U_{eq} is defined as 1/3 of the trace of the orthogonalised U_{ij}.

Atom	x	y	z	U_{eq}
O1	3609(2)	8895.2(18)	10481.2(9)	41.1(5)
O2	2809.9(18)	6284.8(15)	7150.5(9)	34.8(4)
O3	3187.6(19)	10123.5(16)	6477.7(9)	34.7(4)
O4	3066.0(19)	7399.1(16)	4569.8(9)	34.3(4)
O5	2057(2)	8741.4(19)	3898.6(10)	47.5(6)
O6	4179(2)	5669.7(18)	964.4(9)	40.1(5)
O7	5100(2)	4382(2)	1638.3(10)	46.7(5)
N1	4479(2)	5291(2)	1546.8(11)	31.2(5)
C1	2460(3)	7198(2)	5803.2(13)	34.8(6)
C2	2685(3)	8052(2)	5202.6(13)	33.1(6)
C3	3779(3)	8840(3)	5461.5(13)	35.3(6)
C4	3599(3)	8962(2)	6273.2(13)	28.7(5)
C5	4904(3)	8731(2)	6641.0(13)	30.2(6)
C6	5004(3)	9081(2)	7422.0(13)	29.4(6)
C7	4353(2)	8253(2)	7960.1(13)	26.9(5)
C8	2871(2)	8092(2)	7802.1(12)	27.5(5)
C9	2730(2)	7361(2)	7135.0(13)	28.4(5)
C10	2520(2)	8011(2)	6447.1(13)	29.0(6)
C11	2104(3)	7496(2)	8406.8(13)	31.6(6)
C12	2329(3)	8093(3)	9123.5(13)	32.5(6)
C13	3780(3)	8111(2)	9278.9(13)	29.5(5)
C14	4489(2)	8799(2)	8693.1(13)	27.3(5)
C15	5848(3)	9039(2)	9005.4(13)	32.6(6)
C16	5495(3)	9418(3)	9764.8(13)	35.5(6)
C17	4201(3)	8813(2)	9924.7(13)	32.7(6)
C18	4320(3)	6844(2)	9397.2(14)	33.1(6)
C19	2715(2)	7867(2)	3953.2(13)	29.0(6)
C20	3208(2)	7181(2)	3334.1(13)	26.2(5)
C21	3841(2)	6098(2)	3418.4(13)	28.4(5)
C22	4275(2)	5478(2)	2832.3(13)	30.4(5)
C23	4053(2)	5959(2)	2171.2(13)	26.6(5)
C24	3446(2)	7045(2)	2075.7(13)	28.7(5)
C25	3015(3)	7657(2)	2663.2(13)	29.0(5)

Table S16. Anisotropic Displacement Parameters (×10^4^) for **19**. The anisotropic displacement factor exponent takes the form: $-2p^2[h^2a^{*2} \times U_{11} + ... +2hka^* \times b^* \times U_{12}]$.

Atom	U_{11}	U_{22}	U_{33}	U_{23}	U_{13}	U_{12}
O1	51.1(12)	44.2(11)	28.0(9)	-2.7(9)	5.5(9)	8.1(11)
O2	42.9(10)	27.9(10)	33.5(10)	-0.3(7)	-0.1(9)	-0.5(8)
O3	44.3(11)	28.0(9)	31.8(9)	-1.1(7)	-8.6(8)	8.0(8)
O4	48.3(11)	31.8(10)	22.7(8)	-1.6(7)	-3.2(8)	8.9(9)
O5	67.0(14)	43.0(12)	32.6(10)	-0.9(9)	-2.5(10)	25.2(11)
O6	49.9(12)	45.2(11)	25.2(9)	1.9(8)	-0.1(8)	3.2(10)
O7	56.8(13)	48.8(12)	34.6(10)	-4.4(9)	-1.4(10)	23.7(11)
N1	30.3(11)	34.5(12)	28.7(11)	-0.5(9)	-0.2(9)	2.4(10)
C1	44.6(15)	33.1(14)	26.8(13)	-2.2(11)	0.1(11)	-3.0(12)
C2	43.7(15)	31.2(13)	24.2(12)	-3.9(10)	0.2(11)	5.4(13)

Atom	U_{11}	U_{22}	U_{33}	U_{23}	U_{13}	U_{12}
C3	46.1(16)	33.0(14)	26.8(13)	0.7(11)	2.3(11)	-1.5(13)
C4	34.4(13)	23.8(12)	27.9(12)	0.2(10)	1.8(11)	2.5(11)
C5	32.0(13)	29.8(13)	28.6(13)	2.7(10)	3.3(11)	0.3(11)
C6	29.1(12)	29.5(13)	29.7(13)	0.3(10)	-0.8(10)	-1.6(11)
C7	29.6(12)	26.7(12)	24.4(12)	-0.6(10)	0.4(10)	3.8(10)
C8	29.0(12)	30.5(13)	23.1(12)	2.0(10)	-0.1(10)	4.2(11)
C9	27.5(11)	28.9(13)	28.7(12)	0.7(10)	1.6(10)	-0.1(11)
C10	30.7(13)	30.6(13)	25.6(12)	0.4(10)	-0.5(10)	2.4(11)
C11	30.3(12)	35.9(14)	28.5(12)	2.5(11)	2.4(11)	-0.2(11)
C12	31.5(13)	37.4(14)	28.6(13)	0.5(11)	7.0(11)	1.0(12)
C13	34.5(13)	28.7(12)	25.2(12)	-0.4(10)	2.6(11)	2.4(11)
C14	29.8(12)	25.6(12)	26.4(12)	-0.5(10)	0.2(10)	4.7(11)
C15	34.2(14)	35.2(14)	28.4(13)	-3.7(11)	-1.0(11)	-1.2(12)
C16	41.4(15)	35.5(14)	29.6(14)	-6.9(11)	-3.4(12)	2.4(13)
C17	41.0(14)	30.7(13)	26.4(13)	0.9(11)	0.2(11)	9.7(12)
C18	42.2(15)	29.2(13)	27.8(13)	1.4(11)	0.0(11)	4.0(12)
C19	31.6(12)	26.7(13)	28.7(13)	-0.1(10)	-2.5(10)	-1.8(11)
C20	27.5(11)	24.8(12)	26.4(12)	0.0(10)	-2.2(10)	-3.4(10)
C21	31.5(13)	28.4(12)	25.1(12)	2.6(10)	-2.2(10)	-1.5(11)
C22	31.9(13)	29.6(13)	29.6(13)	1.3(11)	-3.1(11)	0.3(11)
C23	24.0(11)	30.2(12)	25.7(12)	-1.7(10)	1.3(10)	-3.4(10)
C24	31.0(12)	30.0(13)	24.9(12)	4.2(10)	-3.2(10)	-3.5(11)
C25	31.5(13)	25.4(12)	30.0(12)	0.6(10)	-3.5(10)	1.5(11)

Table S17. Bond Lengths in Å for **19**.

Atom	Atom	Length/Å	Atom	Atom	Length/Å
O1	C17	1.220(3)	C8	C9	1.515(3)
O2	C9	1.216(3)	C8	C11	1.543(3)
O3	C4	1.428(3)	C9	C10	1.510(3)
O4	C2	1.459(3)	C11	C12	1.532(3)
O4	C19	1.331(3)	C12	C13	1.517(4)
O5	C19	1.198(3)	C13	C14	1.536(3)
O6	N1	1.222(3)	C13	C17	1.519(3)
O7	N1	1.218(3)	C13	C18	1.548(4)
N1	C23	1.468(3)	C14	C15	1.538(4)
C1	C2	1.508(4)	C15	C16	1.544(3)
C1	C10	1.527(3)	C16	C17	1.521(4)
C2	C3	1.512(4)	C19	C20	1.493(3)
C3	C4	1.554(3)	C20	C21	1.391(3)
C4	C5	1.531(4)	C20	C25	1.393(3)
C4	C10	1.575(4)	C21	C22	1.385(3)
C5	C6	1.534(3)	C22	C23	1.383(3)
C6	C7	1.534(4)	C23	C24	1.385(3)
C7	C8	1.558(3)	C24	C25	1.381(4)
C7	C14	1.525(3)			

Table S18. Bond Angles in ° for **19**.

Atom	Atom	Atom	Angle/°
C19	O4	C2	116.6(2)
O6	N1	C23	118.3(2)
O7	N1	O6	123.6(2)
O7	N1	C23	118.1(2)
C2	C1	C10	102.3(2)
O4	C2	C1	109.8(2)
O4	C2	C3	111.3(2)
C1	C2	C3	104.1(2)
C2	C3	C4	106.5(2)
O3	C4	C3	112.6(2)
O3	C4	C5	106.9(2)
O3	C4	C10	111.0(2)
C3	C4	C10	103.3(2)
C5	C4	C3	109.4(2)
C5	C4	C10	113.8(2)
C4	C5	C6	117.0(2)
C5	C6	C7	117.1(2)
C6	C7	C8	111.5(2)
C14	C7	C6	108.7(2)
C14	C7	C8	108.12(19)
C9	C8	C7	108.45(19)
C9	C8	C11	109.5(2)
C11	C8	C7	113.9(2)
O2	C9	C8	121.0(2)
O2	C9	C10	120.9(2)
C10	C9	C8	118.0(2)
C1	C10	C4	105.6(2)
C9	C10	C1	113.8(2)
C9	C10	C4	114.2(2)
C12	C11	C8	112.9(2)
C13	C12	C11	109.0(2)
C12	C13	C14	109.3(2)
C12	C13	C17	116.2(2)
C12	C13	C18	111.5(2)
C14	C13	C18	113.6(2)
C17	C13	C14	100.7(2)
C17	C13	C18	105.2(2)
C7	C14	C13	114.3(2)
C7	C14	C15	120.3(2)
C13	C14	C15	103.9(2)
C14	C15	C16	101.2(2)
C17	C16	C15	105.4(2)
O1	C17	C13	126.4(3)
O1	C17	C16	124.9(3)
C13	C17	C16	108.7(2)
O4	C19	C20	113.1(2)
O5	C19	O4	123.6(2)
O5	C19	C20	123.3(2)
C21	C20	C19	121.5(2)
C21	C20	C25	120.6(2)
C25	C20	C19	118.0(2)
C22	C21	C20	120.0(2)
C23	C22	C21	118.4(2)
C22	C23	N1	118.7(2)
C22	C23	C24	122.5(2)
C24	C23	N1	118.8(2)
C25	C24	C23	118.6(2)
C24	C25	C20	119.8(2)

Table S19. Torsion Angles in ° for **19**.

Atom	Atom	Atom	Atom	Angle/°
O2	C9	C10	C1	-1.5(4)
O2	C9	C10	C4	-122.9(3)
O3	C4	C5	C6	44.5(3)
O3	C4	C10	C1	137.1(2)
O3	C4	C10	C9	-97.1(2)
O4	C2	C3	C4	-151.5(2)
O4	C19	C20	C21	6.6(3)
O4	C19	C20	C25	-173.4(2)
O5	C19	C20	C21	-172.5(3)
O5	C19	C20	C25	7.5(4)
O6	N1	C23	C22	173.7(2)
O6	N1	C23	C24	-6.3(3)
O7	N1	C23	C22	-5.6(3)
O7	N1	C23	C24	174.4(2)
N1	C23	C24	C25	178.3(2)
C1	C2	C3	C4	-33.3(3)

Atom	Atom	Atom	Atom	Angle/°
C2	O4	C19	O5	-4.1(4)
C2	O4	C19	C20	176.7(2)
C2	C1	C10	C4	-36.5(3)
C2	C1	C10	C9	-162.5(2)
C2	C3	C4	O3	-109.6(2)
C2	C3	C4	C5	131.7(2)
C2	C3	C4	C10	10.2(3)
C3	C4	C5	C6	166.6(2)
C3	C4	C10	C1	16.2(3)
C3	C4	C10	C9	142.0(2)
C4	C5	C6	C7	76.8(3)
C5	C4	C10	C1	-102.3(2)
C5	C4	C10	C9	23.5(3)
C5	C6	C7	C8	-57.3(3)
C5	C6	C7	C14	-176.4(2)
C6	C7	C8	C9	70.3(3)
C6	C7	C8	C11	-167.6(2)
C6	C7	C14	C13	175.2(2)
C6	C7	C14	C15	-60.2(3)
C7	C8	C9	O2	81.7(3)
C7	C8	C9	C10	-96.8(2)
C7	C8	C11	C12	51.4(3)
C7	C14	C15	C16	-172.8(2)
C8	C7	C14	C13	54.0(3)
C8	C7	C14	C15	178.6(2)
C8	C9	C10	C1	177.0(2)
C8	C9	C10	C4	55.6(3)
C8	C11	C12	C13	-55.9(3)
C9	C8	C11	C12	173.0(2)
C10	C1	C2	O4	162.3(2)
C10	C1	C2	C3	43.0(3)
C10	C4	C5	C6	-78.5(3)
C11	C8	C9	O2	-43.1(3)
C11	C8	C9	C10	138.4(2)
C11	C12	C13	C14	59.3(3)
C11	C12	C13	C17	172.3(2)
C11	C12	C13	C18	-67.1(3)
C12	C13	C14	C7	-61.7(3)
C12	C13	C14	C15	165.4(2)
C12	C13	C17	O1	36.3(4)
C12	C13	C17	C16	-143.3(2)
C13	C14	C15	C16	-43.5(2)
C14	C7	C8	C9	-170.33(19)
C14	C7	C8	C11	-48.2(3)
C14	C13	C17	O1	154.2(3)
C14	C13	C17	C16	-25.4(3)
C14	C15	C16	C17	26.8(3)
C15	C16	C17	O1	179.5(3)
C15	C16	C17	C13	-0.9(3)

Atom	Atom	Atom	Atom	Angle/˚
C17	C13	C14	C7	175.5(2)
C17	C13	C14	C15	42.5(2)
C18	C13	C14	C7	63.5(3)
C18	C13	C14	C15	-69.4(3)
C18	C13	C17	O1	-87.6(3)
C18	C13	C17	C16	92.9(2)
C19	O4	C2	C1	149.1(2)
C19	O4	C2	C3	-96.2(3)
C19	C20	C21	C22	179.4(2)
C19	C20	C25	C24	-179.4(2)
C20	C21	C22	C23	-0.5(4)
C21	C20	C25	C24	0.5(4)
C21	C22	C23	N1	-178.3(2)
C21	C22	C23	C24	1.6(4)
C22	C23	C24	C25	-1.7(4)
C23	C24	C25	C20	0.6(4)
C25	C20	C21	C22	-0.6(4)

Table S20. Hydrogen Fractional Atomic Coordinates (×10[4]) and Equivalent Isotropic Displacement Parameters (Å^2×10^3) for **19**. U_{eq} is defined as 1/3 of the trace of the orthogonalised U_{ij}.

Atom	x	y	Z	U_{eq}
H3	2639.38	10379.51	6185.37	52
H1A	3150.48	6583.73	5821.31	42
H1B	1598.82	6806.28	5763.62	42
H2	1881.14	8531.12	5110.89	40
H3A	3732	9628.76	5232.26	42
H3B	4635.73	8479.44	5351.86	42
H5A	5591.34	9163.59	6378.73	36
H5B	5101.49	7873.64	6601.48	36
H6A	4618.55	9881.26	7477.37	35
H6B	5940.33	9144.09	7544.67	35
H7	4794.12	7461.8	7950.96	32
H8	2483.76	8893.4	7714.32	33
H10	1665.27	8436.02	6480.39	35
H11A	1161.37	7519.79	8294.17	38
H11B	2367.03	6651.88	8439.19	38
H12A	1984.83	8913.51	9113.9	39
H12B	1864.11	7648.42	9497.83	39
H14	4048.36	9589.34	8666.48	33
H15A	6304.09	9681.96	8748.22	39
H15B	6396.69	8315.75	9002.16	39
H16A	6175.1	9157.04	10101.64	43
H16B	5405.28	10291.51	9796.21	43
H18A	5220.3	6892.37	9568.99	50
H18B	4301.13	6405.16	8950.56	50
H18C	3780.57	6431.68	9746.99	50
H21	3975.55	5783.74	3878.09	34
H22	4713.77	4740.5	2883.19	36

Atom	x	y	Z	U_{eq}
H24	3327.43	7362.79	1615.55	34
H25	2588.64	8399.89	2610.02	35

iii. X-ray crystallographic data of 20

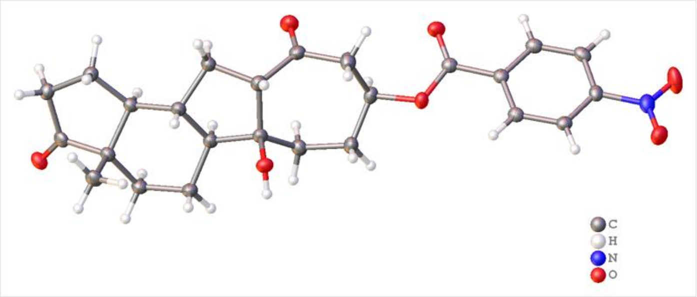

Experimental. Single colourless irregular-shaped crystals of **20** were used as supplied. A suitable crystal with dimensions 0.17 × 0.09 × 0.08 mm^3 was selected and mounted on a dtrek-CrysAlisPro-abstract goniometer imported rigaku-d*trek images diffractometer. The crystal was kept at a steady T = 85 K during data collection. The structure was solved with the ShelXT 2018/2 (Sheldrick, 2018)[8] solution program using dual methods and by using Olex2 1.5-alpha (Dolomanov et al., 2009)[9] as the graphical interface. The model was refined with ShelXL 2019/3 (Sheldrick, 2015)[10] using full matrix least squares minimization on F^2.

Crystal Data. $C_{25}H_{29}NO_7$, M_r = 455.49, orthorhombic, $P2_12_12_1$ (No. 19), a = 6.53180(10) Å, b = 15.11300(10) Å, c = 22.6735(2) Å, $\alpha = \beta = \gamma$ = 90°, V = 2238.22(4) Å^3, T = 85 K, Z = 4, Z' = 1, μ(Cu K_α) = 0.816, 34315 reflections measured, 4154 unique (R_{int} = 0.0336) which were used in all calculations. The final wR_2 was 0.0910 (all data) and R_1 was 0.0346 (I≥2 s(I)).

Compound	20
CCDC	2478759
Formula	$C_{25}H_{29}NO_7$
$D_{calc.}$/ g cm^{-3}	1.352
μ/mm^{-1}	0.816
Formula Weight	455.49
Colour	colourless
Shape	irregular-shaped
Size/mm^3	0.17×0.09×0.08
T/K	85
Crystal System	orthorhombic
Flack Parameter	-0.02(4)
Hooft Parameter	-0.01(3)
Space Group	$P2_12_12_1$
a/Å	6.53180(10)
b/Å	15.11300(10)
c/Å	22.6735(2)
α/°	90
β/°	90
γ/°	90
V/Å^3	2238.22(4)
Z	4
Z'	1
Wavelength/Å	1.54184
Radiation type	Cu K_α
Θ_{min}/°	3.515
Θ_{max}/°	69.338
Measured Refl's.	34315
Indep't Refl's	4154
Refl's I≥2 s(I)	4133
R_{int}	0.0336
Parameters	301
Restraints	0
Largest Peak	0.297
Deepest Hole	-0.231
GooF	1.079
wR_2 (all data)	0.0910
wR_2	0.0908
R_1 (all data)	0.0348
R_1	0.0346

Structure Quality Indicators for 20

**Reflections:**	d min (CuKα) 2Θ=138.7° 0.82	I/σ(I) 63.3	Rint m=8.28 3.36%	Full 135.4° 100	
**Refinement:**	Shift 0.001	Max Peak 0.3	Min Peak -0.2	GooF 1.079	Hooft -.01(3)

A colourless irregular-shaped crystal with dimensions 0.17 × 0.09 × 0.08 mm^3 was mounted. Data were collected using a dtrek-CrysAlisPro-abstract goniometer imported rigaku-d*trek images diffractometer operating at T = 85 K.

Data were measured using ω scans with Cu K_α radiation. The diffraction pattern was indexed, and the total number of runs and images was based on the strategy calculation from the program DTREK_VERSION=d*TREK version 9.9.9.4 W9RSSI – Aug 22, 2012. The maximum resolution that was achieved was Θ = 69.338° (0.82 Å).

The unit cell was refined using CrysAlisPro 1.171.43.130a (Rigaku OD, 2024)[20] on 25081 reflections, 73% of the observed reflections.

Data reduction, scaling and absorption corrections were performed using CrysAlisPro 1.171.43.130a (Rigaku OD, 2024). The final completeness is 100.00 % out to 69.338° in Θ. A multi-scan absorption correction was performed using CrysAlisPro 1.171.43.130a (Rigaku Oxford Diffraction, 2024). Empirical absorption correction using spherical harmonics, implemented in SCALE3 ABSPACK scaling algorithm. The absorption coefficient μ of this material is 0.816 mm^{-1} at this wavelength (λ = 1.54184 Å), and the minimum and maximum transmissions are 0.913 and 1.000.

The structure was solved and the space group $P2_12_12_1$ (# 19) determined by the ShelXT 2018/2 (Sheldrick, 2018) structure solution program using dual methods and refined by full matrix least squares minimization on F^2 using version 2019/3 of ShelXL 2019/3 (Sheldrick, 2015). All non-hydrogen atoms were refined anisotropically. Hydrogen atom positions were calculated geometrically and refined using the riding model.

_exptl_absorpt_process_details: CrysAlisPro 1.171.43.130a (Rigaku Oxford Diffraction, 2024) using spherical harmonics, implemented in SCALE3 ABSPACK scaling algorithm.

There is a single formula unit in the asymmetric unit, which is represented by the reported sum formula. In other words: Z is 4 and Z' is 1. The moiety formula is $C_{25}H_{29}NO_7$.

The Flack parameter was refined to -0.02(4). Determination of absolute structure using Bayesian statistics on Bijvoet differences using the Olex2 results in -0.01(3). The chiral atoms in this structure are: C3(S), C6(S), C8(S), C9(S), C10(S), C13(S), C14(S).

Note: The Flack parameter is used to determine chirality of the crystal studied, the value should be near 0, a value of 1 means that the stereochemistry is wrong and the model should be inverted. A value of 0.5 means that the crystal consists of a racemic mixture of the two enantiomers.

Table S21. Fractional Atomic Coordinates (×10^4) and Equivalent Isotropic Displacement Parameters (Å^2×10^3) for **20.** U_{eq} is defined as 1/3 of the trace of the orthogonalised U_{ij}.

Atom	X	y	z	U_{eq}
O1	-320(3)	-1615.7(11)	2774.9(7)	25.9(4)
O2	3693(2)	2786.3(10)	2922.1(7)	23.0(3)
O3	4646(3)	2197.7(11)	4867.3(7)	33.3(4)
O4	3588(3)	5007.2(10)	4544.3(7)	22.6(4)
O5	4171(3)	4764.2(11)	5512.0(7)	26.0(4)
O6	3994(3)	9484.5(11)	4997.5(8)	33.4(4)
O7	3562(4)	9277.5(12)	5931.1(8)	41.7(5)
N1	3833(3)	9005.9(13)	5427.0(9)	25.9(4)
C1	1414(4)	3009.0(15)	3757.1(10)	23.7(5)
C2	2078(4)	3966.6(15)	3888.3(10)	25.3(5)
C3	3304(4)	4051.9(14)	4451.6(10)	22.6(5)
C4	5401(4)	3603.6(15)	4435.3(12)	28.5(5)
C5	5039(4)	2606.6(15)	4415.2(10)	25.2(5)
C6	5003(4)	2173.5(15)	3818.3(10)	24.7(5)
C7	5171(4)	1159.4(14)	3842.2(10)	23.6(5)
C8	3908(4)	857.6(14)	3311.8(9)	20.1(4)
C9	2046(4)	1471.7(14)	3351.5(9)	21.0(5)
C10	2974(4)	2399.1(14)	3461.2(9)	21.4(5)
C11	531(4)	1342.8(14)	2844.7(10)	23.9(5)
C12	-150(4)	364.3(15)	2808.8(10)	23.6(5)
C13	1697(4)	-254.8(14)	2780.1(9)	20.7(5)
C14	3150(4)	-90.2(14)	3307.7(9)	19.3(5)
C15	4619(4)	-880.7(14)	3292.4(10)	22.0(5)
C16	3194(4)	-1657.2(15)	3128.7(10)	22.4(5)
C17	1281(4)	-1230.3(14)	2869.9(9)	20.9(5)
C18	2779(4)	-193.5(16)	2174.3(9)	26.1(5)
C19	3940(3)	5256.7(14)	5101.3(9)	20.6(4)
C20	3972(3)	6245.1(14)	5161.8(10)	19.4(4)
C21	3962(4)	6811.2(15)	4679.6(9)	19.8(4)
C22	3949(3)	7722.9(14)	4767.2(10)	20.1(4)
C23	3921(4)	8038.8(14)	5338.6(10)	20.6(5)
C24	3930(3)	7493.2(15)	5829.5(10)	22.1(5)
C25	3978(3)	6586.6(15)	5735.2(9)	21.0(4)

Table S22. Anisotropic Displacement Parameters (×10^4) for **20**. The anisotropic displacement factor exponent takes the form: $-2p^2[h^2a^{*2} \times U_{11}+ ... +2hka^* \times b^* \times U_{12}]$.

Atom	U_{11}	U_{22}	U_{33}	U_{23}	U_{13}	U_{12}
O1	27.3(9)	26.7(8)	23.7(8)	-4.8(7)	-2.6(7)	-4.8(7)
O2	25.1(8)	23.5(7)	20.5(7)	4.2(6)	-1.6(6)	0.1(7)
O3	46.1(11)	29.7(9)	24.1(8)	1.0(7)	-5.9(8)	3.6(8)
O4	30.9(9)	16.5(7)	20.4(7)	-0.7(6)	0.6(7)	-0.1(6)
O5	29.7(9)	23.5(8)	24.7(8)	5.5(6)	-4.9(7)	-4.1(7)
O6	42.4(10)	21.1(8)	36.7(10)	3.0(7)	-5.0(9)	-0.8(8)
O7	61.7(14)	28.5(9)	34.8(10)	-13.3(8)	11.2(9)	-8.1(9)
N1	21.7(10)	22.9(10)	33.1(11)	-4.6(9)	0.4(9)	-2.5(8)
C1	27.6(12)	23.2(11)	20.3(10)	-1.5(9)	-0.8(9)	-1.2(9)

Atom	U_{11}	U_{22}	U_{33}	U_{23}	U_{13}	U_{12}
C2	33.3(13)	19.6(10)	23.0(11)	-1.9(9)	-1.6(10)	3.2(10)
C3	25.4(11)	16.0(10)	26.5(11)	1.0(8)	1.2(9)	-0.8(9)
C4	24.2(12)	23.4(12)	37.9(13)	-5.4(10)	-4.7(10)	-1.3(10)
C5	25.8(12)	23.2(11)	26.7(12)	1.2(9)	-7.3(10)	2.7(10)
C6	27.6(12)	22.1(11)	24.5(11)	2.6(9)	-2.8(10)	-0.8(9)
C7	27.2(11)	21.1(10)	22.6(11)	-1.6(8)	-6.5(10)	0.8(9)
C8	23.4(11)	19.9(10)	17.2(9)	0.4(8)	-2.3(9)	-0.6(9)
C9	26.5(12)	18.7(10)	17.9(10)	0.9(8)	-2.3(9)	-0.3(9)
C10	27.4(12)	19.4(10)	17.4(10)	1.5(8)	-2.4(9)	-0.4(9)
C11	25.9(12)	21.5(10)	24.1(11)	0.5(9)	-7.2(9)	2.0(9)
C12	23.1(11)	23.8(10)	24.0(11)	-1.1(9)	-5.6(9)	0.5(9)
C13	23.5(11)	20.7(10)	17.7(10)	-1.3(8)	-1.5(9)	-0.5(9)
C14	20.8(11)	20.5(10)	16.6(10)	-0.2(8)	-1.5(9)	-2.0(9)
C15	23.6(11)	20.7(10)	21.8(10)	0.9(8)	-0.6(9)	1.2(9)
C16	25.1(11)	19.6(10)	22.6(10)	-0.9(9)	1.5(9)	1.4(9)
C17	25.5(12)	23.5(10)	13.7(9)	-4.8(8)	1.8(9)	0.1(9)
C18	34.1(13)	27.4(11)	16.7(10)	0.4(9)	-0.5(10)	-2.4(10)
C19	17.8(10)	22.7(11)	21.3(10)	1.2(9)	0.0(9)	-0.8(9)
C20	15.2(10)	21.7(11)	21.2(10)	0.0(8)	-0.9(9)	0.1(9)
C21	17.1(10)	23.7(11)	18.5(10)	-0.2(8)	0.6(8)	-0.2(9)
C22	17.2(10)	21.2(10)	21.7(10)	1.9(8)	-2.1(8)	-0.6(9)
C23	16.6(10)	19.0(10)	26.0(11)	-3.1(9)	-1.2(9)	-1.1(9)
C24	17.5(10)	29.7(11)	19.2(10)	-2.9(9)	-0.8(9)	-2.4(9)
C25	17.9(10)	25.7(11)	19.5(10)	1.4(9)	0.1(9)	-0.6(9)

Table S23. Bond Lengths in Å for **20**.

Atom	Atom	Length/Å
O1	C17	1.216(3)
O2	C10	1.434(3)
O3	C5	1.224(3)
O4	C3	1.471(3)
O4	C19	1.338(3)
O5	C19	1.202(3)
O6	N1	1.218(3)
O7	N1	1.227(3)
N1	C23	1.476(3)
C1	C2	1.540(3)
C1	C10	1.529(3)
C2	C3	1.513(3)
C3	C4	1.528(3)
C4	C5	1.526(3)
C5	C6	1.503(3)
C6	C7	1.538(3)
C6	C10	1.590(3)
C7	C8	1.528(3)
C8	C9	1.533(3)

Atom	Atom	Length/Å
C8	C14	1.516(3)
C9	C10	1.547(3)
C9	C11	1.529(3)
C11	C12	1.546(3)
C12	C13	1.528(3)
C13	C14	1.547(3)
C13	C17	1.513(3)
C13	C18	1.548(3)
C14	C15	1.533(3)
C15	C16	1.543(3)
C16	C17	1.524(3)
C19	C20	1.500(3)
C20	C21	1.388(3)
C20	C25	1.399(3)
C21	C22	1.392(3)
C22	C23	1.381(3)
C23	C24	1.385(3)
C24	C25	1.387(3)

Table S24. Bond Angles in ° for **20**.

Atom	Atom	Atom	Angle/°
C19	O4	C3	115.68(16)
O6	N1	O7	124.0(2)
O6	N1	C23	118.43(19)
O7	N1	C23	117.6(2)
C10	C1	C2	117.6(2)
C3	C2	C1	113.10(19)
O4	C3	C2	105.73(17)
O4	C3	C4	109.00(18)
C2	C3	C4	114.6(2)
C5	C4	C3	107.44(19)
O3	C5	C4	120.4(2)
O3	C5	C6	122.1(2)
C6	C5	C4	117.3(2)
C5	C6	C7	113.65(18)
C5	C6	C10	112.23(19)
C7	C6	C10	106.94(18)
C8	C7	C6	103.37(18)
C7	C8	C9	101.62(17)
C14	C8	C7	117.61(17)
C14	C8	C9	108.26(19)
C8	C9	C10	104.31(19)
C11	C9	C8	113.11(17)
C11	C9	C10	119.33(18)
O2	C10	C1	110.27(18)
O2	C10	C6	104.40(18)
O2	C10	C9	111.15(17)
C1	C10	C6	117.45(18)
C1	C10	C9	110.8(2)
C9	C10	C6	102.36(17)
C9	C11	C12	110.34(18)
C13	C12	C11	111.16(19)
C12	C13	C14	110.67(17)
C12	C13	C18	111.22(18)
C14	C13	C18	113.34(19)
C17	C13	C12	116.70(19)
C17	C13	C14	99.36(17)
C17	C13	C18	105.06(17)
C8	C14	C13	110.92(17)
C8	C14	C15	122.1(2)
C15	C14	C13	103.97(17)
C14	C15	C16	102.73(18)
C17	C16	C15	105.39(18)
O1	C17	C13	126.7(2)
O1	C17	C16	124.8(2)
C13	C17	C16	108.49(19)
O4	C19	C20	111.68(18)
O5	C19	O4	125.3(2)
O5	C19	C20	123.0(2)
C21	C20	C19	122.8(2)
C21	C20	C25	120.3(2)
C25	C20	C19	116.91(19)
C20	C21	C22	119.8(2)
C23	C22	C21	118.4(2)
C22	C23	N1	118.0(2)
C22	C23	C24	123.2(2)
C24	C23	N1	118.7(2)
C23	C24	C25	117.7(2)
C24	C25	C20	120.5(2)

Table S25. Torsion Angles in ° for **20**.

Atom	Atom	Atom	Atom	Angle/°
O3	C5	C6	C7	-19.8(3)
O3	C5	C6	C10	101.7(3)
O4	C3	C4	C5	173.14(18)
O4	C19	C20	C21	-9.5(3)
O4	C19	C20	C25	169.5(2)
O5	C19	C20	C21	171.7(2)
O5	C19	C20	C25	-9.4(3)
O6	N1	C23	C22	-7.2(3)
O6	N1	C23	C24	174.1(2)
O7	N1	C23	C22	171.5(2)
O7	N1	C23	C24	-7.3(3)
N1	C23	C24	C25	179.0(2)
C1	C2	C3	O4	-173.33(19)
C1	C2	C3	C4	66.6(3)
C2	C1	C10	O2	-55.4(3)
C2	C1	C10	C6	63.9(3)

Atom	Atom	Atom	Atom	Angle/˚
C2	C1	C10	C9	-178.95(19)
C2	C3	C4	C5	-68.6(3)
C3	O4	C19	O5	5.2(3)
C3	O4	C19	C20	-173.55(18)
C3	C4	C5	O3	-80.5(3)
C3	C4	C5	C6	94.3(3)
C4	C5	C6	C7	165.6(2)
C4	C5	C6	C10	-72.9(3)
C5	C6	C7	C8	147.8(2)
C5	C6	C10	O2	123.15(19)
C5	C6	C10	C1	0.7(3)
C5	C6	C10	C9	-120.9(2)
C6	C7	C8	C9	-42.3(2)
C6	C7	C8	C14	-160.2(2)
C7	C6	C10	O2	-111.58(19)
C7	C6	C10	C1	126.0(2)
C7	C6	C10	C9	4.4(2)
C7	C8	C9	C10	46.1(2)
C7	C8	C9	C11	177.27(19)
C7	C8	C14	C13	173.38(19)
C7	C8	C14	C15	-63.6(3)
C8	C9	C10	O2	80.3(2)
C8	C9	C10	C1	-156.69(17)
C8	C9	C10	C6	-30.6(2)
C8	C9	C11	C12	55.3(2)
C8	C14	C15	C16	-165.62(18)
C9	C8	C14	C13	59.1(2)
C9	C8	C14	C15	-177.88(18)
C9	C11	C12	C13	-53.0(2)
C10	C1	C2	C3	-82.1(3)
C10	C6	C7	C8	23.4(2)
C10	C9	C11	C12	178.6(2)
C11	C9	C10	O2	-47.1(3)
C11	C9	C10	C1	75.9(2)
C11	C9	C10	C6	-158.08(19)
C11	C12	C13	C14	55.2(2)
C11	C12	C13	C17	167.79(18)
C11	C12	C13	C18	-71.7(2)
C12	C13	C14	C8	-59.2(2)
C12	C13	C14	C15	167.76(18)
C12	C13	C17	O1	25.6(3)
C12	C13	C17	C16	-151.95(18)
C13	C14	C15	C16	-39.4(2)
C14	C8	C9	C10	170.56(16)
C14	C8	C9	C11	-58.3(2)
C14	C13	C17	O1	144.5(2)
C14	C13	C17	C16	-33.0(2)
C14	C15	C16	C17	18.2(2)
C15	C16	C17	O1	-167.8(2)

Atom	Atom	Atom	Atom	Angle/˚
C15	C16	C17	C13	9.8(2)
C17	C13	C14	C8	177.48(18)
C17	C13	C14	C15	44.5(2)
C18	C13	C14	C8	66.5(2)
C18	C13	C14	C15	-66.5(2)
C18	C13	C17	O1	-98.1(3)
C18	C13	C17	C16	84.4(2)
C19	O4	C3	C2	156.28(19)
C19	O4	C3	C4	-80.1(2)
C19	C20	C21	C22	178.6(2)
C19	C20	C25	C24	-177.6(2)
C20	C21	C22	C23	-0.8(3)
C21	C20	C25	C24	1.4(3)
C21	C22	C23	N1	-177.9(2)
C21	C22	C23	C24	0.8(4)
C22	C23	C24	C25	0.3(4)
C23	C24	C25	C20	-1.4(3)
C25	C20	C21	C22	-0.3(3)

Table S26. Hydrogen Fractional Atomic Coordinates (×10[4]) and Equivalent Isotropic Displacement Parameters (Å^2×10^3) for **20**. U_{eq} is defined as 1/3 of the trace of the orthogonalised U_{ij}.

Atom	x	y	z	U_{eq}
H2	2687.93	2945.28	2716.62	35
H1A	998.15	2732.33	4134.37	28
H1B	182.97	3032.71	3502.88	28
H2A	2911.92	4187.9	3554.81	30
H2B	843.8	4344.01	3917.9	30
H3	2491.11	3803.09	4786.4	27
H4A	6173.4	3796.56	4082.3	34
H4B	6200.9	3762.58	4790.64	34
H6	6194.44	2402.75	3587.51	30
H7A	6613.45	965.8	3805.75	28
H7B	4596.05	924.86	4214.72	28
H8	4679.48	981.23	2939.85	24
H9	1302.34	1305.81	3720.78	25
H11A	1182.57	1518.86	2468.38	29
H11B	-681.01	1724.67	2907.53	29
H12A	-986.84	216.54	3159.2	28
H12B	-1009.18	277.71	2453.86	28
H14	2301.17	-163.02	3671.57	23
H15A	5268.99	-976.37	3681.76	26
H15B	5700.11	-796.61	2991.45	26
H16A	3856.49	-2049.08	2835.41	27
H16B	2847.86	-2010.41	3482.67	27
H18A	3915.45	-616.64	2161.47	39
H18B	3308.83	407.17	2117.47	39
H18C	1799.36	-332.37	1860.38	39
H21	3964.73	6577.01	4290.77	24

Atom	x	y	z	U_{eq}
H22	3959.22	8118.43	4441.56	24
H24	3903.38	7731.53	6217.16	27
H25	4015.84	6194.63	6062.54	25

iv. X-ray crystallographic data of 21

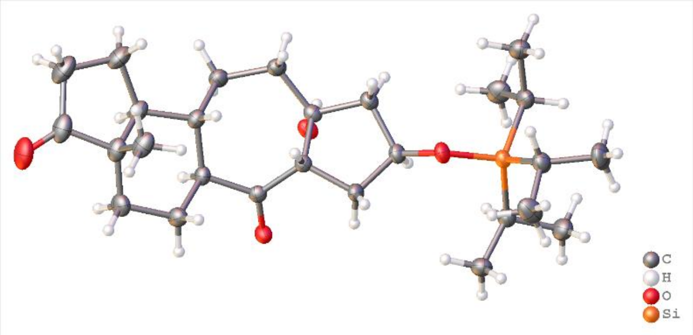

Experimental. Single colourless needle-shaped crystals of **21** were used as supplied. A suitable crystal with dimensions 0.14 × 0.04 × 0.02 mm^3 was selected and mounted on a XtaLAB Synergy, Dualflex, HyPix-Arc 150 diffractometer. The crystal was kept at a steady T = 100.01(10) K during data collection. The structure was solved with the ShelXT 2018/2 (Sheldrick, 2018)[8] solution program using dual methods and by using Olex2 1.5-alpha (Dolomanov et al., 2009)[9] as the graphical interface. The model was refined with ShelXL 2019/3 (Sheldrick, 2015)[10] using full matrix least squares minimization on F^2.

Crystal Data. $C_{27}H_{46}O_4Si$, M_r = 462.73, orthorhombic, $P2_12_12_1$ (No. 19), a = 8.22520(10) Å, b = 15.2526(2) Å, c = 21.8985(3) Å, $\alpha = \beta = \gamma$ = 90°, V = 2747.29(6) Å^3, T = 100.01(10) K, Z = 4, Z' = 1, μ(Cu K$_\alpha$) = 0.967, 23124 reflections measured, 5629 unique (R_{int} = 0.0434) which were used in all calculations. The final wR_2 was 0.0945 (all data) and R_1 was 0.0356 (I≥2 s(I)).

Compound	21
CCDC	2480819
Formula	$C_{27}H_{46}O_4Si$
$D_{calc.}$/ g cm^{-3}	1.119
μ/mm^{-1}	0.967
Formula Weight	462.73
Colour	colourless
Shape	needle-shaped
Size/mm^3	0.14×0.04×0.02
T/K	100.01(10)
Crystal System	orthorhombic
Flack Parameter	0.005(13)
Hooft Parameter	0.018(9)
Space Group	$P2_12_12_1$
a/Å	8.22520(10)
b/Å	15.2526(2)
c/Å	21.8985(3)
α/°	90
β/°	90
γ/°	90
V/Å^3	2747.29(6)
Z	4
Z'	1
Wavelength/Å	1.54184
Radiation type	Cu K$_\alpha$
Θ_{min}/°	3.531
Θ_{max}/°	76.126
Measured Refl's.	23124
Indep't Refl's	5629
Refl's I≥2 s(I)	5288
R_{int}	0.0434
Parameters	297
Restraints	0
Largest Peak	0.342
Deepest Hole	-0.209
GooF	1.051
wR_2 (all data)	0.0945
wR_2	0.0933
R_1 (all data)	0.0383
R_1	0.0356

Structure Quality Indicators for 21

**Reflections:**	d min (CuKα) 2Θ=152.3° 0.79	I/σ(I) 33.6	Rint m=4.12 4.34%	Full 135.4° 99% to 152.3° 100	
**Refinement:**	Shift 0.000	Max Peak 0.3	Min Peak -0.2	GooF 1.051	Hooft .018(9)

A colourless needle-shaped crystal with dimensions 0.14 × 0.04 × 0.02 mm^3 was mounted. Data were collected using a XtaLAB Synergy, Dualflex, HyPix-Arc 150 diffractometer operating at T = 100.01(10) K.

Data were measured using ω scans with Cu K_{α} radiation. The diffraction pattern was indexed and the total number of runs and images was based on the strategy calculation from the program CrysAlisPro system (CCD 44.118a 64-bit (release 22-07-2025)). The maximum resolution that was achieved was Θ = 76.126° (0.79 Å).

The unit cell was refined using CrysAlisPro 1.171.44.118a (Rigaku OD, 2025)[21] on 8327 reflections, 36% of the observed reflections.

Data reduction, scaling and absorption corrections were performed using CrysAlisPro 1.171.44.118a (Rigaku OD, 2025). The final completeness is 100.00 % out to 76.126° in Θ. A gaussian absorption correction was performed using CrysAlisPro 1.171.44.118a (Rigaku Oxford Diffraction, 2025). Numerical absorption correction based on gaussian integration over a multifaceted crystal model. Empirical absorption correction using spherical harmonics, implemented in SCALE3 ABSPACK scaling algorithm. The absorption coefficient μ of this material is 0.967 mm^{-1} at this wavelength (λ = 1.54184 Å) and the minimum and maximum transmissions are 0.803 and 1.000.

The structure was solved and the space group $P2_12_12_1$ (# 19) determined by the ShelXT 2018/2 (Sheldrick, 2018) structure solution program using dual methods and refined by full matrix least squares minimization on F^2 using version 2019/3 of ShelXL 2019/3 (Sheldrick, 2015). All non-hydrogen atoms were refined anisotropically. Hydrogen atom positions were calculated geometrically and refined using the riding model.

_exptl_absorpt_process_details: CrysAlisPro 1.171.44.118a (Rigaku Oxford Diffraction, 2025). Numerical absorption correction based on gaussian integration over multifaceted crystal model. Empirical absorption correction using spherical harmonics, implemented in SCALE3 ABSPACK scaling algorithm.

There is a single formula unit in the asymmetric unit, which is represented by the reported sum formula. In other words: Z is 4 and Z' is 1. The moiety formula is $C_{27}H_{46}O_4Si$.

The Flack parameter was refined to 0.005(13). Determination of absolute structure using Bayesian statistics on Bijvoet differences using the Olex2 results in 0.018(9). The chiral atoms in this structure are: C2(S), C4(R), C7(S), C8(S), C10(S), C13(S), C14(S).

Note: The Flack parameter is used to determine chirality of the crystal studied, the value should be near 0, a value of 1 means that the stereochemistry is wrong and the model should be inverted. A value of 0.5 means that the crystal consists of a racemic mixture of the two enantiomers.

Table S27. Fractional Atomic Coordinates (×10[4]) and Equivalent Isotropic Displacement Parameters (Å[2]×10[3]) for **21**. U_{eq} is defined as 1/3 of the trace of the orthogonalised U_{ij}.

Atom	x	y	z	U_{eq}
Si1	8463.7(7)	5742.7(4)	5826.5(3)	23.97(14)
O1	7840.8(19)	4820.6(9)	6148.9(7)	25.7(3)
O2	5907(2)	2593.0(10)	5163.7(6)	25.8(3)
O3	8776.0(18)	1484.5(9)	5633.4(7)	25.8(3)
O4	4696(3)	-1841.4(13)	7450.6(9)	49.6(5)
C1	8334(3)	3232.2(13)	6083.6(10)	24.5(4)
C2	7454(3)	4036.4(13)	5817.5(10)	24.2(4)
C3	5646(3)	3813.0(13)	5868.7(11)	25.0(4)
C4	5559(3)	2825.1(13)	5787.1(9)	22.7(4)
C5	3916(3)	2458.7(14)	5983.8(10)	25.8(4)
C6	3781(3)	1458.9(14)	5969.0(11)	27.4(5)
C7	4893(3)	988.2(14)	6427.7(10)	24.5(4)
C8	6609(3)	809.3(14)	6161.1(9)	22.9(4)
C9	7573(2)	1605.2(14)	5951.9(9)	21.2(4)
C10	7056(2)	2506.1(14)	6156.6(9)	21.3(4)
C11	7726(3)	286.1(15)	6596.9(11)	29.6(5)
C12	6941(3)	-556.6(16)	6839.4(12)	33.5(5)
C13	5285(3)	-349.1(16)	7111.2(11)	31.6(5)
C14	4217(3)	105.3(15)	6631.6(10)	28.3(5)
C15	2493(3)	51.0(19)	6900.5(12)	38.0(6)
C16	2460(4)	-866(2)	7190.1(14)	47.8(7)
C17	4230(4)	-1129.5(18)	7277.7(11)	39.4(6)
C18	5444(3)	183.0(18)	7710.3(11)	37.3(6)
C19	10183(3)	5484.8(14)	5286.1(11)	27.8(5)
C20	11507(3)	4897.4(16)	5560.3(12)	32.7(5)
C21	10937(3)	6304.3(17)	4996.1(13)	38.6(6)
C22	8983(3)	6452.2(15)	6502.8(11)	31.3(5)
C23	10394(4)	6111.3(18)	6889.7(13)	43.5(7)
C24	9249(4)	7420.7(16)	6350.4(15)	45.3(7)
C25	6772(3)	6299.3(16)	5383.8(11)	31.7(5)
C26	6261(3)	5813.3(18)	4799.0(12)	40.3(6)
C27	5294(3)	6474.5(17)	5794.3(13)	38.2(6)

Table S28. Anisotropic Displacement Parameters (×10[4]) for **21**. The anisotropic displacement factor exponent takes the form: $-2p^2[h^2a^{*2} \times U_{11} + ... +2hka^* \times b^* \times U_{12}]$.

Atom	U_{11}	U_{22}	U_{33}	U_{23}	U_{13}	U_{12}
Si1	24.5(3)	18.0(3)	29.4(3)	1.0(2)	-2.3(2)	0.7(2)
O1	33.6(8)	17.5(7)	25.9(8)	-1.8(6)	-0.1(6)	-0.1(6)
O2	31.6(8)	26.2(8)	19.7(7)	-2.0(6)	-3.8(6)	6.0(6)
O3	26.9(8)	21.8(8)	28.7(8)	2.2(6)	6.1(6)	1.8(6)
O4	68.9(14)	36.4(11)	43.5(11)	13.5(9)	-0.4(10)	-15.9(10)
C1	24.5(9)	18.7(10)	30.3(11)	-2.4(8)	0.7(9)	-0.5(9)
C2	29.3(10)	19.3(10)	24.0(10)	-2.3(8)	1.2(9)	0.0(8)
C3	26.6(10)	21.9(10)	26.5(10)	-1.9(8)	-1.1(9)	3.4(8)
C4	24.9(10)	23.2(10)	20.2(10)	-1.3(8)	-0.5(8)	3.3(8)
C5	22.5(10)	26.7(11)	28.2(11)	-2.4(8)	-2.4(8)	0.6(8)
C6	23.5(10)	28.2(11)	30.3(11)	-1.1(9)	-2.9(8)	-1.3(8)
C7	24.3(10)	26.0(11)	23.2(10)	0.5(8)	0.8(8)	-3.7(8)
C8	26.6(9)	20.6(9)	21.5(9)	2.8(8)	0.4(8)	-2.5(9)
C9	20.9(9)	23.9(10)	18.8(9)	1.5(8)	-2.0(8)	0.7(8)
C10	21.9(10)	21.9(10)	20.0(9)	-0.5(8)	-0.6(8)	0.1(8)
C11	27.3(11)	29.8(12)	31.5(12)	8.2(9)	-0.1(9)	1.4(9)
C12	38.3(13)	28.3(12)	33.8(12)	10.4(10)	-0.3(10)	1.4(10)

Atom	U_{11}	U_{22}	U_{33}	U_{23}	U_{13}	U_{12}
C13	38.1(13)	30.9(12)	25.9(11)	5.0(9)	0.0(10)	-7.2(10)
C14	28.0(11)	30.6(12)	26.3(11)	1.0(9)	0.9(9)	-7.7(9)
C15	30.1(12)	48.4(15)	35.6(13)	3.2(11)	4.2(10)	-12.5(11)
C16	43.2(15)	55.2(19)	44.9(15)	10.1(13)	2.9(12)	-21.2(14)
C17	53.6(16)	36.9(14)	27.6(12)	5.9(10)	0.6(11)	-15.3(12)
C18	41.1(13)	44.3(15)	26.6(12)	7.4(10)	-1.9(10)	-13.4(12)
C19	26.7(11)	25.2(11)	31.6(12)	1.1(9)	-1.0(9)	0.0(9)
C20	25.6(10)	33.0(12)	39.6(13)	2.8(10)	-0.8(10)	3.0(10)
C21	34.4(13)	35.1(13)	46.2(15)	9.6(11)	4.6(12)	-2.0(10)
C22	32.7(12)	24.5(11)	36.5(12)	-3.7(9)	-3.3(10)	0.8(10)
C23	48.4(15)	39.3(15)	42.8(15)	-10.4(12)	-16.9(13)	6.1(12)
C24	52.8(17)	23.7(12)	59.4(18)	-7.9(12)	-9.3(14)	-3.2(12)
C25	28.8(11)	26.2(11)	40.0(13)	9.6(10)	-2.2(10)	1.0(9)
C26	37.2(13)	46.1(15)	37.6(13)	11.2(12)	-10.0(11)	1.5(12)
C27	28.4(11)	35.5(13)	50.5(15)	4.2(12)	-3.2(11)	2.1(10)

Table S29. Bond Lengths in Å for **21**.

Atom	Atom	Length/Å
Si1	O1	1.6550(16)
Si1	C19	1.885(2)
Si1	C22	1.883(2)
Si1	C25	1.897(2)
O1	C2	1.435(2)
O2	C4	1.439(2)
O3	C9	1.225(3)
O4	C17	1.212(3)
C1	C2	1.539(3)
C1	C10	1.535(3)
C2	C3	1.530(3)
C3	C4	1.519(3)
C4	C5	1.524(3)
C4	C10	1.552(3)
C5	C6	1.529(3)
C6	C7	1.537(3)
C7	C8	1.552(3)
C7	C14	1.524(3)

Atom	Atom	Length/Å
C8	C9	1.521(3)
C8	C11	1.547(3)
C9	C10	1.507(3)
C11	C12	1.533(3)
C12	C13	1.520(3)
C13	C14	1.534(3)
C13	C17	1.517(3)
C13	C18	1.548(3)
C14	C15	1.538(3)
C15	C16	1.536(4)
C16	C17	1.522(4)
C19	C20	1.533(3)
C19	C21	1.533(3)
C22	C23	1.528(3)
C22	C24	1.530(3)
C25	C26	1.538(4)
C25	C27	1.535(3)

Table S30. Bond Angles in ° for **21**.

Atom	Atom	Atom	Angle/°
O1	Si1	C19	108.83(9)
O1	Si1	C22	102.89(10)
O1	Si1	C25	111.80(10)
C19	Si1	C25	108.84(11)
C22	Si1	C19	116.32(11)
C22	Si1	C25	108.12(11)
C2	O1	Si1	124.14(14)
C10	C1	C2	107.01(17)
O1	C2	C1	111.64(17)
O1	C2	C3	111.35(17)
C3	C2	C1	104.59(17)
C4	C3	C2	104.97(17)
O2	C4	C3	110.26(17)
O2	C4	C5	110.76(17)
O2	C4	C10	105.05(16)

Atom	Atom	Atom	Angle/°
C3	C4	C5	111.86(18)
C3	C4	C10	102.26(17)
C5	C4	C10	116.16(17)
C4	C5	C6	115.10(18)
C5	C6	C7	114.11(18)
C6	C7	C8	112.20(17)
C14	C7	C6	112.80(18)
C14	C7	C8	106.65(18)
C9	C8	C7	116.57(17)
C9	C8	C11	106.74(17)
C11	C8	C7	113.52(17)
O3	C9	C8	118.23(18)
O3	C9	C10	122.33(18)
C10	C9	C8	119.42(17)
C1	C10	C4	105.23(16)

Atom	Atom	Atom	Angle/˚
C9	C10	C1	115.69(17)
C9	C10	C4	110.78(16)
C12	C11	C8	113.32(19)
C13	C12	C11	109.79(19)
C12	C13	C14	109.81(19)
C12	C13	C18	111.4(2)
C14	C13	C18	113.1(2)
C17	C13	C12	116.3(2)
C17	C13	C14	101.1(2)
C17	C13	C18	104.8(2)
C7	C14	C13	113.03(18)
C7	C14	C15	119.7(2)
C13	C14	C15	103.96(19)
C16	C15	C14	102.9(2)

Atom	Atom	Atom	Angle/˚
C17	C16	C15	106.0(2)
O4	C17	C13	126.6(3)
O4	C17	C16	125.3(2)
C13	C17	C16	108.0(2)
C20	C19	Si1	114.15(16)
C20	C19	C21	110.6(2)
C21	C19	Si1	113.17(16)
C23	C22	Si1	114.38(17)
C23	C22	C24	109.9(2)
C24	C22	Si1	114.57(19)
C26	C25	Si1	114.22(17)
C27	C25	Si1	111.08(17)
C27	C25	C26	110.8(2)

Table S31. Torsion Angles in ˚ for **21**.

Atom	Atom	Atom	Atom	Angle/˚
Si1	O1	C2	C1	129.22(16)
Si1	O1	C2	C3	-114.29(18)
O1	Si1	C19	C20	-48.20(19)
O1	Si1	C19	C21	-175.81(17)
O1	Si1	C22	C23	64.5(2)
O1	Si1	C22	C24	-167.33(19)
O1	Si1	C25	C26	-69.88(19)
O1	Si1	C25	C27	56.3(2)
O1	C2	C3	C4	-153.17(17)
O2	C4	C5	C6	61.7(2)
O2	C4	C10	C1	82.24(19)
O2	C4	C10	C9	-43.5(2)
O3	C9	C10	C1	-14.8(3)
O3	C9	C10	C4	104.8(2)
C1	C2	C3	C4	-32.4(2)
C2	C1	C10	C4	13.4(2)
C2	C1	C10	C9	136.03(18)
C2	C3	C4	O2	-70.8(2)
C2	C3	C4	C5	165.46(18)
C2	C3	C4	C10	40.5(2)
C3	C4	C5	C6	-174.86(19)
C3	C4	C10	C1	-32.9(2)
C3	C4	C10	C9	-158.66(17)
C4	C5	C6	C7	65.3(2)
C5	C4	C10	C1	-155.01(18)
C5	C4	C10	C9	79.3(2)
C5	C6	C7	C8	-87.5(2)
C5	C6	C7	C14	152.06(19)
C6	C7	C8	C9	58.8(2)
C6	C7	C8	C11	-176.49(18)
C6	C7	C14	C13	-177.43(19)
C6	C7	C14	C15	-54.4(3)
C7	C8	C9	O3	-165.90(18)
C7	C8	C9	C10	16.0(3)
C7	C8	C11	C12	51.5(3)
C7	C14	C15	C16	-166.1(2)
C8	C7	C14	C13	59.0(2)
C8	C7	C14	C15	-178.02(19)

Atom	Atom	Atom	Atom	Angle/˚
C8	C9	C10	C1	163.17(18)
C8	C9	C10	C4	-77.2(2)
C8	C11	C12	C13	-52.0(3)
C9	C8	C11	C12	-178.68(19)
C10	C1	C2	O1	131.81(17)
C10	C1	C2	C3	11.3(2)
C10	C4	C5	C6	-58.0(2)
C11	C8	C9	O3	66.0(2)
C11	C8	C9	C10	-112.1(2)
C11	C12	C13	C14	56.4(3)
C11	C12	C13	C17	170.4(2)
C11	C12	C13	C18	-69.6(2)
C12	C13	C14	C7	-63.2(3)
C12	C13	C14	C15	165.4(2)
C12	C13	C17	O4	31.6(4)
C12	C13	C17	C16	-148.3(2)
C13	C14	C15	C16	-38.7(2)
C14	C7	C8	C9	-177.19(17)
C14	C7	C8	C11	-52.5(2)
C14	C13	C17	O4	150.4(3)
C14	C13	C17	C16	-29.5(3)
C14	C15	C16	C17	19.9(3)
C15	C16	C17	O4	-173.8(3)
C15	C16	C17	C13	6.1(3)
C17	C13	C14	C7	173.4(2)
C17	C13	C14	C15	42.0(2)
C18	C13	C14	C7	61.9(3)
C18	C13	C14	C15	-69.5(2)
C18	C13	C17	O4	-91.9(3)
C18	C13	C17	C16	88.2(3)
C19	Si1	O1	C2	-49.74(18)
C19	Si1	C22	C23	-54.4(2)
C19	Si1	C22	C24	73.8(2)
C19	Si1	C25	C26	50.4(2)
C19	Si1	C25	C27	176.55(17)
C22	Si1	O1	C2	-173.69(16)
C22	Si1	C19	C20	67.37(19)
C22	Si1	C19	C21	-60.2(2)
C22	Si1	C25	C26	177.56(18)
C22	Si1	C25	C27	-56.2(2)
C25	Si1	O1	C2	70.51(18)
C25	Si1	C19	C20	-170.26(17)
C25	Si1	C19	C21	62.1(2)
C25	Si1	C22	C23	-177.13(19)
C25	Si1	C22	C24	-48.9(2)

Table S32. Hydrogen Fractional Atomic Coordinates (×10[4]) and Equivalent Isotropic Displacement Parameters (Å[2]×10[3]) for **21**. U_{eq} is defined as 1/3 of the trace of the orthogonalised U_{ij}.

Atom	x	y	z	U_{eq}
H2	5196.08	2810.02	4933.66	39
H1A	9210.3	3038.17	5804.84	29
H1B	8821.65	3377.94	6484.23	29
H2A	7756.33	4110.39	5378.21	29
H3A	5214.69	3987.17	6272.9	30
H3B	5014.29	4115.94	5546.93	30

Atom	x	y	z	U_{eq}
H5A	3066.33	2707.02	5714.48	31
H5B	3687.34	2661.36	6404.64	31
H6A	4048.31	1252.15	5552.42	33
H6B	2639.64	1292.36	6054.27	33
H7	5014.38	1370.08	6796.06	29
H8	6445.92	433.95	5791.75	27
H10	6741.36	2472.89	6597.16	26
H11A	8739.91	132.45	6378.07	35
H11B	8023.29	664.16	6946.99	35
H12A	6817.29	-984.22	6502.47	40
H12B	7646.93	-821.66	7155.52	40
H14	4224.42	-281.51	6262.89	34
H15A	2314.48	512.64	7211.17	46
H15B	1657.71	106.84	6577.23	46
H16A	1886.5	-851.61	7587.44	57
H16B	1898.45	-1287.24	6918.32	57
H18A	5879.54	766.46	7618.59	56
H18B	6182.03	-122.21	7990.12	56
H18C	4372.45	241.62	7901.42	56
H19	9690.95	5143.09	4942.89	33
H20A	12057.54	5210.79	5891.78	49
H20B	12299.27	4745.27	5243.17	49
H20C	11011.67	4360.66	5721.09	49
H21A	10094.87	6632.81	4777.29	58
H21B	11791.8	6128.91	4709.23	58
H21C	11406.49	6674.91	5316.27	58
H22	8006.62	6434.65	6774.61	38
H23A	10242.41	5484.13	6968.6	65
H23B	10425.47	6429.26	7278.4	65
H23C	11418.64	6201.82	6670.28	65
H24A	10245.25	7484.53	6108.84	68
H24B	9352.15	7757.24	6729.69	68
H24C	8321.3	7640.53	6115.25	68
H25	7196.97	6884.43	5253.69	38
H26A	7190.05	5776.19	4520.15	60
H26B	5372.93	6133.11	4600.28	60
H26C	5893.32	5220.92	4903.89	60
H27A	4839.57	5915.42	5934.11	57
H27B	4467.38	6796.44	5562.67	57
H27C	5630.88	6823.37	6148.1	57

v. X-ray crystallographic data of 22

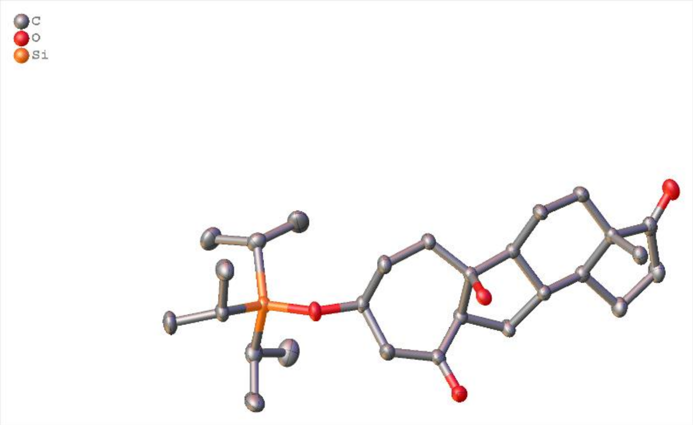

Experimental. Single colourless needle-shaped crystals of **22** were used as supplied. A suitable crystal with dimensions 0.12 × 0.06 × 0.04 mm^3 was selected and mounted on a XtaLAB Synergy, Dualflex, HyPix-Arc 150 diffractometer. The crystal was kept at a steady T = 100.01(10) K during data collection. The structure was solved with the ShelXT 2018/2 (Sheldrick, 2018)[8] solution program using dual methods and by using Olex2 1.5 (Dolomanov et al., 2009)[9] as the graphical interface. The model was refined with ShelXL 2019/3 (Sheldrick, 2015)[10] using full matrix least squares minimization on F^2.

Crystal Data. $C_{27}H_{46}O_4Si$, M_r = 462.73, monoclinic, $P2_1$ (No. 4), a = 12.6411(3) Å, b = 7.83510(10) Å, c = 14.6803(3) Å, β = 113.279(2)°, $\alpha = \gamma$ = 90°, V = 1335.63(5) Å^3, T = 100.01(10) K, Z = 2, Z' = 1, μ(Cu K$_\alpha$) = 0.995, 21534 reflections measured, 5422 unique (R_{int} = 0.0296) which were used in all calculations. The final wR_2 was 0.0982 (all data) and R_1 was 0.0363 (I≥2s(I)).

Compound	22
CCDC	2497586
Formula	$C_{27}H_{46}O_4Si$
$D_{calc.}$/ g cm^{-3}	1.151
μ/mm^{-1}	0.995
Formula Weight	462.73
Colour	colourless
Shape	needle-shaped
Size/mm	0.12×0.06×0.04
T/K	100.01(10)
Crystal System	monoclinic
Flack Parameter	0.000(18)
Hooft Parameter	-0.004(8)
Space Group	$P2_1$
a/Å	12.6411(3)
b/Å	7.83510(10)
c/Å	14.6803(3)
α/°	90
β/°	113.279(2)
γ/°	90
V/Å^3	1335.63(5)
Z	2
Z'	1
Wavelength/Å	1.54184
Radiation type	Cu K$_\alpha$
Θ_{min}/°	3.277
Θ_{max}/°	75.873
Index range h	-15 ≥ h ≥ 15
Index range k	-9 ≥ k ≥ 9
Index range l	-12 ≥ l ≥ 18
Measured Refl's.	21534
Indep't Refl's	5422
Refl's I≥2s(I)	5172
R_{int}	0.0296
Parameters	317
Restraints	2
Largest Peak/eÅ^3	0.256
Deepest Hole/eÅ^3	-0.254
GooF	1.061
R_1 (I≥2s(I) / all)	0.0363 / 0.0382
wR_2 (I≥2s(I) / all)	0.0972 / 0.0982

Structure Quality Indicators for 22

**Reflections:**	d min (CuKα) 2Θ=151.7° 0.79	I/σ(I) 37.7	Rint m=3.97 2.96%	Full 135.4° 99% to 151.7° 99.9	
**Refinement:**	Shift 0.000	Max Peak 0.3	Min Peak -0.2	GooF 1.061	Hooft -.004(8)

A colourless needle-shaped crystal with dimensions 0.12 × 0.06 × 0.04 mm^3 was mounted. Data were collected using a XtaLAB Synergy, Dualflex, HyPix-Arc 150 diffractometer operating at T = 100.01(10) K.

Data were measured using ω scans with Cu K_α radiation. The diffraction pattern was indexed and the total number of runs and images was based on the strategy calculation from the program CrysAlisPro 1.171.44.120a (Rigaku Oxford Diffraction, 2025)[22]. The maximum resolution achieved was Θ = 75.873° (0.79 Å).

The unit cell was refined using CrysAlisPro on 9641 reflections, 45% of the observed reflections.

Data reduction, scaling and absorption corrections were performed using CrysAlisPro. The final completeness is 99.90 % out to 75.873° in Θ. A gaussian absorption correction was performed using CrysAlisPro. The absorption coefficient μ of this material is 0.995 mm^{-1} at this wavelength (λ = 1.54184Å) and the minimum and maximum transmissions are 0.810 and 1.000.

The structure was solved in the space group $P2_1$ (# 4) by ShelXT 2018/2 (Sheldrick, 2018) using dual methods. It was refined by full matrix least squares minimization on F^2 using version 2019/3 of ShelXL 2019/3 (Sheldrick, 2015). All non-hydrogen atoms were refined anisotropically. Hydrogen atom positions were calculated geometrically and refined using the riding model.

_exptl_absorpt_process_details: CrysAlisPro 1.171.44.120a (Rigaku Oxford Diffraction, 2025). Numerical absorption correction based on gaussian integration over a multifaceted crystal model. Empirical absorption correction using spherical harmonics, implemented in SCALE3 ABSPACK scaling algorithm.

There is a single formula unit in the asymmetric unit, which is represented by the reported sum formula. In other words: Z is 2 and Z' is 1. The moiety formula is $C_{27}H_{46}$O4Si.

The Flack parameter was refined to 0.000(18). Determination of absolute structure using Bayesian statistics on Bijvoet differences using the Olex2 results in -0.004(8). The chiral atoms in this structure are: C3(S), C6(R), C8(S), C9(S), C10(S), C13(S), C14(S), C25(S), C25A(R).

Note: The Flack parameter is used to determine chirality of the crystal studied, the value should be near 0, a value of 1 means that the stereochemistry is wrong and the model should be inverted. A value of 0.5 means that the crystal consists of a racemic mixture of the two enantiomers.

Table S33. Fractional Atomic Coordinates (×10[4]) and Equivalent Isotropic Displacement Parameters (Å^2×10^3) for **22**. U_{eq} is defined as 1/3 of the trace of the orthogonalised U_{ij}.

Atom	x	y	z	U_{eq}
Si1	9995.5(5)	2504.0(8)	7634.8(4)	25.05(16)
O1	3555.7(16)	7950(3)	-422.8(12)	34.6(4)
O2	5208.6(13)	5739(2)	4693.9(11)	22.9(3)
O3	6778.3(15)	8544(2)	6008.0(11)	30.0(4)
O4	8687.8(14)	3314(2)	7166.5(11)	26.2(4)
C1	6523(2)	3613(3)	4518.6(16)	24.0(5)
C2	7052(2)	3149(3)	5622.8(17)	25.6(5)
C3	8083.8(19)	4221(3)	6260.2(16)	22.9(4)
C4	7750(2)	5932(3)	6563.7(17)	28.3(5)
C5	7125.2(18)	7241(3)	5773.2(16)	23.1(5)
C6	6977.0(19)	6856(3)	4719.4(16)	21.9(4)
C7	6590(2)	8392(3)	3999.1(16)	26.3(5)
C8	5465.0(19)	7808(3)	3155.8(16)	21.3(4)
C9	5582.1(19)	5858(3)	3174.8(15)	21.0(4)
C10	6044.9(18)	5426(3)	4279.9(15)	20.5(4)
C11	4504(2)	4966(3)	2452.6(16)	25.0(5)
C12	4216(2)	5639(3)	1390.5(16)	27.3(5)
C13	4106.8(19)	7579(3)	1364.9(15)	22.8(4)
C14	5219.5(19)	8417(3)	2113.3(16)	22.9(4)
C15	5071(2)	10320(3)	1844.7(17)	30.1(5)
C16	4547(2)	10260(4)	700.6(18)	33.8(6)
C17	4000(2)	8500(4)	416.4(16)	27.2(5)
C18	3016(2)	8113(3)	1515.1(17)	26.9(5)
C19	9947(2)	1277(3)	8717.0(17)	28.5(5)
C20	9155(3)	-285(4)	8375(2)	40.3(6)
C21	11115(2)	759(4)	9525(2)	39.2(6)
C22	11114(2)	4242(4)	8089(2)	36.2(6)
C23	10947(3)	5322(4)	8891(2)	43.8(7)
C24	11182(3)	5407(5)	7273(3)	49.0(8)
C25	10220(6)	913(9)	6749(5)	30.3(12)
C25A	10431(15)	1460(20)	6669(13)	33(3)
C26	9863(4)	1500(7)	5662(3)	48.1(16)
C26A	9488(9)	332(17)	5938(9)	48(4)
C27	11495(3)	337(4)	7163(2)	43.7(7)

Table S34. Anisotropic Displacement Parameters (×10[4]) for **22**. The anisotropic displacement factor exponent takes the form: $-2p^2[h^2a^{*2} \times U_{11} + ... +2hka^* \times b^* \times U_{12}]$

Atom	U_{11}	U_{22}	U_{33}	U_{23}	U_{13}	U_{12}
Si1	20.3(3)	27.7(3)	22.5(3)	0.8(3)	3.6(2)	5.6(3)
O1	35.3(10)	45.7(11)	20.9(8)	3.2(7)	9.1(7)	8.3(8)
O2	17.6(7)	28.6(9)	21.0(7)	-0.7(6)	6.0(6)	-1.7(6)
O3	27.7(9)	29.3(9)	26.2(8)	-3.8(7)	3.5(7)	6.4(7)
O4	20.5(8)	32.2(9)	21.3(7)	5.9(7)	3.5(6)	6.5(7)
C1	24.2(11)	23.0(11)	19.9(10)	1.4(8)	3.7(8)	0.1(9)
C2	22.2(11)	26.3(11)	23.8(10)	5.1(9)	4.4(9)	1.4(9)
C3	19.4(10)	26.7(11)	20.1(10)	4.4(9)	5.0(8)	4.3(9)
C4	30.2(12)	29.5(13)	20.6(10)	-0.4(9)	5.3(9)	5.5(10)
C5	16.9(9)	26.0(12)	22.0(10)	-1.1(9)	3.0(8)	-0.7(8)
C6	18.2(9)	23.0(10)	21.3(11)	1.2(8)	4.6(8)	-1.0(8)
C7	25.1(11)	24.4(12)	22.0(10)	3.3(9)	1.4(9)	-2.7(9)
C8	20.1(10)	21.5(11)	19.3(10)	1.0(8)	4.7(8)	0.8(8)
C9	19.5(10)	21.6(10)	19.8(10)	0.4(8)	5.6(8)	1.1(8)
C10	17.1(10)	23.0(11)	20.0(10)	0.5(8)	5.6(8)	-0.9(8)

Atom	U_{11}	U_{22}	U_{33}	U_{23}	U_{13}	U_{12}
C11	26.0(11)	23.0(11)	20.1(10)	-0.4(8)	2.7(9)	-1.0(9)
C12	30.1(12)	27.9(12)	19.5(10)	-2.4(9)	5.0(9)	0.9(10)
C13	22.8(10)	27.2(11)	17.2(9)	1.5(10)	6.8(8)	3.2(10)
C14	20.7(10)	24.8(11)	22.8(10)	1.1(9)	8.3(8)	1.5(9)
C15	30.3(12)	27.7(12)	28.4(12)	7.7(10)	7.4(10)	0.0(10)
C16	34.1(13)	36.5(14)	28.5(12)	10.6(11)	9.8(10)	0.6(11)
C17	24.2(11)	35.9(13)	21.4(10)	4.3(10)	8.8(9)	7.0(10)
C18	22.0(11)	32.3(12)	23.4(11)	2.9(9)	5.9(9)	2.8(9)
C19	25.1(12)	29.3(12)	23.9(11)	4.2(9)	2.0(9)	3.5(10)
C20	39.8(15)	34.8(14)	37.5(14)	6.5(11)	6.0(12)	-2.8(12)
C21	32.0(14)	45.1(16)	30.4(13)	9.4(12)	1.5(11)	11.3(12)
C22	23.7(12)	36.2(14)	42.2(15)	4.2(12)	6.1(11)	0.0(11)
C23	42.8(16)	35.4(15)	38.1(15)	-2.3(12)	-0.1(12)	-7.6(12)
C24	36.5(16)	49.4(19)	63(2)	14.0(16)	21.3(14)	0.2(13)
C25	32(3)	30(3)	33(2)	0(2)	17.0(19)	3(2)
C25A	36(7)	29(8)	41(6)	-2(6)	23(5)	-1(5)
C26	49(3)	61(4)	35(2)	-6(2)	17.1(19)	17(2)
C26A	31(5)	55(8)	55(7)	-36(6)	13(5)	-4(5)
C27	36.8(15)	46.0(17)	51.6(17)	-0.3(14)	21.1(13)	14.3(13)

Table S35. Bond Lengths in Å for **22**.

Atom	Atom	Length/Å
Si1	O4	1.6462(17)
Si1	C19	1.879(3)
Si1	C22	1.885(3)
Si1	C25	1.902(7)
Si1	C25A	1.899(17)
O1	C17	1.213(3)
O2	C10	1.433(3)
O3	C5	1.213(3)
O4	C3	1.434(3)
C1	C2	1.533(3)
C1	C10	1.529(3)
C2	C3	1.522(3)
C3	C4	1.524(3)
C4	C5	1.516(3)
C5	C6	1.513(3)
C6	C7	1.548(3)
C6	C10	1.568(3)
C7	C8	1.540(3)
C8	C9	1.534(3)

Atom	Atom	Length/Å
C8	C14	1.514(3)
C9	C10	1.529(3)
C9	C11	1.526(3)
C11	C12	1.547(3)
C12	C13	1.525(4)
C13	C14	1.548(3)
C13	C17	1.526(3)
C13	C18	1.538(3)
C14	C15	1.535(3)
C15	C16	1.544(3)
C16	C17	1.525(4)
C19	C20	1.535(4)
C19	C21	1.539(3)
C22	C23	1.532(4)
C22	C24	1.534(4)
C25	C26	1.546(7)
C25	C27	1.548(7)
C25A	C26A	1.529(19)
C25A	C27	1.527(18)

Table S36. Bond Angles in ° for **22**.

Atom	Atom	Atom	Angle/°
O4	Si1	C19	100.79(10)
O4	Si1	C22	111.01(11)
O4	Si1	C25	111.3(2)
O4	Si1	C25A	113.2(5)
C19	Si1	C22	109.97(12)
C19	Si1	C25	107.9(2)
C19	Si1	C25A	121.8(5)
C22	Si1	C25	114.9(2)
C22	Si1	C25A	100.2(5)

Atom	Atom	Atom	Angle/°
C3	O4	Si1	131.09(15)
C10	C1	C2	115.48(19)
C3	C2	C1	115.41(19)
O4	C3	C2	108.05(19)
O4	C3	C4	105.79(18)
C2	C3	C4	113.3(2)
C5	C4	C3	119.48(19)
O3	C5	C4	119.3(2)
O3	C5	C6	123.1(2)

Atom	Atom	Atom	Angle/°
C6	C5	C4	117.6(2)
C5	C6	C7	114.9(2)
C5	C6	C10	109.42(17)
C7	C6	C10	106.92(17)
C8	C7	C6	105.08(18)
C9	C8	C7	103.26(18)
C14	C8	C7	118.31(19)
C14	C8	C9	108.18(18)
C10	C9	C8	103.49(17)
C11	C9	C8	112.98(19)
C11	C9	C10	119.86(19)
O2	C10	C1	110.63(17)
O2	C10	C6	105.29(17)
O2	C10	C9	112.02(17)
C1	C10	C6	113.88(18)
C9	C10	C1	113.54(18)
C9	C10	C6	100.88(17)
C9	C11	C12	109.5(2)
C13	C12	C11	110.34(19)
C12	C13	C14	110.94(19)
C12	C13	C17	117.9(2)
C12	C13	C18	110.2(2)
C17	C13	C14	100.07(19)

Atom	Atom	Atom	Angle/°
C17	C13	C18	103.95(18)
C18	C13	C14	113.41(19)
C8	C14	C13	110.47(19)
C8	C14	C15	121.4(2)
C15	C14	C13	104.21(19)
C14	C15	C16	101.9(2)
C17	C16	C15	106.2(2)
O1	C17	C13	126.7(2)
O1	C17	C16	125.2(2)
C16	C17	C13	108.1(2)
C20	C19	Si1	111.49(17)
C20	C19	C21	110.2(2)
C21	C19	Si1	116.41(19)
C23	C22	Si1	111.03(19)
C23	C22	C24	109.8(3)
C24	C22	Si1	114.8(2)
C26	C25	Si1	116.7(4)
C26	C25	C27	109.0(5)
C27	C25	Si1	109.6(4)
C26A	C25A	Si1	113.4(11)
C27	C25A	Si1	110.7(10)
C27	C25A	C26A	107.4(11)

Table S37. Torsion Angles in ° for **22**.

Atom	Atom	Atom	Atom	Angle/°
Si1	O4	C3	C2	116.1(2)
Si1	O4	C3	C4	-122.2(2)
O3	C5	C6	C7	12.7(3)
O3	C5	C6	C10	-107.6(3)
O4	Si1	C19	C20	69.8(2)
O4	Si1	C19	C21	-162.6(2)
O4	Si1	C22	C23	59.6(2)
O4	Si1	C22	C24	-65.6(2)
O4	Si1	C25A	C26A	-44.3(13)
O4	Si1	C25A	C27	-165.1(6)
O4	C3	C4	C5	178.0(2)
C1	C2	C3	O4	-161.77(19)
C1	C2	C3	C4	81.4(2)
C2	C1	C10	O2	-56.0(2)
C2	C1	C10	C6	62.4(2)
C2	C1	C10	C9	177.08(19)
C2	C3	C4	C5	-63.8(3)
C3	C4	C5	O3	174.1(2)
C3	C4	C5	C6	-6.6(3)
C4	C5	C6	C7	-166.7(2)
C4	C5	C6	C10	73.1(2)
C5	C6	C7	C8	-122.7(2)
C5	C6	C10	O2	35.5(2)
C5	C6	C10	C1	-85.9(2)
C5	C6	C10	C9	152.10(18)
C6	C7	C8	C9	-25.5(2)
C6	C7	C8	C14	-144.9(2)
C7	C6	C10	O2	-89.6(2)
C7	C6	C10	C1	149.06(19)

Atom	Atom	Atom	Atom	Angle/˚
C7	C6	C10	C9	27.1(2)
C7	C8	C9	C10	43.6(2)
C7	C8	C9	C11	174.77(18)
C7	C8	C14	C13	175.34(19)
C7	C8	C14	C15	-62.3(3)
C8	C9	C10	O2	68.4(2)
C8	C9	C10	C1	-165.39(18)
C8	C9	C10	C6	-43.2(2)
C8	C9	C11	C12	57.2(2)
C8	C14	C15	C16	-166.4(2)
C9	C8	C14	C13	58.5(2)
C9	C8	C14	C15	-179.1(2)
C9	C11	C12	C13	-54.9(3)
C10	C1	C2	C3	-61.3(3)
C10	C6	C7	C8	-1.1(2)
C10	C9	C11	C12	179.55(19)
C11	C9	C10	O2	-58.5(3)
C11	C9	C10	C1	67.7(3)
C11	C9	C10	C6	-170.08(19)
C11	C12	C13	C14	56.7(3)
C11	C12	C13	C17	171.21(19)
C11	C12	C13	C18	-69.7(2)
C12	C13	C14	C8	-59.5(2)
C12	C13	C14	C15	168.55(19)
C12	C13	C17	O1	31.1(3)
C12	C13	C17	C16	-149.3(2)
C13	C14	C15	C16	-41.1(2)
C14	C8	C9	C10	169.81(17)
C14	C8	C9	C11	-59.1(2)
C14	C13	C17	O1	151.4(2)
C14	C13	C17	C16	-29.0(2)
C14	C15	C16	C17	22.3(3)
C15	C16	C17	O1	-175.9(2)
C15	C16	C17	C13	4.5(3)
C17	C13	C14	C8	175.29(18)
C17	C13	C14	C15	43.3(2)
C18	C13	C14	C8	65.2(3)
C18	C13	C14	C15	-66.8(2)
C18	C13	C17	O1	-91.2(3)
C18	C13	C17	C16	88.4(2)
C19	Si1	O4	C3	-171.4(2)
C19	Si1	C22	C23	-51.1(2)
C19	Si1	C22	C24	-176.3(2)
C19	Si1	C25A	C26A	76.1(13)
C19	Si1	C25A	C27	-44.7(11)
C22	Si1	O4	C3	72.1(2)
C22	Si1	C19	C20	-172.9(2)
C22	Si1	C19	C21	-45.4(2)
C22	Si1	C25A	C26A	-162.6(11)
C22	Si1	C25A	C27	76.6(9)
C25	Si1	O4	C3	-57.2(3)
C25	Si1	C19	C20	-46.9(3)
C25	Si1	C19	C21	80.6(3)
C25	Si1	C22	C23	-173.0(3)
C25	Si1	C22	C24	61.8(3)
C25A	Si1	O4	C3	-39.7(6)
C25A	Si1	C19	C20	-56.4(6)

Atom	Atom	Atom	Atom	Angle/˚
C25A	Si1	C19	C21	71.2(6)
C25A	Si1	C22	C23	179.5(6)
C25A	Si1	C22	C24	54.3(6)

Table S38. Hydrogen Fractional Atomic Coordinates (×10[4]) and Equivalent Isotropic Displacement Parameters (Å^2×10^3) for **22**. U_{eq} is defined as 1/3 of the trace of the orthogonalised U_{ij}.

Atom	x	y	z	U_{eq}
H2	4680.58	5009.19	4478.27	34
H1A	7117.78	3457.96	4245.18	29
H1B	5892.16	2799.1	4173	29
H2A	6448.41	3258.68	5890.07	31
H2B	7290.96	1936.96	5686.05	31
H3	8603.66	4408.03	5903.27	28
H4A	7260.88	5692.48	6934.83	34
H4B	8463.95	6473.31	7034.88	34
H6	7726.13	6422.58	4730.79	26
H7A	6453.67	9409.62	4337.56	32
H7B	7181.56	8673.11	3738.53	32
H8	4800.7	8120.45	3329.86	26
H9	6213.54	5592.57	2948.81	25
H11A	3849.34	5188.17	2645.68	30
H11B	4634.46	3717.81	2473.93	30
H12A	4832.01	5299.07	1170.53	33
H12B	3484.05	5127.7	929.19	33
H14	5866.17	7995.05	1944	27
H15A	5820.35	10923.5	2102.26	36
H15B	4543.43	10880.04	2102.19	36
H16A	3959.29	11164.36	426.96	41
H16B	5153.1	10426.33	440.91	41
H18A	2948.15	9359.81	1486.64	40
H18B	3066.31	7710.44	2163.02	40
H18C	2337.97	7609.54	991.27	40
H19	9578.33	2054	9047.03	34
H20A	8419.49	55.67	7852.17	60
H20B	9018.26	-755.03	8937.32	60
H20C	9521.62	-1152.89	8116.27	60
H21A	11492.84	-67.58	9251.92	59
H21B	10992.37	242.39	10083.75	59
H21C	11603.12	1772.32	9754.71	59
H22	11879.43	3666.24	8406.98	43
H23A	10200.48	5902.7	8608.85	66
H23B	11563.73	6173.37	9141.56	66
H23C	10970.78	4582.61	9437.33	66
H24A	11302.49	4712.51	6767.63	74
H24B	11824.58	6206.51	7563.38	74
H24C	10460.64	6047.97	6967.89	74
H25	9748.3	-118.02	6736.1	36
H25A	10623.43	2374.14	6285.91	39
H26A	9047.43	1820.21	5385.7	72
H26B	9986.07	565.62	5270.65	72
H26C	10330.01	2485.85	5640.75	72
H26D	9189.31	-441.61	6304.62	72
H26E	9808.4	-337.49	5542.94	72
H26F	8861.4	1053.25	5496.92	72
H27A	11986.02	1313.47	7177.05	65

Atom	x	y	z	U_{eq}
H27B	11593.63	-558.32	6736.87	65
H27C	11712.42	-107.71	7836.4	65
H27D	12073.09	964.54	7712.23	65
H27E	11815.48	33.62	6675.88	65
H27F	11279.03	-704.22	7417.03	65

Table S39. Atomic Occupancies for all atoms that are not fully occupied in **22**.

Atom	Occupancy	Atom	Occupancy
C25	0.702(10)	H26D	0.298(10)
H25	0.702(10)	H26E	0.298(10)
C25A	0.298(10)	H26F	0.298(10)
H25A	0.298(10)	H27A	0.702(10)
C26	0.702(10)	H27B	0.702(10)
H26A	0.702(10)	H27C	0.702(10)
H26B	0.702(10)	H27D	0.298(10)
H26C	0.702(10)	H27E	0.298(10)
C26A	0.298(10)	H27F	0.298(10)

vi. X-ray crystallographic data of 31

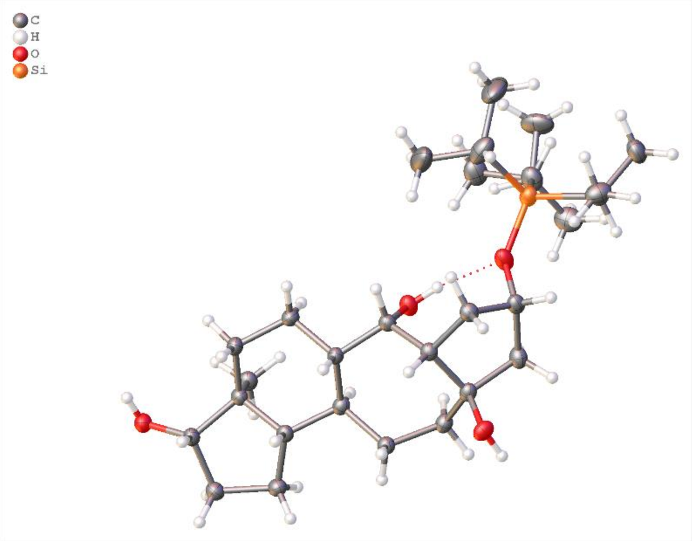

Compound	31
CCDC	2478758
Formula	$C_{27}H_{50}O_4Si$
$D_{calc.}$/ g cm^{-3}	1.194
μ/mm^{-1}	1.024
Formula Weight	466.76
Colour	colourless
Shape	needle-shaped
Size/mm^3	0.13×0.07×0.06
T/K	85
Crystal System	trigonal
Flack Parameter	0.021(12)
Hooft Parameter	0.015(7)
Space Group	$P3_1$
a/Å	17.5256(2)
b/Å	17.5256(2)
c/Å	7.31860(10)
α/°	90
β/°	90
γ/°	120
V/Å^3	1946.72(5)
Z	3
Z'	1
Wavelength/Å	1.54184
Radiation type	Cu K_α
Θ_{min}/°	2.911
Θ_{max}/°	69.001
Measured Refl's.	28229
Indep't Refl's	4719
Refl's I≥2 *s*(I)	4666
R_{int}	0.0342
Parameters	310
Restraints	2
Largest Peak	0.388
Deepest Hole	-0.371
GooF	1.045
wR_2 (all data)	0.0900
wR_2	0.0896
R_1 (all data)	0.0357
R_1	0.0353

Experimental. Single colourless needle-shaped crystals of **31** were used as supplied. A suitable crystal with dimensions 0.13 × 0.07 × 0.06 mm^3 was selected and mounted on a dtrek-CrysAlisPro-abstract goniometer imported rigaku-d*trek images diffractometer. The crystal was kept at a steady *T* = 85 K during data collection. The structure was solved with the ShelXT 2018/2 (Sheldrick, 2018)[8] solution program using dual methods and by using Olex2 1.5-alpha (Dolomanov et al., 2009)[9] as the graphical interface. The model was refined with ShelXL 2019/3 (Sheldrick, 2015)[10] using full matrix least squares minimization on F^2.

Crystal Data. $C_{27}H_{50}O_4Si$, M_r = 466.76, trigonal, $P3_1$ (No. 144), *a* = 17.5256(2) Å, *b* = 17.5256(2) Å, *c* = 7.31860(10) Å, α = 90°, β = 90°, γ = 120°, *V* = 1946.72(5) Å^3, *T* = 85 K, *Z* = 3, *Z'* = 1, μ(Cu K_α) = 1.024, 28229 reflections measured, 4719 unique (R_{int} = 0.0342) which were used in all calculations. The final wR_2 was 0.0900 (all data) and R_1 was 0.0353 (I≥2 *s*(I)).

Structure Quality Indicators for 31

**Reflections:**	d min (CuKα) 2Θ=138.0° 0.83	I/σ(I) 46.5	Rint m=5.99 3.42%	Full 135.4° 100	
**Refinement:**	Shift 0.000	Max Peak 0.4	Min Peak -0.4	GooF 1.045	Hooft .015(7)

A colourless needle-shaped crystal with dimensions 0.13 × 0.07 × 0.06 mm^3 was mounted. Data were collected using a dtrek-CrysAlisPro-abstract goniometer imported rigaku-d*trek images diffractometer operating at T = 85 K.

Data were measured using ω scans with Cu K$_\alpha$ radiation. The diffraction pattern was indexed and the total number of runs and images was based on the strategy calculation from the program DTREK_VERSION=d*TREK version 9.9.9.4 W9RSSI – Aug 22, 2012. The maximum resolution that was achieved was Θ = 69.001° (0.83 Å).

The unit cell was refined using CrysAlisPro 1.171.43.95a (Rigaku OD, 2023)[19] on 19076 reflections, 68% of the observed reflections.

Data reduction, scaling and absorption corrections were performed using CrysAlisPro 1.171.43.95a (Rigaku OD, 2023). The final completeness is 100.00 % out to 69.001° in Θ. A multi-scan absorption correction was performed using CrysAlisPro 1.171.43.95a (Rigaku Oxford Diffraction, 2023). Empirical absorption correction using spherical harmonics, implemented in SCALE3 ABSPACK scaling algorithm. The absorption coefficient μ of this material is 1.024 mm^{-1} at this wavelength (λ = 1.54184 Å) and the minimum and maximum transmissions are 0.905 and 1.000.

The structure was solved and the space group $P3_1$ (# 144) determined by the ShelXT 2018/2 (Sheldrick, 2018) structure solution program using dual methods and refined by full matrix least squares minimization on F^2 using version 2019/3 of ShelXL 2019/3 (Sheldrick, 2015). All non-hydrogen atoms were refined anisotropically. Hydrogen atom positions were calculated geometrically and refined using the riding model.

_exptl_absorpt_process_details: CrysAlisPro 1.171.43.95a (Rigaku Oxford Diffraction, 2023) using spherical harmonics, implemented in SCALE3 ABSPACK scaling algorithm.

There is a single formula unit in the asymmetric unit, which is represented by the reported sum formula. In other words: Z is 3 and Z' is 1. The moiety formula is $C_{27}H_{50}O_4Si$.

The Flack parameter was refined to 0.021(12). Determination of absolute structure using Bayesian statistics on Bijvoet differences using the Olex2 results in 0.015(7). The chiral atoms in this structure are: C2(S), C4(R), C7(S), C8(S), C9(S), C10(S), C13(S), C14(S), C17(S).

Note: The Flack parameter is used to determine chirality of the crystal studied, the value should be near 0, a value of 1 means that the stereochemistry is wrong and the model should be inverted. A value of 0.5 means that the crystal consists of a racemic mixture of the two enantiomers.

Table S40. Fractional Atomic Coordinates (×10^4) and Equivalent Isotropic Displacement Parameters (Å^2×10^3) for **31**. U_{eq} is defined as 1/3 of of the trace of the orthogonalised U_{IJ} tensor.

Atom	*x*	*y*	*z*	U_{eq}
Si1	5480.8(5)	6137.1(5)	8320.4(10)	25.19(18)
O1	566.7(13)	506.1(13)	-171(3)	25.3(4)
O2	4120.8(13)	4207.7(12)	4519(3)	25.0(4)
O3	6104.4(13)	3388.9(13)	4676(3)	26.0(4)
O4	5429.2(13)	5382.9(13)	6864(3)	29.1(4)
C1	5321.8(18)	3961.5(18)	7556(4)	23.2(5)
C2	5908.8(18)	4908.9(18)	6881(4)	24.7(6)
C3	6149.1(19)	4805.0(18)	4909(4)	26.3(6)
C4	5588.3(17)	3820.8(17)	4376(4)	21.8(5)
C5	5279.7(18)	3683.0(18)	2399(4)	23.0(5)
C6	4526.0(17)	2749.6(17)	1931(4)	22.1(5)
C7	3604.9(17)	2632.4(17)	2178(4)	21.0(5)
C8	3343.2(17)	2632.5(17)	4201(3)	20.8(5)
C9	3990.8(17)	3416.2(17)	5371(4)	21.1(5)
C10	4850.3(17)	3414.7(16)	5837(3)	20.1(5)
C11	2419.7(17)	2529.0(18)	4332(4)	23.6(5)
C12	1705.4(18)	1694.6(18)	3365(4)	23.9(6)
C13	1954.8(17)	1663.2(17)	1380(4)	20.9(5)
C14	2884.5(17)	1768.4(17)	1325(4)	20.6(5)
C15	2974.8(18)	1527.3(19)	-650(4)	24.9(6)
C16	2022.6(19)	823.8(19)	-1192(4)	26.5(6)
C17	1437.3(17)	759.6(18)	443(4)	22.6(5)
C18	1883.0(19)	2360.0(19)	228(4)	24.8(6)
C19	6618.3(19)	6745.2(19)	9373(4)	26.4(6)
C20	6720(2)	6265(2)	11022(4)	35.3(7)
C21	6922(2)	7705(2)	9918(5)	38.4(7)
C22	4621(2)	5592(2)	10149(5)	44.0(9)
C23	3734(3)	4850(3)	9545(8)	62.5(13)
C24	4489(3)	6272(4)	11216(7)	68.1(14)
C25	5292(2)	6895(2)	6826(5)	34.5(7)
C26	5989(3)	7278(2)	5331(5)	42.4(8)
C27	4345(3)	6468(3)	6120(7)	41.1(13)
C27A	5064(15)	7483(12)	7640(30)	49(6)

Table S41. Anisotropic Displacement Parameters (Å^2×10^3) for **31**. The Anisotropic displacement factor exponent takes the form: $-2\pi^2[h^2a^{*2}U_{11}+2hka^*b^*U_{12}+\ldots]$.

Atom	U_{11}	U_{22}	U_{33}	U_{23}	U_{13}	U_{12}
Si1	20.6(4)	20.2(4)	34.6(4)	1.2(3)	1.0(3)	10.1(3)
O1	20.3(9)	24.9(10)	28.1(9)	-3.6(8)	-1.3(7)	9.3(8)
O2	23.8(10)	19.3(9)	32.2(10)	-1.2(7)	-5.0(8)	11.1(8)
O3	22.6(10)	31.5(11)	28.3(10)	3.3(8)	0.7(7)	16.7(9)
O4	25.8(10)	21.4(10)	42.3(11)	-4.9(8)	-8.6(8)	13.6(8)
C1	23.9(13)	22.9(13)	24.3(12)	0.0(10)	-2.1(10)	12.7(11)

Atom	U_{11}	U_{22}	U_{33}	U_{23}	U_{13}	U_{12}
C2	21.8(13)	22.1(14)	31.3(15)	-2.1(11)	-4.0(10)	11.9(11)
C3	24.4(14)	21.4(14)	29.3(14)	1.5(10)	-1.7(11)	8.5(11)
C4	19.1(13)	19.0(13)	28.1(13)	1.5(10)	0.3(10)	10.1(11)
C5	19.6(12)	22.5(13)	26.0(13)	2.0(10)	1.0(10)	9.9(11)
C6	21.2(13)	20.7(13)	24.1(12)	-0.3(10)	0.8(10)	10.1(11)
C7	19.4(13)	20.7(13)	24.2(13)	1.5(10)	0.8(10)	11.0(11)
C8	17.5(12)	19.4(12)	24.1(13)	-2.0(10)	0.5(10)	8.3(10)
C9	20.5(13)	18.9(12)	24.4(12)	-1.1(10)	-0.4(10)	10.2(11)
C10	20.3(13)	18.2(12)	22.3(12)	1.2(10)	-0.8(10)	10.0(10)
C11	20.0(13)	24.8(14)	25.1(13)	-3.4(11)	1.0(10)	10.6(11)
C12	18.1(13)	22.6(13)	28.4(14)	-2.5(10)	0.8(10)	8.2(11)
C13	19.9(13)	18.0(12)	23.6(12)	-1.5(10)	0.3(10)	8.5(10)
C14	19.9(12)	16.8(12)	24.9(13)	0.0(10)	0.5(10)	9.1(10)
C15	23.1(14)	24.2(14)	26.5(13)	-1.9(10)	2.5(10)	11.2(12)
C16	25.0(14)	25.0(14)	27.6(14)	-5.4(11)	-0.9(11)	11.1(12)
C17	19.1(13)	20.5(13)	27.3(13)	-3.2(10)	-1.7(10)	9.1(11)
C18	24.0(13)	21.6(13)	30.4(14)	-0.1(10)	-1.4(11)	12.6(11)
C19	25.3(14)	26.6(14)	27.2(13)	-1.8(11)	0.6(11)	12.7(12)
C20	39.1(17)	47.5(19)	27.5(15)	2.9(13)	3.7(13)	27.9(16)
C21	32.7(17)	28.3(16)	48.3(19)	-11.0(14)	-8.1(14)	10.8(13)
C22	32.2(17)	42.0(19)	61(2)	16.3(17)	13.4(15)	20.7(16)
C23	33.9(19)	34.2(19)	109(4)	-9(2)	21(2)	9.3(16)
C24	37(2)	92(3)	58(2)	-20(2)	15.2(18)	19(2)
C25	34.5(16)	24.4(15)	46.1(18)	0.4(13)	-5.9(13)	15.7(13)
C26	56(2)	38.0(18)	37.2(17)	6.1(14)	2.7(15)	26.8(17)
C27	38(2)	36(2)	53(3)	-1.6(19)	-11.5(19)	22(2)
C27A	72(13)	40(10)	52(11)	2(8)	11(9)	42(10)

Table S42. Bond Lengths for **31**.

Atom	Atom	Length/Å	Atom	Atom	Length/Å
Si1	O4	1.665(2)	C8	C9	1.533(4)
Si1	C19	1.892(3)	C8	C11	1.539(4)
Si1	C22	1.880(4)	C9	C10	1.546(4)
Si1	C25	1.873(3)	C11	C12	1.541(4)
O1	C17	1.432(3)	C12	C13	1.526(4)
O2	C9	1.431(3)	C13	C14	1.546(4)
O3	C4	1.457(3)	C13	C17	1.538(4)
O4	C2	1.447(3)	C13	C18	1.540(4)
C1	C2	1.533(4)	C14	C15	1.536(4)
C1	C10	1.547(4)	C15	C16	1.551(4)
C2	C3	1.540(4)	C16	C17	1.543(4)
C3	C4	1.549(4)	C19	C20	1.532(4)
C4	C5	1.521(4)	C19	C21	1.543(4)
C4	C10	1.550(4)	C22	C23	1.511(6)
C5	C6	1.542(4)	C22	C24	1.535(6)

Atom	Atom	Length/Å	Atom	Atom	Length/Å
C6	C7	1.533(4)	C25	C26	1.523(5)
C7	C8	1.550(4)	C25	C27	1.529(5)
C7	C14	1.538(3)	C25	C27A	1.410(15)

Table S43. Bond Angles for **31**.

Atom	Atom	Atom	Angle/˚	Atom	Atom	Atom	Angle/˚
O4	Si1	C19	109.36(11)	C9	C10	C1	113.4(2)
O4	Si1	C22	109.94(15)	C9	C10	C4	115.9(2)
O4	Si1	C25	103.31(13)	C8	C11	C12	113.0(2)
C22	Si1	C19	110.34(16)	C13	C12	C11	111.1(2)
C25	Si1	C19	110.39(14)	C12	C13	C14	108.9(2)
C25	Si1	C22	113.24(16)	C12	C13	C17	115.4(2)
C2	O4	Si1	129.30(18)	C12	C13	C18	110.8(2)
C2	C1	C10	105.7(2)	C17	C13	C14	98.9(2)
O4	C2	C1	110.9(2)	C17	C13	C18	109.4(2)
O4	C2	C3	109.3(2)	C18	C13	C14	113.0(2)
C1	C2	C3	104.4(2)	C7	C14	C13	113.9(2)
C2	C3	C4	108.0(2)	C15	C14	C7	120.4(2)
O3	C4	C3	109.1(2)	C15	C14	C13	103.8(2)
O3	C4	C5	108.9(2)	C14	C15	C16	104.0(2)
O3	C4	C10	104.5(2)	C17	C16	C15	105.8(2)
C3	C4	C10	104.9(2)	O1	C17	C13	116.6(2)
C5	C4	C3	113.2(2)	O1	C17	C16	110.2(2)
C5	C4	C10	115.7(2)	C13	C17	C16	104.0(2)
C4	C5	C6	115.3(2)	C20	C19	Si1	113.0(2)
C7	C6	C5	113.7(2)	C20	C19	C21	108.9(3)
C6	C7	C8	113.9(2)	C21	C19	Si1	114.3(2)
C6	C7	C14	112.0(2)	C23	C22	Si1	116.6(3)
C14	C7	C8	106.9(2)	C23	C22	C24	109.1(3)
C9	C8	C7	116.6(2)	C24	C22	Si1	111.2(3)
C9	C8	C11	109.6(2)	C26	C25	Si1	110.2(2)
C11	C8	C7	110.7(2)	C26	C25	C27	114.1(3)
O2	C9	C8	108.2(2)	C27	C25	Si1	112.5(2)
O2	C9	C10	114.5(2)	C27A	C25	Si1	119.2(8)
C8	C9	C10	114.0(2)	C27A	C25	C26	116.0(9)
C1	C10	C4	102.3(2)				

Table S44. Torsion Angles for **31**.

A	B	C	D	Angle/˚	A	B	C	D	Angle/˚
Si1	O4	C2	C1	103.5(3)	C10	C1	C2	O4	88.0(3)
Si1	O4	C2	C3	-141.9(2)	C10	C1	C2	C3	-29.7(3)
O2	C9	C10	C1	77.0(3)	C10	C4	C5	C6	-43.0(3)
O2	C9	C10	C4	-40.9(3)	C11	C8	C9	O2	-69.4(3)
O3	C4	C5	C6	74.3(3)	C11	C8	C9	C10	161.9(2)
O3	C4	C10	C1	82.0(2)	C11	C12	C13	C14	53.5(3)

A	B	C	D	Angle/˚	A	B	C	D	Angle/˚
O3	C4	C10	C9	-154.1(2)	C11	C12	C13	C17	163.6(2)
O4	Si1	C19	C20	-84.3(2)	C11	C12	C13	C18	-71.4(3)
O4	Si1	C19	C21	150.4(2)	C12	C13	C14	C7	-59.1(3)
O4	Si1	C22	C23	-39.6(3)	C12	C13	C14	C15	168.1(2)
O4	Si1	C22	C24	-165.5(3)	C12	C13	C17	O1	78.4(3)
O4	Si1	C25	C26	-58.6(3)	C12	C13	C17	C16	-160.0(2)
O4	Si1	C25	C27	69.9(3)	C13	C14	C15	C16	-32.3(3)
O4	Si1	C25	C27A	163.7(11)	C14	C7	C8	C9	177.2(2)
O4	C2	C3	C4	-110.0(2)	C14	C7	C8	C11	-56.8(3)
C1	C2	C3	C4	8.7(3)	C14	C13	C17	O1	-165.7(2)
C2	C1	C10	C4	39.0(3)	C14	C13	C17	C16	-44.1(2)
C2	C1	C10	C9	-86.5(3)	C14	C15	C16	C17	4.4(3)
C2	C3	C4	O3	-96.2(2)	C15	C16	C17	O1	150.9(2)
C2	C3	C4	C5	142.4(2)	C15	C16	C17	C13	25.2(3)
C2	C3	C4	C10	15.3(3)	C17	C13	C14	C7	180.0(2)
C3	C4	C5	C6	-164.1(2)	C17	C13	C14	C15	47.3(2)
C3	C4	C10	C1	-32.7(2)	C18	C13	C14	C7	64.4(3)
C3	C4	C10	C9	91.2(3)	C18	C13	C14	C15	-68.3(3)
C4	C5	C6	C7	90.3(3)	C18	C13	C17	O1	-47.3(3)
C5	C4	C10	C1	-158.2(2)	C18	C13	C17	C16	74.3(3)
C5	C4	C10	C9	-34.3(3)	C19	Si1	O4	C2	28.6(3)
C5	C6	C7	C8	-70.6(3)	C19	Si1	C22	C23	-160.3(3)
C5	C6	C7	C14	168.0(2)	C19	Si1	C22	C24	73.7(3)
C6	C7	C8	C9	52.9(3)	C19	Si1	C25	C26	58.3(3)
C6	C7	C8	C11	179.0(2)	C19	Si1	C25	C27	-173.2(3)
C6	C7	C14	C13	-174.5(2)	C19	Si1	C25	C27A	-79.5(11)
C6	C7	C14	C15	-50.3(3)	C22	Si1	O4	C2	-92.7(3)
C7	C8	C9	O2	57.3(3)	C22	Si1	C19	C20	36.8(3)
C7	C8	C9	C10	-71.4(3)	C22	Si1	C19	C21	-88.5(3)
C7	C8	C11	C12	56.4(3)	C22	Si1	C25	C26	-177.5(2)
C7	C14	C15	C16	-161.1(2)	C22	Si1	C25	C27	-48.9(3)
C8	C7	C14	C13	60.1(3)	C22	Si1	C25	C27A	44.8(11)
C8	C7	C14	C15	-175.7(2)	C25	Si1	O4	C2	146.1(2)
C8	C9	C10	C1	-157.6(2)	C25	Si1	C19	C20	162.7(2)
C8	C9	C10	C4	84.6(3)	C25	Si1	C19	C21	37.4(3)
C8	C11	C12	C13	-54.5(3)	C25	Si1	C22	C23	75.4(3)
C9	C8	C11	C12	-173.7(2)	C25	Si1	C22	C24	-50.6(3)

Table S45. Hydrogen Atom Coordinates (Å×10^4) and Isotropic Displacement Parameters (Å^2×10^3) for **31**.

Atom	*x*	*y*	*z*	U_{eq}
H1	226.29	362.25	732.23	38
H2	4476.23	4644.71	5142.33	37
H3	6484.75	3537.16	3847.81	39
H1A	4887.17	3933.15	8458.04	28
H1B	5681.78	3737.46	8136.3	28

Atom	*x*	*y*	*z*	U_{eq}
H2A	6450.76	5223.19	7651.48	30
H3A	6783.99	4997.63	4826.86	32
H3B	6024.53	5171.95	4068.17	32
H5A	5788.83	3819.98	1601.93	28
H5B	5083.6	4109.63	2106.32	28
H6A	4593.27	2615.32	647	27
H6B	4578.63	2318.9	2718.54	27
H7	3593.93	3132.18	1543.26	25
H8	3300.17	2094.43	4774.07	25
H9	3687.3	3365.98	6558.84	25
H10	4701.08	2791.82	6054.03	24
H11A	2435.22	3052.52	3781.64	28
H11B	2259.66	2506.43	5636.37	28
H12A	1628.86	1165.83	4017.23	29
H12B	1137.43	1687.79	3405.78	29
H14	2851.85	1284.5	2105.63	25
H15A	3375.6	1282.91	-716.07	30
H15B	3202.23	2048.86	-1458.55	30
H16A	1837.16	1005.13	-2310.19	32
H16B	1983.67	248.52	-1419.51	32
H17	1400.57	298.6	1301.87	27
H18A	2096.65	2365.94	-1012.79	37
H18B	2240.51	2941.73	786.27	37
H18C	1266.29	2215.72	176.09	37
H19	7039.39	6770.7	8419.9	32
H20A	6357.56	6272.03	12033.28	53
H20B	7339.52	6561.48	11399.6	53
H20C	6528.97	5653.73	10686.78	53
H21A	6916.43	8034.11	8839.42	58
H21B	7520.09	7980.51	10417.34	58
H21C	6520.94	7711.79	10844.36	58
H22	4859.87	5331.81	11039.43	53
H23A	3497.48	5060.61	8578.25	94
H23B	3327.7	4645.43	10586.23	94
H23C	3799.93	4362.27	9077.54	94
H24A	5063.22	6760.73	11590.67	102
H24B	4128.9	5991.98	12301.64	102
H24C	4189.99	6496.16	10436.42	102
H25	5384.6	7400.67	7612.55	41
H25A	4746.02	6481.33	6138.55	41
H26A	6575.31	7596.25	5888.72	64
H26B	5892.72	7683.69	4575.87	64
H26C	5947.69	6799.28	4566.32	64
H27A	4193.64	5914.83	5496	62
H27B	4292.97	6868.3	5261.8	62

Atom	*x*	*y*	*z*	U_{eq}
H27C	3943.11	6346.24	7149.67	62
H27D	4464.41	7152.48	8127.96	73
H27E	5093.5	7904.05	6719.35	73
H27F	5476.78	7803.01	8632.07	73

Table S46. Atomic Occupancy for **31**.

Atom	Occupancy	Atom	Occupancy	Atom	Occupancy
H25	0.799(11)	H25A	0.201(11)	C27	0.799(11)
H27A	0.799(11)	H27B	0.799(11)	H27C	0.799(11)
C27A	0.201(11)	H27D	0.201(11)	H27E	0.201(11)
H27F	0.201(11)				

vii. X-ray crystallographic data of 64

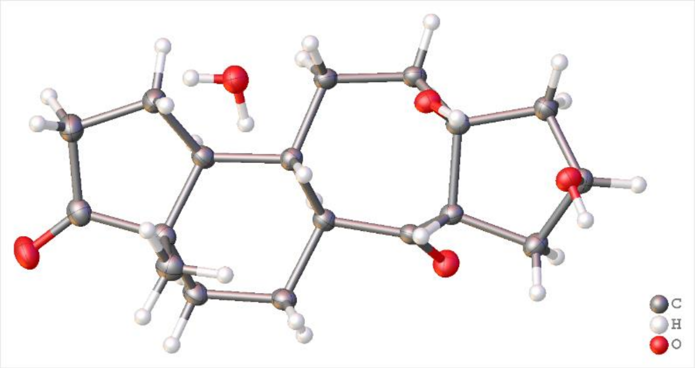

Experimental. Single colourless needle-shaped crystals of **64** were used as supplied. A suitable crystal with dimensions 0.17 × 0.08 × 0.07 mm^3 was selected and mounted on a XtaLAB Synergy, Dualflex, HyPix-Arc 150 diffractometer. The crystal was kept at a steady T = 100.00(10) K during data collection. The structure was solved with the ShelXT 2018/2 (Sheldrick, 2018)[8] solution program using dual methods and by using Olex2 1.5-alpha (Dolomanov et al., 2009)[9] as the graphical interface. The model was refined with ShelXL 2019/3 (Sheldrick, 2015)[10] using full matrix least squares minimization on F^2.

Crystal Data. $C_{18}H_{28}O_5$, M_r = 324.40, trigonal, R3 (No. 146), a = 25.3386(3) Å, b = 25.3386(3) Å, c = 6.86420(10) Å, α = 90°, β = 90°, γ = 120°, V = 3816.68(11) $Å^3$, T = 100.00(10) K, Z = 9, Z' = 1, μ(Cu K_α) = 0.744, 27209 reflections measured, 3450 unique (R_{int} = 0.0222) which were used in all calculations. The final wR_2 was 0.0515 (all data) and R_1 was 0.0194 (I≥2 s(I)).

**Compound**	**64**
CCDC	2480818
Formula	$C_{18}H_{26}O_4 \cdot H_2O$
$D_{calc.}$/ g cm^{-3}	1.270
μ/mm^{-1}	0.744
Formula Weight	324.40
Colour	colourless
Shape	needle-shaped
Size/mm^3	0.17×0.08×0.07
T/K	100.00(10)
Crystal System	trigonal
Flack Parameter	0.00(5)
Hooft Parameter	0.03(2)
Space Group	R3
a/Å	25.3386(3)
b/Å	25.3386(3)
c/Å	6.86420(10)
α/°	90
β/°	90
γ/°	120
V/$Å^3$	3816.68(11)
Z	9
Z'	1
Wavelength/Å	1.54184
Radiation type	Cu K_α
Θ_{min}/°	3.489
Θ_{max}/°	76.632
Measured Refl's.	27209
Indep't Refl's	3450
Refl's I≥2 s(I)	3449
R_{int}	0.0222
Parameters	216
Restraints	1
Largest Peak	0.138
Deepest Hole	-0.089
GooF	1.049
wR_2 (all data)	0.0515
wR_2	0.0514
R_1 (all data)	0.0194
R_1	0.0194

Structure Quality Indicators for 64

**Reflections:**	d min (CuKα) 2Θ=153.3° 0.79	I/σ(I) 75.9	Rint m=7.89 2.22%	Full 135.4° 99% to 153.3° 100	
**Refinement:**	Shift 0.000	Max Peak 0.1	Min Peak -0.1	GooF 1.049	Hooft .03(2)

A colourless needle-shaped crystal with dimensions 0.17 × 0.08 × 0.07 mm^3 was mounted. Data were collected using a XtaLAB Synergy, Dualflex, HyPix-Arc 150 diffractometer operating at T = 100.00(10) K.

Data were measured using ω scans with Cu K_α radiation. The diffraction pattern was indexed and the total number of runs and images was based on the strategy calculation from the program CrysAlisPro system (CCD 44.118a 64-bit (release 22-07-2025)). The maximum resolution that was achieved was Θ = 76.632° (0.79 Å).

The unit cell was refined using CrysAlisPro 1.171.44.118a (Rigaku OD, 2025)[21] on 14313 reflections, 53% of the observed reflections.

Data reduction, scaling and absorption corrections were performed using CrysAlisPro 1.171.44.118a (Rigaku OD, 2025). The final completeness is 100.00 % out to 76.632° in Θ. A gaussian absorption correction was performed using CrysAlisPro 1.171.44.118a (Rigaku Oxford Diffraction, 2025). Numerical absorption correction based on gaussian integration over a multifaceted crystal model. Empirical absorption correction using spherical harmonics, implemented in SCALE3 ABSPACK scaling algorithm. The absorption coefficient μ of this material is 0.744 mm^{-1} at this wavelength (λ = 1.54184 Å) and the minimum and maximum transmissions are 0.820 and 1.000.

The structure was solved and the space group R3 (# 146) determined by the ShelXT 2018/2 (Sheldrick, 2018) structure solution program using dual methods and refined by full matrix least squares minimization on F^2 using version 2019/3 of ShelXL 2019/3 (Sheldrick, 2015). All non-hydrogen atoms were refined anisotropically. Hydrogen atom positions were calculated geometrically and refined using the riding model.

_refine_special_details: Refined as a 2-component twin.

_exptl_absorpt_process_details: CrysAlisPro 1.171.44.118a (Rigaku Oxford Diffraction, 2025). Numerical absorption correction based on gaussian integration over multifaceted crystal model. Empirical absorption correction using spherical harmonics, implemented in SCALE3 ABSPACK scaling algorithm.

There is a single formula unit in the asymmetric unit, which is represented by the reported sum formula. In other words: Z is 9 and Z' is 1. The moiety formula is $C_{18}H_{26}O_4 \cdot H_2O$.

The Flack parameter was refined to 0.00(5). Determination of absolute structure using Bayesian statistics on Bijvoet differences using the Olex2 results in 0.03(2). The chiral atoms in this structure are: C2(S), C4(S), C7(S), C8(S), C10(S), C13(S), C14(S).

Note: The Flack parameter is used to determine chirality of the crystal studied, the value should be near 0, a value of 1 means that the stereochemistry is wrong and the model should be inverted. A value of 0.5 means that the crystal consists of a racemic mixture of the two enantiomers.

Table S47. Fractional Atomic Coordinates (×10[4]) and Equivalent Isotropic Displacement Parameters (Å[2]×10[3]) for **64**. U_{eq} is defined as 1/3 of the trace of the orthogonalised U_{ij}.

Atom	x	y	z	U_{eq}
O1	6906.9(6)	6207.7(6)	9567.5(18)	19.6(3)
O2	5980.1(6)	6046.3(6)	6846.7(18)	18.6(3)
O3	4857.9(6)	4263.8(6)	10499.4(19)	22.6(3)
O4	2587.8(6)	5176.7(7)	8228(2)	30.5(3)
O5	3389.3(6)	3710.3(7)	4680(2)	25.6(3)
C1	6051.0(8)	5259.5(9)	10779(3)	20.7(4)
C2	6617.4(8)	5553.1(8)	9510(3)	18.8(3)
C3	6360.1(8)	5345.6(9)	7479(3)	20.8(4)
C4	5799.5(8)	5427.3(8)	7402(3)	16.9(3)
C5	5334.3(8)	5009.1(8)	5896(3)	17.7(3)
C6	4809.3(8)	5130.7(8)	5516(2)	18.2(3)
C7	4529.9(8)	5243.3(8)	7342(2)	17.1(3)
C8	4381.3(8)	4748.0(8)	8896(3)	17.5(3)
C9	4930.7(8)	4728.0(8)	9711(2)	17.7(3)
C10	5555.3(8)	5296.8(8)	9571(2)	17.1(3)
C11	4030.6(8)	4805.2(9)	10657(3)	19.9(4)
C12	3449.6(8)	4807.0(8)	10043(3)	20.8(4)
C13	3611.1(8)	5319.6(8)	8574(3)	19.5(3)
C14	3936.8(8)	5230.3(8)	6821(3)	18.5(3)
C15	3939.3(9)	5671.0(9)	5277(3)	23.4(4)
C16	3288.5(10)	5572.2(10)	5485(3)	28.3(4)
C17	3083.8(9)	5329.3(8)	7549(3)	22.0(4)
C18	3970.6(9)	5949.9(9)	9554(3)	24.4(4)

Table S48. Anisotropic Displacement Parameters (×10[4]) for **64**. The anisotropic displacement factor exponent takes the form: $-2p^2[h^2a^{*2} \times U_{11} + ... +2hka^* \times b^* \times U_{12}]$.

Atom	U_{11}	U_{22}	U_{33}	U_{23}	U_{13}	U_{12}
O1	19.8(6)	18.4(6)	18.3(6)	-1.6(5)	-2.6(5)	7.8(5)
O2	19.5(6)	16.3(6)	17.0(6)	1.6(5)	0.0(5)	6.7(5)
O3	20.8(6)	20.3(6)	24.9(6)	4.9(5)	1.4(5)	8.9(5)
O4	23.1(7)	33.4(8)	39.1(7)	7.1(6)	7.0(6)	17.3(6)
O5	22.3(7)	28.8(7)	26.1(7)	0.1(6)	0.1(6)	13.1(6)
C1	20.1(8)	21.5(8)	19.3(8)	2.4(7)	-1.4(7)	9.4(7)
C2	18.9(8)	18.0(8)	20.1(8)	-0.5(7)	-1.7(7)	9.7(7)
C3	20.1(8)	24.1(8)	19.4(8)	-1.9(7)	-0.5(7)	12.0(7)
C4	17.3(8)	14.4(7)	16.7(8)	0.6(6)	0.8(6)	6.2(6)
C5	18.5(8)	18.9(8)	15.0(7)	-1.5(6)	0.1(6)	8.7(7)
C6	17.3(8)	21.0(8)	14.6(7)	-0.2(6)	-0.2(6)	8.4(7)
C7	16.8(8)	16.8(8)	16.9(8)	0.6(6)	-0.4(6)	7.9(7)
C8	17.0(8)	18.1(8)	17.3(8)	-0.3(7)	1.0(6)	8.6(7)
C9	19.1(8)	20.5(8)	14.6(7)	0.2(6)	2.6(6)	10.7(7)
C10	18.6(8)	17.3(8)	15.1(7)	1.3(6)	0.6(7)	8.9(7)
C11	22.1(8)	21.7(9)	16.9(8)	2.6(7)	3.7(7)	11.7(7)
C12	19.7(8)	21.7(8)	21.1(8)	3.9(7)	5.1(7)	10.5(7)
C13	19.3(8)	18.9(8)	20.6(8)	1.7(7)	3.6(7)	9.8(7)
C14	16.5(8)	20.2(8)	17.9(8)	-1.0(6)	1.0(7)	8.6(7)
C15	21.8(9)	30.7(10)	20.3(8)	4.4(7)	1.6(7)	15.0(8)
C16	24.4(9)	37.5(11)	27.2(9)	5.4(8)	2.5(7)	18.6(8)
C17	21.5(9)	19.4(8)	26.6(9)	0.4(7)	1.2(7)	11.3(7)
C18	27.2(10)	21.5(9)	24.8(9)	-1.6(7)	1.3(7)	12.4(8)

Table S49. Bond Lengths in Å for **64**.

Atom	Atom	Length/Å
O1	C2	1.440(2)
O2	C4	1.448(2)
O3	C9	1.222(2)
O4	C17	1.208(2)
C1	C2	1.518(3)
C1	C10	1.547(2)
C2	C3	1.518(3)
C3	C4	1.535(2)
C4	C5	1.527(2)
C4	C10	1.582(2)
C5	C6	1.530(2)
C6	C7	1.535(2)
C7	C8	1.544(2)
C7	C14	1.529(2)
C8	C9	1.524(2)
C8	C11	1.549(2)
C9	C10	1.520(2)
C11	C12	1.533(3)
C12	C13	1.529(2)
C13	C14	1.539(2)
C13	C17	1.521(3)
C13	C18	1.542(3)
C14	C15	1.537(2)
C15	C16	1.546(3)
C16	C17	1.529(3)

Table S50. Bond Angles in ° for **64**.

Atom	Atom	Atom	Angle/°
C2	C1	C10	105.07(14)
O1	C2	C1	111.17(14)
O1	C2	C3	107.88(14)
C3	C2	C1	102.74(14)
C2	C3	C4	104.53(15)
O2	C4	C3	110.02(14)
O2	C4	C5	106.83(14)
O2	C4	C10	110.65(14)
C3	C4	C10	103.36(14)
C5	C4	C3	112.02(14)
C5	C4	C10	113.98(14)
C4	C5	C6	115.01(15)
C5	C6	C7	115.23(14)
C6	C7	C8	111.17(14)
C14	C7	C6	110.16(13)
C14	C7	C8	107.85(13)
C7	C8	C11	112.41(14)
C9	C8	C7	115.16(14)
C9	C8	C11	107.04(14)
O3	C9	C8	119.58(16)
O3	C9	C10	121.70(16)
C10	C9	C8	118.70(15)
C1	C10	C4	105.82(13)
C9	C10	C1	113.59(14)
C9	C10	C4	111.77(14)
C12	C11	C8	112.49(15)
C13	C12	C11	109.51(14)
C12	C13	C14	109.03(14)
C12	C13	C18	111.15(15)
C14	C13	C18	114.26(15)
C17	C13	C12	117.03(15)
C17	C13	C14	100.41(14)
C17	C13	C18	104.69(14)
C7	C14	C13	114.05(15)
C7	C14	C15	119.89(14)
C15	C14	C13	103.90(14)
C14	C15	C16	101.65(15)
C17	C16	C15	105.70(15)
O4	C17	C13	126.75(18)
O4	C17	C16	125.12(18)
C13	C17	C16	108.13(15)

Table S51. Torsion Angles in ° for **64**.

Atom	Atom	Atom	Atom	Angle/°
O1	C2	C3	C4	-73.71(17)
O2	C4	C5	C6	-50.69(19)
O2	C4	C10	C1	-108.43(15)
O2	C4	C10	C9	127.44(15)
O3	C9	C10	C1	-9.1(2)
O3	C9	C10	C4	110.58(19)
C1	C2	C3	C4	43.81(17)
C2	C1	C10	C4	17.04(18)
C2	C1	C10	C9	140.03(15)
C2	C3	C4	O2	85.74(17)
C2	C3	C4	C5	-155.59(15)
C2	C3	C4	C10	-32.45(17)

Atom	Atom	Atom	Atom	Angle/°
C3	C4	C5	C6	-171.23(15)
C3	C4	C10	C1	9.32(17)
C3	C4	C10	C9	-114.81(15)
C4	C5	C6	C7	-43.5(2)
C5	C4	C10	C1	131.15(15)
C5	C4	C10	C9	7.0(2)
C5	C6	C7	C8	-49.1(2)
C5	C6	C7	C14	-168.61(14)
C6	C7	C8	C9	63.73(19)
C6	C7	C8	C11	-173.33(14)
C6	C7	C14	C13	178.23(14)
C6	C7	C14	C15	-57.7(2)
C7	C8	C9	O3	-159.39(15)
C7	C8	C9	C10	22.4(2)
C7	C8	C11	C12	54.7(2)
C7	C14	C15	C16	-171.02(16)
C8	C7	C14	C13	56.73(18)
C8	C7	C14	C15	-179.20(16)
C8	C9	C10	C1	169.12(15)
C8	C9	C10	C4	-71.2(2)
C8	C11	C12	C13	-56.2(2)
C9	C8	C11	C12	-177.92(15)
C10	C1	C2	O1	77.94(17)
C10	C1	C2	C3	-37.22(18)
C10	C4	C5	C6	71.9(2)
C11	C8	C9	O3	74.9(2)
C11	C8	C9	C10	-103.38(17)
C11	C12	C13	C14	57.34(19)
C11	C12	C13	C17	170.33(16)
C11	C12	C13	C18	-69.49(18)
C12	C13	C14	C7	-60.43(19)
C12	C13	C14	C15	167.29(15)
C12	C13	C17	O4	34.1(3)
C12	C13	C17	C16	-146.31(16)
C13	C14	C15	C16	-42.22(19)
C14	C7	C8	C9	-175.41(15)
C14	C7	C8	C11	-52.47(18)
C14	C13	C17	O4	151.84(19)
C14	C13	C17	C16	-28.54(18)
C14	C15	C16	C17	23.7(2)
C15	C16	C17	O4	-177.20(18)
C15	C16	C17	C13	3.2(2)
C17	C13	C14	C7	176.06(14)
C17	C13	C14	C15	43.78(17)
C18	C13	C14	C7	64.6(2)
C18	C13	C14	C15	-67.67(19)
C18	C13	C17	O4	-89.5(2)
C18	C13	C17	C16	90.16(18)

Table S52. Hydrogen Fractional Atomic Coordinates (×10[4]) and Equivalent Isotropic Displacement Parameters (Å[2]×10[3]) for **64**. U_{eq} is defined as 1/3 of the trace of the orthogonalised U_{ij}.

Atom	x	y	z	U_{eq}
H1	7032.1	6330.82	10702.06	29
H2	6291.24	6290.41	7479.94	28
H5C	3146.53	3800.17	4428.96	38
H5D	3286.04	3514.85	5782.14	38
H1A	6124.29	5482.87	12024.42	25
H1B	5927.72	4830.14	11065.88	25
H2A	6907.66	5410	9869.77	23
H3A	6242.3	4913.84	7269.59	25
H3B	6661.98	5598.84	6475.35	25
H5A	5161.26	4582.15	6336.67	21
H5B	5549.13	5051.28	4650.51	21
H6A	4483.91	4777.51	4806.05	22
H6B	4958.74	5490.6	4653.17	22
H7	4824.45	5651.24	7898.59	20
H8	4109.66	4344.58	8269.92	21
H10	5522.25	5652.47	10043.76	20
H11A	3920.72	4460.77	11559.1	24
H11B	4300.55	5187.26	11366.31	24
H12A	3158.81	4410.03	9448.38	25
H12B	3253.29	4868.12	11200.65	25
H14	3654.83	4810.73	6318.41	22
H15A	4009.68	5562.17	3955.07	28
H15B	4252.35	6097.78	5565.63	28
H16A	3290.74	5960.74	5299.11	34
H16B	3012.06	5274.28	4509.2	34
H18A	4050.2	6268.67	8598.26	37
H18B	4358.27	6006.74	10050.1	37
H18C	3731.66	5974.9	10635.9	37

5. Flood Lamp Emission Spectra

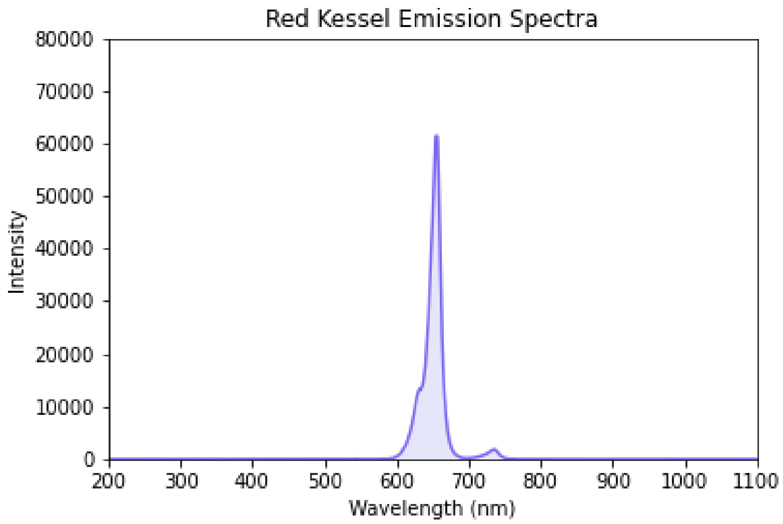

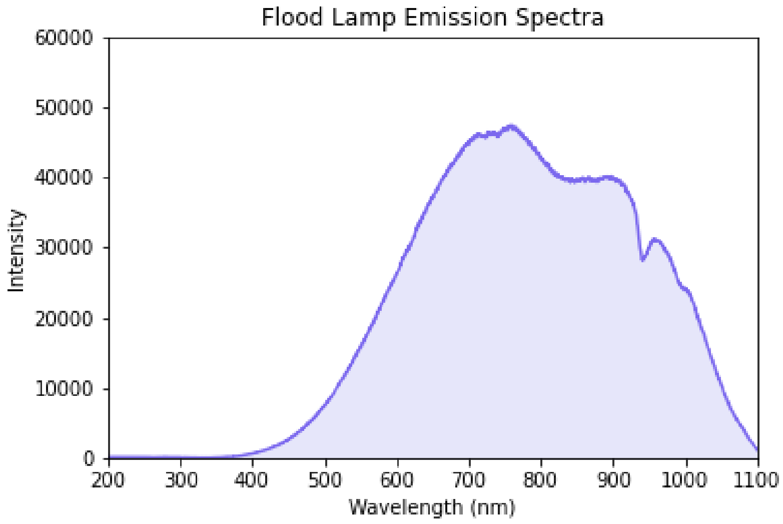

6. Computational Studies

All density functional theory (DFT) calculations were performed using the Q-Chem 5.2 software package.[11] Geometry optimizations for all structures were carried out with the B3LYP[12] functional in conjunction with the 6-311G**[13] basis set. Harmonic vibrational frequency analyses were conducted at the same level of theory (B3LYP/6-311G**).

Single-point energy calculations were subsequently performed at the ωB97X-D/cc-pVTZ[14,15] level of theory, incorporating the SMD[16] implicit solvation model with tetrahydrofuran (THF) as the solvent. Thermodynamic corrections were computed by scaling the translational entropy by a factor of 0.5 to account for restricted molecular motion in solution. Additionally, all vibrational frequencies below 50 cm^{-1} were replaced by 50 cm^{-1} for all stationary points, in order to reduce the overestimation of vibrational entropy. Reported energies correspond to solvent-phase Gibbs free energies, obtained by adding thermal corrections for enthalpy and entropy (from the B3LYP/6-311G** frequency calculations) to the SMD-solvated single-point energies.

It was observed that standard gas-phase optimizations tend to overestimate intramolecular hydrogen bonding in certain structures. To more accurately represent the experimentally relevant intermolecular hydrogen bonding, all key structures were also optimized in the presence of an explicit THF molecule, forming an intermolecular hydrogen bond with the compound under study.

Conformational sampling was performed using the CREST (Conformer-Rotamer Ensemble Sampling Tool)[17] program to generate an ensemble of possible conformers. The lowest-energy conformer identified through this procedure was subjected to frequency and single-point energy calculations, as described above. Visualization of transition state structures was performed in VMD software package.[18]

Optimized Structures

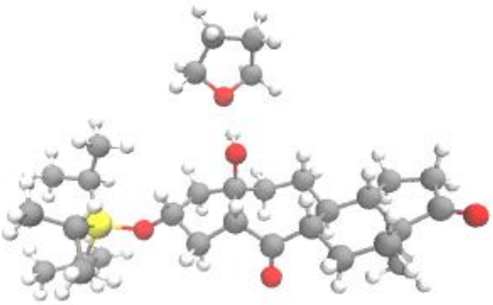

10

11

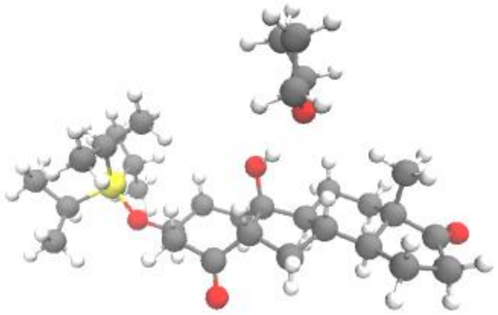

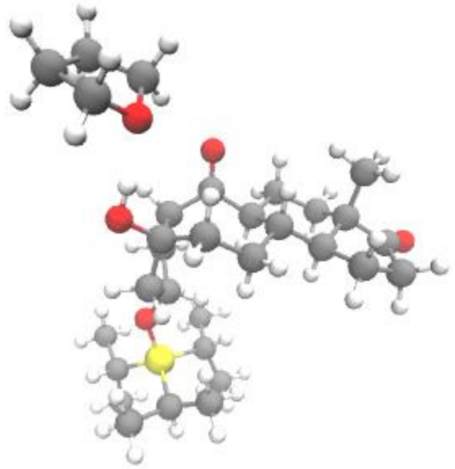

21

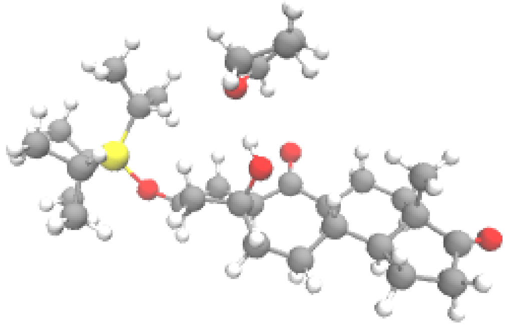

24

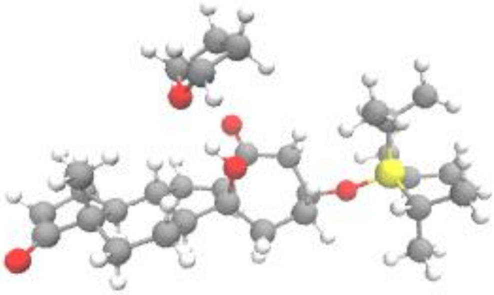

6-*epi*-11

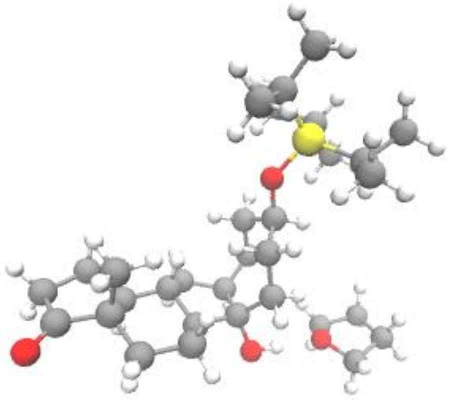

6-10-*epi*-11

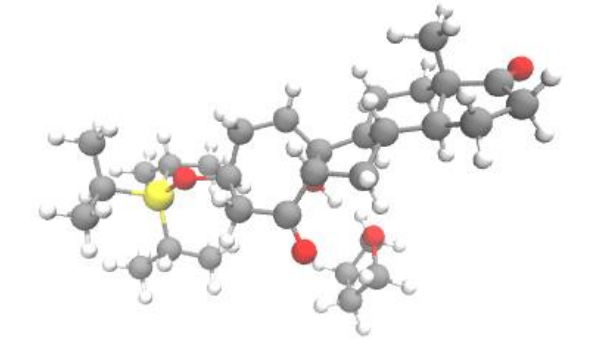

10-*epi*-11

4-*epi*-10

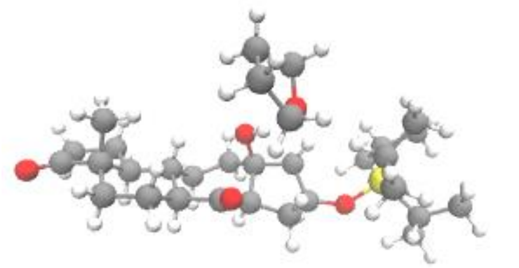

Cartesian Coordinates of the structures

Compound 10 (0 kcal/mol)

C	-0.4395838142	0.9264122926	0.5869638163
C	0.7965777576	1.7967460854	0.3401100752
C	-1.3668999986	-0.5665024236	-1.3966600466
H	1.6051462350	-1.1079761003	-1.1697859945
C	-0.2730880287	-0.3586038369	-0.3237487303
C	1.8797004420	0.7950693659	-0.0655944024
H	-0.4398146160	0.5386308879	1.6096197018
C	-1.7646872032	1.6313088347	0.3715217513
C	1.1087903287	-0.1514111911	-0.9920283442
H	0.6151402768	2.4813617381	-0.4923352499
H	1.0645976427	2.3979048535	1.2116160503
H	0.9980238812	0.3538015189	-1.9573634778
O	-0.2406584583	-1.4629231956	0.5852048874
H	2.2178502168	0.2429107665	0.8204452085
O	2.9777896306	1.4196228545	-0.7120178553
C	-3.9583909384	1.8938645788	1.5688223891
C	-5.0273492968	-0.3887299235	0.0878112411
C	-3.0209850509	0.8950299453	0.8490398405
C	-2.7660297510	-0.9713377969	-0.8975344468
H	-8.1484862244	-1.2354375367	-0.7572017327
O	-1.8463063632	2.7091043600	-0.1800949705
C	-3.6689874711	0.1576313911	-0.3650382989
H	-1.4317498026	0.3334299869	-2.0193911053
H	-1.0002719266	-1.3614965947	-2.0574092117
H	-3.8218657982	0.8893621938	-1.1665802219
H	-2.7166182258	0.1139424762	1.5563296949
H	-2.6482172140	-1.7306214309	-0.1162943378
H	-3.2877331396	-1.4552851786	-1.7294302816
Si	4.6241049761	1.3892253627	-0.3712649362
C	4.8421260529	1.6793825424	1.5081591088
C	5.3170867869	-0.3092201885	-0.9223538980
C	5.2593983095	2.8382201663	-1.4323471619
H	4.1623026606	0.9503432072	1.9726868004
C	6.2460978618	1.3995554357	2.0798775665
C	4.3539034014	3.0822560328	1.9219690479
C	5.0584597108	2.5780494558	-2.9380913439
H	4.5787486249	3.6548538960	-1.1549108504

C	6.6921876306	3.3183440844	-1.1358815579
C	6.8409865040	-0.3636392999	-1.1415177843
C	4.8597636348	-1.4635511610	-0.0087171214
H	4.8432442306	-0.4682342890	-1.9011639677
H	7.3961815438	-0.1781846128	-0.2177567756
H	7.1416039336	-1.3530827161	-1.5055521000
H	7.1744190145	0.3700382100	-1.8788301642
H	5.2782200483	-1.3722865833	0.9980800858
H	3.7717609272	-1.5044220758	0.0892643633
H	5.1887980779	-2.4297213244	-0.4092677632
H	6.5699409254	0.3711026277	1.9041157053
H	7.0045450779	2.0600451888	1.6502033101
H	6.2574064553	1.5623852976	3.1640866551
H	3.3545775673	3.2968541288	1.5362238675
H	4.3201134641	3.1780831767	3.0130636951
H	5.0240337441	3.8626696063	1.5488741538
H	4.0272345923	2.2952342262	-3.1614699310
H	5.2934044230	3.4754459509	-3.5219024660
H	5.7125115196	1.7777333471	-3.2997325561
H	7.4429242393	2.5630936879	-1.3856904430
H	6.9262112223	4.2108896864	-1.7274564942
H	6.8291840316	3.5815098424	-0.0841040578
H	-0.0409397723	-2.2762166523	0.0873273109
H	-5.9938170768	2.1089648782	2.3040867091
C	-5.9168841150	-1.2009591864	-0.8765258749
C	-5.3490416233	1.3257289372	1.8955871154
H	-4.8156286479	-1.0586492473	0.9377919397
H	-3.4720760817	2.2198763516	2.4944195794
H	-4.0444206725	2.7880003876	0.9475058001
C	-5.9721015956	0.7060216556	0.6440104143
H	-5.2666123756	0.5538690598	2.6696557732
C	-6.3426646419	1.7985309979	-0.3940450501
C	-7.2590042509	-0.0932531486	0.8645865287
O	-8.1235862128	0.1492879128	1.6690925703
H	-6.0315259116	-0.6826246134	-1.8332317855
H	-5.5094597270	-2.1902680306	-1.0908789861
H	-5.4706161485	2.3709339609	-0.7112274194
H	-6.8098870756	1.3846842133	-1.2916783022
C	-7.2618329127	-1.2760444145	-0.1200587522
H	-7.0576566835	2.4903335763	0.0580069439
H	-7.3442890663	-2.1936226934	0.4716655967
O	0.4695219864	-3.8935365473	-0.6615117090
C	-0.4946531844	-4.9240797120	-0.9106684954
H	0.8709006408	-5.2037251934	1.9622050379
H	-0.6766327635	-6.8534917642	0.1085998928
C	1.5675692518	-4.5318033937	0.0059224099
H	-1.2486747797	-5.4785359787	1.0523064913
H	-1.4515135176	-4.4412112098	-1.1097670098
H	-0.1953193892	-5.4938374366	-1.8007322954
C	0.9359362359	-5.5815465586	0.9409525164
H	2.2181135341	-5.0042637169	-0.7413242401

H	2.1319330943	-3.7570864981	0.5242648698
C	-0.4823586373	-5.8065289369	0.3480306809
H	1.5262291327	-6.4990663831	0.9627195134

Compound 11 (1.15 kcal/mol)

C	-0.9690921286	-0.9373225876	-1.1982031403
C	-0.0460185880	-2.1344884098	-1.3370547872
H	1.6805268525	-0.8365892271	-1.4903134687
H	1.6017587706	-0.1026583502	1.0642120958
C	1.4429212360	-1.8609100912	-1.1915734120
C	-1.0179639709	-0.3649724413	0.2962356720
H	-0.5379203860	-0.1305942248	-1.7987568803
C	-2.4234272022	-1.1971603230	-1.6333742346
C	-0.2423179856	-1.1865653352	1.3442468414
O	-0.4619763347	0.9457365921	0.3475682140
C	-2.5429459841	-0.3893309035	0.6055245602
C	1.2950141397	-1.1299959989	1.2724976228
H	-0.5469904357	-0.8021512032	2.3219983928
H	-0.5827223691	-2.2269111475	1.3023675633
H	1.6931610354	-1.3925677634	2.2567198154
H	-5.5709461038	0.4120081116	-2.3944169512
O	-0.4575367841	-3.2630103419	-1.5083802705
C	1.9239251626	-2.0888280848	0.2558420423
H	1.9819892859	-2.5589984151	-1.8346715609
O	3.3427629944	-2.0707036291	0.2735601966
H	1.6384936419	-3.1120176870	0.5292215896
C	-3.0933159292	0.5533592194	1.6815917186
H	-2.5576573993	-1.0426419076	-2.7070543946
H	-2.6916798167	-2.2363155926	-1.4191594345
C	-3.2336519848	-0.2321431256	-0.7655335084
H	-2.7639263467	-1.4172300200	0.9299940049
H	-3.0703518607	0.7889850599	-1.1316453564
C	-4.7244334555	-0.4607793878	-0.5767320263
Si	4.6872915500	-1.1199943638	0.5535667103
C	4.7674904354	0.4526743408	-0.5374540688
C	4.7044203939	-0.5856906230	2.3923648493
C	6.1247138378	-2.3211538594	0.1740740665
C	3.7299407065	1.5367355958	-0.1862538526
H	5.7572532736	0.8690496631	-0.2993740983
C	4.7536396974	0.1648375298	-2.0511453444
C	5.8439335771	0.3795669651	2.7746852679
H	3.7622077646	-0.0369092095	2.5227723535
C	4.6702232108	-1.7878011024	3.3563128181
C	5.8386120758	-3.2902503890	-0.9891088908
C	7.4895922696	-1.6302968064	-0.0146124702
H	6.1956478897	-2.9353681147	1.0824422773
H	3.8182820440	-0.3085286811	-2.3633178525
H	4.8548653903	1.0948725869	-2.6226358040
H	5.5692773346	-0.4957649486	-2.3537758375
H	3.9167283536	2.4470650680	-0.7687635311
H	2.7087690326	1.2168674063	-0.4123906813

H	3.7567788373	1.8151878253	0.8706590501
H	5.8069911463	-2.7708879667	-1.9517205561
H	6.6271026453	-4.0487747134	-1.0581255023
H	4.8836153328	-3.8007780159	-0.8576034548
H	7.5081325924	-1.0106001028	-0.9168028875
H	7.7552327663	-0.9890020077	0.8294908926
H	8.2862871898	-2.3751004953	-0.1211915461
H	5.8864644964	1.2534455069	2.1191138868
H	5.7140068710	0.7441576843	3.8000168018
H	6.8206960465	-0.1122868136	2.7347792462
H	5.6047331937	-2.3562996919	3.3154367989
H	4.5399666129	-1.4554111658	4.3926536342
H	3.8566605942	-2.4800806777	3.1244575503
H	-4.8292278143	-1.4491190682	-0.0995530856
H	-4.8755493113	-0.5717934450	2.2124318916
C	-4.6313699660	0.4139866528	1.8005683454
C	-5.3136920584	0.5605154897	0.4333907864
H	-2.6363997134	0.3357316136	2.6521860387
H	-2.8257749421	1.5849187807	1.4424329087
H	-5.0289360115	1.1501905885	2.5048938784
C	-5.7216534960	-0.4586681735	-1.7501614341
C	-5.2456371191	2.0328553590	-0.0566838848
C	-6.7995885949	0.1929518054	0.3716566715
O	-7.6114917486	0.3296716830	1.2525039192
H	-7.5199553709	-1.3872019630	-0.8812620689
H	-7.8469411341	0.2054813195	-1.5352050991
H	-5.7485360995	2.1775004202	-1.0163580302
H	-5.7482134627	2.6714186416	0.6746056051
C	-7.0870758136	-0.3922060602	-1.0256924372
H	-4.2194649640	2.3851093221	-0.1709710186
H	-5.6209798115	-1.3467764028	-2.3769253229
C	-1.0099505580	5.1990541018	-1.9177934432
C	-0.4402668437	5.1954250412	-0.4910017692
H	-2.2623289104	4.6370121279	0.5710058280
H	-0.8572031822	3.6420931030	1.0228918298
C	-1.3723422413	3.7260442004	-2.1253338837
H	0.5984799873	4.8527149920	-0.4912649873
H	-1.9067661711	5.8229659503	-1.9649886432
H	-0.3031502928	5.5632232845	-2.6653077584
O	-1.6807558277	3.2007681216	-0.8141719238
H	-2.2424445772	3.5812058102	-2.7693547068
H	-0.5303148331	3.1596398740	-2.5386652357
C	-1.3395938660	4.1747090049	0.2018421648
H	-0.4749787092	6.1751853787	-0.0111266217
H	-0.9855013252	1.5625730099	-0.1929768723

Compound 21(1.24 kcal/mol)

C	-0.3914752257	1.9164129949	-0.3321491675
H	4.9952429667	1.1843466719	-0.3123006697
C	0.5648240559	1.2163777620	2.0465698974
H	4.5073117629	-3.7663247054	0.7132770308

C	-0.6791972095	1.5931606981	1.2080684054
H	6.2266662936	0.2013417080	-1.1120222339
H	5.9105224674	-0.0026970469	0.6107739133
C	1.0858120710	1.8977035462	-0.6683527915
H	4.5202217017	-0.4254492587	-2.8485660090
C	4.0046830519	-1.7771734143	1.5107640640
C	3.7534219276	-0.5893553258	-2.0862851928
H	2.6033613781	-1.8482595886	-0.0855127646
C	4.9527216833	-2.7699985069	0.8004617699
H	2.3020641231	0.7844349474	-2.9202031834
H	3.4332049507	1.5489448333	-1.8338723621
C	2.8405393985	0.6439104319	-1.9769612275
C	3.2847552239	-1.0890151945	0.3323695241
C	1.8105684125	0.5596404519	-0.8266060260
C	1.3911178478	-0.0242075271	1.6588042435
H	5.9214623959	-2.9037662393	1.2876982516
O	1.7107990777	2.9397398216	-0.7505798893
C	2.4414127039	0.1713226001	0.5497287024
H	1.2240439796	2.0909524725	2.0954088285
H	0.1825443862	1.0781705814	3.0623695474
H	3.0966895823	0.9950962329	0.8595538617
H	1.0804557107	-0.2206318027	-1.0663162210
H	0.7297090656	-0.8606670011	1.3981055877
H	1.9254434988	-0.3467996095	2.5571636741
C	4.3936653352	-0.8949843880	-0.7311831776
H	3.1742104828	-1.4610724683	-2.4129706930
C	5.4378878704	0.1887196190	-0.3554799923
C	5.1453253912	-2.2260496543	-0.6267246465
O	5.7774395838	-2.7617800679	-1.5021404861
H	4.5664124545	-1.0522469634	2.1072109307
H	3.3137867134	-2.2833339300	2.1871917412
C	-1.2864345742	0.9510755172	-1.1442192335
H	-0.7127841093	2.9459084596	-0.4809086015
O	-1.2818993353	2.7236925144	1.8274317468
C	-1.7464469074	0.4800976888	1.1719197518
H	-0.5923527824	3.3950536104	1.9840083097
H	-0.8298467654	0.6029835181	-2.0743153301
H	-1.6431123686	-0.2164446957	2.0068077472
C	-1.6643959079	-0.1995434787	-0.1982690848
H	-2.7257920253	0.9578959649	1.2507184746
H	-2.2141888534	1.4620639292	-1.4128944378
O	-2.8961356544	-0.7944540055	-0.5783108271
H	-0.8722383236	-0.9594453558	-0.2009741051
Si	-3.3272690620	-2.3964838412	-0.8494723023
C	-2.0453857438	-3.2068906180	-2.0172521902
C	-3.3396558118	-3.3835272452	0.7886060814
C	-5.1081663851	-2.2254623202	-1.5035889620
H	-1.0885190713	-3.0549122452	-1.4949510613
C	-2.2260048902	-4.7300223067	-2.1776436972
C	-1.9140106146	-2.5326982940	-3.3962193555
C	-1.9394649726	-3.5413345824	1.4156526131

C	-4.3261937142	-2.8057942405	1.8224921722
H	-3.6887928429	-4.3909361067	0.5235455366
C	-5.2560578223	-1.2428109474	-2.6805513359
C	-5.8039464439	-3.5648836978	-1.8123632569
H	-5.6333304822	-1.7728975444	-0.6503925871
H	-2.2269072605	-5.2559508650	-1.2192402628
H	-3.1635591710	-4.9732598107	-2.6860680543
H	-1.4145456448	-5.1521530531	-2.7812318367
H	-1.5204568282	-2.5724347730	1.7033638175
H	-1.9890781765	-4.1517040502	2.3244477955
H	-1.2281162589	-4.0225374139	0.7385153841
H	-5.3571153860	-2.8153465712	1.4593412179
H	-4.3010233504	-3.3867759778	2.7514643423
H	-4.0771246998	-1.7708645832	2.0754804313
H	-4.8094883103	-1.6384560783	-3.5971622206
H	-6.3149212131	-1.0550691776	-2.8927963235
H	-4.7812994942	-0.2838906768	-2.4638082478
H	-5.3663129115	-4.0522601034	-2.6893023964
H	-5.7466483556	-4.2708437792	-0.9785905151
H	-6.8653598186	-3.4052194075	-2.0323665340
H	-1.7801359147	-1.4507258972	-3.3192810724
H	-1.0542818550	-2.9371704412	-3.9424340691
H	-2.7990405805	-2.7104878077	-4.0138963869
O	0.6137894289	4.7312805163	2.4356045466
C	1.1286662483	5.5929692275	1.4052088119
C	0.1047798740	5.6014049806	3.4526628212
H	1.2140340270	5.0017875032	0.4947514447
H	0.6651271868	7.7183761439	1.1650586423
C	-0.6008531245	6.7356903979	2.6913734829
C	0.1411242373	6.7743789485	1.3262972172
H	-0.5521628672	5.0130276993	4.0936447904
H	0.9404385501	5.9849893260	4.0539811142
H	-0.5605519060	6.6412365719	0.5014997240
H	2.1294786850	5.9355381345	1.6998162597
H	-0.5442902178	7.6830848242	3.2307790665
H	-1.6555345641	6.4947970128	2.5476753343

Compound 24 (1.34 kcal/mol)

C	1.2835215502	-0.8108530541	0.8281382961
C	2.2727791391	-0.6375988920	-0.3320735507
H	1.8538961030	-2.2810552253	-1.7057324678
C	-1.2820755767	-1.0062538205	0.9538964688
C	-0.0733754048	-0.5316479611	0.1787220376
C	1.5358003773	-1.2494534606	-1.5385761035
H	1.3174203147	-1.8431354368	1.1858955629
H	1.5033710144	-0.1599980295	1.6760833881
H	-0.1700884548	0.5418035236	-0.0103116948
O	2.5315391116	0.7434690029	-0.6096076959
H	3.2166569865	-1.1547557901	-0.1394210422
C	0.0090715866	-1.2549656532	-1.2176894143
H	1.7684957056	-0.6803714487	-2.4399155428

O	-0.4999169114	-2.5856787592	-1.1345019020
C	-0.8331851287	-0.5568838037	-2.2841286842
H	0.0156354019	-3.1084254776	-0.4930488309
C	-2.6797893637	-0.5177241900	0.5535079626
H	4.8820674637	0.6867731400	2.6171151381
O	-1.1697205828	-1.7645013679	1.8976880714
H	-2.7060378928	-1.5651097180	-1.9335383679
H	-3.2627097197	-1.4414688252	0.4379918373
C	-3.2621080715	0.2360146494	1.7815110762
C	-2.3449827455	-0.5349130803	-2.0153647238
H	-0.4600125361	0.4657839614	-2.4102838989
H	-0.6606261865	-1.0771800735	-3.2326282596
C	-2.8167903784	0.2700496205	-0.7834622751
H	-2.8166717255	-0.1113681822	-2.9066969905
C	-4.3015593277	0.6604081349	-0.9069888334
H	-2.2220466660	1.1911992867	-0.7269900554
Si	3.6592002777	1.8295621489	-0.0013104902
C	3.9843088468	2.9929737376	-1.4815971355
C	2.9333427973	2.6557518972	1.5679754760
C	5.3070608899	0.9800799809	0.4758006789
C	1.5742303718	3.3447382070	1.3451845053
H	2.7509918495	1.7999541768	2.2350582340
C	3.9042495255	3.5976220082	2.3054784336
C	4.9352558874	4.1658241580	-1.1760639054
H	4.5004420229	2.3382517267	-2.1984794114
C	2.7073503697	3.4914699149	-2.1848977398
C	5.2583977444	0.1253197796	1.7582294903
C	5.9429127034	0.1885219068	-0.6845182256
H	5.9797652906	1.8241185946	0.6890403699
H	4.4740123266	4.8952917190	-0.5039002059
H	5.8721386100	3.8381293370	-0.7161874174
H	5.1944397681	4.6996623181	-2.0974928372
H	2.1475821087	4.1919939945	-1.5584129788
H	2.9612011342	4.0199995192	-3.1112390374
H	2.0409683584	2.6651206115	-2.4404358824
H	1.1294899629	3.6422370328	2.3018703684
H	1.6773948243	4.2527981958	0.7434018427
H	0.8611760272	2.6911373468	0.8362866530
H	4.1016737579	4.5059981660	1.7295992137
H	3.4815709092	3.9131078747	3.2662693031
H	4.8680855437	3.1256009871	2.5141960778
H	6.0727730215	0.8016675795	-1.5796493488
H	6.9307081413	-0.1907463823	-0.3994150340
H	5.3334452039	-0.6750296361	-0.9667455854
H	4.6237427566	-0.7586274915	1.6427343477
H	6.2607363804	-0.2346091744	2.0178734751
H	-5.0307914340	1.3236745594	2.4292746773
C	-6.2334717948	1.8558956221	-0.1879100884
C	-4.7036706212	0.7241888008	1.5749023536
C	-4.0987899004	2.8942527005	0.4038841897
H	-3.2112254875	-0.4372271185	2.6391650076

H	-2.6126704619	1.0860865249	2.0232634921
C	-4.8115864734	1.5222263353	0.2751374395
H	-5.3786317409	-0.1376620906	1.5222762472
C	-4.8357342389	1.3890382142	-2.1590902981
H	-4.8673257999	-0.2840418453	-0.8499211722
H	-4.2111361420	2.2513916542	-2.4116378511
H	-7.0159195534	1.1125189122	-2.0344569971
H	-6.5633144689	2.8042987273	-2.1132493012
H	-3.0427288716	2.7848094502	0.6525029516
H	-4.1607340108	3.4855749828	-0.5127488468
H	-4.5757715498	3.4704093644	1.2008727340
C	-6.2508574210	1.8318107405	-1.7248245639
H	-4.8648030226	0.7448799721	-3.0387803005
O	-7.1783803382	2.0951783922	0.5215953048
O	0.8882632613	-4.3784714496	0.5375998940
C	0.1647629253	-4.8366553655	1.6972404749
C	1.2213610882	-5.5535674956	-0.2101125411
H	2.0899712881	-6.0448599325	0.2498998236
H	-0.7002798153	-6.2081939852	-0.9635220765
C	-0.0285381833	-6.4394523359	-0.1348746005
C	-0.6757780806	-6.0392896335	1.2183319692
H	0.8881583364	-5.1406262557	2.4649003866
H	-0.4167326399	-3.9944165656	2.0667679372
H	0.2184245531	-7.5014004775	-0.1890632722
H	1.4865850026	-5.2417461097	-1.2207424781
H	-1.7201088046	-5.7566458004	1.0798424970
H	-0.6465055907	-6.8529295960	1.9456181582

Compound 6-*epi*-11(1.65 kcal/mol)

C	-0.8842124692	-1.2659320075	-0.9932118971
C	0.0454951947	-0.3020120576	-1.7037672415
C	-5.4259628025	-0.7502610955	0.7585563593
H	-4.7559854109	-2.1763376864	2.2305522120
C	-5.6711663692	-1.2627730714	-1.6171093033
C	-1.0628386712	-0.7791451921	0.5097457906
H	-4.5965314908	-2.4543474752	-0.2265873464
H	-5.4255835976	-1.9412820029	-2.4365935576
H	-7.2796177883	-2.6658400347	-1.0650134653
H	-7.8742992520	-1.0356007972	-1.3107289782
H	-6.1878018073	1.0536443240	-0.2709672981
H	-6.2154394064	1.1335934250	1.4887407797
H	-4.6932605907	1.3350155086	0.6072325009
C	-7.0200717499	-1.6095068002	-0.9432625411
O	-7.6275737992	-1.5931323345	1.4274977857
H	-5.6806778062	-0.2501735934	-2.0295060769
C	-2.3029017421	-1.4127069489	-1.5987415333
H	-0.3861624924	-2.2388032500	-0.9658996108
C	-2.5245766964	-1.1911208804	0.7896872076
H	-2.7243030151	-0.9955587896	2.9447504052
H	-3.1583257313	0.4458900774	2.0496324642
H	-2.5203524417	-2.4719451092	-1.7720368028

H	-2.5255167498	-2.2903530413	0.8626989176
C	-3.2266796954	-0.6446992070	2.0374569058
C	-3.2524761707	-0.8483853884	-0.5298934407
H	-2.3809028467	-0.8960502926	-2.5562106242
C	-4.6754401082	-1.3768999940	-0.4486228636
H	-3.2891954416	0.2404276744	-0.6407764313
H	-5.2269627645	-0.6344126910	2.9182808698
C	-6.8204123887	-1.3528093606	0.5648266640
C	-4.7082728048	-1.0927315029	2.0716724962
C	-5.6342097494	0.7838121704	0.6315837790
C	-0.0337813021	-1.4173795632	1.4441387230
O	-0.8700518283	0.6297460658	0.6349941385
H	-1.5272847203	1.1378764783	0.1258193772
H	2.0462193924	-1.4823723602	1.9470908485
O	3.4279577262	-1.1997398869	-0.1562085934
C	1.5102739318	-0.2702682210	-1.2767013341
C	1.4163154393	-0.9523861034	1.2277920320
H	-0.3127093157	-1.1766541049	2.4749268619
H	-0.0964523507	-2.5068244994	1.3441297338
C	2.0052780946	-1.2182487089	-0.1687751677
O	-0.3364692105	0.4456966033	-2.5813222253
H	2.0924642695	-0.4724100837	-2.1820632551
H	1.7501054253	-2.2481193087	-0.4509874862
H	1.4830567140	0.1136773108	1.4590559941
H	1.7233650284	0.7659812082	-1.0015065973
Si	4.7730045013	-0.3045357634	0.2921756904
C	4.9908878521	-0.4715602855	2.1846471009
C	6.1218894573	-1.2278428453	-0.6951242056
C	4.5842632577	1.5293036677	-0.2396686390
C	6.0933098310	0.4006290299	2.8162864132
H	4.0343903776	-0.1145785146	2.5912068352
C	5.1485854426	-1.9428200066	2.6141374103
C	3.6384964125	2.3337295853	0.6733413645
C	5.9180337758	2.2846547570	-0.4164985542
H	4.1185391777	1.4715512895	-1.2325322088
C	7.5883634298	-0.9630257019	-0.3094494761
H	5.9074983896	-2.2769446191	-0.4477060795
C	5.9137331684	-1.0767701394	-2.2147236419
H	5.9575115709	1.4623188627	2.5976231337
H	6.0903124374	0.2891441961	3.9068272401
H	7.0908373497	0.1181421253	2.4689958419
H	6.1095249459	-2.3528182409	2.2875999246
H	5.1124675626	-2.0362861426	3.7054582307
H	4.3628320771	-2.5778017801	2.1971007118
H	7.7846774340	-1.1822566600	0.7427413314
H	8.2564019671	-1.5985748857	-0.9022506535
H	7.8884930974	0.0729811957	-0.4917926899
H	6.5862913882	-1.7415641756	-2.7687538915
H	4.8899009140	-1.3234019507	-2.5054557439
H	6.1203419046	-0.0560650432	-2.5531095010
H	4.0670298423	2.4691167125	1.6706678633

H	3.4565534817	3.3328309477	0.2611660731
H	2.6668498335	1.8511824433	0.8014125737
H	6.4880685156	2.3400071503	0.5151758321
H	6.5579089288	1.8179082090	-1.1679697469
H	5.7323822209	3.3148602186	-0.7419989722
C	-2.3308222286	3.0244177223	-1.8895756600
C	-1.0620620639	3.8962899819	-2.0087886457
H	-2.2683020196	2.1019251962	-2.4633632153
H	-2.6976022767	4.5611265778	0.3025296112
H	-3.2304417460	3.5805349041	-2.1811306620
C	-0.7020262269	4.2446372298	-0.5404756204
H	-0.2590350048	3.3367391054	-2.4881973084
H	-1.2552418313	4.7905395532	-2.6046763634
C	-1.9297116286	3.7770994594	0.2475546342
H	0.1779518743	3.6847374160	-0.2196826880
H	-0.4988646294	5.3071390298	-0.3933366027
O	-2.4390553400	2.6620062526	-0.4989638216
H	-1.7084778446	3.4250614038	1.2551616585

Compound 6,10-epi-11 (2.55 kcal/mol)

C	-1.8341685078	-0.8074889160	-1.5707125846
C	-0.3804543066	-1.0232136333	-1.9354464285
H	0.2048726481	1.0517249543	-1.5770172103
H	-0.1096358889	0.8844020456	0.9784654714
C	0.6401744493	0.0513774426	-1.5963573269
C	-2.1226541208	-0.9809712569	-0.0018308041
H	4.4825328420	-2.2404980176	-1.0422239655
H	3.6384108847	1.0739145644	-2.8266393604
C	-0.8669213788	-1.1839200617	0.8868532406
H	3.3215613638	-1.5110112811	-2.1525442701
H	5.1154766845	1.8879094666	-2.3116317828
C	0.2673651336	-0.1406543120	0.9271540435
H	-1.2523521899	-1.3123586570	1.9015215584
H	-0.4420343332	-2.1560186407	0.6106616404
H	0.8277944449	-0.2974814002	1.8531715118
H	4.9666002168	-1.8277603676	-2.6864411901
O	-0.0019099351	-2.0658208561	-2.4397018812
C	1.2786782776	-0.2230443909	-0.2215267852
H	1.4197820438	0.0242107750	-2.3591848919
O	2.3057446722	0.7388009766	-0.0034395879
H	1.7010097455	-1.2358867326	-0.2431765301
H	5.2021704502	0.5344777407	-3.4397569133
H	2.7810951235	0.0817235894	2.9974929357
H	3.8259060750	-1.1559518177	3.7042554307
H	4.4588238005	0.4565928188	3.3869228361
H	6.4005122551	-0.5621571976	1.9347842979
H	5.7560109354	-2.1359193838	2.3921318334
H	6.0049941625	-1.7455910379	0.6892303312
Si	3.9617178591	0.5257598267	0.2190731619
C	4.5641292382	2.2890988268	0.6178675999
C	4.2428527090	-0.7799073990	1.5932358106

C	4.8101703614	-0.0840132104	-1.3823508431
C	6.0595365086	2.3543342273	0.9817380727
H	4.4359558046	2.8242486927	-0.3331183703
C	3.7056165927	3.0362633959	1.6560668684
C	3.7967075674	-0.3207274384	2.9950403094
H	3.5883145470	-1.6160803523	1.3045977167
C	5.6810359107	-1.3348133580	1.6480790390
C	4.3673954943	-1.4930548832	-1.8321929573
C	4.6848859634	0.9122217629	-2.5506266773
H	5.8760305350	-0.1514798160	-1.1203329267
H	3.7907532575	2.5924229066	2.6517436205
H	4.0274229232	4.0808888038	1.7383196548
H	2.6501291551	3.0300783370	1.3771343540
H	6.6932520528	1.8692769030	0.2333925169
H	6.3932326574	3.3944734368	1.0661412005
H	6.2588527191	1.8742547916	1.9449090353
C	-2.4047112047	0.5850710812	-1.9732442104
H	-2.3873907531	-1.6062744688	-2.0626354949
C	-2.9901662236	0.2584965416	0.3201155859
O	-2.9651190422	-2.1068898502	0.2151939696
H	-2.4448436129	-2.9267278761	0.1410781800
H	-3.3917720616	0.4434479612	-2.4239262748
H	-3.9892964659	-0.0505186956	-0.0184878231
C	-3.1569431153	0.7255625152	1.7698639722
C	-2.5510202005	1.3643016205	-0.6573034293
H	-1.7892732747	1.1000111014	-2.7156471306
C	-3.5730957612	2.4888899450	-0.5816546124
H	-1.5700767070	1.7563792804	-0.3671014045
H	-4.1793942837	2.3282313176	2.8566157879
C	-4.6870354017	4.1861346440	0.6442157116
C	-4.1196916984	1.9365952616	1.8374816222
C	-2.3950941650	3.7386137970	1.3713076466
H	-3.5575388055	-0.0936810696	2.3741234286
H	-2.1912968767	0.9934659413	2.2089616013
C	-3.6874416223	3.0445317016	0.8657713469
H	-5.1301510382	1.6055995108	1.5717619142
C	-3.4952472735	3.7436899411	-1.4697090552
H	-4.5506320000	2.0287245343	-0.7997715394
H	-3.7376710187	3.5277776123	-2.5123400393
H	-5.5068046889	4.6242313360	-1.2868403889
H	-4.2451145250	5.7426537970	-0.8079910275
H	-2.0555975013	4.5335013438	0.7033420166
H	-2.5910792758	4.1895659049	2.3473712496
H	-1.5715450888	3.0331392693	1.4874314991
C	-4.5257157815	4.6864554929	-0.8055246265
O	-5.4683667216	4.6237721886	1.4509472911
H	-2.4923526271	4.1799070741	-1.4559380898
C	-0.1023933984	-6.1043876145	-1.1086538057
C	-0.1960364988	-6.4326597733	0.4077459585
H	-2.1339837701	-5.5407081338	-1.6988149592
H	-1.2563574456	-5.1603359468	1.8884641985

C	-1.2142047149	-5.0630235005	-1.3343227183
C	-1.3823401119	-5.5818720695	0.8909485335
H	0.8736047250	-5.6825417460	-1.3532943674
H	-0.2481195621	-6.9862382327	-1.7352301435
O	-1.4614612016	-4.4956616519	-0.0368079183
H	-0.3591903301	-7.4947512980	0.5999600093
H	0.7206500930	-6.1391624772	0.9223095642
H	-2.3187106722	-6.1564589060	0.8660266229
H	-0.9360919494	-4.2475018503	-1.9995112597

Compound 10-epi-11 (2.89 kcal/mol)

C	1.0620870837	-0.0729571595	-1.5018219611
C	5.3747386605	-2.9265032291	-0.5002690163
C	5.3855947905	-1.5150389737	0.1426659582
H	4.8486257609	-0.5993407003	2.0223241939
C	5.8360586643	-0.2470740595	-1.9006978049
C	1.0741732384	-0.5716441520	0.0042356762
H	4.8566294288	0.5255992132	-0.1819437811
H	5.7339773776	0.7035099345	-2.4280982044
H	7.5780193271	0.6222152635	-0.8687328958
H	7.9703734198	-0.8792209586	-1.6831216581
H	4.3599368356	-3.3008863278	-0.6394541889
H	5.8721613754	-2.9512701075	-1.4723782643
H	5.9024422624	-3.6218178317	0.1576680704
C	7.1776211957	-0.3602243618	-1.1385695069
O	7.6476422676	-1.3205880573	1.0611325752
H	5.7372837672	-1.0411303341	-2.6465669165
C	2.4946218501	0.3828531873	-1.8181822283
C	4.6578323130	-1.5411300300	1.4949217119
C	2.5954308386	-0.6627194568	0.3281190548
H	2.6317365956	-1.6115143478	2.2769072629
H	2.9088038086	-2.7264581597	0.9523283250
H	2.6691650008	1.3921145340	-1.4348455688
H	2.8316147791	0.3120245750	0.7757075268
C	3.1291672776	-1.7144472305	1.3074438332
C	3.3215479610	-0.6477770083	-1.0362109492
H	2.6978250863	0.3964833615	-2.8915798173
C	4.7995005260	-0.4027951926	-0.7722500720
H	3.2032254894	-1.6208970820	-1.5326811368
H	5.0611007889	-2.3363963280	2.1279867614
C	6.8612067411	-1.1047299868	0.1731521938
H	0.8318594335	-0.9397081010	-2.1316264739
C	-0.0567692104	0.9327618366	-1.6864925788
O	0.4349490861	0.3636655335	0.8809654442
C	0.2709247127	-1.8656371891	0.1594294065
H	0.9428823298	1.1964667968	0.9128068613
H	-1.8419236493	-0.0940556741	0.3615849136
H	-1.6059879117	-0.1348398731	-2.6703071206
O	0.1587656470	2.1203898226	-1.8247391246
C	-1.1008581329	-1.9032320501	-0.5575734009
H	0.8659504576	-2.7087659966	-0.2051351654

H	0.1265282245	-2.0199776826	1.2323271515
C	-1.9147588434	-0.5970809857	-0.6040403193
H	-0.9886446217	-2.2662729995	-1.5858583245
H	-1.7254384651	-2.6447435687	-0.0538045864
C	-1.4770124812	0.3880159631	-1.7124150927
O	-3.2716071123	-0.9474161770	-0.8931825402
H	-2.1493281443	1.2470257344	-1.7188157332
Si	-4.7242529611	-0.6054097055	-0.1203524256
C	-4.7786207997	-1.5229383000	1.5587828083
C	-4.9825844353	1.2898438370	-0.0437753076
C	-6.0043965535	-1.3885339056	-1.2943269690
C	-6.1775060829	-1.5406878606	2.2096163612
H	-4.5471579331	-2.5566044471	1.2658509427
C	-3.7163522034	-1.1077391477	2.5956315712
H	-6.1824768759	-2.1954410611	3.0885636161
H	-6.4772457509	-0.5451818623	2.5513340953
H	-6.9521880299	-1.9021792591	1.5280330316
H	-3.9130957759	-0.1106435577	2.9994061159
H	-3.7200429971	-1.8026746090	3.4435657884
H	-2.7040684589	-1.1029434084	2.1845359344
C	-4.0080195328	2.0658945034	0.8644821183
H	-4.7694849068	1.5997212261	-1.0767517903
C	-6.4343832005	1.7112834758	0.2631805027
H	-4.1287461989	3.1456247206	0.7166361838
H	-4.1965800917	1.8653502172	1.9225586779
H	-2.9612912922	1.8258891552	0.6644881786
H	-6.5429670280	2.7978405386	0.1689476036
H	-7.1585078746	1.2521560935	-0.4148101104
H	-6.7261301390	1.4485204589	1.2850364441
C	-6.0366252185	-0.6968837876	-2.6708848716
C	-5.7739784647	-2.9031009107	-1.4642583478
H	-6.9883930523	-1.2510564913	-0.8259018373
H	-6.3068059517	0.3603040296	-2.5996650265
H	-5.0611237563	-0.7572851947	-3.1623025887
H	-6.7689958169	-1.1774782231	-3.3294497692
H	-4.7832305258	-3.1004591433	-1.8828444378
H	-5.8488188247	-3.4460242372	-0.5176080550
H	-6.5162282886	-3.3337158116	-2.1460779778
O	1.7986094564	2.7718329258	1.3437415747
C	1.4820812762	3.1770721328	2.6819871233
C	1.6560298674	3.9485636294	0.5269471811
H	0.2902419525	4.9047946303	3.3107612740
H	2.5768484280	4.5419695843	0.5997944136
C	0.2865446780	4.1319322060	2.5396445230
H	1.5109153141	3.6177849995	-0.4994977582
H	1.2703557316	2.2757355882	3.2576866581
H	2.3505570710	3.6855307881	3.1223096740
C	0.4494190423	4.7115922485	1.1076980680
H	-0.6508397790	3.5798540975	2.6250042637
H	-0.4429927220	4.5252485777	0.5090137421
H	0.6271077123	5.7887004057	1.1140935238

Compound 4-epi-10 (3.96 kcal/mol)

C	0.3893187669	1.3369105097	0.6519306965
H	4.6213897317	-1.5952216512	-0.0321283820
C	1.1936159299	1.6070477055	-1.7658205699
H	7.4749436353	2.4235593734	-1.2141450282
C	0.2975014810	0.7895791026	-0.8314207285
H	6.3099759110	-1.8255835029	0.4424783484
H	5.9116236045	-1.2738179017	-1.1844350726
C	1.4620978254	0.5740972717	1.4086187090
H	5.9238916066	-0.3736615667	2.4626587472
C	5.6041806131	1.3991863470	-1.7555642567
C	5.3420108527	0.3402903621	1.8728388998
H	4.9903680439	2.2402135614	0.1009856322
C	7.0897239930	1.4057706558	-1.3332415448
H	3.5515722578	0.2392549254	3.0885442719
H	3.6143678675	-0.9743542705	1.8521288806
C	3.8392084799	0.0774128470	2.0469854220
C	4.8607238253	1.2692992495	-0.4066903133
C	2.9310828083	0.9460314352	1.1491813375
C	2.5744102708	2.0095628649	-1.2058140914
H	7.7635705326	0.8969587591	-2.0266710054
O	1.1640457404	-0.2952525372	2.2024776577
C	3.3528964619	0.9648385941	-0.3684085869
H	1.3195377199	1.0131145484	-2.6763891866
H	0.6675337747	2.5232877821	-2.0551344049
H	3.1646927128	-0.0249516413	-0.7908079016
H	3.0164705907	1.9934607858	1.4842656817
H	2.4842195643	2.9291118135	-0.6141983170
H	3.1848546918	2.2872283718	-2.0684703557
C	5.7207754365	0.2634561868	0.3958537886
H	5.5966759371	1.3365859131	2.2529203664
C	5.6292806241	-1.1922848577	-0.1321147829
C	7.1370749035	0.7312530164	0.0472848439
O	8.1209210860	0.6038805236	0.7323545184
H	5.3828306861	0.5494835751	-2.4079767078
H	5.3350542195	2.3043143643	-2.3015273901
H	0.6859460919	2.3895792937	0.5995982004
C	-1.0355037145	1.2505003869	1.1995904269
O	0.7745027702	-0.5530726701	-0.9296612318
H	1.7087877540	-3.8288308190	-0.6820884049
H	-1.2092294181	1.8924012335	2.0656995038
H	-1.3955956853	1.3190656297	-2.1537499626
C	-1.8688942009	1.6687227234	-0.0208248222
C	-1.2173572110	0.8765973906	-1.1719477410
H	-1.6229540831	-0.1374988942	-1.1901104752
H	-1.2749352978	0.2241716085	1.4898310633
O	-3.2628764266	1.5328273350	0.1562540155
H	-1.6969147844	2.7396777916	-0.1954077548
Si	-4.5033378036	0.4599504859	-0.1959366993
C	-5.9410519399	1.2463926095	0.7801398081
C	-4.7538213038	0.3739919726	-2.0963108836

C	-4.0456552793	-1.2647277207	0.5064339183
C	-6.2668568816	2.6866258883	0.3390695707
C	-7.2297036559	0.4068614304	0.8785134242
H	-5.5188048965	1.3141349414	1.7923739789
C	-4.0486794400	-1.2719214603	2.0481989233
C	-4.8465751044	-2.4630537720	-0.0383895192
H	-3.0059884776	-1.4214118650	0.1903774462
C	-4.5013994503	1.7166739823	-2.8127422845
H	-3.9853008978	-0.3314846056	-2.4445380702
C	-6.1241795834	-0.1903163916	-2.5253441849
H	-6.7430851079	2.7071086074	-0.6464723540
H	-6.9650639965	3.1560657888	1.0419789814
H	-5.3694366525	3.3075700772	0.2949062949
H	-7.9410566399	0.8793628816	1.5655842075
H	-7.7311609966	0.3168430541	-0.0888152931
H	-7.0447972474	-0.6045323092	1.2495539295
H	-5.9097561596	-2.3995787433	0.2126798611
H	-4.7663939687	-2.5548987893	-1.1246260979
H	-4.4717257407	-3.3982486323	0.3940167123
H	-3.5374469367	2.1541512867	-2.5485144979
H	-4.5228260586	1.5815751481	-3.9003771081
H	-5.2714619555	2.4525389056	-2.5631177716
H	-6.1568873170	-0.3385064604	-3.6109609102
H	-6.3575667428	-1.1486535189	-2.0568930179
H	-6.9287956340	0.5060556329	-2.2729492006
H	-5.0656339583	-1.2100501098	2.4461648988
H	-3.6075067201	-2.1983070486	2.4335094579
H	-3.4785331586	-0.4376512536	2.4646731572
O	-0.6744633511	-2.6730275426	0.1353184488
C	-0.3575280747	-3.2191151411	1.4343668267
H	0.3749106171	-5.2725571765	1.6149329858
H	-1.2892708589	-4.4044736292	-0.8412577673
C	0.7060976098	-4.3190453268	1.1981881648
C	-0.4386230057	-3.7091581823	-0.8274361771
H	-0.3503714589	-3.2347461455	-1.8051850034
H	-0.0057203449	-2.3923955543	2.0489869600
H	-1.2709648232	-3.6353252138	1.8740160387
C	0.8385332017	-4.3945098484	-0.3424002752
H	1.6564289672	-4.0656898475	1.6694482379
H	0.9323425319	-5.4174665448	-0.7119475626
H	0.2072025139	-1.1641756800	-0.4223071325

DFT calculations were performed to evaluate the relative stabilities of the α- and β-endoperoxides formed during singlet oxygen oxidation of bufogargarizin A and B. For bufogargarizin B, the β-endoperoxide was more stable than the α-isomer by 0.22 kcal/mol, consistent with the experimentally observed β:α ratio of 3.3:1. In contrast, for bufogargarizin A, the α-endoperoxide was favored by 0.73 kcal/mol, matching the experimental α:β ratio of 2:1. These results suggest that the energy differences between the endoperoxide intermediates may reflect similar trends in the transition states, contributing to the observed diastereoselectivity.

To understand the origin of the opposite diastereoselectivity observed in the singlet oxygen oxidation of bufogargarizin A and B, we analyzed the torsional strain in their respective AB-ring systems. This was assessed by comparing the C7–C8–C14–C15 dihedral angles in the diene precursors and corresponding endoperoxides. All geometries were optimized using the B3LYP[12] functional with the 6-311G**[13] basis set.

**bufogargarizin-B-diene-precursor**	**bufogargarizin -A-diene-precursor**
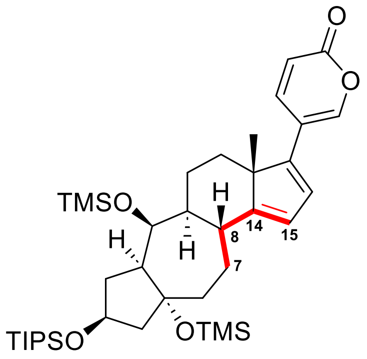	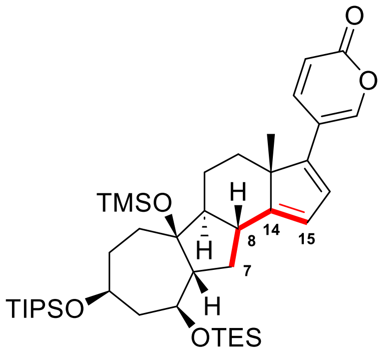
**bufogargarizin -B-endoperoxide**	**bufogargarizin -A-endoperoxide**
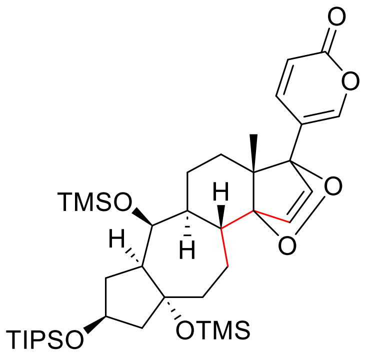	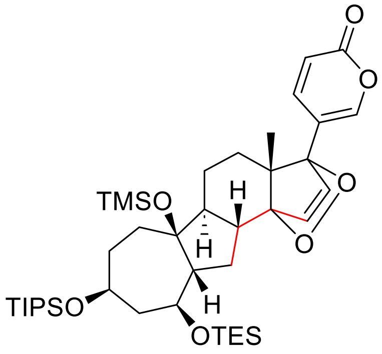

Bufogargarizin B exhibited two low-energy conformers within an energy window of 0.4 kcal/mol, with C7–C8–C14–C15 dihedral angles of 7.80° (diene-precursor-1) and 16.9° (diene-precursor-2), respectively, indicating a slightly puckered conformation. In contrast, bufogargarizin A adopted a nearly planar geometry with a dihedral angle of 0.17°, reflecting the structural differences in their AB-ring systems.

Upon oxidation, the dihedral angles in the bufogargarizin B endoperoxides were found to be −54.12° for the α-isomer and +61.58° for the β-isomer. The transition from the diene conformers (7.80° or 16.9°) to the β-endoperoxide (61.58°) involves a conformational change that proceeds in the same direction as the initial puckering, making it energetically more favorable. In contrast, formation of the α-isomer requires a reversal in puckering direction, resulting in a more substantial and less favorable conformational shift.

These geometric and energetic considerations indicate that torsional strain in the AB-ring system plays a critical role in shaping the transition state landscape, thereby influencing the diastereoselectivity observed in the singlet oxygen oxidation of bufogargarizin A and B.

<table>
<tr><th>bufogargarizin-B-diene-precursor-1</th><th>bufogargarizin -B-diene-precursor-2</th></tr>
<tr><td>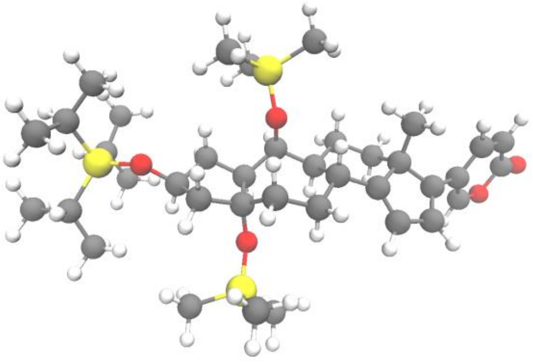</td><td>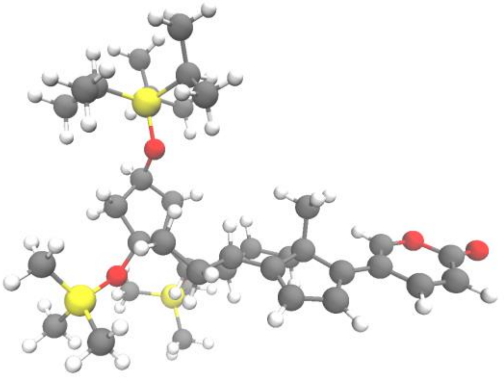</td></tr>
<tr><th colspan="2">bufogargarizin -A-diene-precursor</th></tr>
<tr><td colspan="2">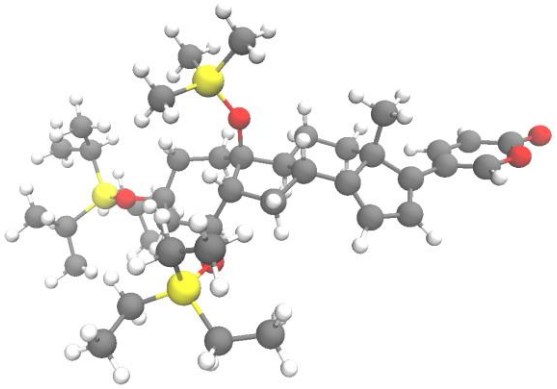</td></tr>
</table>

bufogargarizin -B-β-endoperoxide	bufogargarizin -B-α-endoperoxide
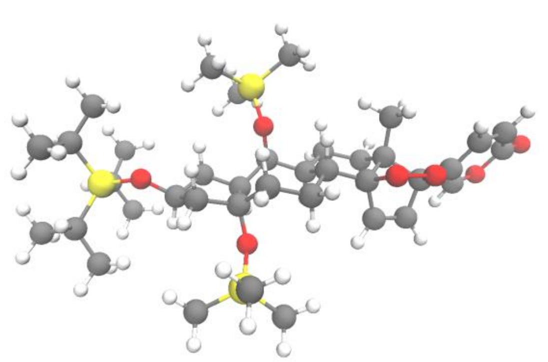	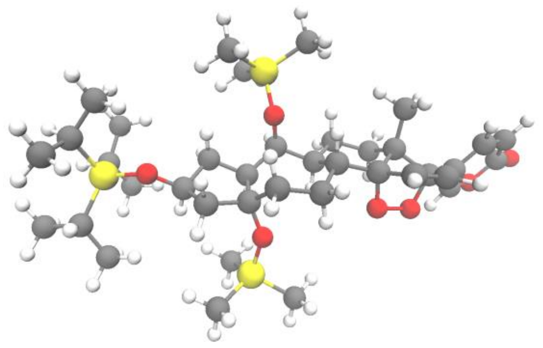
**bufogargarizin -A-β-endoperoxide**	**bufogargarizin -A-α-endoperoxide**
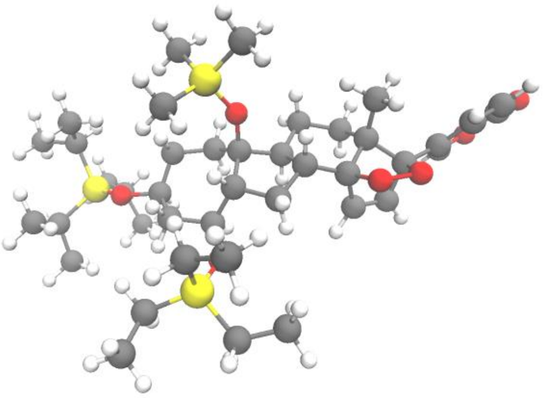	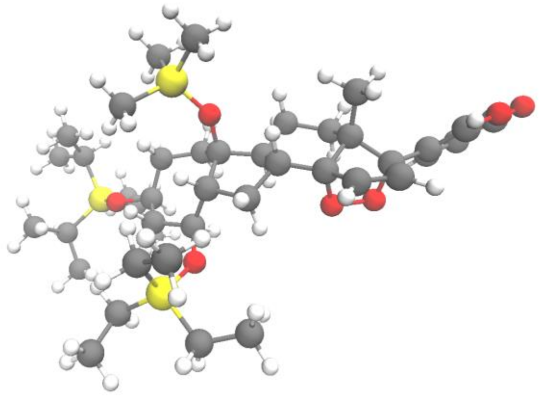

Cartesian Coordinates of the optimized structures (B3LYP, 6-311G**)

bufogargarizin-B-diene-precursor-1

110

C	-4.2964675014	1.8716591041	0.5624420571
C	-4.8854670914	1.4908736868	1.9356047060
C	-2.2352832865	0.5508672771	-0.4162421701
H	-4.1744218106	-1.5247475682	0.6123297198
C	-3.7288685589	0.5321757295	-0.0493724959
C	-5.2036832617	-0.0040548013	1.8445050737
H	-5.1161798532	2.1499377331	-0.1046792369
C	-3.3968734271	3.1206059072	0.6385057812
C	-4.0153478900	-0.5303577168	1.0348386496
H	-4.1341825123	1.6087079616	2.7205396136
H	-5.7525156829	2.0990436405	2.2049163907
H	-3.1650060993	-0.5962373897	1.7215948028
O	-4.5217255126	0.2909103209	-1.2282141251
H	-6.1380872390	-0.1609168048	1.2934222656
O	-5.2709940232	-0.6290883176	3.1193194854
C	-2.6874915844	5.1861755992	-0.6294658866
C	-1.1999927953	3.6752300550	-2.5357470154
C	-2.9199885789	3.6640062402	-0.7354212697

C	-1.8562776876	1.4448040960	-1.6022476519
H	1.2783785338	7.4666400330	-3.3185539809
O	-2.2848236994	2.9301097657	1.5149825726
C	-1.6585325717	2.9395010904	-1.3061050888
H	-1.6707617094	0.8434451630	0.4713298650
H	-1.9455922416	-0.4785118916	-0.6506995636
H	-0.8822270770	3.0304924784	-0.5353818676
H	-3.7400307113	3.5009634065	-1.4467434771
H	-2.6097221732	1.3323902603	-2.3869151480
H	-0.9154816564	1.0726468189	-2.0212323294
Si	-6.5023806651	-1.1572800442	4.1256708778
C	-7.3444831983	-2.7230605111	3.4083032514
C	-5.5980085683	-1.6402908518	5.7416727834
C	-7.8280497973	0.1836219193	4.4759760431
H	-6.5633527693	-3.4902384744	3.4865302854
C	-8.5278772448	-3.1792633671	4.2842576919
C	-7.7543577268	-2.6777622577	1.9252060595
C	-8.5966525795	0.6585586916	3.2269310397
H	-8.5526031026	-0.3430565370	5.1147666442
C	-7.3235338734	1.3962483650	5.2820436357
C	-5.0190917833	-3.0695499483	5.7167725320
C	-4.4829004314	-0.6490820546	6.1330733200
H	-6.3641099458	-1.6092133323	6.5302598468
H	-4.2831802229	-3.1851219991	4.9144516504
H	-4.5073039989	-3.2905552460	6.6602306821
H	-5.7869384974	-3.8338779518	5.5767836674
H	-3.6842539022	-0.6516528405	5.3869299798
H	-4.8416645971	0.3777234218	6.2240224035
H	-4.0423054091	-0.9320205754	7.0957509965
H	-8.2566387244	-3.2715405762	5.3402964669
H	-9.3639856944	-2.4759207499	4.2212894160
H	-8.9022618752	-4.1557924866	3.9568285397
H	-6.9159841446	-2.4200959154	1.2755127141
H	-8.1263777631	-3.6572466895	1.6022114972
H	-8.5535963326	-1.9561279248	1.7357301778
H	-9.0723320774	-0.1656761321	2.6920669818
H	-9.3860886861	1.3651454133	3.5080589429
H	-7.9415767559	1.1771257301	2.5203879024
H	-6.5328087638	1.9368681426	4.7521211841
H	-8.1407100784	2.1052982730	5.4587117002
H	-6.9285185817	1.1054891049	6.2576285896
Si	-4.8002450623	-0.9489414909	-2.3139601480
H	-2.0599469233	6.9118191215	-1.7964898684
C	-1.0883290982	3.2561751584	-3.8133230330
C	-2.2195287572	5.8372081187	-1.9379319033
H	-2.1252252888	6.7694883290	-5.8692206627
H	-3.6206111710	5.6649648078	-0.3134860388
H	-1.9588735470	5.3781680153	0.1648407005
C	-0.9280246924	5.1724016774	-2.4759765217
H	-3.0043267029	5.7288770502	-2.6946945907
C	0.2900262450	5.4840622664	-1.5772063233

C	-0.6695645730	5.5239431165	-3.9424214039
O	-0.1338764783	10.5085186841	-6.2923077938
O	-1.1640631520	8.5320597797	-6.0682646620
H	1.5129724701	9.6808315365	-4.3711565649
H	1.1990409482	5.0463054135	-1.9969159877
H	0.4354986313	6.5623366155	-1.4730452620
C	-0.7572648921	4.3898395305	-4.6720866954
H	0.1571147666	5.0751624162	-0.5742534566
C	0.7232469673	8.9986556789	-4.6567918680
C	-5.3539352651	-0.0866620089	-3.8885774824
C	-3.2719690703	-1.9985241597	-2.6572438423
C	-6.1757217935	-2.0801329498	-1.7029788842
H	-4.5739428391	0.5756687322	-4.2739790162
H	-5.5942703488	-0.8130992061	-4.6712413820
H	-6.2463477594	0.5195586036	-3.7094028058
H	-2.4475949486	-1.4024776544	-3.0578999736
H	-2.9132360948	-2.5115458407	-1.7604602826
H	-3.5129231636	-2.7691027028	-3.3974259141
H	-7.0644342856	-1.5101338277	-1.4188269947
H	-6.4681708799	-2.7818905841	-2.4909041569
H	-5.8646477815	-2.6708092422	-0.8378947948
H	-4.0434039021	3.9004225143	1.0643966455
Si	-1.8818028563	3.6238578602	2.9893276309
C	-1.5012045730	2.2188811831	4.1771196190
C	-0.3393780601	4.6789624458	2.7625124861
C	-3.2871305638	4.6905698651	3.6536955002
H	-0.7257517603	1.5650175198	3.7675944428
H	-1.1388171915	2.6027676019	5.1360068351
H	-2.3831855131	1.6040527317	4.3767810827
H	-4.2134339306	4.1229603351	3.7771574217
H	-3.0102698508	5.0828457714	4.6378561105
H	-3.4989442985	5.5503288745	3.0117459057
H	0.4706183911	4.0991907493	2.3101895286
H	-0.5313465244	5.5449319700	2.1228387757
H	0.0192203467	5.0521759859	3.7272365613
C	-0.4701968909	6.8773470583	-4.4896736186
C	-1.2895664466	7.3328667514	-5.4744660907
C	-0.1590720810	9.4607587123	-5.7079757342
C	0.5768470067	7.7803383071	-4.0819264736
H	-0.6083837606	4.3241067490	-5.7435459077
H	-1.2419471549	2.2496016841	-4.1782142342

bufogargarizin -B-diene-precursor-2

110

C	3.2211971393	-1.2807379914	0.0275942940
C	3.2801283162	-2.2429867006	-1.1549731228
C	4.7546860658	-2.6697155566	-1.1592127736
C	2.2767060288	-3.4008963696	-0.8675228337
H	2.7160555122	-4.0520573848	-0.1042840014
H	2.1372297187	-4.0171149502	-1.7612530796
C	1.8871619788	-0.7628737487	0.4744602540

H	1.4045207190	-0.3324883446	-0.4127240273
C	1.0537931964	-2.0206335102	0.8699466137
H	1.6734941106	-2.5911111092	1.5755643937
C	0.9023401546	-2.9114705231	-0.3723575631
H	0.2826888486	-3.7754280799	-0.1295238395
H	0.3810211525	-2.3703863471	-1.1695937029
C	-0.2447361357	-1.7245260458	1.6667951479
C	1.9259334119	0.3579092189	1.5270846426
H	2.8460163522	0.9345047499	1.3918527470
H	1.9660999647	-0.0533935214	2.5404224852
C	0.7464543751	1.3327556709	1.3694378659
H	0.9587242628	2.2574816031	1.9168502972
H	0.6817835333	1.6316223949	0.3183762320
C	-1.1218888905	-0.5206100996	1.2898560500
C	-0.6508878907	0.8622312833	1.8247721404
C	2.9355915301	-1.4964619633	-2.4659381229
H	3.0265197229	-2.1562497810	-3.3319765524
H	3.6123216075	-0.6519607986	-2.6149163668
H	1.9133165573	-1.1134143998	-2.4479833521
C	-1.7399976304	1.7804307228	1.2189677883
H	-1.4236962885	2.8191575477	1.1190883539
H	-2.6087056436	1.7502505275	1.8821497744
C	-2.1112070662	1.1803952164	-0.1648545404
O	-0.7173705799	0.7937725246	3.2540797808
H	-2.0502020880	-0.6897371375	1.8455313884
C	-1.5197106548	-0.2558996470	-0.1680422273
H	-2.2226650021	-1.0010448895	-0.5445921110
H	-0.6590774512	-0.2479215961	-0.8375289119
H	-3.2008851105	1.1471363114	-0.2650345826
O	-1.5564849926	1.9398398384	-1.2402916280
Si	-2.3358773063	2.8018025870	-2.4598283710
C	-3.8030340157	1.7592058694	-3.1121631447
H	-4.4330331439	1.5639671556	-2.2323269534
C	-0.9982664216	3.0361202152	-3.8025425289
H	-1.0270874599	2.0973729989	-4.3731745437
C	-2.9937053118	4.4653171330	-1.7876608889
H	-3.4316585669	4.9787974205	-2.6552125332
C	5.3947833839	-3.5782533774	-2.1101178867
C	4.7149687708	-4.3409009890	-3.0116761159
H	3.6418008781	-4.3611621128	-3.1204530512
O	5.3011967068	-5.1839148285	-3.8844402911
C	6.8328816605	-3.7273940104	-2.1458120124
H	7.4369106353	-3.1388498151	-1.4658875017
C	7.4459397463	-4.5686841573	-3.0075611132
H	8.5214987961	-4.6809884354	-3.0409202263
C	6.6958463799	-5.3703186758	-3.9534025463
O	7.1192232397	-6.1466128319	-4.7653050711
H	0.0738531777	-1.5137504337	2.6949731654
O	-1.0450682603	-2.9164006591	1.6663902922
Si	-1.6893670901	-3.6794899217	3.0161673485
C	-0.3208116324	-4.0942689677	4.2443434125

H	-0.7286194207	-4.6292765035	5.1082002370
H	0.1753519837	-3.1961917597	4.6245768661
H	0.4420848393	-4.7321952008	3.7888010129
C	-2.9649018095	-2.5839901266	3.8656099504
H	-3.8205168379	-2.3862972086	3.2133465377
H	-2.5312026395	-1.6225356596	4.1551387420
H	-3.3428921150	-3.0653417918	4.7734327007
C	-2.4875598025	-5.2407462001	2.3513925001
H	-3.2524326424	-5.0031086292	1.6067250391
H	-2.9673586213	-5.8093830687	3.1539686728
H	-1.7480983900	-5.8909992027	1.8760984531
C	4.4468565534	-1.1671247552	0.5760986307
H	4.7135669294	-0.5735612439	1.4410249570
C	5.3832311775	-2.0097620097	-0.1517731093
H	6.4316705634	-2.0876808870	0.1061670566
Si	-0.4346729848	1.8688343273	4.5088026688
C	1.3827883922	1.8564852579	5.0049980794
H	1.7089210420	0.8482012635	5.2763289155
H	1.5458567960	2.5020832091	5.8741626016
H	2.0334905727	2.2136061601	4.2025816933
C	-1.4741360490	1.2300970851	5.9361788357
H	-1.2156453557	0.1963884704	6.1822874827
H	-2.5402699136	1.2580637288	5.6948629300
H	-1.3171102492	1.8358833341	6.8342794140
C	-0.9529110278	3.6283191086	4.0737133957
H	-0.8197946719	4.2787897360	4.9446388736
H	-2.0057221127	3.6767080160	3.7832539379
H	-0.3596757007	4.0499439088	3.2578841815
C	-3.3648663218	0.3911359044	-3.6681901642
H	-2.7701679459	-0.1755274495	-2.9478946285
H	-2.7633559525	0.5020069251	-4.5762686394
H	-4.2358877075	-0.2196775811	-3.9312981963
C	-4.6823257756	2.5022273291	-4.1370859260
H	-5.0292834368	3.4695056291	-3.7648357408
H	-5.5685057114	1.9092450291	-4.3892735238
H	-4.1412106130	2.6817973147	-5.0711510194
C	-4.1168815458	4.2893530382	-0.7454476460
H	-3.7583953057	3.7619243175	0.1438706487
H	-4.9690612497	3.7296421751	-1.1406646353
H	-4.4922277313	5.2633284815	-0.4109607329
C	-1.8764000720	5.3667123675	-1.2309234119
H	-1.3595375591	4.8905861476	-0.3920798046
H	-2.2856018074	6.3161541095	-0.8672114843
H	-1.1243048688	5.6004998020	-1.9877257892
C	-1.3121642446	4.1763865357	-4.7927177382
H	-0.5798267562	4.1871154400	-5.6075251958
H	-1.2660890654	5.1563789341	-4.3081275283
H	-2.3022082096	4.0813508577	-5.2462651034
C	0.4325371557	3.1763136536	-3.2486194452
H	1.1599015757	3.1967537267	-4.0686246314
H	0.6898660723	2.3486354602	-2.5860528344

H	0.5587715699	4.1029692154	-2.6813174348

bufogargarizin -A-diene-precursor

119

C	-1.2759639161	1.5706864844	-3.0659129555
C	-1.5384537235	0.2803842571	-2.2482717075
H	0.4213722331	-0.5689652761	-2.6134680617
H	1.6325129564	1.5612157323	-1.7247331132
C	-0.2778350733	-0.4573122953	-1.7766596494
C	-0.7688711561	2.8345079030	-2.2568544846
H	-0.5074035218	1.3353092450	-3.8048231412
C	-2.5773364745	2.0510746690	-3.7910876917
C	-0.0950563662	2.6040335048	-0.8945187516
O	0.0902642927	3.6017900347	-3.1099707051
C	-2.0638684030	3.6675344344	-2.1071027523
C	1.0165471669	1.5480860899	-0.8224636472
H	0.3093770444	3.5729056258	-0.5904074067
H	-0.8683623837	2.3577891715	-0.1574680261
H	1.6833393250	1.7979300967	0.0087191115
Si	1.5889874747	3.6897031965	-3.8308888582
H	1.0717388939	5.9284854595	-4.8088545921
C	0.4936878123	0.1283795200	-0.5933367120
H	-0.5810412896	-1.4628548920	-1.4811459191
O	1.5629150533	-0.7817106381	-0.3408442687
H	-0.1725594435	0.1555876471	0.2802556423
C	-1.9953957744	5.1138238483	-1.6258065371
H	-2.5542766032	1.8240554614	-4.8572266893
H	-3.4383552982	1.5252615167	-3.3748893717
C	-2.6913668071	3.5510094358	-3.5105529099
H	-2.7153817331	3.1064000060	-1.4204097193
H	-2.0242127471	4.0950420914	-4.1904159708
C	-4.0256251711	4.2086385667	-3.5152223035
Si	2.4658279107	-1.1390912272	1.0322276785
C	2.7714207478	-3.0197787019	0.9330603090
C	4.1228436751	-0.1682497772	0.9654582177
C	1.5077580117	-0.5445864622	2.5860362302
C	3.5620839123	-3.3977596469	-0.3366282289
H	3.3942582465	-3.2716210560	1.8029111801
C	1.4919091129	-3.8752594680	1.0044348440
C	4.6115414116	0.1556647401	-0.4607539610
H	3.9030653566	0.7940777762	1.4515055863
C	5.2581588704	-0.8368406164	1.7707588774
C	0.1509510695	-1.2265356327	2.8521109950
C	2.3531888908	-0.5574308002	3.8762911126
H	1.2980551693	0.5118915287	2.3591602678
H	0.7957272716	-3.6136223287	0.2018659937
H	1.7387964856	-4.9365750153	0.8854443425
H	0.9621910301	-3.7713726537	1.9526417341
H	4.5316091666	-2.8986752357	-0.3928484993
H	3.7469784982	-4.4775184420	-0.3646487167
H	3.0010599731	-3.1357058386	-1.2381293094

H	0.2894545599	-2.2324421976	3.2556686215
H	-0.4208080974	-0.6605269194	3.5965015005
H	-0.4711781876	-1.3171451919	1.9583053354
H	2.6789317323	-1.5705484315	4.1353593412
H	3.2441565712	0.0693470605	3.7976673176
H	1.7661215866	-0.1830142751	4.7224047644
H	3.8467819552	0.6606595855	-1.0520759587
H	5.4937685700	0.8051331772	-0.4234856730
H	4.8972865156	-0.7480507826	-1.0063599816
H	5.5612386463	-1.7835852407	1.3147759622
H	6.1438747537	-0.1923205526	1.7969114628
H	4.9784056561	-1.0482514143	2.8052671301
H	0.6312388568	4.6331487421	-5.9280459812
H	-4.0332605722	5.2510501786	-0.9129454198
C	-3.4018998146	5.7545348538	-1.6536795445
C	-4.1037580745	5.6588444293	-3.0409976751
H	-1.6113345635	5.1740477004	-0.6015677026
H	-1.3000864554	5.6741781706	-2.2565863281
H	-3.3231967489	6.8033313170	-1.3511484873
C	-5.2674955290	3.7323720837	-3.7283147452
C	-3.4506568171	6.6225311615	-4.0583929706
C	-5.6167678666	5.9025792162	-3.0065603995
O	-8.3663024735	10.6292776350	-2.1470469783
H	2.3180707039	1.2668999465	-3.8634305306
H	-7.4703254259	7.2131533556	-4.5237299517
H	-3.9225992007	6.5254151335	-5.0388670123
H	-3.5606956110	7.6597550018	-3.7292419508
C	-6.2415940239	4.7771116942	-3.4248621630
H	-2.3847204604	6.4168003078	-4.1741737880
H	2.3245850872	5.1038475516	-5.7429747331
H	-2.1474740348	0.5411670983	-1.3726295538
O	-2.3604743443	-0.6099716318	-3.0074054008
Si	-2.2078350513	-1.8636953970	-4.1077073237
C	-3.9981894853	-2.3547151791	-4.4755410406
C	-1.2612100394	-1.3620452632	-5.6800663911
C	-1.3170555020	-3.3642852730	-3.3564482806
C	-5.0236358303	-1.2155001162	-4.6140442563
H	-4.0049432575	-2.9740372430	-5.3815165910
H	-4.3168557990	-3.0229662463	-3.6654625559
H	-1.7262873777	-3.5334856730	-2.3531374831
C	-1.4406289831	-4.6597685441	-4.1828068441
H	-0.2574832947	-3.1263320301	-3.2121736504
C	-2.0478200582	-0.6557154446	-6.7986815506
H	-0.3977910643	-0.7523459282	-5.3861070904
H	-0.8266668851	-2.2854032974	-6.0849751478
H	-4.7867187826	-0.5476272062	-5.4465522039
H	-6.0297474524	-1.6090937253	-4.7891524590
H	-5.0551864465	-0.6090177839	-3.7065416084
H	-2.4749140201	0.2928332101	-6.4647959560
H	-1.4099958063	-0.4396057097	-7.6613961996
H	-2.8749714113	-1.2762413337	-7.1536793944

H	-2.4844633367	-4.9580138738	-4.3141227282
H	-1.0044504573	-4.5499550401	-5.1803349176
H	-0.9234628245	-5.4922680562	-3.6966006322
C	1.3835738070	4.9572747198	-5.2030359533
C	2.8854567942	4.2931588834	-2.6057476549
C	2.1520754573	2.0592803943	-4.5980719151
H	2.5934762763	5.2526860107	-2.1690473307
H	3.0430002711	3.5866363345	-1.7877413024
H	3.8476340954	4.4368140231	-3.1079907159
H	1.4335681567	1.6902064819	-5.3357443209
H	3.1016899579	2.2186921397	-5.1199830839
C	-6.2778716850	7.1863323665	-2.7247348038
H	-5.3086635625	7.6340420644	-0.8179355484
C	-7.7020251384	9.6341990636	-2.2395108153
C	-6.0308024464	7.9807710437	-1.5473878465
O	-7.8933488411	8.8207522456	-3.3814398315
H	-6.5361322498	9.7351675091	-0.4340398021
C	-6.7015941881	9.1355381613	-1.3193805457
C	-7.2051248488	7.6845835699	-3.5861319377
H	-5.5243834454	2.7297250886	-4.0451093888
H	-7.3150661008	4.6560253141	-3.5105590036

bufogargarizin -B-β-endoperoxide. (0 kcal/mol)
112

C	-4.3307102346	1.8619699078	0.5121527352
C	-4.9229961147	1.4676239917	1.8805114879
C	-2.2001393714	0.6327157627	-0.4283403680
H	-4.0563461642	-1.5257134745	0.6026137214
C	-3.6947980020	0.5436638901	-0.0790196748
C	-5.1700415125	-0.0413007260	1.8023875973
H	-5.1516173928	2.1005117207	-0.1692520963
C	-3.4893032174	3.1510748379	0.6015545448
C	-3.9483229224	-0.5197608681	1.0132664619
H	-4.1888963662	1.6272628316	2.6741150256
H	-5.8200283331	2.0391185273	2.1304173122
H	-3.1049362475	-0.5415094146	1.7113512443
O	-4.4580242120	0.2535464540	-1.2691813376
H	-6.0896805252	-0.2483307956	1.2430186163
O	-5.2210501293	-0.6585699153	3.0816677074
C	-2.8348839694	5.2594664337	-0.6141497692
C	-1.3602245392	3.7982507496	-2.5935309343
C	-3.0219719412	3.7276399131	-0.7628171102
C	-1.8614335488	1.5351562839	-1.6203901187
H	1.8474381679	6.4036686612	-4.0082328984
O	-2.3791247985	3.0091835116	1.4872983597
C	-1.7447962463	3.0426956667	-1.3278171028
H	-1.6596021241	0.9613122257	0.4612680662
H	-1.8539476963	-0.3812195327	-0.6513167695
H	-0.9428013080	3.1863192026	-0.5986893186
H	-3.8419745364	3.5551974632	-1.4711782637
H	-2.6059553182	1.3699267071	-2.4030838032

H	-0.9015506084	1.2166540933	-2.0365362308
Si	-6.4335921993	-1.1973917955	4.1058148937
C	-7.2755432922	-2.7703883648	3.4036220288
C	-5.5028171868	-1.6747980131	5.7082258197
C	-7.7631910602	0.1344318009	4.4738007862
H	-6.4891035593	-3.5328123242	3.4737471218
C	-8.4445227079	-3.2304620219	4.2971118483
C	-7.7062914508	-2.7341342771	1.9261874529
C	-8.5704994577	0.5792996922	3.2380772164
H	-8.4643784801	-0.3890224516	5.1406845463
C	-7.2489548940	1.3643281993	5.2469208507
C	-4.9118515257	-3.0989102571	5.6723383158
C	-4.3916856250	-0.6750597108	6.0889908462
H	-6.2595134140	-1.6523088327	6.5061418503
H	-4.1861245662	-3.2063097647	4.8597066879
H	-4.3853710974	-3.3174526757	6.6082151909
H	-5.6750120070	-3.8694052600	5.5409661196
H	-3.6004845294	-0.6699092275	5.3349111193
H	-4.7575840503	0.3487297409	6.1855624340
H	-3.9386164758	-0.9557265152	7.0464990870
H	-8.1580757583	-3.3207520301	5.3492318615
H	-9.2842573085	-2.5305447723	4.2454215966
H	-8.8193219152	-4.2088219323	3.9757925882
H	-6.8779352946	-2.4753154392	1.2642090051
H	-8.0768453780	-3.7173324456	1.6130779890
H	-8.5116385347	-2.0178738434	1.7429975243
H	-9.0581657532	-0.2590673521	2.7372056876
H	-9.3544215862	1.2892334843	3.5260942822
H	-7.9394238327	1.0827949741	2.4997297450
H	-6.4765820045	1.9030381562	4.6888851954
H	-8.0675948664	2.0691088341	5.4336147816
H	-6.8268103338	1.0922465631	6.2164898172
Si	-4.6584024889	-1.0503724273	-2.3006284803
H	-2.1399063743	7.0423011696	-1.6369629996
C	-2.3005264631	3.7559701003	-3.7898963465
C	-2.3480899583	5.9953239297	-1.8804506643
H	-2.1495537631	7.4358854875	-5.2358260624
H	-3.7875151263	5.6966796449	-0.2974347872
H	-2.1291092980	5.4368670123	0.2022872112
C	-1.0851071114	5.3139224946	-2.4214326942
H	-3.1436037325	5.9953252239	-2.6282178951
C	0.1243730531	5.6454827354	-1.5384699334
C	-0.7577381665	5.4299714623	-3.9511708966
H	-3.0157246875	2.9706338997	-3.9904068132
O	-0.7032045802	8.7930903308	-5.5793984044
H	2.5970802747	8.6002966355	-4.8864375217
H	1.0209401618	5.1225368442	-1.8634067989
H	0.3165706310	6.7223113667	-1.5613090316
C	-1.9388227996	4.7464265512	-4.6064241873
H	-0.0588912509	5.3718583942	-0.4982583922
C	1.5601399002	8.2944259760	-4.9235683312

C	-5.0429468849	-0.2928845967	-3.9772655762
C	-3.1290679493	-2.1442706031	-2.4231367970
C	-6.1169823386	-2.1031736571	-1.7478765344
H	-4.1888914911	0.2664508482	-4.3696600054
H	-5.2946310191	-1.0676072834	-4.7085215407
H	-5.8926837439	0.3923418768	-3.9112621221
H	-2.2602009436	-1.5925596187	-2.7911882710
H	-2.8618569239	-2.5922586788	-1.4622945196
H	-3.3207284285	-2.9635265472	-3.1238804114
H	-7.0207322324	-1.4996946825	-1.6250116958
H	-6.3319637900	-2.8771333927	-2.4918389720
H	-5.9186521040	-2.6071666048	-0.7989142066
H	-4.1756617628	3.8988343470	1.0226925744
Si	-2.0193819235	3.7123733904	2.9699760553
C	-1.6282438223	2.3202577560	4.1687862983
C	-0.4976945113	4.8003551932	2.7670871178
C	-3.4669320623	4.7459196311	3.5951296132
H	-0.8326951172	1.6821917171	3.7730722618
H	-1.2876567198	2.7160036727	5.1308625972
H	-2.4987684068	1.6865842748	4.3590796065
H	-4.3750815293	4.1501609563	3.7217458043
H	-3.2171729317	5.1735798485	4.5716220019
H	-3.6998744823	5.5800877585	2.9269592479
H	0.3266742130	4.2416800265	2.3143019788
H	-0.7014687576	5.6698946245	2.1362269690
H	-0.1533768326	5.1692508175	3.7386010460
C	-0.2432294299	6.7176179357	-4.5180296383
C	-1.0874130651	7.6000922807	-5.1025513435
C	0.6449938221	9.2411917511	-5.5266240018
C	1.1410288273	7.0996615177	-4.4420009260
O	0.8824035448	10.3260585734	-5.9767834107
O	-0.0757294072	3.3409294537	-3.1362276304
O	0.3285368424	4.4289320290	-4.0530443862
H	-2.2758002566	4.9327195935	-5.6178325901

bufogargarizin -B-α-endoperoxide. (0.22 kcal/mol)

112

C	-3.5048878155	0.7020054451	3.7722666478
C	-4.1420242668	0.2541008635	5.0991567611
C	-1.3033382380	-0.5831091217	3.1065314648
H	-3.5345573930	-2.6505521406	3.5119020672
C	-2.8436393942	-0.5894757636	3.1570693079
C	-4.5250520348	-1.2110079487	4.8746124966
H	-4.3117026320	0.9670295389	3.0820402872
C	-2.6680597013	1.9880884456	3.9099117795
C	-3.3279964130	-1.7409310871	4.0772011848
H	-3.4048458818	0.2816455926	5.9056538310
H	-4.9886990329	0.8800536225	5.3938989941
H	-2.5486327713	-1.9782188903	4.8074289513
O	-3.2779725333	-0.7468204022	1.8027611213
H	-5.4408430813	-1.2671544401	4.2739210508

O	-4.6838816494	-1.9279932932	6.0901023789
C	-1.9634308647	4.0239588836	2.6030573752
C	0.0421487451	2.5458327314	1.1390793349
C	-2.0219592956	2.4780669836	2.5840527006
C	-0.6706192494	0.3131742323	2.0374482010
H	0.1591624690	4.3276483709	7.2523541222
O	-1.6849842595	1.8689754425	4.9422369733
C	-0.6356579268	1.8327513222	2.3070487102
H	-0.9194845551	-0.3343577125	4.0989973532
H	-0.9990813355	-1.6136055039	2.8909785459
H	-0.0087516339	2.0127614270	3.1871552517
H	-2.6904861793	2.1795849025	1.7708274417
H	-1.1735580450	0.1267157817	1.0872592380
H	0.3637555937	-0.0220622717	1.9104791123
Si	-5.9687546808	-2.5406730849	6.9748426480
C	-6.7313953600	-4.0595729803	6.0950020627
C	-5.1535755626	-3.1045553580	8.6138607431
C	-7.3178529378	-1.2270768755	7.3256697113
H	-5.9494166772	-4.8245185477	6.1858713415
C	-7.9728418970	-4.5811636875	6.8449607915
C	-7.0191932851	-3.9135702170	4.5899440136
C	-8.0096803502	-0.6788991978	6.0628160517
H	-8.0792114913	-1.7816713374	7.8933517499
C	-6.8448388633	-0.0657756835	8.2221543532
C	-4.6164994891	-4.5497188180	8.5734565266
C	-4.0268481592	-2.1550182876	9.0688555746
H	-5.9487667315	-3.0706901354	9.3726203770
H	-3.8570631350	-4.6711046107	7.7942445188
H	-4.1435526111	-4.8069493508	9.5278597590
H	-5.4014468592	-5.2879764647	8.3918595835
H	-3.2115556059	-2.1509481175	8.3415727924
H	-4.3654192857	-1.1238146150	9.1845270258
H	-3.6189366051	-2.4806149780	10.0325026564
H	-7.7784305338	-4.7342082937	7.9111321211
H	-8.8116575780	-3.8829385716	6.7627372704
H	-8.3086686586	-5.5402136687	6.4344531903
H	-6.1320020440	-3.5960543818	4.0371968606
H	-7.3397238041	-4.8716746461	4.1647963796
H	-7.8148114400	-3.1917994283	4.3858097057
H	-8.4625481997	-1.4701673094	5.4618769985
H	-8.8063208893	0.0230525569	6.3345303172
H	-7.3099195784	-0.1344454773	5.4211044332
H	-6.0145387625	0.4822630553	7.7663072582
H	-7.6587813654	0.6497750527	8.3864901236
H	-6.5122756316	-0.4123895933	9.2030186319
Si	-4.5251095797	-1.2665697371	0.8315426698
H	-1.2428493911	5.7544662064	1.5182745299
C	1.4236553879	2.1634236221	0.6407387393
C	-1.2967410679	4.6699556019	1.3777776533
H	-0.2747574898	4.6989442965	5.5829833530
H	-2.9866498139	4.4054003494	2.6854081282

H	-1.4491595803	4.3443101636	3.5155563464
C	0.1114234244	4.0965366205	1.1755738910
H	-1.9060113026	4.4897896403	0.4919941938
C	1.0868143098	4.6971318405	2.1936649494
C	0.6474063243	4.1102177482	-0.3017821982
H	0.8630067510	3.3898944103	5.9336330538
C	0.8603683197	5.4136127507	-1.0068338814
H	2.6489706655	3.1075877617	-0.8890205089
H	2.0802936564	4.2536713567	2.1404916783
H	1.1805310218	5.7746606156	2.0288911664
C	1.7934985130	3.1115983118	-0.2259012369
H	0.7198554259	4.5610387301	3.2122617314
C	1.2466555619	7.9194487453	-2.3230575875
C	-4.0134581847	-0.7549887761	-0.8990354862
C	-4.7306348266	-3.1393177624	0.9058372165
C	-6.1700216053	-0.4480017360	1.2643408713
H	-3.0666907495	-1.2233956622	-1.1811938327
H	-4.7666241996	-1.0474157251	-1.6375820930
H	-3.8764314506	0.3277685851	-0.9657784667
H	-3.7857416122	-3.6474506604	0.6918941275
H	-5.0858328065	-3.4840921549	1.8800744246
H	-5.4596446617	-3.4679775250	0.1576104799
H	-6.0975160726	0.6428386433	1.2210995339
H	-6.9341056659	-0.7534021255	0.5414324989
H	-6.5388801243	-0.7213302904	2.2570241150
H	-3.3904025034	2.7620212955	4.2059326597
Si	-1.4770799591	2.6570462017	6.4072257885
C	-1.0264757965	1.3403616359	7.6681316090
C	-0.0526498013	3.8819901648	6.2747286552
C	-3.0461085646	3.5652291017	6.9164355783
H	-0.1586833677	0.7643454060	7.3337695961
H	-0.7715824542	1.7937740800	8.6314211118
H	-1.8466863299	0.6378317717	7.8375012387
H	-3.8961047597	2.8848541831	7.0152247967
H	-2.8951152192	4.0472040354	7.8875785735
H	-3.3212114822	4.3484555337	6.2043727188
O	1.2819624229	8.9580831793	-2.9210972804
H	2.8354686478	5.7856569146	-0.1572607105
C	2.0623602424	6.1743995407	-0.8115713001
O	0.0996947799	7.1039332067	-2.4908033151
H	3.1421322184	7.9551527951	-1.3020169185
C	2.2456194798	7.3641470435	-1.4330927029
C	-0.0594433122	5.9312265230	-1.8564918802
H	-0.9909265053	5.4465149941	-2.1115214042
O	-0.4145594284	3.3835142118	-0.9984852669
O	-0.8228655087	2.3442178809	-0.0486818931
H	1.9267584070	1.2275916212	0.8401026425

bufogargarizin -A-β-endoperoxide. (0.73 kcal/mol)
121

C	-1.2891466177	1.5694695720	-3.0778943418
C	-1.5528200825	0.3044121012	-2.2220321287
H	0.3884458327	-0.5793743459	-2.6000379593
H	1.6478802943	1.5516304769	-1.7992069432
C	-0.2927925653	-0.4354908056	-1.7531992392
C	-0.7544783544	2.8506733921	-2.3161179310
H	-0.5365804674	1.3074970975	-3.8249071720
C	-2.5994735067	2.0422161217	-3.7911111081
C	-0.0472359835	2.6491387188	-0.9666312131
O	0.0837751490	3.5864221054	-3.2199843541
C	-2.0402302703	3.6930960150	-2.1500589849
C	1.0490699828	1.5778138489	-0.8856918945
H	0.3772378787	3.6197077089	-0.6970117047
H	-0.8077242412	2.4345154145	-0.2066720038
H	1.7352995106	1.8413300225	-0.0748122683
Si	1.5911642623	3.6609775905	-3.9248207379
H	1.0807926326	5.8783246154	-4.9550083213
C	0.5078981398	0.1748247048	-0.6021305383
H	-0.6022890481	-1.4280685599	-1.4224874334
O	1.5682260760	-0.7423520385	-0.3378752222
H	-0.1427701711	0.2421191447	0.2809477904
C	-1.9476464478	5.1465946263	-1.6808681789
H	-2.5872803011	1.7998606917	-4.8542420850
H	-3.4587517261	1.5305680703	-3.3571535481
C	-2.6968034852	3.5507379469	-3.5310270639
H	-2.6717480080	3.1475522845	-1.4382366342
H	-2.0757581300	4.0872019159	-4.2555806850
C	-4.0514951276	4.2181459943	-3.5470177587
Si	2.4676761335	-1.0888192333	1.0400074692
C	2.7592923121	-2.9731990847	0.9657921080
C	4.1320250148	-0.1322001347	0.9617518140
C	1.5114536052	-0.4672461303	2.5847266660
C	3.5526584790	-3.3746265213	-0.2951964078
H	3.3757695175	-3.2192767851	1.8418519375
C	1.4728726912	-3.8178909087	1.0406717374
C	4.6279220448	0.1630652350	-0.4684182367
H	3.9184008430	0.8404516418	1.4295664789
C	5.2597050326	-0.7948388920	1.7823090558
C	0.1497079315	-1.1366848925	2.8568007181
C	2.3539494532	-0.4701302577	3.8769976660
H	1.3094413284	0.5876808639	2.3440047318
H	0.7822024322	-3.5593833693	0.2324238928
H	1.7124098913	-4.8821349351	0.9336174764
H	0.9397926910	-3.7005518327	1.9853659453
H	4.5281397997	-2.8875471326	-0.3526412065
H	3.7254795180	-4.4566979143	-0.3094697837
H	2.9991035552	-3.1172713516	-1.2026996829
H	0.2809988628	-2.1393952495	3.2706901545
H	-0.4180903343	-0.5591213250	3.5953064477

H	-0.4735300130	-1.2318245417	1.9642147421
H	2.6747608452	-1.4819341953	4.1470710669
H	3.2477280396	0.1521785019	3.7947743724
H	1.7661620236	-0.0847349758	4.7176519823
H	3.8696308803	0.6654205494	-1.0702037412
H	5.5161576148	0.8047197326	-0.4391557802
H	4.9063914970	-0.7522148763	-0.9979774554
H	5.5579284886	-1.7514830995	1.3439297168
H	6.1498767617	-0.1561617648	1.8004428346
H	4.9748758821	-0.9863754413	2.8192209964
H	0.6450892501	4.5576410145	-6.0470212325
H	-3.9669589033	5.3144701046	-0.9329420603
C	-3.3502172753	5.8044121091	-1.6873407685
C	-4.0277928830	5.7003910820	-3.0677935214
H	-1.5423971936	5.2046998511	-0.6653828530
H	-1.2559512149	5.6941696007	-2.3269853715
H	-3.2618558402	6.8574631194	-1.4017180451
C	-4.9780358801	4.2590551684	-4.7423642001
C	-3.4001044998	6.7061936307	-4.0372600826
C	-5.5976443268	5.7233615557	-3.0796789426
O	-8.1475726447	10.3524929869	-1.3996139220
H	2.3334866670	1.2405271103	-3.9059644780
H	-6.8287046946	5.3719251418	-5.0048752879
H	-3.8224284435	6.6487161515	-5.0394381421
H	-3.5521727404	7.7222787866	-3.6608736663
C	-5.9120447592	5.1749910607	-4.4643758977
H	-2.3228181315	6.5482635434	-4.1184633102
H	2.3375353966	5.0321548084	-5.8645829354
H	-2.1436565816	0.5957770394	-1.3439820388
O	-2.3971671003	-0.5944605178	-2.9452958417
Si	-2.2758482589	-1.8824828439	-4.0088233110
C	-4.0766974498	-2.3448470958	-4.3597722535
C	-1.3203246925	-1.4462021647	-5.5958580194
C	-1.4134395177	-3.3774022658	-3.2136884126
C	-5.0824854854	-1.1893364108	-4.5073759865
H	-4.0996533682	-2.9765075435	-5.2570447406
H	-4.4029232460	-2.9961659934	-3.5389658453
H	-1.8128532439	-3.4976120543	-2.1995069622
C	-1.5814098474	-4.6999591221	-3.9877291989
H	-0.3461631540	-3.1612089327	-3.0930035810
C	-2.0890369520	-0.7438494975	-6.7292045929
H	-0.4397895239	-0.8541351380	-5.3178668162
H	-0.9131753346	-2.3909682487	-5.9790904025
H	-4.8381369706	-0.5357612802	-5.3490767530
H	-6.0957918043	-1.5677131627	-4.6739968392
H	-5.1013938317	-0.5700418728	-3.6087013911
H	-2.4778121867	0.2288898708	-6.4187093905
H	-1.4506902820	-0.5758707043	-7.6022409524
H	-2.9418956475	-1.3421258661	-7.0605245253
H	-2.6343637452	-4.9751801968	-4.0936564865
H	-1.1567077396	-4.6394880676	-4.9942259404

H	-1.0788701999	-5.5264524091	-3.4765621927
C	1.3939431499	4.8984611840	-5.3260412397
C	2.8788317006	4.2920138403	-2.7042341961
C	2.1589039110	2.0159264180	-4.6566755936
H	2.5877479970	5.2643876630	-2.2962830404
H	3.0258350902	3.6067163703	-1.8665776097
H	3.8459261197	4.4174597661	-3.2018871254
H	1.4361317050	1.6277077722	-5.3802871900
H	3.1039455384	2.1669897804	-5.1892460184
C	-6.3247342771	6.9302708936	-2.5735937096
H	-6.1923898435	6.4183251881	-0.4635496497
C	-7.6092472744	9.3122782980	-1.6526052984
C	-6.5244165040	7.1688048009	-1.1693730955
O	-7.3954774744	9.0033474050	-3.0231799862
H	-7.3059675770	8.4944706383	0.3144173799
C	-7.1363341878	8.2961329045	-0.7353943997
C	-6.7912472559	7.8745881548	-3.4240105770
H	-6.7170834167	7.8190303231	-4.5027446743
O	-5.9088127935	4.6053158327	-2.1807059536
O	-4.8828292103	3.6011454800	-2.4898379579
H	-4.9656760433	3.5635776449	-5.5712262951

bufogargarizin -A-α-endoperoxide. (0 kcal/mol)

121

C	-1.4077124497	1.4791508978	-2.7384175056
C	-1.5480606348	0.0906850486	-2.0646956354
H	0.4537361541	-0.5547911316	-2.5895949621
H	1.5567762262	1.5211270794	-1.4731717933
C	-0.2200317793	-0.6007205445	-1.7259741579
C	-0.9435002762	2.6813277283	-1.8195080860
H	-0.6617553786	1.3708287984	-3.5266211687
C	-2.7646533049	1.9382618708	-3.3625415860
C	-0.2230396994	2.3420997871	-0.5050844795
O	-0.1457438569	3.5794708063	-2.5982332102
C	-2.2756922829	3.4290328246	-1.5469335723
C	0.9606971150	1.3659355747	-0.5706738441
H	0.1214714841	3.2936661362	-0.0924269746
H	-0.9609632583	1.9539584117	0.2079820498
H	1.6258195468	1.5702165084	0.2739900847
Si	1.2857920015	3.7924323620	-3.4236340554
H	0.6195586660	6.0960131251	-4.1005691894
C	0.5457279609	-0.1045808532	-0.4983336704
H	-0.4375340799	-1.6550402237	-1.5480371353
O	1.6807445826	-0.9598317936	-0.3807803773
H	-0.0973846171	-0.2274597453	0.3848250043
C	-2.2388575201	4.8249088274	-0.9127317502
H	-2.7807218777	1.8212734937	-4.4457060303
H	-3.5704571374	1.3146842932	-2.9724309466
C	-2.9488686579	3.3959488137	-2.9326629736
H	-2.8604027529	2.7702188039	-0.8876195184
H	-2.3855485669	4.0481425178	-3.6037088306

C	-4.3377358856	3.9642588387	-2.7879777938
Si	2.7068724280	-1.3820929691	0.8812910955
C	3.1186577329	-3.2182221109	0.5685752083
C	4.3030249830	-0.3116467996	0.8047855104
C	1.8440393712	-0.9899481761	2.5483232905
C	3.6946954706	-3.4254021961	-0.8476814875
H	3.9036361635	-3.4819158390	1.2904046147
C	1.9361481366	-4.1809153778	0.7877076434
C	4.6494011030	0.2239812497	-0.5981348335
H	4.0834620226	0.5644855930	1.4324955148
C	5.5331251187	-1.0143815267	1.4180274718
C	0.5075746105	-1.7117590921	2.8141958000
C	2.7659449213	-1.1293913568	3.7767833196
H	1.6142971873	0.0825515020	2.4492964584
H	1.0754277258	-3.9022048050	0.1721795472
H	2.2186524223	-5.2020768168	0.5066617078
H	1.6083851188	-4.2102702102	1.8284252169
H	4.5927233152	-2.8301556826	-1.0282355171
H	3.9616228630	-4.4770731178	-1.0007611079
H	2.9590803572	-3.1548750528	-1.6101399651
H	0.6672818117	-2.7574594075	3.0865826301
H	-0.0207142951	-1.2400823112	3.6504708241
H	-0.1627775613	-1.6999299484	1.9516120334
H	3.0848214987	-2.1662319095	3.9270563896
H	3.6641270380	-0.5126023579	3.6975384438
H	2.2398152200	-0.8218648140	4.6876411978
H	3.8349169846	0.8082959659	-1.0287636996
H	5.5350405549	0.8683941586	-0.5527539735
H	4.8726108972	-0.5849927611	-1.2989411230
H	5.8503334236	-1.8635895725	0.8062773200
H	6.3818064264	-0.3240449402	1.4774089528
H	5.3459065846	-1.3903036812	2.4268037915
H	0.1439688560	4.9218765510	-5.3322820086
H	-4.2527756667	4.9286823410	-0.1149699124
C	-3.6540185528	5.4624121435	-0.8555798715
C	-4.3349603245	5.4198822836	-2.2337157195
H	-1.8412260419	4.7712516501	0.1053087583
H	-1.5547750819	5.4580812524	-1.4822220717
H	-3.5717005495	6.4986409511	-0.5126239274
C	-5.3534304363	3.2592513478	-1.9047295344
C	-3.6953920493	6.4541674224	-3.1686782889
C	-5.9028690099	5.4389938258	-2.3065877815
H	2.7523385982	2.4985701021	-4.9370412195
H	2.0397386195	1.4057478086	-3.7545614898
C	-8.0285713596	9.1213231260	-1.3547548966
H	-4.0945468495	6.3934153470	-4.1784620820
H	-3.8715804487	7.4615069657	-2.7794914570
C	-6.2959451986	4.1548736625	-1.6050261183
H	-2.6144214555	6.3179115482	-3.2258737036
H	1.8283545410	5.4421037731	-5.2101656174
H	-2.1350543781	0.2017487205	-1.1429500215

O	-2.3401027539	-0.7624346287	-2.8955072738
Si	-2.1233063477	-1.8417434219	-4.1625695443
C	-3.8845402420	-2.3539003150	-4.6211539412
C	-1.1669752053	-1.0882225935	-5.6260904297
C	-1.1717733250	-3.3828740916	-3.5882050266
C	-4.9442466772	-1.2400329174	-4.6947454502
H	-3.8452556053	-2.8931580347	-5.5763950649
H	-4.1997929554	-3.0991812235	-3.8800180382
H	-1.5909878936	-3.6969213046	-2.6245977455
C	-1.2065970974	-4.5664398213	-4.5735046754
H	-0.1311487777	-3.1065677853	-3.3855336976
C	-1.9627623076	-0.3186822234	-6.6972942391
H	-0.3733442949	-0.4504949912	-5.2188489218
H	-0.6396778429	-1.9186138947	-6.1132298636
H	-4.7109198182	-0.5001285993	-5.4642156716
H	-5.9320723094	-1.6494741805	-4.9271080809
H	-5.0194825057	-0.7095875927	-3.7432745994
H	-2.4578963155	0.5670916185	-6.2916308349
H	-1.3116635759	0.0181891958	-7.5098260493
H	-2.7385425708	-0.9481772780	-7.1414566427
H	-2.2279258214	-4.9128264112	-4.7538249305
H	-0.7758643546	-4.3004229188	-5.5435428452
H	-0.6371467013	-5.4192194481	-4.1923329579
C	0.9360224048	5.1914110779	-4.6277741301
C	2.6579114384	4.3149010587	-2.2441705496
C	1.8305437278	2.2680046224	-4.3923337042
H	2.3531419148	5.1842460944	-1.6544180371
H	2.9367044687	3.5184208327	-1.5506354603
H	3.5558855108	4.5941107046	-2.8050961165
H	1.0807250613	1.9691912760	-5.1303153951
C	-6.6592054761	6.6996658522	-2.0140028878
H	-6.5077893461	7.5534330615	-4.0070807532
O	-8.6095519566	10.0872097972	-0.9478525784
H	-5.3731915507	2.1961144498	-1.7066764359
C	-6.8676595410	7.7242943441	-3.0007511642
O	-7.7957698707	8.0630048557	-0.4341944361
H	-7.6972147669	9.6505570072	-3.4139231911
C	-7.5198117213	8.8694814753	-2.6869632321
C	-7.1545207180	6.9359103963	-0.7765079422
H	-7.0771813445	6.2470185920	0.0553575254
O	-5.0316187904	4.1470854392	-4.0620379951
O	-6.0955329554	5.1271204760	-3.7405790675
H	-7.2513208335	3.9829356281	-1.1264734975

7. References

[1] L.-P. Zhong, R. Feng, J.-J. Wang, C.-C. Li, *J. Am. Chem. Soc.* **2023**, *145*, 2098–2103.

[2] Y. Xie, H. Yang, Y. Tang, J. Zhao, H. Li, L. Xu, *Tetrahedron Lett.* **2025**, *164*, 155627.

[3] A. Inoue, H. Shinokubo, K. Oshima, *Org. Lett.* **2000**, *2*, 651–653.

[4] C. Tichvon, E. Zviagin, Z. Surma, P. Nagorny, *Org. Lett.* **2024**, *26*, 2445–2450.

[5] F. Balssa, M. Fischer, Y. Bonnaire, *Steroids* **2014**, *86*, 1–4.

[6] G. R. Pettit, B. R. Moser, R. F. Mendonça, J. C. Knight, F. Hogan, *J. Nat. Prod.* **2012**, *75*, 1063–1069.

[7] H. Tian, L. Wang, X. Zhang, Y. Wang, D. Zhang, R. Jiang, Z. Liu, J. Liu, Y. Li, W. Ye, *Chem. – A Eur. J.* **2010**, *16*, 10989–10993.

[8] G. M. Sheldrick, *Acta Crystallogr. Sect. A Found. Adv.* **2015**, *71*, 3–8.

[9] O. V. Dolomanov, L. J. Bourhis, R. J. Gildea, J. A. K. Howard, H. Puschmann, *J. Appl. Crystallogr.* **2009**, *42*, 339–341.

[10] G. M. Sheldrick, *Acta Crystallogr. Sect. C Struct. Chem.* **2015**, *71*, 3–8.

[11] E. Epifanovsky, A. T. B. Gilbert, X. Feng, J. Lee, Y. Mao, N. Mardirossian, P. Pokhilko, A. F. White, M. P. Coons, A. L. Dempwolff, Z. Gan, D. Hait, P. R. Horn, L. D. Jacobson, I. Kaliman, J. Kussmann, A. W. Lange, K. U. Lao, D. S. Levine, J. Liu, S. C. McKenzie, A. F. Morrison, K. D. Nanda, F. Plasser, D. R. Rehn, M. L. Vidal, Z.-Q. You, Y. Zhu, B. Alam, B. J. Albrecht, A. Aldossary, E. Alguire, J. H. Andersen, V. Athavale, D. Barton, K. Begam, A. Behn, N. Bellonzi, Y. A. Bernard, E. J. Berquist, H. G. A. Burton, A. Carreras, K. Carter-Fenk, R. Chakraborty, A. D. Chien, K. D. Closser, V. Cofer-Shabica, S. Dasgupta, M. de Wergifosse, J. Deng, M. Diedenhofen, H. Do, S. Ehlert, P.-T. Fang, S. Fatehi, Q. Feng, T. Friedhoff, J. Gayvert, Q. Ge, G. Gidofalvi, M. Goldey, J. Gomes, C. E. González-Espinoza, S. Gulania, A. O. Gunina, M. W. D. Hanson-Heine, P. H. P. Harbach, A. Hauser, M. F. Herbst, M. Hernández Vera, M. Hodecker, Z. C. Holden, S. Houck, X. Huang, K. Hui, B. C. Huynh, M. Ivanov, Á. Jász, H. Ji, H. Jiang, B. Kaduk, S. Kähler, K. Khistyaev, J. Kim, G. Kis, P. Klunzinger, Z. Koczor-Benda, J. H. Koh, D. Kosenkov, L. Koulias, T. Kowalczyk, C. M. Krauter, K. Kue, A. Kunitsa, T. Kus, I. Ladjánszki, A. Landau, K. V. Lawler, D. Lefrancois, S. Lehtola, R. R. Li, Y.-P. Li, J. Liang, M. Liebenthal, H.-H. Lin, Y.-S. Lin, F. Liu, K.-Y. Liu, M. Loipersberger, A. Luenser, A. Manjanath, P. Manohar, E. Mansoor, S. F. Manzer, S.-P. Mao, A. V. Marenich, T. Markovich, S. Mason, S. A. Maurer, P. F. McLaughlin, M. F. S. J. Menger, J.-M. Mewes, S. A. Mewes, P. Morgante, J. W. Mullinax, K. J. Oosterbaan, G. Paran, A. C. Paul, S. K. Paul, F. Pavošević, Z. Pei, S. Prager, E. I. Proynov, Á. Rák, E. Ramos-Cordoba, B. Rana, A. E. Rask, A. Rettig, R. M. Richard, F. Rob, E. Rossomme, T. Scheele, M. Scheurer, M. Schneider, N. Sergueev, S. M. Sharada, W. Skomorowski, D. W. Small, C. J. Stein, Y.-C. Su, E. J. Sundstrom, Z. Tao, J. Thirman, G. J. Tornai, T. Tsuchimochi, N. M. Tubman, S. P. Veccham, O. Vydrov, J. Wenzel, J. Witte, A. Yamada, K. Yao, S. Yeganeh, S. R. Yost, A. Zech, I. Y. Zhang, X. Zhang, Y. Zhang, D. Zuev, A. Aspuru-Guzik, A. T. Bell, N. A. Besley, K. B. Bravaya, B. R. Brooks, D. Casanova, J.-D. Chai, S. Coriani, C. J. Cramer, G. Cserey, A. E. DePrince, R. A. DiStasio, A. Dreuw, B. D. Dunietz, T. R. Furlani, W. A. Goddard, S. Hammes-Schiffer, T. Head-Gordon, W. J. Hehre, C.-P. Hsu, T.-C. Jagau, Y. Jung, A. Klamt, J. Kong, D. S. Lambrecht, W. Liang, N. J. Mayhall, C. W. McCurdy, J. B. Neaton, C. Ochsenfeld, J. A. Parkhill, R. Peverati, V. A. Rassolov, Y. Shao, L. V. Slipchenko, T. Stauch, R. P. Steele, J. E. Subotnik, A. J. W. Thom, A. Tkatchenko, D. G. Truhlar, T. Van Voorhis, T. A. Wesolowski, K. B. Whaley, H. L. Woodcock, P. M. Zimmerman, S. Faraji, P. M. W. Gill, M. Head-Gordon, J. M. Herbert, A. I. Krylov, *J.*

Chem. Phys. **2021**, *155*, 084801.

[12] P. J. Stephens, F. J. Devlin, C. F. Chabalowski, M. J. Frisch, *J. Phys. Chem.* **1994**, *98*, 11623–11627.

[13] R. Krishnan, J. S. Binkley, R. Seeger, J. A. Pople, *J. Chem. Phys.* **1980**, *72*, 650–654.

[14] Y.-S. Lin, G.-D. Li, S.-P. Mao, J.-D. Chai, *J. Chem. Theory Comput.* **2013**, *9*, 263–272.

[15] R. A. Kendall, T. H. Dunning, R. J. Harrison, *J. Chem. Phys.* **1992**, *96*, 6796–6806.

[16] A. V. Marenich, C. J. Cramer, D. G. Truhlar, *J. Phys. Chem. B* **2009**, *113*, 6378–6396.

[17] P. Pracht, S. Grimme, C. Bannwarth, F. Bohle, S. Ehlert, G. Feldmann, J. Gorges, M. Müller, T. Neudecker, C. Plett, S. Spicher, P. Steinbach, P. A. Wesołowski, F. Zeller, *J. Chem. Phys.* **2024**, *160*, 114110.

[18] W. Humphrey, A. Dalke, K. Schulten, *J. Mol. Graph.* **1996**, *14*, 33–38.

[19] Rigaku Oxford Diffraction, *CrysAlisPro*, Version 1.171.43.95a, Rigaku Oxford Diffraction Ltd., Yarnton, Oxfordshire, England, **2023**.

[20] Rigaku Oxford Diffraction, *CrysAlisPro*, Version 1.171.43.130a, Rigaku Oxford Diffraction Ltd., Yarnton, Oxfordshire, England, **2024**.

[21] Rigaku Oxford Diffraction, *CrysAlisPro*, Version 1.171.44.118a, Rigaku Oxford Diffraction Ltd., Yarnton, Oxfordshire, England, **2025**.

[22] Rigaku Oxford Diffraction, *CrysAlisPro*, Version 1.171.44.120a, Rigaku Oxford Diffraction Ltd., Yarnton, Oxfordshire, England, **2025**.

8. ^{1}H, ^{13}C, and 2D NMR Spectra of Synthesized Compounds

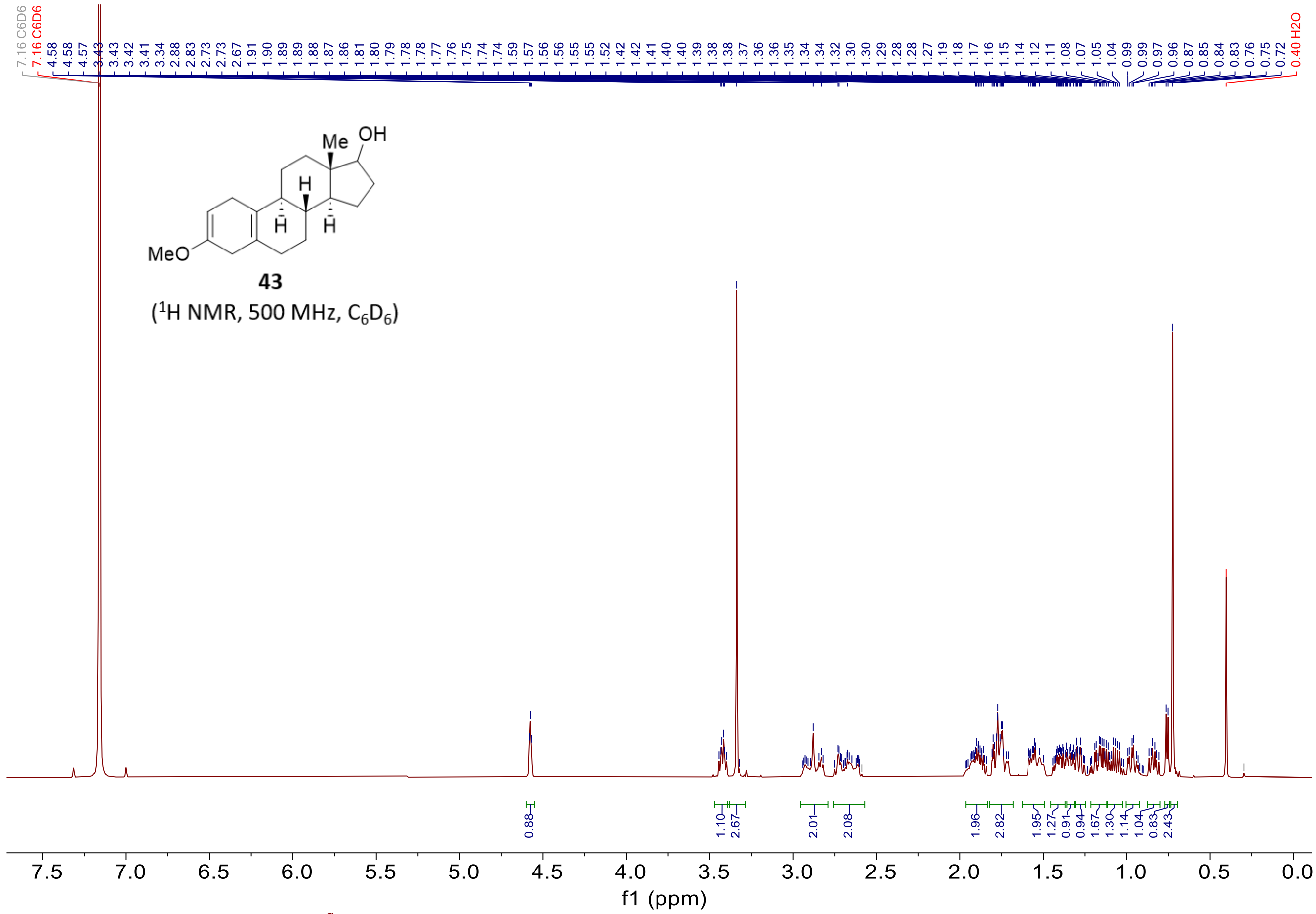

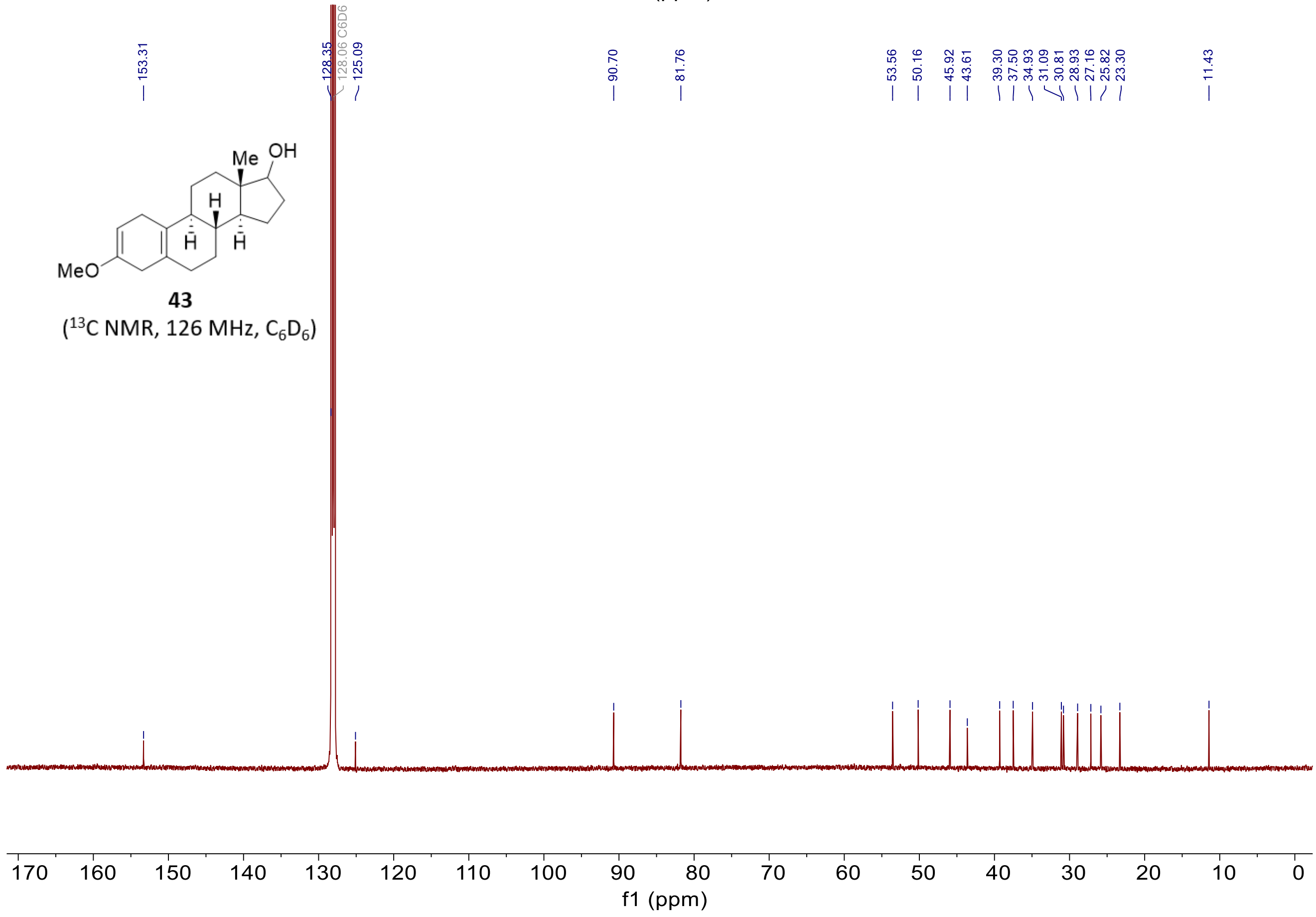

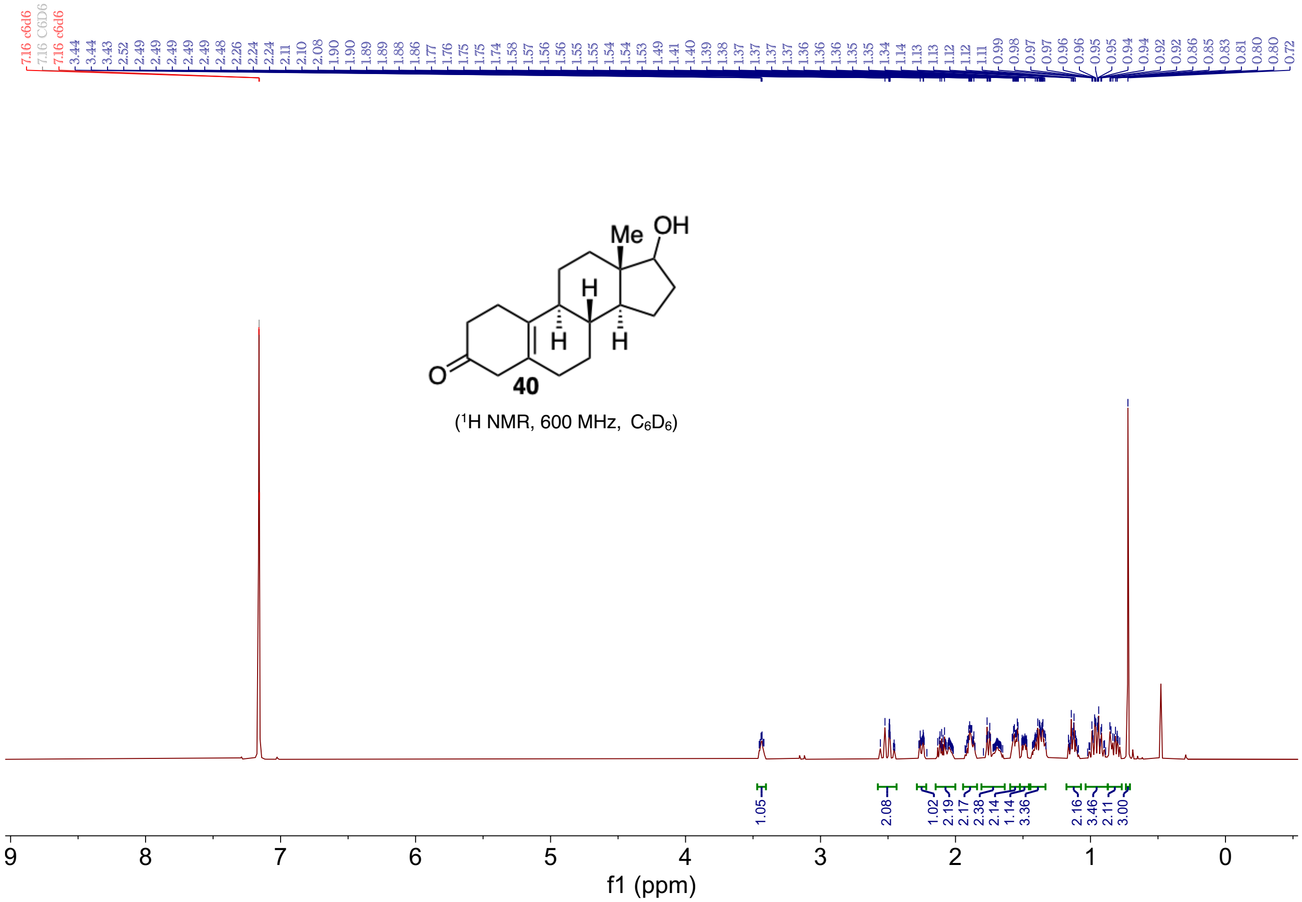
7.16 c6d6
7.16 C6D6
7.16 c6d6
3.44
3.44
3.43
2.52
2.49
2.49
2.49
2.49
2.49
2.48
2.26
2.24
2.24
2.11
2.10
2.08
1.90
1.90
1.89
1.89
1.88
1.86
1.77
1.76
1.75
1.75
1.74
1.58
1.57
1.56
1.56
1.55
1.55
1.54
1.54
1.53
1.49
1.41
1.40
1.39
1.38
1.37
1.37
1.37
1.37
1.36
1.36
1.36
1.35
1.35
1.34
1.14
1.13
1.13
1.13
1.12
1.12
1.11
0.99
0.98
0.97
0.97
0.96
0.96
0.95
0.95
0.94
0.94
0.92
0.92
0.86
0.85
0.83
0.81
0.80
0.80
0.72
Me
OH
H
H
H
O
40
(^{1}H NMR, 600 MHz, C_6D_6)
1.05
2.08
1.02
2.19
2.17
2.38
2.14
1.14
3.36
2.16
3.46
2.11
3.00
9
8
7
6
5
4
3
2
1
0
f1 (ppm)

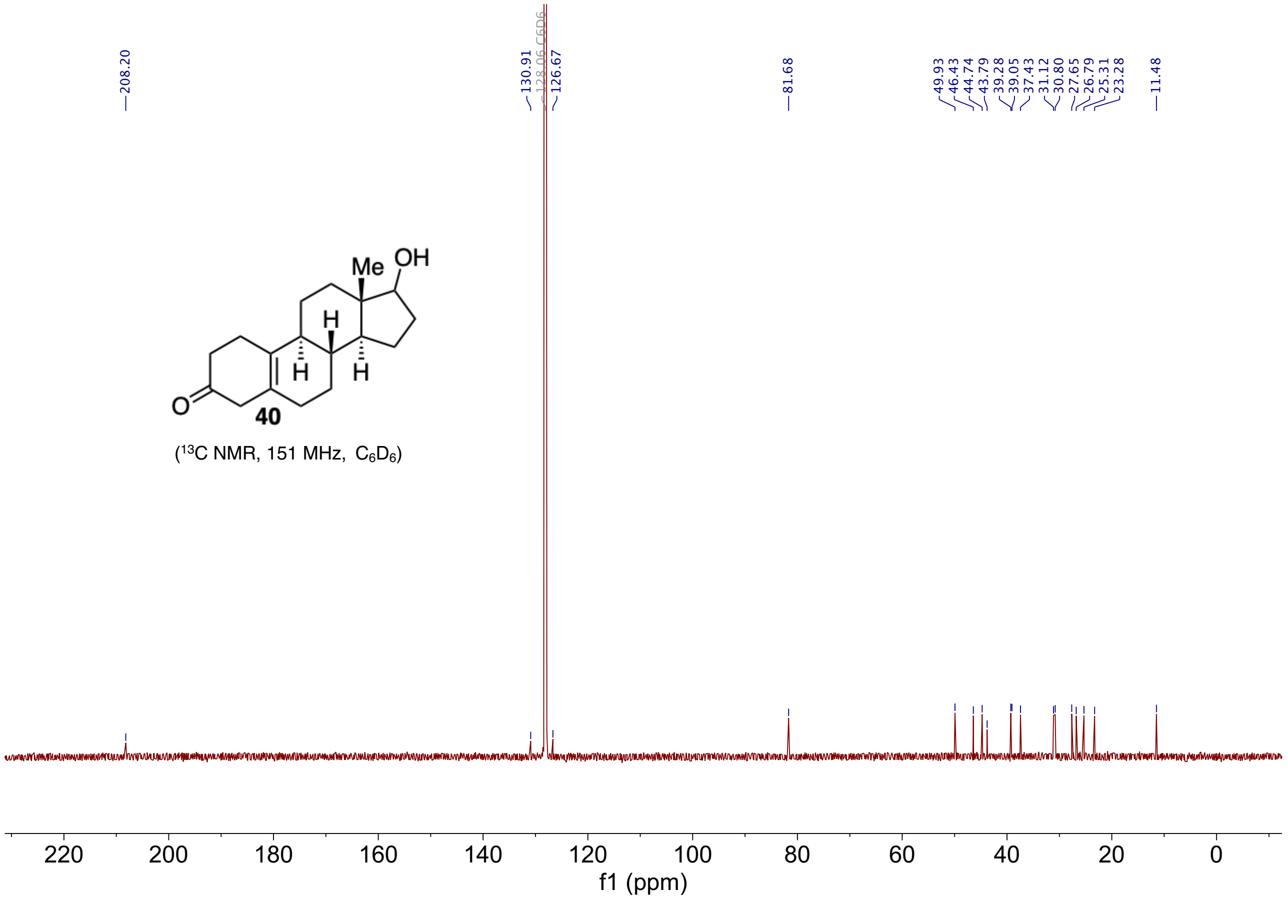
208.20
130.91
128.06 C6D6
126.67
81.68
49.93
46.43
44.74
43.79
39.28
39.05
37.43
31.12
30.80
27.65
26.79
25.31
23.28
11.48
Me
OH
H
H
H
O
40
(^{13}C NMR, 151 MHz, C_6D_6)
220
200
180
160
140
120
100
80
60
40
20
0
f1 (ppm)

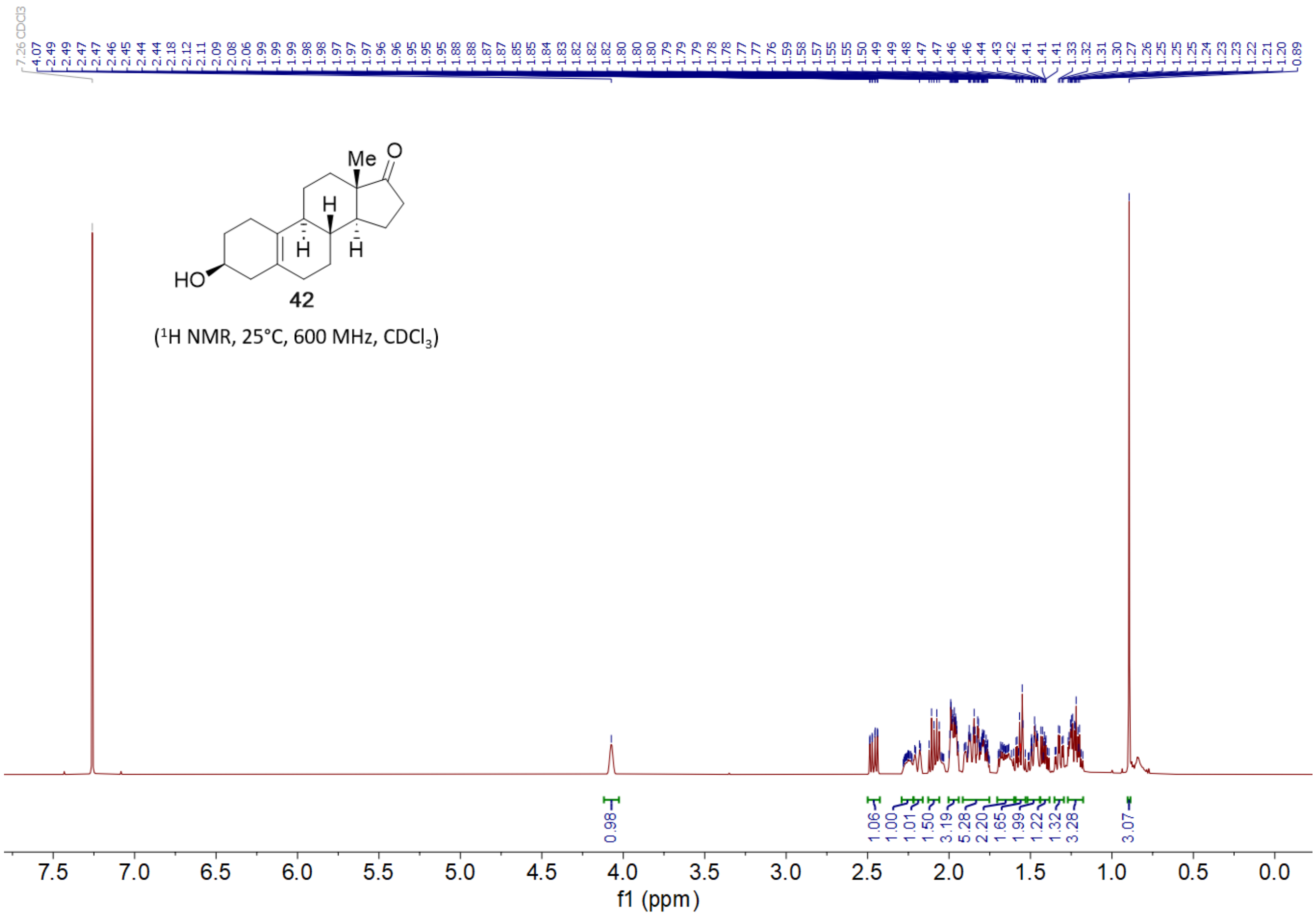
7.26 CDCl3
4.07
2.49 2.49 2.47 2.47 2.46 2.45 2.44 2.44 2.18 2.12 2.11 2.09 2.08 2.06 1.99 1.99 1.99 1.98 1.98 1.97 1.97 1.97 1.96 1.96 1.95 1.95 1.95 1.88 1.88 1.87 1.87 1.85 1.85 1.84 1.83 1.82 1.82 1.82 1.80 1.80 1.80 1.79 1.79 1.79 1.78 1.78 1.77 1.77 1.76 1.59 1.58 1.57 1.55 1.55 1.55 1.50 1.49 1.49 1.48 1.47 1.47 1.46 1.46 1.44 1.43 1.42 1.41 1.41 1.41 1.33 1.32 1.31 1.30 1.27 1.26 1.25 1.25 1.24 1.23 1.23 1.23 1.22 1.21 1.20 0.89
Me
O
H
H
H
HO
42
(1H NMR, 25°C, 600 MHz, CDCl3)
0.98
1.06 1.00 1.01 1.50 3.19 5.28 2.20 1.65 1.99 1.22 1.32 3.28
3.07
7.5 7.0 6.5 6.0 5.5 5.0 4.5 4.0 3.5 3.0 2.5 2.0 1.5 1.0 0.5 0.0
f1 (ppm)

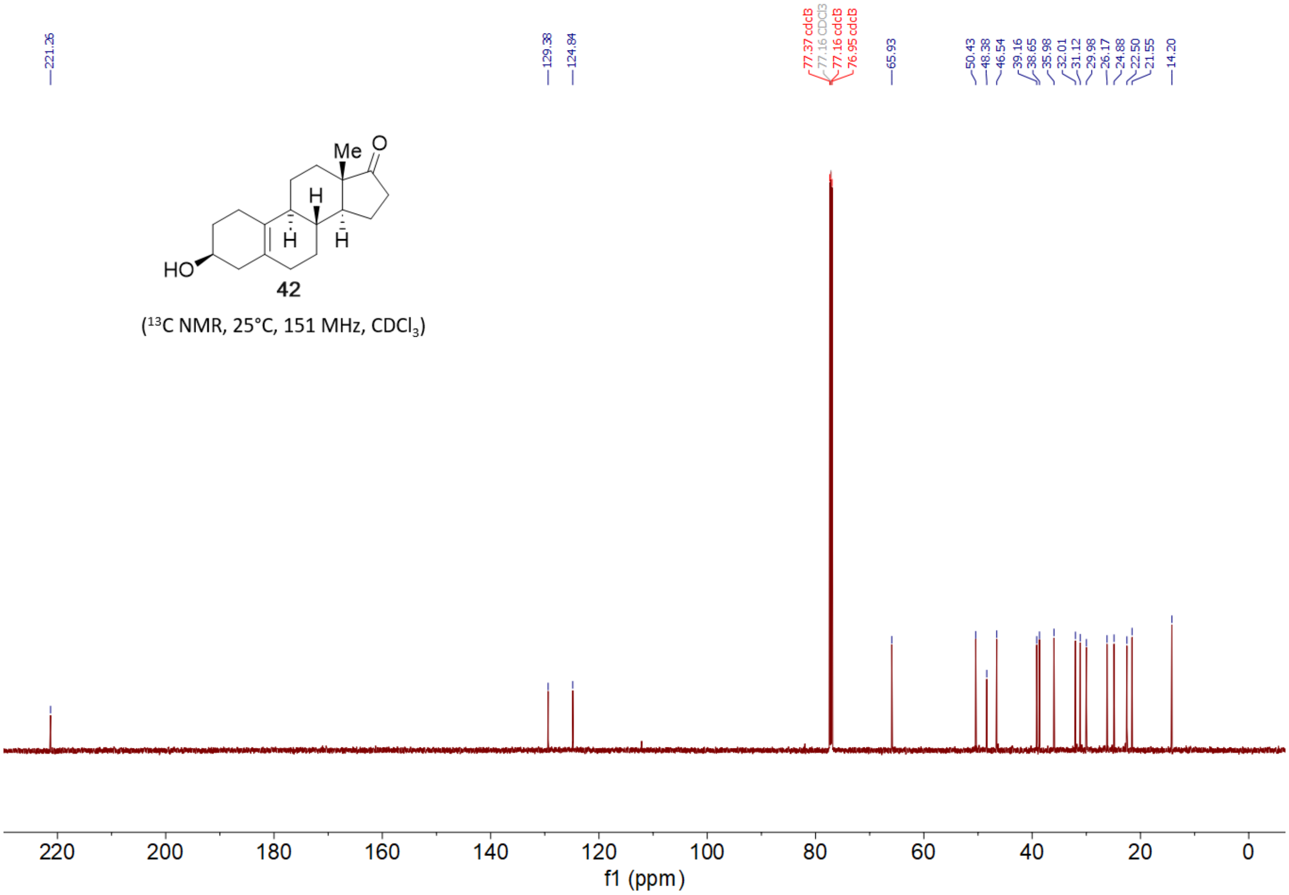
221.26
129.38
124.84
77.37 cdcl3
77.16 CDCl3
77.16 cdcl3
76.95 cdcl3
65.93
50.43 48.38 46.54 39.16 38.65 35.98 32.01 31.12 29.98 26.17 24.88 22.50 21.55
14.20
Me
O
H
H
H
HO
42
(13C NMR, 25°C, 151 MHz, CDCl3)
220 200 180 160 140 120 100 80 60 40 20 0
f1 (ppm)

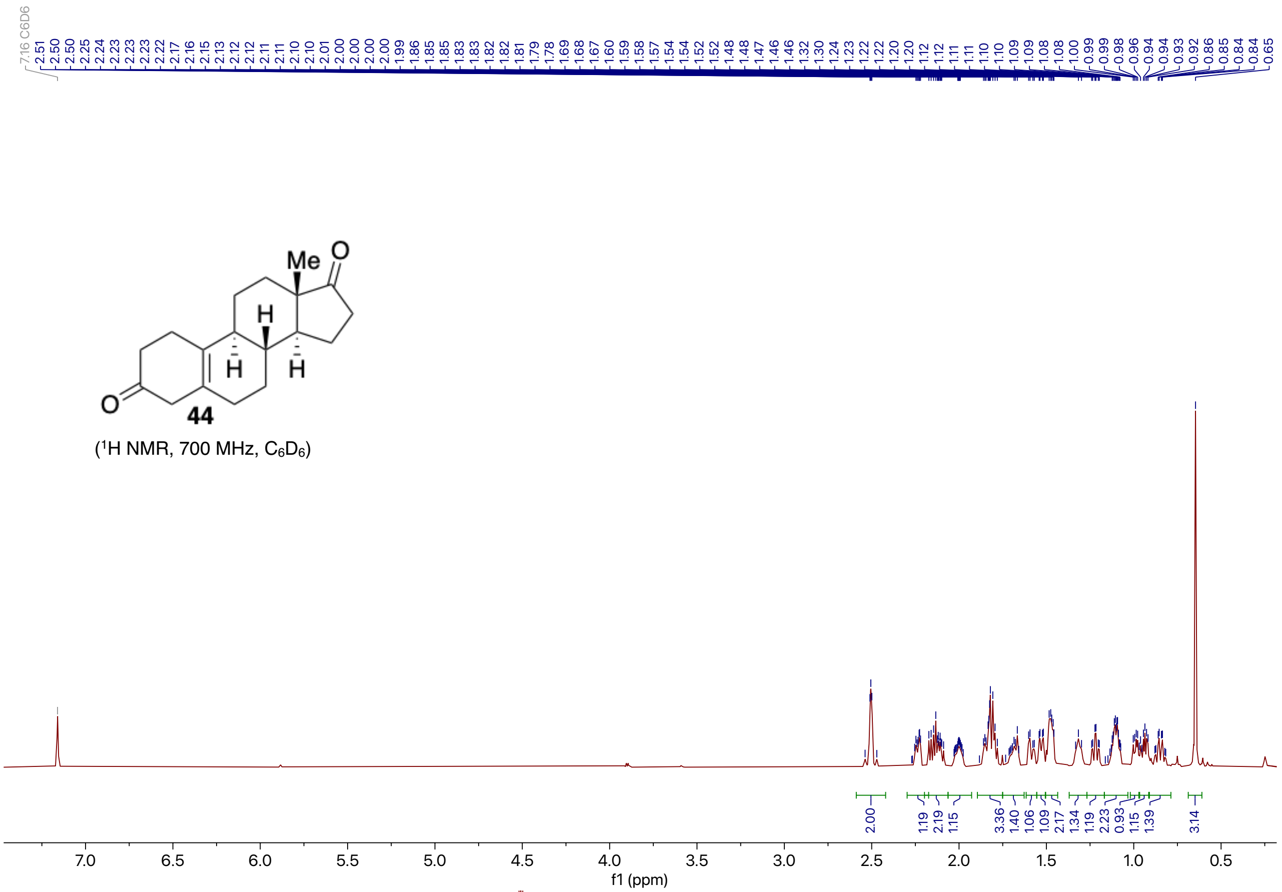

(¹H NMR, 700 MHz, C_6D_6)

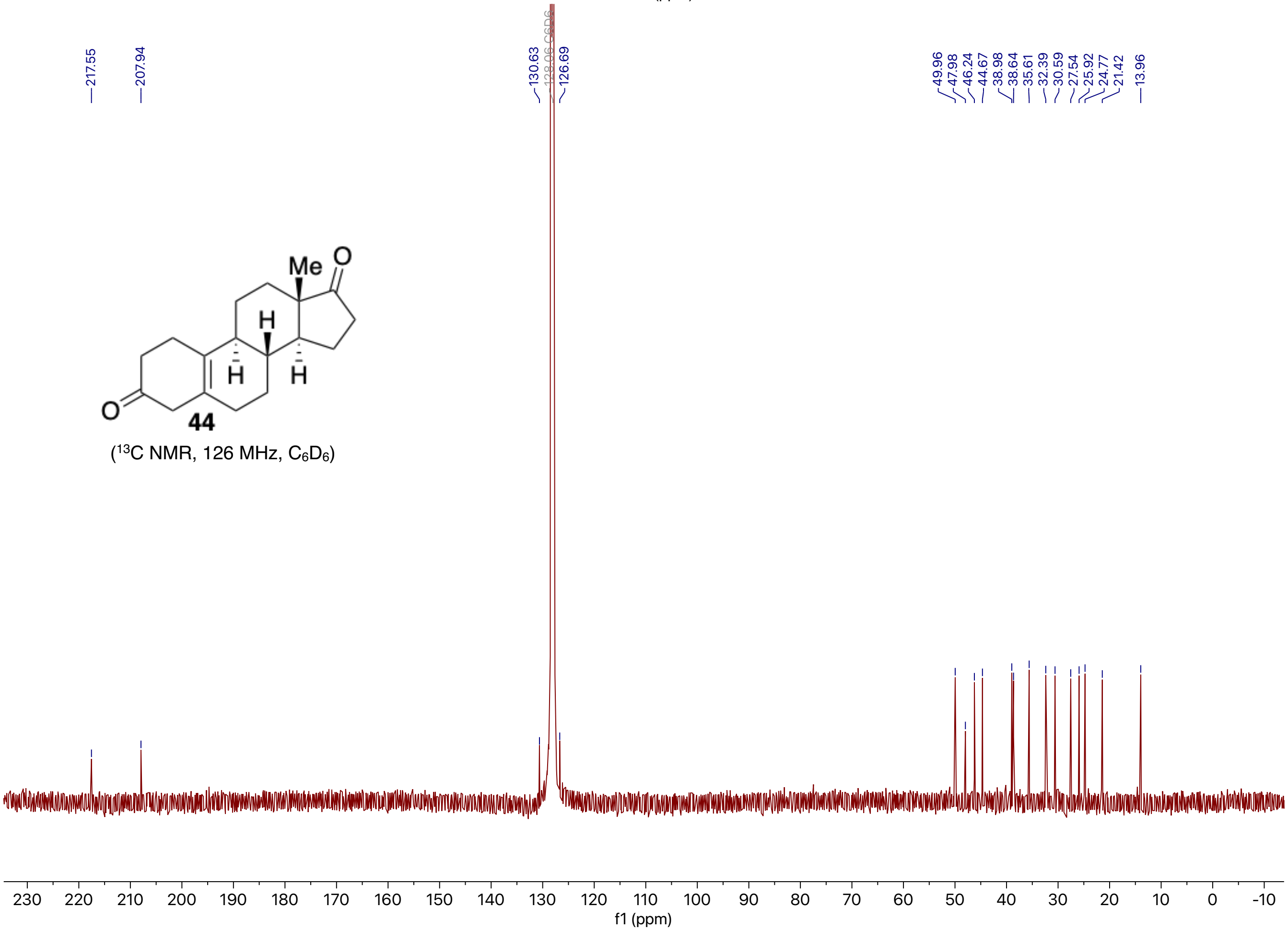

(¹³C NMR, 126 MHz, C_6D_6)

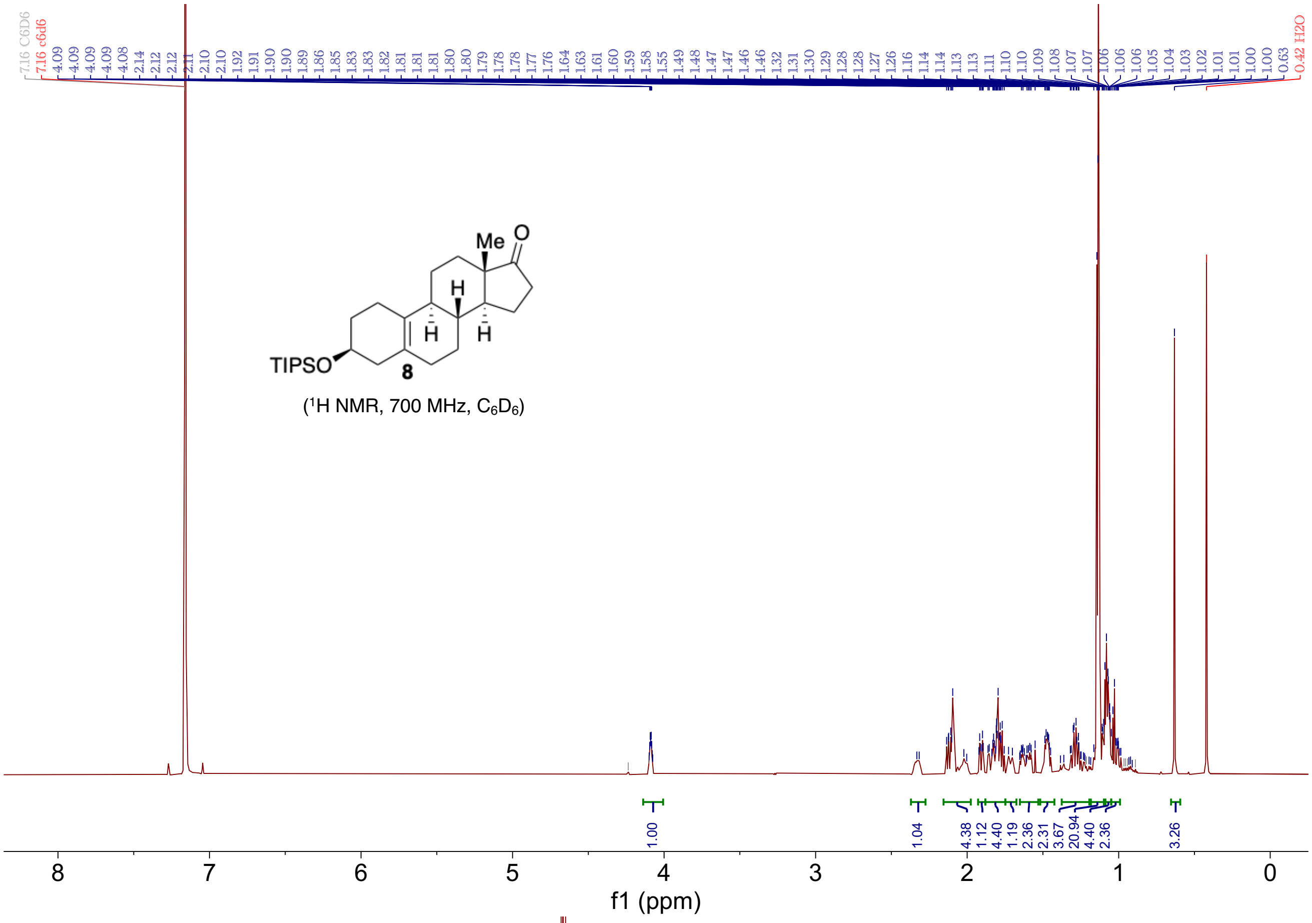
TIPSO
Me
O
H
H
H
8
(1H NMR, 700 MHz, C6D6)
7.16 C6D6
7.16 c6d6
0.42 H2O
1.00
1.04
4.38
1.12
4.40
1.19
2.36
2.31
3.67
20.94
4.40
2.36
3.26
f1 (ppm)

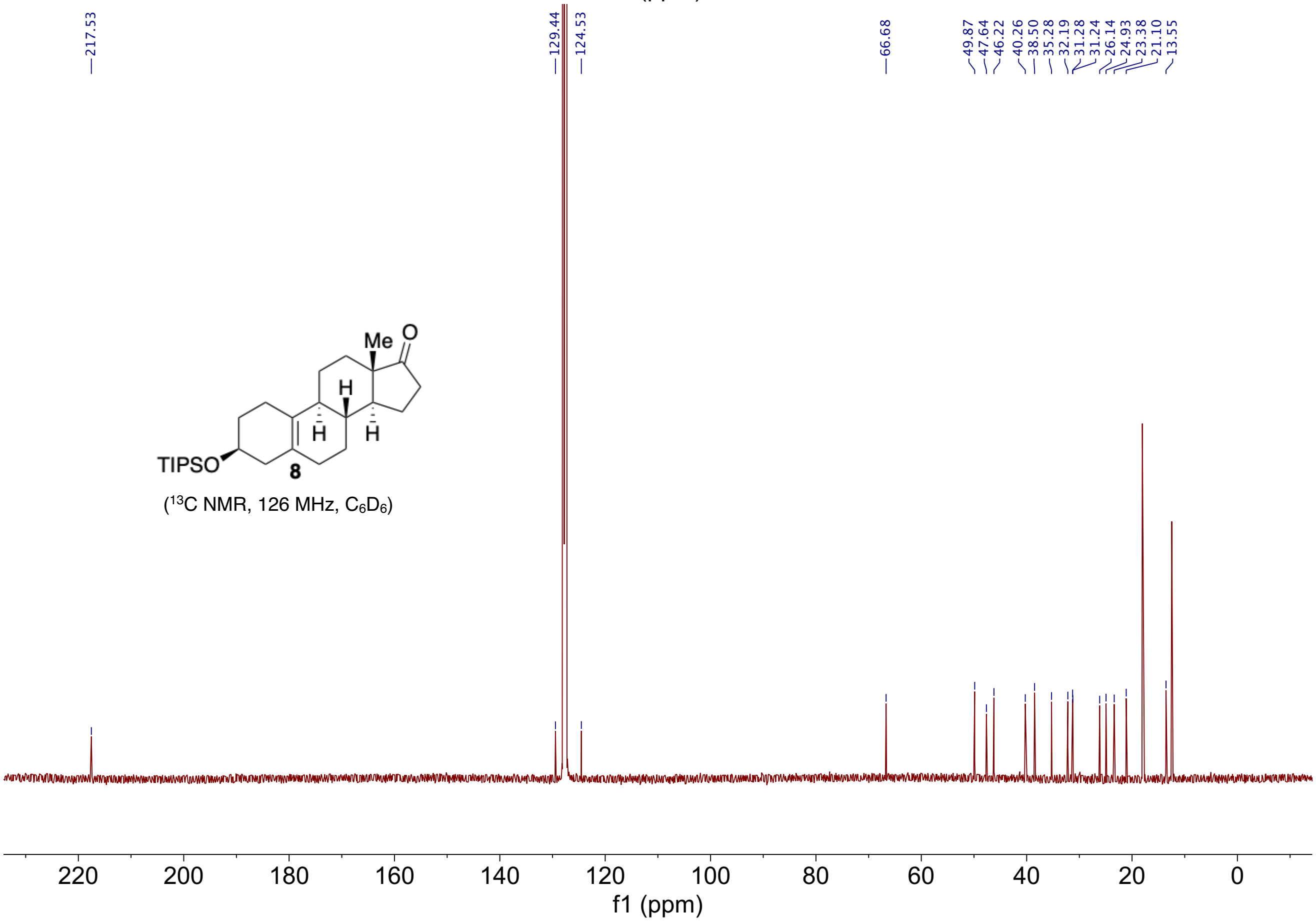
217.53
129.44
124.53
66.68
49.87
47.64
46.22
40.26
38.50
35.28
32.19
31.28
31.24
26.14
24.93
23.38
21.10
13.55
TIPSO
Me
O
H
H
H
8
(13C NMR, 126 MHz, C6D6)
f1 (ppm)

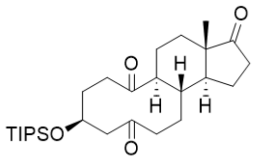

18

(^{1}H NMR, 25°C, 500 MHz, C_6D_6)

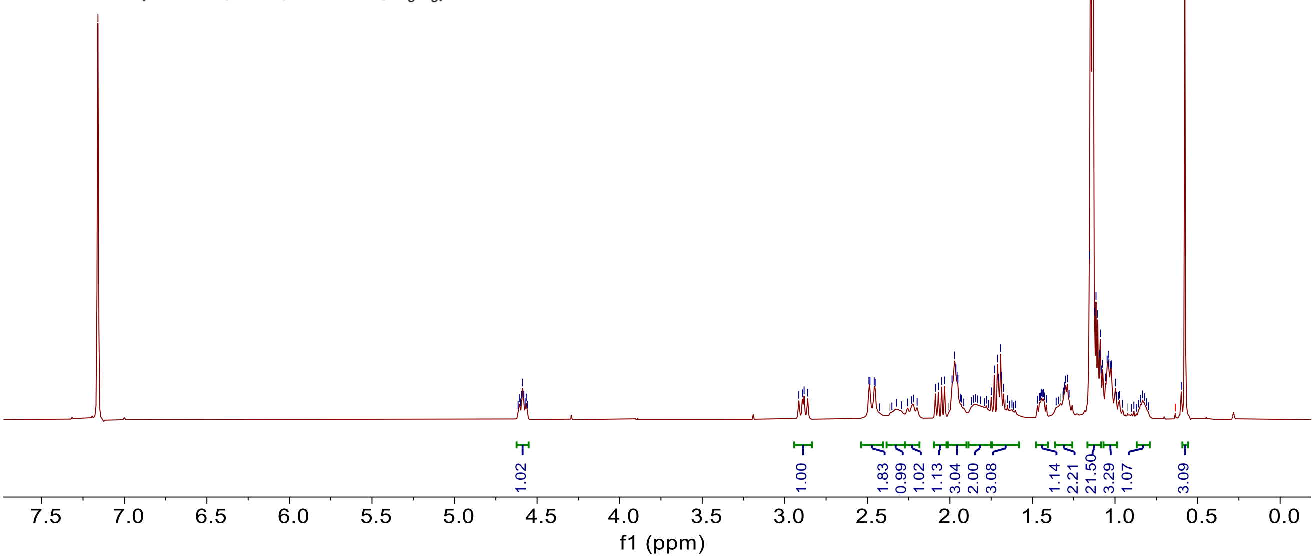

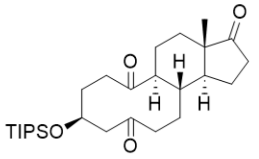

18

(^{13}C NMR, 25°C, 126 MHz, C_6D_6)

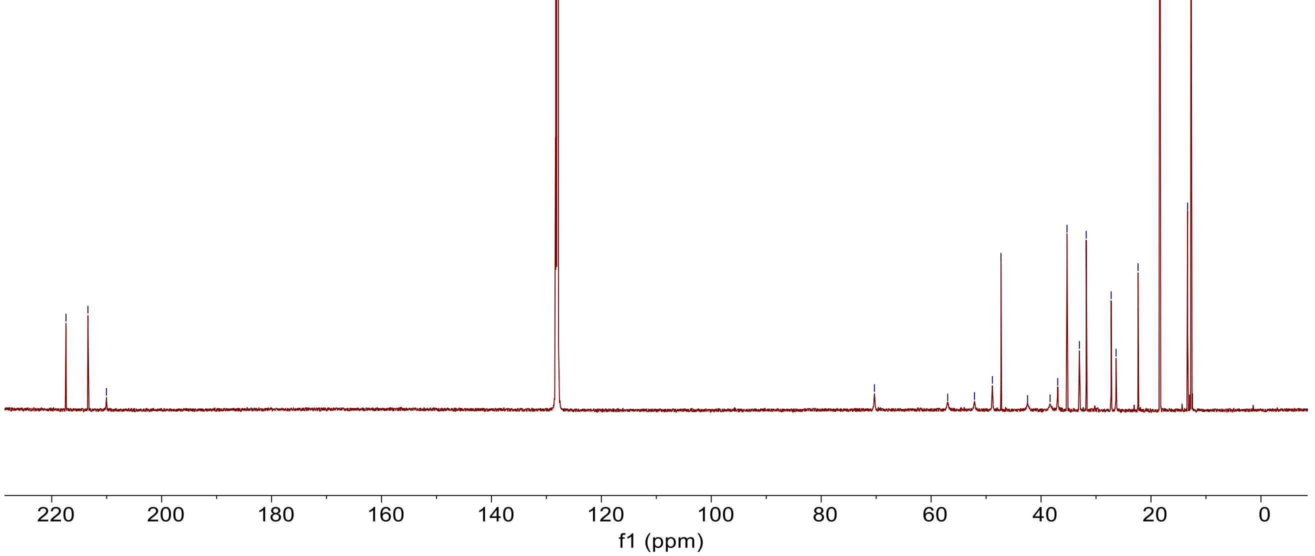

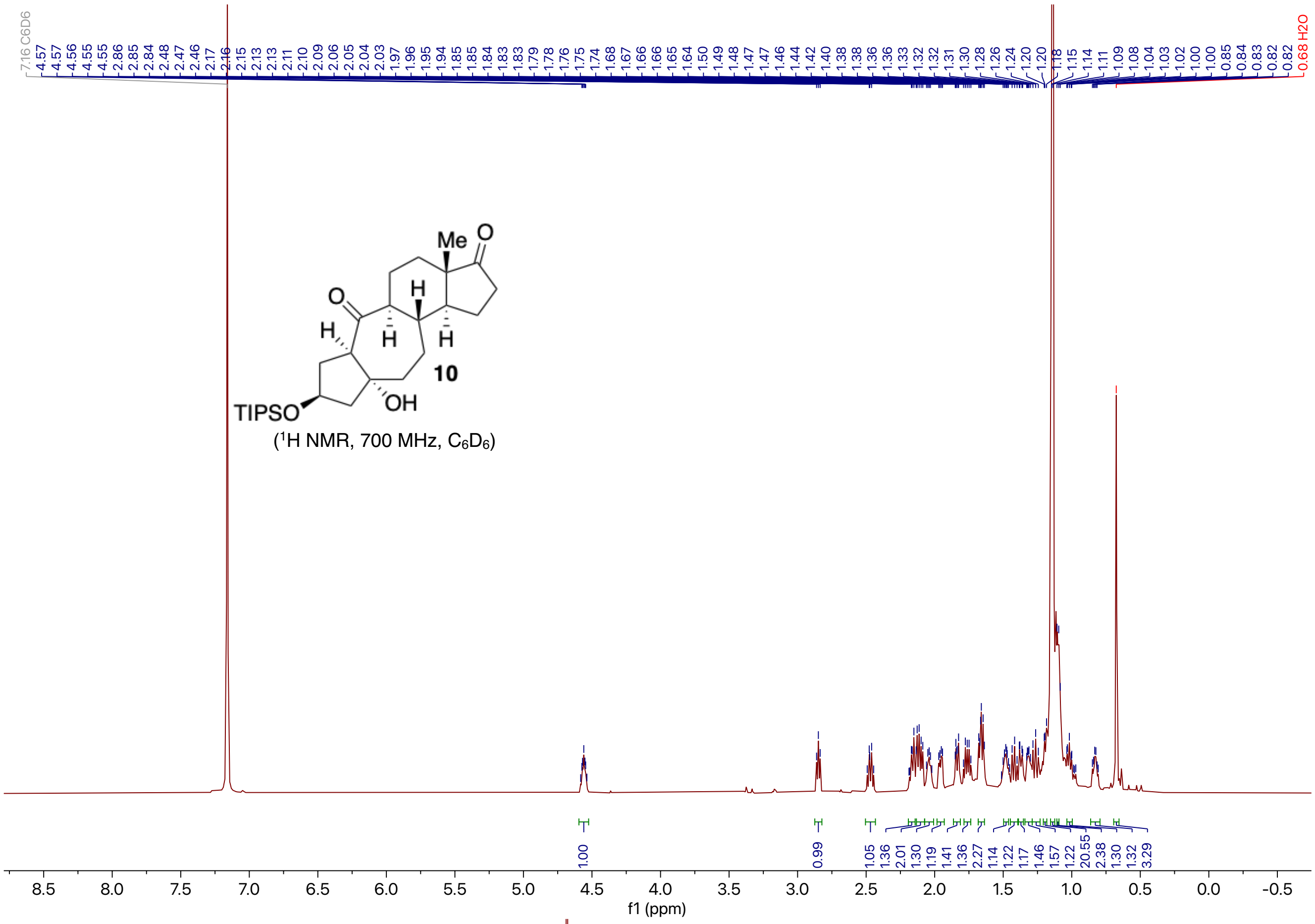

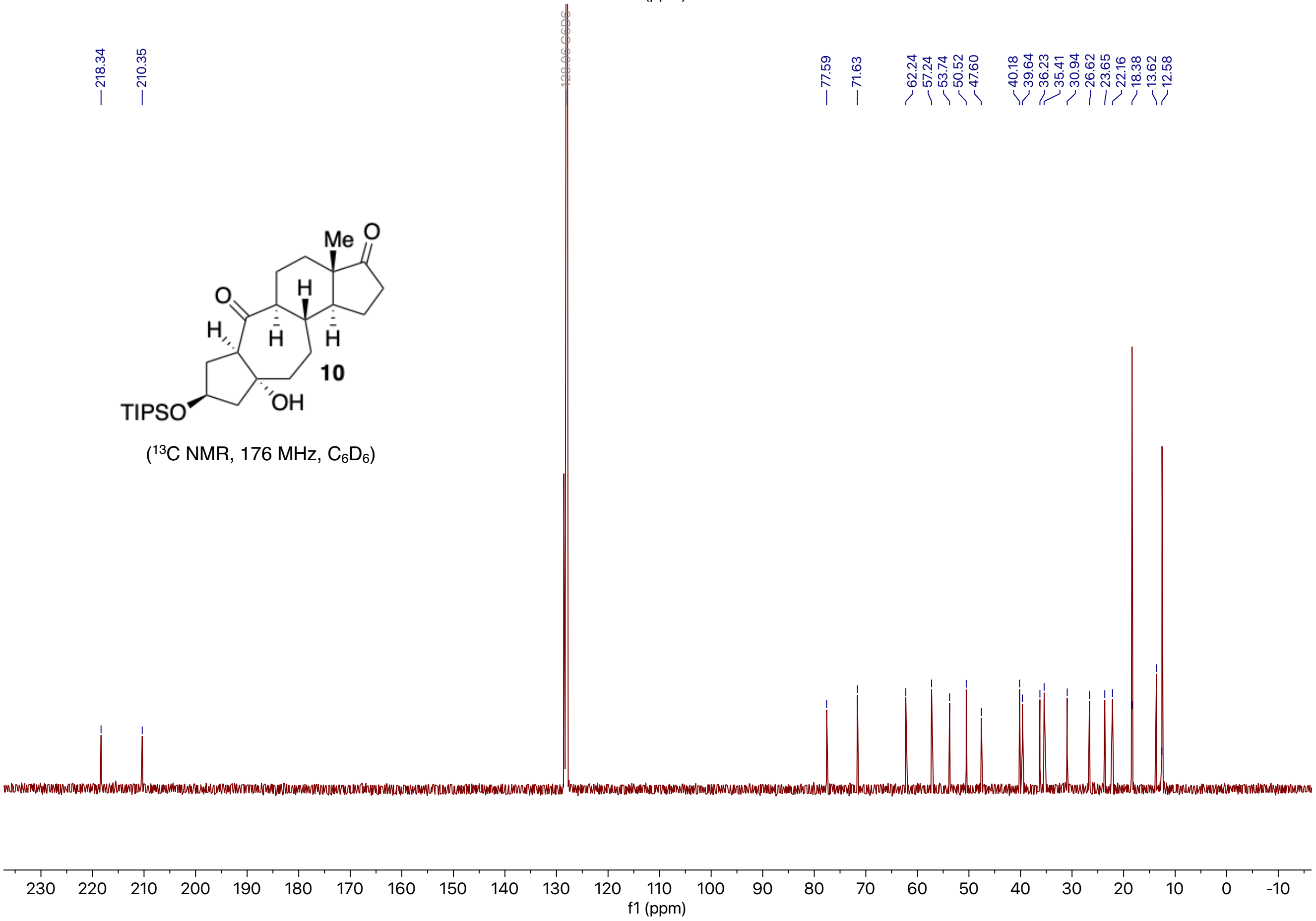

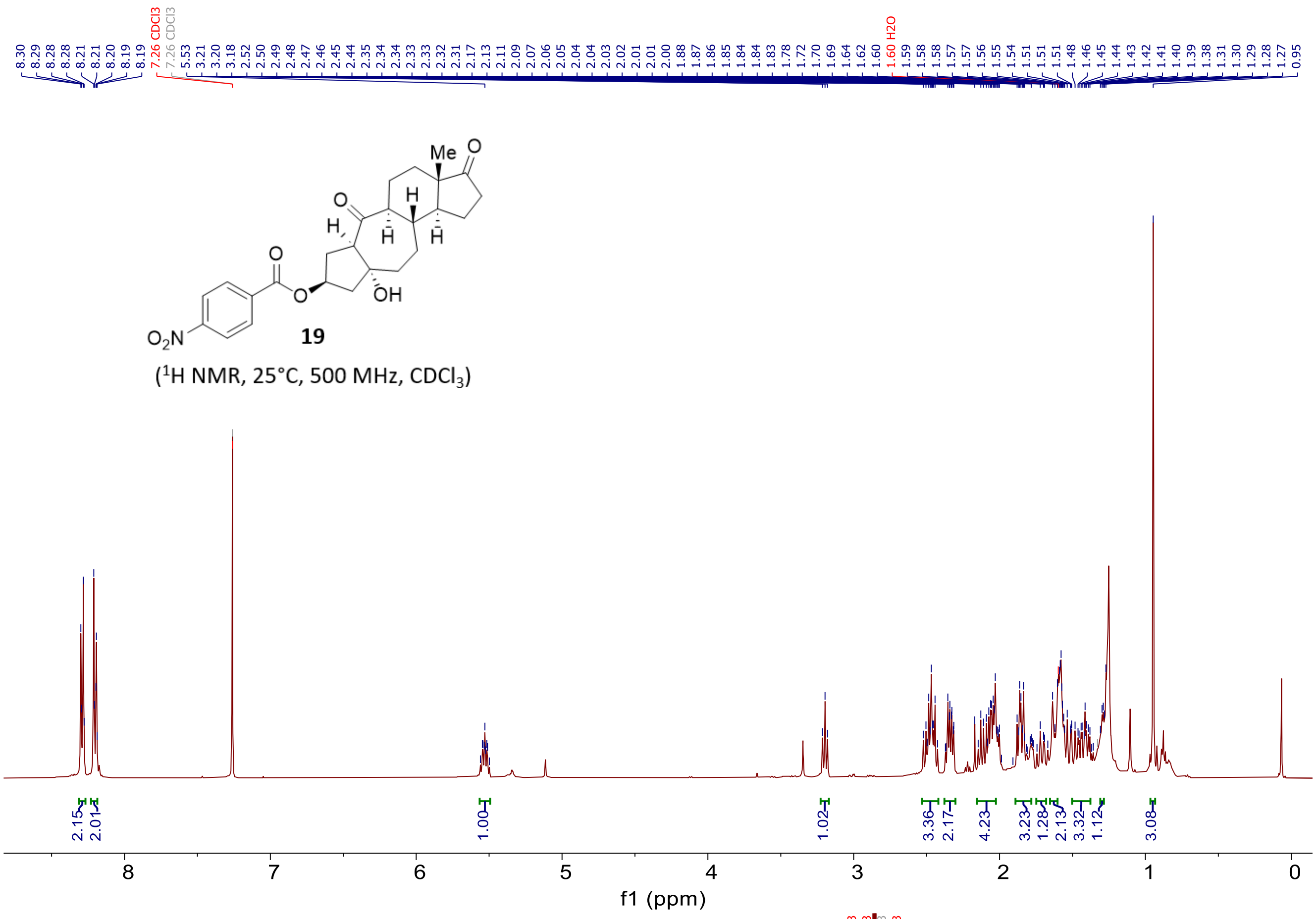
19
(¹H NMR, 25°C, 500 MHz, CDCl₃)
f1 (ppm)

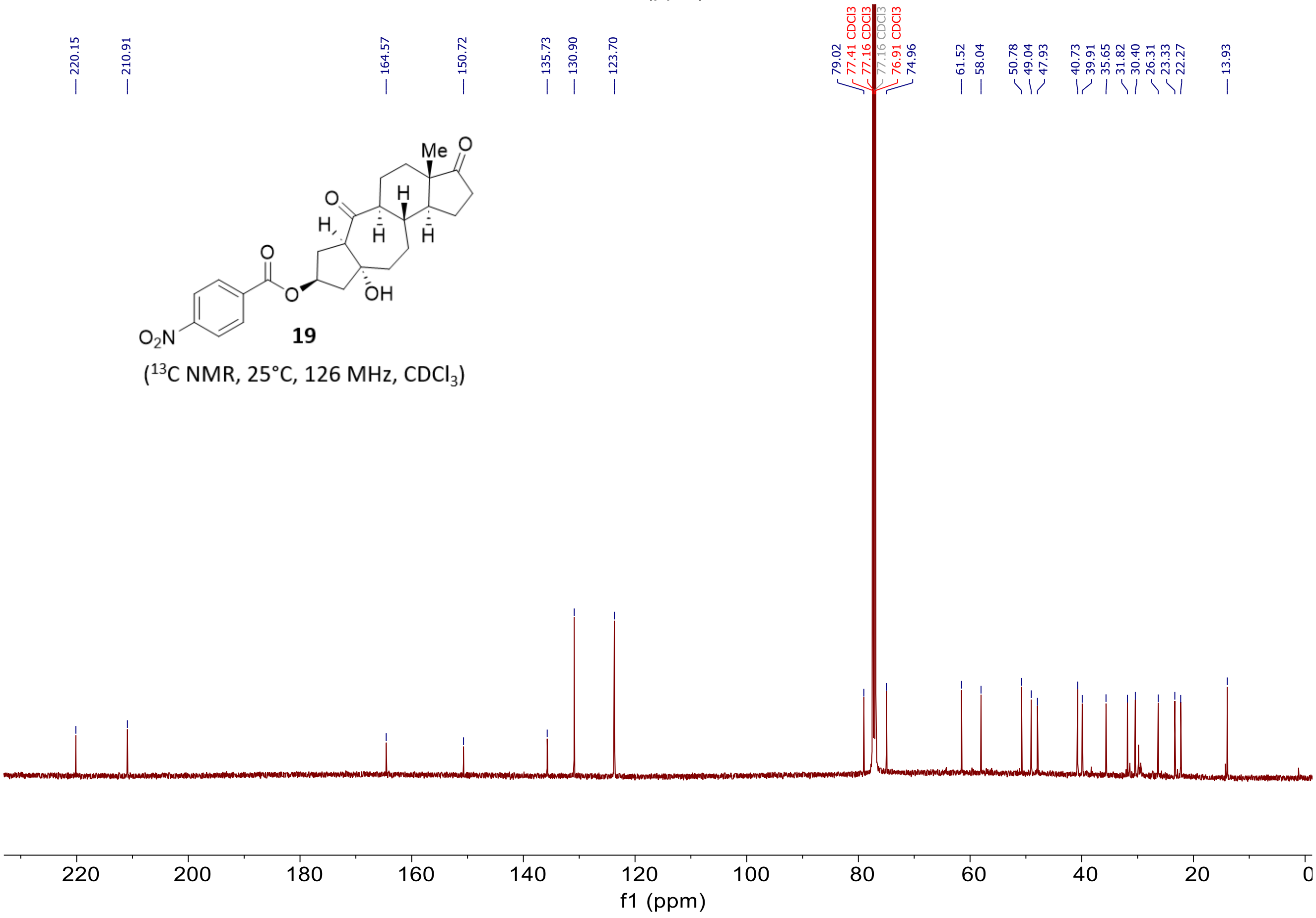
19
(¹³C NMR, 25°C, 126 MHz, CDCl₃)
f1 (ppm)

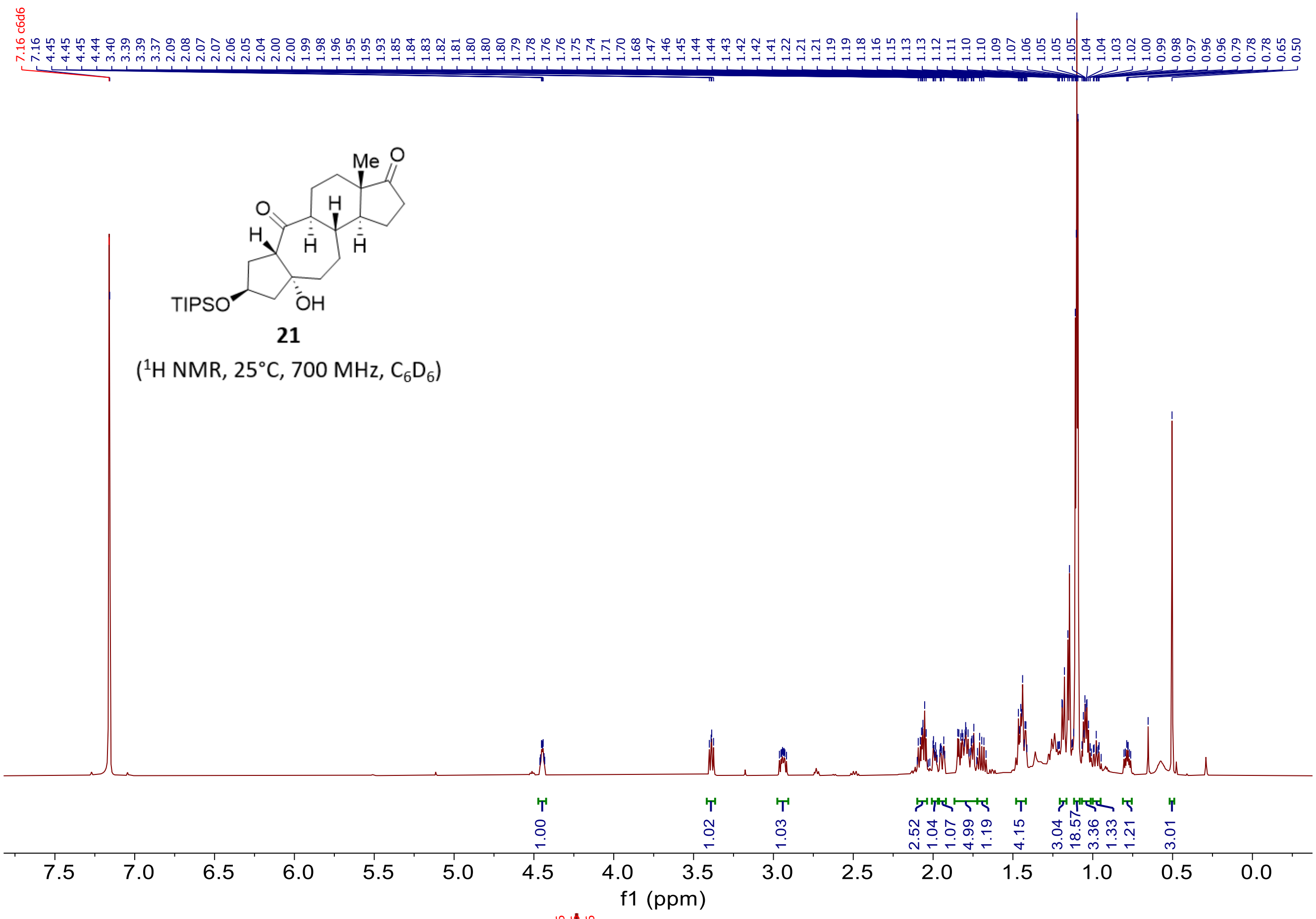
Me
O
H
TIPSO
OH
21
(1H NMR, 25°C, 700 MHz, C6D6)
f1 (ppm)

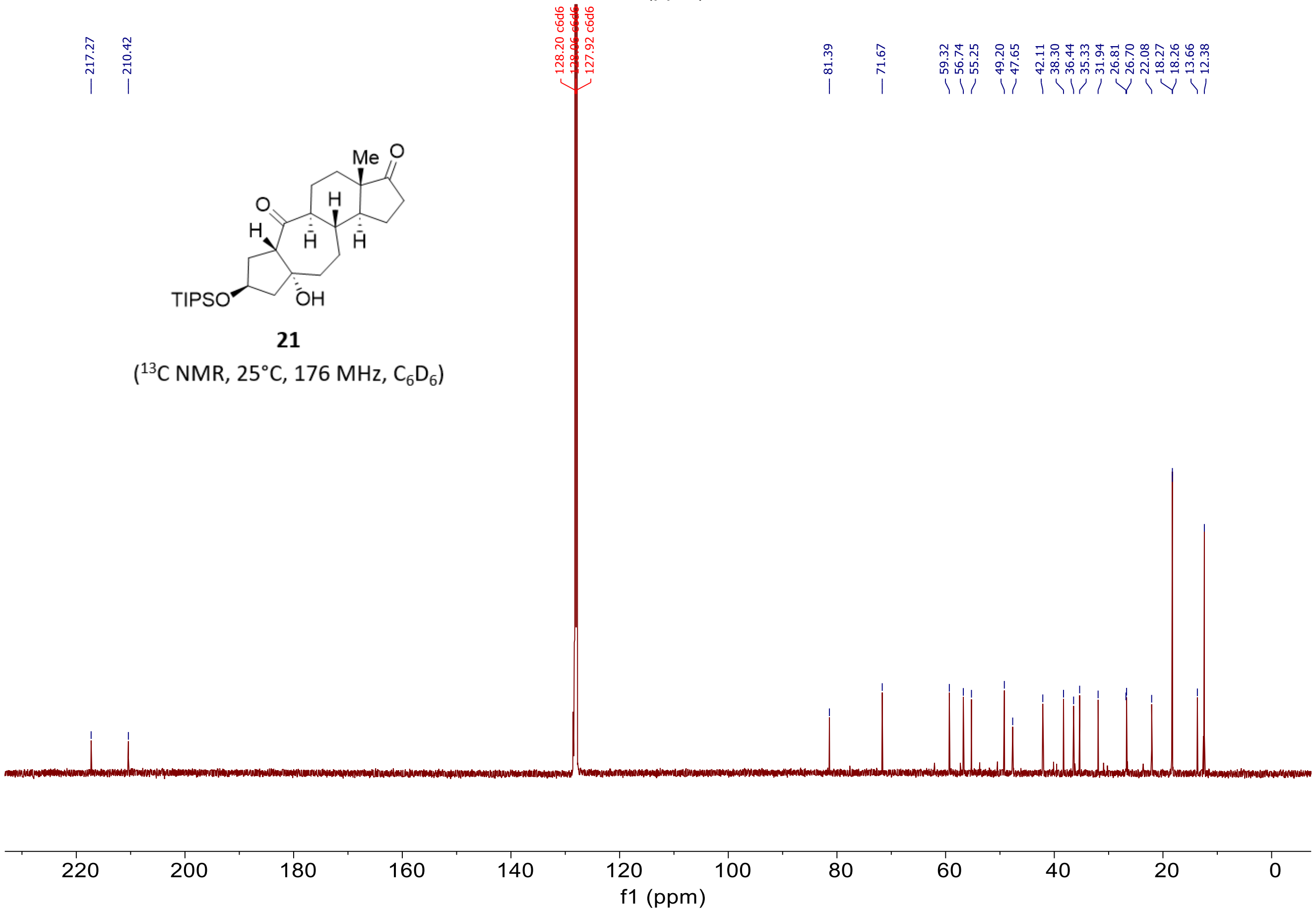
Me
O
H
TIPSO
OH
21
(13C NMR, 25°C, 176 MHz, C6D6)
f1 (ppm)

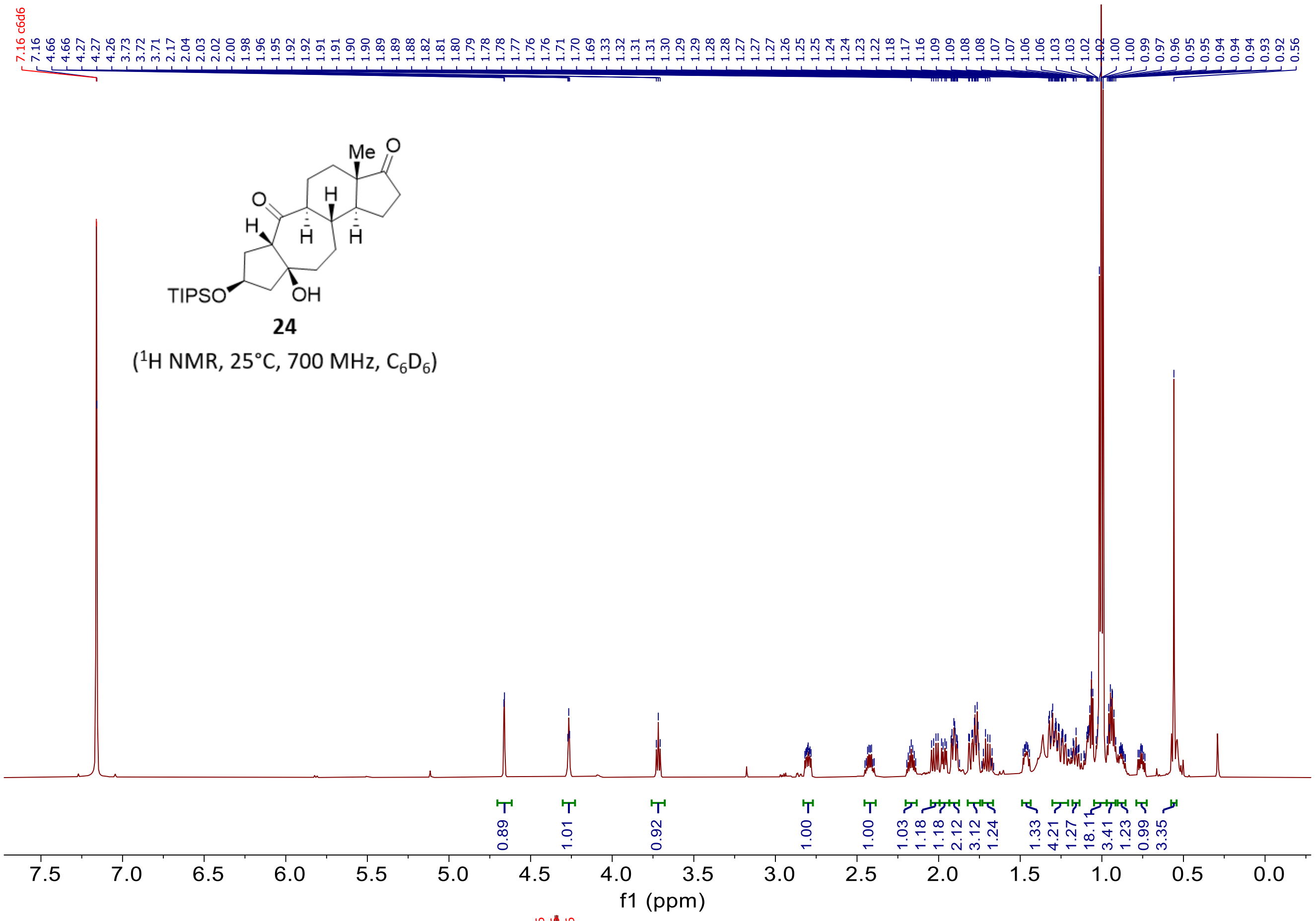
Me
O
O
H
H
H
H
TIPSO
OH
24
(1H NMR, 25°C, 700 MHz, C6D6)
7.16 c6d6
f1 (ppm)

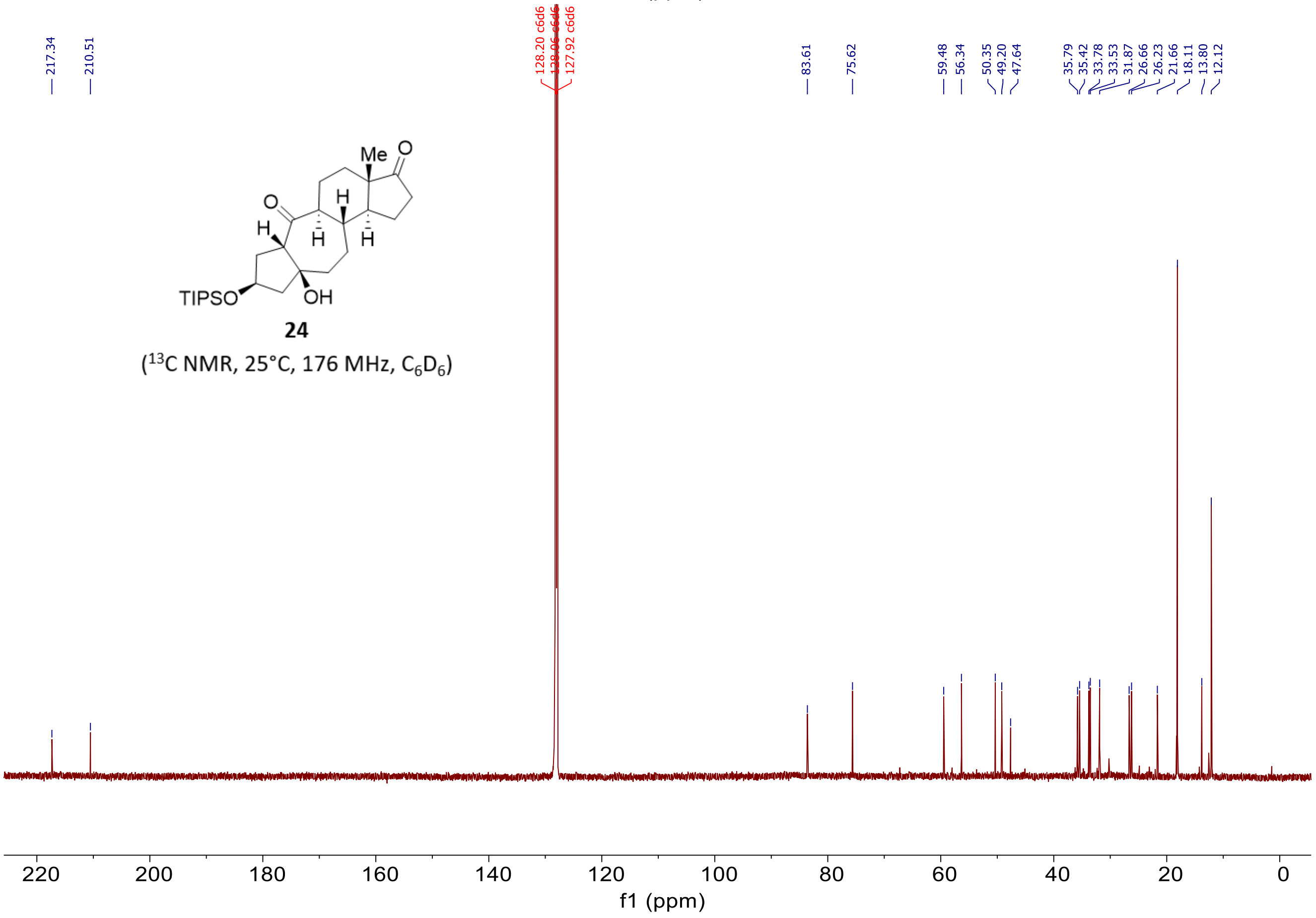
217.34
210.51
128.20 c6d6
128.06 c6d6
127.92 c6d6
83.61
75.62
59.48
56.34
50.35
49.20
47.64
35.79
35.42
33.78
33.53
31.87
26.66
26.23
21.66
18.11
13.80
12.12
Me
O
O
H
H
H
H
TIPSO
OH
24
(13C NMR, 25°C, 176 MHz, C6D6)
f1 (ppm)

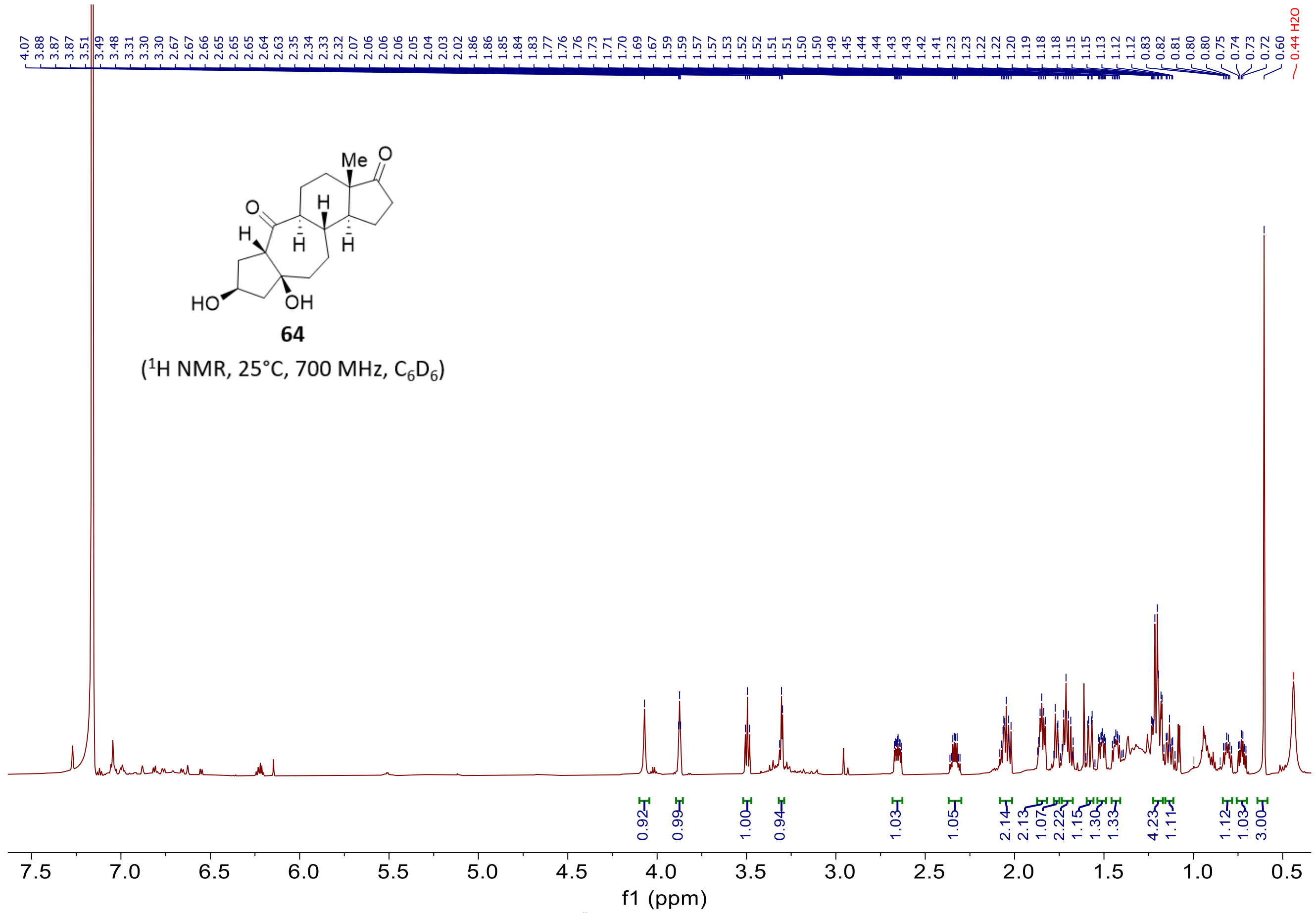
4.07 3.88 3.87 3.87 3.51 3.49 3.48 3.31 3.30 3.30 2.67 2.67 2.66 2.65 2.65 2.65 2.64 2.63 2.35 2.34 2.33 2.32 2.07 2.06 2.06 2.06 2.05 2.04 2.03 2.02 1.86 1.86 1.85 1.84 1.83 1.77 1.76 1.76 1.73 1.71 1.70 1.69 1.67 1.59 1.59 1.57 1.57 1.53 1.52 1.52 1.51 1.51 1.50 1.50 1.49 1.45 1.44 1.44 1.43 1.43 1.42 1.41 1.23 1.23 1.23 1.22 1.22 1.20 1.19 1.18 1.18 1.15 1.15 1.13 1.12 1.12 0.83 0.82 0.81 0.80 0.80 0.75 0.74 0.73 0.72 0.60
0.44 H2O
Me
O
H
O
H
H
H
HO
OH
64
(^{1}H NMR, 25°C, 700 MHz, C_6D_6)
0.92 0.99 1.00 0.94 1.03 1.05 2.14 2.13 1.07 2.22 1.15 1.30 1.33 4.23 1.11 1.12 1.03 3.00
7.5 7.0 6.5 6.0 5.5 5.0 4.5 4.0 3.5 3.0 2.5 2.0 1.5 1.0 0.5
f1 (ppm)

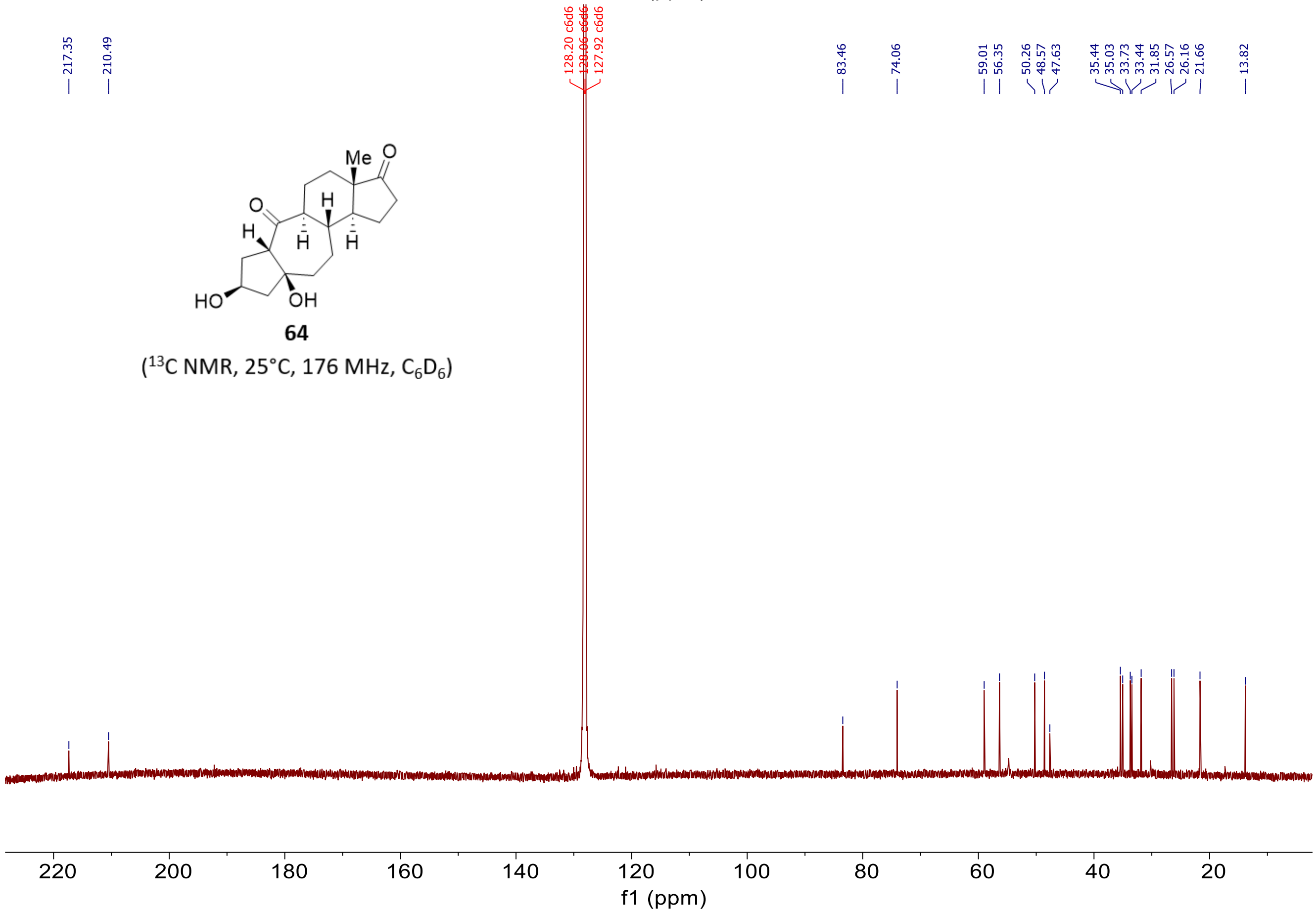
217.35
210.49
128.20 c6d6
128.06 c6d6
127.92 c6d6
83.46
74.06
59.01 56.35 50.26 48.57 47.63
35.44 35.03 33.73 33.44 31.85 26.57 26.16 21.66
13.82
Me
O
H
O
H
H
H
HO
OH
64
(^{13}C NMR, 25°C, 176 MHz, C_6D_6)
220 200 180 160 140 120 100 80 60 40 20
f1 (ppm)

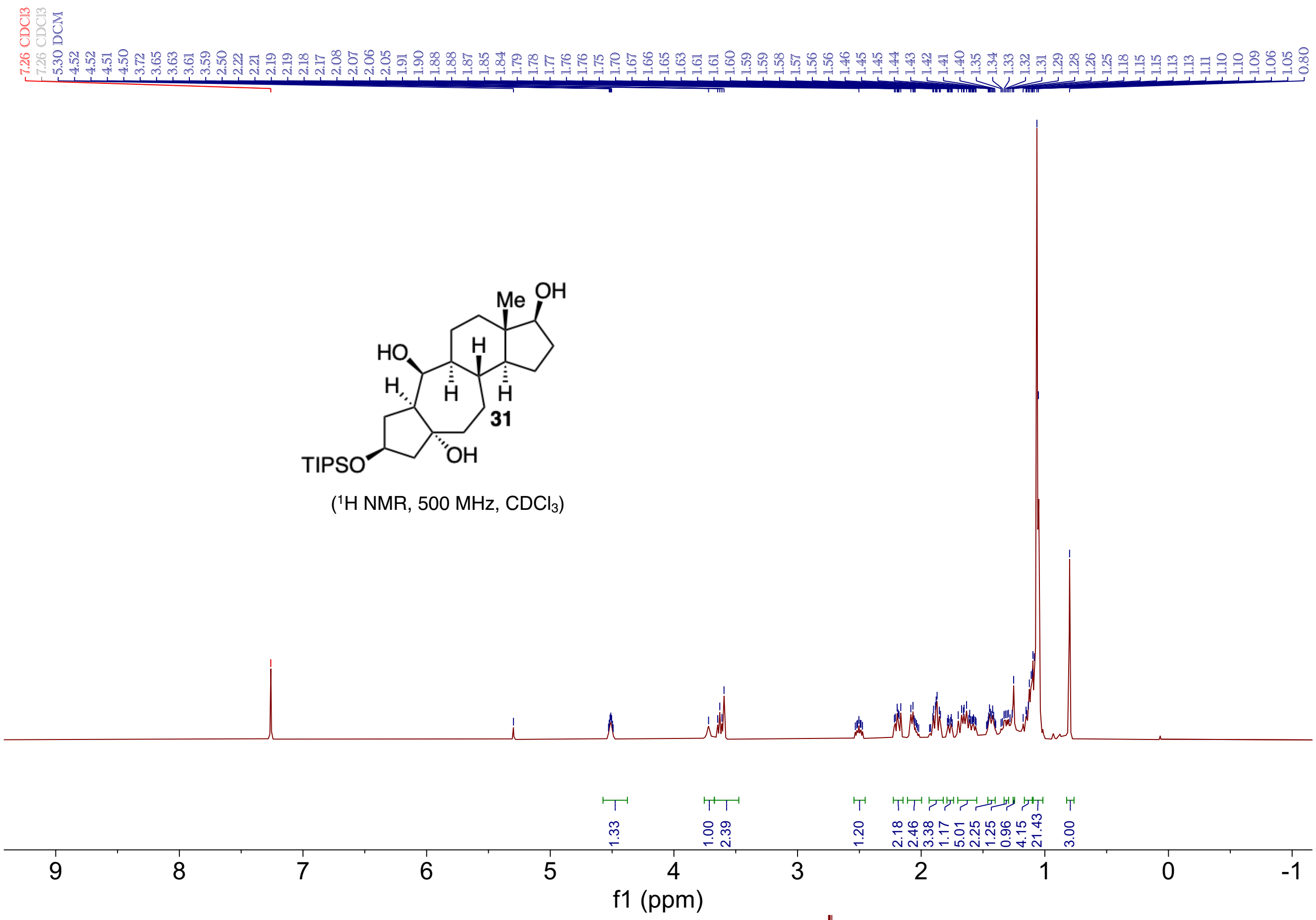
7.26 CDCl3
7.26 CDCl3
5.30 DCM
4.52 4.52 4.51 4.50 3.72 3.65 3.63 3.61 3.59 2.50 2.22 2.21 2.19 2.19 2.18 2.17 2.08 2.07 2.06 2.05 1.91 1.90 1.88 1.88 1.87 1.85 1.84 1.79 1.78 1.77 1.76 1.76 1.75 1.70 1.67 1.66 1.65 1.63 1.61 1.61 1.60 1.59 1.59 1.58 1.57 1.56 1.56 1.46 1.45 1.45 1.44 1.43 1.42 1.41 1.40 1.35 1.34 1.33 1.32 1.31 1.29 1.28 1.26 1.25 1.18 1.15 1.15 1.13 1.13 1.11 1.10 1.10 1.09 1.06 1.05 0.80
Me
OH
HO
H
H
H
H
31
TIPSO
OH
(1H NMR, 500 MHz, CDCl3)
1.33
1.00
2.39
1.20
2.18
2.46
3.38
1.17
5.01
2.25
1.25
0.96
4.15
21.43
3.00
9 8 7 6 5 4 3 2 1 0 -1
f1 (ppm)

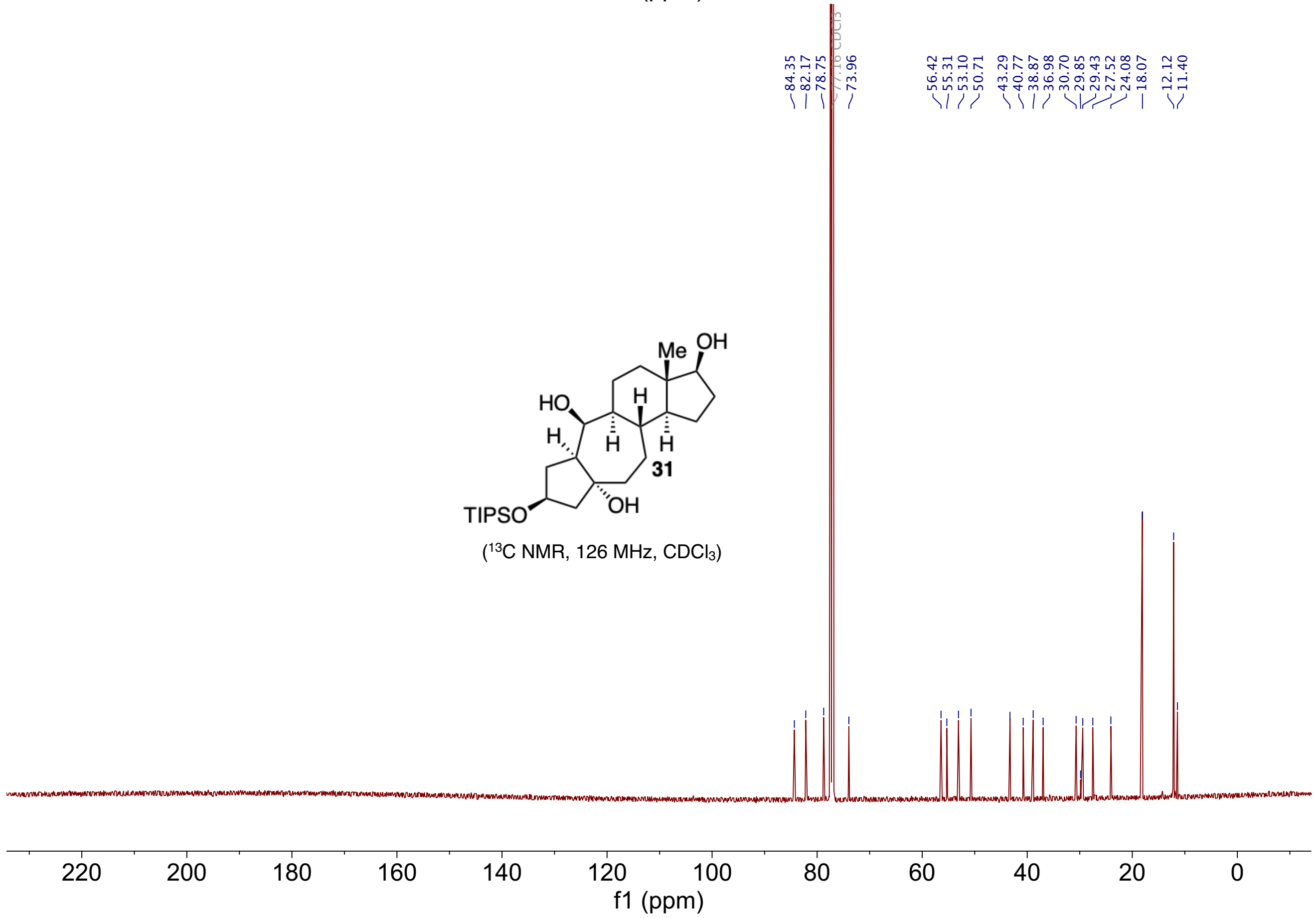
84.35
82.17
78.75
77.16 CDCl3
73.96
56.42
55.31
53.10
50.71
43.29
40.77
38.87
36.98
30.70
29.85
29.43
27.52
24.08
18.07
12.12
11.40
Me
OH
HO
H
H
H
H
31
TIPSO
OH
(13C NMR, 126 MHz, CDCl3)
220 200 180 160 140 120 100 80 60 40 20 0
f1 (ppm)

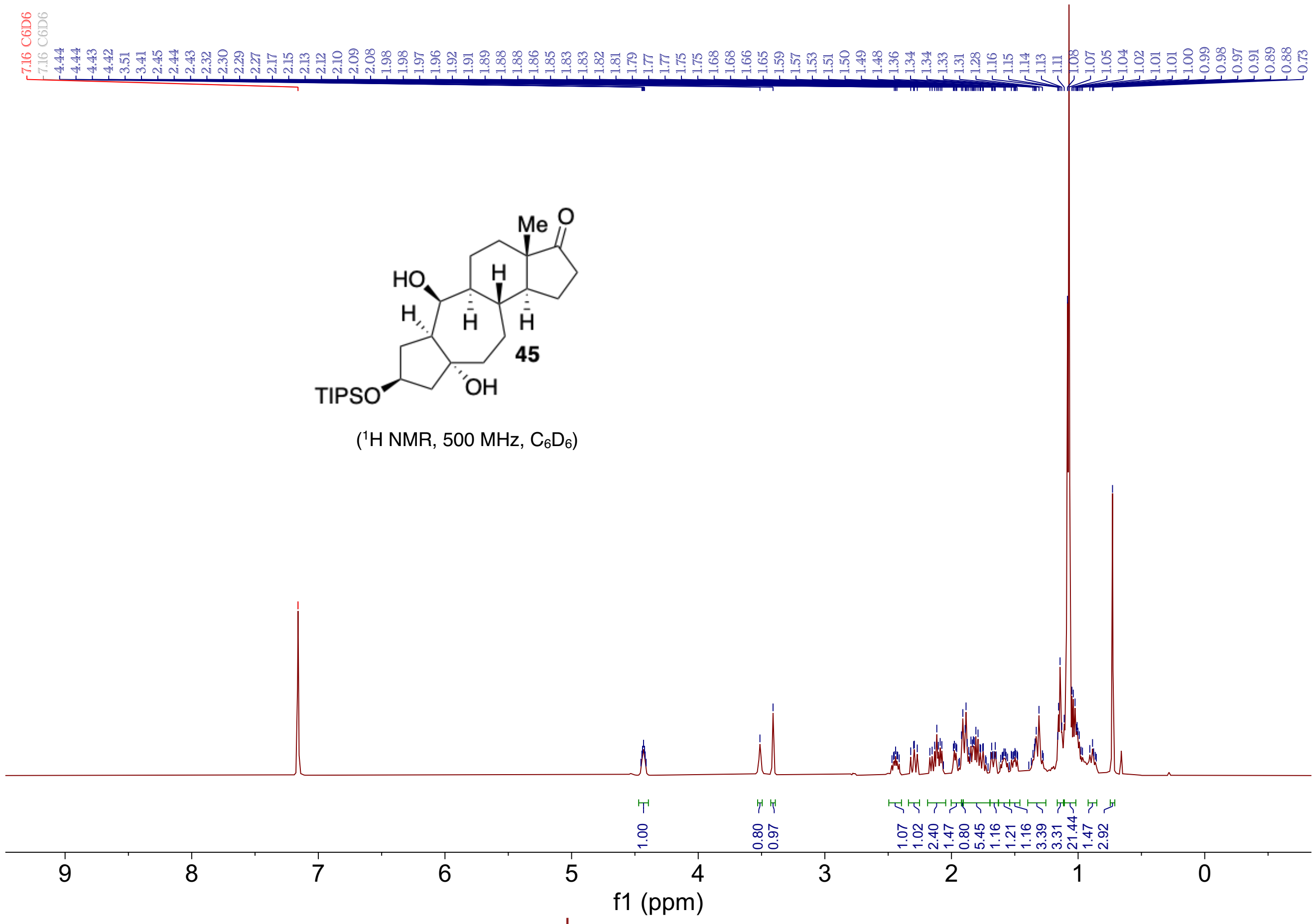
Me
O
HO
H
H
H
H
45
TIPSO
OH
(1H NMR, 500 MHz, C6D6)
1.00
0.80
0.97
1.07
1.02
2.40
1.47
0.80
5.45
1.16
1.21
1.16
3.39
3.31
21.44
1.47
2.92
f1 (ppm)

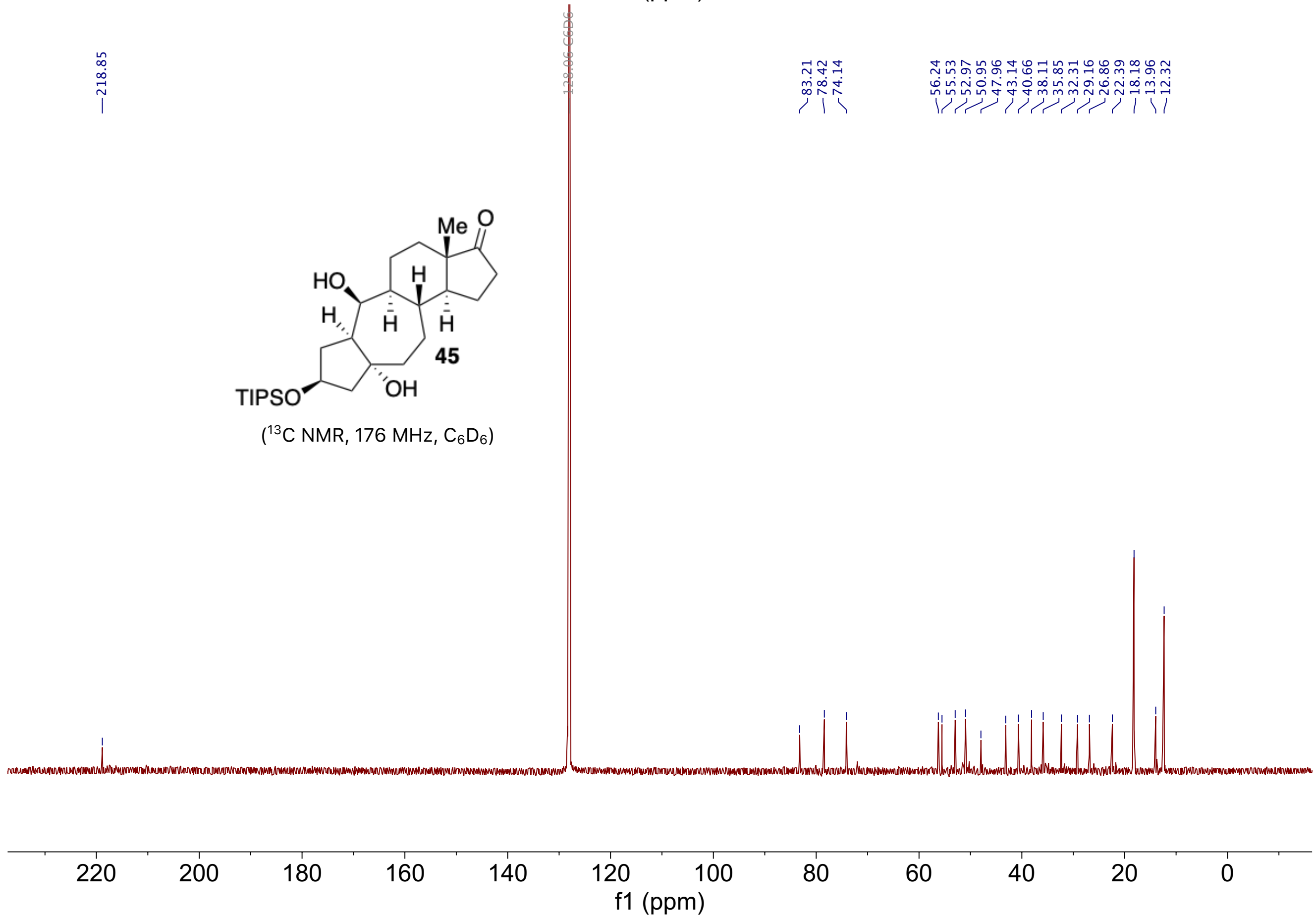
218.85
128.06 C6D6
83.21
78.42
74.14
56.24
55.53
52.97
50.95
47.96
43.14
40.66
38.11
35.85
32.31
29.16
26.86
22.39
18.18
13.96
12.32
Me
O
HO
H
H
H
H
45
TIPSO
OH
(13C NMR, 176 MHz, C6D6)
f1 (ppm)

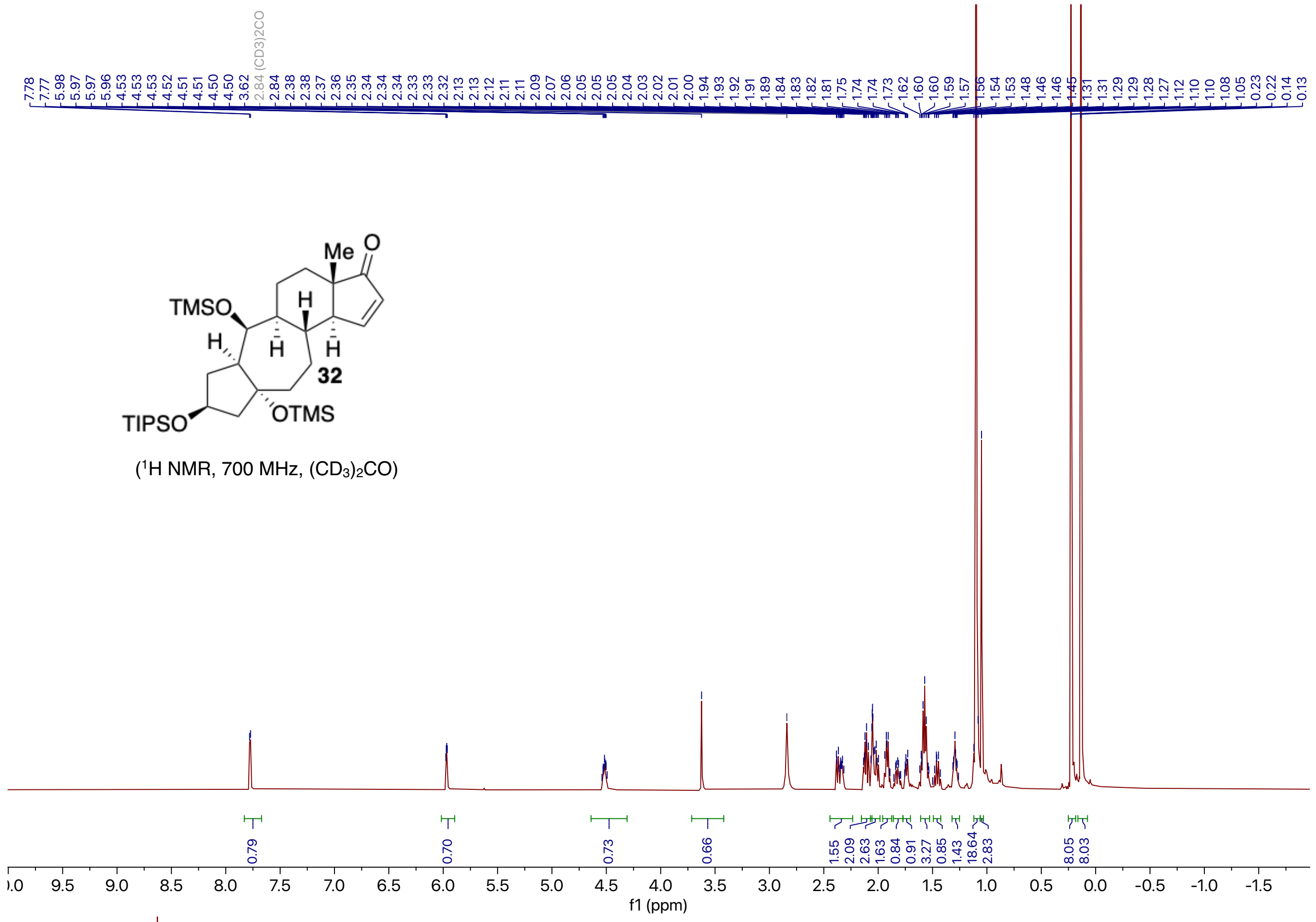

TMSO
TIPSO
OTMS
Me
32
(¹H NMR, 700 MHz, (CD₃)₂CO)
f1 (ppm)

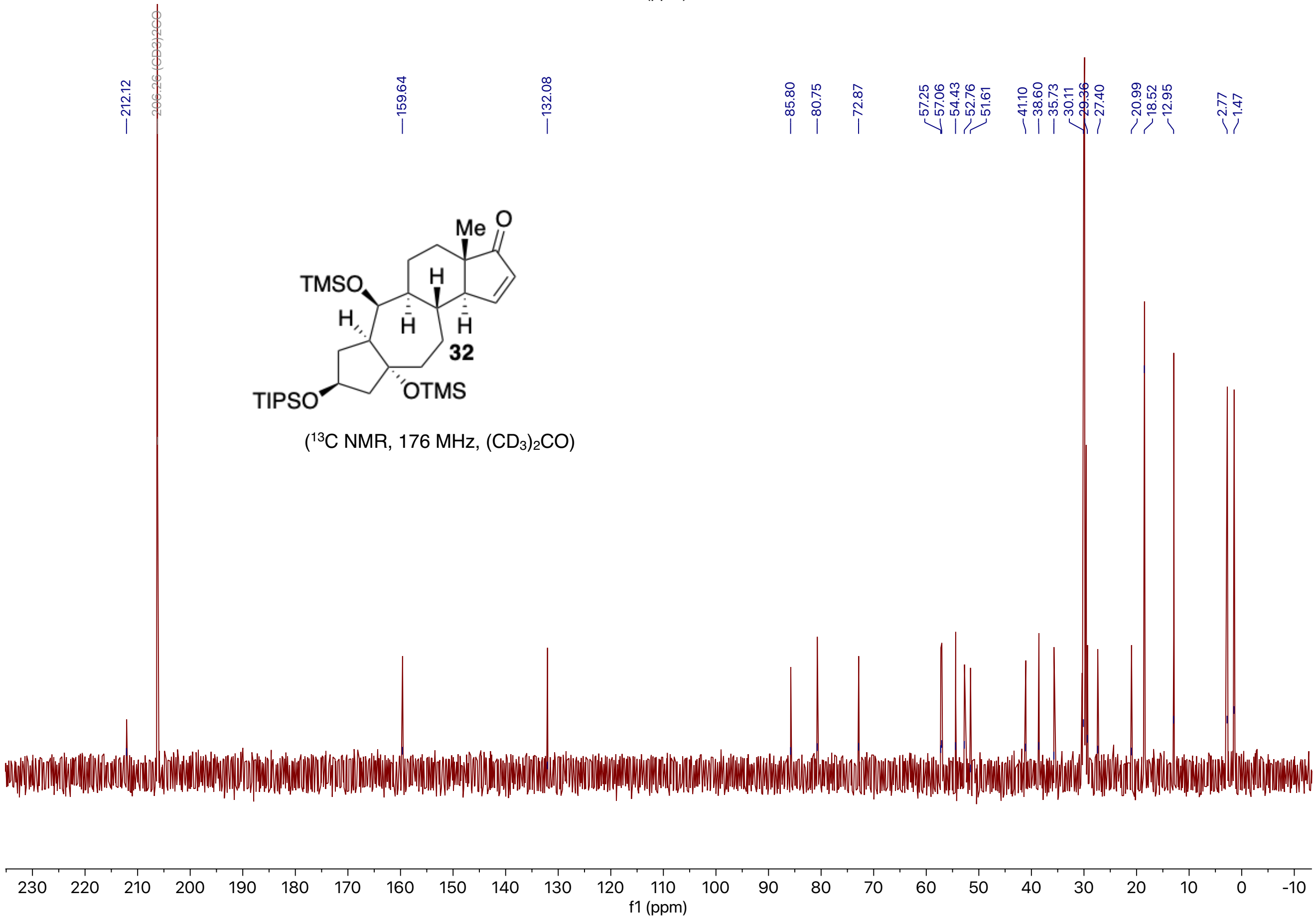

TMSO
TIPSO
OTMS
Me
32
(¹³C NMR, 176 MHz, (CD₃)₂CO)
f1 (ppm)

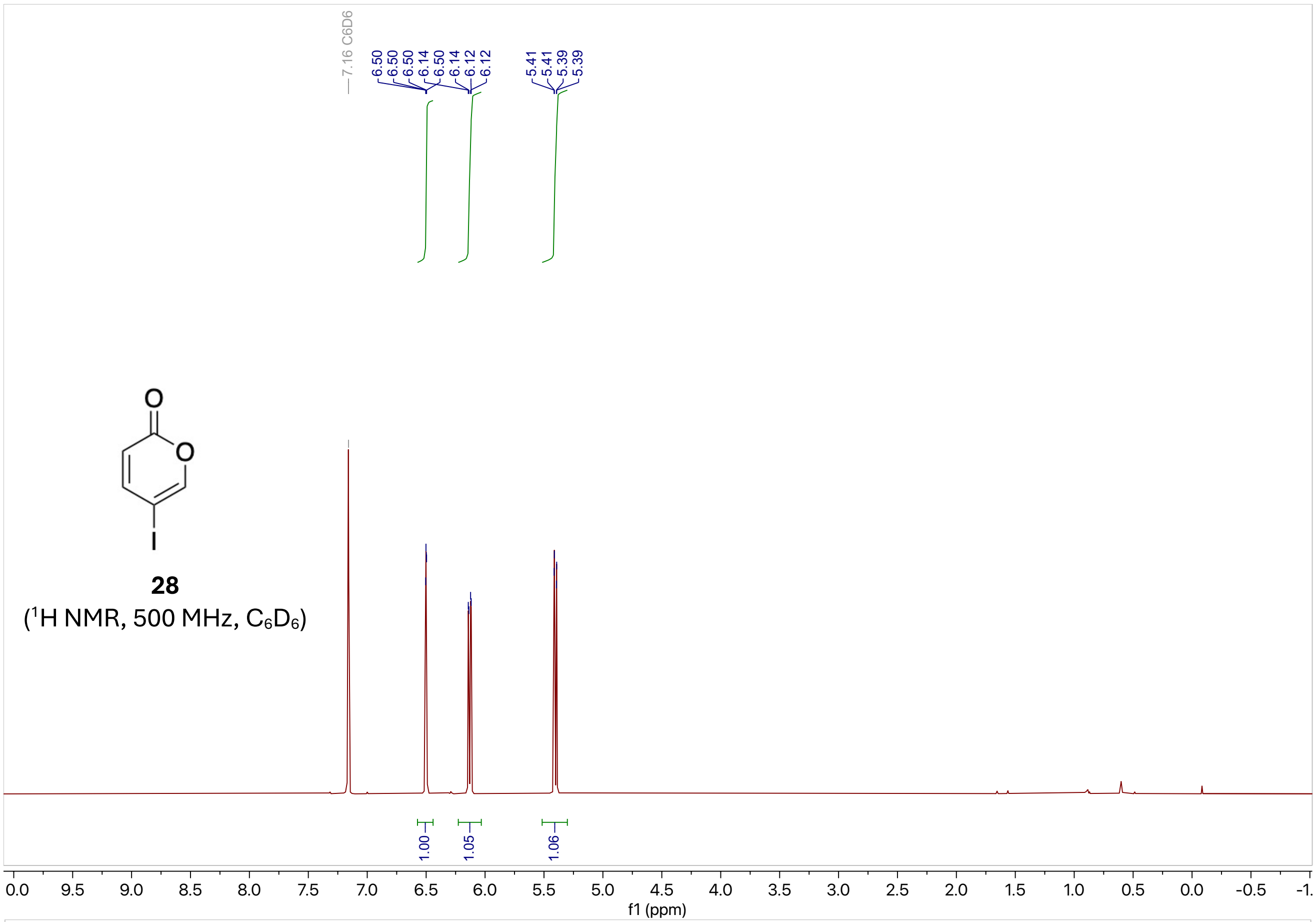

28
(^{1}H NMR, 500 MHz, C_6D_6)

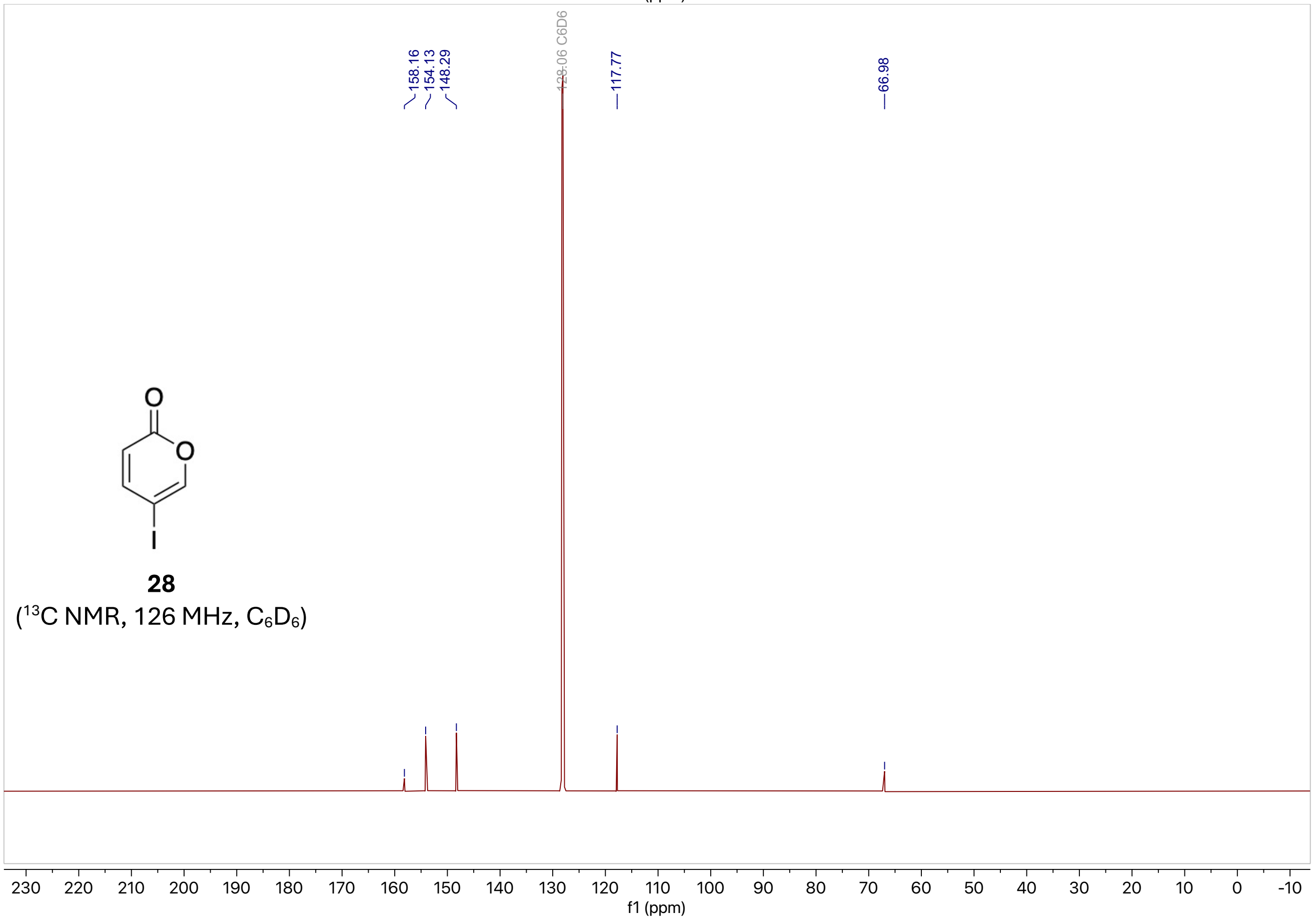

28
(^{13}C NMR, 126 MHz, C_6D_6)

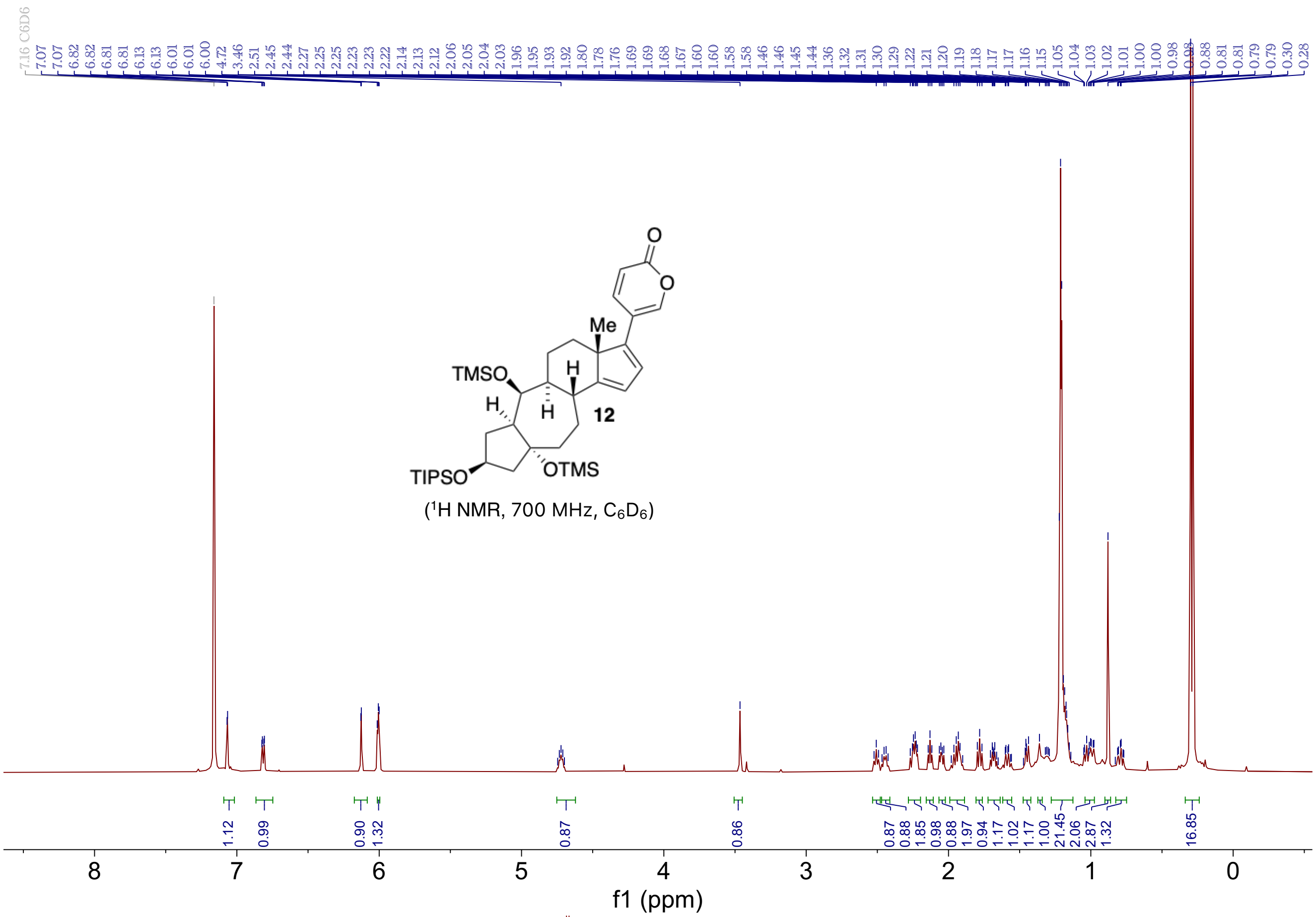
7.16 C6D6
7.07 7.07 6.82 6.82 6.81 6.81 6.13 6.13 6.01 6.01 6.00 4.72 3.46 2.51 2.45 2.44 2.27 2.25 2.25 2.23 2.23 2.22 2.14 2.13 2.12 2.06 2.05 2.04 2.03 1.96 1.95 1.93 1.92 1.80 1.78 1.76 1.69 1.69 1.68 1.67 1.60 1.60 1.58 1.58 1.46 1.46 1.45 1.44 1.36 1.32 1.31 1.30 1.29 1.22 1.21 1.20 1.19 1.18 1.17 1.17 1.17 1.16 1.15 1.05 1.04 1.03 1.02 1.01 1.00 1.00 0.98 0.88 0.81 0.81 0.79 0.79 0.30 0.28
TMSO
H
H
H
Me
O
O
12
TIPSO
OTMS
(^{1}H NMR, 700 MHz, C_6D_6)
1.12 0.99 0.90 1.32 0.87 0.86 0.87 0.88 1.85 0.98 0.88 1.97 0.94 1.17 1.02 1.17 1.00 21.45 2.06 2.87 1.32 16.85
8 7 6 5 4 3 2 1 0
f1 (ppm)

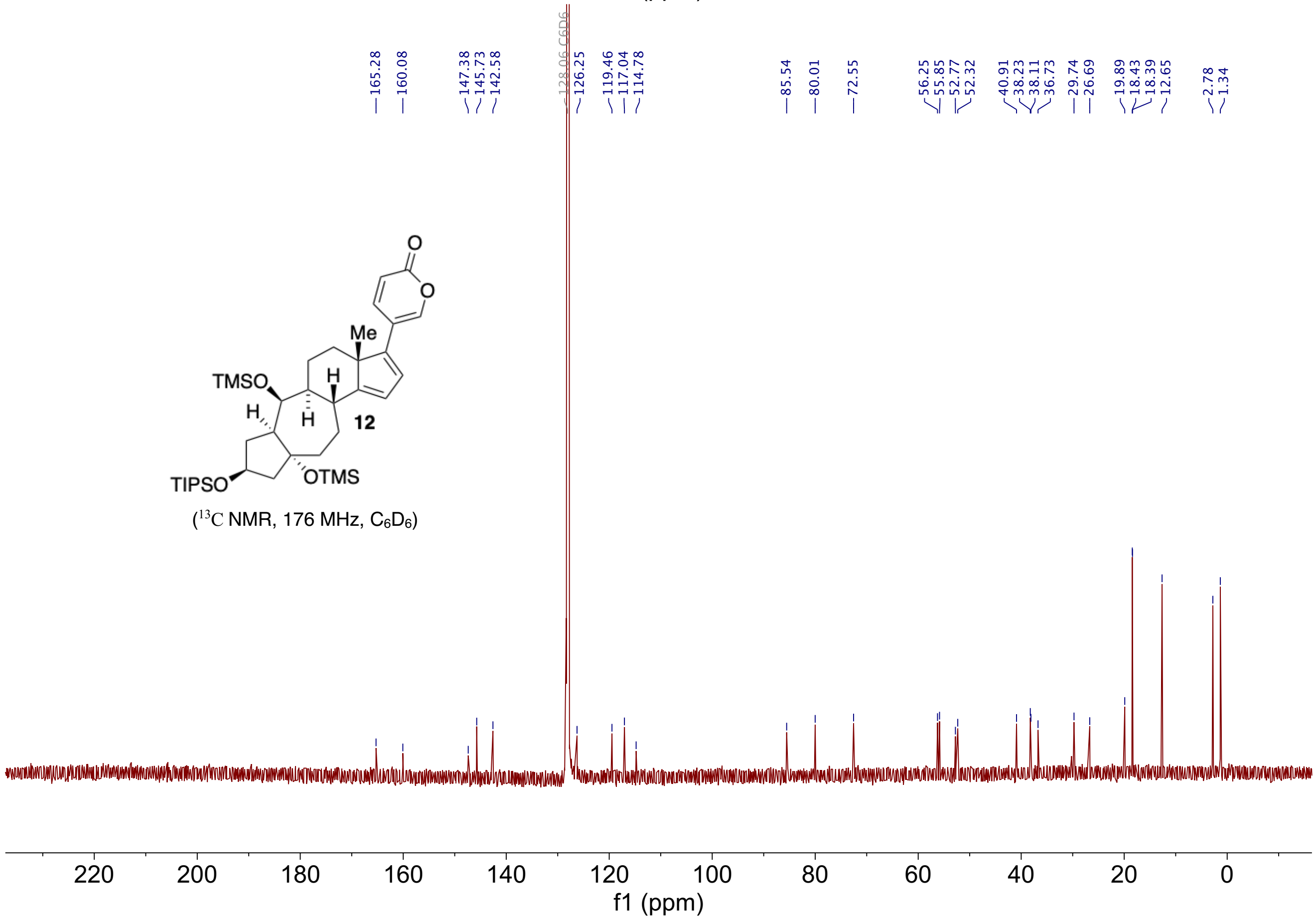
165.28 160.08 147.38 145.73 142.58 128.06 C6D6 126.25 119.46 117.04 114.78 85.54 80.01 72.55 56.25 55.85 52.77 52.32 40.91 38.23 38.11 36.73 29.74 26.69 19.89 18.43 18.39 12.65 2.78 1.34
TMSO
H
H
H
Me
O
O
12
TIPSO
OTMS
(^{13}C NMR, 176 MHz, C_6D_6)
220 200 180 160 140 120 100 80 60 40 20 0
f1 (ppm)

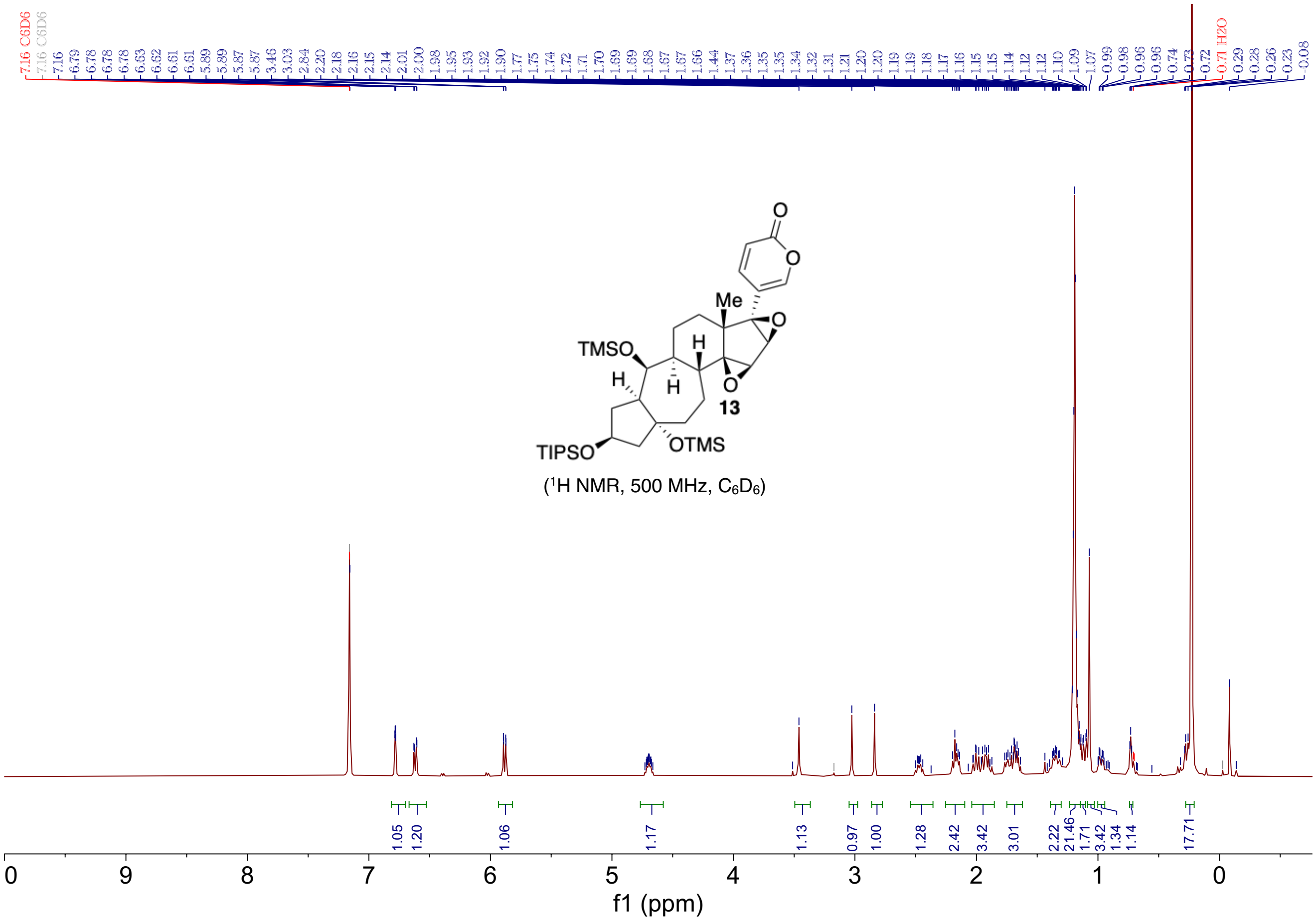
7.16 C6D6
7.16 C6D6
(¹H NMR, 500 MHz, C6D6)
TMSO
TIPSO
OTMS
Me
13
f1 (ppm)

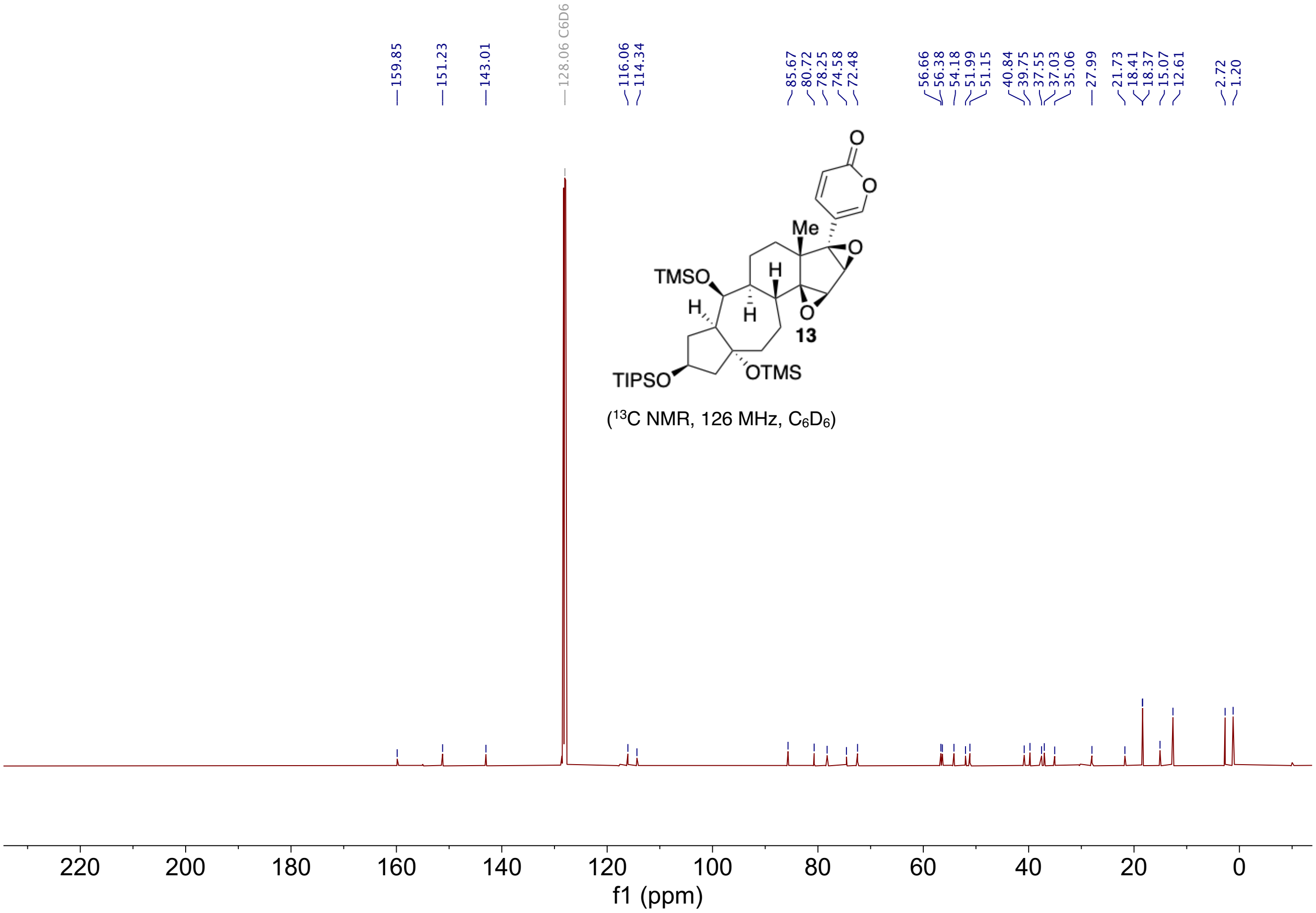
128.06 C6D6
159.85
151.23
143.01
116.06
114.34
(¹³C NMR, 126 MHz, C6D6)
TMSO
TIPSO
OTMS
Me
13
f1 (ppm)

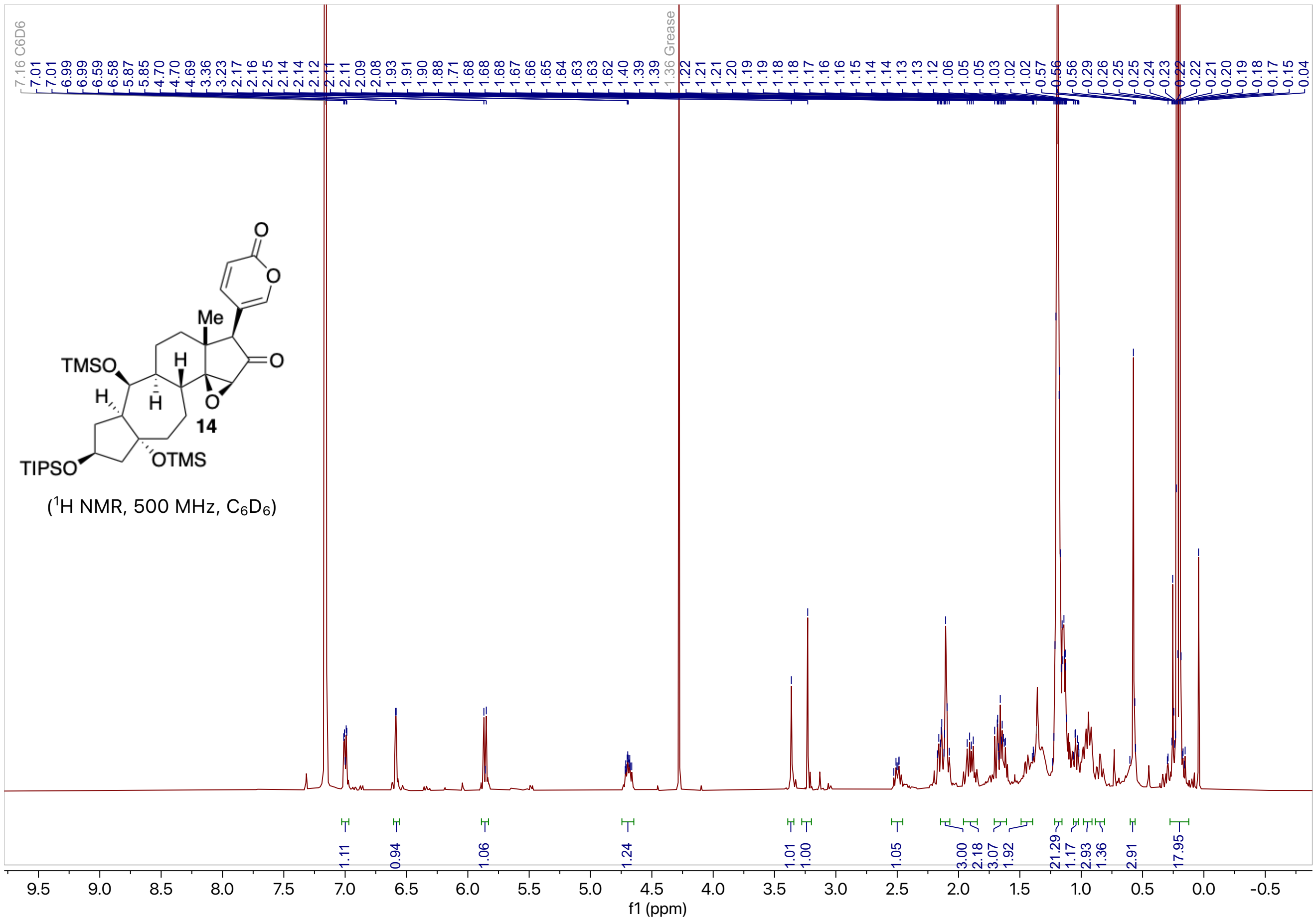

(¹H NMR, 500 MHz, C_6D_6)

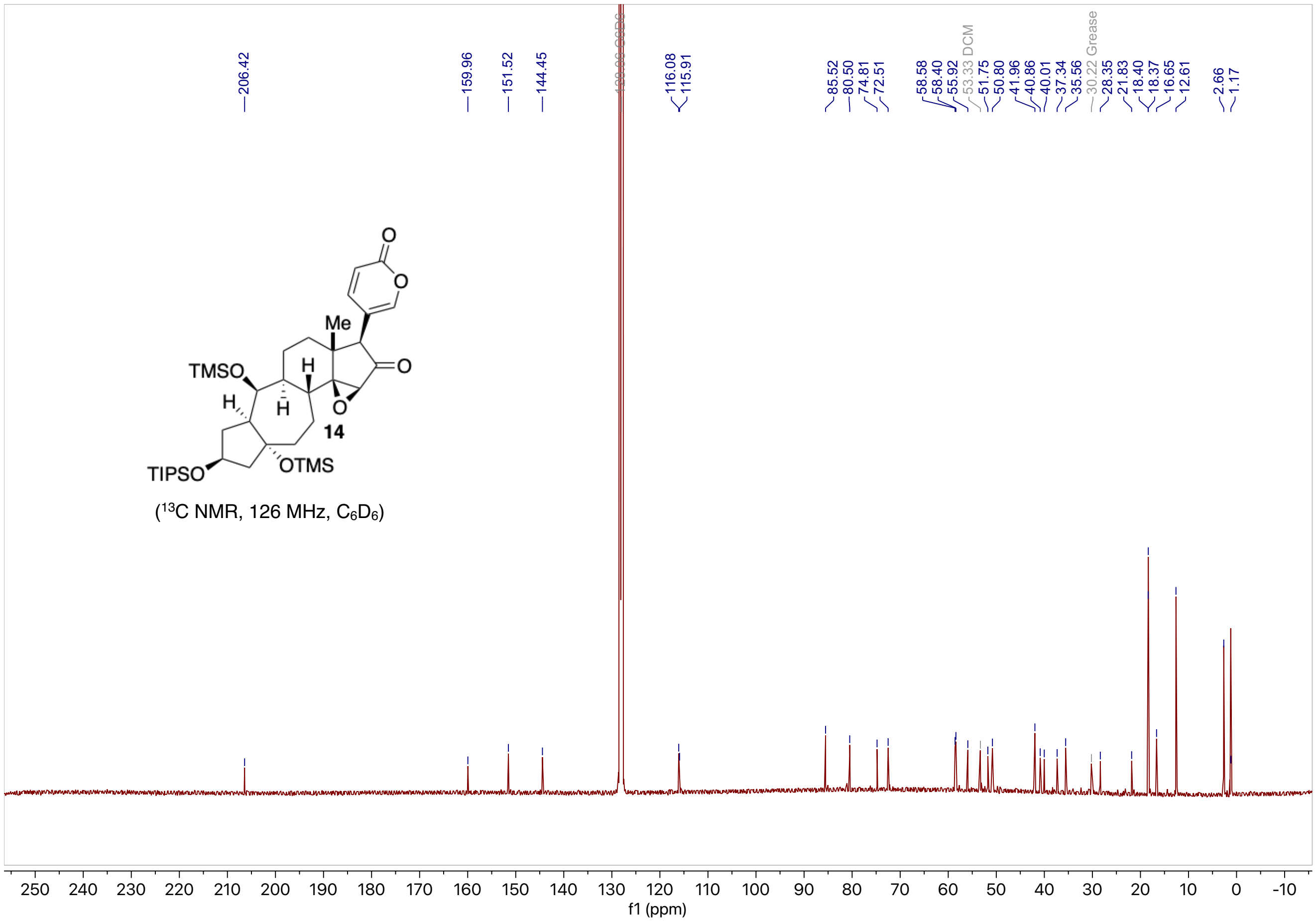

(¹³C NMR, 126 MHz, C_6D_6)

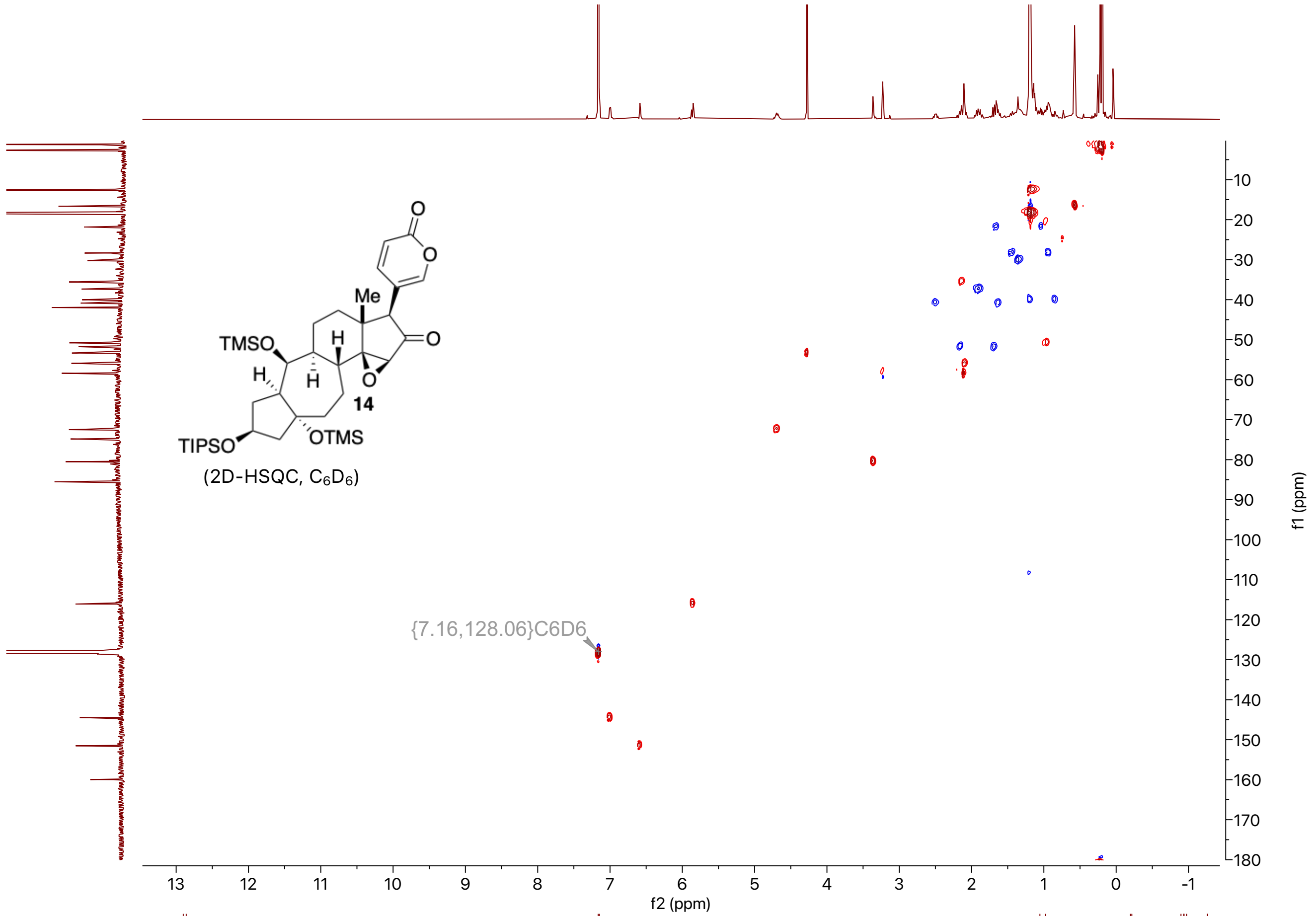
TMSO
H
H
Me
O
O
O
14
TIPSO
OTMS
(2D-HSQC, C6D6)
{7.16,128.06}C6D6
f2 (ppm)
f1 (ppm)

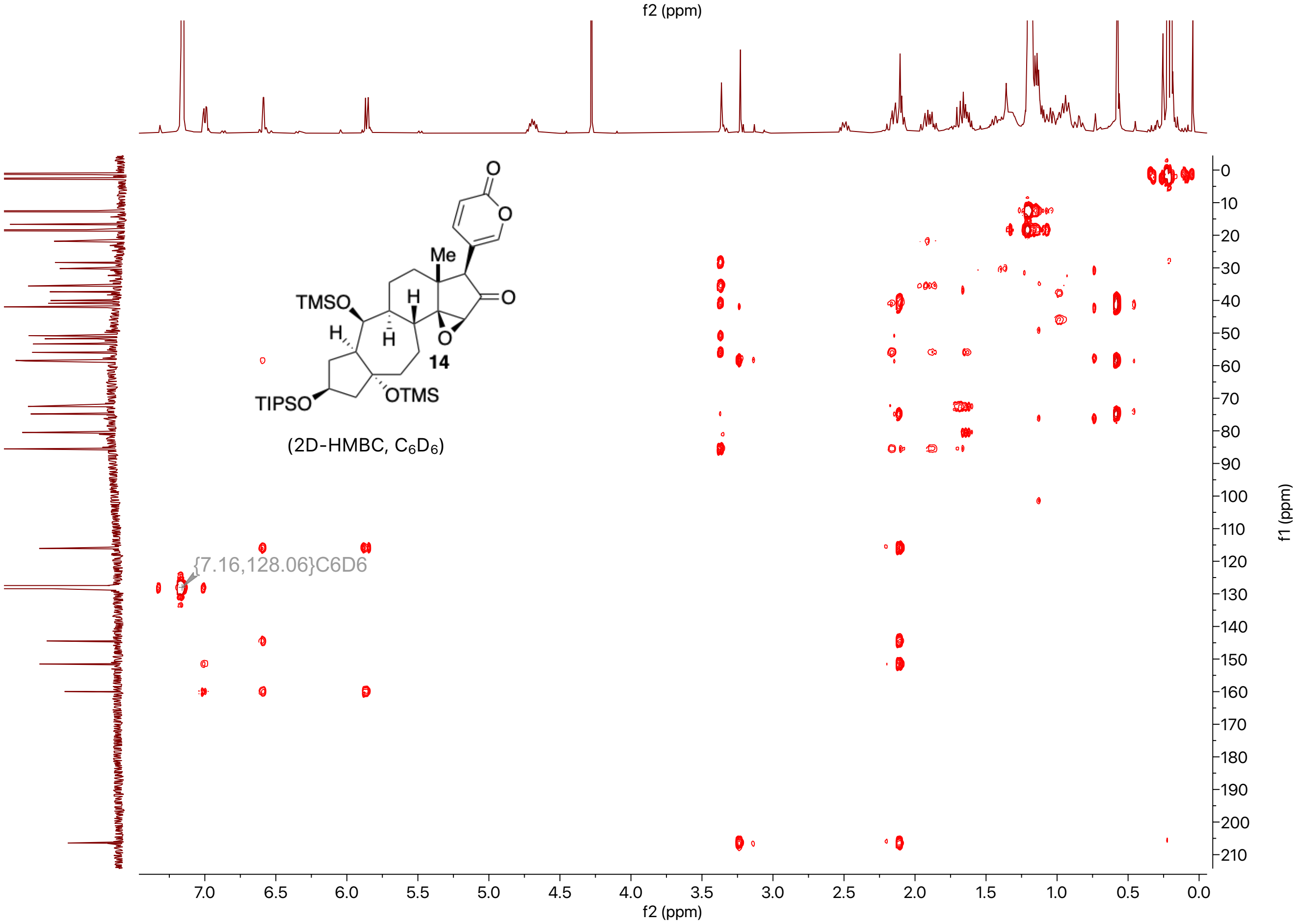
TMSO
H
H
Me
O
O
O
14
TIPSO
OTMS
(2D-HMBC, C6D6)
{7.16,128.06}C6D6
f2 (ppm)
f1 (ppm)

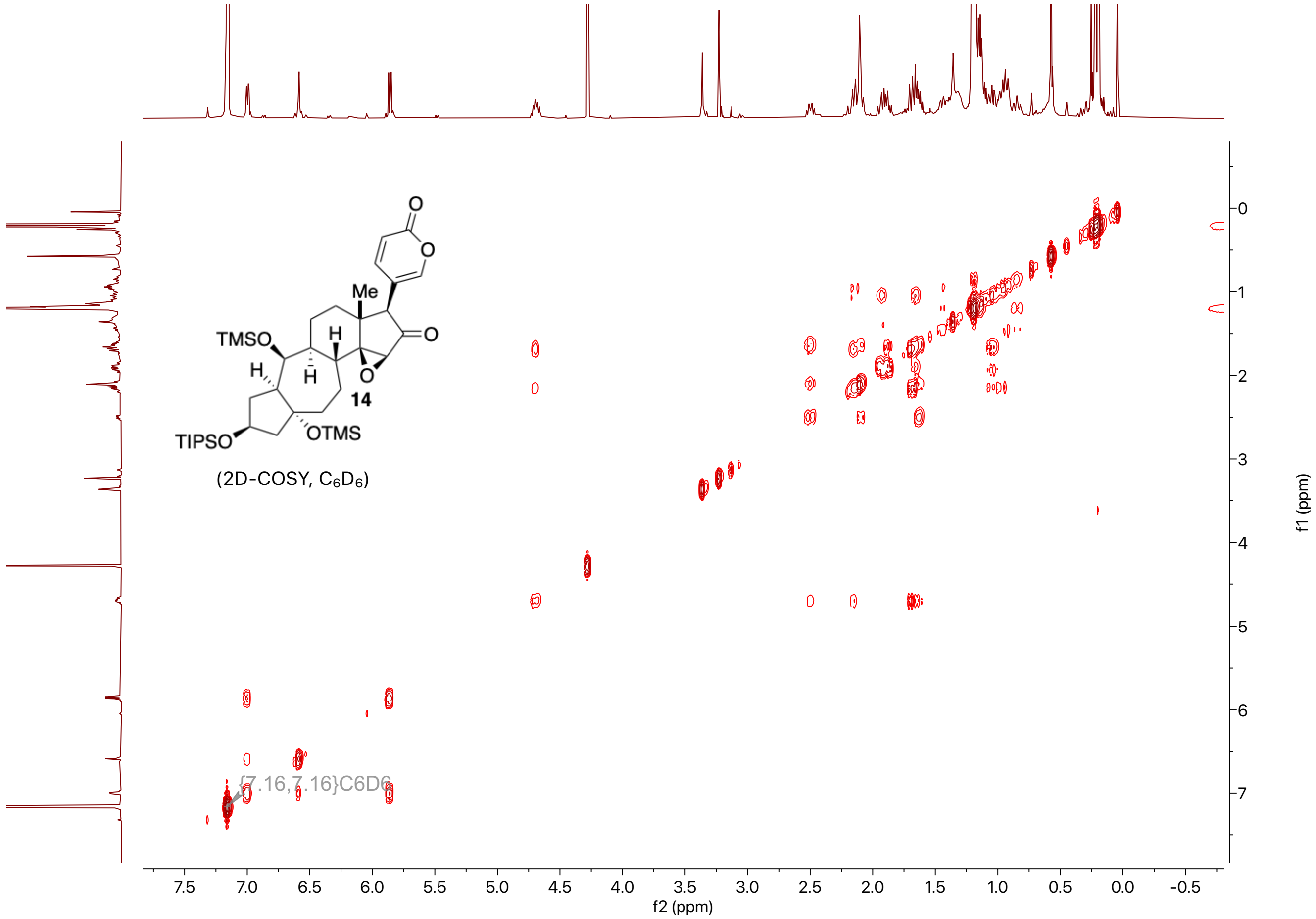

TMSO
H
H
H
Me
O
O
O
14
TIPSO
OTMS
(2D-COSY, C6D6)
{7.16,7.16}C6D6
f2 (ppm)
f1 (ppm)

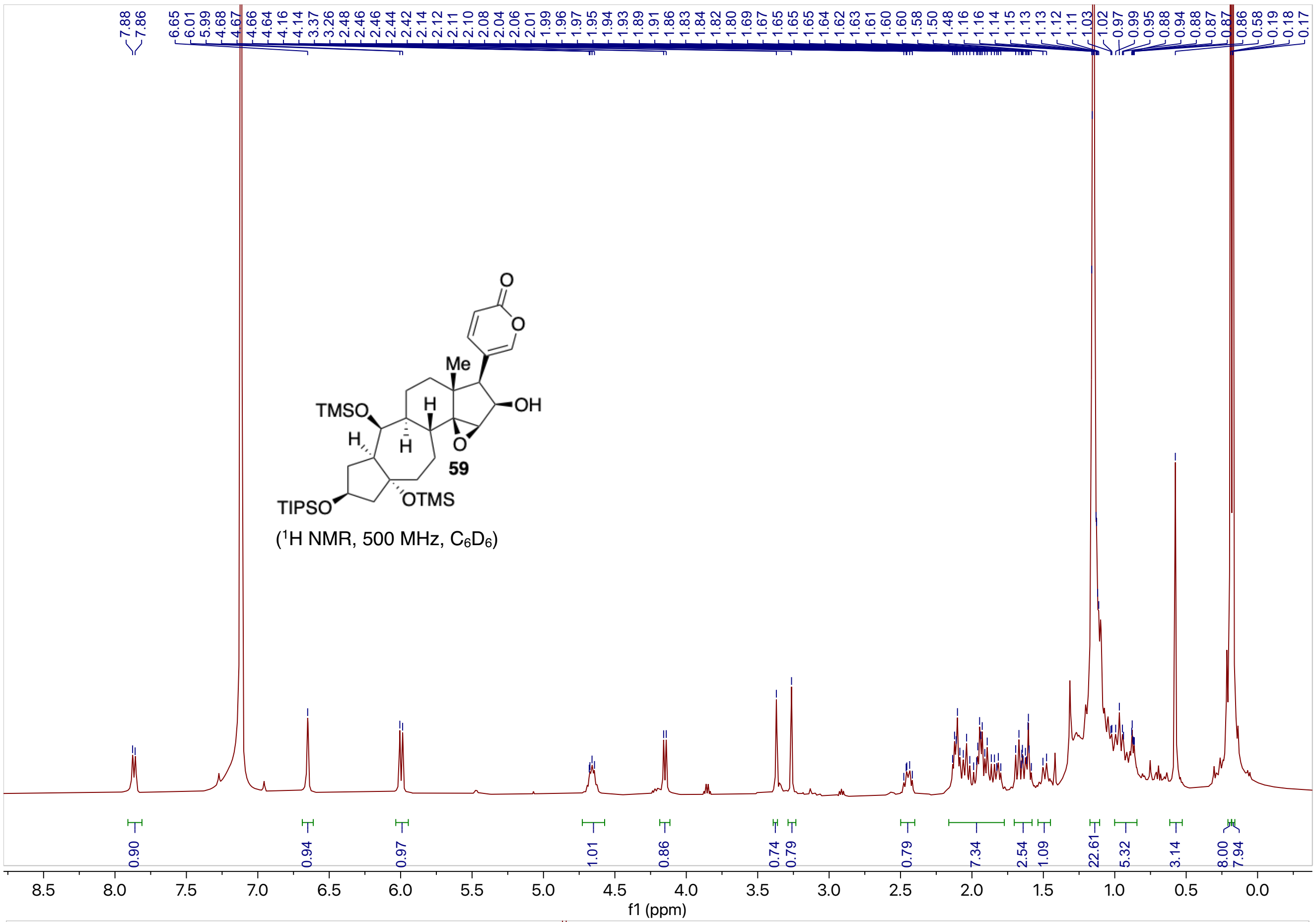
7.88
7.86
6.65
6.01
5.99
4.68
4.67
4.66
4.64
4.16
4.14
3.37
3.26
2.48
2.46
2.46
2.44
2.42
2.14
2.12
2.11
2.10
2.08
2.06
2.04
2.01
1.99
1.96
1.97
1.95
1.94
1.93
1.89
1.91
1.86
1.83
1.84
1.82
1.80
1.69
1.67
1.65
1.65
1.64
1.62
1.63
1.61
1.60
1.60
1.58
1.50
1.48
1.16
1.16
1.14
1.15
1.13
1.13
1.12
1.11
1.03
1.02
0.97
0.99
0.95
0.88
0.94
0.88
0.87
0.87
0.86
0.58
0.19
0.18
0.17
O
O
Me
TMSO
H
OH
H
H
O
59
TIPSO
OTMS
(^{1}H NMR, 500 MHz, C_6D_6)
0.90
0.94
0.97
1.01
0.86
0.74
0.79
0.79
7.34
2.54
1.09
22.61
5.32
3.14
8.00
7.94
8.5
8.0
7.5
7.0
6.5
6.0
5.5
5.0
4.5
4.0
3.5
3.0
2.5
2.0
1.5
1.0
0.5
0.0
f1 (ppm)

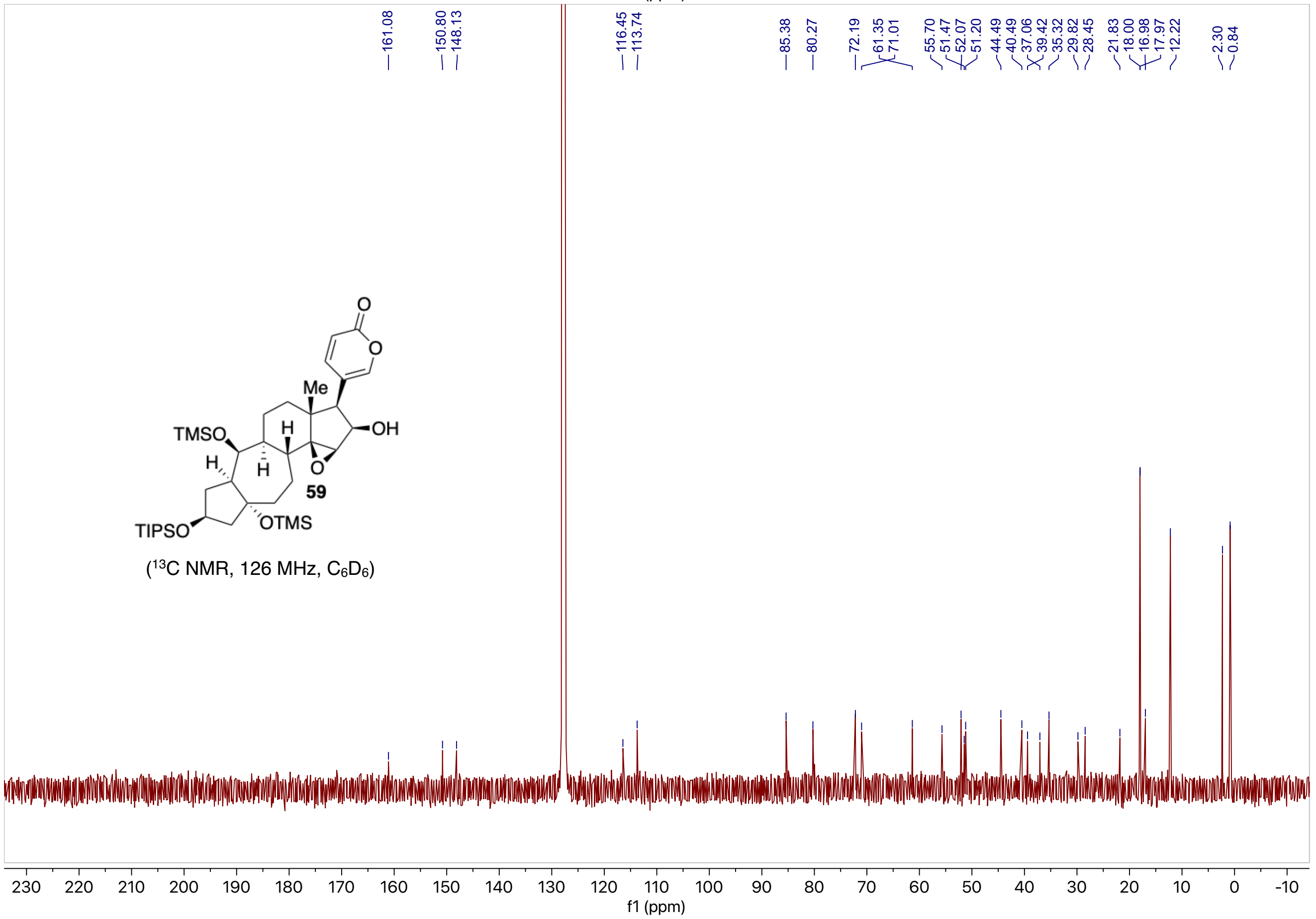
161.08
150.80
148.13
116.45
113.74
85.38
80.27
72.19
61.35
71.01
55.70
51.47
52.07
51.20
44.49
40.49
37.06
39.42
35.32
29.82
28.45
21.83
18.00
16.98
17.97
12.22
2.30
0.84
O
O
Me
TMSO
H
OH
H
H
O
59
TIPSO
OTMS
(^{13}C NMR, 126 MHz, C_6D_6)
230
220
210
200
190
180
170
160
150
140
130
120
110
100
90
80
70
60
50
40
30
20
10
0
-10
f1 (ppm)

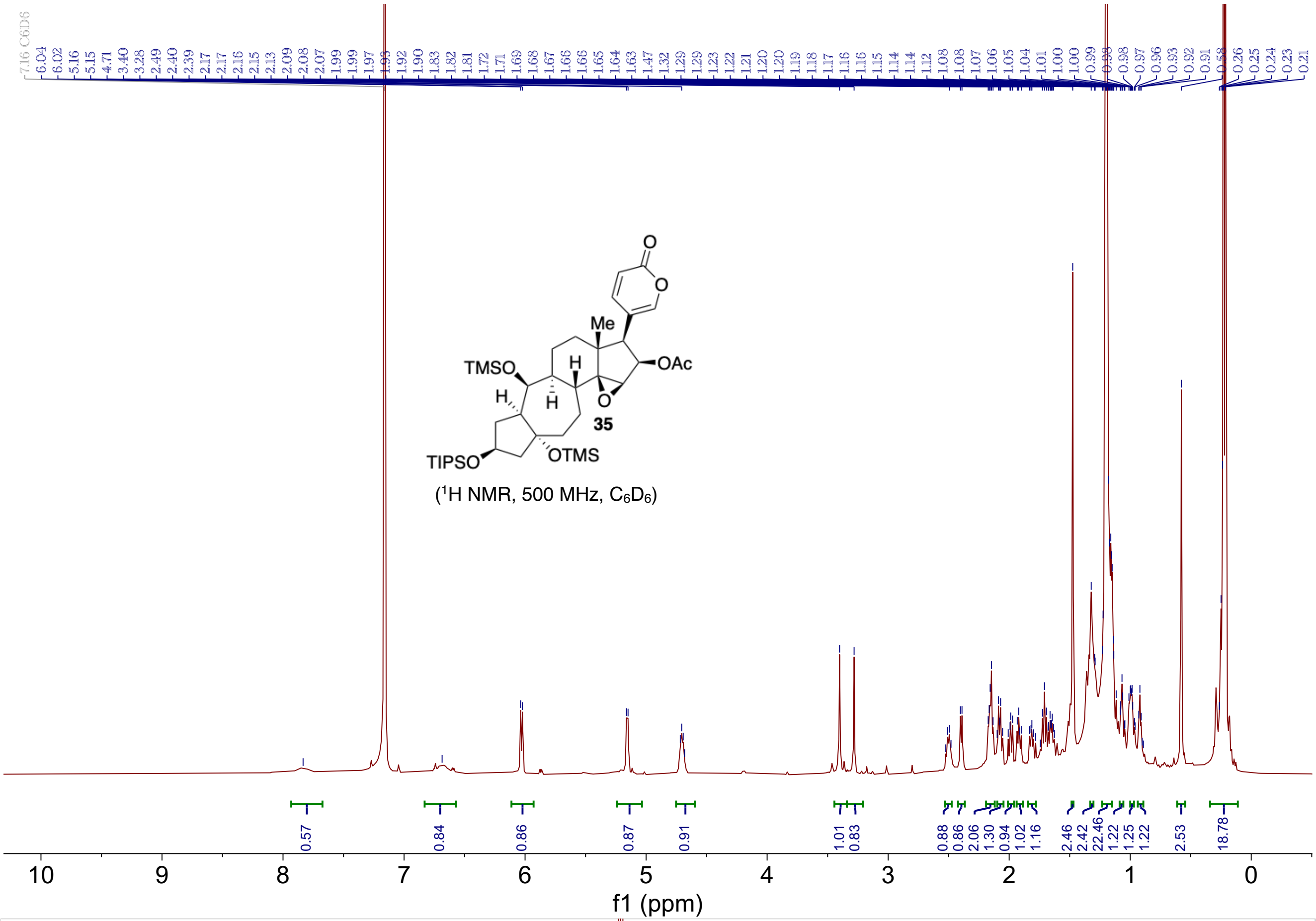
7.16 C6D6
6.04
6.02
5.16
5.15
4.71
3.40
3.28
2.49
2.40
2.39
2.17
2.17
2.16
2.15
2.13
2.09
2.08
2.07
1.99
1.99
1.97
1.93
1.92
1.90
1.83
1.82
1.81
1.72
1.71
1.69
1.68
1.67
1.66
1.66
1.65
1.64
1.63
1.47
1.32
1.29
1.29
1.23
1.22
1.21
1.20
1.20
1.19
1.18
1.17
1.16
1.16
1.15
1.14
1.14
1.12
1.08
1.08
1.07
1.06
1.05
1.04
1.01
1.00
1.00
0.99
0.98
0.98
0.97
0.96
0.93
0.92
0.91
0.58
0.26
0.25
0.24
0.23
0.21
O
Me
TMSO
H
H
H
OAc
O
35
TIPSO
OTMS
(^{1}H NMR, 500 MHz, C_6D_6)
0.57
0.84
0.86
0.87
0.91
1.01
0.83
0.88
0.86
2.06
1.30
0.94
1.02
1.16
2.46
2.42
22.46
1.22
1.25
1.22
2.53
18.78
10 9 8 7 6 5 4 3 2 1 0
f1 (ppm)

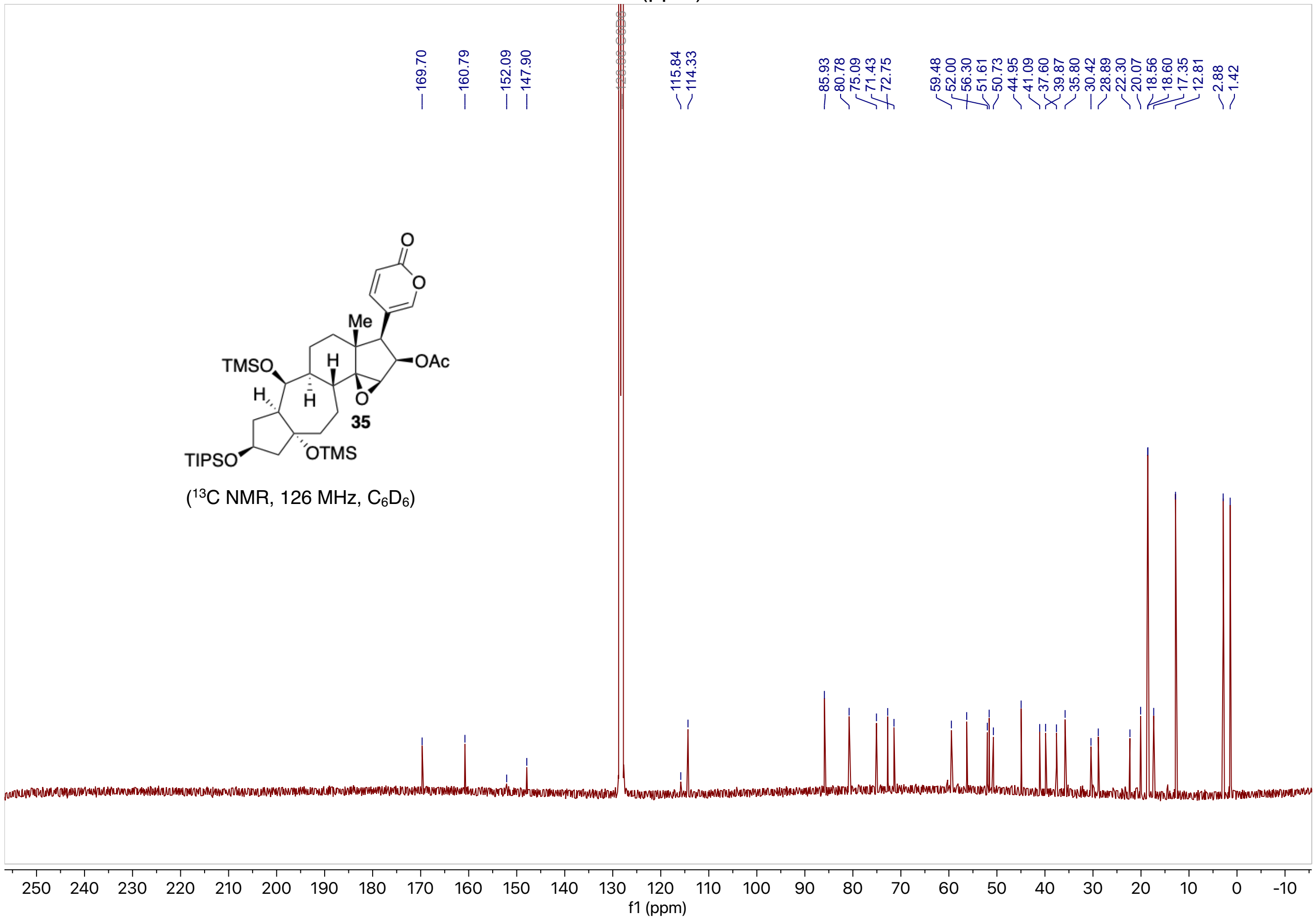
169.70
160.79
152.09
147.90
115.84
114.33
85.93
80.78
75.09
71.43
72.75
59.48
52.00
56.30
51.61
50.73
44.95
41.09
37.60
39.87
35.80
30.42
28.89
22.30
20.07
18.56
18.60
17.35
12.81
2.88
1.42
O
Me
TMSO
H
H
H
OAc
O
35
TIPSO
OTMS
(^{13}C NMR, 126 MHz, C_6D_6)
250 240 230 220 210 200 190 180 170 160 150 140 130 120 110 100 90 80 70 60 50 40 30 20 10 0 -10
f1 (ppm)

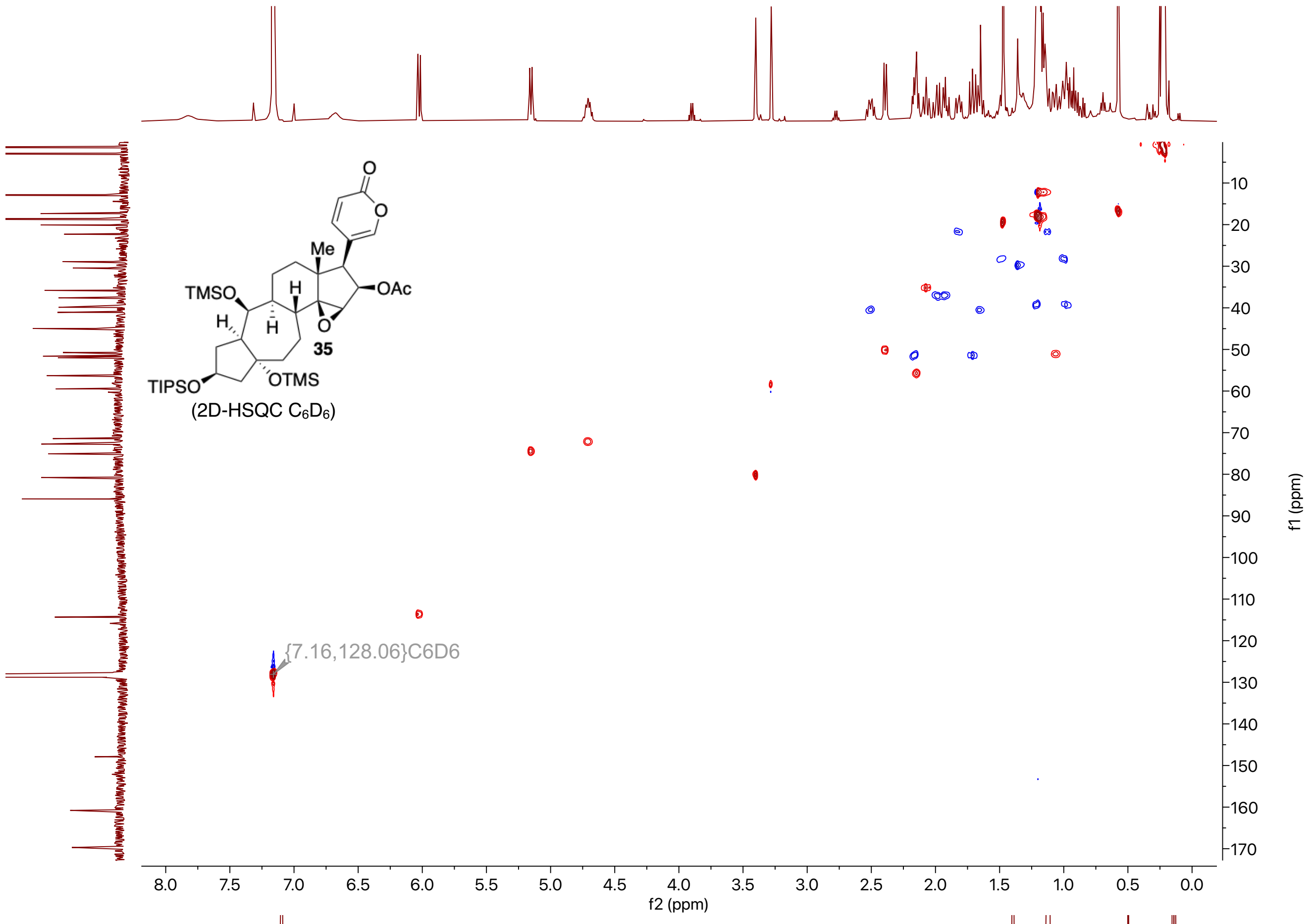

O
O
Me
TMSO
H
OAc
H
H
O
35
TIPSO
OTMS
(2D-HSQC C6D6)
{7.16,128.06}C6D6
f1 (ppm)
f2 (ppm)

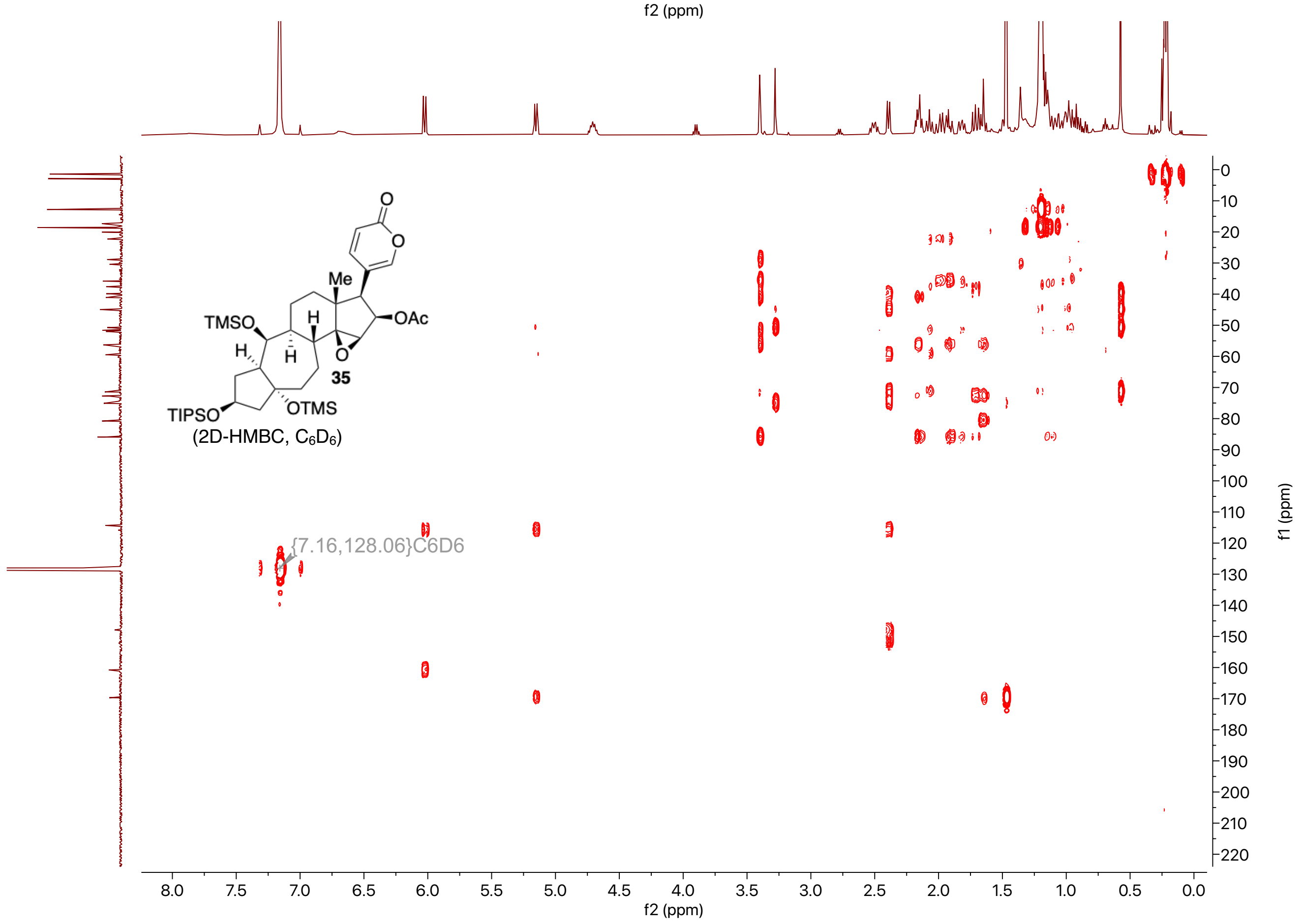

O
O
Me
TMSO
H
OAc
H
H
O
35
TIPSO
OTMS
(2D-HMBC, C6D6)
{7.16,128.06}C6D6
f1 (ppm)
f2 (ppm)

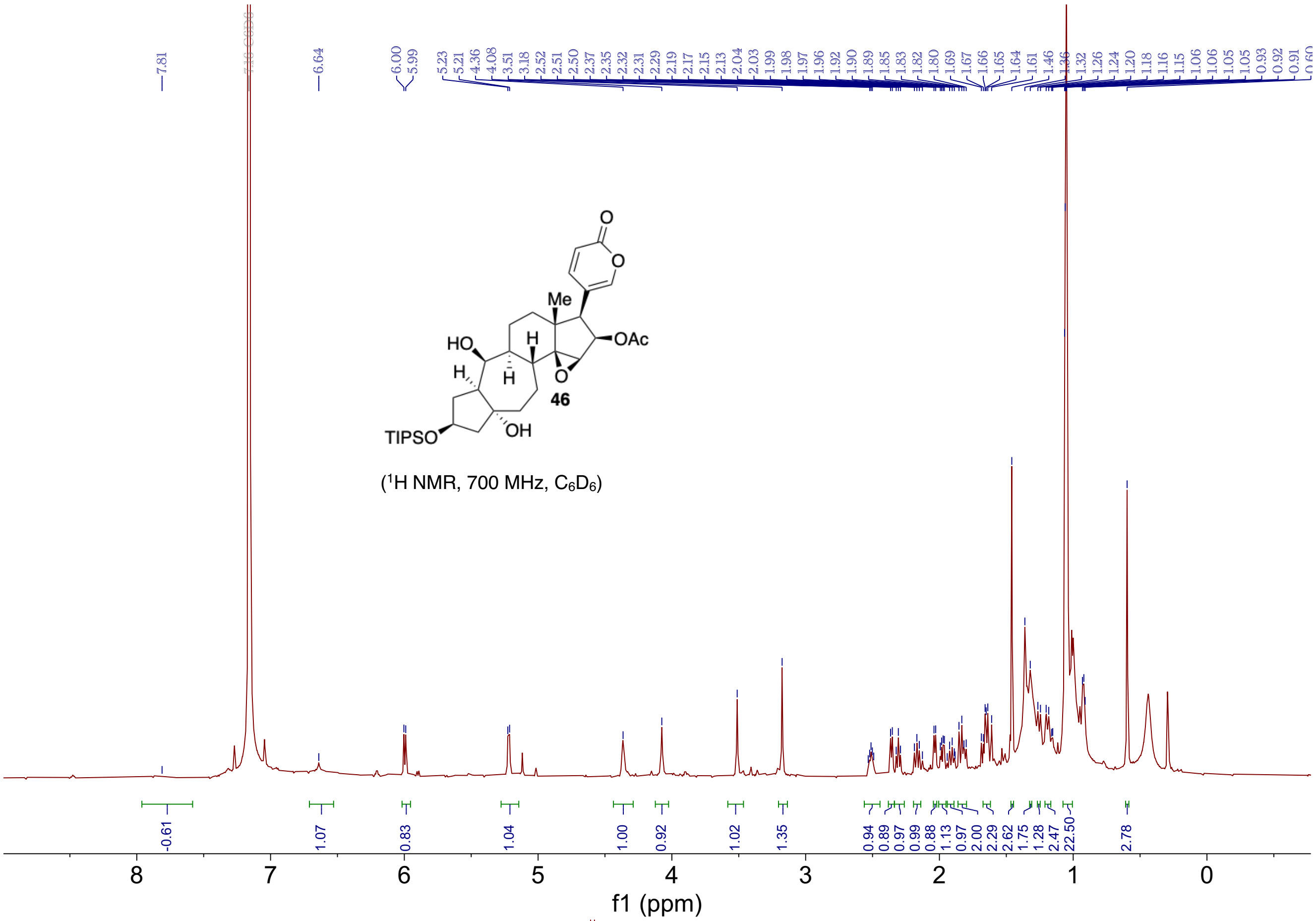

(^{1}H NMR, 700 MHz, C_6D_6)

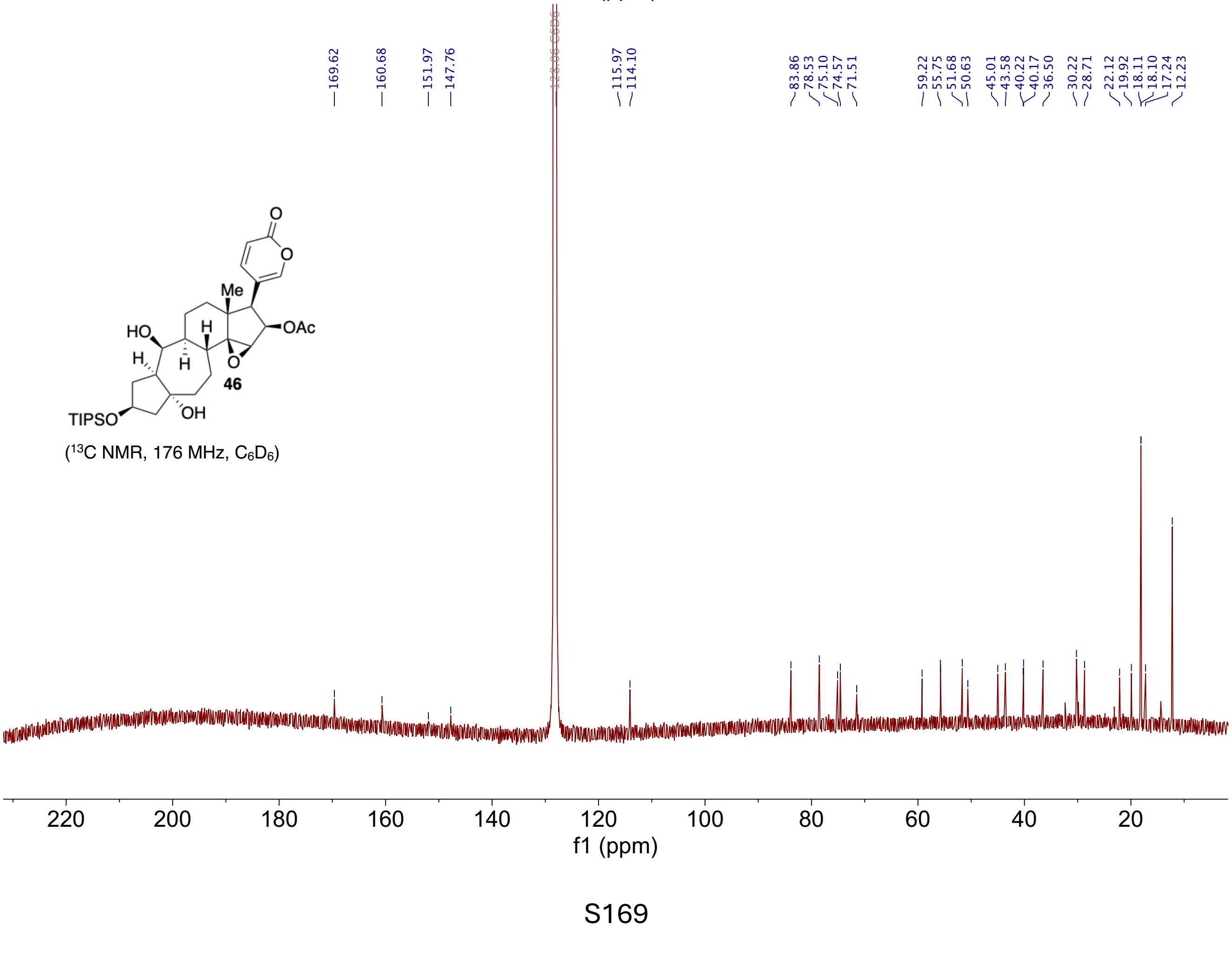

(^{13}C NMR, 176 MHz, C_6D_6)

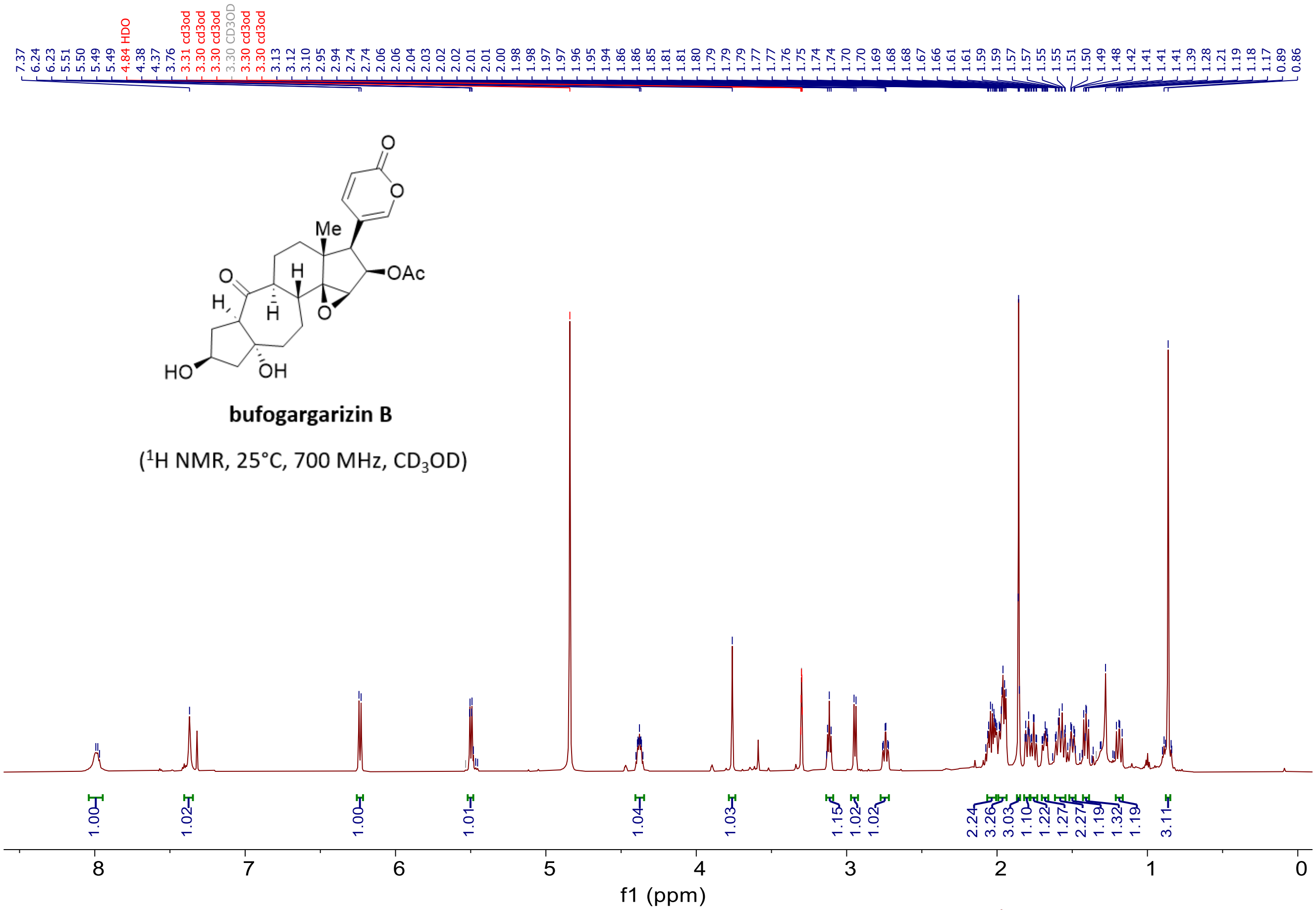
7.37 6.24 6.23 5.51 5.50 5.49 5.49 4.84 HDO 4.38 4.37 3.76 3.31 cd3od 3.30 cd3od 3.30 cd3od 3.30 CD3OD 3.30 cd3od 3.30 cd3od 3.13 3.12 3.10 2.95 2.94 2.74 2.74 2.06 2.06 2.04 2.03 2.02 2.02 2.01 2.01 2.00 1.98 1.98 1.97 1.97 1.96 1.95 1.94 1.86 1.85 1.81 1.81 1.80 1.79 1.79 1.79 1.77 1.76 1.75 1.74 1.74 1.70 1.70 1.69 1.68 1.68 1.67 1.66 1.61 1.61 1.59 1.59 1.59 1.57 1.57 1.55 1.55 1.51 1.50 1.49 1.48 1.42 1.41 1.41 1.41 1.39 1.28 1.21 1.19 1.18 1.17 0.89 0.86
Me
OAc
HO
OH
bufogargarizin B
(1H NMR, 25°C, 700 MHz, CD3OD)
1.00 1.02 1.00 1.01 1.04 1.03 1.15 1.02 1.02 2.24 3.26 3.03 1.10 1.22 1.27 2.27 1.19 1.32 1.19 3.11
f1 (ppm)

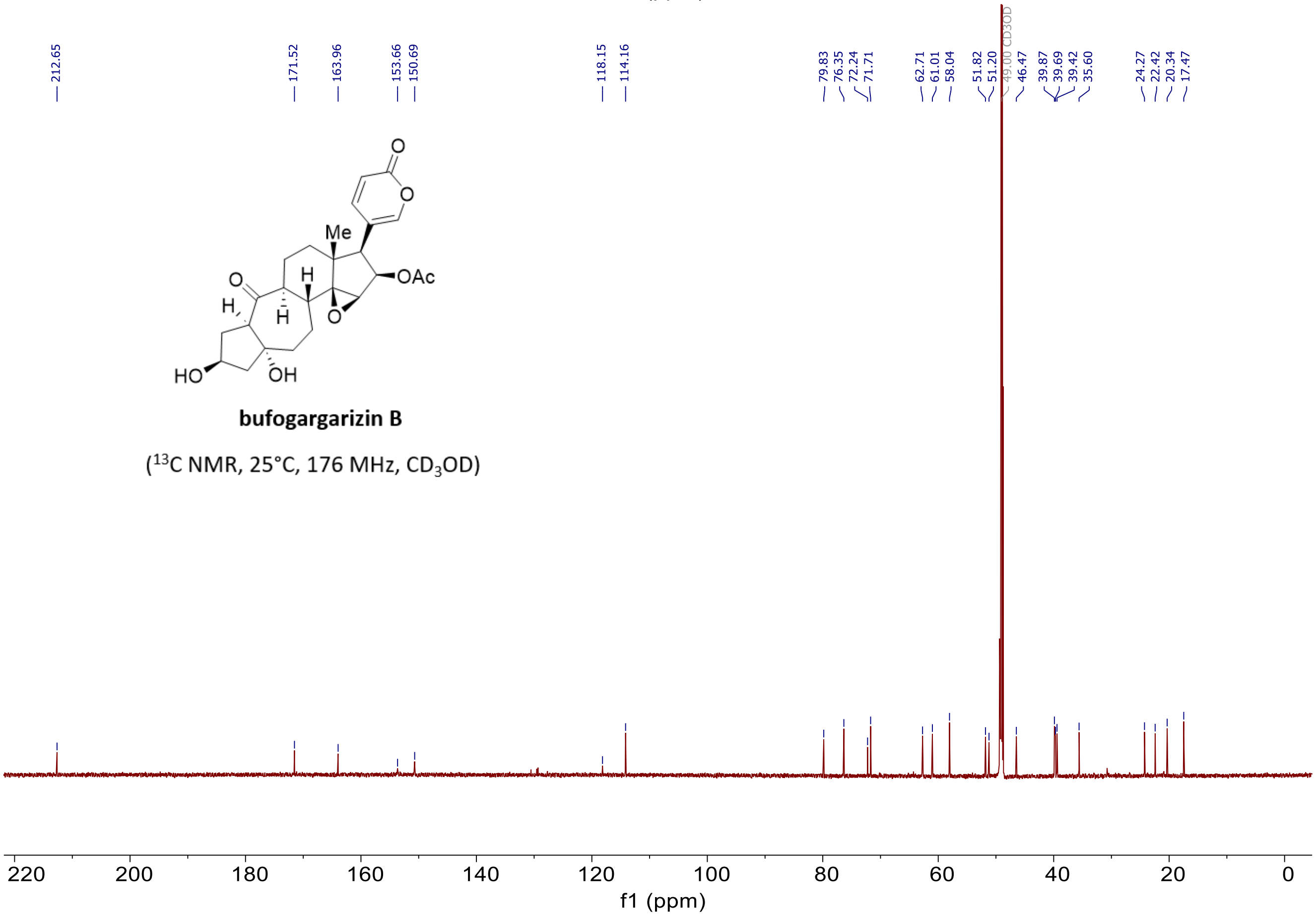
212.65 171.52 163.96 153.66 150.69 118.15 114.16 79.83 76.35 72.24 71.71 62.71 61.01 58.04 51.82 51.20 49.00 CD3OD 46.47 39.87 39.69 39.42 35.60 24.27 22.42 20.34 17.47
Me
OAc
HO
OH
bufogargarizin B
(13C NMR, 25°C, 176 MHz, CD3OD)
f1 (ppm)

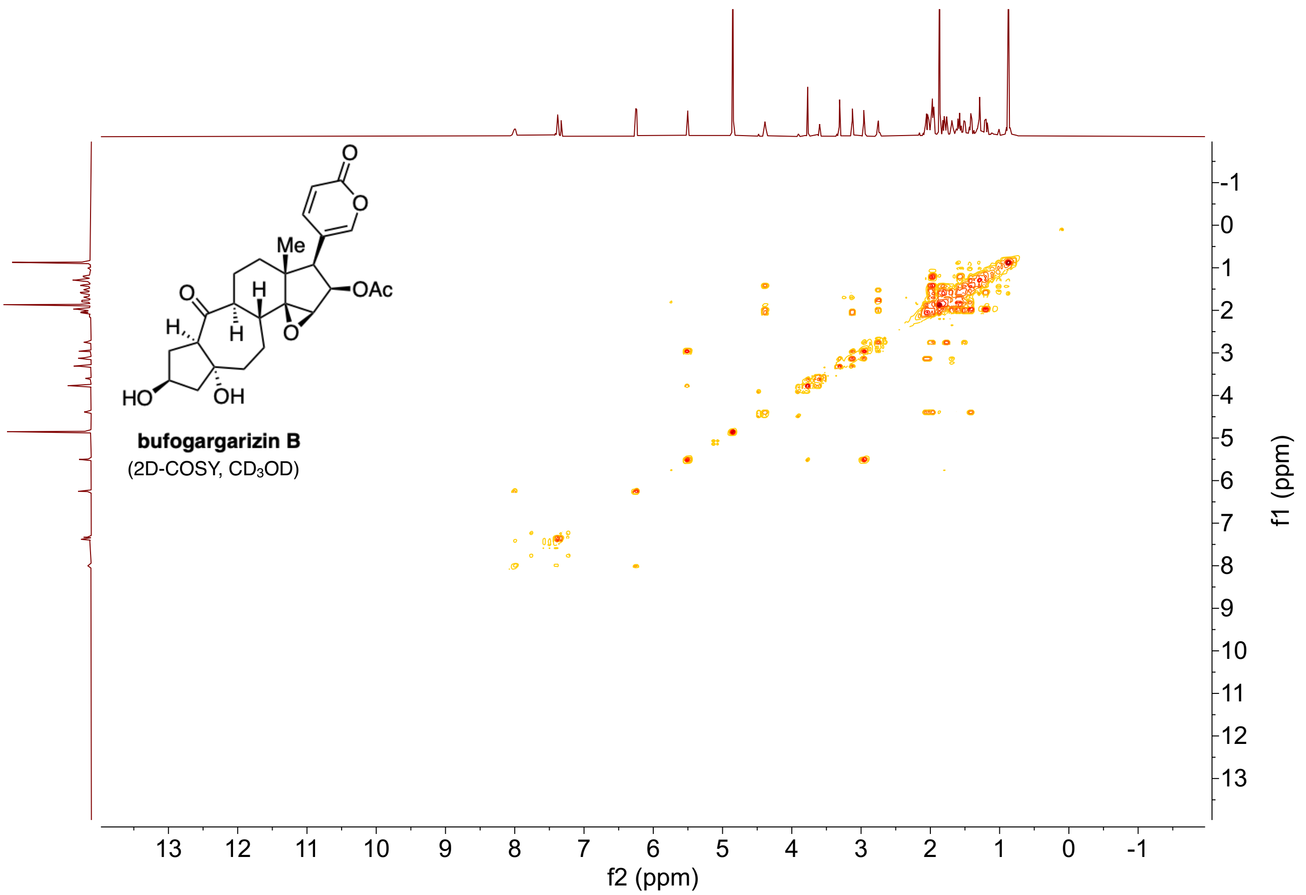
O
O
Me
OAc
O
H
H
H
O
HO
OH
bufogargarizin B
(2D-COSY, CD3OD)
f1 (ppm)
f2 (ppm)

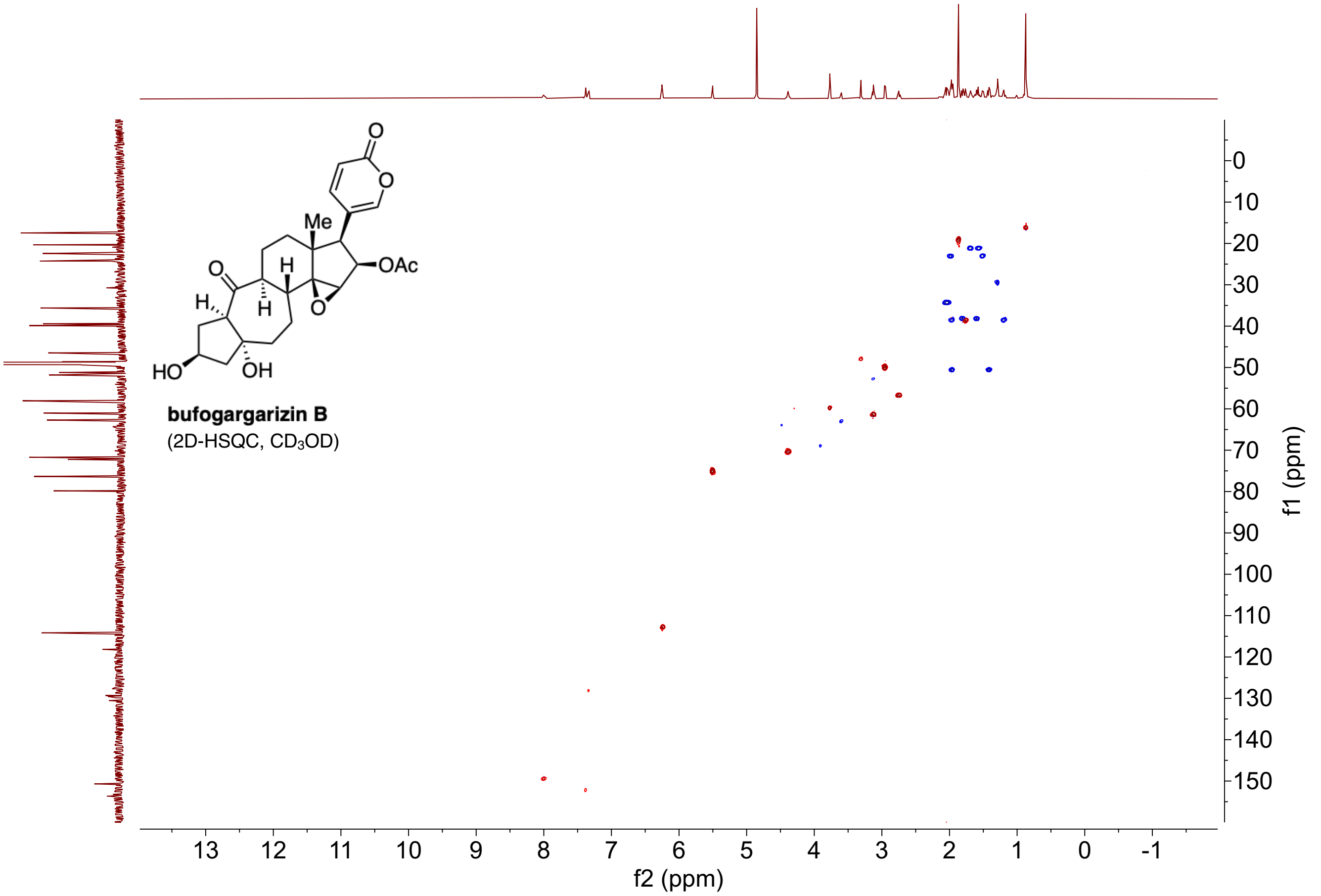
O
O
Me
OAc
O
H
H
H
O
HO
OH
bufogargarizin B
(2D-HSQC, CD3OD)
f1 (ppm)
f2 (ppm)

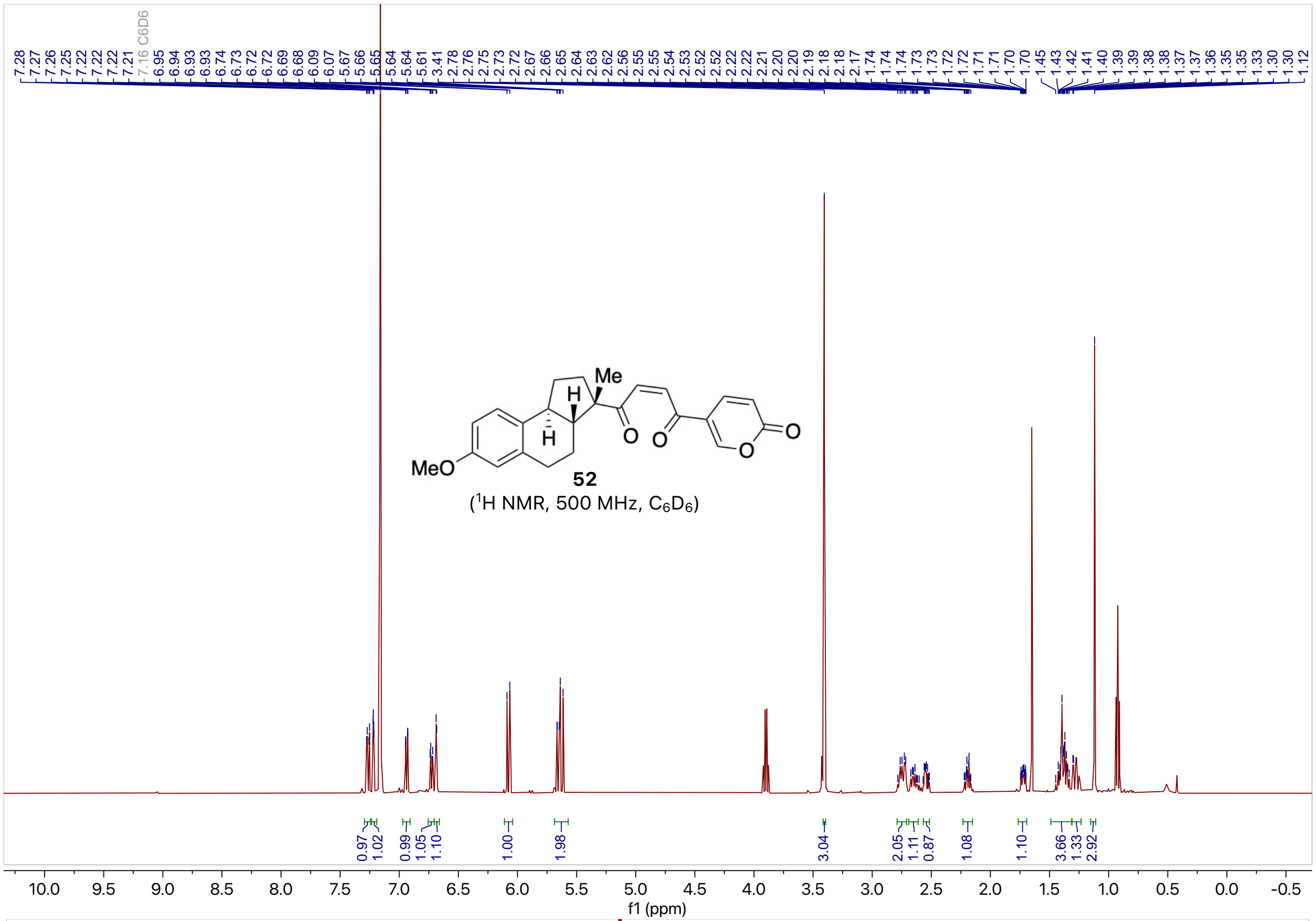

52
(^{1}H NMR, 500 MHz, C_6D_6)

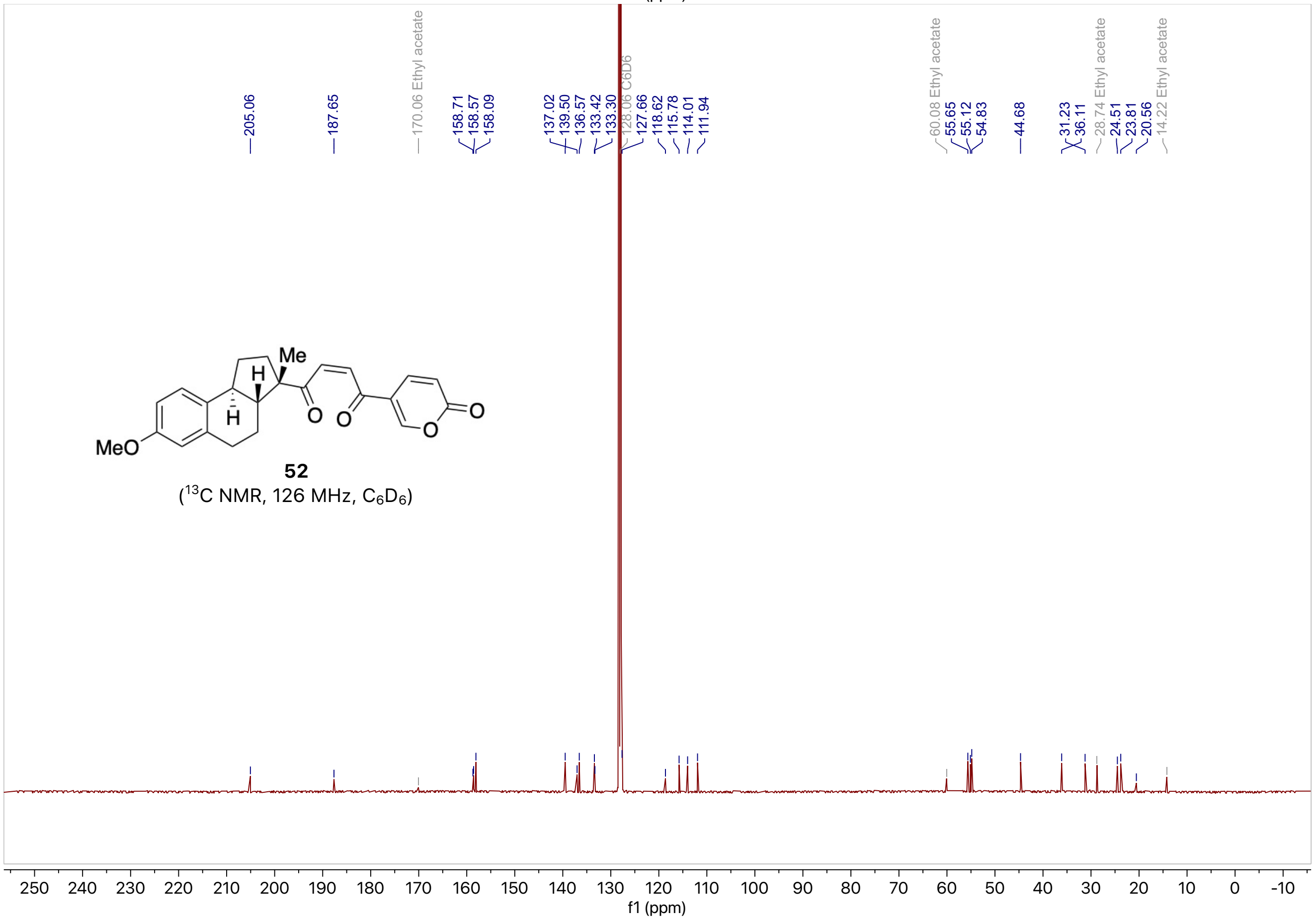

52
(^{13}C NMR, 126 MHz, C_6D_6)

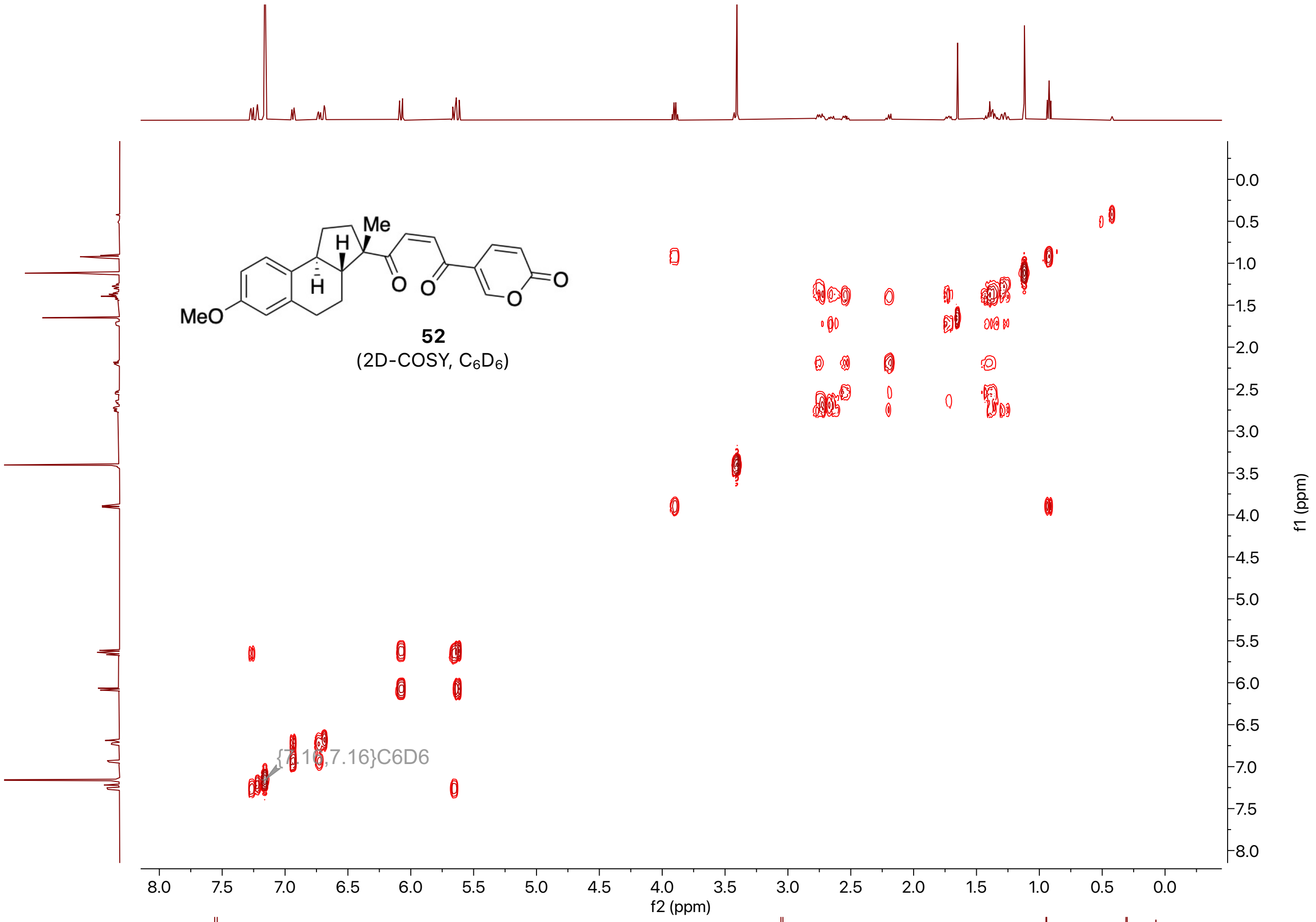
Me
H
H
MeO
O
O
O
O
52
(2D-COSY, C_6D_6)
{7.16,7.16}C6D6
f2 (ppm)
f1 (ppm)

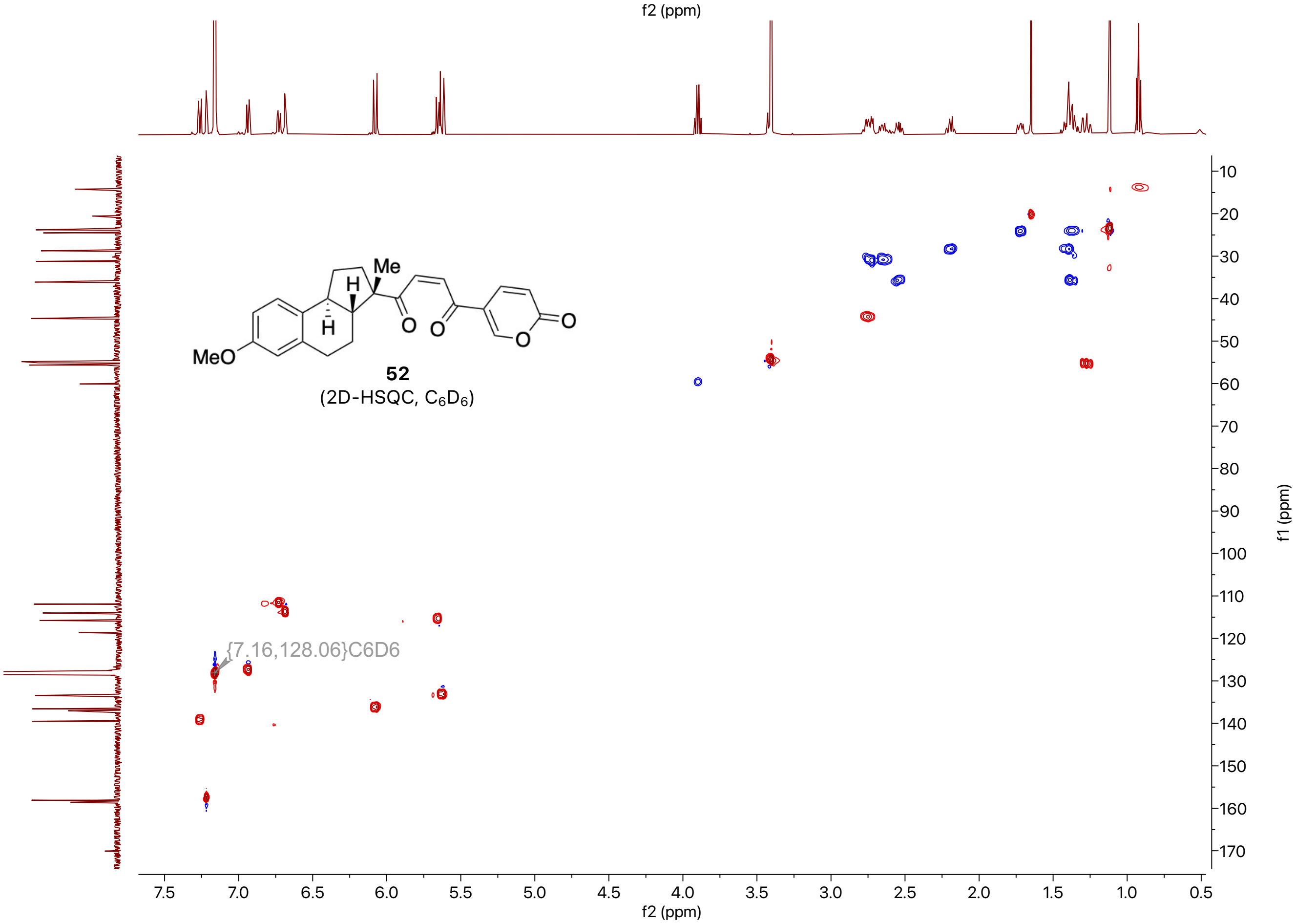
Me
H
H
MeO
O
O
O
O
52
(2D-HSQC, C_6D_6)
{7.16,128.06}C6D6
f2 (ppm)
f1 (ppm)

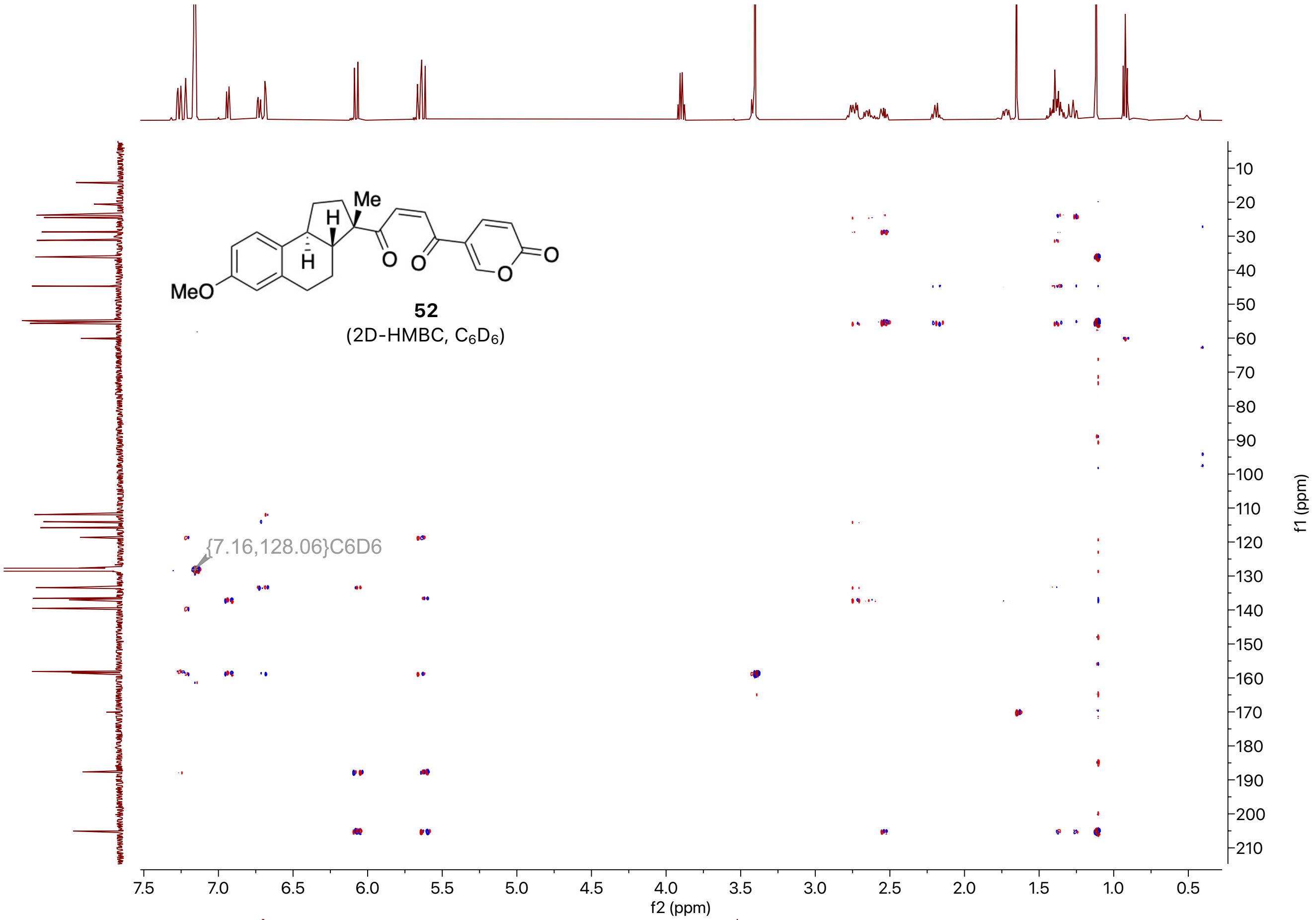
Me
H
H
MeO
O
O
O
O
52
(2D-HMBC, C6D6)
{7.16,128.06}C6D6
f2 (ppm)
f1 (ppm)

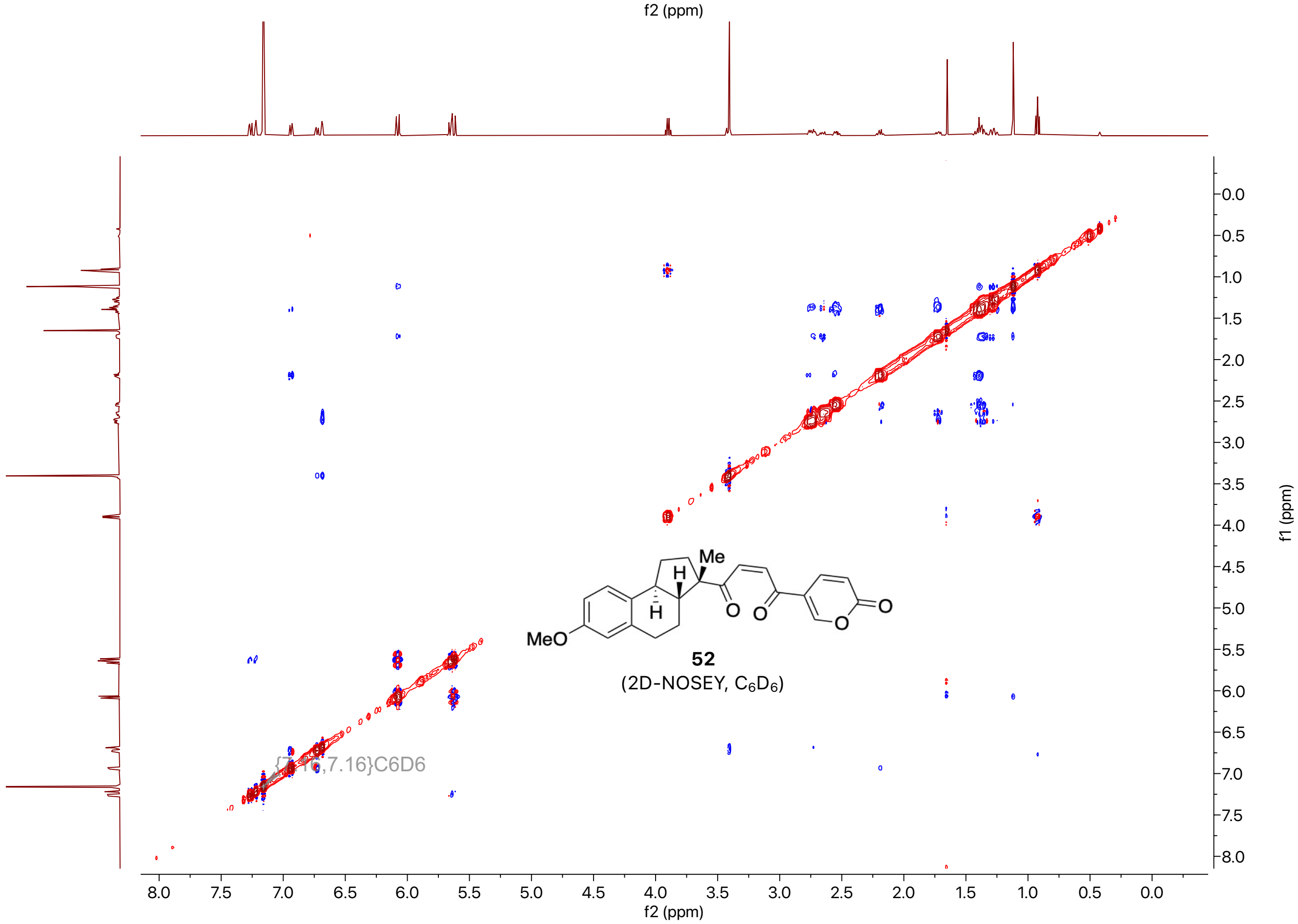
Me
H
H
MeO
O
O
O
O
52
(2D-NOSEY, C6D6)
f2 (ppm)
f1 (ppm)

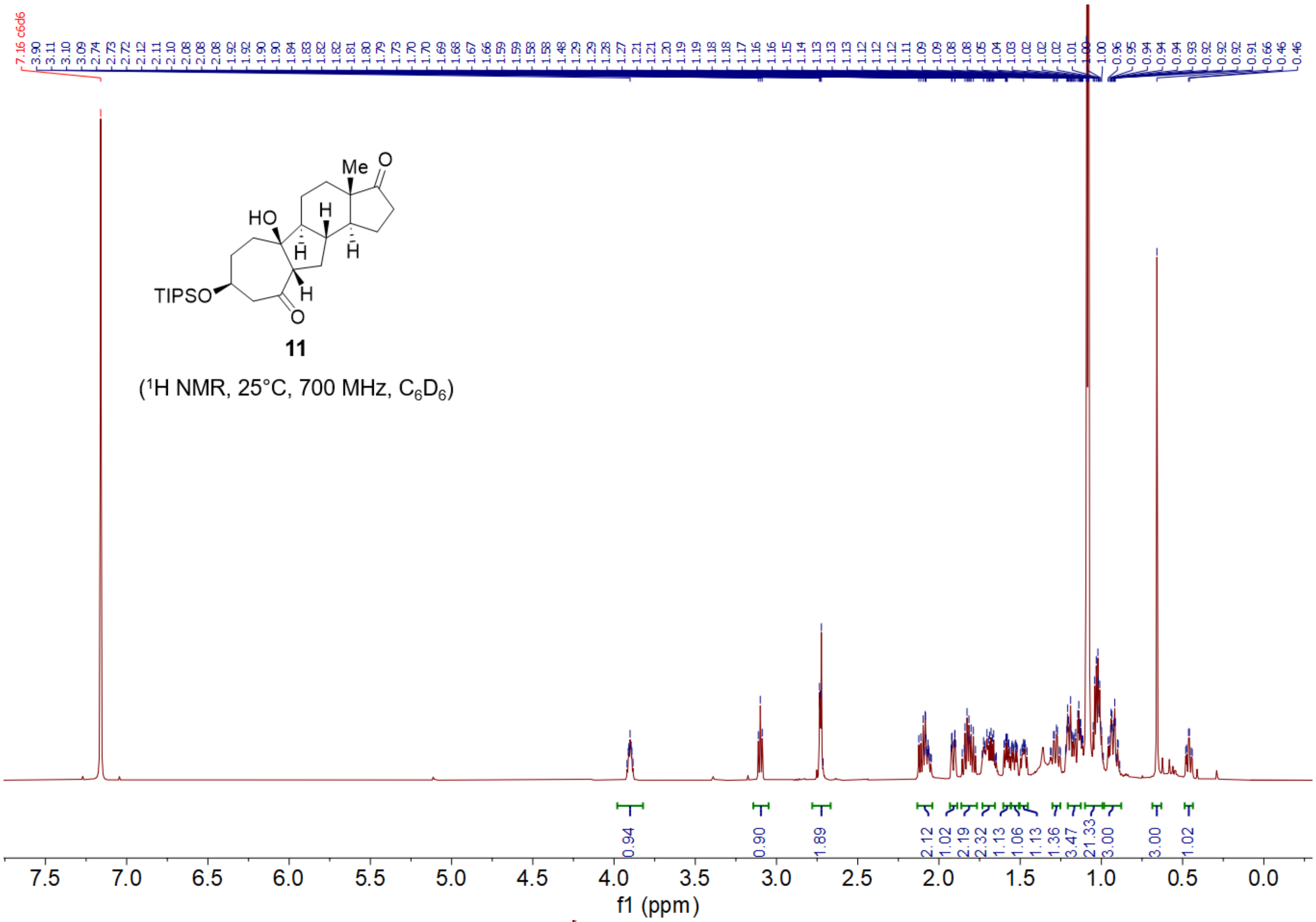
11
(¹H NMR, 25°C, 700 MHz, C6D6)
f1 (ppm)

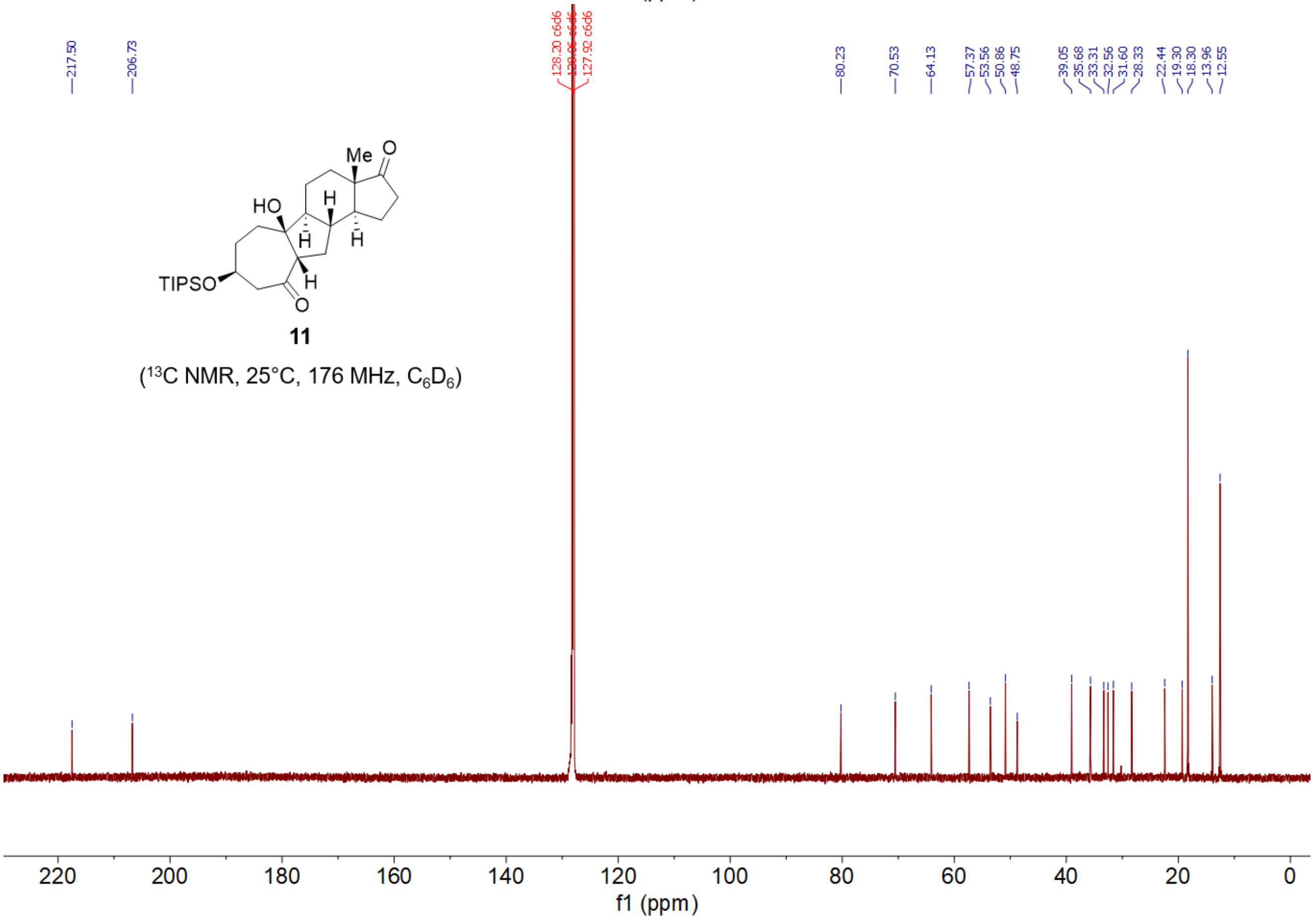
11
(¹³C NMR, 25°C, 176 MHz, C6D6)
f1 (ppm)

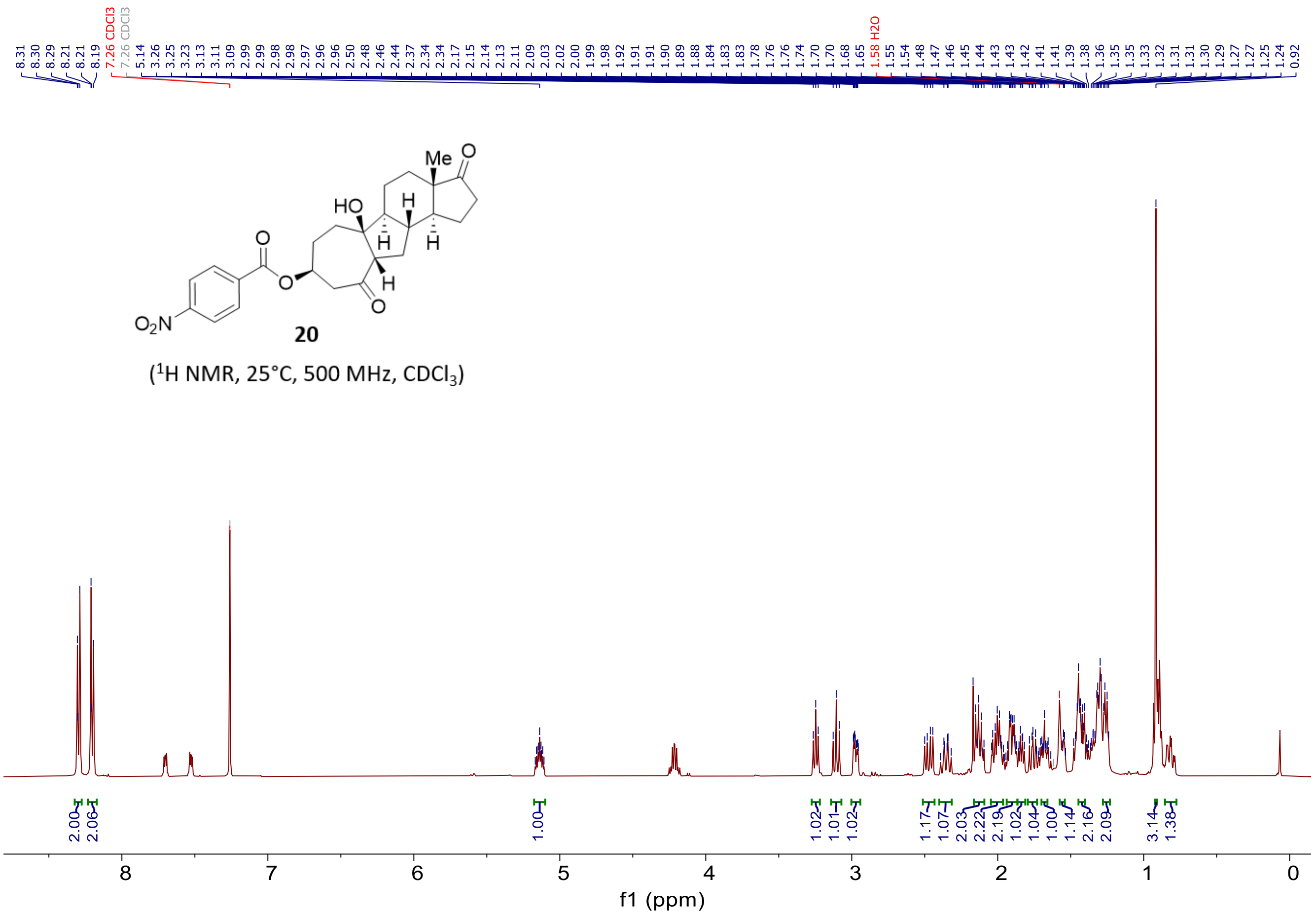
8.31 8.30 8.29 8.21 8.21 8.19 7.26 CDCl3 7.26 CDCl3 5.14 3.26 3.25 3.23 3.13 3.11 3.09 2.99 2.99 2.98 2.98 2.97 2.96 2.96 2.50 2.48 2.46 2.44 2.37 2.34 2.34 2.17 2.15 2.14 2.13 2.11 2.09 2.03 2.02 2.00 1.99 1.98 1.92 1.91 1.91 1.90 1.89 1.88 1.84 1.83 1.83 1.78 1.76 1.76 1.74 1.70 1.70 1.68 1.65 1.58 H2O 1.55 1.54 1.48 1.47 1.46 1.45 1.44 1.43 1.42 1.41 1.41 1.39 1.38 1.36 1.35 1.33 1.32 1.31 1.31 1.30 1.29 1.27 1.25 1.24 0.92
Me
HO
H
H
H
H
O
O
O
O
O₂N
20
(¹H NMR, 25°C, 500 MHz, CDCl₃)
2.00 2.06 1.00 1.02 1.01 1.02 1.17 1.07 2.03 2.22 2.19 1.02 1.04 1.00 1.14 2.16 2.09 3.14 1.38
8 7 6 5 4 3 2 1 0
f1 (ppm)

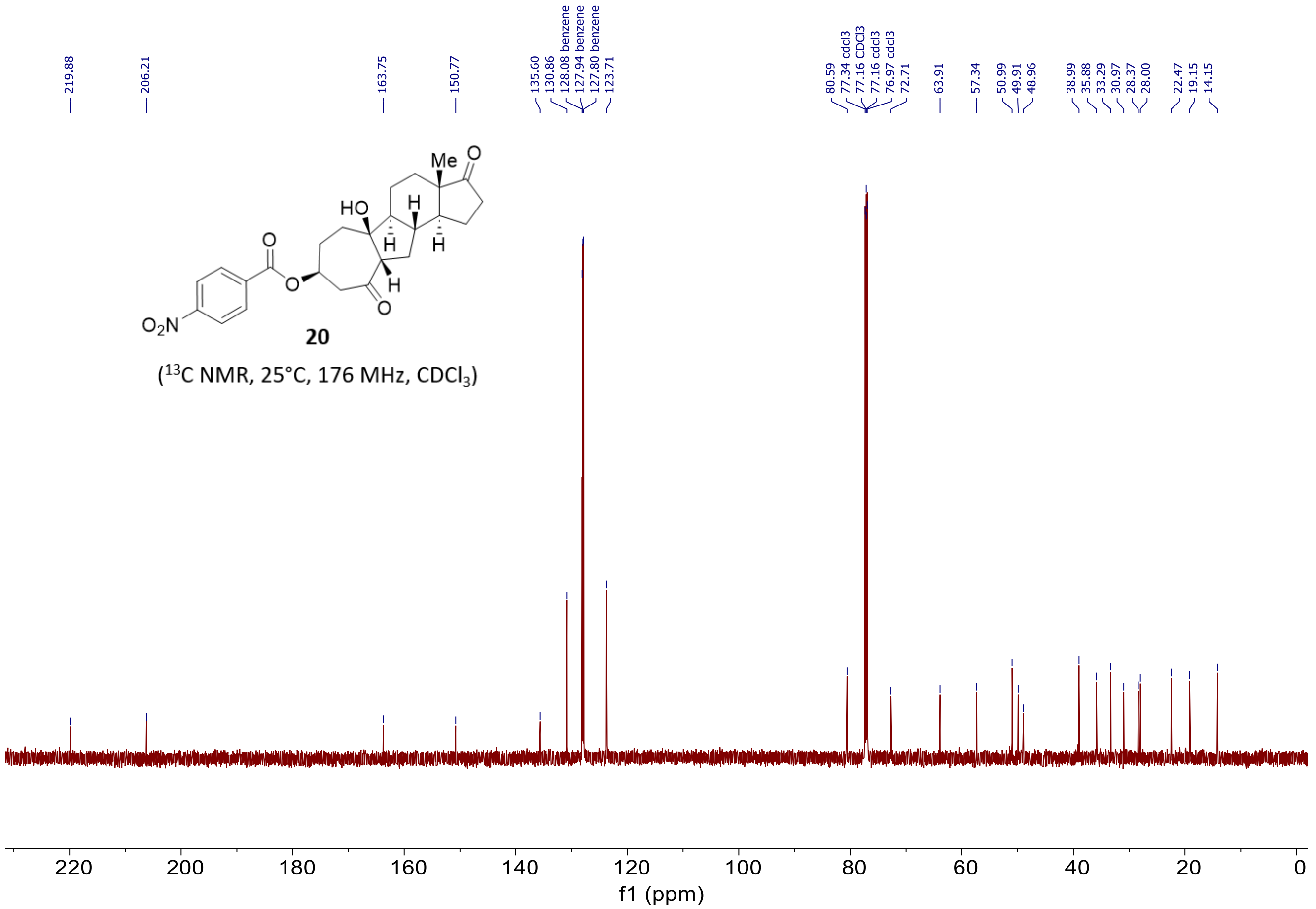
219.88 206.21 163.75 150.77 135.60 130.86 128.08 benzene 127.94 benzene 127.80 benzene 123.71 80.59 77.34 cdcl3 77.16 CDCl3 77.16 cdcl3 76.97 cdcl3 72.71 63.91 57.34 50.99 49.91 48.96 38.99 35.88 33.29 30.97 28.37 28.00 22.47 19.15 14.15
Me
HO
H
H
H
H
O
O
O
O
O₂N
20
(¹³C NMR, 25°C, 176 MHz, CDCl₃)
220 200 180 160 140 120 100 80 60 40 20 0
f1 (ppm)

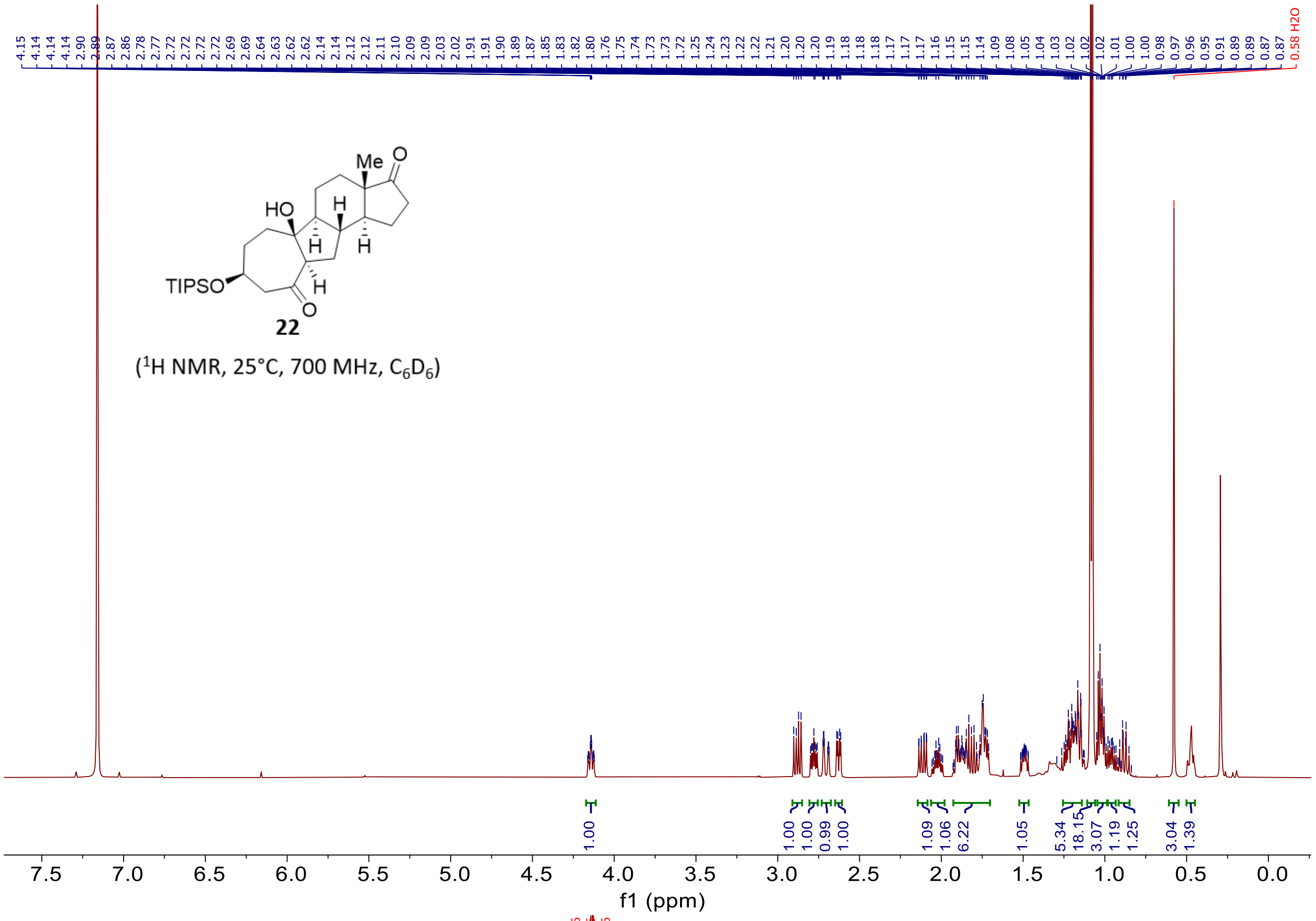
Me
HO
TIPSO
H
22
(1H NMR, 25°C, 700 MHz, C6D6)
0.58 H2O
f1 (ppm)

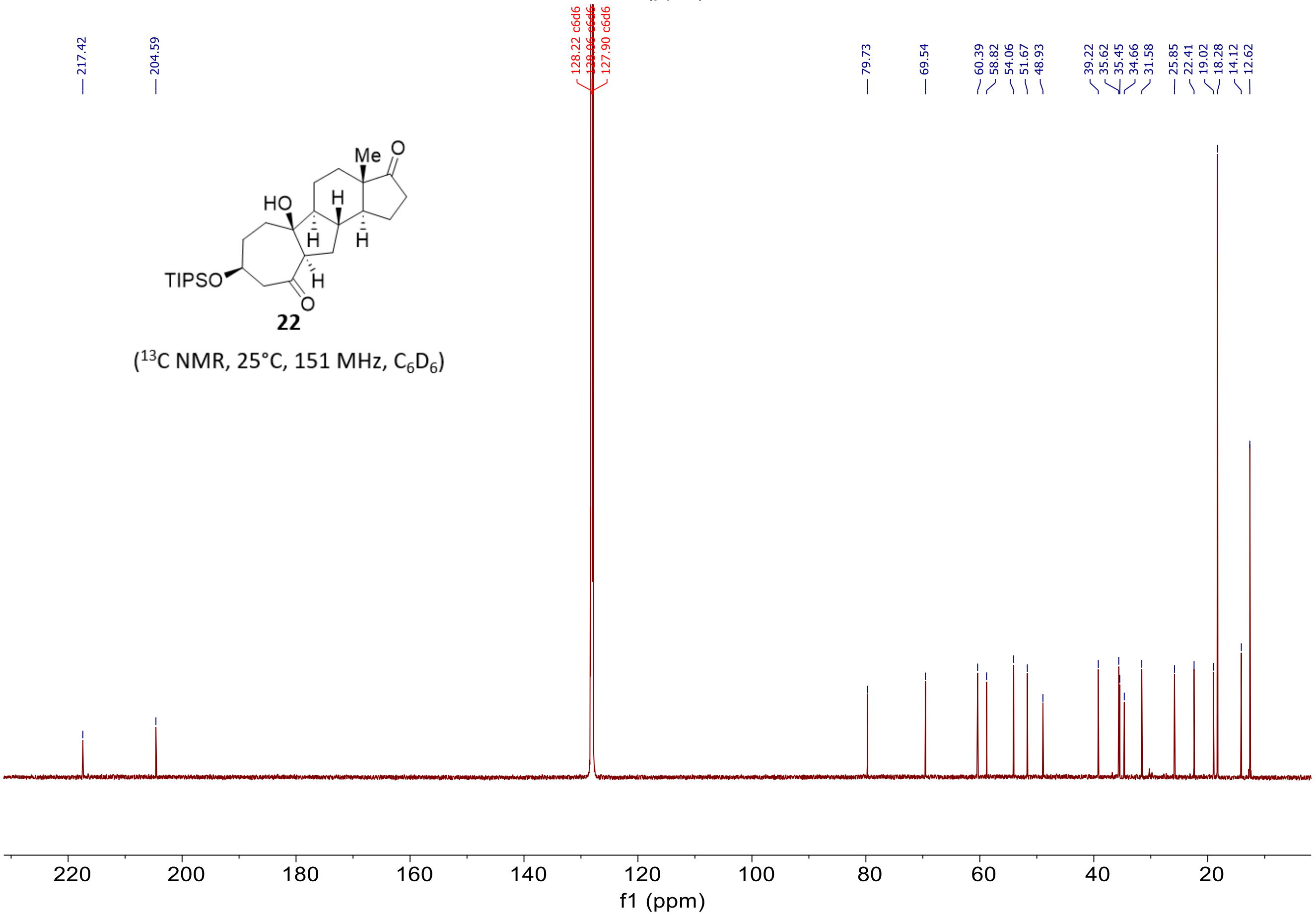
217.42
204.59
128.22 c6d6
128.06 c6d6
127.90 c6d6
79.73
69.54
60.39
58.82
54.06
51.67
48.93
39.22
35.62
35.45
34.66
31.58
25.85
22.41
19.02
18.28
14.12
12.62
Me
HO
TIPSO
H
22
(13C NMR, 25°C, 151 MHz, C6D6)
f1 (ppm)

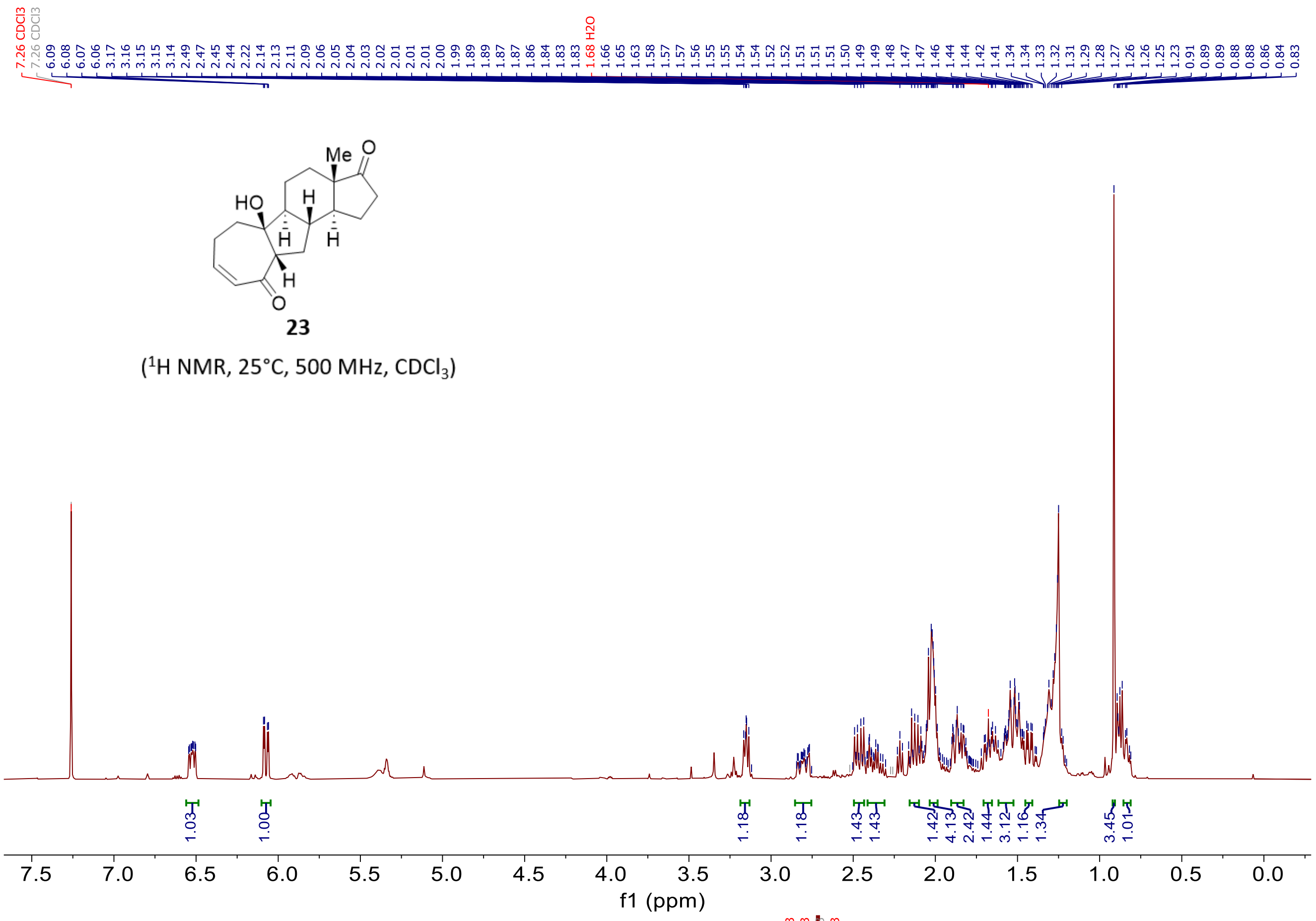

23

(1H NMR, 25°C, 500 MHz, $CDCl_3$)

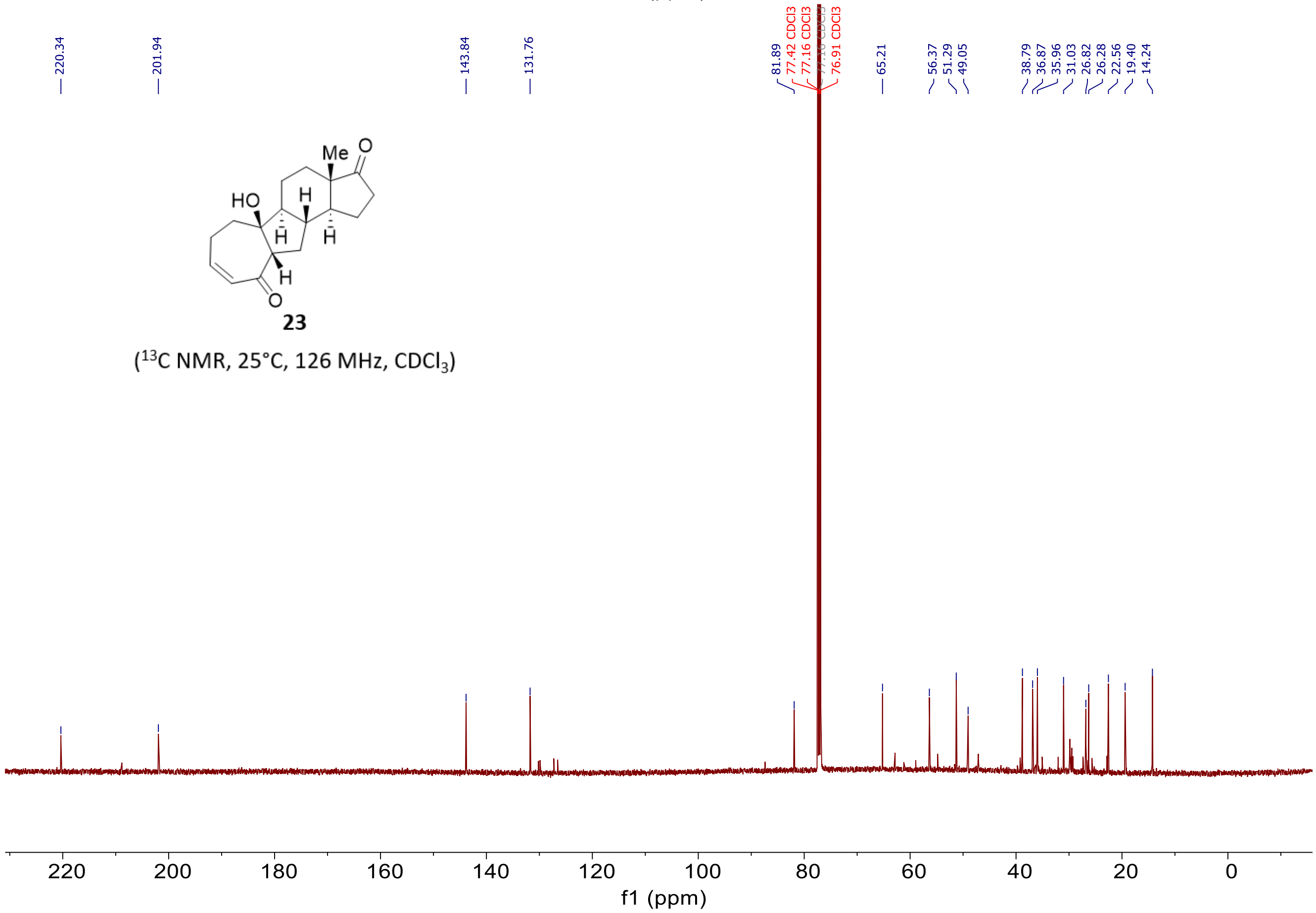

23

(13C NMR, 25°C, 126 MHz, $CDCl_3$)

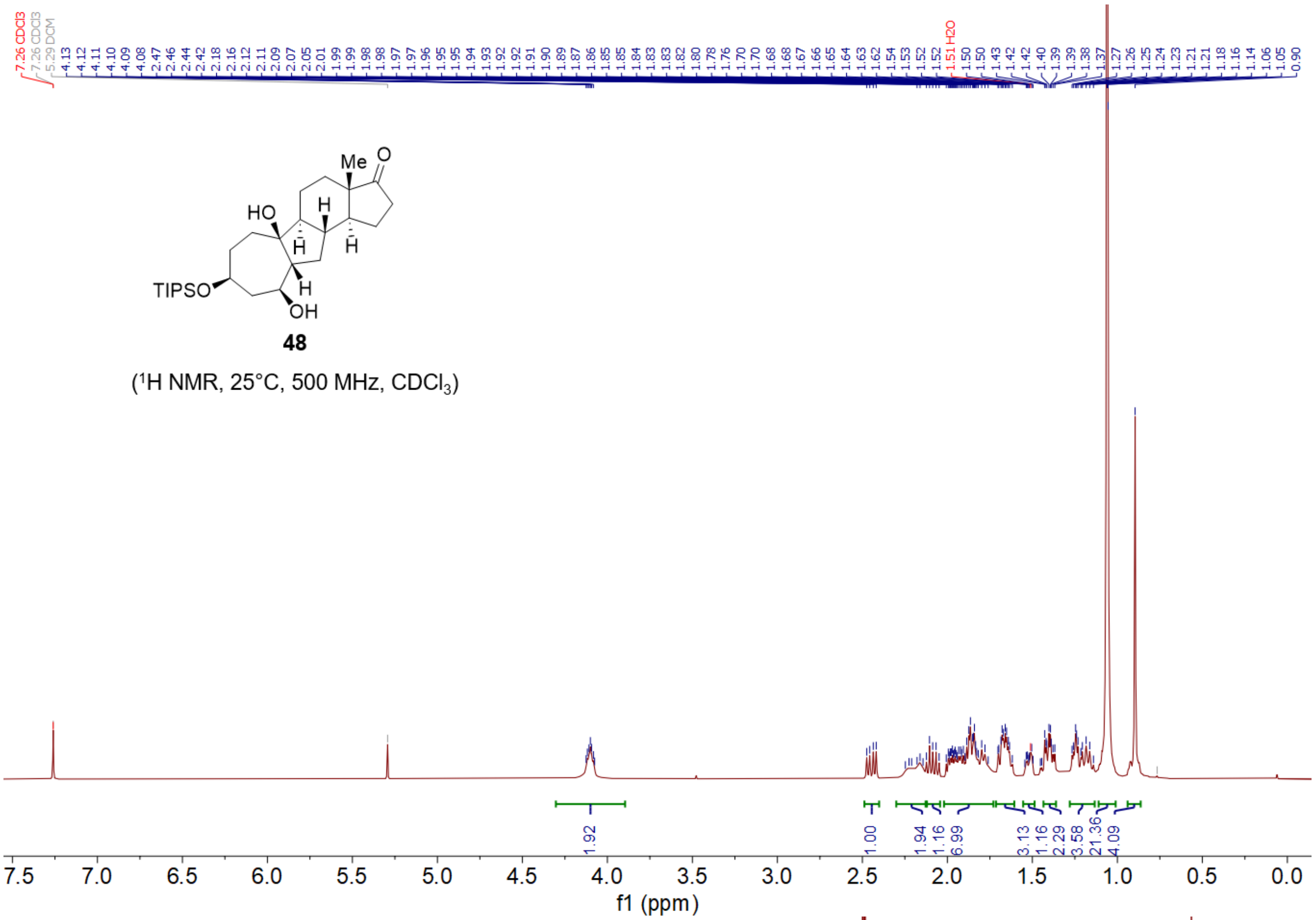

48

(^{1}H NMR, 25°C, 500 MHz, $CDCl_3$)

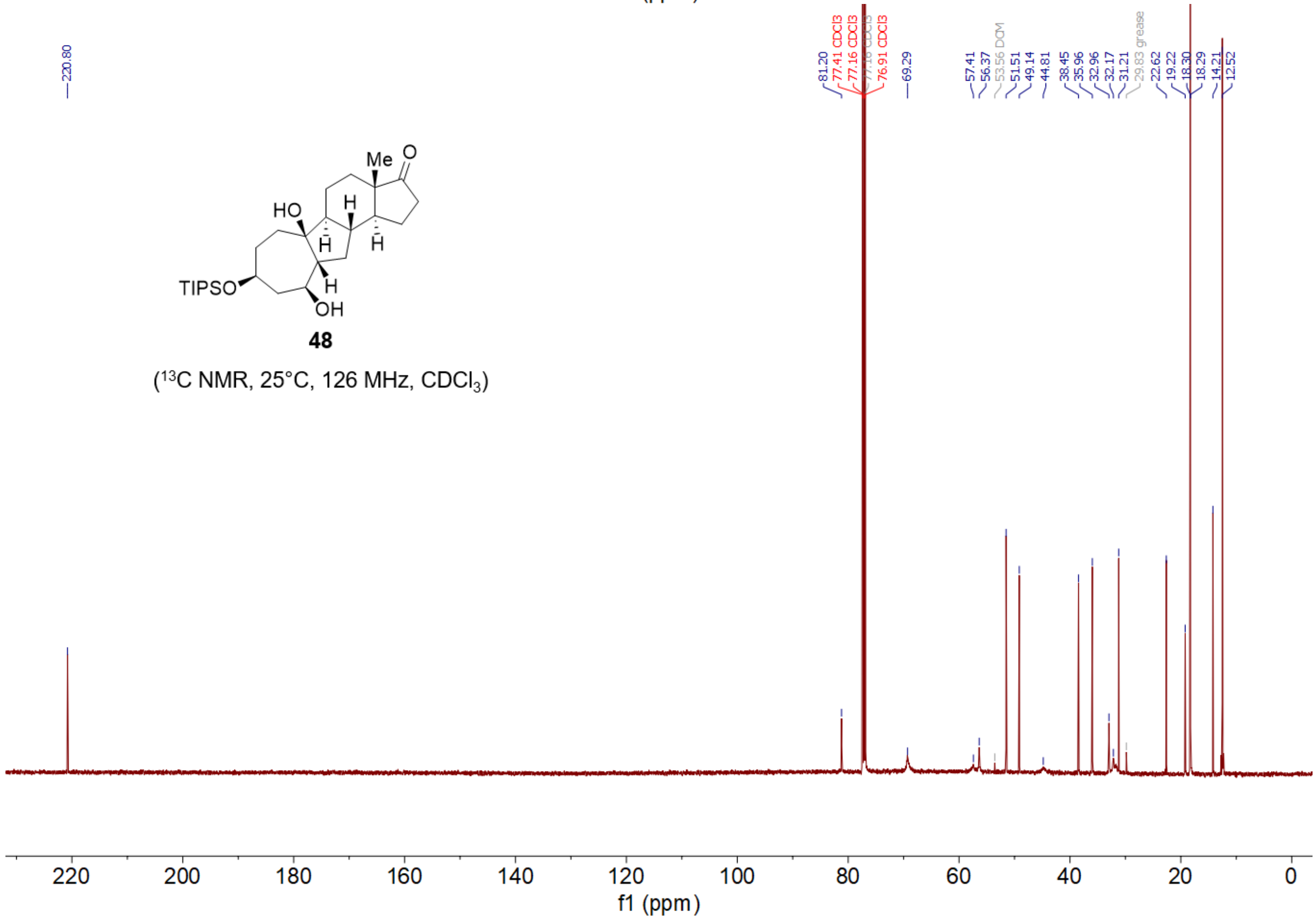

48

(^{13}C NMR, 25°C, 126 MHz, $CDCl_3$)

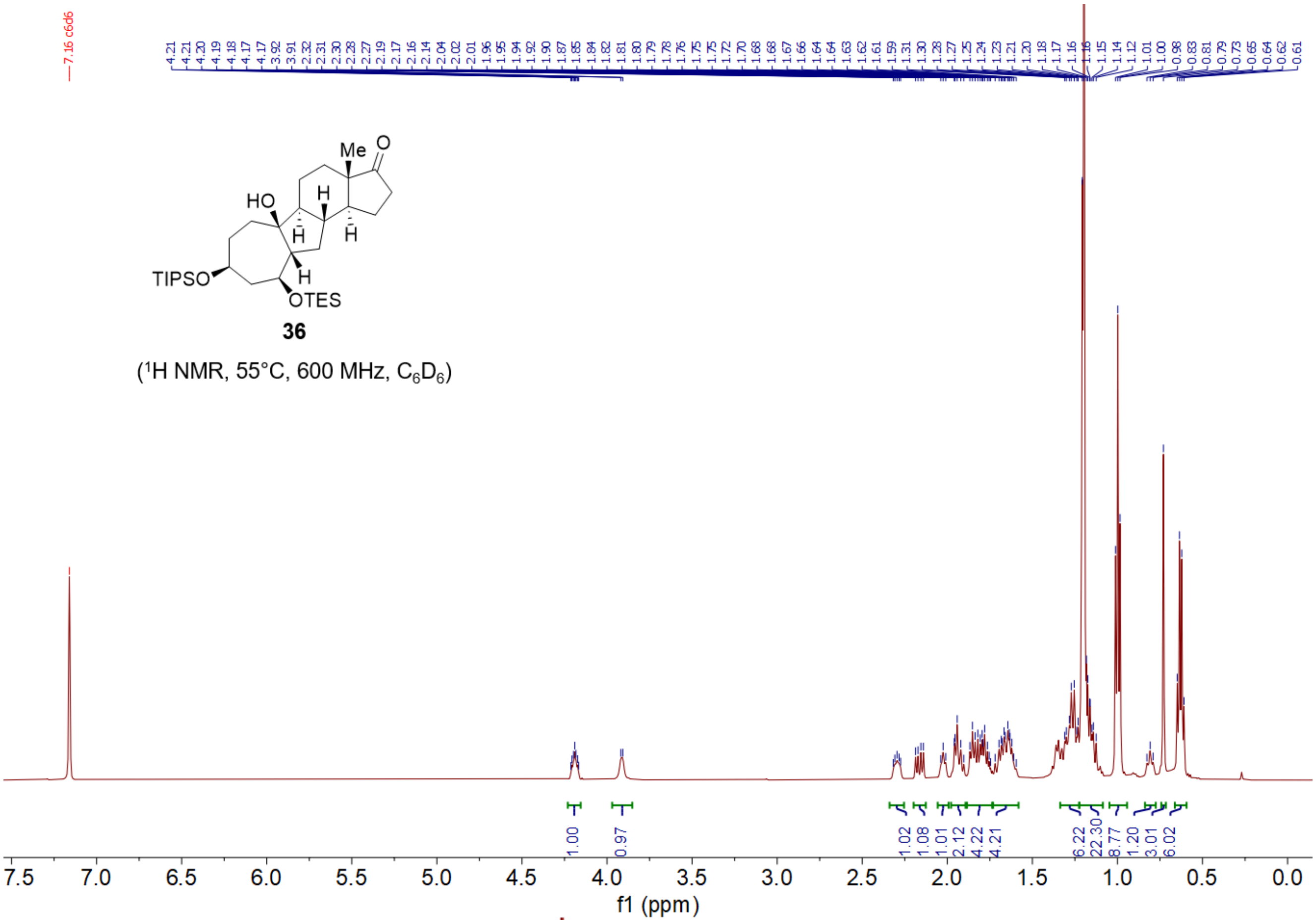
7.16 c6d6
Me
O
HO
H
H
H
TIPSO
H
OTES
36
(¹H NMR, 55°C, 600 MHz, C6D6)
1.00
0.97
1.02
1.08
1.01
2.12
4.22
4.21
6.22
22.30
8.77
1.20
3.01
6.02
f1 (ppm)

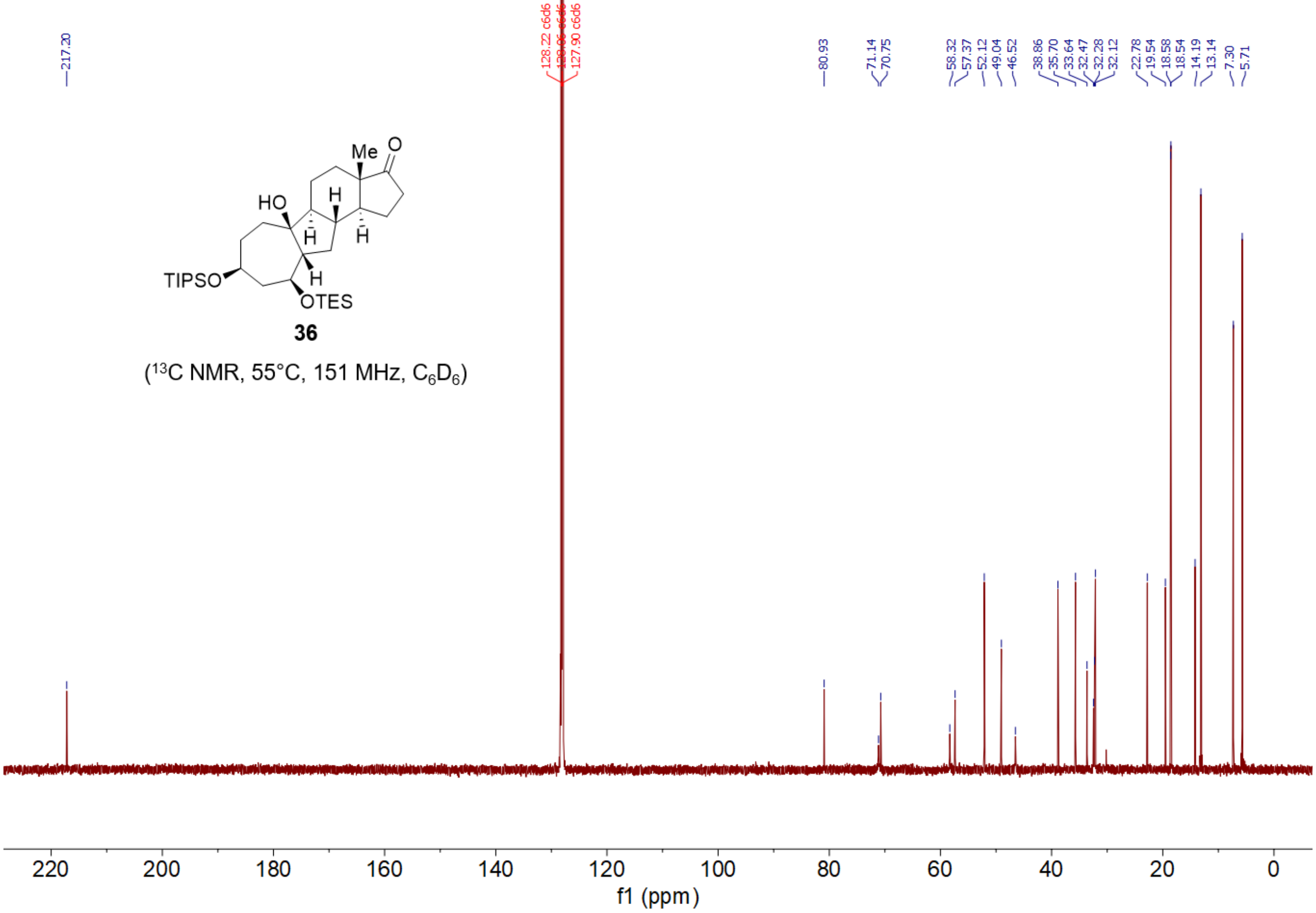
217.20
128.22 c6d6
127.90 c6d6
80.93
71.14
70.75
58.32
57.37
52.12
49.04
46.52
38.86
35.70
33.64
32.47
32.28
32.12
22.78
19.54
18.58
18.54
14.19
13.14
7.30
5.71
Me
O
HO
H
H
H
TIPSO
H
OTES
36
(¹³C NMR, 55°C, 151 MHz, C6D6)
f1 (ppm)

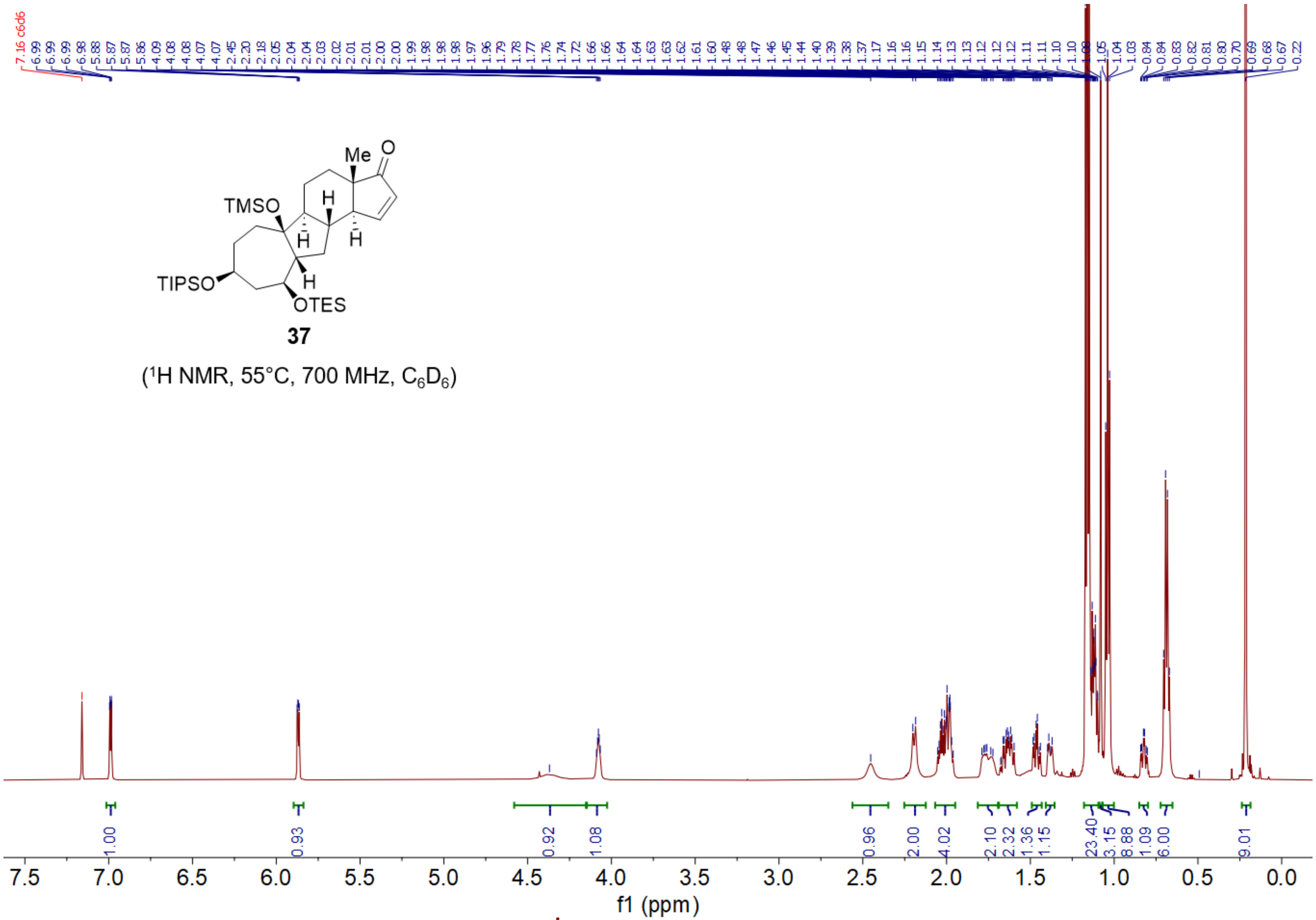
Me
O
TMSO
H
H
H
TIPSO
H
OTES
37
(1H NMR, 55°C, 700 MHz, C6D6)
f1 (ppm)

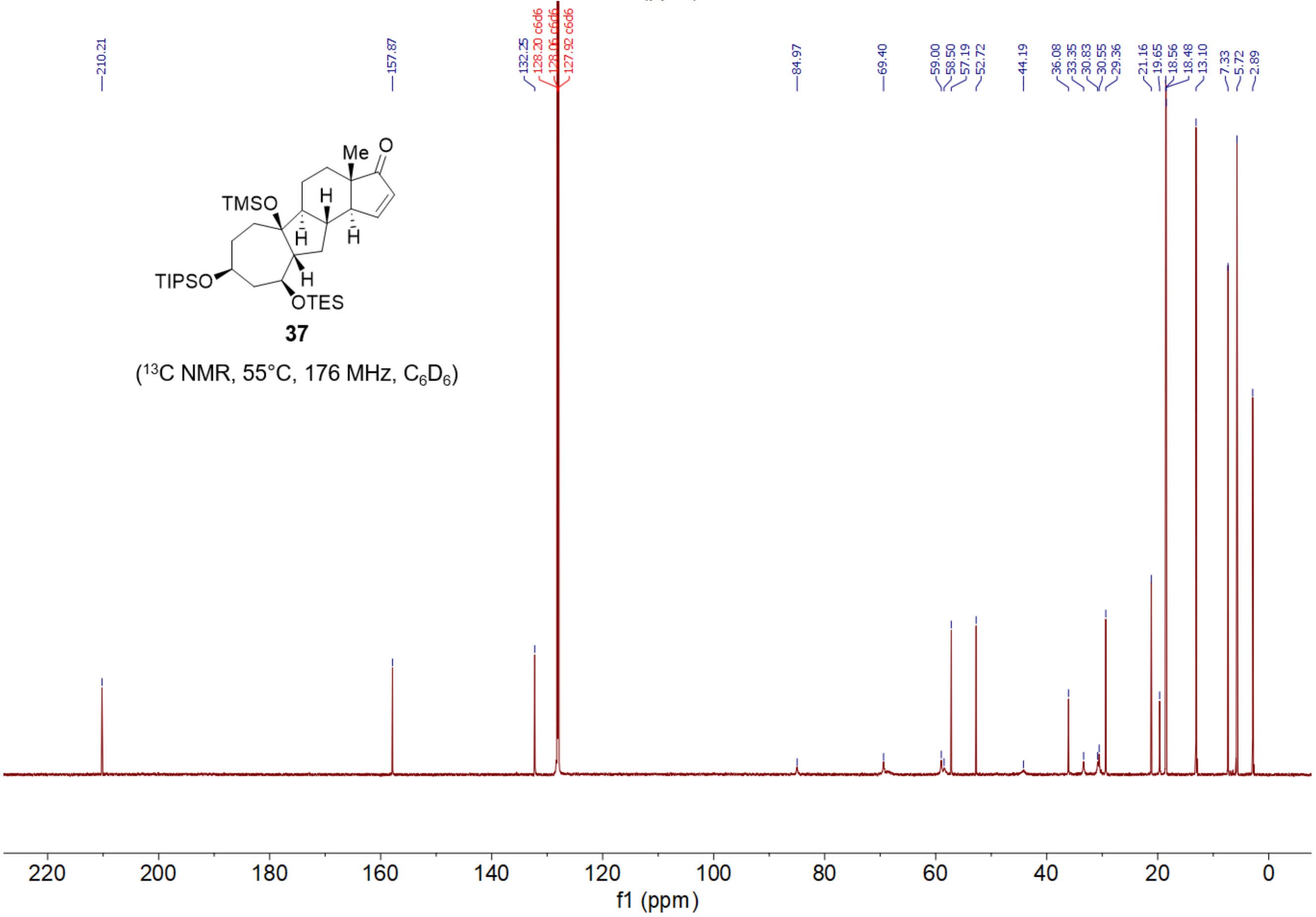
210.21
157.87
132.25
128.20 c6d6
128.06 c6d6
127.92 c6d6
84.97
69.40
59.00
58.50
57.19
52.72
44.19
36.08
33.35
30.83
30.55
29.36
21.16
19.65
18.56
18.48
13.10
7.33
5.72
2.89
Me
O
TMSO
H
H
H
TIPSO
H
OTES
37
(13C NMR, 55°C, 176 MHz, C6D6)
f1 (ppm)

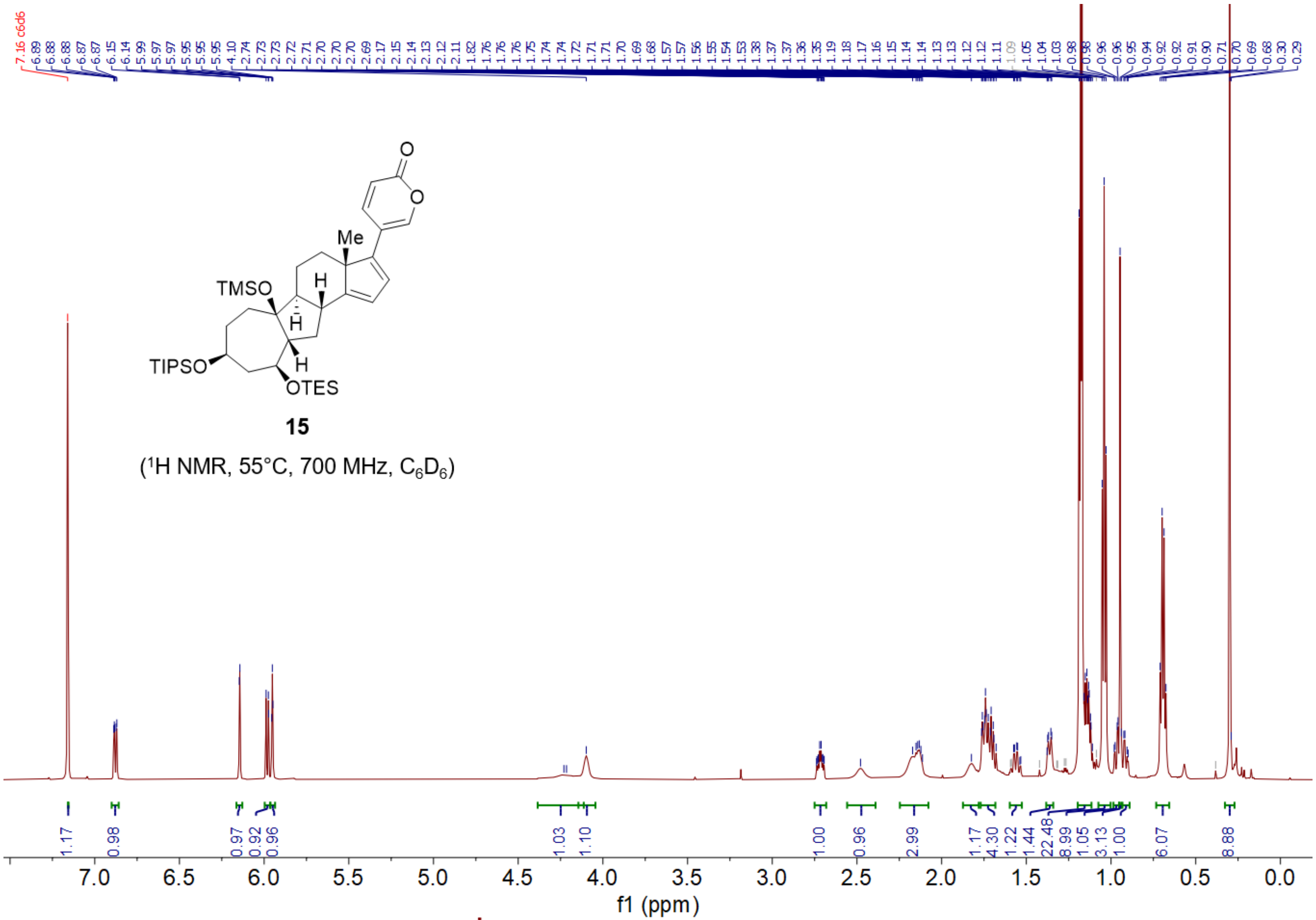

15

(^{1}H NMR, 55°C, 700 MHz, C_6D_6)

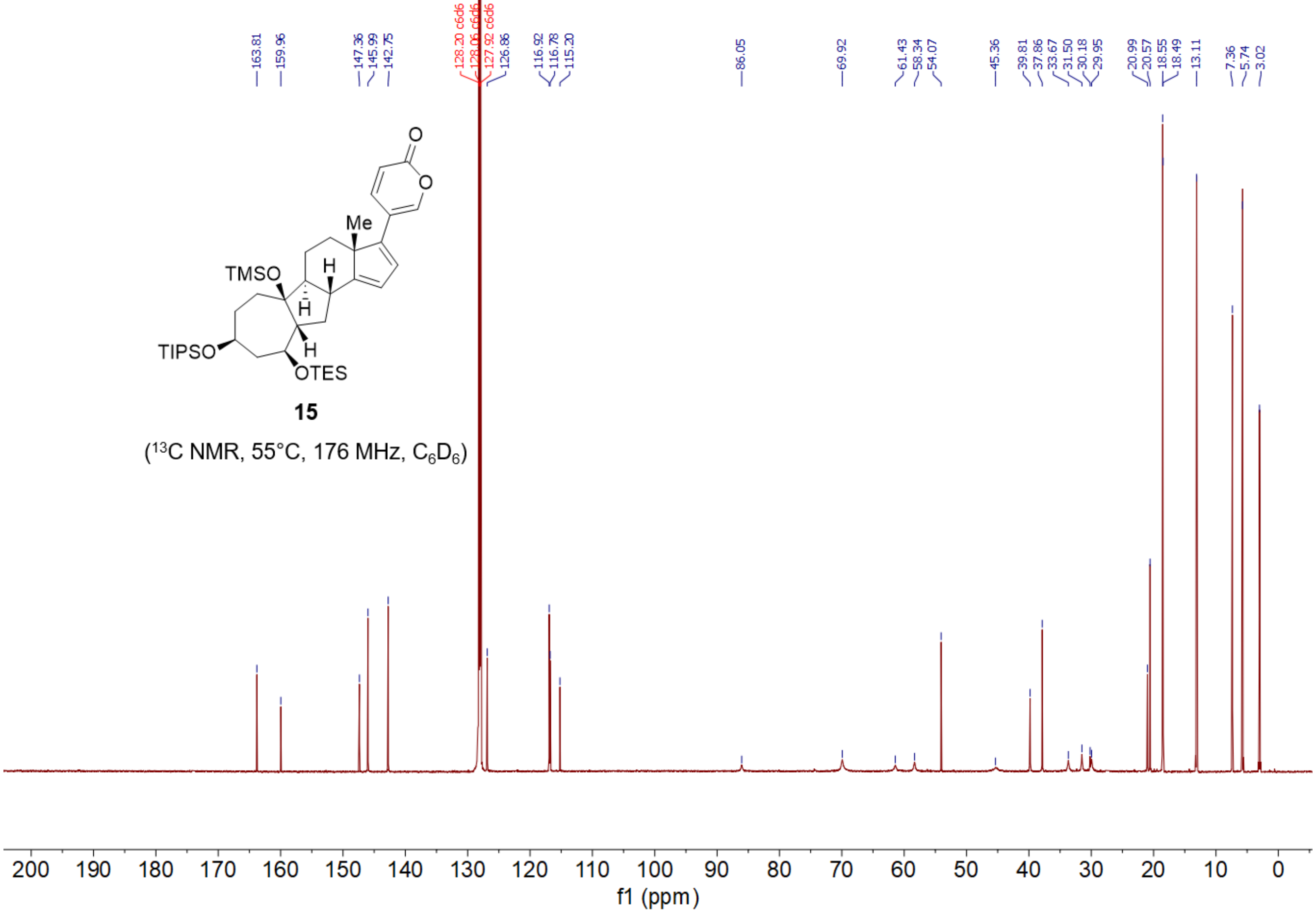

15

(^{13}C NMR, 55°C, 176 MHz, C_6D_6)

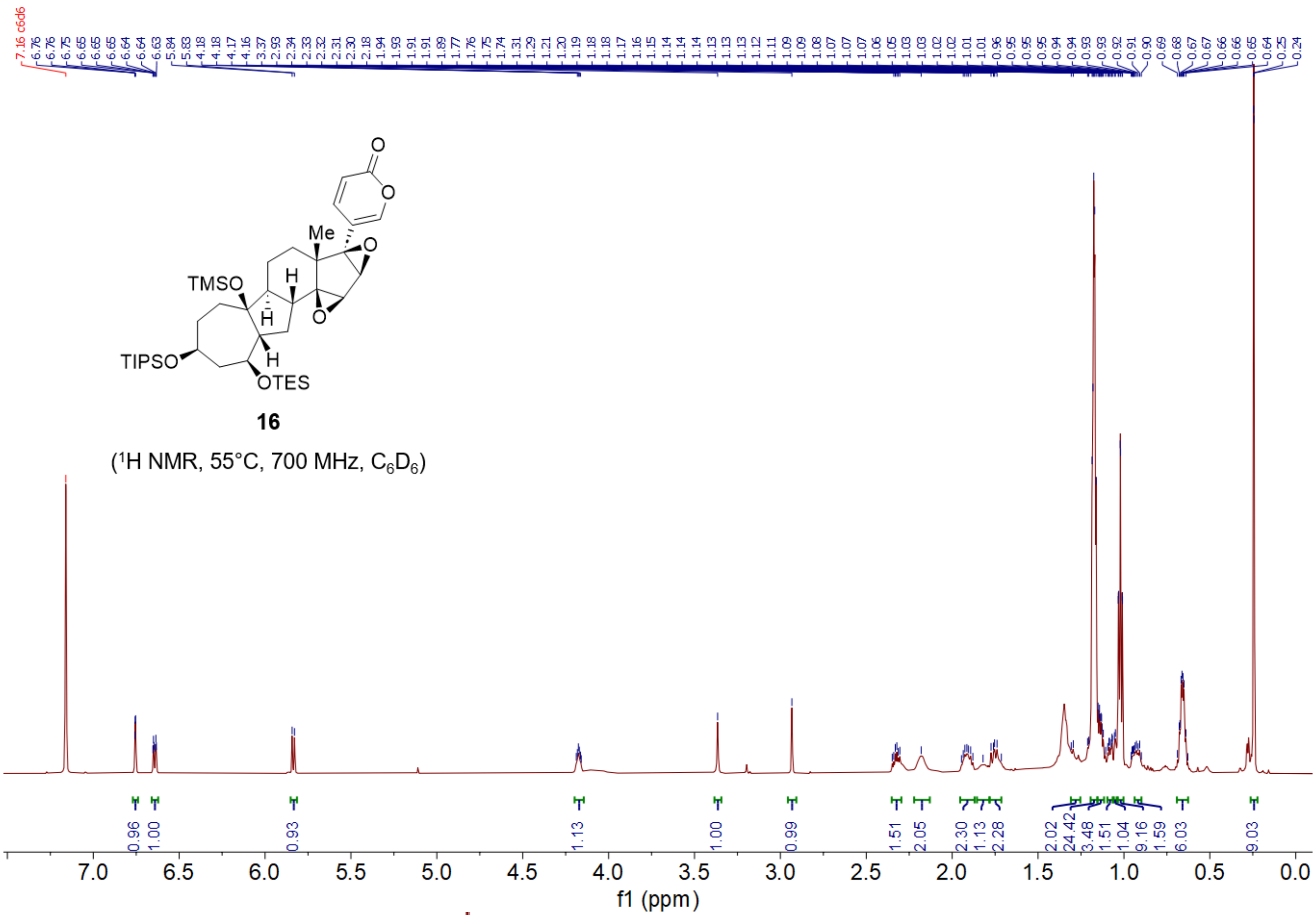

16

(^{1}H NMR, 55°C, 700 MHz, C_6D_6)

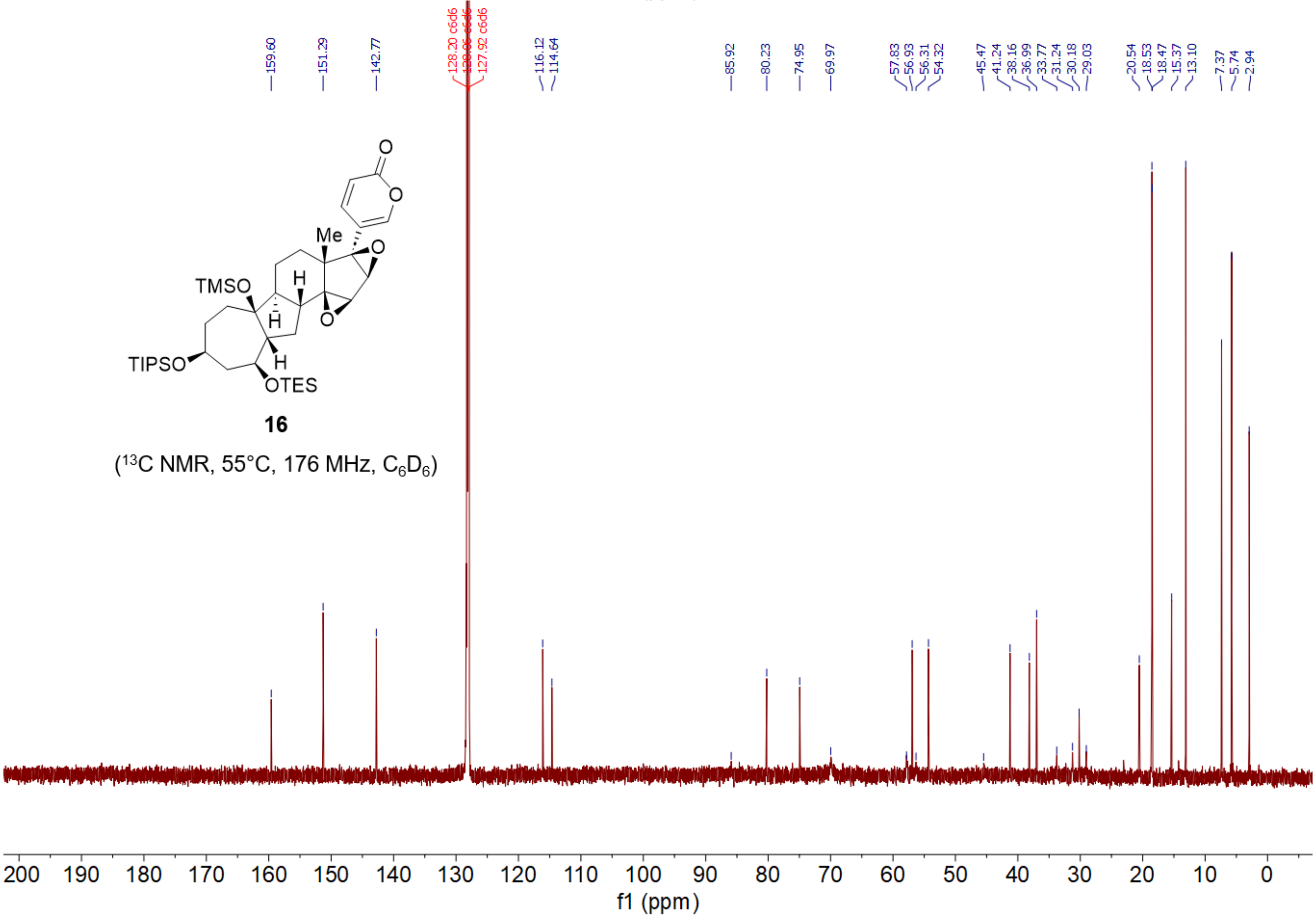

16

(^{13}C NMR, 55°C, 176 MHz, C_6D_6)

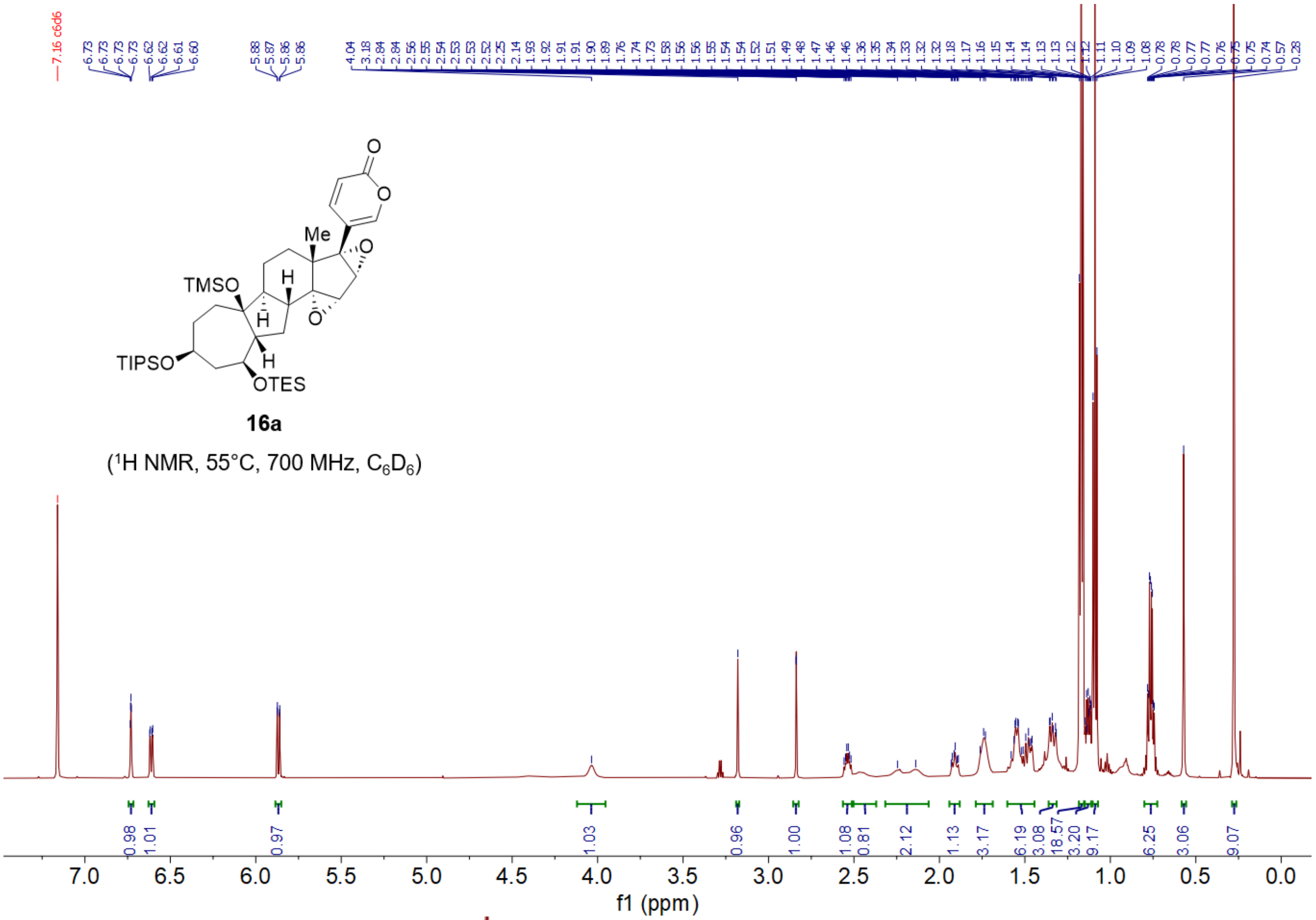
TMSO
TIPSO
OTES
Me
16a
(1H NMR, 55°C, 700 MHz, C6D6)
f1 (ppm)

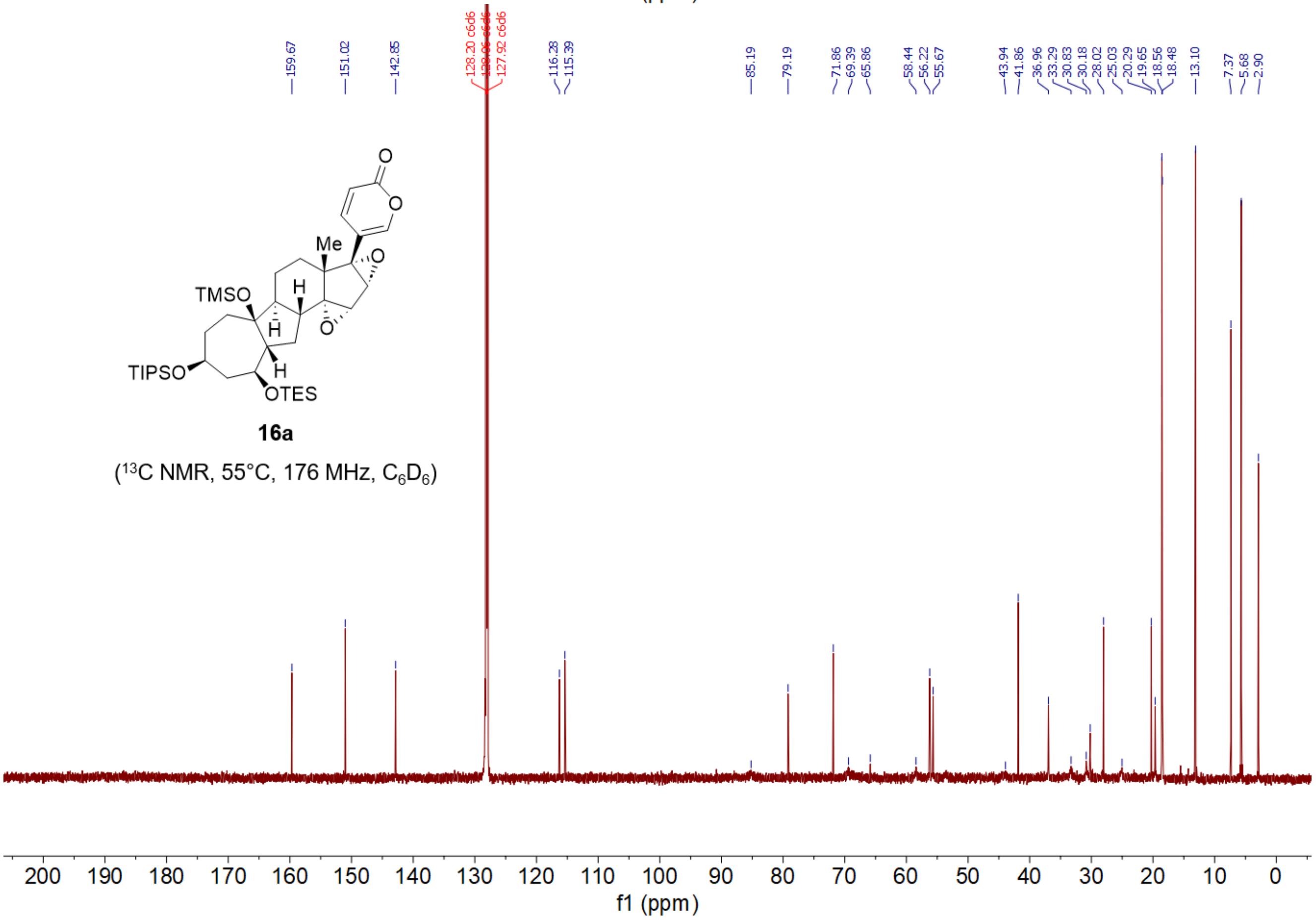
TMSO
TIPSO
OTES
Me
16a
(13C NMR, 55°C, 176 MHz, C6D6)
f1 (ppm)

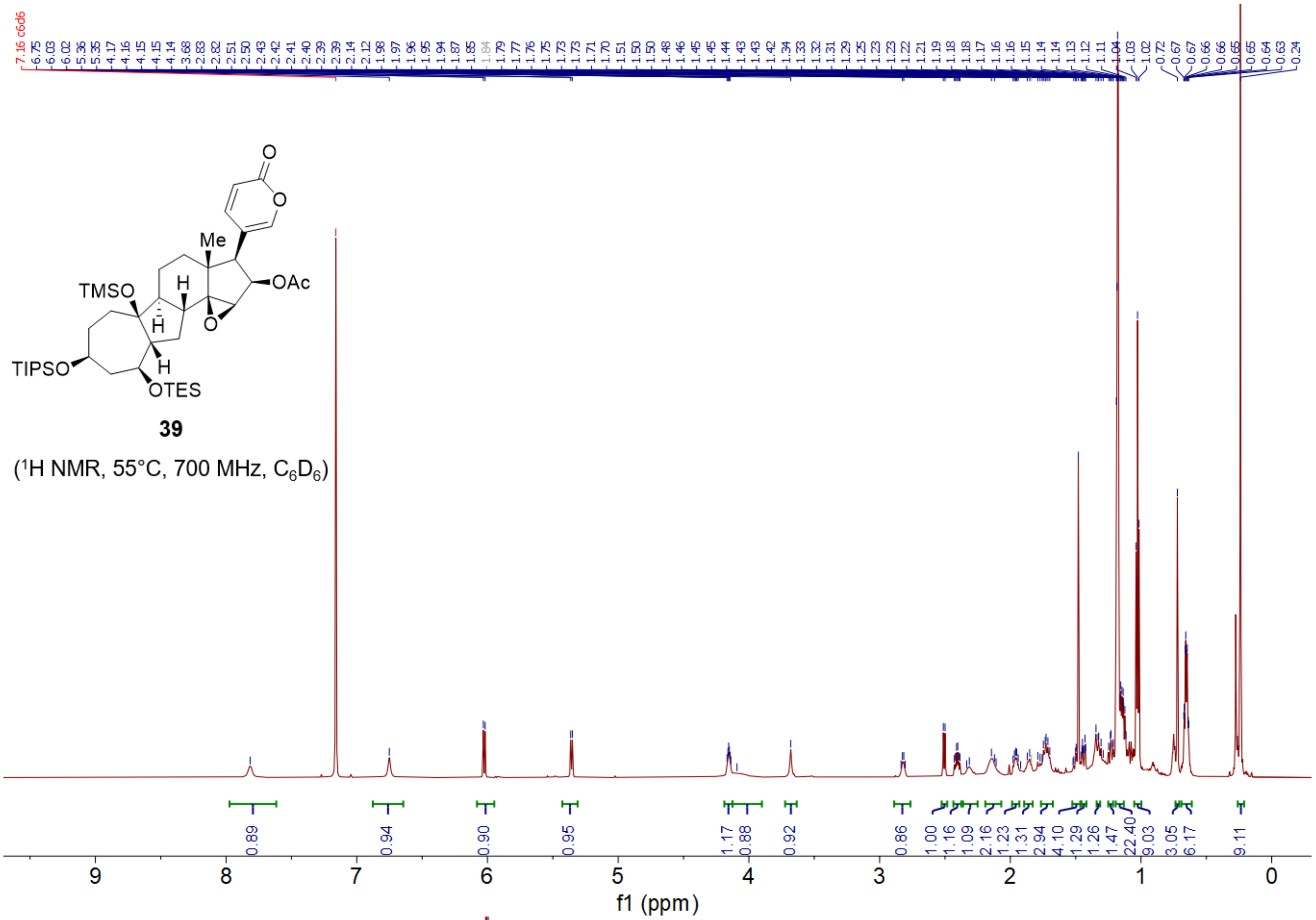

39

(^{1}H NMR, 55°C, 700 MHz, C_6D_6)

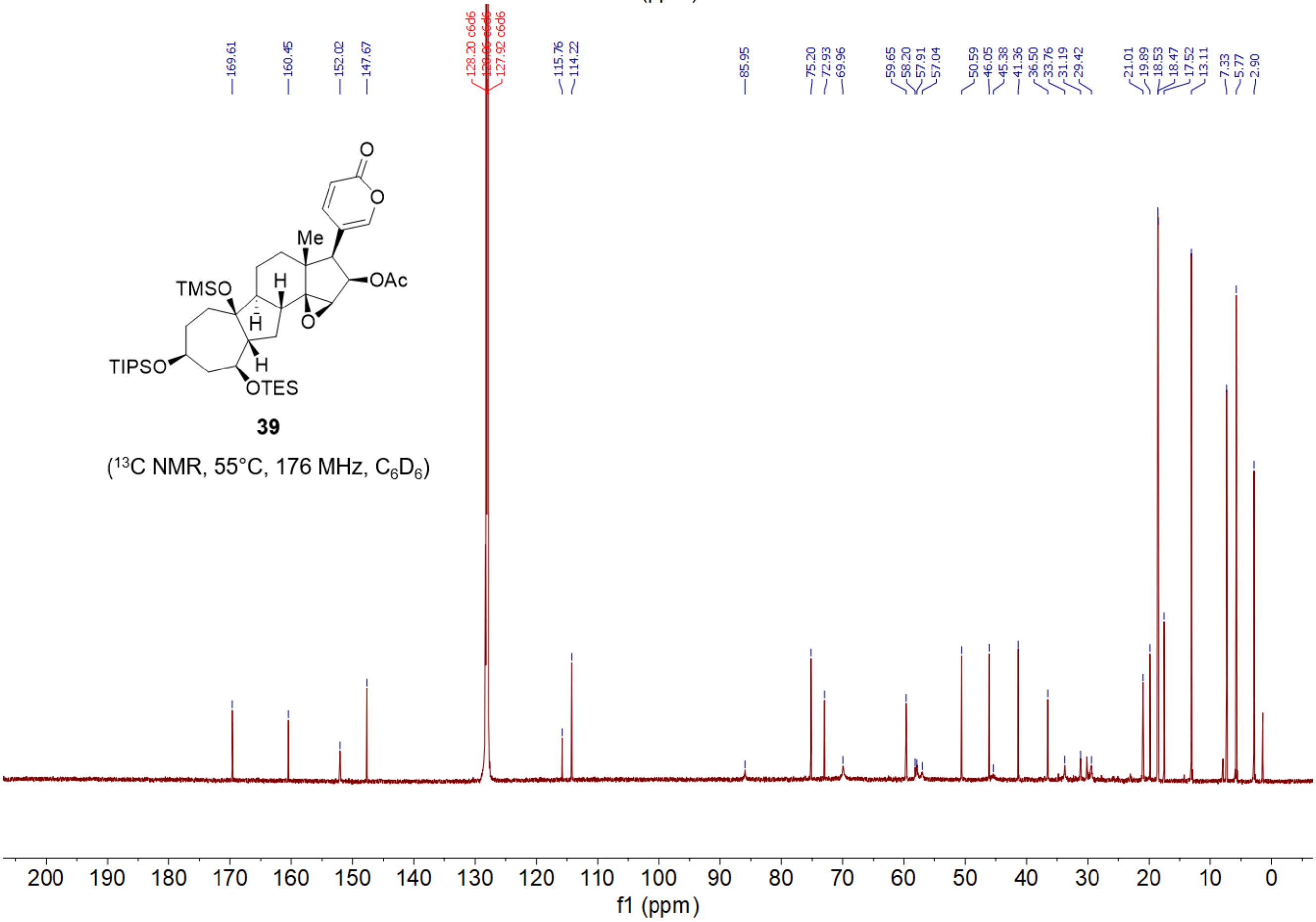

39

(^{13}C NMR, 55°C, 176 MHz, C_6D_6)

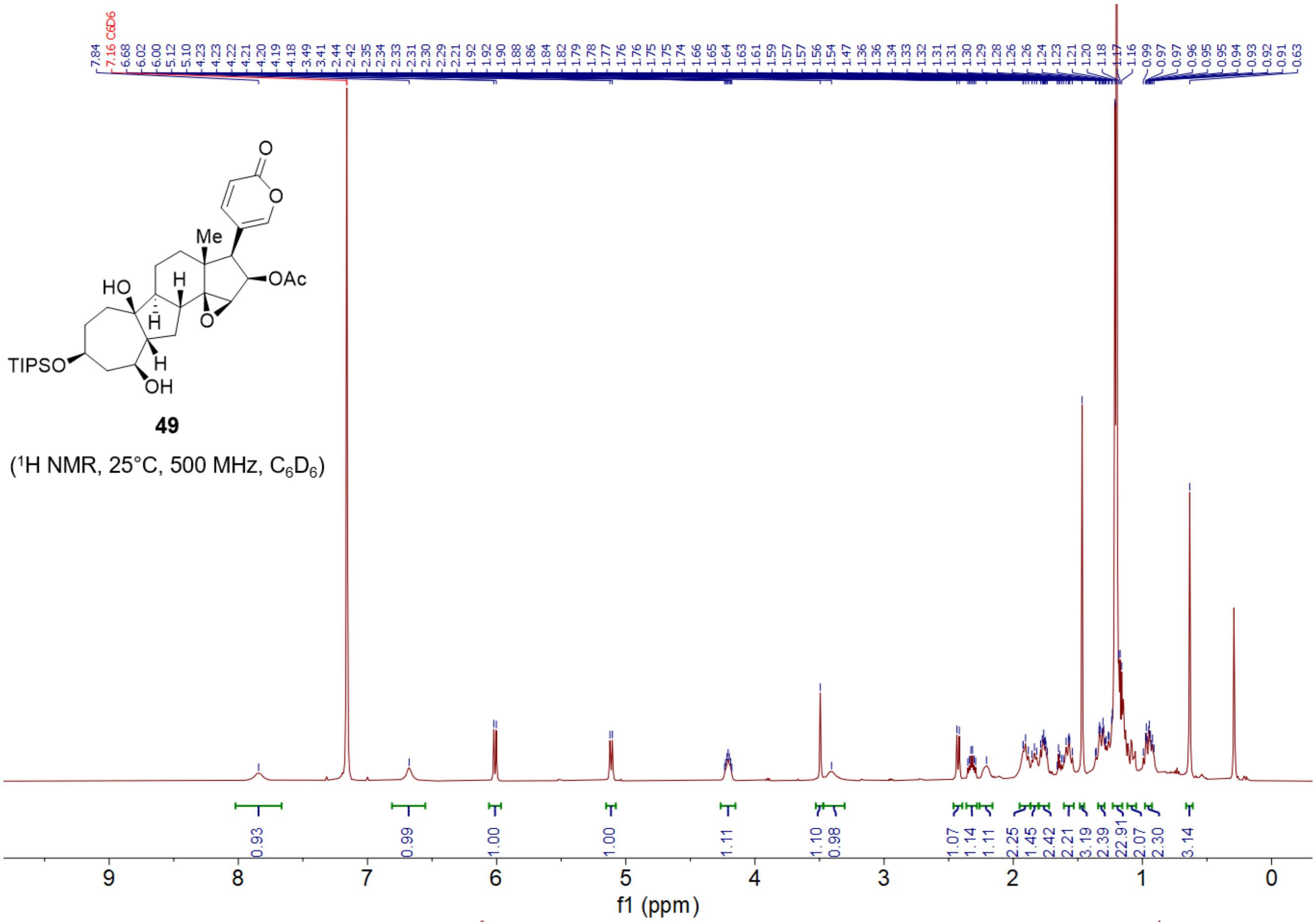

Me
HO
H
OAc
TIPSO
OH
49
(1H NMR, 25°C, 500 MHz, C6D6)
f1 (ppm)

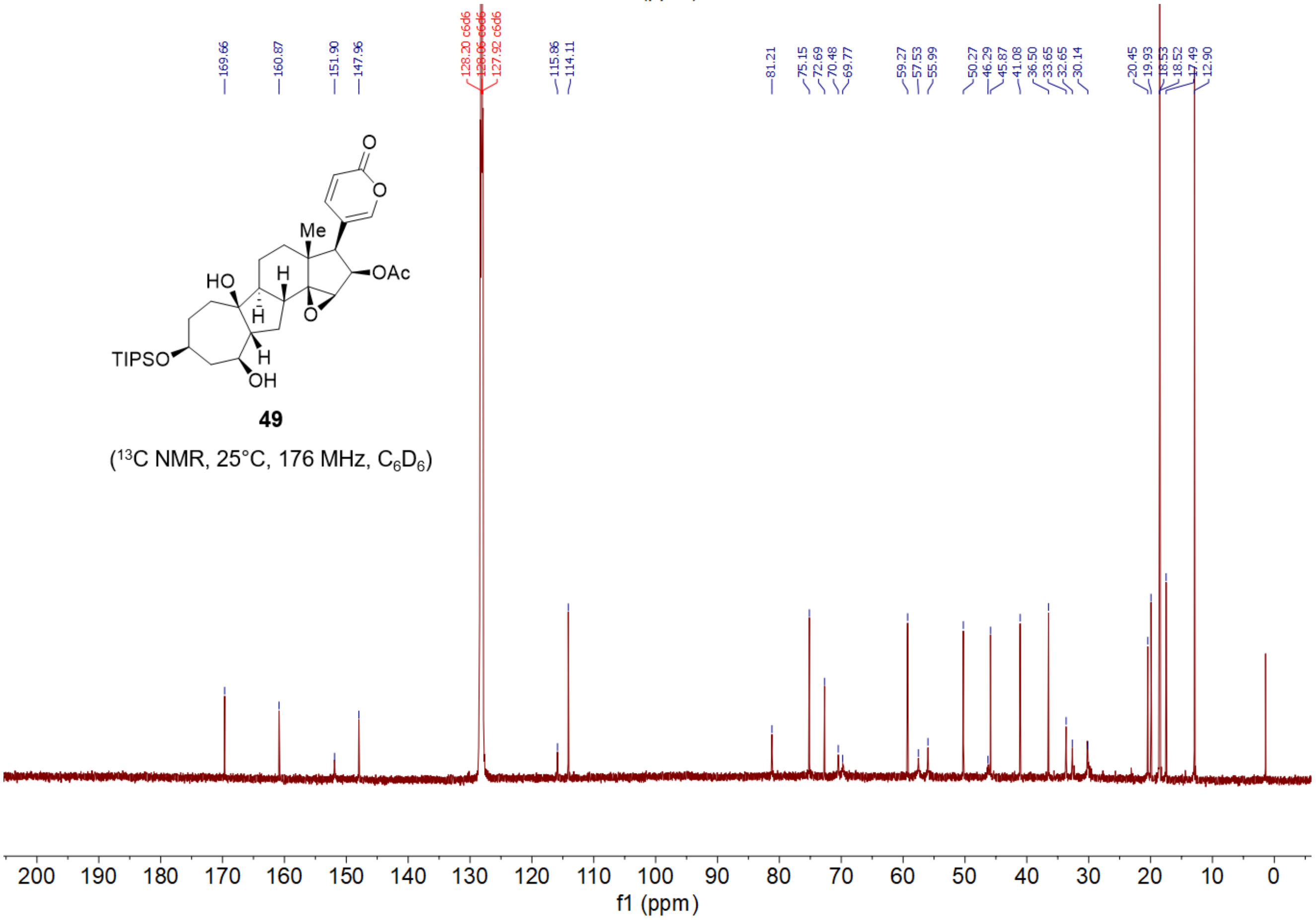

Me
HO
H
OAc
TIPSO
OH
49
(13C NMR, 25°C, 176 MHz, C6D6)
f1 (ppm)

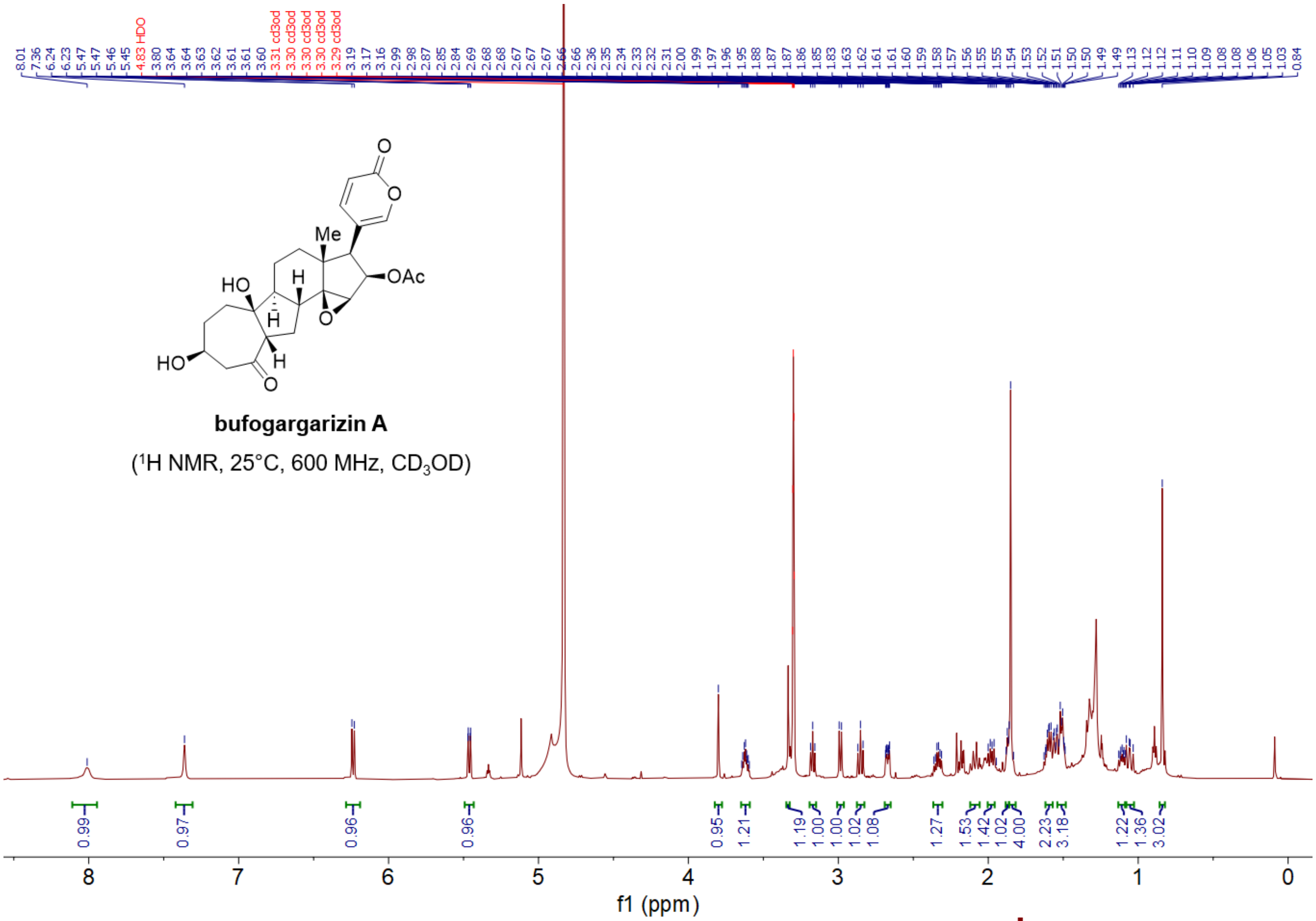

bufogargarizin A

(^{1}H NMR, 25°C, 600 MHz, CD_3OD)

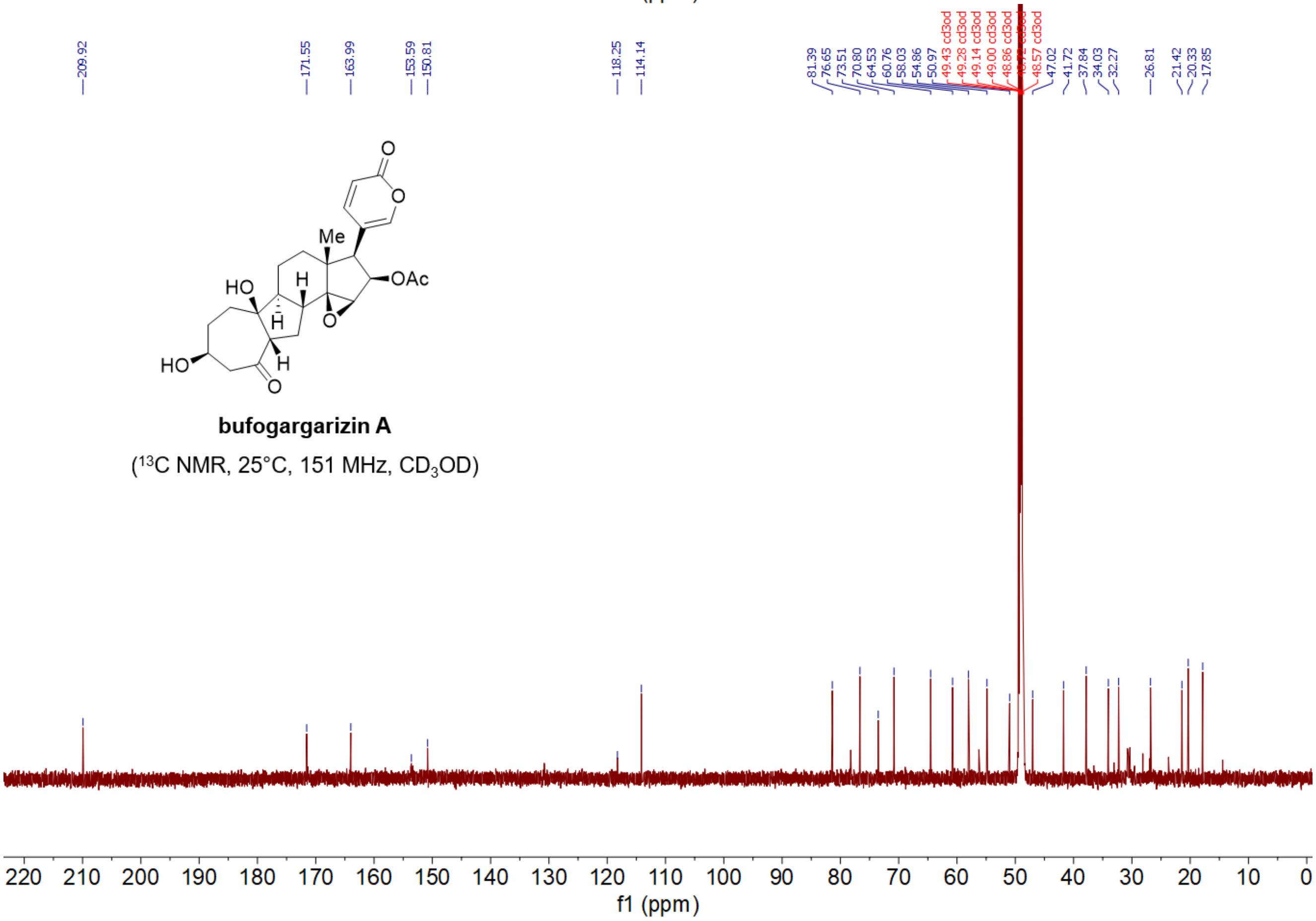

bufogargarizin A

(^{13}C NMR, 25°C, 151 MHz, CD_3OD)

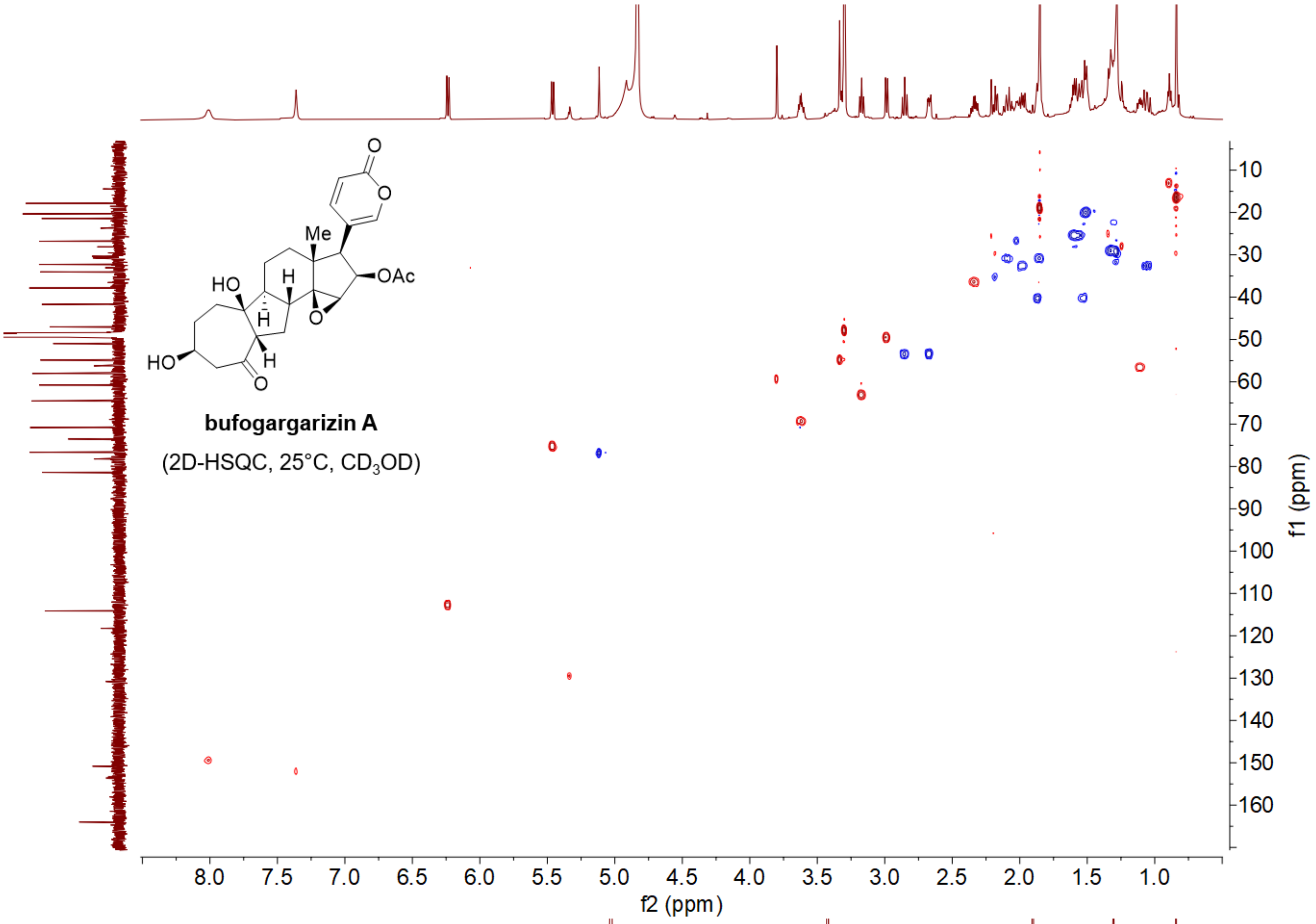

O
O
Me
OAc
HO
H
H
O
HO
H
O
bufogargarizin A
(2D-HSQC, 25°C, CD$_3$OD)
f1 (ppm)
f2 (ppm)

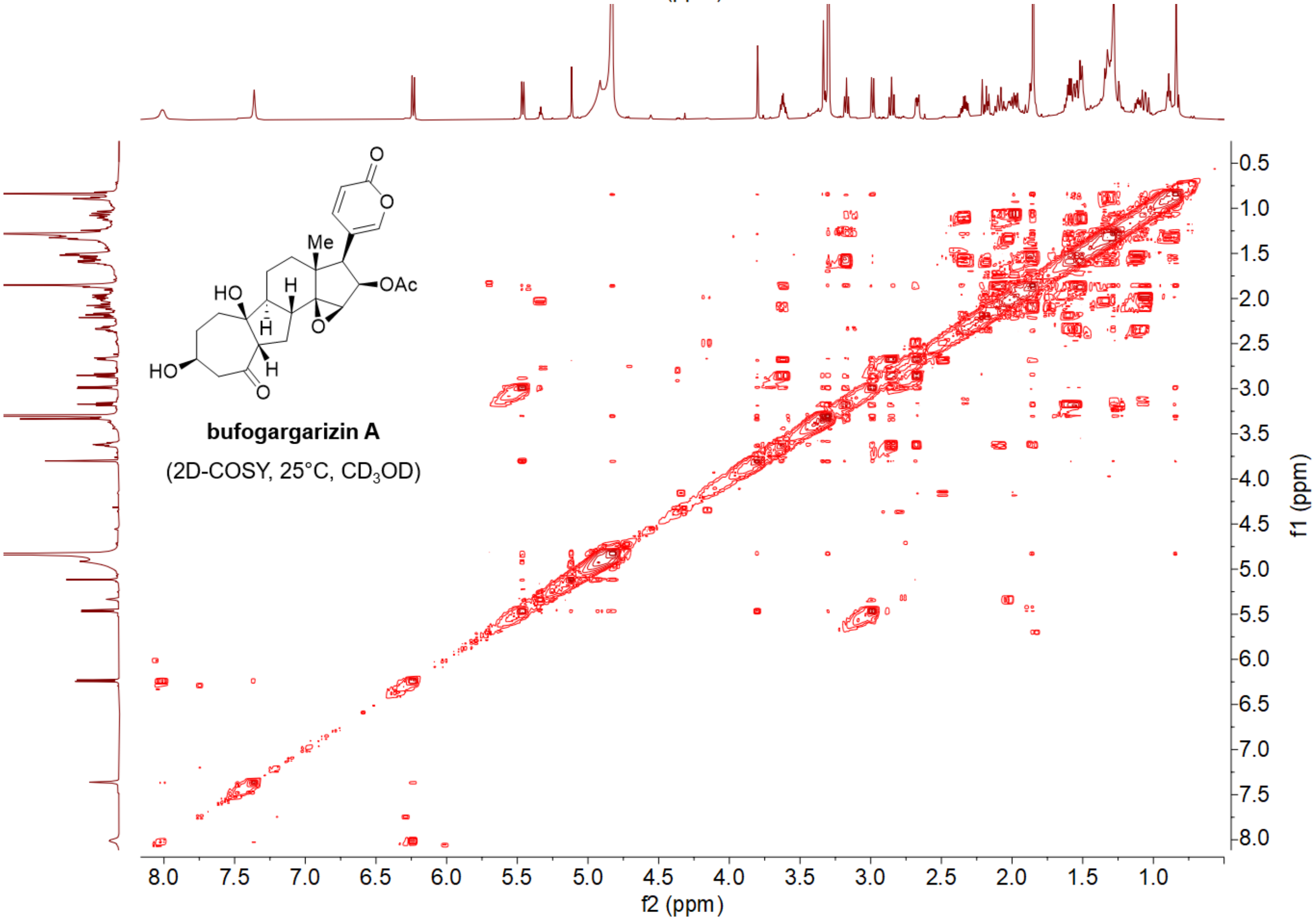

O
O
Me
OAc
HO
H
H
O
HO
H
O
bufogargarizin A
(2D-COSY, 25°C, CD$_3$OD)
f1 (ppm)
f2 (ppm)
